# Full-length transcriptome sequencing reveals the molecular mechanism of monoterpene and sesquiterpene biosynthesis in *Cinnamomum burmannii*

**DOI:** 10.3389/fgene.2022.1087495

**Published:** 2023-01-06

**Authors:** Chen Hou, Qian Zhang, Peiwu Xie, Huiming Lian, Yingli Wang, Dongcheng Liang, Yanling Cai, Boxiang He

**Affiliations:** Guangdong Provincial Key Laboratory of Silviculture, Protection and Utilization, Guangdong Academy of Forestry, Guangzhou, Guangdong, China

**Keywords:** full-length transcriptome, terpenoid synthase, lncRNA, leaf anatomy, *Cinnamomum burmanii*

## Abstract

Essential oil of *Cinnamomum burmannii* is rich in monoterpenes and sesquiterpenes and is widely used in cosmetics and medicines. Knowledge about the enzymes that catalyze the formation of monoterpenes and sesquiterpenes in *C. burmannii* is insufficient. Therefore, anatomy observation of *C. burmannii* at the four developmental stages (7 days, CBS1; 14 days, CBS2; 21 days, CBS3, and 28 days, CBS4) were conducted to elucidate the origins of essential oil production. Twelve full-length transcriptomes of *C. burmannii* leaves at the four stages were generated using Oxford Nanopore Technologies. GC-MS analysis revealed 15 monoterpene and sesquiterpenes dramatically increased from CBS1 to CBS4. A weighted correlation network analysis (WGCNA) in association and differentially expressed genes across four developmental stages were performed. A total of 44 differentially expressed genes (DEGs) were involved in terpenoid syntheses during leaf development. Among them, the DEGs of the mevalonate acid (MVA) pathway were predominantly expressed at CBS1, while those of the 2-C-methyl-D-erythritol 4-phosphate (MEP) pathway showed increased expression from CBS2 to CBS4. Besides, fourteen genes were associated with monoterpene synthesis and nine with sesquiterpene synthesis. Functions of these DEGs were further predicted with regard to gene expression profile and phylogenetic relationship with those characterized in previous studies. In addition, 922 long noncoding RNAs (lncRNAs) were detected, of which twelve were predicted to regulate monoterpene and sesquiterpene biosynthesis. The present study provided new insights the molecular mechanisms of monoterpenoid and sesquiterpenoid syntheses of *C. burmannii*.

## Introduction

**FIGURE 1:**
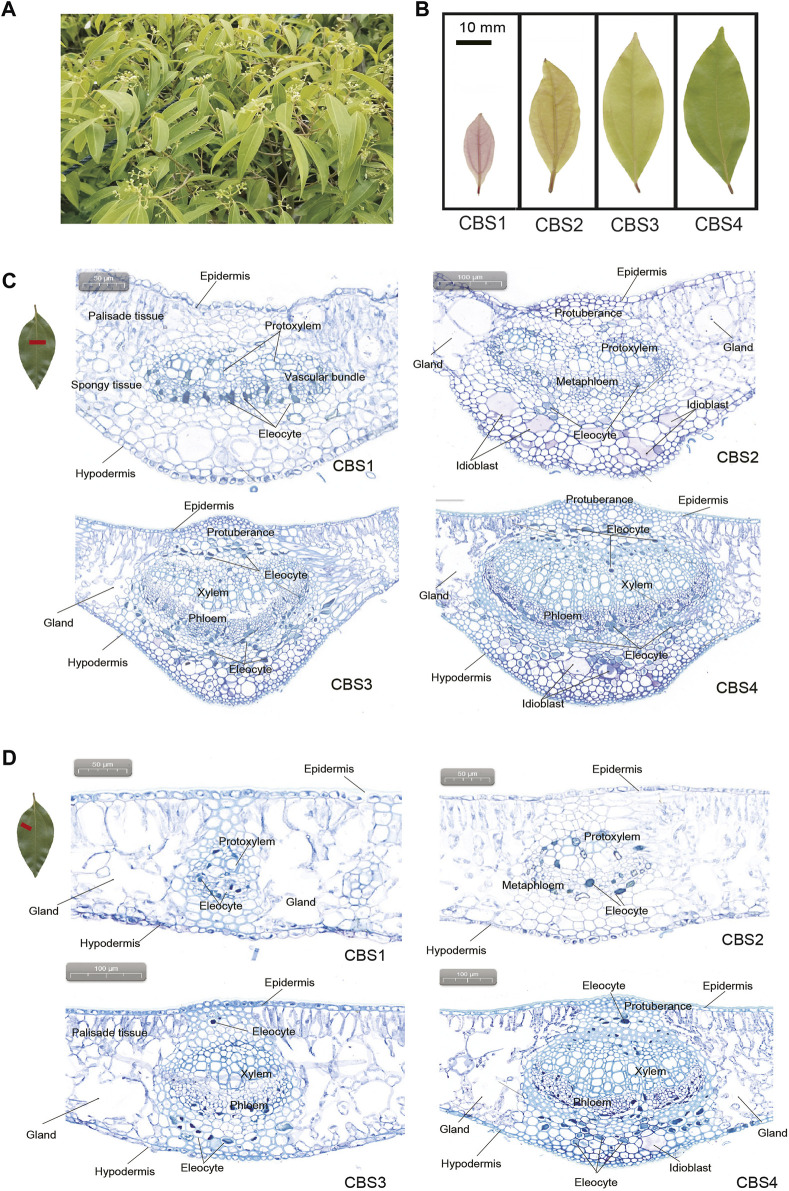
Gross morphology and anatomy of *Cinnamomum burmannii* leaves. **(A)** Six-year-old *C. burmannii* plant. **(B)** Leaves at four developmental stages (CBS1‒CBS4). **(C)** Anatomy and histology of the main vein of *C. burmannii* leaves at four developmental stages. **(D)** Anatomy and histology of the lateral vein of *C. burmannii* leaves at four developmental stages.

*Cinnamomum burmannii* (Nees & T. Nees) Blume, belonging to the Lauraceae family, is a broad-leaf tree species in Southeast of China, Indonesia and Philippines (Al-Dhubiab 2012; Shan et al., 2020) (Figure 1A). The stems and leaves of *C. burmannii* contain various volatile compounds, such as monoterpenes, sesquiterpenes, and other aromatic compounds (Ding et al., 1994). Based on chemical polymorphisms of dominant monoterpenes in leaves, *C. burmannii* are classified into five chemotypes: borneol-type, 1,8-cineole-type, camphor-type, terpinen-4-ol-type, and alpha-terpineol-type (Ji et al., 1991; Wu et al., 2020). Besides, leaves of *C. burmannii* are rich in various sesquiterpenes, e.g., caryophyllene, copaene, germacrene D and phellandrene (Yang et al., 2020; Ma et al., 2021). Among these compounds, d-borneol is permeable to human skin and possesses antibacterial and antimicrobial properties (Shan et al., 2007; Chen L et al., 2011; Al-Dhubiab 2012). Thus, the essential oils derived from *C. burmannii* leaves, especially the borneol-type are an important raw material for cosmetic and medical use. Studies have demonstrated the anatomy of *C. camphora* and *C. longepaniculatum* leaves (Tian et al., 2021; Zhao et al., 2022); however, the physiological mechanisms regulating the biosynthesis of d-borneol and other sesquiterpenes during *C. burmannii* leaf development remain unknown.

Monoterpenes and sesquiterpenes are synthesized from the five carbon units dimethylallyl pyrophosphate (DMAPP) and isopentenyl pyrophosphate (IPP) (Degenhardt et al., 2009; Chen F et al., 2011). The DMAPP and IPP are formed *via* the mevalonate acid (MVA) pathway and the 2-C-methyl-D-erythritol 4-phosphate (MEP) pathway in all land plants (Vranová et al., 2013; Tholl 2015). Several researchers have analyzed the expression of upstream genes of the MVA and MEP pathways in different vegetative and reproductive organs (Yang et al., 2020) and different d-borneol chemotypes (Li et al., 2022). However, the role of MVA and MEP pathways during *C. burmannii* leaf development has not been investigated. Typically, isopentenyl-diphosphate isomerases (IDI) isomerize IPP to DMAPP, which together produce geranyl diphosphate (GPP) and farnesyl diphosphate (FPP), the precursors of monoterpenoids. So far, six trans-IDI genes of *C. camphora* have been characterized (Yang et al., 2021), while those of *C. burmannii* remain ignored. Subsequent steps in the biosynthesis of monoterpenes and sesquiterpenes involve the action of terpene synthases (TPS), which have been reported in *C. burmannii* (Yang et al., 2020; Li et al., 2022; Ma et al., 2022), *C. camphora* (Zuo et al., 2017; Chen et al., 2018; Hou et al., 2020; Tian et al., 2021), *C. kanehirae* (Chaw et al., 2019)*, C. osmophloeum* (Lin et al., 2014), *C. porrectum* (Qiu et al., 2019), and *C. ternuipilum* (Yang et al., 2005). However, knowledge of the TPS genes associated with monoterpenes and sesquiterpenes of *C. burmannii* is limited. Moreover, the regulation of MVA and MEP pathways in *C. burmannii* remains elusive.

Recent studies have correlated long noncoding RNAs (lncRNAs) with terpene synthesis in angiosperms, such as *Citrus limon* (Bordoloi et al., 2022), *Gardenia jasminoides* (Shen et al., 2022), and *Zanthoxylum armatum* (Liu et al., 2022), and gymnosperms, such as *Ginkgo bioloba* (Han X et al., 2021) and *Pinus massoniana* (Feng et al., 2022). A previous study reported that lncRNAs regulate cineole, linalool, and nerolidol biosynthesis in *C. camphora* (Ni et al., 2021). It is clear that the lncRNAs are non-protein coding sequences involved in transcriptional and post-transcriptional regulation of gene expression in plants (Liu et al., 2015; Yuting et al., 2022). However, their significance in regulating monoterpene and sesquiterpene biosynthesis has not been reported in *C. burmannii*. The advent of ONT (Oxford Nanopore Technologies) sequencing has allowed the generation of the full-length transcriptome assisting the quantification of gene expression and detection of key regulatory elements (lncRNAs, alternative splicing, and alternative polyadenylation) (Hou et al., 2021a; Hou et al., 2021b). Compared to Pacbio technology used in the previous studies, ONT sequencing generates more accurate data at the transcription level (Cui et al., 2020). Therefore, this technique is expected to give an accurate idea about the prediction and targets of lncRNAs in *C. burmannii*.

Therefore, the present study used ONT sequencing to generate the full-length transcriptomes of *C. burmannii* leaves at four developmental stages. We performed the differentially expressed genes (DEGs), linked the gene expression with the results of GC-MS using weighted correlation network analysis (WGCNA), and investigated the differentially expressed lncRNAs and transcripts to identify the genes involved in the monoterpene and sesquiterpene synthesis across the four stages. Thus, the study’s findings will help elucidate the molecular mechanisms regulating secondary metabolism in *C. burmannii*.

## Materials and methods

### Sample collection and preparation

Leaf materials of the borneol-type *C. burmannii* were collected from a six-year-old plant (Figure 1A) in the Tianluhu Forestry Park, Guangzhou, China, which permitted sample collection. Leaves at four developmental stages, including 7 days (CBS1), 14 days (CBS2), 21 days (CBS3), and 28 days (CBS4) (Figure 1B), were collected. For each developmental stage, three replicates were prepared, resulting in a total of 12 samples. The samples were immediately stored in a -80°C freezer and used for leaf transverse sectioning, GC-MS analysis, ONT sequencing, and qRT-PCR analysis.

### Anatomic analysis of *C. Burmannii* leaves

The *C. burmannii* leaf samples were soaked in a formaldehyde-acetic acid-alcohol mixture (FAA) for 48 h and subsequently embedded in paraffin wax. Then, 8 μm thick transverse sections were obtained from these paraffin blocks using a rotary microtome (Leica, RM2265, Germany). The freshly cut sections were dried on a slide drier (KEDEE, KD-P, US) at 40°C and stained with the safranin O-fast green (Getzy et al., 1982). The prepared sections were observed using a light microscope (Nikon TE2000-U, Nikon, Japan).

### Terpenoid extraction and GC-MS analysis

The leaf samples were ground into a powder with liquid nitrogen and dissolved in 4 ml of hexyl hydride, ethanol, and ethyl acetate mixture (2:1:1). The organic solution containing the tissue powder was treated with ultrasonic waves at 20 kHz for 20 min and incubated in a water bath at 56°C for 30 min. After centrifugation, the supernatant was collected, mixed with cyclohexanone (Sigma-Aldrich, Shanghai, China), and analyzed on a gas chromatograph-mass spectrometer (GC-MS) with the SH-Rxi-5Sil.MS quartz capillary column (30 m × 0.25 mm × 0.25 µm). To assess relative content of d-borneol with GC-MS, the initial column temperature was set at 70°C, then raised to 160°C at a rate of 2°C/min, maintained for 2 min, then to 220°C at a rate of 10°C/min, and maintained for 5 min; it took 51 min for the warming program to detect one sample. The ionization temperature was set at 200°C, the interface temperature at 250°C, and the scanning range (m/z) at 50–500. Besides, to measure absolute content of d-borneol, 200 mg of standard d-borneol (purity ≥99%, Sigma-Aldrich, Shanghai, China) were measured and transferred into a 10 ml volumetric flask, added with cyclohexanone to the constant volume, yielding 20 mg/ml of d-borneol standard solution. 0.005, 0.025, 0.1, 0.5, and 2.5 ml of the above solution was transferred into five 10 ml volumetric flasks, correspondingly. Cyclohexanone was added to each constant volume, yielding 10, 50, 200, 1,000, and 5,000 mg/L of d-borneol standard solution. 1 ml of each sample was transferred into the injection bottle for GC-MS testing. Under the same GC-MS conditions shown above, the absolute content of borneol in the leave samples were calculated according to the linear equation.

### RNA extraction and nanopore sequencing

Total RNA was extracted from the leaf samples using a RNeasy Plant Mini Kit (Qiagen, Valencia, CA, United States, product No. 74903). After removal of the relic using RNase-free DNase (Qiagen), the quality of the RNA sample was assessed using 1% agarose gel electrophoresis, a NanoDrop spectrophotometer (ThermoFisher Scientific, Wilmington, DE, United States), and an Agilent 2,100 Bioanalyzer (Agilent Technologies, Palo Alto, CA, United States). The RNA was then reverse-transcribed to cDNA according to the protocol by Oxford Nanopore Technologies (Jain et al., 2016). The sequencing libraries were created using a preparation kit (Ultra-Long, Grandomics, Wuhan, China) by fragmenting the cDNA and adding PCR adapters to both ends. After PCR amplification (14 cycles) with LongAmp Taq (NEB, New England Biolabs LTD., Beijing, China), the ONT adaptors were ligated onto the PCR products using T4 DNA ligase (NEB). The ligated PCR products were sequenced on a MinION Mk1B sequencer (Oxford Nanopore, Oxford, United Kingdom). All sequencing data have been deposited in the NCBI Sequence Read Archive (SRA) under the data have been released from the SRA database since November 23, 2022.

### Genome mapping and annotation

Raw sequencing data were quality filtered to remove the adapter sequences, reads with quality scores ≤7, and reads with length ≤500 bp using MinKNOW (version 2.2) (Oxford, United Kingdom). Reads similar to ribosomal RNA were also deleted by searching against the Silva rRNA database (https://www.arb-silva.de). We then identified the full-length reads and clustered them after mapping to the *C. burmannii* reference genome (Hou et al. unpublished) using minimap2 (version 2.16) with the setting (-t 6 -ax splice--split-prefix -uf--secondary = no) (Li 2018). Each cluster of full-length reads was collapsed to obtain a consensus sequence compared with the *C. burmannii* reference genome using pinfish (https://github.com/nanoporetech/pinfish, version 0.1.0) in the minimap2 software. The full-length reads with a minimum coverage of 85% and a minimum identity of 90% were saved using the cDNA_Cupcake package (version 5.80), and the remaining redundant reads were deleted. The full-length reads with different 5′ ends were not considered redundant.

### Gene expression analysis and DEG identification

Gene expression levels were quantified using the non-redundant full-length reads by calculating the CPM (counts per million) values as follows: CPM = reads mapped to transcripts/total reads aligned to one sample × 1,000,000. The differential expression analysis was performed using the DESeq2 R package (version 1.10.1) to identify the genes differentially expressed between two developmental stages (Love et al., 2014). Genes with fold change ≥2 and false discovery rate (FDR) < 0.01 were defined as the differentially expressed genes (DEGs). Adjusted *p*-values with the Benjamini–Hochberg approach were used to assess the DEGs accurately. Further, the gene ontology (GO, http://www.geneontology.org) annotation and Kyoto Encyclopedia of Genes and Genomes (KEGG, http://www.genome.jp/kegg/) enrichment analysis were performed for the DEGs. Genes enrichment were performed using the Fisher’s exact test.

### Weighted correlation network analysis

After GC-MS analysis, a trait matrix of based on relative content was prepared for the most 10 abundant monoterpenoids and sesquiterpenoids (Supplementary Table S1). Then, the correlation of co-expressed genes and these metabolites was analyzed in a weighted correlation network analysis (WGCNA) implemented in the R package WGCNA (version 1.42) (Langfelder and Horvath 2008). A hierarchal clustering tree based on the co-expressed genes was constructed using the Dynamic Tree Cut R package (Langfelder et al., 2008). Genes significantly correlated with the abundance of the ten terpenoids were grouped into different modules and further annotated based on the KEGG database.

### Identification of coding sequences and lncRNAs

The coding sequences (CDS) of the non-redundant full-length reads were identified with TransDecoder based on log-likelihood score and open reading frames (ORFs) length. After searching against the CPC (Coding Potential Calculator) (Kong et al., 2007), CNCI (Coding-Non-Coding Index) (Sun et al., 2013), Pfam, and CPAT (Coding Potential Assessment Tool) (Wang et al., 2013) datasets, the remaining non-protein-coding reads with two exons and at least 200 nt long were identified as lncRNAs. These lncRNAs were further divided into four groups: lincRNA, antisense lncRNA, intronic lncRNA, and sense lncRNA. We further predicted the target genes regulated by the identified lncRNAs using LncTar (version 1.0) (Li et al., 2015). The lncRNA expression was quantified as CPM values, and the differentially expressed lncRNAs were identified with the setting (-p 2 -d -0.1 -s F). The target genes regulated by lncRNAs through *cis-* or *trans-*action were predicted separately using lncTar (Kung et al., 2013; Yang et al., 2014).

### The qRT-PCR validation

We selected nine DEGs (the *TPS* genes) for transcript expression validation using qRT*–*PCR. Primers were designed using the Primer Premier 5 software (Lalitha 2000). The information related to qRT-PCR is shown in Supplementary Table S2. The PCR was carried out using the following program: an initial denaturation at 95°C for 30 s, followed by 40 cycles of 10 s at 95°C, 30 s at 60°C, and 5 s at 95°C, and a final extension at 60°C for 5 s and 90°C for 5 s; the melting curves were drawn for every 0.5°C. The *Actin* gene was used as the endogenous control, and the relative expression levels were determined using the ΔΔCt method (Livak and Schmittgen 2001). Three replicates were maintained per sample, and the gene expression levels were represented as mean ± standard deviation. Differences of expression levels between CBS1 and the other three stages of *C. burmannii* leaves were analyzed by one-way analysis of variance (ANOVA), followed by an independent sample *t*-test with the R software. *p* values <0.05 was thought to be statistically significant.

### Sequence alignment and phylogenetic analysis

In order to predict functions of *TPS* genes, the sequences of the *TPS* genes identified in this study were aligned using MUSCLE (version v3.8.31) (Edgar 2004). The sequence alignment comprises the sequences of seven *TPS* genes reported earlier (*CbTPS1-S7* in Ma et al., 2021) and those mentioned by Yang et al., 2020 (see Supplementary_data18, DOI: 10.7717/peerj.9311/supp-18). Besides, the sequences of the *IDS* genes were also aligned, as mentioned by Yang et al., 2021 (See Supplementary Table S3). We performed multiple alignments for the *IDS* and *TPS* genes of *C. burmannii* using MUSCLE (version 3.8.31) (Edgar 2004). Then, RAxML-HPC2 (version 8.2.12) (Stamatakis 2014) implemented in CIPRES Gateway (version 3.3) (www.phylo.org) was used to construct two maximum likelihood (ML) trees, one for *IDS* genes and the other for *TPS* genes. Before the phylogenetic analysis, the GTR + CAT model was identified as the best-fit model for amino acid using jModeltest2 (version 2.1.6) (Darriba et al., 2012). A rapid bootstrapping was performed to search for trees with the highest score, and statistical support for the ML trees for *IDI* and *TPS* genes was derived from 1,000 pseudo-replicates of simulated bootstraps.

## Results

### Morphological and anatomical characters of *C. burmannii* leaves

The cross sections showed that *C. burmannii* leaves are mainly composed of epidermis, palisade tissues, spongy tissues, vascular bundles, and hypodermis (Figures 1C,D). Glands were found at the lateral sides of vascular bundles in both the middle and lateral veins. In addition, dramatic changes were observed in the anatomical structures of the main veins (Figure 1C) and lateral veins (Figure 1D) across four developmental stages of *C. burmannii* leaves. At the CBS1 and CBS2 stages, protoxylem and metaphloem were present in both the main and lateral veins. At CBS3 and CBS4, the xylem and phloem substituted protoxylem and metaphloem. Besides, protuberances, composed of parenchymatous cells, were found above the vascular bundles of the middle and lateral veins from CBS2 to CBS4 (Figures 1C,D). In addition, a few eleocytes, which produce essential oil, emerged in the peripheral areas of the vascular bundles in the middle and lateral veins at CBS1 and CBS2. Subsequently, numerous eleocytes were found at CBS3 and CBS4. Besides, some idioblasts emerged at CBS2 and more found at CBS4 in the middle vein (Figure 1C), while many were merely found in the lateral veins at CBS4 (Figure 1D).

### Terpenoid content at different developmental stages

**FIGURE 2:**
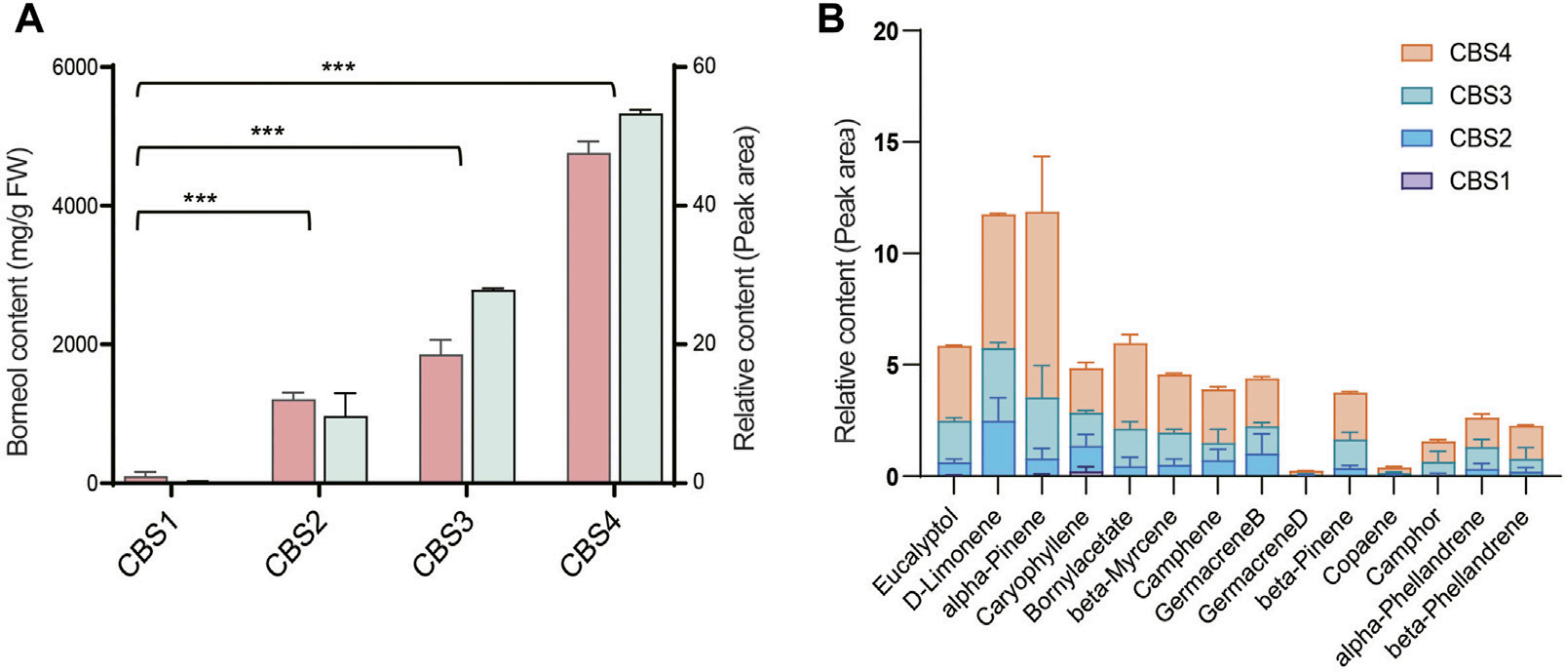
Terpenoid content of *Cinnamomum burmannii* leaves across four developmental stages. **(A)** The bars in salmon and pale turquoise indicate content of d-borneol (left *y*-axis, fresh weight—mg/g) and relative content of d-borneol (right *y*-axis, peak area—%), respectively. Symbol *** indicates the *p* values < 0.01 using Student’s *t*-test analysis. **(B)** The relative content of other monoterpenes and sesquiterpenes.

We further analyzed the secondary metabolites in the leaves of *C. burmannii* using GC-MS. The relative content of borneol significantly increased (*p*-value < 0.01) from CBS1 to CBS2 (Figure 2A). The relative content of d-borneol increased from 0.2 at CBS1 to 53.34 at CBS4, while the absolute content increased from 100.64 mg/g to 4762.67 mg/g. Other terpenoids in the *C. burmannii* leaves also increased from CBS1 and CBS4 (Figure 2B). We found the top 10 terpenoids from CBS2 to CBS4 were eucalyptol, d-limonene, alpha-pinene, caryophyllene, bornyl acetate, beta-myrcene, camphene, germacrene B, beta-pinene, and alpha-phellandrene.

### Processing of sequencing data

Sequencing on the ONT platform generated 2,341,602 to 3,941,015 raw reads per sample (Supplementary Table S3), with a mean length ranging from 988 to 1,228 bp and N50 length ranging from 1,092 to 1,329 bp. After removal of adapters and low-quality reads, we obtained 2,218,026 to 3,725,516 clean reads, comprising 1,935,584 to 3,316,638 full-length reads (Supplementary Table S4). Of this, 1,920,760 to 3,284,474 reads were mapped to the *C. burmannii* reference genome, with mapping ratios ranging from 98.72% to 99.35% (Supplementary Table S5). After collapsing each cluster, the data generated 23,587 to 37,176 consensus sequences, with N50 length ranging from 1,257 to 1,497 (Supplementary Table S6). Subsequently, after deleting the redundant reads, 11,615 to 20,320 non-redundant full-length transcripts were obtained, with N50 lengths ranging from 1,415 to 1700 bp and mean lengths ranging from 1,283 to 1,463 bp (Supplementary Table S6).

### Detection and annotation of differentially expressed genes

**FIGURE 3:**
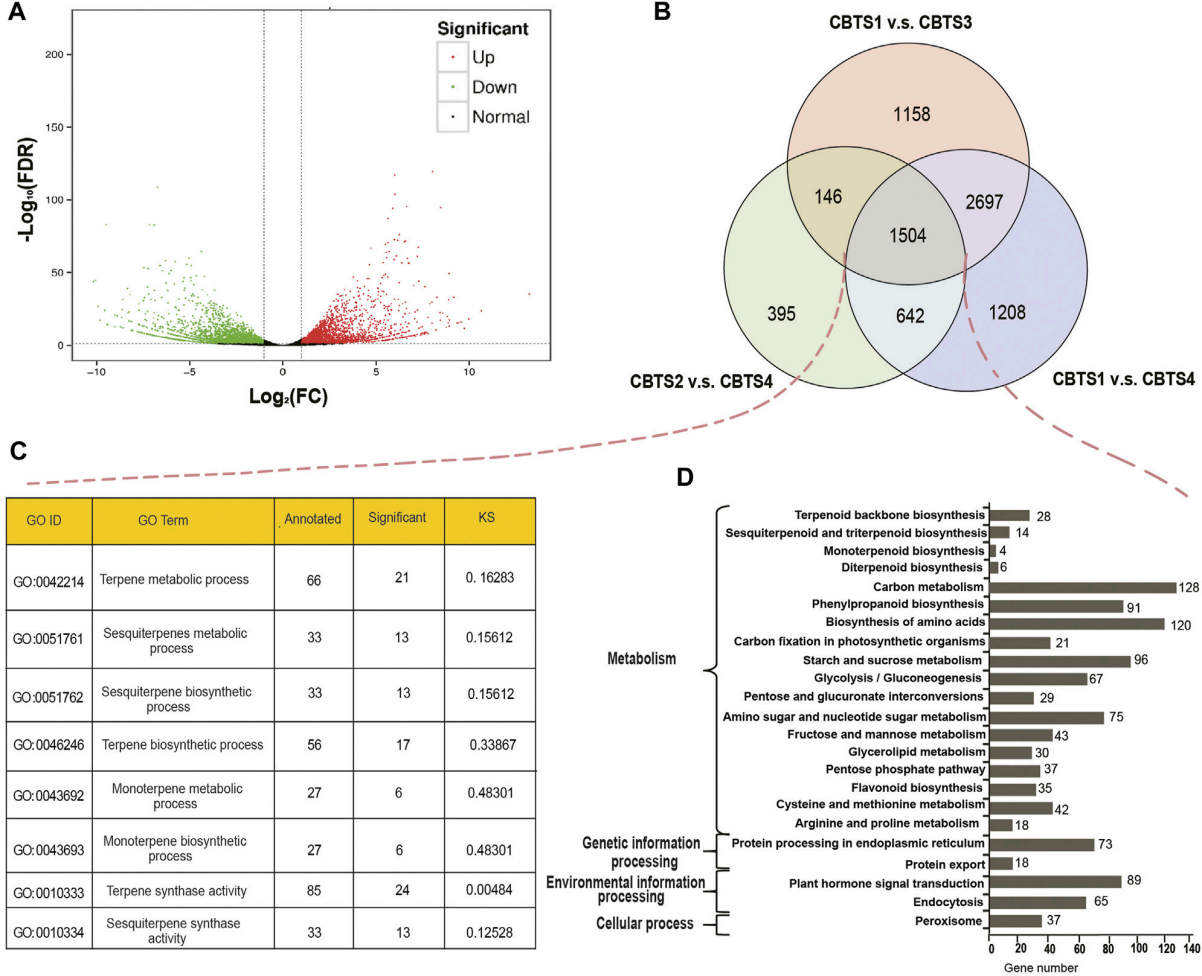
Differentially expressed genes (DEGs) of *Cinnamomum burmannii* leaves across four developmental stages and their annotations. **(A)** A volcano plot shows the number of DEGs between CBS1 and CBS4 in red (upregulated genes) and green dots (downregulated genes). **(B)** Venn diagram shows the overlap in DEGs. **(C,D)** GO enrichment analysis of the overlapping DEGs between CBS1 and CBS3, CBS2 and CBS4, and CBS1 and CBS4. **(D)** KEGG (Kyoto Encyclopedia of Genes and Genomes) enrichment analysis of the overlapping DEGs between CBS1 and CBS3, CBS2 and CBS4, and CBS1 and CBS4.

Further analysis identified 881 (CBS2 vs. CBS3) to 6,051 (CBS1 vs. CBS4) differentially expressed genes (Supplementary Table S8). Among the 6,051 DEGs between CBS1 and CBS4, 2,749 were upregulated, and 3,302 were downregulated (Figure 3A). Venn diagram showed that 1,504 DEGs were commonly shared among the three comparison groups (CBS1 and CBS4, 6,051 DEGs; CBS1 and CBS3, 5,505 DEGs; CBS2 and CBS4, 2,687 DEGs; Figure 3B). We then annotated these 1,504 DEGs based on GO and KEGG databases. GO annotation showed that 21 DEGs were involved in the terpene metabolic process (GO ID: 0,042,214), 24 in terpene synthase activity (GO:0010333), six in the monoterpene metabolic process (GO:0043692), and six in monoterpene biosynthetic process (GO:0043692) (Figure 3C). Meanwhile, KEGG annotation showed that 28 DEGs were involved in the terpenoid backbone biosynthesis (KEGG orthology number: ko00900), 14 in sesquiterpenoid and triterpenoid biosynthesis (ko00909), six in diterpenoid biosynthesis (ko00904), and four in monoterpenoid biosynthesis (ko00902).

### Weighted correlation network analysis

**FIGURE 4:**
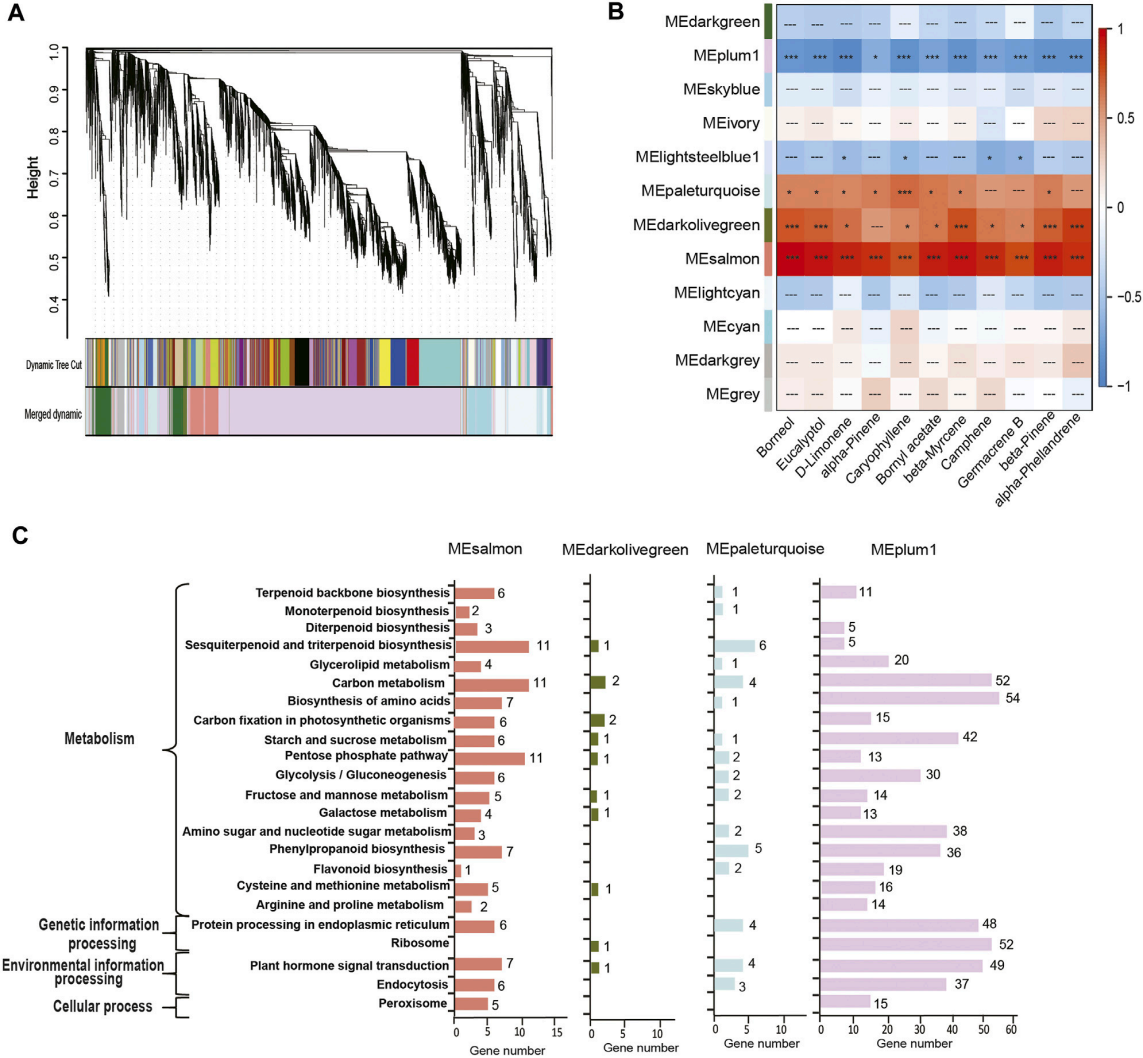
Weighted correlation network analysis of genes across four developmental stages in *C. burmannii*. **(A)** Hierarchical clustering of co-expressed genes shows the co-expression modules. Each branch in the phylogenetic tree corresponds to an individual gene, and the interconnected genes are grouped into twelve modules. **(B)** Heatmap based on gene expression patterns shows the module-metabolite relationships. The color bar shows the correlation between the twelve modules and the top 11 monoterpenes and sesquiterpenes; blocks were colored in red and blue indicate positive and negative correlations. Symbols “***“,”*”, and “---” represent highly significant (Fisher’s exact test; *p* < 0.01), significant (*p* < 0.05), and non-significant (*p* ≥ 0.05) correlations. **(C)** KEGG pathway analysis of the DEGs of the four modules significantly associated with the terpenoids.

We then performed a weighted correlation network analysis (WGCNA) for correlations between the contents of top 11 terpenoids and co-expressed genes involved in the biosynthesis of various terpenoids. A phylogeny of co-expressed genes indicates 12 modules (Figure 4A), of which four modules (represented by the colors plum, salmon, dark olive green, and pale turquoise) accounted for most co-expressed genes involved in the synthesis of the top 11 terpenoids (Figure 4B). The co-expressed genes belonging to the salmon module showed positive correlations with the top 11 terpenoids (*p* < 0.01), while those of the plum module showed negative correlations (*p* < 0.05; Figure 4B). Co-expressed genes in the dark olive-green module and pale turquoise module exhibited significantly positive correlations with a few exceptions (Figure 4C). KEGG annotation of the co-expressed genes in the four modules revealed that 18 genes (salmon-6, pale turquoise-1, and plum-11) were involved in terpenoid backbone biosynthesis, 3 (salmon-2 and pale turquoise-1) in monoterpenoid biosynthesis, and 23 (salmon-11, dark olive green-1, pale turquoise-6, and plum-5) in sesquiterpenoid and triterpenoid biosynthesis.

### Identification of open reading frames and LncRNAs

**FIGURE 5:**
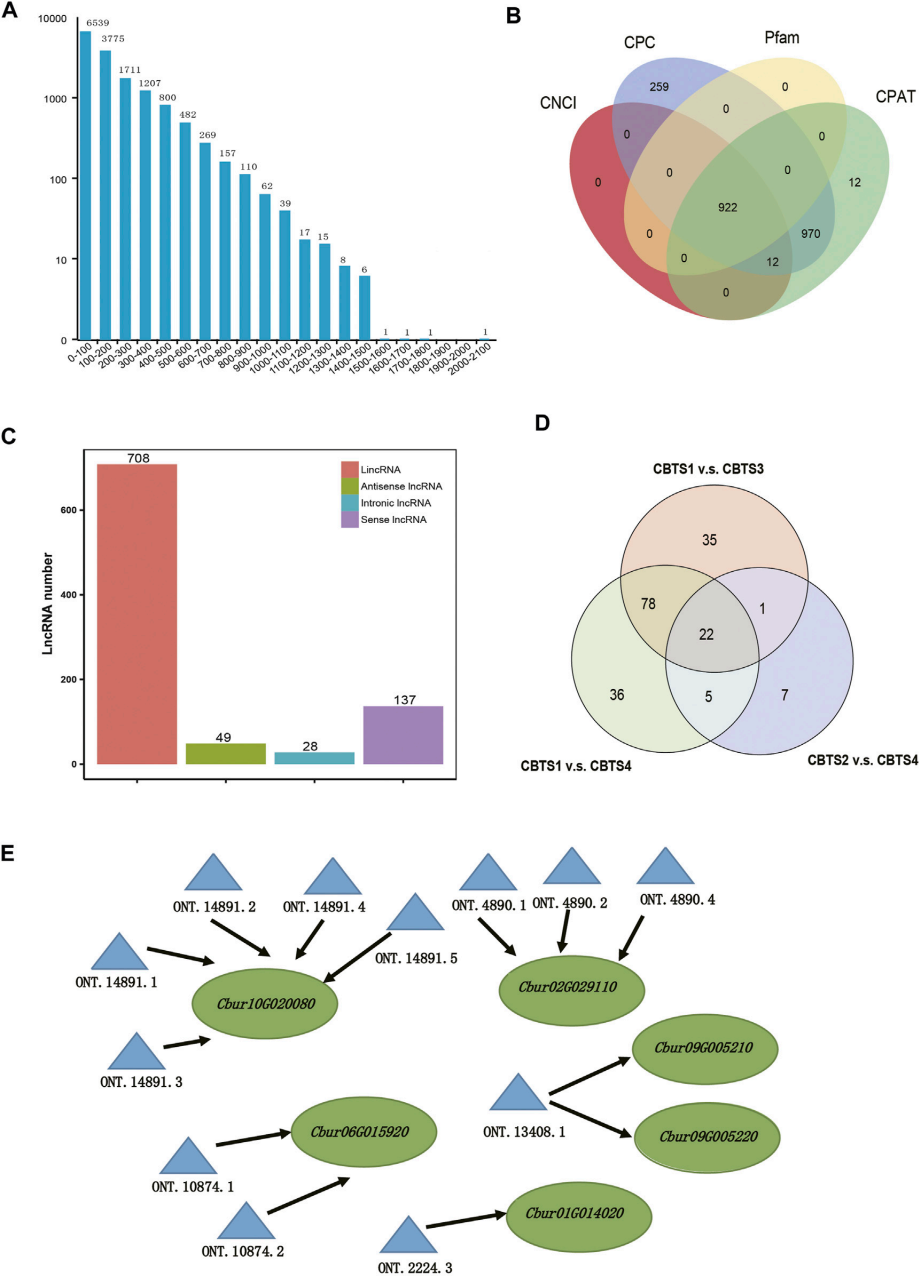
Analysis of the open reading frames (ORFs) and identification of lncRNAs based on 12 full-length transcriptomes of *Cinnamomum burmannii*. **(A)** Length distribution of ORFs detected in the full-length transcripts. **(B)** Venn diagram shows the number of lncRNAs filtered and identified using CNCI, CPC, CPAT, and Pfam. **(C)** Venn diagram shows the overlap in differentially expressed lncRNAs of *C. burmannii* leaves across the four developmental stages. **(D)** Solid lines represent the expression of *TPS* genes regulated in a *cis-*manner by the differentially expressed lncRNAs. **(E)** Representatives of predicted interaction networks among lncRNAs and their target genes. Solid lines represent the expression regulation by the lncRNAs in cis.

Further, 15,201 ORFs were identified from the non-redundant full-length reads, obtained by TransDecoder, of which the majority were shorter than 1,000 bp (Figure 5A). Data filtering based on the four databases (CNCI, CPC, Pfam, and CPAT) identified 922 lncRNA with at least two exons and longer than 200 nt (Figure 5B, Supplementary Table S9). These lncRNAs were further classified into four groups, including 708 lincRNAs (74.9%), 49 antisense lncRNAs (4.3%), 28 intronic lncRNAs (2.1%), and 137 sense lncRNAs (18.7%) (Figure 5C). In addition, a total of 22 differentially expressed lncRNAs were commonly shared among the abundant DEGs of the three comparison groups (CBS1 and CBS4, 141 lncRNAs; CBS1 and CBS3, 1,365 lncRNAs; CBS2 and CBS4, 35 lncRNAs) (Figure 5D). Further analysis using LncTar identified 3,337 *cis*-regulated target genes/full-length reads for the lncRNAs and 536 *trans*-regulated genes. These *cis-*regulated target genes included five involved in monoterpenoid synthesis regulated by 12 differentially expressed lncRNAs (Figure 5E).

### Detection of candidate genes involved in terpenoid synthesis

**FIGURE 6:**
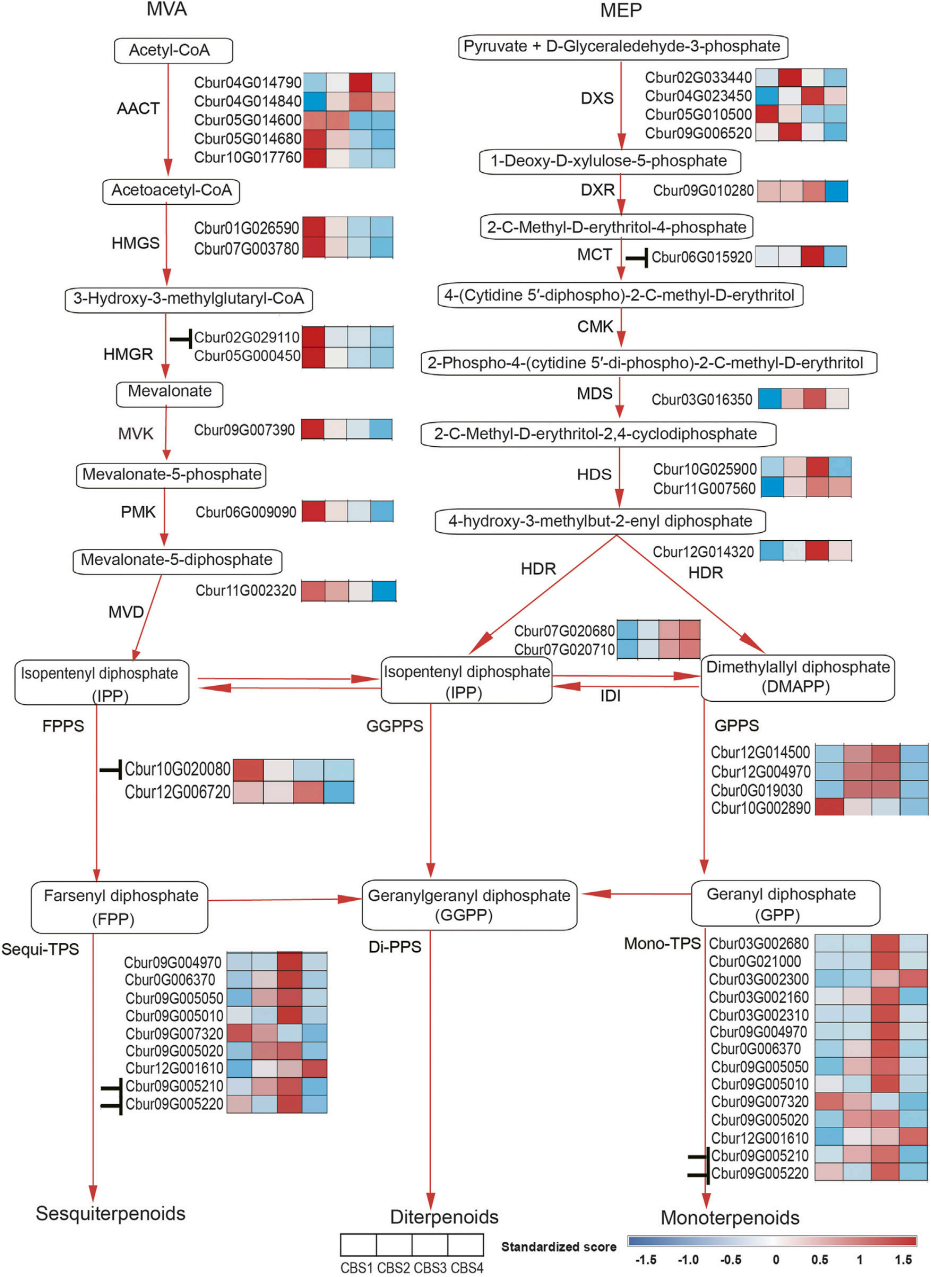
Differentially expressed genes (DEGs) of MVA and MEP pathways in *Cinnamomum burmannii*. Heatmaps show the expression levels of the DEGs involved in MVA and MEP pathways across four leaf developmental stages. Based on the target gene prediction using LncTar. The symbol“⊥” represents the regulation of gene expression by the lncRNAs detected in the present study.

**FIGURE 7:**
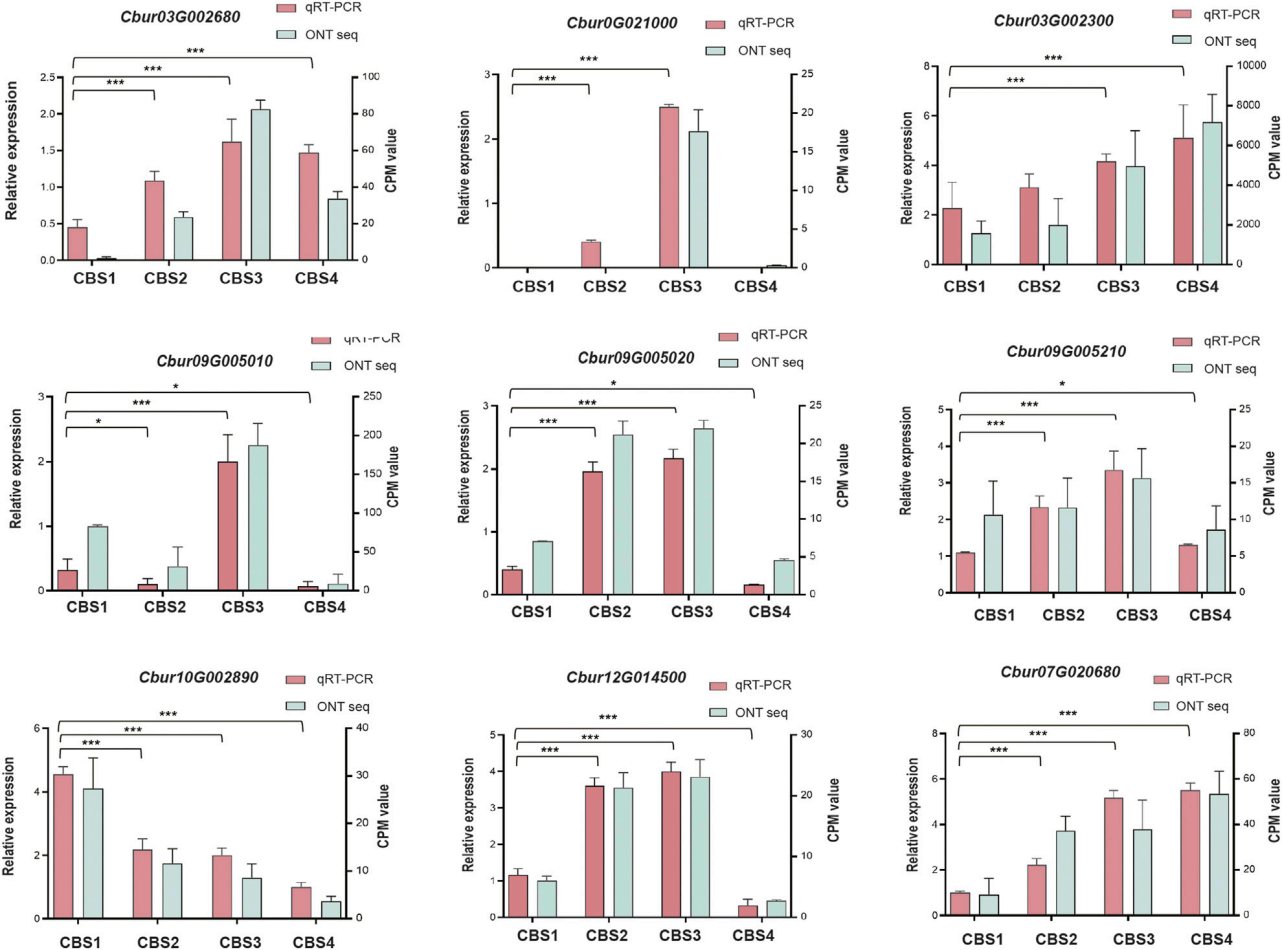
qRT-PCR validation of the expression patterns of nine selected genes. The qRT-PCR was performed using ACTIN as an internal control. Counts per million (CPM) values of Nanopore sequencing data are indicated on the left *y*-axis, and the relative expression levels based on qRT-PCR are indicated on the right *y*-axis. Symbol * and *** indicates the *p* values < 0.05 and values < 0.01 using one-way analysis of variance (ANOVA) and paired *t*-test analysis, respectively.

We further identified 44 DEGs (22—upstream and 22—downstream) associated with the molecular pathways regulating the synthesis of the top 10 monoterpenoids and sesquiterpenoids (Figure 6). The sequences of these DEGs are shown in Supplementary Table S10. Of the 44 DEGs detected, in the MVA and MEP pathways, our analysis identified more DEGs of the MVA pathway (12 genes) than the MEP pathway (ten genes). Moreover, the expression profiles of DEGs dramatically differed between the two pathways. The DEGs associated with the MVA pathway were highly expressed at CBS1 except for acetoacetyl-coenzyme A thiolase enzyme (AACT, *Cbur04G014790* and *Cbur04G014840*) while the DEGs in the MEP pathway showed high expression at CBS3 except for 1-deoxy-d-xylulose 5-phosphate synthase (DXS, *Cbur02G033440*, *Cbur05G01050*, and *Cbur04G023450*). Besides, two IDI genes, *Cbur07G020680* and *Cbur07G020710*, probably involved in inter-conventing between DMAPP and IPP, were highly expressed at CBS4. In the downstream, two DEGs were annotated as *FPPS* genes and four as *GPPS* genes, of which *Cbur10G020080* (a *FPPS* gene) and *Cbur10G002890* (a *GPPS* gene) were highly expressed at CB1, while the remaining four genes were highly expressed at CBS3. We found nine genes annotated as sesqui-*TPS*, which were highly expressed at CBS3 and CBS4, except for *Cbur09G007320*. Meanwhile, 14 genes were annotated as mono-*TPS* genes, of which 10 genes (*Cbur03G002680*, *Cbur0G021000*, *Cbur03G002160*, *Cbur03G002310*, *Cbur09G004970*, *Cbur0G006370*, *Cbur09G005050*, *Cbur09G005010*, *Cbur09G005210* and *Cbur09G005220*) showed high expression at CBS3, while *Cbur03G002300* and *Cbur09G007320* were highly expressed at CBS4 and CBS1, respectively. The high expression of the mono-terpene synthase genes at CBS3 are most likely to explain the increasing content of monoterpenoids and sesquiterpenoids from CBS2 to CBS4. We further validated the expression levels of nine *TPS* genes at four developmental stages using qRT-PCR are consistent with the gene expression generated by the ONT sequencing (Figure 7).

### Phylogenetic analyses of terpene synthases and isopentenyl-diphosphate isomerases genes

**FIGURE 8:**
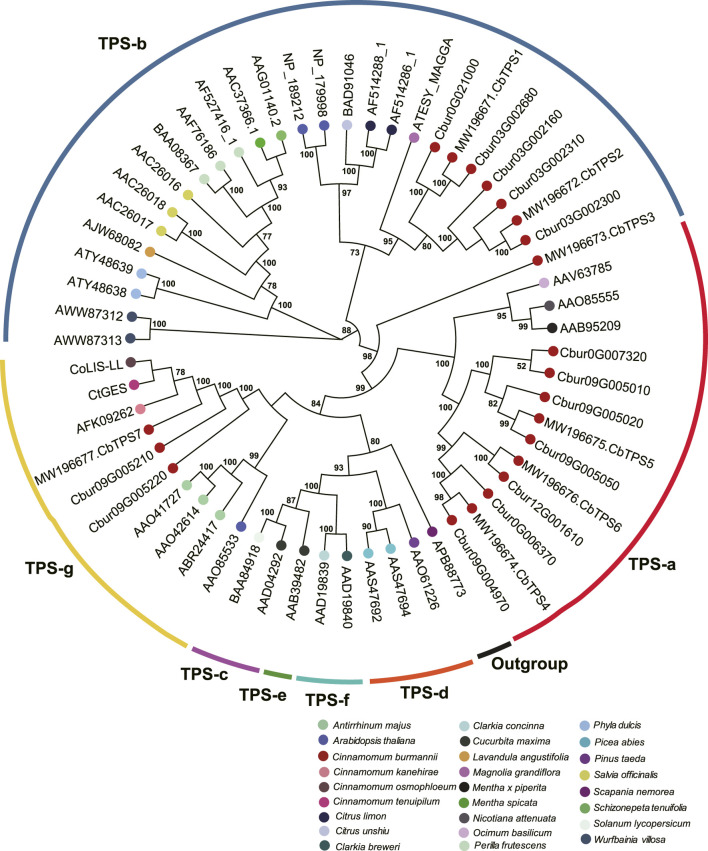
Phylogenetic relationship among the *TPS* genes of *Cinnamomum burmannii* established by the maximum likelihood method. Bootstrap values ≥ 50 are given at the nodes. The *TPS* genes of *C. burmannii* identified in the present study and other rereprentative *TPS* genes are indicated by the dots in different color. The accession information used for phylogenetic reconstruction is provided.

**FIGURE 9:**
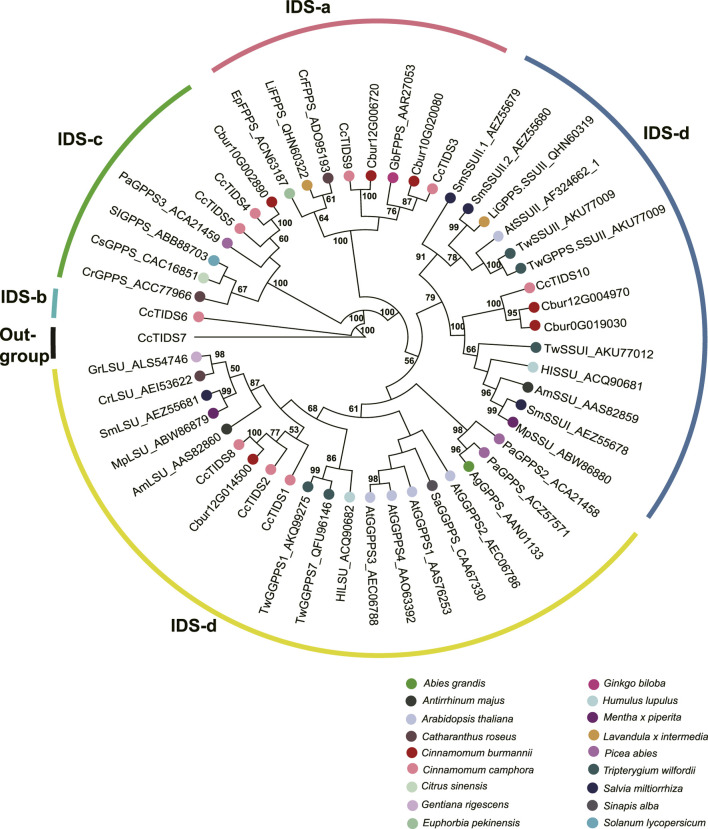
Phylogenetic relationship among the *IDS* genes of *Cinnamomum burmannii* established by the maximum likelihood method. Bootstrap values ≥ 50 are given at the nodes. The *IDS* genes of *C. burmannii* identified in the present study and other rereprentative *IDS* genes are indicated by the dots in different color. The accession information used for phylogenetic reconstruction is provided.

In order to characterize *TPS* and *IDI* genes identified in the present study, two phylogenetic trees were established using the maximum likelihood method. In the *TPS* tree, we found the 14 *TPS* genes were resolved into seven clades (Figure 8); seven were assigned to the TPS-a subfamily, five to the TPS-b subfamily, and two to the TPS-g subfamily. In the *IDS* tree, the six *IDI* genes were resolved into four clades (Figure 9); two each were nested within the two clades representing the IDS-a and IDS-d subfamilies. Meanwhile, *Cbur10G002890* alone was assigned to the *IDS*-c clade, while *Cbur12G014500* was assigned to the IDS-d clade.

## Discussion

Knowledge about leave anatomy in Lauraceae leaves is rare, let alone the observation of essential oil production during leave development. Previous studies have found eleocytes in the mature leaves of *C. camphora* (Zuo et al., 2017; Tian et al., 2021), which store and secrete essential oil (Zuo et al., 2017). Accordingly, the present study analyzed the leaf anatomic features to gain insight into essential oil accumulation during *C. burmannii* leaf development. We found a dramatic increase in the number of eleocytes from CBS3 to CBS4 (Figure 1C), indicating an increasing in the production of essential oil with leaf development. Besides, a few idioblasts were observed from CBS2 to CBS4 in the leaves of *C. burmannii*. Oil-secreting idioblasts have been also found in the leaves of *Scrophularia striata* (Amiri et al., 2011), *Laurus nobilis* (Serebrynaya et al., 2017), and *C. longepaniculatum* (Zhao et al., 2022), indicating the importance of idioblasts in the production of essential oil. Moreover, we found glandular structures flanking the middle veins of *C. burmannii* leaves, similar to that found in the mature leaves of *C. camphora* chemotypes (Tian et al., 2021). Although functions of these glandular structures remain unknown, few studies in peppermint and spearmint have indicated the role of glandular trichomes in synthesizing monoterpenes (Gershenzon et al., 1989; McCaskill et al., 1992). These observations collectively indicated an increasing of essential oil production with progress in leave development in *C. burmannii*.

Since d-borneol is dominant in the essential oil of borneol-type *C. burmannii*, bornyl diphosphate synthase is the key enzyme involved in the synthesis of borneol. A previous study shows that *CbTPS1* of *C. burmannii* encoding bornyl diphosphate synthase transform GPP to borneol both *in vitro* and in genetically modified *Saccharomyces cerevisiae* (Ma et al., 2021). Another study revealed higher expression of *CbTPS1* in the leaves of the high-borneol chemotype than those in the low-borneol chemotype (Li et al., 2022). The present study found two genes, *Cbur03G002680* and *Cbur0G021000*, closely related to *CbTPS1* in the TPS-b clade (Figure 8). Surprisingly, these two genes were strongly expressed at CBS3 but weakly at CBS4 (Figure 6). This result indicates that CBS3 is the key developmental stage for producing d-borneol in the *C. burmannii* leaves; the increased number of eleocytes around the middle and lateral veins of *C. burmannii* leaves at this stage corroborates this conclusion (Figures 1C,D). Meanwhile, the high d-borneol content detected at CBS4 is probably due to the accumulation starting from CBS3. Moreover, bornyl acetate and camphor are formed from borneol in the *C. burmannii* leaves (Ma et al., 2022). The increasing content of these two compounds from CBS1 to CBS3 could also be associated with the increase in the expression of *Cbur03G002680* and *Cbur0G021000.*

*CbTPS2* and *CbTPS3*, involved in the production of monoterpenes, belong to the TPS-b subfamily (Ma et al., 2022). In the present study, we found a close association of *Cbur03G002300, Cbur03G002160*, and *Cbur03G002310* with *CbTPS2* of the TPS-b subfamily clade (Figure 8). *Cbur03G002300* was strongly expressed at CBS4, while the other two were highly expressed at CBS3 (Figure 6). In addition, Ma et al. found that the enzyme CbTPS2 catalyzes the formation of alpha- and beta-pinene from GPP, while CbTPS3 were involved in the biosynthesis of linalool (Ma et al., 2022). Thus, the increase in the expression of *Cbur03G002300* from CBS2 to CBS4 and that of *Cbur03G002160* from CBS2 to CBS3 explain the rise in the relative content of alpha- and beta-pinene from CBS2 to CBS4 (Figure 2B).

Our study also found a significant increase in the content of two monoterpenes, D-limonene and beta-myrcene, and two sesquiterpenes, caryophyllene and germacrene B, from CBS1 to CBS4 (Figure 2B). Studies have associated the biosynthesis of these monoterpenes and sesquiterpenes with *CbTPS4*, *CbTPS5*, and *CbTPS6*, of the TPS-a subfamily (Ma et al., 2022). In the present study, seven genes belonged to the TPS-a clade, of which *Cbur09G004970* and *Cbur0G006370* were closely related to *CbTPS4*, *Cbur09G005050*, *Cbur09G005010*, *Cbur09G007320*, and *Cbur09G005020* to *CbTPS5,* and *Cbur12G001610* to *CbTPS6* (Figure 8). Among these, the expression levels of *Cbur0G006370*, *Cbur09G005050*, *Cbur09G005020*, and *Cbur12G001610* increased from CBS1 to CBS3, but dropped at CBS4; only *Cbur09G007320* showed a continuous decrease in expression from CBS1 to CBS4 (Figure 6). Genes *Cbur0G006370*, *Cbur09G005050*, *Cbur09G005020*, and *Cbur12G001610* are most likely to participate in top 10 the monoterpene and sesquiterpenes biosyntheses in the *C. burmannii* leaves at the four leave developmental stages.

The remaining two *TPS* genes, *Cbur09G005210* and *Cbur09G005220*, clustered close to *CbTPS7* of the TPS-g clade. Ma et al. demonstrated the role of *CbTPS7* in linalool (monoterpene) and nerolidol (sesquiterpene) syntheses (Ma et al., 2022). Meanwhile, *CoLIS-LL* was closely related to *CbTPS7* of the TPS-g clade in the *C. osmophloeum* leaves, which transforms GPP to linalool and FPP to nerolidol (Figure 8) (Lin et al., 2014). In the present study, the expression of the two *CbTPS7*-like genes i.e., *Cbur09G005210* and *Cbur09G005220*, was higher at CBS3 than at other developmental stages (Figure 6), they probably involve in the biosynthesis of linalool and nerolidol at CBS3 of *C. burmannii* leaves (1.2% and 0.45%, respectively). In *C. tenuipilum*, *CtGES* (closely related to *CbTPS7* of the TPS-g clade; Figure 8) encodes a geraniol-synthase (Yang et al., 2005). Nevertheless, the content of geraniol was hardly detected during leaf development of *C. burmannii*, indicating that the annotated *CbTPS7*-like genes *Cbur09G005210* and *Cbur09G005220* might be involved in the synthesis of other monoterpenes and sesquiterpenes too in *C. burmannii.*

Furthermore, *Cbur07G020680* and *Cbur07G020710*, with increasing expression from CBS1 and CBS4, were identified as genes encoding the IDI enzymes (Figure 6). The increased expression of these two genes explains the increasing content of d-borneol from CBS1 to CBS4. Moreover, *Cbur12G014500*, *Cbur12G004970*, *Cbur0019030*, and *Cbur10G002890* encoding GPPS were also identified, of which the first three showed an increase in expression from CBS1 to CBS4, while the last one demonstrated a decrease. A previous study showed that CcTIDS8 and CcTIPS10 of *C. camphora* form a heterodimeric functional GPPS, transforming DMAPP and IPP to GPP (Yang et al., 2021). In the present study, *Cbur12G014500*, annotated as a small unit of the GPPS enzyme, was identified as a sister to *CcTIDS8* in the IDS-c clade, while *Cbur12G004970* and *Cbur0G019030*, encoding a large unit of the GPPS enzyme, were closely related to *CcTIDS10* (Figure 9). These enzymes, most likely involved in the formation of GPP from DMAPP to GPP, resulted in a significant increase in the content of d-borneol in the *C. burmannii* leaves at the late developmental stages. In addition, *Cbur10G020080* and *Cbur12G006720* were annotated as FPPS in the IDSa clade. The expression of *Cbur12G006720* increased from CBS1 to CBS3, while that of *Cbur10G020080* decreased from CBS1 to CBS4 (Figure 6). Our phylogenetic analysis showed that *Cbur12G006720* and *Cbur10G020080* were closely related to *CcTIDS3* and *CcTIDS9*, respectively (Figure 9), both of which transform IPP to FPP (Yang et al., 2021). Thus, the increased expression of *Cbur12G006720* from CBS1 to CBS3 explains the increasing content of sesquiterpenes (caryophyllene and germacrene B) in the *C. burmannii* leaves.

The present study revealed dramatic differences in the gene expression profiles of the upstream molecular pathways: the DEGs in the MVA pathway exhibited a decrease in expression except for AACT (*Cbur04G014790* and *Cbur04G014840*) (Figure 6). In contrast, the expression of DEGs in the MEP pathway increased from CBS1 and CBS3 but decreased at CBS4, except for DXS (*Cbur02G033440*, *Cbur05G01050*, and *Cbur04G023450*). These observations indicate that the MEP pathway is more important than the MVA pathway for generating monoterpenes and sesquiterpenes in the *C. burmannii* leaves, consistent with other *Cinnamomum* species in the earlier reports (Yang et al., 2020; Li et al., 2022). Detailed analysis revealed the increased expression of the DEGs encoding the catalyzing enzymes DXS, 1-deoxy-d-xylulose-5-phosphate reductoisomerase (DXR), MEP cytidylyltransferase (MCT), 2C-methyl-d-erythritol 2,4-cyclodiphosphate synthase (MDS), hydroxymethylbutenyl diphosphate. Synthase (HDS), and 1-hydroxy-2-methyl-2-E-butenyl 4-diphosphate reductase (HDR), which likely influence the biosynthesis of monoterpene and sesquiterpene precursors. These DEGs encoding DXS, DXR, MCT, HDS, and HDR in the MEP pathway detected in the present study are consistent with the previous study, which compared with high and low content of d-borneol *C. burmannii* (Yang et al., 2020).

Typically, lncRNAs regulate the biosynthesis of secondary metabolites in plants *in cis* and *in trans*. Although the present study detected 922 lncRNAs (Figure 5B), only 12 were identified to regulate the biosynthesis monoterpene and sesquiterpene metabolism *in cis* rather than *in trans* (Figure 6). Chemotype-specific expression trends in *C. camphora* were found in merely trans-target genes rather than cis-target genes (Ni et al., 2021). The scenario, however, is different from the results of present study that lncRNAs that associated with monoterpene and sesquiterpene metabolism were all regulated *in cis*. Detailed analysis showed that the lncRNAs ONT.4890.2 and ONT.10874.2 regulate *Cbur02G029110* encoding HMGR *in cis* in the MVA pathway and *Cbur06G015920* encoding MCT *in cis* in the MEP pathway, respectively. In the previous study, lncRNAs were found to regulate HMGS and DXS enzymes in *C. camphora* leaves (Ni et al., 2021). These results corroborated that lncRNAs regulate the production of monoterpenes and sesquiterpenes by controlling the key enzymes of the MVA and MEP pathways. With regard to the down-stream genes, ONT.14891.1 was predicted to regulate *Cbur10G020080* encoding the FPPS enzyme, indicating that lncRNAs directly regulate the catalyzing steps of the sesquiterpene biosynthetic pathway. Moreover, we predicted that three lncRNAs, ONT.13408.1, ONT.10874.1, and ONT.10874.2, regulate two *CbTPS7*-like genes, *Cbur09G005210* and *Cbur09G005220*. Although *CbTPS7*-like genes might not generate the major monoterpene and sesquiterpene compounds, the increasing content of *CbTPS7*-like enzymes probably acts as an essential signal facilitating secondary metabolite synthesis in *C. burmannii* leaves during development. Thus, the lncRNA-mediated regulatory mechanisms in *C. burmannii* differ from those in *C. camphora*, where lncRNAs directly regulate the monoterpenes and sesquiterpenes targeting *TPS04-like* and *TPS21*-like genes (Ni et al., 2021).

## Conclusion

In the present study, we found origins of essential oil production through the observation of anatomy at different developmental stages of *Cinnamomum burmannii* leaves. We further generated 12 full-length transcriptomes of *Cinnamomum burmannii* leaves at four developmental stages using Nanopore sequencing technology. The differentially expression gene and WGCNA analysis revealed that a total of 44 DEGs were involved in monoterpenoid and sesquiterpenoid syntheses during leaf development. Functions of these genes were further predicted with regard to gene expression profile and phylogenetic relationship with those characterized in previous studies. Besides, a total of 922 lncRNAs were identified, of which 12 lncRNAs were predicted to regulate the genes associated with monoterpenoid and sesquiterpenoid syntheses *in cis*. The present study provided new insights the molecular mechanisms of monoterpenoid and sesquiterpenoid syntheses of *C. burmannii*.

## Data Availability

The original contributions presented in the study are publicly available. This sequencing data can be found in the NCBI Sequence Read Archive (SRA) under the BioProject accession number PRJNA892157.

## References

[B1] Al-Dhubiab B. E. (2012). Pharmaceutical applications and phytochemical profile of *Cinnamomum burmannii* . Pharmacogn. Rev. 6, 125–131. 10.4103/0973-7847.99946 23055638 PMC3459454

[B2] Amiri H. Lari Yazdi H. Esmaeili A. Samsamnia F. Eghbali D. Viskarami G. (2011). Essential oil composition and anatomical study of Scrophularia striata Boiss. Iran. J. Med. Arom. Plants Res. 27, 271–278.

[B3] Bordoloi K. S. Baruah P. M. Das M. Agarwala N. (2022). Unravelling lncRNA mediated gene expression as potential mechanism for regulating secondary metabolism in *Citrus limon* . Food Biosci. 46, 101448. 10.1016/j.fbio.2021.101448

[B4] Chaw S-M. Liu Y-C. Wu Y-W. Wang H-Y. Lin C-Y. I. Wu C-S. (2019). Stout camphor tree genome fills gaps in understanding of flowering plant genome evolution. Nat. Plants 5, 63–73. 10.1038/s41477-018-0337-0 30626928 PMC6784883

[B5] Chen C. Zheng Y. Zhong Y. Wu Y. Li Z. Xu L-A. (2018). Transcriptome analysis and identification of genes related to terpenoid biosynthesis in *Cinnamomum camphora* . BMC Genomics 19, 550. 10.1186/s12864-018-4941-1 30041601 PMC6057064

[B6] Chen F. Tholl D. Bohlmann J. Pichersky E. (2011). The family of terpene synthases in plants: A mid‐size family of genes for specialized metabolism that is highly diversified throughout the kingdom. Plant J. 66, 212–229. 10.1111/j.1365-313X.2011.04520.x 21443633

[B7] Chen L. Jianyu S. Li L. Li B. Li W. (2011). A new source of natural D-borneol and its characteristic. Jk. Med.Plants Res. 5, 3440–3447.

[B8] Cui J. Lu Z. Xu G. Wang Y. Jin B. (2020). Analysis and comprehensive comparison of PacBio and nanopore-based RNA sequencing of the *Arabidopsis* transcriptome. Plant Methods 16, 85. 10.1186/s13007-020-00629-x 32536962 PMC7291481

[B9] Darriba D. Taboada G. L. Doallo R. Posada D. (2012). jModelTest 2: more models, new heuristics and parallel computing. Nat. Methods 9, 772. 10.1038/nmeth.2109 PMC4594756 22847109

[B10] Degenhardt J. Köllner T. G. Gershenzon J. (2009). Monoterpene and sesquiterpene synthases and the origin of terpene skeletal diversity in plants. Phytochemistry 70, 1621–1637. 10.1016/j.phytochem.2009.07.030 19793600

[B11] Ding J. Yu X. Ding Z. Cheng B. Yi Y. Yu W. (1994). Essential oils of some Lauraceae species from the southwestern parts of China. J. Essent. Oil Res. 6, 577–585. 10.1080/10412905.1994.9699349

[B12] Edgar R. C. (2004). Muscle: Multiple sequence alignment with high accuracy and high throughput. Nucleic Acids Res. 32, 1792–1797. 10.1093/nar/gkh340 15034147 PMC390337

[B13] Feng Y. Shen T. Yang Z. Tan J. Xu K. Chen X. (2022). Identification of genes involved in oleoresin biosynthesis in *Pinus massoniana* through the combination of SMRT and Illumina sequencing. Ind. Crops Prod. 188, 115553. 10.1016/j.indcrop.2022.115553

[B14] Gershenzon J. Maffei M. Croteau R. (1989). Biochemical and histochemical localization of monoterpene biosynthesis in the glandular trichomes of spearmint (*Mentha spicata*). Plant Physiol. 89, 1351–1357. 10.1104/pp.89.4.1351 16666709 PMC1056021

[B15] Han X. He B. Xin Y. Xu M. Xu L-A. (2021). Full-length sequencing of *Ginkgo biloba* L. reveals the synthesis of terpenoids during seed development. Ind. Crops Prod. 170, 113714. 10.1016/j.indcrop.2021.113714

[B16] Hou C. Lian H. Cai Y. Wang Y. Liang D. He B. (2021a). Comparative Analyses of full-Length transcriptomes reveal *Gnetum luofuense* stem developmental dynamics. Front. Genet. 12, 615284. 10.3389/fgene.2021.615284 33841494 PMC8027257

[B17] Hou C. Tian Y. Wang Y. Lian H. Liang D. Shi S. (2021b). Revealing the developmental dynamics in male strobilus transcriptome of *Gnetum luofuense* using nanopore sequencing technology. Sci. Rep. 11, 10516. 10.1038/s41598-021-90082-0 34006996 PMC8131605

[B18] Hou J. Zhang J. Zhang B. Jin X. Zhang H. Jin Z. (2020). Transcriptional analysis of metabolic pathways and regulatory mechanisms of essential oil biosynthesis in the leaves of *Cinnamomum camphora* (L.) Presl. Front. Genet. 11, 598714. 10.3389/fgene.2020.598714 33281883 PMC7689033

[B19] Jain M. Olsen H. E. Paten B. Akeson M. (2016). Erratum to: The Oxford nanopore MinION: Delivery of nanopore sequencing to the genomics community. Genome Biol. 17, 256. 10.1186/s13059-016-1122-x 27887629 PMC5124260

[B20] Ji X. Pu Q. Garraffo H. Pannell L. (1991). Essential oils of the leaf, bark and branch of *Cinnamomum burmannii* Blume. J. Essent. Oil Res. 3, 373–375. 10.1080/10412905.1991.9697964

[B21] Kong L. Zhang Y. Ye Z. Q. Liu X. Q. Zhao S. Q. Wei L. (2007). CPC: Assess the protein-coding potential of transcripts using sequence features and support vector machine. Nucleic Acids Res. 35, 345–349. 10.1093/nar/gkm391 PMC1933232 17631615

[B22] Kung J. T. Colognori D. Lee J. T. (2013). Long noncoding RNAs: Past, present, and future. Genetics 193, 651–669. 10.1534/genetics.112.146704 23463798 PMC3583990

[B23] Lalitha S. (2000). Primer premier 5. Biotech Softw. Internet Rep. 1, 270–272. 10.1089/152791600459894

[B24] Langfelder P. Horvath S. (2008). Wgcna: an R package for weighted correlation network analysis. BMC Bioinforma. 9, 559. 10.1186/1471-2105-9-559 PMC2631488 19114008

[B25] Langfelder P. Zhang B. Horvath S. (2008). Defining clusters from a hierarchical cluster tree: The dynamic tree cut package for R. Bioinformatics 24, 719–720. 10.1093/bioinformatics/btm563 18024473

[B26] Li F. Huang S. Mei Y. Wu B. Hou Z. Zhan P. (2022). Genome assembly provided new insights into the *Cinnamomum burmannii* evolution and D-borneol biosynthesis differences between chemotypes. Ind. Crops Prod. 186, 115181. 10.1016/j.indcrop.2022.115181

[B27] Li H. (2018). Minimap2: Pairwise alignment for nucleotide sequences. Bioinformatics 34, 3094–3100. 10.1093/bioinformatics/bty191 29750242 PMC6137996

[B28] Li J. W. Ma W. Zeng P. Wang J. Y. Geng B. Yang J. C. (2015). LncTar: A tool for predicting the RNA targets of long noncoding RNAs. Brief. Bioinform. 16, 806–812. 10.1093/bib/bbu048 25524864

[B29] Lin Y-L. Lee Y-R. Huang W-K. Chang S-T. Chu F-H. (2014). Characterization of S-(+)-linalool synthase from several provenances of Cinnamomum osmophloeum. Tree Genet. Genomes 10, 75–86. 10.1007/s11295-013-0665-1

[B30] Liu J. Wang H. Chua N. H. (2015). Long noncoding RNA transcriptome of plants. Plant Biotechnol. J. 13, 319–328. 10.1111/pbi.12336 25615265

[B31] Liu X. Tang N. Xu F. Chen Z. Zhang X. Ye J. (2022). SMRT and Illumina RNA sequencing reveal the complexity of terpenoid biosynthesis in *Zanthoxylum armatum* . Tree Physiol. 42, 664–683. 10.1093/treephys/tpab114 34448876

[B32] Livak K. J. Schmittgen T. D. (2001). Analysis of relative gene expression data using real-time quantitative PCR and the 2(-Delta Delta C(T)) Method. Methods 25, 402–408. 10.1006/meth.2001.1262 11846609

[B33] Love M. I. Huber W. Anders S. (2014). Moderated estimation of fold change and dispersion for RNA-seq data with DESeq2. Genome Biol. 15, 550. 10.1186/s13059-014-0550-8 25516281 PMC4302049

[B34] Ma Q. Ma R. Su P. Jin B. Guo J. Tang J. (2022). Elucidation of the essential oil biosynthetic pathways in *Cinnamomum burmannii* through identification of six terpene synthases. Plant Sci. 317, 111203. 10.1016/j.plantsci.2022.111203 35193750

[B35] Ma R. Su P. Guo J. Jin B. Ma Q. Zhang H. (2021). Bornyl diphosphate synthase from *Cinnamomum burmanni* and its application for (+)-borneol biosynthesis in yeast. Front. Bioeng. Biotechnol. 9, 631863. 10.3389/fbioe.2021.631863 33644023 PMC7905068

[B36] McCaskill D. Gershenzon J. Croteau R. (1992). Morphology and monoterpene biosynthetic capabilities of secretory cell clusters isolated from glandular trichomes of peppermint (*Mentha piperita* L.). Planta 187, 445–454. 10.1007/BF00199962 24178138

[B37] Ni Z. Han X. Chen C. Zhong Y. Xu M. Xu L. A. (2021). Integrating GC-MS and ssRNA-Seq analysis to identify long non-coding RNAs related to terpenoid biosynthesis in *Cinnamomum camphora* . Ind. Crops Prod. 171, 113875. 10.1016/j.indcrop.2021.113875

[B38] Qiu F. Wang X. Zheng Y. Wang H. Liu X. Su X. (2019). Full-length transcriptome sequencing and different chemotype expression profile analysis of genes related to monoterpenoid biosynthesis in *Cinnamomum porrectum* . Int. J. Mol. Sci. 20, 6230. 10.3390/ijms20246230 31835605 PMC6941020

[B39] Serebrynaya F. K. Nasuhova N. M. Konovalov D. A. (2017). Morphological and anatomical study of the leaves of *Laurus nobilis* L.(Lauraceae), growing in the introduction of the northern caucasus region (Russia). Phcog. J. 9, 519–522. 10.5530/pj.2017.4.83

[B40] Shan B. Cai Y-Z. Brooks J. D. Corke H. (2007). Antibacterial properties and major bioactive components of cinnamon stick *(Cinnamomum burmannii*): Activity against foodborne pathogenic bacteri. J. Agric. Food Chem. 55, 5484–5490. 10.1021/jf070424d 17567030

[B41] Shan T. Wu C. Shahid H. Zhang C. Wang J. Ding P. (2020). Powdery-fruit disease of *Cinnamomum burmannii* and its influence on fruit essential oil. Inter. J. Agric. Biol. 24, 1077–1083.

[B42] Shen T. Zheng Y. Liu Q. Chen C. Huang L. Deng S. (2022). Integrated SMRT and illumina sequencing provide new insights into crocin biosynthesis of Gardenia jasminoides. Int. J. Mol. Sci. 23, 6321. 10.3390/ijms23116321 35683000 PMC9181021

[B43] Stamatakis A. (2014). RAxML version 8: A tool for phylogenetic analysis and post-analysis of large phylogenies. Bioinformatics 30, 1312–1313. 10.1093/bioinformatics/btu033 24451623 PMC3998144

[B44] Sun L. Luo H. T. Bu D. C. Zhao G. G. Yu K. T. Zhang C. H. (2013). Utilizing sequence intrinsic composition to classify protein-coding and long non-coding transcripts. Nucleic Acids Res. 41, e166. 10.1093/nar/gkt646 23892401 PMC3783192

[B45] Tholl D. (2015). Biosynthesis and biological functions of terpenoids in plants. Adv. Biochem. Eng. Biotechnol. 148, 63–106. 10.1007/10_2014_295 25583224

[B46] Tian Z. Luo Q. Zuo Z. (2021). Seasonal emission of monoterpenes from four chemotypes of *Cinnamomum camphora* . Ind. Crops Prod. 163, 113327. 10.1016/j.indcrop.2021.113327

[B47] Vranová E. Coman D. Gruissem W. (2013). Network analysis of the MVA and MEP pathways for isoprenoid synthesis. Annu. Rev. Plant Biol. 64, 665–700. 10.1146/annurev-arplant-050312-120116 23451776

[B48] Wang L. Park H. J. Dasari S. Wang S. Q. Kocher J. P. Li W. (2013). Cpat: Coding-Potential Assessment Tool using an alignment-free logistic regression model. Nucleic Acids Res. 41, e74. 10.1093/nar/gkt006 23335781 PMC3616698

[B49] Wu G. D. Lian H. M. Z C. H. (2020). Content variation and evaluation of essential oil and its main chemical components of *Cinnamomum burmannii* in Guangdong province. For. Environ.Sci. 36, 88–95.

[B50] Yang L. Froberg J. E. Lee J. T. (2014). Long noncoding RNAs: Fresh perspectives into the RNA world. Trends biochem. Sci. 39, 35–43. 10.1016/j.tibs.2013.10.002 24290031 PMC3904784

[B51] Yang T. Li J. Wang H. Zeng Y. (2005). A geraniol-synthase gene from *Cinnamomum tenuipilum* . Phytochemistry 66, 285–293. 10.1016/j.phytochem.2004.12.004 15680985

[B52] Yang Z. An W. Liu S. Huang Y. Xie C. Huang S. (2020). Mining of candidate genes involved in the biosynthesis of dextrorotatory borneol in *Cinnamomum burmannii* by transcriptomic analysis on three chemotypes. PeerJ 8, e9311. 10.7717/peerj.9311 32566406 PMC7293187

[B53] Yang Z. Xie C. Zhan T. Li L. Liu S. Huang Y. (2021). Genome-wide identification and functional characterization of the trans-isopentenyl diphosphate synthases gene family in *Cinnamomum camphora* . Front. Plant Sci. 12, 708697. 10.3389/fpls.2021.708697 34589098 PMC8475955

[B54] Yuting L. Huan H. Jiabao Y. Feng X. Zhang W. Yongling L. (2022). Regulation mechanism of long non-coding RNA in plant secondary metabolite biosynthesis. Not. Bot. Horti Agrobot. Cluj. Napoca. 50, 12604. 10.15835/nbha50212604

[B55] Zhao X. Yan Y. Zhou W-H. Feng R-Z. Shuai Y-K. Yang L. (2022). Transcriptome and metabolome reveal the accumulation of secondary metabolites in different varieties of *Cinnamomum longepaniculatum* . BMC Plant Biol. 22, 243. 10.1186/s12870-022-03637-2 35585490 PMC9116011

[B56] Zuo Z. Wang B. Ying B. Zhou L. Zhang R. (2017). Monoterpene emissions contribute to thermotolerance in *Cinnamomum camphora* . Trees 31, 1759–1771. 10.1007/s00468-017-1582-y

