# Supplementary material for: Full-length transcriptome sequencing reveals the molecular mechanism of monoterpene and sesquiterpene biosynthesis in Cinnamomum burmannii
Source: Front Genet. 2023 Jan 6;13:1087495. doi: 10.3389/fgene.2022.1087495 (PMC9852720; doi:10.3389/fgene.2022.1087495)
Supplement: Supplementary file 1 [file Table8.DOCX]

**Table S8** Statistics of DEGs

| **Comparisons** | **Gene Number** | **Up-regulated genes** | **Down-regulated genes** |
| --- | --- | --- | --- |
| CBS1_vs_CBS2 | 2259 | 1322 | 937 |
| CBS1_vs_CBS3 | 5505 | 2739 | 2766 |
| CBS1_vs_CBS4 | 6051 | 2749 | 3302 |
| CBS2_vs_CBS3 | 881 | 326 | 555 |
| CBS2_vs_CBS4 | 2687 | 1075 | 1612 |
| CBS3_vs_CBS4 | 862 | 279 | 583 |
| CBS1_vs_CBS2 | 2259 | 1322 | 937 |
