# Supplementary material for: Full-length transcriptome sequencing reveals the molecular mechanism of monoterpene and sesquiterpene biosynthesis in Cinnamomum burmannii
Source: Front Genet. 2023 Jan 6;13:1087495. doi: 10.3389/fgene.2022.1087495 (PMC9852720; doi:10.3389/fgene.2022.1087495)
Supplement: Supplementary file 2 [file Table7.DOCX]

**Table S7** Statistics of non-redundant consensus reads

| **Sample name** | **Number of non-redundant reads** | **Number of base pairs (bp)** | **N50** | **Mean length**  **(bp)** | **Maximum length (bp)** |
| --- | --- | --- | --- | --- | --- |
| CBS11 | 20,103 | 27,265,498 | 1577 | 1356 | 5700 |
| CBS12 | 20,320 | 29,738,105 | 1700 | 1463 | 7362 |
| CBS13 | 19,654 | 25,229,033 | 1496 | 1283 | 4962 |
| CBS21 | 17,857 | 23,302,286 | 1520 | 1304 | 5494 |
| CBS22 | 18,828 | 24,981,451 | 1542 | 1326 | 5678 |
| CBS23 | 17,883 | 24,733,899 | 1619 | 1383 | 6926 |
| CBS31 | 17,383 | 24,376,415 | 1629 | 1402 | 6828 |
| CBS32 | 16,856 | 22,607,586 | 1565 | 1341 | 5708 |
| CBS33 | 17,496 | 24,279,437 | 1617 | 1387 | 5742 |
| CBS41 | 11,615 | 14,310,706 | 1415 | 1232 | 5173 |
| CBS42 | 12,136 | 15,669,821 | 1491 | 1291 | 5431 |
| CBS43 | 14,065 | 18,424,308 | 1518 | 1309 | 5609 |
