# Supplementary material for: Full-length transcriptome sequencing reveals the molecular mechanism of monoterpene and sesquiterpene biosynthesis in Cinnamomum burmannii
Source: Front Genet. 2023 Jan 6;13:1087495. doi: 10.3389/fgene.2022.1087495 (PMC9852720; doi:10.3389/fgene.2022.1087495)
Supplement: Supplementary file 3 [file Table9.DOCX]

Table S9 Sequences of all lncRNAs detected in the present study

>ONT.1165.1 LG01 25564347-25566565

ATCTTTAACTCTTTCATTCATTTCATTCACTCCACTCACCAACCTCAACAGTAGCCGACTCATCTTCTTCTCTTCCTTTCTTTCATCTTCTTCTTCTCCGTCGACGGGTATCGGAACTCCGGCGAAGAATCCAAGCCACTCATCATCTTCCTCAAGTCTCCAAGAACACAACTATACACTTTTCTAAGTGTTTCACCTACAACCCATTTAAAGGAGCTAATTGGGTTTGGAGAGTTGGTTCACCCCTTTGGAGCTTCTTAGAGTTGTTTGTGTCCAAAGTCTTCTTGAGTTTGAGTCTAGCACATCACCAAGGCAAGTACCGAGCCCTACTTTGAGGAGGAAGAAATGACGGAAGAAGTTCAGGCGGATCAGCCTGATGACTTCCTTGAGTACAGTACGGACGACATAGTATAGCCGTACTGACCCCGTACGCGAGAGTCTCCACCTTTTGGACTAGTTTCATATTTCTGTTCTTTTATCTTTTGATTAGGCCCGAGTTTGGTGTGGCATGATGTTGTAATTAATATAGTCTTAAACTTGAACCTTGTGTATGATATTCAGTATTGTTCTTAAACTATACATCTATGAGATACCGCTTGTGTTTGAAGCTTCCGCT

>ONT.17936.8 LG12 50040420-50041290

AGATAAATAAGGAAATAGTTAAGACCCAGCGCGTGATTGCACCTGTGGGCCAGATGCAGCTGAAGAGCCTCATAGAAGCTCATGTGGTAAGATTAGATGGGAGCACCAAAGGTTCTACCATTTTAAGAGAGTGGGATTCCTATCTTCCTCGGTTCTGGCAGTTGGTGCCCCCAAGTGAGGAAGACACCCCAGAGGCCTGTGCAGAGTTTGATAGTAGAGCAGCTGAACAGGTGACATTGCAGTCTGCACGACAATAACGCTGCCATGGCATGGAGGAGGAAATATAAGTGCTCATTGTGAATAGAAAACAAGATGCCAAGTACAGATGAACTCAAACCTCTGTAGATACCAGCTGTGTAAGATGATAGCCGTTTCTTCTCTGAAGAAGAGAGAGTCGCATCAAATGGATTGGCAACTGCCCAAAATCTTGTTGTACTTCAATGTCAAATCTCATCAAGGGATGTGCCATGAAAGGGATAGGCGATGCATTCTTTTCCCACTTAACCAAGAAAAAGTATTGCTTGCTCCCAAAAATTTTGGGGATGTTTTACGCATCATATTGGATGTATTCATCTTTTATGCATATGTAATTTGAGCTTTGTATGGTGATCCCTTCCAGTAAAAATAAGTCACGAGGTTTGCAGTCATGGACAACACATTGCCCAAATATGTATCCCAAACCCAAATGGATCTATTTTTGTTTCAATGGATTT

>ONT.2913.1 LG02 4232250-4234980

AGCCAATTAAGAACACATGCACCATATCTTTCTTGAAACTCAGGAGTGCTCTGTAAGAACTCCAATCCTGGATGAGTGCCCAAAAGTTCTCGAAGGACAGGTTTGAAGTCTTCCTGAATCCACCTCCCCCTCCCACCGCAGCCGGATCTCTCTCTCTCCCTCCCTCCCTCTCCCTCTGCACCATGCGCTCCGGCGCCCATGCCACTGCCGGATTGAGCCTCCCCCCCACCCCCCCATCCCCTCTCTCTGTGCACCGCGCGCTCCGGCGCCCACGCCACAGCCGAATCCAGATTCGTGCATTCCATCACCCTCGTTCTCGCCGGAAATTGAATCAGGGCCCGAATCGTCCTTCATTGGTGTGTTCTAACTCTAGATTGAGTTAAGAGGCAGTGTGCCTTCAATCAATTTCTAAAGGTTTGAGAGCATCAGATCTACCACGGCATCCTGCACGGACATTTTTTTTTTTTTTGTTTTCTATAATTGTAATGGATATAATTTGTTGAATGTTTTGTAATGTTTTGAATGTAATTAATATAAGACTTTCTTTTGTT

>ONT.17509.1 LG12 2570273-2586238

TTTGTTGACTGTTAATGGCTTCTTCTTCCTCTTCGGTGGTGTACTGTAAAGAAGGGGGTTTTCTATCTTATGTGCTTCGTTGACGATCTCGAAACGCATCTTCGCTTGCATTCTTGAAAGAAAGATGCAGTCCCCTTGATAGTCATTTCTAATGGTAATCTCAATATTGACCAGAGCTAATCCACCTTTAACCAAGTTGCGGCAAGCTGTCTCTCAAGGTTCGATGCTGTTGTCTTCTCCATTTGTTCTTTCCCAATTCAAAATCTGTGTTTGATCCACTTGACCAGGCATGTGCACCTATGATCACCCTAAATATGAACATGACACTCAAAAATATAAATTAATAAATTAATTAATGATTGGAATTCTTAATGAAACTCTGATTCCATTTTCTGTGGCCTCACCAAACAATATTAATCTGTTGTATTGATGATCATTTTTTAAATGTTAAAAACTGGAGTTTTC

>ONT.5007.1 LG02 116612740-116622426

ATCTTCGCGTTTTCCCCATTTCCATGTTTGTCTCAGATTGAGCTTCTCGTCCCTTATCTTTGCTGATTTTTTTTGGTTTTCTTTCGTGTTTTTGGCTGAATGGTACTCCCGCTCAGTGATTCCATGAAGGTTTTCATCTTTTTGTCGTTTTTAAGCACTAACGCTTGTCATCTGTTGCAGCAGATCGTTCATGTGGAAGCTTTTTTCCTCAGATCCATGGAGATTTCTGCATTCCATCATCAGATCTTCTTTATGGCTTCAAAGGAAAGTAGTTTGGTGTGAAAATTGAAAAAACTTGTGAAACTGACATGGTTTATGATCTTGATTGGGCTGATAGCAATGTAGGTATGAATTAACACCAAAAGAGGGCTTGTGTCAGTGGATCTATGTGATCTCTGACCTTTGCCATGTCATGTGCGCTTGCATGGCAGGTCAAAAACCCTAATGAAATTCTCCGTTTTCTGTTATTAGGGAAGATAGATCTCATGGGGGCTGTAGCCTGGGTTCTCCATTCTCTTGTGTCCTCTGTTGACTCCCTTGTCTTTTGTCTCCTGTCTTTATGTATATTCCTTGGAAAGTTAATGTTCCAGGGGAAGTGAGGAATCCTCAAATCCACAGATTCCTCACGTATTTCCAAATGGGACCTTGGTTTTTCCTCTGTGGTGCTTGTTGCTGTAACAAGTGCTGGAAAGACAATGTAAAAGTGCGGATGTTGCATATTCTTTCCATACAATGAGTGGGATTTTTGATTCCGAGGGGGCTGGTTGGAACTGGGTATCCACATGTGAGGGAAATTGCTAATGTTACATTAAAGGTGTCTTATACATGGCAACCTTTTTCATTTTTGTGATGAATATGGTCTGCACACCCATTCCCGAAGATTTGTTTGTTTGGTTGAGGAATTATATGAATTCCTGGAAAGGAATCCTGTTTGGTGAGAAATTGATGTTCTAGAAATGAGGGTTTTTTTCCCCATCTTGTCCAGTTGGATGAAATTCCATTTTTTACATATTTTTTTTGGTTTTATTTTTTCCATTTCATCATATGCTTTTGTACTTAATTTTGCCTCATTTGTGAAAAGAAGGGTTGACTACAATATGGTTTTTTGATATTCGCAATTTCTTGGCTGCTTGTTAATTCCATTCCTTCTAGTTCTCAACCAAACAGATCGTGAAGGTAATCATTAAACTTGCTTTTTTAGTGATCTATAGACCCTTTCACATTATTGTAGTTCCTTTTTTTTTTCGTCGGAATCCTTTCACAGAATACTTGTACAACCCCAAGAGGGATCAAGACTCCTGCCAAGAGTGATATGAAGATGGTAGCATGAGTGACCTGTTACAAAAAGTTTGGAGCTTTTGCTTAGACTTGATCGATGTCATTCAATATTGAAGAGTAATAACGGTGCTCCAATTGAGAGTGAGTACAAGATTTTACCTATTAATGAAATATGGCAAATTGAAGACAATGACAGTTATCTTCCATGGGGGCTTGAGTTTTATTGACTTCAATCAAATGTGGATCCATTAGGTTAGTCTTGATCAGGTTTTGGATTTGGGGTATTTTGGTAATTGTATTGGGCTTTTTAAGGCCATAAAGGATTCTCTCTCTCTCTATATATATATATGGTGTAAATTGTAAATCCTAGATACACGGTCATATATTATCTAAATGTTCTGTGTGATTTCTCA

>ONT.4373.1 LG02 20093799-20096126

GAGATGATTCATCTCAACGATGAGTCATTCCAACAAGAACAGCAAGAGAGACACAGAGAGGTGGAGAACTGCTCATCTTCGTTTCTGTTTTTGTGAGAGCTACTCATCTTTGTTTTTGCTTTTGAGATTATCAAGTGATTTTCAAAAGAGAAAAGGGAATTCATCTTCTTCATCATTCCATCAAGTGGGTTGCTGGTTTTCTTAGAAATTGGTGAGTTTGACTCAAAGAAAGGTTGAAACAAGGGGCTTTTGTGTTCAAGCACAGAAAAAAGGCAACTGCCATTGCCGGTCCTTCTAAGACCACAGCGCCACCGGAAACGTCCGGCACGGTTCCTCTAGGAGGACTAGTAGAATGGAAATGATAGAAAAAGACTGAGAATCGAACTTTTATACTTTTGATCCTTGCATTGTATTTAAATATGCAAGTGATGAATTTTGTATATAGTGTAAAGAACTTTTGATGTGATACCAGCAATGTATATTTGTGAATATTTCCGCATAATGTATAGTGTTATCCTTTGTGAA

>ONT.11892.1 LG07 24982046-24985196

ATCCTTTTCCCTGCAACGGTTCCAGCAAGAGCTGGAATCACTCCTCTCTCAGCCACTCTCTCTCTCCTCTCTCCCTTGGCTTCTCTTGATTGTAAATGGAGATTTGATCTCCTCATCATCTTCCATACCTTCCAAGCTTTCCAAAGATGTTATTTTCATGGATTTTGAGCAAAGGCACCTTCTCTTTTCAAGCTCTTCTTCTTGGTTCAAGGCTTTAAAGGGTTTTCAAGGGAAAATTTGGGGGTTTTGGTGGGTTTGGAGCTAGAGACTTCTTTGAGGCTTTTGTGGAGTGATTTGTGCATCTTCTAACCCAAGGCAAGTTCAGATTTCTTCGTCCCGAGATGAGTGTTTGGTTGGCTATGGAGCCATGTCTTGCAGGTTGTTTTGTCGGCTATGTCATATAGACATTGGGCTCGACATCGGAGCTACGTCACATATGGGTAGTAGTTAATAGCTTATTTTGTATGGCTTTTATTAGCCTTTTACTGGGGATTCTTAATTAATGTATATTGATAATTGTAACTTGAACACATTTGGGAATGTAATATCCTGTTATTGTCG

>ONT.15878.1 LG10 46952413-46952934

ATTCTTGGCCTCCATCACCACGCAAGAAAAAATAGCAGAGGCAAATGAGTTGTAAACCCCACAAAAGTTTCAAGCTTATGTCAGTGCAAGGATCTTCTGTTACCTTCAAGATAAAGCTATGGAGTCAGGGATTCCTTTCAAGCCAGAAACCAAATGATGGAGAAAAGACTATTGCCATGGGGAACGGAAGGAATCACGAGTCGAAGCAGTAGGCATTTATAGATTGGTGCTAGACACCAGATTTGTTTTGGACCTCCATAATGTTTTCCTTATTCCTGAGATTTCTAGAAATTTAATTTCTGTTTCAAAGTTGGTTCATGAAGGGTTTGAATTTATTTTTGGACAAAACTATATGAGAATTTCTAGAAATGAATGTTTAATTGGGAATGGTTATTT

>ONT.1093.1 LG01 20572509-20578722

AGAAACTGAACAAACCTGCGTTTGAGAATCCTTTGAAGCTAAATTGAAGAAATGGAGATGGCATTGCTAAACCCATCACCTTCCTACTGCTTTTCCAACAGCTCGTCTACCCGTATCGGTCCCTCGGAGACCGTCCAGGCATCGGAGGAGGCCCATGCGCTATCTCCAGGAGGTCTAGCGGAGTGGACTTGATTTTGTTAGAAGAAACATTTAGTTCTTTGGAAACTTTTTGAGAATTATTTTGTATAAACATTTTGATATTGTAAGCAATGTAAATATTATTCCCTGCATTGTACTAAATATGCAGTGGTTGATTTGGATGTATGAAACGATGTACATGGACTTTATGATTCATGGTGTTTAAATAGGTTGTCAATGT

>ONT.5138.1 LG02 124639762-124640428

GGGGCTAATTGGATCTTGATAGGGGTGCATTTTAAAGTCTCAAAAATCAAAGATGAAAACAGGCATTTCAATCCAACAGCTAGTGCTGAAGCAAGAATGCGCAAGTTGCACATGTGGGAGCAAACCTTTAGTGTGATCTTATGATGATGACGACGACGACGAAGACCATTATGATGAAGAAAAAATGGAGTGGTTTGGTTGAGAATTCTGGTGGCAGCTGGTGTTGTGGAAACTGAAACAGCTCCCCAAGGAACCACCACTTTGTGTACTGTGTTGTGTACATATAAGAATCCCTTTAGATATGTCTTTGATTGCTGGAGGGTCATGAGGAAGTTTCTGCTCAAAAGAGAGCTTGTAATTAAAACAGATTTTGGATACTATGTTCTCTATATCAGTTAGTTTGTGCTTTTAACTTCTCAGGCATAATACATGAATTGCCTTTGATGATTTAAATTATAATAAGGTCTTGACTCTTGGCA

>ONT.9725.11 LG05 90482630-90484605

GTGCTACTTCATCTCTGAAATCATCTGTGCACTTTCCTCAAGTTCTGATTTCAAAATCTTTGCTAAAAACCAGTGACCTAAAGAGACTATGAACGCATCTGAAGACACTTCTCTATTTTCACTCTGGTAAGCACAACCACTACTCCATTTTTGACATCCTCTATGCCTCATTCAATGCCCTTTAAGTGTTTCCCACGTGAAGTCAATTTTGTACCCTCAATGAAGTTTCCTCAAAAGTTTGTTCACTTTTGTTTTAGATGATACTCCATTTCTGAAATTATCTGTGTATTTGCTAAAAATCAAGTAAGTTGTCTATGTTATTTTAGTTCAGGCTATTTGATTTCTCCCTTTGATTGATTGTTAAGATGGATAATCAGTAATGTAATTTAGGGGGTGTAGTAATGTAATTTAGGGGGCGTCTCCAGTGCAGCGACGCTGCATCGTAATACCGTGCATCTTCCGGCTCCTCGTATGAGCGGATGCGTTCAACTTTTGTTGTTGCAATGAAAAACGGATTTCAAGA

>ONT.8871.5 LG05 107669627-107676873

AAACCCTAAACCCTAAGCCCTCTTACTCTCTTCGTTATCGCCTCCTCTCGTTCTGGTCTGCGCCTCTGCCTCTCTGCGAACTGCTCTCTTCTCCCACTCAGCTCTCCGTGATCTTCATCTCTCGCTTAGCTCTCTGCTCTCTACTCTCTGCTCTGCTCCCGCCGTCGCTGCGAGCCCTCGCACAGTCGCACGCAGCACGTTGACGCCGCCACTCTCGCACCTCGCACCGACGCACGCACGCCGCACGCCGCACGGTGACGCTGCCTTTCTGCACTCTCTCTCTCTCCCTACCTCGCCTCACCCAGTCTCCCTCTCTCACCCTCTTTCTGTTAATTAGCTGGGCCCAAGGTGATTCAATGAAGCAGGTTGATGAAGTTGGATCTGAGATGTTTGATTAAGTTCAAAAGGTACTCAAGTTGCAAAAAGGTTGTTTTTTTTTTTATCACTACAGACTAATGTATTTTTTTTATTTTATTCTAACTTCACTAGTGTATTTCTAAACTCTTGAATGTAAATAAATAGAAAAAAAATTCAGTAAAGGTTAGTTATCTTTATTGGATGATTATTATTTATTTTC

>ONT.8851.11 LG05 106341698-106343079

GGAATTGCCGCGGAAATCAAGGGTGGGGTCCTGACTAGGGAAGGTGTAATGGACGGCCTCGATCTGCTGCTGAGGAGGGAAGAAGGGAAAAAGATGAGAGAGCAGGTGGGATCACTGAAGCTAATTGGCAAGAATGCAGTAAGCCAAACAGGAAGTACAACCAAGAATCTAAAGGCTCTTTTGGAGATTATAAGTGGATCTGCTTAACAGAGTGTGATACTGGGGACTTTATTTTATGATGCTAACCCAATACTTTATTTTTATTCTATCATGCTATTGGAGTTTAATTTGATTTGTATTATTGTTGGAGTTTGATAATGCTCATCTACACTTACATTGAACTATTATGGAGTGTTGGAACTGGGCTCCTTTTGCTTTTATGGATTCTTTCTTATTTTAGTTAATTTTATTGTTGCAATCC

>ONT.14191.2 LG09 64509285-64509966

AAGCAGACATTGAAATGTTGTTACTTGTAATTTGATGATGATGGACGGCCCAGAAGAGGATAAGAGTTTATGTTGTGAGGGAAATGAAAGTGTGGTAGGAGGAGGTGGGAAAGGGGCAAAGATGGAATAAAAAGGCAGGGAAAAGGTAGAGCATGGGAGAGGTCACCAACAGAATAGGCCTGTTGTTGCCCGTCCCCTGCACTGCCTCTTCCCTGGCTCTCTCTCTCTCGCTCTCTCTCTCTTAACTTTCTCTTTTTCTCTGCTCTTATTTCCCTCCCTGGTTCTCTCACTGAGGGAGATGTCTTACCATTGAGCTACTAGTGGATTGGCACTGCATTTCTATAATTCTGCACCATTACTTTTTTTTTTTCTAATGTTTTTTTGAAGTTTGTGAATTATAATATTAGAATTTTCAAATAAATGTTGTCTATCATGTAGTTCAAAGGAGGCAGATGCTTCTAAAGGGATGAATCATTGGATTGATCATAGTTGGATTATGTAATGTGATGGGTAAAAACGGTGTTTATAGTCCTAATGTCTGTAATTCTGATTCTATCAATACTTGGGTGGTTACAATGCCA

>ONT.11411.1 LG07 67336478-67343665

CCCCCCCAAAAGAAAAAAAATCTATCATCTAGGGTTTTCTCTCGAGCTCTTCGTCTTTCGTTTCAGGGAAGGAGGAAATGTTACCCATTCCTGAAGGTATTCAATCAAATGATGCTTTATTACCAAAGGATATATAGGCAATTCCATTCAGACCGCTCTCTCAAAGACGACTGACATTTCTTCAGCTTGGGCATAGGGATAGTGGCAGCTTCTTGAGTTAGAGCAACTAAAGCCCCGGACATATCAACATCTTGGAGTTCACCCTTTTCTTGCATGTACTCAAGAGGTAGTAAAATTAGATCAAAGACGTGCAACTTATCCAAGGATTTGATTCAAGATAGATCGGAATGGAGAAACAAAATTCATGTAGCCGACCCCAACATAGGTGGGACAAGTCTTTGATGATGGTGATGATGAAATAACTCTGAGGAAGTAGATCGTAGCGGCGTCATTATAGCGTGTAAAAGATTATGGGCATAATTTTGGAACTGTTATTATTGTTTTTATATTTTTAATTTGAATGGAATTTTGAAGTGAAGATTTTAGTTTTCCTCCTAGGGATTACCTTGTTTATTTCGATCACAGACATTTGTGGATGTTAAAGATTTTGGAAAATGATCAAATAATAATATTTGTGTATTTTAGAGATT

>ONT.12454.6 LG08 47133241-47135103

GCCCTATTTCCCTTCACAGAAACTTACCGAGACCTGGCTGTTGAAGAAAGTTGAGAAGCCAAACATCATGTTGTTGTTGAAGCTCAAATCCCTTACCATTTTCGTCGCTGAAGGTCCTTTGCTCAGTTGCTTGAGGCACGCTCCTCCTGCCGCTGCTTGCTACTCCTTCCCCTTCTTCTCGCTTCTTTTTCCTCTTCTCCTCCTCCTTTCCATCCTCTCTCCTTCTTGACCCTTTTTCCGCATGAAGGAAATGAGGCAGCAGCAGATTTTCTTTCCCGAAAGGCATCCCCTCCCTATGGCGTTCTGAAATTCAATGTGGATGGTGCCGCTCATGGTAAACTGGACTGGCTGGGGCCGGCAGGGATCTTAGATTTCTTAGTACAACAATGAGTGGATGTCTTCTTTTTGGTGGCCTTTTATTAATGCTTTTGAGCAACAAAGGGTTTGGTTTGCGGGGCTTCTGGTGGGCACTTGCAGGATTTCAATGGGCTCGGTTTTCTCTAGCTTTACAGAGGGTTACTTCTCCTCAGGGTATGTTGTACTGTGATGAGTTCCAACAGCCTGAGTTTGGTAAGCTAAAAGCCACTTAGAGGAGCAGATATTTATAACGTCCTACAGGAGACGTTTGTGGTGGGTGAGCATCTTCTCCGTTCCACTGTGCAGTGGCCTCCATGAAGCAATGTTCCTGCATCCTCATAGTTGGTATGGATGCAATTGTCCAAGCAAGAGGTATAGATCAACATGATTAAGTCCAAACGGCTATGGGAATTGTTTCTTTTAGTTGAATTTTATCTTTCAATAGTTTCTTCCTTCCTAATTTTGCAGCATCTGTAGTTCAACTTAAACCATTTATCACATTGTAGATTCCTGTTCTTTAGTCG

>ONT.15413.7 LG10 11281465-11286422

ACTTTCACAGTTCGCACTAGCCGAGAGGGGAGATGAGGCGGGAAAGGTGTGGAGAGGAGGAGGAGATGAATAGGGAATAGGGAATAGGGAAGAGGAACGAGAAGGAGGGAAAGAGAGAAAGATGGGAGGAGAGGTGGAGAAGTAGAAGAGGAAGGATAACGATGCAGCAGCAGCAGCCGGAGATGGATGCCAGATGCAGCCCTTGAGGTCCAGCTGTCCAGTCCATTTCACAGATAAGTGCTGAAAAAGGATGACTAGGAGGATGATTTATATGGCTCATGTTGCTTGCATACTTGTAGGTTGAATGCAGAACTTGAACTTGTGTGTGTGTGTGTTTTTTCTCGATTGGTACTCTCCCGAATGTAATACTGCTTTTCAACGTGTAGCAGAGAGGGATGGCTAGAAGTTAAGAACTGAAGATTTTTGGGACACCGCAGTCTGTTGCGCGCTTTCCTTGGATACAATGAGTATACAGAATTATAATTGAAGTTGCTGTTGAAACCTGTTTGTGAAGCAGGAGCTTTTATTATTTGTTTGGTAAGGTTACCAAAAGGTTTGTTTAATTGCAACCTAATCTTTC

>ONT.4890.1 LG02 108471931-108483787

AGCAAGAAATGGAGAACCTGGTCACCACTTCTCCTCTTCCTTTTTGGCTCTCTTTGTGAAATGCCCAACGAAAAGGAAAACATCATCGCTTCCTCTTATTACTCTTCCAATCTCTCTCAAGAATTAGAATTTGGCCATGGCTTCGAAGAGGATCGTCAAAGTGCAGCTCAAGGGTCTGCAAAAAGATCCTCCAACTTCCAGGGGCGGTGGCCTACCAGCAATTGTGTTGACCAAACAACTCAGGTAATTCCCATTAAATGCATTTTTGCAAATGAAAACACTATTTGTCCTTAGTAATAAGAACTCAATTGGTCAGGGACATGGGTGCATGTACAAGCACCAAACTCACCAGTGATTGTTTGAGAGGTTTTTCGTGAGATACTTAGGAGTTAGGATATATTTGTGCAATATTGAGGATTTCTTTATTGTACGGAAAAGTTTATAATTTTCTGCATTTGGTATTTTTTCACTGTTTAGCGCAGCATTTCTCTATGTGGAATGAGGAAAAGCATTAAATTTTCCTCGACTCTGCCGAAAAATACATTTCCTATTGGAAAGGGGTAGGAGTCACTTTTAATGGATTGTTAGTGTTAAATAATAAGTGAGTTTGGCATGTGCACCATTATAATTGATTTTTCATTGTAGTGGATAAACTGTTTTTTCTTTTTC

>ONT.416.3 Contig01188 18629-19291

CAGCAATACCCACCATCTTCAAAGCATTGGACATCAAATCGGGAGTCTGCCATTGAAATTCTTCAAGTGCAAATTCTTGAAAGGCAGTTTTGAAATTTGGAGCAATTCTATGGTGTAAGAATCTGAAGCTATGTGCGAATGGATTATCAGATCATTTGTGGCATTATGATCGGGTAGTTCGTATTAGAAAGTCCAGGGAGCTGAATTTTCTTCAGTGGGAGTCCCGCCATGTATGAAACCTCATCTCTGCTGTTAATGCAGAAGGGGCCCCTCTGCATTGAAGGCCAACTTGAGTTTTCATGAACGGCTCAGATTGCGATGCCCACAGAAGAAAGCAGGATGTTTAATTGTCTTTCTTTTTTTTATTGCTTTCTTATATGTAGATGAGAATCATGTATAATTCTTTTCAAGTGATGGTTAATATTGATGTTGGTGTTTAGCTATAACTTTTTATTTGTTACATTTTTTATATTTAAATTAGAATTCTGTTTTATTCGTTGC

>ONT.987.2 LG01 14244904-14253649

CAGCCAAAGCATGTTGGAAATGATGTGTGGGATGTTGCTATGGAAAGGAGGGACAAGAAGCTAATGGAGAAAGCGGCTGCTGGACAGGATTAAGAAAGAGCTTGTCACAGGTCAATGGATTGACAAACATGGGAATAAACCGCTCATCTGATCCAGCTGAATCTCTATTGAAAGTTTGCCTATGTAGCCATTGGGACTATATTATTTTTATCGTTTGATGTATGTTCTGTTACTTTATGACTGTTCTGCAGCTCCTAGTCTGCATGCAGTGTTTGCTTCAGCGAATTCATCAATATTTGAATCTGAACCATTAATGTTTGAGGGGTTAAAATTTG

>ONT.7268.2 LG04 7725801-7727642

CCTAGAGCGGCGCAGGATAGCGCTCTCTCCGTCTCTCCACCTTTGAATCTCTTCTATCAAAACCAATATTGGAATCCGATCAATACATCTTTCCCATTCACGGGTCACGAAGATCTCCCTCGCATTTCGCTTGATTCCGAAAGATCAGATCTAAAAATCCATTCTTGATTTTTCTCGAATGAAATACGCCTAGGGTTAGGGCTTCGAAATCTCTCTGCTGATGGTCCTCTGATGATTTATTGGATCAGATCGTTCATCACATAGTTTCCAACTTACTATGAAGGTAATGCTGACAGAAATGCTTATCGACGGTCGAGTTCTGATGGGATTCAATCTTCAGTCCTCAACATTATCATAAGCTAAAGCTACCTAAGCTGCCATCTCCTTTAATGTCTCTCCATTCTTGATGGAAAGATTCAAATGGCAGGGCAGCTGACATTTTGTATAGAATCTTATCAAATTTGATTTATAATTTTTGTACAAAATTCCAAATTATCTATTGGATATGTGAAAACGGACCATTCGTCCATCTAAAAGCTGATTGTTTTCCTGAGGAATTGGAGGTATTTATTTTTGTAGC

>ONT.17604.1 LG12 12524692-12528851

CTCCCGTCATTTCTTTTCCCTCATCTACTTATCTCTCTCTCTCTCTCTCTCTCTCTCTCTCTCTCATCTGCTCATCTCTCTCGTTTCCTTCTCCCGCCAACGACGCTCTAAAACCCTCTTTTCTTTTCCCTCATCTGCTCATCTCTCTCTCTGTCTTTCTCATCTACTCATCTCTCTCTGGATCGGCGCTCTCTCTCTCATCTGCTCATCTCTCTCATTTCCCTCTCCCGCCAACGACGTTCTAAAACCCTCGTTTCTTTTCCCTCATCTGCTCATCTCTCTCTATCTCTCTCATCTACTCATCTCTCTCTGTCTCCCTCACCATCTCCATCTCCATTCCCTCATCGGCATCATCTCCATTACCACTCACACAGACCTCACCCTCTCCATCTCTGTTGATCTCAAAAAACCCATCTTTGTCGATCTCCAAAAACCCATCACAGTTATCGGGCGCAGACCCATCCCACTGAAGAAAACTGGTGCCCCCTTCTCCCGTCGTCTTCTCCAAAGCCAACACGAGTTTGACCCCTTTTCCGGTCGTTTCTCCAAAGCCAGCTGGAGTTTGCTGCCTCTGCATCTCTTCCCTCTCTACAGATTGATGGATGCTTTGGGGTCGGTTACAGACTAGTTAAATGTATTTTGTTTTTTTAGTTTATTTGTATATGTATATTGGACTGTATTATTATTGATTGTATATTTGTTTAATGAGATGAATGATTTGATGAAAAA

>ONT.16527.1 LG11 2593414-2597484

ACGACGCCAGCAGCTGCAACTTTTCCCTTTCTCTTTATCTTCTCCATTTCTTTCCAAAATCAAGTATTAAAGGTCGTCAATTGGTAAGCCAACACCGCCACGCAAACCATTGGCCTCTTCTTCACCTTCTCCGTCGTTTTCACGTGTTTCCATGACCCGCAGTCCCTTCTACCCACTTCATCTTCTTCGTTGGACCTGCTAGAGGTACAAACCCTGTTGCCTTTGCGGAAGAGCCATATGAGACAGTTGAAGAGCCGCAACTCGTAGGAGAATACCATGCTGATGATGTCCTATAGACATCGGGCTGAGCCATGAGATGCGTGACACCTTTTGGGGAGTGAAACACTGTTATGGGTTCATTTTGTTACGCTGGCCTTTGTTGGCCTTTATTTTTGGGGATGTTATTAACTAAACTCATCTTTTGTATAAATACTTAACACTAATGCTTGGGAATGCACAGTGATACATTGCTCACTCTGACATCATGTTTATTAGTTTTAATCATCAGTTTCTTTTATTGTAGATTTATTTTATGG

>ONT.15958.2 LG10 72254063-72257376

AGCCAGCTGCCGCTTTCCCTTCTCCCTTCTCCCTTCTCTGATCGATCGGCTCTCTCTCTCTCTCTCTACACTACAGCATCTCTGCCTTTTTCAACTGTTGTAAACACGTTCGTGGCCCTTAACTACCCTCAGCATGTCAGCTTTTGTAAGATTTGGAAGCCTTGCACCCAAGACTAAGAACTTCATTGTGGCTGGAGGTTTGACAACCTTTGTTTTTGGGGTGTATTTCTACACCATGAGGGCTGTTGGAGGTACTGATGAGCTGCAGGTGGCAATTGATAAATTTGAAGAGCAGAAGGACAAAAAAGAGATTGATACGAGTAGATTATCAAAGTCCTGAATCATCGCCTTTTTCACTTTATTGGCATTTTCTTGTTAGAGTCATGTAGCAAATACTGTTGAAAAGAAAAATAAAGGAGTGAACTTTTGCAT

>ONT.14983.3 LG10 61667782-61669951

AAGCTTTCATCTTTGCCGCTCGTCTCTTCCTCGTCTCTTCCCCATCTCTTGTACTCTCTGCCGTCTCTCAATCGCCAATCTCCTTGCAGGTGATGGCTCTCATCCCTTTCTCCTTCCAGATGCCATGCACTATATATGGTCTGACTTGATGTGTGTTTAATGTTTTATATGATAATTTGTGGATTTATGGAAGCTCTATTTTGTAGGCTTTAAATGAAGACTGGTTGACATGGTTTCGCAGTTGCATGTGGTTGTGATTGGCATGAGAAGTTGAATGTTTGTTTTGGATGCTACCCATTGTATGGAATTAACGGCAATTGGATTTTGTAATGTTGGTGGTTGTTAGAGGTTGTTTAGTTCTATCCATTGTTTTTTATGGGATTTGATGTTGGAGGTTGTTATTAATGTAATTTGCTTAGGTCTATTGGATTTTGGGCCCGACTCCAATGTCTCGAAGTTATTTGATTTTATGTTGGATGCTGGAAATTGGTGTTAGATGTTGGATTTTGCAAATGTGCTTCGAAGCTGTTGAAATATTGCCTTTTTACAAATGGATATTCCAGTATTATAAATGAATGTATAGAATGTTGGTTGTGTC

>ONT.5954.2 LG03 37422028-37423508

TTTCCTTCTTCTATCGGTTACTACTTCACTTTCACCAGCGACCACCGGCACACCGGCGACCACTAACAACTACTCAAGCATCAATAGACATCGACGACGGATATGGGATGCTGCCTGCAGCGTTCTCTCCCCCCTTCTCTCTCTCAATGCTATCGTTAGTTTTAGTTGGGTTTGTTTCTTCTTTGCATTACTATGATGTGGGTTGGATTTATGGAGACAGCCTATGTTGATCATGTGGGATTTGCTCAAATGAAATCATGCTTCTTCCATTTCCAACTTAGACTTTGCAGAATTGAGTTGACAAGGTTTGCTAAATTGAAGTCATTCTTCTTGAATCAGCATGTAAATCCTGTGTGGTTGAAGTTTCAAGTTACTCAAAATTCAGAATACATTTCTCTCCAGCAGTCCCCTTGACCGGTGAAAATGGTTGTGACAAGCTTATTTGGAAAGATGGGGTATTCATGGACGAGATCGAATATGCAAATTCCGCGATTAATATATCTAAAAAATTTGTCATTTGTCAATGAAGGTGAAGTGCTCTAAATCAAGGTTGAAGTCGGTAGATGCAGAGGTGGAGCACATGAACACATATTTGTTCAGCCTGGAAATTGTAGAGATGCTGCGCAAACTTTTTCATTTTGTTCAAATTATGTAATTTATTATCTTTGTAACCTATATGTTTTTTTAACATAGGCATGTGATCATGTGTGGAACATATAAGTGATAACAAAGGATCAATGAATGTAAACTTCTTGATTATAGTG

>ONT.12952.6 LG08 43208021-43208614

CTCGCGCCCCTGCATGTCTCGCTCCCCTGCGTACCAAGCAGCCCTCCATCTCGACCTCATTCATCTCTCTGGCCCTCTAGCTCCTTCTCTGTCTCGCCATCCCTCTCAGCCGATCCCTCCTCTTTCATTCCATCCCTCCTCTCACTCATCCATCGCCGGACGACTGAACTTCCTCTTCAACCGAAAAGCCTCAAATCTCCAACCCTTACCCTGGTCTCTCTCGAAGCATCGTCCATCCCATCATCGAAAACCCTCTCCATTCGAAAACCCCCAAATCAAAAGTGTTCAGACCTTCTCCCTCGACCTCTCTCGACTCTCTCAGTTTCCAGGCAACATAAAACGAAGGAAGGGAACAAAGAAGGGGAGGAAAAGAGGAAGAAAAAAGGAGAGAGGAAGAGAAAAAGAAATGCCTTGAAAACTCAGCCCTCAATCTGATTTGTTGGAGAAATTGACGGATAAAAAGAACTCTGCTTTGACGAGAAATAAAGAGC

>ONT.1507.5 LG01 109766854-109798878

TCTCTCTCTCTATGTGGGGTAACCCGTATCGTCTCAAGTGACTGGAATCGAAAAGAGGAATTCATTGCAGAGGGGGTTTTCTCGAAAGCGAGAAGTGAAGGTTCTGTTGGTTCCTTGGGAAGTTGCTAGTATTGTGGATTACTTGATAGAAGGGACAGGGGTGGCTGCTGCCATATTGCTTTTTGGGTTTTGAGTCAGGACGTGGATAAATTTCAACAGAAAGCTCAAAATGAGAAGTTCTGCACTTTCTTCCCTGGTGGCAAAGCTGAAAGAAATGTTCAACAGAGAAAACGGATAATTGTAAATGTTGATGTGATTTATACTATTTTCAACAGATGCATGTATTTCATCTCCCCAGTGGTTGGATGAGAGTTCCTAATTCTGAGTTTTCCCTAATATCTGTTTATCAAAGTACTTGTTTTACATGTTCTTTTGTTGAAAGACATGACACAACTATCAGGGACCCTTTTGTAGGCCATTTGCAAGGATAACCAACTTTCATAAATGTTCAATGCTCGCTCATTGCTATC

>ONT.5307.1 LG03 25766630-25769792

ATCCATTTCCCTGCAACGGTTCCAGCAAGAGCTGGAACCACTCCTCTCTCTCAGCCACTCTCTCTCTCCTCTCTCCCTTGGCTTCTCTTGATTGTAAATGGAGATTTGATCTCCTCATCATCTTCCATACCTTCCAAGCTTTCCAAAGATGTTTTTTTCATGGATTTTGAGCAAAGGCAACTTCTCTTTTCAAGCTATTCTTCTTGGTTCAAGGCCTTAAAGGGTTTTCATGGGAAAATTTGGGGGTTTTGGTGGGTTTGGAGCTAGAGACTTCTTTGAGGCTTTTGTGGAGTGATTTGTGCATCTTCTTATCTAAGGTTTCTTGTTCTTCCTGCTTTTCAGATGTGTGGATCGGGGGAGGCGGTAGAGCCACCGGTCATCGTGAGAGCCTACTTAAGGATATCATATAGATGTCAAGGTGGGCAACAGAGTTCGTAACAACTGTTGGGATTAGTATTTGTTTAGACATTTTATTTTGGGTATGGCTTTTATTAGCCTTTTCTTGGGTTTTGTTAATGAAAATACTTTGTTTTAATTGTAACTTGAATATTCATGGGAAATAAGAATATCCTGTTACTGTCA

>ONT.11157.4 LG07 5637534-5638879

GAGACTCTATCCGGCGATCGTTTTGCTCTAGCGAGAACGTCTCTCTCTTCGGTGACGCTCCTTCTCCTGCCCTGGGCTTTTCTCTCCAGCGACTGTCTCTTTGAGGTTGTGACGAGGTTGGAGAAAGATTGGGTAGGATGGGATTGATTGGAGTCTAAGTTTGAGATGGGTTCAAGGGGATGGCTTGGATACAATTTTCCTGCAGATTTTCATTCTCTTTGTGCTCTTTAGTTTTTCTCCCTCTGCAACGGGATAAGTGTCGATGGAGAAGGCATGTTGAGAGTTGACATTCTGGATCATTTTGAGTTGTTCATGAGACCTGACATTTTGGATCATTTTTTAGTGGTTGAGAGTTGATTTTATGGTTGATTCTGAGTTGTTGTATTTGGCATGTAATTTGATTTTTTGTGTGTTTGGAATAGATTTTTTTTTGGATTTGATATGGTGTGTTTCAAATTTGTGGTTTGAAATTTTTTTGTAGAAATGAATGGATGACTCGTCATGTAA

>ONT.431.1 Contig01223 36506-45690

TCAGTCGCTCATCGCTCATCTCCTCTCCTCTGCTCTCAATCTGGGCCTCGATCGCTCATCTCCTCTGCAATCTTTCTCTCTCTCTCTCTCTCTCTCCTTCCCTTCTCTCTCTTCTCTCCCTCTCCTTCCCTTCTCTCCCCCTCTCTCCCTGCGTTCACCCTCTCTGGCCAGAGCCCTGTGAAACCCTTCCCACTGATTCTCTCTCTCTTCTTCTCTCCTCTCCCTCTCCTTCCCTTCTCTCCCCCTCTCTCCCTGTGTTTCCTCTCTGCCCAGCCGCCAGCGCCCTATGAAACCCTTCCCACCTACTCTCTCTCTCTCTCTCTCTCTCTCTCTCTCTCTCCAGTGCATCCCTCACCTCACGCTTTTCTCGTCTCCTTCGGCATCTCCCTCCTACGCTCACGGTCGCCAGCGCATCCTCCGGCCGGCATCCATATGACTCCTCAGGTGAGTACTACTACGAGGAGCAATCAGGTGAGGAGATCCCAGAGGATCTCCAACCGGCTGAGGAGGAATGAGAGTAGCGTGCCGATGATGTAGAATAGCGTCATCATTTCCGGCACGAGAGTGATTCATCGGGACTTTCTTAGATTTTATTTAATTCTTATTTATCTCTCATTGAATCTTTCATATCATTGACTTCTTAATTGTTTTAAATGGCATTGAGTATTGGGATGGCCATTATAATAAATTAACTTCATTGTTTAAGTTTTATTTTATTCCTTGTGCAACTTTAACTATCAATGAGATTTATTATTCATTGCTTCG

>ONT.15186.1 LG10 87483630-87487500

AAAGGAGAAAACACTCTCCTCTTTCCTTTCCTCTTCTCTCCTCTCCTTCCATGACATCTTGAAGAACTTCACATGGAGGCTCCTTTTGCAAGTCTAACCCAGCCAAGCAACATAATCTATTTTGGTGCGGATCCAACAGTGGCAGCCCCTTCATCAGTCACCTTTCCTTTCTTTGAGCTTCAAATAGAGATTTTGGTGGGTAGTTTGGAAGAGAAAACTTTTGATTTTGGAGCTTGAAGTGATGGCTAGGTTGCATTTGATTCTTGATTTGAGGCAAGATTCGCTAGTTATTCAAGAGTGGGAGCTAACGACACAGAGTAGCGTCTAAAGCTGCAGAACTCATAACATCATCTTTTGGGTTTTTGTTATTTTGGGGAATTTCATCTTTTGATGATTATAATTATTGGCCAGTGTTTTTTCACGGCCTTATTCTGGGATGTAATTAAACTTAATATTTTGTTAGACCTGAATACTAACTGATAGAATGAATTCCTTTATGCTATC

>ONT.249.3 Contig00712 5911-95870

ACAGGAAAGGAAGGATTTTTTTTCTCTGTAGTTTGGAGTTTGGACAGTGGACAAAAAAATAACTTCCCCACCTTCTCTCTCTTACATGCACGCAGAACCTCGAAATCTCTGTAGCAATGGCGGCATGCGCGGCCGCGGCTCGTCAAGCCGCGTCTCTATCTCGGCTCTCATCTCCCAAATCGGCTGCTCAAGCCTCAAATCTAGTGCAAAGACGCCGTCTTGCGGGAGGTGGAGATCATTATGGATCCCCCAAGGTTCACTGCTGGCAGGACCCATTAACTCCATCTAGATGGAAGGAAGAGCATTTTGTGATTGTTTCTTTATCTGGTTGGGGGTTACTTTTCTATGGAGGCTACAAATTCTTCTCTGGTGGCAAGAAAGACAAAATTTCATAAAATCACAGGAGCACTCCACTAGGCTTGATGGTGCAGTGTGACAAGTTTCGTTGTTGAGTCACATTTGAAACTAAATTATTTTCCATAACTTTGCAATATGTAATGAACGTATTTTGGTTATGAGATGTCGATTTTGAGTGACTAGTTTACATAACCCCTCGTTTATGGTTGAAAAATGCTGAGACCATGTTGCATGTCCCGGTCATGTGGGCAGCCATTGTTGATCCTTTCTTATTTCTGAATGGTTGCAGTGTTCTACCTTCTTTTATGG

>ONT.11344.1 LG07 54543124-54551945

TTCTCCTCCTTCTCTCCATCATCAAGCTAGGGCACTCCCAAGGATTCCAAGGCTCCCCAAGCTCCATAGGCATTCCCCACCATCTCGATCATCATTCTCTTTCCTCATCTTCACCTCTAGATAAGCATATCCTTCCCCCAAACTCATGTAGCTTAGCTTCTTCAATACTAGGATCATGCATTCATGATATGGTGAGTTCTTTCTTCTCTTTTGGCATGTAAGAGAGCATTCTTCAACTTGGTTTGAGGTGGGGATGTGTGAAGGGCAAGGAATGAAGAAACCCTAGGCTCCAAGAAAGGGGTTTTTGCACAACTAAGCTAAGGCAAATTATCAACATACTGGGGAATGCAAATGCAGTTGCTGAGTGCATGGAGGCTCTAATAACTGTGCTGAAGCATGCAGGCAGGAGAGGAGAGAGAAGCATCCTCGTCATGTTCCTCATGACCAGGTAGAGAGAATAGATGTTCAGGTAGAAAGAATAGATGTTTGGGAACGTTGTTAGAAGCTGGCTTCCTCACAGTACTCTATTTGCGCATATTAGAAAAGGCCCATGACTCGTTTTCTTTGGCAGCCATTAATTCTCCTAATTGCCTCAAATAACTAGTGATTAATTATGGCGGCTACTCATGATATGATTGCAAAATCACAGAAGGATCAAAAGGTTTCACATGGTTCATGGGTATTTTTCTATGGGCTGCTTTTGAATGTTCCAAACCTCAACTGGTGATGACTCTATTTTGCCATCCGTAGCAGTGATGCATCTAGTTGGAGCTGAAACCTTGAAGTTCTGACAGAGGCAGAAGAACTCTTTACCAGTAGGGAGCTTTTCGGGTGAGGTGCACCCATGTATGGAGAAGAAGGGAGCTTTAAGCATTCCAATGAAAATCTTTTATCTATTGTAAGCTTTGCTGTTTGCTGGGGTTTTTGGATTGTAAAACTGTACTGCTAATTTGAAGATTGGCATGATGCGCCCCAACATCAATCATCAGGCACTGAAAATAATACCATATTGGACTATCTTAACCTTCAATTCATGTCCTTTAAATTGATAAAATGGCAAGGTTCGGTGTTACAC

>ONT.483.2 Contig01419 77815-90756

GCCCTCTTTGTTAGTCTTTTATGAACGAAGAGGTCTCCTTGGCCTCTCTGTCTCTCTGCGCTCTGCTCCCTCTTTCTATCTCTCCGCCCTAAATCCAAACCAAAGTCCAAGATTTCGATGATCCTCTACAAACAATAGAGAAATTGAGAAATCCGTCCCAATCTCCTCCAAGTTCTTCTTTACAATCAGTTCTGGTTTTCGGACTAGTGGCTGCTACTTGCGGAGGAGTCTTCATTCGATTTCTTCTTCAAAGCAGCCGTGATTGAAGAGGATTACTGACTTTGCTTCTTCTACGATATTTGATCAAGATCACATTAGACAACTATGAGGGCATCAAAACCTTTCTTTTCAACTGCTCTTCCAAAGCACCTAGCTGCTAGACTCTTTTCCAGGTACTCAAGAGGTAGTAAAATTAGATCAAAGACGTGCAACTTATCCAAGGATTTGACTCAAGATAGATCGGAATGGAGAAACAAAATTCATGTAGCCGACCCTAATATAGGTGAAACAAGTCTTTGAAGATGGTGATGATGAAGTAACTCTGAGGAAATAGATCGTAACTGCGTCATTGTAGCATGTAAAAGATTATGGGCATAATTTTAGAACTGTTATTATTGTTTTTATATTTTTAATTTGAATGGAATTTTGAAGTGAAGATTTTATAGTTTTCCTCTTAGGGATTACCTTGTTTATTTCGATCACAGACATTTGTGGATGTTAAAGATTTTTGGAAATGATCAAGTAATAATATTTGTGTATTTTAGAGA

>ONT.16551.1 LG11 18393225-18396321

AAATGAAATTGTCTCTTCTCCCTCGACGATACTAGCATCTCGTGGTGTCTCCGTTGACGAAACCCACTCAATGAGATCGGCGGAGAGACTCTGCTCCTCTTCTCGTCTCATCTAGTCTTCTTAGTTCTTCGTCGCTCAGTGTCAACATTGTCGGAGAGTCTGCTTGTCCAAAATAGCGTCGCATCCCTCAACGATACCCTCCTGTGTTCGTTGCTCTTTCTACTATGTTTACTAAAGGTTGTGAAGGTTAAATGTGAACAAATTGTAGAGGGTTCGGAGCATTCAAGCATTGGGCAGGAAAATTTTAGGATGTGGGTTTTGTTGTTTTGGTGAAAGGTTTGAGTGGATTATGGATGTATATTTATGTTGGTACAAAAAAAAAAAAAAAACATTATTCAGTTGTTTCACACTTTTTTGGTTGTGAATTGTTGCGTGGATTATGGATGGGCATTGGGGTTTGGTAAAAGTTTTGAACTTTTCAGTGGTGTATGTAAAACATCGGATTGCAGAAGAATCCCCTCAGCTACGGCGTTAATTGCCTCACAGTGATAAGCTCATGGCAGCCATCCAATAATGAGTTAACTTCTACTTTATTCAAACTCATAGCTTGCGTAGGTTTCTCAAACTTTGAATAATTGCACCAATAATGGTGACAGTGTTTGTTCAAAGCGCTACATTATCTTGTAAAACACTCTATTGCAAAGGAGTAGATGGCCAGAAAAGAGTACAATATTGTTGTGGAAGTTTTTTTCCCTTGGCTATTTG

>ONT.6932.1 LG04 77786066-77786646

GATTGAAACAGTAAGGGTAACGAAAAGACAAAAATGAGGGAAAGAAAGAAGAAGAGGAGAATAGGAAGAGGGAAAGAGAAGGAGGGGAAGAGAGAGAGAGAGAGAACCAGGCAGCGGTAGCAGTGGTTGCGGCGGCGGCGGATGTGGCGGGCGGCCAGGGCTTAAGGTTCTGGACATCTACAAACCAAGATGGGAGTTTGAATGCACAATTTGAATGTGCCGAAGGCGCGTCTTTGGACAAAAGATTCAAATTCTTCAAAGGGGCAGTCATGTGTTATGATTTATGATAAATTATCACCTTATTTGAAGGACATGTTTGTTTGTTTGCCTAGAATTGAATTTTGAGAAATTTTAAGGAATGTAAGGGTTAGAATTGAAAGGTGAAAATTCATAAATGGCTATCAAAGACTCTTTGCTACCAGGTTATTGGGGATCCTGCTTTTGAATCATAAATAATAATTGAATTTTTG

>ONT.9670.1 LG05 78141569-78143206

TTGGTAAGAGGTCAGGCTACGTAAAGGGGTATGGGATATACTCGAGGACCTCTTCCTCTTCCACACAGTCTCGGTCACCAAACCCTGAGGTGGTCGCCCTGCGCGAGCAGCTAGCTGAGCAGGGTAGGAGGCAGGCTGAGCAGCTAGCTCAGCAGGACCGGAGGACACGCCAGCTTGAGGCCCTCGTTCAAAGGCTGGCAATTGTGGCAAGAATAGATCCGGCCGCCTTTCTTGACACCACGGGAGAGAGCACTTCTGCTGGGGATGGCACAGCTCCTGGAGGATCATGATGCGACTCTTTGATTGATGGACGCTTTCTGATCGGATGCACACTTTGGTGTTGTTTTGTTGCTTTTAGTTTATTTGTATATGTATATTGGACATTTTTATTCGACTGTATTTTTATTAATTTAGACATTTTTATTCGACTGTATGTTTATTAATTGTACTGTTTATTTAAGGAGATGAATGATTTGATGA

>ONT.8525.1 LG05 45637135-45638405

ACTTCTAAACCCATCTCTCTCTCTCTCTCTCTCTCTCTCTCTCTCTCAATCTGTTTGTGTGTGTGTGTGTGTGAGAGAGAGAGAGAGAGACATAGAAGACTTAACCTCATCCAGAAGAAGATAGAGGAGAAAAAGGTTAAGACCATGTATGGATTCAGCACCAAATTCTCCAATTCCATCATGAGGACCGGAATCTTTGGAGGCGACAGAATCTTTGCAGCCGGATTTGTGATCATGGATTCCAATCTAGCCGGCGTAAGACTTTTTAATGCAATGGTCAACGCAAAATGTTCGCCTTAAATAGGCGCTATCCTACCTGAGCTTTTATCTATTTTCTTCACTATCCACCCATCCTTCCCACTCCTCGCACTCCGGAACTTTCTTCCTTCCAAAGTCACATTTGCCTCCTTTGTTCCTATGGCCGATGCTGCCATCTTCTTGACCTTGTAAGGCCTTTTCTTGACCTTGTAAGACCCCGCATGAAGGTGGCTTTGTTGACTCTTAATGGACCCAGAGGCTCTCAATTAGTACCTCCTAATTGTGAATTAGGGTTCCATCTTTTCCCCATCCCATCAATCCATTCTTCTCTACCCCTGTCTATCCCTCATGAGCTATTCAAACCCAAAGCAACATTTTCATCCTATCAATCGAGATCTTGACTGTTACATGTTTCTTTCTTTTTACTGTCTATGTTTGTGTAACTTGTCGGAGAGTGAGGAAAAAAAAAGGGTAAATAGAGAGAAATGATTGAAATTTAGCTGTAGTATTGTTTTCTTCTCATTTCTTTTAGGGTTTTGTGCTAGTTTTGTAGCATGTGTGCAGAGAAATATGCCTTGCAGGCATCAGTTTCGTTGTTTTATATGTTGTTTGGGTGATGGATGAGTAGACAGTAAATATTCTCTTAACTCTCTGTAGAGTCACCTCGTGACTGTTCAAGACACTTTGCTATTACTTCCTTTTGTTCT

>ONT.3788.9 LG02 112772954-112777940

CTCAATGCCCATCTAACCGGAAACCTTTCACGCTGGTCTGCCCGCTCTCATCGATCCACCGCCGGACCTCGAGCCATCGATCGCCGAAGCCGTCTCCATCTCTCAGCCCTAAGAGGATAGCTTCTCACACACCATCAAAGGCCTCAGTATTGAGATTCTTCATAAAACCCATGTCTAAATCGGAGGGACTCAAGGATTTCTCATAATCTTCATCTCGGAACTACAGCTATCGGGCTAATCAAATGGGATGAAGGCTGACTCTATCCAAGGGATGATGTTCGTAGCTCAAAGATGTTGAGACAACCAAAAACAACCCCACACTGCTCACAGCTATTCACAACCAGCCTTATGCGACTATGTAGTAGAGCTGAGGATGGAATGCATGGGATTTGGTGATTAAATCGGTGGACGAGATCAGATCTATCGGTCGAATATCAGGTAGACACATTTGAATGGTATTGACGTGCACATGAATGCGTTAAAGCGAGCCGGCGGGATTCTCTTTGTAATTCATGGGTTTAGGTTTCCATCTCATCTTCAATTAAGAAGCAGCAGCGACACGACACAGAG

>ONT.6079.1 LG03 48441924-48447617

GAGTCCCATGGTCCTCATTTCTCTCTATTTTTTTCTTTTCCTTTTTTTCCACTTTATCCTCTCCATAGAAGAAATTCCTTCCTCTCATCAAGTATCTTGTCGGTACATTGACACAGACAAGGACCCAAGAATGTGAGAGATAATTCGCTCCTTCTATGGCTAGATTGATTAGATTTTTGAACCAAGCTCTTTCGGGCCTGTTTCTCTCCATGAAATTGAACACACAGGAATTCTTGGCAGGCTCCAACAAAGGATCATTAGAGTCGAAGGGTCAGGGATGAGATGTAACCATAATTTTCTTAGGATTTCAATTACCTGGGTGGTGATCAGATCTAGGTTCCGTTGGCCCTGAGGAGGAGCACGAGGAGGAGGCTATAGGGCAGCCTGTTGAGCGTGAATGGCAACCCAATGATGTCATCTAGACATCGGTTTGGGGTTTGAGATGCGTAGCACTTGGGAGACATTATCTTTTATCTTTTGGGAATTGTTATTAGCCTCTGTTGGTTTTTTTTTGGGGGGGGAATTGTAAAACTTAACTTTACTTTCTGTTTAGTATGTTGCACAACTTGAAACCTTAATTATGGTTATGCTAGTTATGTTCTCCCTCTGATATGAGTTCCTTT

>ONT.10651.1 LG06 27906866-27909328

TTGCACGGCCAGAACTCCTTCCAGCCGCCTCCTCTCCTTCCTCTCTTAATCTTCCACCGAAAGATCATGATACTAAAGGTGTTTCTCATCAAATCCAAGCCTAGGGAGACGTTTTCCATCATCTTCTTGCTTCCTCTCCATCGTTTTGGTGGAGAACTGCACGTGGCAGCCCCTTAACTACACTTCAACTTCTTCATTGGGATCTCTAGAGGGTTTTCTTGTGTAGATCTGGGATCTTCTTGCGGGGATAGTTTTCTGCGATTTCTGGTTGTGTGAGAAGGTTTTTGGATTTGCTTGGAGCTCAAGGCAAGTATCGAACTCCTTGTGCCCAAACAGGACATTGTGGCGGTCCAACATGATCCGCCGTTTGTACGAGTGGTAAATTGATGACATAGAGTAGATCGTCTGAATCTGCAGAGTCACGATAATATGTTGGGTTTTATCTTAGGGGTTTTACTTTATGGGCTTTAATAATAGACTTTGGAGATTGTTTAGTTTGTGAACGTTGGCTAATGTTTAGGCCTTTTTATTTGAGACTTTCAATTAAGATTTATTCCTCTTTTTATTTCTGTTTAAACTTGAATCTTAAGTTATGGAAATAAGTTTCATTCTGACATCAGTCTATTAATGCC

>ONT.14993.1 LG10 63258704-63261570

CCCCATTTTTCTCCCGTTTCTGCAACATTTCCAGCTGTGGCTGGAACCCAGTTCCAGCCACTCTTTCCCCTTTTTGTTTCCCATTTTCTTGAAGCCAAAATGGAGGAGTGATCTCACAACTTTCTTCCCCACCTTCTAAGCTTTCCAAAGATGTTATTTTCATGGATTTTGAGCAAAGGCAACTTCTCTTTTCAAGCTCTTCTTCTTGGTTCAAGGCTTTAAAGGGTTTTCATGGGAAAATTTGGGGGTTTTGGTGGGTTTGGAGCTAGAGACTTCTTTGAGGCTTTTGTGGAGTGATTTGTGCATCTTCTTATCTAAGGTTTCTTGTTCTTCCCTACTTTTCAGATGTGTGGATCGGGGGAGGCGATAGAGCCACCGGTCATCGTGAGAGCCTACTTAAGGATATCATATAGATGTCAAGGTGGGCAACAGAGTTCGTAACAACTGTTGGGATTAGTATTTGTTTAGACATTTTATTTTGGGTATGGCTTTTATTAGCCTTTTCTTGGGTTTTGTTAATGAAAATACTTTGTTTTAATTGTAACTTGAATATTCATGGGAAATAAGAATATCCTGTTACTGTCA

>ONT.4721.1 LG02 65973347-65975549

TTCCTTTTCATTTCTTTTCCTTTGCCCATTTCCTTCTCCATTCCTATTCTCCTCTTCTTCTTCAATCACTACAAGAGTGAGGGCATTTGTCTTGGAAGCTAAGGGTTCACCCATCCTCACCCATCTTCATCATCTTCTTCTTCCTCTCTCCCTTAGTGACAAGAAACCATCTCCACCATTTCCTATTCTCTTCAAATTCGAAATTCTTGTTAAGAGGGTTTGGAAATTTGGGGGTTTTGGTTTCTTTTGAGCCAAATGTATAGAAGGAGCTTGGAAAGCTTAATTTGAGGCAAATGTAGGTGCAGCTCCCTGTCCTGAGGAGGCCGAGGAGGAAGAGGAGGTTACCCAAGAGGTACCTCCAGGACTAGAGGAGTGGCCAAAACAAGGGCCTGCTTAGAAATTCTCTTTGGAGGGCGTGAACATATCTTTTGAGAGCCTCATTAGAGTGTGACTAACCCCTTTTTGTGTGGGTCACACTTAGACTCCGGTTATTCTTTTGTTTATACTTTTGGTAGTGTTGTATAAGGTTCTTTTATGCATCCTCTTTTGTAAAAGTAATATTTCTACTTGTATAATTCTTTTGTATCAAACTTGAACAACTATTTAGATTGAATGGACAATGATACCTTATGTATGAATTATAGCTTCTGTTA

>ONT.5827.1 LG03 2960626-2964668

CCCTCCAAAAAAAAAATTCCCTCCCCTCCCTCCAAAAACGAAACCCCTCACCCCTCACCTCCCCTCTCTTCCAATAGATGATTCCTCCTCCGTCAAGGTTCCTCCATCTCTCACAGGGTACTCGCAAATGGCTGGACCCTAACCCTTCTCATCTCTCACAGGGTACTCGCATCTCCTCGTTCGTCTTCATCTCTCCAGCATCTCCAAAGCAACGAATCCATCTTCTCTCTTCTCCGTCTTCATTTTTCTAGACCTCCTCCGTCTTTGTCTCCAAAGCAGCAAGCCCATCCCTTGTCTTCTCCATTTTCATTTTTCTAAACTTCCTCATCCCAAAGCAACGAGCCCACTTCTGTCTTCTCCAAAGCAATGAGCCCATCCTGTGTCTTCTCCGTCTTCATTTTTCTGGACCTCCTCTCTCTTTGTATCTCCGGCATCTCCAAGAACCGAGAAAGAACTGCATCGGCGCCAAGACATGCTTGTGAATCTTAGATCTAAAGCTAACCAGATGGCTTCTACATTAAGCATGCCAAACTTTGCTAACAGATTGTAAAAAAAGACGTTACCACCTTGGAAAGCGATTAATGTCTCCCTGCAACGACTGTGTGCAGGTCTTAGTTCTACAGTTTAACTTCTTATTTTGGATAGTCTTTACCATTGTCTATGTCGCTTGTAATTTCATATTCTGTGGTACTTGTGCTGTCGACAGTAATGGATTCGAAAGCTCTTGTTGTTTGGGCTGGAAAATGGTAGCTCGTGTTCTGCCTTTTACTTAACTCAAGTCTCTTCAGTGAC

>ONT.7483.1 LG04 24559779-24561890

CCACATTTCACATTTCCCCCCAAATTCGTTTCCTCCATCTATCTCCCTCCACGAATCGGTTCTCCTCCTCGCACATCTTTCCTGATCATCAATCGTCTTCTTCCCGAGCATCTCTCTCCCGTCACGCAGCGCATCTCTCTCATCCTTCTCCCGGCCAGCATCAAACGAGGACGGAGAAGATGTCCATCAGCAGCGGCAGATGCGCATCTCTCTCCTGGCGTCGCGCATCCCTCTCATGGCCAGCAACCAACAATCTCCGATCTTGGCACTTGGCGGACCTGCTGCTCTGCTCCCACAAAGAAGAAAAACATAGCCCTATTTCGATCTGCAGCGGCAGCAGTGCTGTACGCACCATCCCTTATCTCCACCTGCCAGGGGTAAAGAAACAGTCCAGGTGCCCCAGATCTACTTGGTGAGCGAACTCCGACCTTTTTGACTGCGTTCTAAAATACGATCAGGTTTGCCTAACCCTCCTCTTTAATCTTTATTTTTTATTTTTTATGCTTCTCTCTGTTTTCTTCCCTTCCATTATTCTCTTTGGTCGCGTGCGTGCAATGGGATTTTCATGGGTGAAACGACAATGTAAGAGCATTATAATGGATCAACTGTCTTTTTCTATTTTCAGCTTATGTTGTCCATAGAGCTGTATCTAAATCTTATCCTTCTCCATTTATGTCGTTGTTGGATCAAATTCATTGATGACCTCCCCCCCCTAGATTGCTCTAAATCTCAAACCAAAACTGATCCAACCTGCCAGTGTGATGGGCCACCTCTCAAATGGAGCATAGCCCAAAAAAACAATTTGATTTGTGTTTCTGCCTAACCAAGCGGGTCGCTCCTGTTCACCACTCATCAAGCCTTAAAAACTACACACAATATCAGTGGCCACCAAAGATGTTTCCCACCAATCTGACAGTCCAAATCTATAATGTGGACACCTCATGATGTTATATCATACTTTAAAGTTTCAGTGTATAATCTCTGTATTGAAGCTGGAGTGGGGTTTCAGAACTCAGATATTTCACTTTCATGACGAATGATGAATCTATGTTGTTGTGGACTGTTGAATGATTGAATGTTGCCAGTGCAACAAACCCAACTAAAGGATCCCAGAGGTAGAGAAGGTAATGAATTCTTCATAGGTAAAGATGTAGTTCATGTCAGAGATCTTGTTACAAACAACCTTGCCACAAGCATTCTTCTATCTTTAGTATTCCTAGTATCGAAAGACCATCACAAAGGTTTGAGAATAAACAAAAGAAAGCAAAATAAAAGAGTGCTATTTTCAGTTCATTGATTTCTTTGATTTCTGATAGTAGGTCTCTTCTTGTCGCTGTTAATTGACTATTAATTGATTTTCAGTTATTTTCCAAGTTCTTCTCATCACTCTCAGAATTTCTTGATAACATAGTTTTAGCGTGATGGGCCATGGAGGGTAGTGTTTTTTTGGATTTGAATCTTGCTTGTGGCTGAATTGAACACATAACTTGGTCTCGATGCTAACTCTTCATGATAGGATGTCATCGTCTAATTCTTCTTCCTCCAAATATGTGCCAAAATGCTCATGTGGAGAGATGATTTAATTGACTTCTCGTAAAGTTCTAATCGAAATAGAAAGTTTTGGAGAAGTCCTAATTGGAAGGATAGAAAAGGCTGTGGTAGATTCATATGGAAAGATGAAGTGGACAATGGAAAGAATTTAGCGGATCTTATTGAAGATTTTACGAATACAATGGAGAAGCAGAATGAAATTTTATGGGCAACAGTTCAAGAGACGAAATATGCATCCGAAGTTGGGAAAAAATCAAATAAATTTAATTGTATATTGGTGTTTTTCTATTGTCTTATAAGTTTAATATGCATTATGAAAACATTCTGTTAAAATGTATTAATTTGAGAAATCAATCTCTAGATGTTTGACCATGACTAGATTTTGTATATGTTGATGAACCCAGTGAATGGATATTGTATATGTTGATGAATCCAATGTTTTTGAGAAATCAATCTCTAGATGTTTGAT

>ONT.8024.1 LG05 9574739-9576580

GGGATTTTGACCGCGTAAGACGCGTTGGCGCCTCGCTGTCATTTTAGTAAATAATCCCTCTCTCGTTTACCAAATGTCAAGGCATTTTCTTTCCGTTTTGAAACTCTCGAGTCTCAGTCCCAGCAGAAAACCCACCCAAAAATCCAATCCCTCGTCCATCTCTCCGCCAATACGAACTCCGCAAAGTCCTCAAAATCTATATATCCTTTCGAGAACCCTAGAAATTGCCTTCTCCAGACTCTCTGCATCAGAACTGGAGCTCGAAAACAATGGAGTTCTGCGGCCCTACCAAGGGGCTGGGAACCAAAAGAGAGTCTGCACACATACGGGAAGGTAGCCCACCAAGTGGGAAGGAGGCCCGCACAAGTGGGAAGAGGTGACATACCTAGGGTTCAATCCTGGGCAGCCAGGTAAAAACTGACGCTCCCTACCACCCGGGTAAGGAGTTGTTCTTAGTCCCATTTTTAATTGAATCTTATGATTCAACTATGAATATTACAATTTGATACTTATTCTAATAGAACTTATGATTTAAACATTTCATTTGAGTGAGAATAGCCACAATTGAATTGTTAATGTTTCCTTCTCTTTTTTTTATTTTCTAATTTTAAAAGGTTGGTTATCT

>ONT.4908.1 LG02 109578855-109594410

GGCGGAAAATCTCCATCTCCCTAAAAATCTTTCCATCTCATATCCTCCCTTCATAAACCTCACAAAACCCAACCATCTTCTCCCCAAAACCTTCACTCCTAGGCTGAGGTCGTGCCAGCAGACCTAGTGCATGCCTGATGCTGTTGGTTTTTTTTAAGTGCAGGTATTCATCCAGACTCAAACTAGGGGCTGTTACAATTGAATTACGCGTTCAATGACAATTATGCTAAGAGGTTCCACTTTGGACTTGAGCCTCAGAACTGAGTTGGTCTTCAGAACTACTTTTGTCTTCCTCTTTATGATTGTATCCAAATGAGGCATTCTCATCCTTGTGTGGTACATTTTTTTCCTTTGCTTTATTGGCGACGTTTGTTCTCATTACTCATATCTTTAAGTATAAATTTCATTTTTATTTGCTTAAA

>ONT.10691.3 LG06 45928644-45932235

AGTCATAACTCAGAAATCCATAACCAAACGCAAAACATCTCCTTCGAAAACCCTACGTCTTCGTCTTCAAGTCCTCTCCAACTCCTCTGTCTCTCTCTATATGGAAAAAAAATCCAAATTGCAGTGGCAAATCAGCACACCATGACCATCTCTCTTTCTCTCTCGTGGGCAGAAAACCCCCAAATCGTAGAATGAGAAAAAACCCCAAATCCCAGCAAAGAAGGCGGTGATAGCTGCAAGGAATGGTGTGGGCTTTCTTCAAATCTCTAATAGGATGTATTCTGGCGTCCTCACTTTGCATCCTCTCTCTCTCTCTCTCTCTCTCTCTCAGGAAGGATTGACTGCTTTGATTGAAAGCCGGTTGTAGGACTTCTTCTCACTGTGGAACCCACCGGGATTCATCCAAAACCAAATCTATCTCTTCTTCAAGATATTTTTGTTCCAACTTACAAGCTTCTAGAGATTGATTTTGGATATTATTGTGCTGGCTGCGGGCTCTTTAGGGATAACTTTTTTTTATTTTGATGACAGACATTTCTATATGTTAAAGATTTTTGAAAATTATCAAGTAGTAATATTTATCTATTTTAGAGATTTGTGGGTTTATTTTGGTCTATGGATGTAGGATATTTGGTTGTGGATGTTATTTTCGATTTGTTAATGAATCTATTTTAAATTGGGTTGTGCA

>ONT.17058.1 LG12 16792980-16806105

ACTCAAATCCTTTGTGCCCTAATGTCCCCGCTCTCGCTCTAATTTCCTTCTCTGGCTCTCTCCCTCTCTCGCTCACCGCCCATCACCTTCATCTCTCCTTCCCACCTCTCTCTCCCCTGAACACGTACGGGCACACCATCCTTCTCAAATCTTCACTCCTCCCACCATCCATGCACGTAGCAGCACCGCTCTCACGCCATCCCCCTGCAAGCCTCCATCTCTTGACCTCCTTTATCTCTCTGGCCCTCTCCATCTCTCTCACGCCACCTCCTTGTGCATCCTACATCGTCCCCTCTCGCCGTCTTTTCCAAGTAACGATTCTGCTGGTGGGGTTTAAGGGATCAAGAAAGAGTTCTCTCATAAATCTTGTGTATAGTGTTCATGTTCTCATCCCTTTCGCCCAAACCTCTAGCCCTCTCACCGGACCTAGCTTTCTCCATCACCAAGCCCACATCACCACTCGCTCAGTGATGGAACCGTCACCACCGAGCCATCATCTTGTTCCTCCAAGTTGACTAGAGGTATCAAAATGGACAAAAGATGGTACAATATCAGATAGGAAACATATAGCCAAGATGAACAATAGCCAGAAATGGAAGAGGTTGATGATGGTTATGGCTTTCATACTTTTGGGGGATTTGCTTGGGTGCTTTTTCTGGTTTTGGTTGGTGTAAGGTGGGCTCTGTTTTAATTCACTTTCTTTTCTTGGATTCTTGATCCCCTTTTGTTTTGCTTCTTTTCATTATTTACTGAAATCTGAATTATTAGTTCCTTTTTGTTTTGCAAAAATTTTTATGTGTTACAGTTTTATGATGTGGTCAGGTTATAATGAGATGGGATGAATTGAATGTTAGGTTTATATTGTAAGGTTTCTTGTATTGTGGAATGGAATGTTTATGTATGGCACTAAAATTGTTACATTTGTAAGAATTTTTATTTTTTTTGGTAAATGTGACATTTGTTGTGGCTTTATATTGTCTAAGTTTTATATTCAAACAATAATTCAACATATTCATCTA

>ONT.4735.2 LG02 77278728-77282105

GTCAGACATTTAGGTGGAGATCCTTTCTGCAGTCGGCTCCTCCATTAGCTCTTGAATATTGGCTTCAAATACTTCCGACCTCATAATTGACACCTTCTGATGGGTCTACTTCACAAATATCCCCACCAACCACCATTCCTCTCTCTTCTCTTGCACGTCCAGCGGCTGCTGGAACCCATCTGCTTCTCTTTTCTTCTTTCTTTCCCTTTCATGGTGGATTTTATCTCCATAGAGGCTCCAGTTGCAATTTTCGACTCCACCAATCGAGTCTACGTCATCTTCTTACGTCGTTTTGGCGAGAATCCATTGTTGAGCATCTTAAAGGTGGTTTTGTGGAGGATTTTTGGGGATTTTTCTTGAGGTGATTTCATCCAGATAAGGGGTGATTAGAAGGTCATTTTGGTGGCTAAGAGGCTAAGGCAAGATTTCCCCGACCTTTGACCTAGAGTGGTATCCAAGTGATTAATGTGCTGAGGTTATCATATAGACTCCGCTCCTTGCACATGAGATGTTATCACATGGGAGTCTTTATTTTGGGTTTTGTATCACATCGCATAGGCCTCTGTTGGTCTTTATTTTGGGAATGTATTTAATGCATCTTTTGTAACTCTCTAACTTGAATTATGGGAATATAATCTCCTGTTTACAGGTCTA

>ONT.4106.5 LG02 1698176-1705497

CTCCTAAGTCCTAACCGGAAACCTTTCTCGCTGGCTGCCCCCTCTCATCGATCCACCGCCGGACCTCGAGCCATCGATCGCCGAAGCCGTCTTGTCTCTCAGCCCTAAGGATATCTTCTCACACACCATCAAAGGCCTCAGTATTGAGGTACCGTAAGAGTGAAGGCACAAGAATGGCTTTTGTGATGAGGTGAAGTCTTTGAAGAGTCTTCATGAGTTCCAGAAACAGTTAGGCCAACCAGATGGATTTCCATGGTACACATAGAAACCATGGTGTTGGCCAATCAAGTGAGACATTAACATATGTCAGAAAAGAGAAATCTGGAACACTCCTTAGTGAATACATTGCTTTTGAGGGCAATTCCTGGGCTGAATGAATTTGGCAAGGCTTTATTAGGTGAATGAAGAACTTAATTAAACGGAGATGGAATGCCTTTTGCTAAGCGGATATACTAATTATGTTAAGTTAATTTTGGCTAGTTAGGGATTTCCGGAAATTGTTTACAATGTAGCTTGCCTTTTTTTGGATGCTTTTGTGAAGGTTTTGGATTATGTCGTGACGCTTTGGTTACGAATTCGAAGGTTAATGTAAATAGTTTGGA

>ONT.7073.1 LG04 87641744-87643483

ACAAGGCCAGCTTCCAATTTCTCAATTCTAGCATCAGGACCACTTCAATTGAAGTAACTTCGCCAACTTTGCCATGGACATTCAATTTAGTCTTGGGTGCTCTAAGAAGTCACCACCGCCCCAGCCTAATAACAAGACCAAAAATGCCTAGACTGGTGGGGGCAAGCATACAACCCACAAGTCCAAGGAGGGAACAAATCAGACGGAGGTGGCAAGATCAATATGGGACCTGAAATCACTGATGGTGGAAACTTCACAATTAACTGAAAAATAGCCCCTACTCAATCAGAGTGCTGAAGCTACCCTCAGCGCTTGCTGCTACTTATGTCTTATGTAATATGAAGCTCTATCTACAGTCATCCAAGTCAGGCTCTCCAGATGTGATTATACAATCAGTAATGTTAGCCTGTTTTTACAAATGAAAATAAGAAGATGGTATATTTTGGGATCTC

>ONT.10662.5 LG06 33950225-33960475

GGCTGCTGGAACTGTAGGCGGAGGGGTTTCAGTCGAGAGGGAGAGAAATGAGGAAGAAGGAGGCTTGGGGTCTTATATACTGAGGGTCTCTTCCCTACTCATACCCTTCTTCCCACTACATCATTCTGTTTGTTAGAGGGTTTTCTCGGTCACGCTCACCCTTCTTCTCAGACCCCGGATATAAGTTGGAAAGGTATACAGCTAATGACTCACGTATTATGGTAGATGTTGTATATAATCCAAAGTGGAACTTTCCTCATGCTCTGCCAGGTAAATATTATGTAATTGACACAGGGTATCCTAACACACAAGGTTTCCTTTGACCTTATAACCACCGTCGCTCTCATATTCAAGACTTCCGCCATGATGCTGGATCTAGAGATAATGCGGAGCTCTTTAATTTCTATCATTCCTCACTAAAGAATGTAATAGAAGTATAGAACGTTGCTTCAGGAAGCTAAAGGCGATCTTCCCAATATTGAAGGAAATGACTCCTTGCCCATTTCCTACTCAAATGTATATAATCGTCACTACAATGACAATGTATAACTTCATCCACCAGTAAGCGATAATTGATACCTTGTTCAATTATTCATAT

>ONT.8066.1 LG05 11946509-11947613

ACCCATTTCTCCACATGGGGTGGTTGCAGTCCCTCTTCTCCCCATTGAAGAAGGTGTGGGATCGCCTAAGTTCACCACACAAAAAGAGAAGAGGGATTTACATTCTCTATGAAGACGTGAAGTCTTGCCAATATGAGGATGTGCACGTGCTATGGTCCATCTTAGTGGAGTCAAATACACCAACATTGAAAACTAAAGGATAAGGGTTCCCTTCCCTGTGAACCCAAACGAATGTTTCCATGTACATAGATATTTATACACATATAGGCCTTTTTTCTTCATGTACCTAGAGGCCCTTTTCTCATCTGTTCATGATCCATGAGCATTGTAACCAGATACAATACCAGATGACTTTCTCATGCGGAATTTGCGAGTGCTTCTTAATGTGTTTGTTTAATGTTTTTTTCTTTTTCCTTGCTCTTCTTAGTCTTGCAATGTTTACATGTCTGAGTTAGATTGCAGCATTTGGTATCTCCACTGTGCATGGTCCAATATATGAATTGAGACCGTTGATCAGGTCAGGTTGGCTCCACTTTGGGAATTTCACGCGGTTTGGGTGGTGATGATCTTCCAGAAAATGTGCACTTCATTGTAGAGGGTGGGCTATTGTCTTTGTTTTGTTTCAACCATGTCAGAAGGCCTTGGGATGGTCCAGATGACCCTGCATGTGGATTTTTCTGCACCACTGGAGTTGACAAAGGGGCCCATTGGATGGACGGTTCTGGCCATGATCGGTGGACCCATTTACAATGGAGGGTTTGGCAGTTGGTTGATGAGCATGGCATACTAACTGCCATTTTCCCTATTTTCTATTTTCTTAATCCATACGGTGGGCGTGTTGTATTGTCTATTTCGGCAGTTGGTAGGCCGTTGGAATTTCACTTCAGTGACTTTGGCTTCTCTCTTA

>ONT.16140.3 LG11 20997467-21003056

ACACCCTTGCGCCCTTTCACCCTCTTTCCCTCTCTCCCACTACTTACCCTATCTATTTTCCCCCTTTCTCCCTCTCTCCCCCCTACGCACATATTTGATCGGCAGCGGCACCTCTCCCTCTCTCCCACTCGGTCTCTCTCCCTCTCCTCAACACCTGCAGGGGATGAATGAATGCCCCTGCAGCACTTGCTTTTGAAAACGGCATTTGTTGCTACTATTCCTACTCCCATTTAAATACACTTTTACCTTCTCTTCTCCATCTGGGTTACAGGCGTCAAAACATCACTCGATGATGTTATTACTGCATATGAATCTGACAGCTCGGTGGCGCAAGGTCTTTGGTCGTGATAGCTAAGGATGCATACGTGGCTTAGGCACTGTTTCAAAAACGGCCATAAAACATTCAGCTCCTTATAAAAGAGCCTTGGAGGAGGAGCATGAAAGCAATACACATCTTCAAACTGAGGTGAAAAGGTTGAAGGAGGATTAGATGGGCATACGGAGGGAAATTGATGAATTGAAACAAACTCTTACCCAAGTTGGGACACAAGTGTGACCTTCAACTTATCATGATAGTTCATCTCAGGCTCAGGATAATTCCTCATGTCAAGTGGCATGTACAAATCAACCGAACATTAATACACAGCAGGGTACGTGCAGGTTGTTGCATTACGTGCGAAAAGATATTATTGTTGCCCTTGGACGTGTTCTTGGTTTGTCAACTTAGGAGGAAGGATGTTATAGGATTGTTGTAGATGAGATCTTAAAGTTTAACATAGAGTTGTTGGGTGGAGAAAAGATGTTTGGGGTTCTCACCGTGGGTGACATCATTTCATGGCCCACATATAGAACCGTGTTTGGTTAGTTACAAAGCCCACATGATTTACTATTTAGATGACTTTATTTGTAGTATTTATTATGATGTGTGTGATCATGGTATTAGTAATACCGAACGTGAACGTGGTTTTTGTTAATTTTTTACCATTTACTGTAGTTATACTTTGATGGTTTAAATTTTAAGTGGATGGATGATTTATTAGCT

>ONT.8256.1 LG05 21903919-21907579

GGGCCTCACGTAATAAACTCATCCCAAAACCCTAAACCCCAATACAGAACCACATTTTCCAAAGCTACCGCAAAATCCCTAACCCTAATCTCTCTCTCTCCCTCTCCCTCTCCCTCTCTCTCTCGTGGTGTTCTCATGGCGAAACCTGCAATCTCCATGCAGAAAGTCAAGGAATTCTGCTATTCTCAGTTCCAAGACGAGGAGAAATTCTCCGTCAACATGAAGCTGCTGCGTGGAGTCGGACTGTTTGCTGGCTCCATATTTCTAATGCGCAATTTTGGTGAACTCATGGCCATTTGAAGATTGTTAAGGCATTATGTACCTCACAGACAGTATTTTCTATTTTGTAGTTCATTTTGTTGTTAAATGCTAAGAGATTTTTAGCTTTAACAAAGCTGCTTAACTGAGTTTACTGGATTACTGCAAAACTTTTGGGTATTTTTGTAGAATTCCCTGTCCTTTTCTTGATTTTCTAATGCAACCTCTCAAATATCAGAAATTAGAATGCGGTTGCTC

>ONT.11718.2 LG07 3579779-3581866

ATTCGTTGCAGGTTGATGGCTGGGAGAGTATGAGTAGAGAAAATTTTCTCTCTTCTTCCTTTTCAACTTCTCCCTCTTTCATCCATTACTTTAACTTTATACAAGTGAAAATCTTCTTCTTCATCATTTCCTTCATCTTCTCCACCCTTGCTTCAAGAGACCATTTGAGCTCCAACTCTTAATTTTGGGTTCTTTGTCAAGACACAGATACAACCCCCGGTCCTGAAGAGGTCCAGGAGGGAGAGAAGAGCACCCAGGAGATATATTCAGGACTAGAAGAGTGGCCAAAACCAAGGCCCGTCTAAAATTCATCTTCTCGGAAGTGCGAGCTAACCTTGTAGATATCATCGTTCGTATGATTATTCTCTTTTCATATAGTCATACGGAATTCTTTTGGAGATACTTTTGGTAGAGATTGTATAGGGTTCTTTCGTTTACACATCTCTTGTAACTTAATATATATGTTTGTTTACTTTTGTATATCAAACTTTAAATGTTTAATTCGTACTTGATGGAATTTGATACCTATATCAATGAATTATGACTTCC

>ONT.8842.2 LG05 104974517-104986139

GCTTCTTCTCCCAAGTCCCAAACAGCCAAACCCATCCCAACGGCAAACCTTCATCTCCCATCTCTTCCATCTCGCATCCGTGGCTTCTTCTTCGCTAGGCATTTCGTTCCTGAGAGATCCAACGGGTTCTCCAATAGAATTGCCCTTCCCTAACCCAATAGATTCGAAACCAACCCCATGAAATACATAATCATCGCCAGCCCCAAAACCCAAGGCATCAATATGAATCCGAACACGAACGTCACAGATCCGCACAACATAACATCAGAGAGAGCGAGATCCCCAGAAGAAGAAATGCAAAACCCAAAGGAGAAAACTCCGCCCGCCTCGCCGGCTGAGGGGAGGCGACGGTGATGGAAAGAGGTGGAGGGCTAAGGATGTTGAGGAGGCTGTAGAGCTCGTAGAGAACCCTAGATTGCTGTTTTTGCTTGAATACGTAGCCTCCTTTGAAATTTCTGGGGCTAGGATTTCTATCTCAACTTTCAAAATTTTTTTTTTTTTGCTTGATGTGTATGTATGGTTAGAGAAAAACTTAGCTTCTAGGTGGAGAAGGGAGAAATTGCTAGAGATTTGAGAAGCCCATTTCCTTCGTCTCTTGCTCGACCCATCATCAACAGAACCCAAAAGCAGAGGAAGAATTCATAGGTTTTTTTCTTTTTTTTTCCAAGTAATGTACATGGACGGATTGAACGGATTAATCCGATCCGATTCGGTCATCCACGGATCGGATTGGATCGGATCTTAATGGCCATGGATTGGTGACGGATTTCAAATCCGTCAATCCATGGGTAACGGATTGGTTACAGATTTAGCTCAATCCGCACCAATCCGATCCGTGTACAATCCTACTGATTAGGACTCTGCCTTGCTGATTCGTTTCCATTTCATTCTTAGAGTCCCATTGGAACTCCAAAACCAAAAGTCTTGTGCACGAACCCCCTTGCAGCCATAATGATTATGCTTGAGCTATCTAATTCTCTCAAGCATCAAGCATATGTCTAGAGAGATTTTGTGGGCTTATTTTGCAAGGGAGACTGGGCCGAAGTTTTCTTCTTCTTTCAAGCAACTTGAAGGGGCATTCAAGGCGAGTTTCAAGCTAGCACGTGAAAGGTGAATGATGGGTCGGCGGCAAGTTCTTCATAGTCCTTTAGGTTGAGTTTCAATTGTGTCAAAGGTCTGGTGAGTTCACTTGTAATCACTTTGCTTTTTGGTGGTCTCTACTACCCGTGGTTTTTTCCTAAGTAGGGTTTTCCATTTTA

>ONT.17923.1 LG12 49406136-49411719

ACAAAATTTAAGAGCAGCAGAAGCTTTTTTCTCTTTCGCTAAGCCTCCCCAATAAATGGCATTGCTGTCCGCTTCGATTGCTTCCCTTTCTCGCTCAAGAACGGCCATCGTTGCACTCCAAAACGATTGTGACACCAACTCTTTTGATGGATGAATCATTTCCATGGCACCAACTCCATTGAAATACCAAACCCACTGCTTATTTGGTGGTCCTATAGGGTAGCCCAGCAAGTTTGGTGAAACTCGAACCGGTGCATGATCAACATGTTGCGAAGAAGTATTTGCTCCCAAGCATCTGGAGTTGGAAAACACACTTGGTATGATGGTAAAACACACTTGAGGCATTGTTGGCAACACTTCTCTTCAAATTGTAAATAGTATCTTTTTTTATTTTCAGTTAAAGAGACTGTAAATAATATCATGTGTAAAAGAATGTAAGGAAGAGGACCTGTTGATAACAACAATTGTATTTGGTGTTTTTTTTTTTAAATATAGCGAATTGTGTTCATTTGCTTTACTGTTG

>ONT.17610.1 LG12 13289388-13320313

ATTTCTTGCACAACTAAAGCCCATGTTCTCTTCTTTTCTTTTTCTCCCTTCTTCCTTCTCCATTTCTCCACCAAACCTTATTGCCAAAGGCCGGATCTCCACCATCTTGAGCTTCTCTTCGTTGACATCATCATCATCTTCCCCAACTCGCCCTCCCTTGAGCGTGGTGATGACCACAACCATATGGCCAGCAGCAGTGATTCGTCTTCCTCAAGCTTCCAATTCATACAAGGTTACGACACCACTACCGATAAGTAGGAAATTGAGAAGTTGTTAGAGACTCCGCAAGGCTTCAGGGAATGGAGTGTAGATGATGTGATATGTGCATCGGGATTGACACCAAGAGACACGTTATCACTTGTTGGGGATAGACATTTTTGGGTTTTGTTACCATTGTATTGGCCTTTTACTTTGAGATGTAATTAATGCACTTTTGGTATACACAACCTTAACATTTGGGGAATGAAATACCCTATTTTCTTA

>ONT.12996.1 LG08 51824302-51830170

GTCGCAGGCTTCCTCTTCACAAATCCCATTTCGCTAGTTTTCCATGCCATTTTCTTTCAACGGTCCGGCTTCTTACTGCAGGTGTCTCTGCCCCCAACTGAATTTTGGGCCACAAAACAGTCTCATGGTGGGCCCCACCCGATGTCTGGATGGCCACCAAATCACTTCATGGTCACCAAAGCATCCGGAGATTGTTATCCACTTGTTTGAAGTGGTGATGATGTTTTGCTTTCACCACCTGAGGAAGAGCCAAAGAATGGTAGACCACAAAAAAACTTTTTCGATATCTGTGTGCCTAAAATGCATAGGTATAACCATTCATTCTGCCAAATGACAAAAGTTATGATTGACCTTCTTGAAGGTTAATCCATGTTGCTCAAGACATATATTAGAACACAGAAATGGGCTATAACTCATTATAGCTTCTTGGAATTACTTACTATAGCCATATATATGTGTACAATATTGTGAGATATTAAGATATATTTTACTTAATTTGTTAG

>ONT.14904.1 LG10 38424002-38429098

CCCTTTCAATTCCCATCTACTCTAGCGATTTCAGAAGCTCTTCTTGGCGGTTCGACATGCTCATCTCACATCTCCTTCATATCTCTACCAAATCACATCAAACAACTCATGATCTTCTCCTTTCTTCTTTTTCTCTCTTCATGGAGCAAAGGAACGTGCTCCAACCCTTCTTCTATTCATTGTTTCCAACAAAGGACAAACCCTTTTATTGTGCTTTCCTAGTAGTTCGAGTACTTGGGGGTAAACTTTCACTTGCATCTTCTAGAGGGTCTTTTAGGGTATTTCATCCATGAATTTTAGTTTTTGGGGATCTTCGCGGGAGGAGGTTCCAATGAGTTTCGAGAGTGATTTGGAGTGTTTTGGTAGTGCTTGGAAGCCAAGGCAAGAGATTCCGGCGTTCCAAAATGATACACTAGAGATCAGGGAATGGACAATCAATGATGAGATGTGATGCATTGGAGGCCCAGAGATCCTCATTTCAATCTTTTGGATTTTATTTATTTTATTTATTTTTTGAATCTATCTTTTGTTATTTTTGTTATTAGTCGTTGTTTTTGCAAGCCATTATTCAGGGACGTAATTAAACTTATTTTTGTTGTAAATGCCCAACTTTAATGTAAATGAATGGAAATATATTTCTATCTG

>ONT.3125.1 LG02 18307958-18315466

GAAGGTGAGTTGGAAGTGTTTTCTCTTTATTCCGCCAGGGGTTTTAGTCATTCTCCAACTCATTTCCTGTTTCCTTTTCCCATTCTCTCTATACGATTTCTTTCCGGTCTTTGCAACCCTAGCTAGATTCATACATTTTCGTTTTCTACTTTGAGGATTCACATTTTCTTTGGCATCAATAAAGCTGCATGTGATCGCATTCCAAAAACCCCCACGAGATTCAACTTCTATCAGTCCGGAACTGAGAATTCCCTCTTCTGAATCTGCAGTCGACTACACGCTCGTTCGTTTCTTCTATTCAATTTAGGGTTTGAGATTTCTTTTCCCTCTCTCTCTTAAAACTAGGGTTTTGGAGCTTTTGCTTTCTTCTTTCACTAGTCACTACACTTGATATGAATTTCATTTGTCAACTCTTGCTGCAATCATGCATGCCAATCTCTGGGTCACCTATTTAGGTCACAGTAAATTGGGCTTTCCTCCCACAAATTGGCATTAGATAGTGAATGGATCTCCTACCAGAGTTTCATTTTGTAAAGCGCTCCTTGATAAATAACTGCATTTTGTCTGAACAACAGTACAGAAGGAATAAGGCTTAACATGCTCGTGGGAAATGATAAGACCTACATCCTTATGGTCTGGCAACTCATTTCCTATTTCCTGAGTGGTTGCCAGGAGATCCTGTTCGTACTGGAGTTGGATGCTATGATGGGCTCCACTGGAAATTAGAATAAATTTCCTCTCTATCAGCATGCTGGGAGTTTCTTGTCGGATACAACTAAATTGCCCCCATATCAGGCGTTACTTTGTCTGGGCAGAGCCTGTCCTAAGGTTGGTTTGTTTATGCATGTTGTTGCTGTGTTTGAGAAACTTGCCAAATGATATACGTGGAGCTCATTTATTTTTCTTTTTTAATTTAATTTTTTAGTATGTTTGAGGCTGATGTCCTTTCTAGTATGTGCTCATGGTTTCCTCATGTAGACTGCTTGTGTTCATTTCCAAGATTTCTTCAATTGCTGAC

>ONT.7647.1 LG04 74415910-74425880

TCTTCTTTCGAGTCCCTAGAATGAAAACTTCCCAACATACAATACACAAGGGAAAGCTGAGAGAAACGGTGATAACCTGAATTGAGGCATGGAATCCCAGCAAACTGATGGTCTATTCATTCAAGGTGATCCAGTATCCAGATTGCGCTCAAAATATTCAAGATTTTCAAGTTAAGTATTTGAGATTTTGCATTCAAGGCGTTTGAGATCTTGATTAGAAATTGAGAGCCCTCCATGCCAGTCATTCAAGCATCTGATGAAAGTTCCCGATCACCTTACAGCTAAATAGTGACCACAGGTACCTTCGGGATTGACAGAGTTCTAACTTCTAGAAATAATTAGAGGAACATCCTCGTTAATTTTTCAAACTTGCAAATGAGGCTGATGCTGTTGAAGGGGAGGTGCAAGTTTTGTTGAAAGGTTTGTGAATTTTCTCCAACCACTTCTCCTGTCAGTAGAAGAGTACTCAGCTAAGGCCATAAATTATGAATTCTTGAATTCCAAGGAACCTGGGGTGCTTGCTTTCTTAATGTAAATATGCTTCTACTAGATGTTTACATGTTCATTTGCTCATGAAATTAAAAAGGCTGATGAGGTCGCTCTTTTCTTCATTTCA

>ONT.7803.2 LG04 88135264-88136167

AGGCAGTTTCTAACTTCTCAATTACAACTTCAAGACCACTTCAACTTAAGCTGATTTTCCATGGACATTCAATTTAGTCTTGGGAGCTCTGATAAGTCACCACCACCCCAGCCTAATAACACGACCAAAAATGCCCAGACTGGTGGGGGCAAGTACAACCCACAAGTCCAAGGGGGGAACAAAGCAGATGGAGGTGGCAACATCAATATGGGACCTCAAATATCTGATGGTGGTAACTTTACTATTAATTGAAAAAATAGCTTCCCCCTCTAAATGAGGATGTTGAAGCTACTACCATCCGTGCTCAAGTCTTTAAGCTTTGTCTACTTTCAACCAAGTCAGGGTCCTCTAGTAGTTACTGAAAGCGCCTCCGGCCTCCCAAAGTCATAAAAAATACTGGAAGTAATGTTAGCCTCTGTTTACACAAAATAAAAATAAAAATAAAAAAATAGTTATCTGCATCGTTTCTGTA

>ONT.10564.1 LG06 6396453-6400644

CTCTTCCCCTTCCCATCTCTCCGAAGGAGAAAAAAATTCCAAGGAGCTCCTCTCCCTCTCCTGCTTCCATCTAGTGTTGCTGGTTCTTCCTGAAATGAAAAGACTTCATGATAAATCAAAGATATTAGCCCAGATTGTGCACTGCTGGACATCAAGGAACCCGCTGAAGTCCAAGATCAAACCCACCATTGGATGGCCCTCCTTATGCATACTGCGTTATGATCATGAGAAAATCAAAGCCTCCTGAATCCTGATGTTTTGTATTTCCTCTTATTGTTAATGTTTTTGTAAAGGGCTGGAGTTTCTCCCTATTTGGACAACGTTGTTGTTGTTTCATTATATATATTTTATAAAGAGATGTAATCGTATGATTGGAAGGGAAGTTTATCCTTCAGTTGTATGTTTGAACTTTTAATGTGGTTTCCTGTGGAGGGGTTTGAATTTGATGCCTAGGCTT

>ONT.2256.1 LG01 31042535-31044732

AAAGCTTTCATCTTTCCCGCTCGTCTCTTCCTCGTCTCTCGTACTCTCTGCTGTCTCTCAATCGCCAATCTCCTTGCAGGTGACGGCTCTCATCCCTTTCTCCTTCCAGGCGAGGCCTTGTAAAGGTCAACTTCTAATGACAAGACATTGAACAATTGCAGCTCTATTTTGTAGGCTTTAAATGAAGAGTGGTTGACATGGTTTCGCCGTTGCATGTGGTTGTGATTGGCATGAGAAGTTGAATGTTGGTTTTGGATGCTACCCATTGTATGGAATTAACTGCAACTGGATTTTGTAATGTTGGTGGTTGTTAGAGGTTGCTTAGTTCTATCCATTTTTTTTTTATGGGATTTGATGTTGGAGGTTGTTATTAATGTAATTTGCTTAGTTCTATTGGATTTTGGGCCCGACTCCAATGTCTCTAAGTTATTTGATTTTATGTTGGATGCTGGAAATTGGTGTTAGATGTTAGGATTTTGCAAATGTGCTTCGAAGCTGTTGAAATATTGCCCTTTTACAAATGGATATTCCAGTATTATAAATGAATGTATAGAATGTTGGTTGTGTCCATGTATTTTGGAATTGCAAA

>ONT.12836.2 LG08 6795078-6799231

GGGTACAAACTACATTGGTCTTGCACGAGACGGTCCATATGGATACCCATCTTGGGATTATGCACCGAGATGATTTGATGAGGTACCATGGACCCCATCTCATCTAATGACCGGGTAGTGAACCCCACTCTCCTCCATCTCCCAGGCGACCTCATCCACCGCTCCCAGGCGACTTCATCCAGCGCAGAACCACTCACCAGAGTCTCACCCTCTCCACGCCTGTCTCTCCATTACCGACCCATCTCACACCCTCCCCTGCGCATCACACAGCTCGGACTCTCTATCCCCATCACCTGAGGCCGATCCCTGCAACCCATCCCTCACCTCACCTCTGCGTCTCTCTCGTCTCCCTCCGCACCTCTCTCTGTGTGCGTCTCCCAGGTTATGAAGACGAGAGGTTGCTGCAAATGTTGCTGAAAGAAGCCGTCCATGTGGTCAGCTGTGTGGCTATGTGGCCAATACAGAATGGGGCAAAGAGGCATTTTACAAAGTTATTGGAAGTTGGGCTTCAAAGAAGTTTATGACAGGATGGTGTGCATCATTTCTCAATTTGTGTTTGATCTTTATCTTTCACTCAGTGTTAAAACTGATCTTTTCTTACTGTCATAGATATTGTTTATCTATTGTCCCTTTCCGAAGTTTTTTCCTTGATTATGATATTTTGTGTTTTCTCTTTT

>ONT.12805.3 LG08 3431838-3446805

GAGAGGCAGAAGCACAGAGGTGTTGGTCTTCGTGCACCTACCACTCAAAATAATGCGAGGTGCCTGGGAAACTATCAACGGAGCTTGAGCTCTACTACAGTCAGGCGCTTTTTACTTTCACAAGTAATTGGGCTTTTTTCTGCCCAAACAGGGTACCCTACCCATGTGAGCAACTGAAAAGATTCATTCAAATTTTTACTTCAATCATAAGCAGAGGTTAAAATAAGTCTTGCAAACCTTTTTCAATTACTTCCTTCTAGCTCTAGCTTGACTGGCTCTCATATACACCACAACTCATATCCGATTCATACTATCTCTCACAAACGGTTTGGGAAAACAGCAATCCGAATCAAAGTTCTGAGTATCTCCTCTTTGCTAAATTCTGATTAGGTGAATTGTGGCATTTGAAGTTCAATAAAGTATTAAAGGTGTGGATTGCTGGAGTTTGATAAAGAGTTATAGGGATATTGGCAACTTCTTAATGGTGTGAATAGTTGGAGTCAGATAAAGAATAGTAGGGATCTTTGCACCTTCTTATTGGTCTGGGCAATTCATGGAGTTTAAAAACAAACAAAACATTCCTTCATGATGTTGTTTGAACTCATGGTTGTGTTTTTTGAGTCGCTTTATTTGGCTTTGGTTTAACTATGAATTTACTGGTGTAGTTGTACTGTGTGGATCTTGCTTGTCATCTTTGGTTTGTTCTTATTCACTTGTTTGGAACTAAAATGATTCTCTATTTTTACCTTCTGTATGTGAGGAAATTATATAGACCATGGATGTAATCCTAATGACTTGATTATCTTCAAGTGGGGCCCATGATTTTGTCA

>ONT.3605.1 LG02 99510402-99514868

AGTTGAGAGAGGAGAATGAAATACCGATCTATTAAGGCACGGTTGAAAAGGTAAAGAAGGGACATGCCCCACTAAAAATATCCCTTCCTTGTTCATTACCTGAGGTCACAGCCTTAGGAATTTCCGAACCTCGGGCCCTTGAAATAGCCCAGCGGATTGCTCTAGGAATAGTACTGGACCATCAAGCCGACTCGTTTGCCGACAAGTCCAACATGGCCTCTGAGGCATGCAGCACAGTCTCCGAAGGAATAAAGAAAAGTTAAAGACATCATTTTCTACTTTCAGCTCCTTTCGGCTAAGGCAAGATAATTGTCTTCTTAAGAACAGGGAAAAGGGACAAAGTACCCGAGGCTGTCCCTTGCGGAGTTGTAAAATATTTTATCTTTTATTTCATTTTGGTTTTGAATGGTTTGGGGTCCGGCAGTTTGTTGCCTTTATAAGGACTATTGTCGATTAAGTAAGTAGAGGAGATTGGTGGTGGAGAAACTTGTAGTAGAAGCTTGTCTCTCCTTAAGGTATGATTGACTAAGGCCCTTTCCAGGTAGCTGGAGCGGCGTCTTTCTAAGATGTAATAGTCTATTTGTACTTTTATGTGGTTTAATAATAAGAAGTTTGTTATTTTCTGTTTC

>ONT.10323.3 LG06 63894151-63895884

TTCATCCGTTTTCTTCTTCTGTCTTTCGTTTTCTTCTTCTGTCTTTCACTTCTTCTTAATCCAAGGTGAGTTAAGTGAAGAGGAGTCCATTTCTCCACCTTTCTCATCATCTTCTTAAAGAGATATTGAGATCAAAGCTTCAAGTCAAGGGATTTTCACTCAATCCAAGCTAAGAAAGGGGTTTTTGTTTCATTTCATCTTCACTTAGGCTCAGTTGCCCGTGCCAGTACCTCCGAGACCGTCCCGTCAGCGGAGGAGGCCCATTCGCTACCTCCAAGAGGTCTAGCCGAATGGACATGATTAAGAGCGGACACGTGGAAATCTTTTGAGAACTTTTAGAAATGATTTTATAGATTCATTTTGGTATTGTACGTATGTAAATGATGTCCCCTGCAATGTATTTTAAATATGCAGTGGTTGATAATAGGAAAACTGAAATGATGTAAAGTAACTTTACTGTTCATAGTAAAGTGTATGATTCGTTTTCTTAA

>ONT.6351.1 LG03 61065836-61067438

AGCTCTGCCTCTCTCTCTCATCTCTTTCTGACTCTCGACTCTCTTTCGCCTCTCTCTCTCTCGGACGGCGACGCCCAAAAAAACCCATCTCTCTCTGTCTCTCGCCTGCTCTCTCTGACCACGACGACGATCGGACGACGACTCTGAGATCTCTCTCTCTCACGATCTGTCTGTCGCCTGATCTCTCTCTCTGCGAAGCTCTCTCCCTCGCTTCTGTGTCTCCCTCGCCTGATCTCTCTCTCTCTCAGCTCAGGTCTCTCCCTCGCTGTTGTCTCTCCCCTATTGCTGCTCATCTCTCTCTCCCTCTTTGCGACGAAGCCCTGCTGCTGCTGCGCACATCTCCCTCTCTCTCGCCTGCGTCCGTCCGATTCACACGCCTGCAGCTGAACTTTTGAAATCTCTTGTGTAGTGTAGAGAACAACTATGTGCCTTTTCATGAAAATGAGGCATCAAAGAGATCTTCTAAGGATGAGGCACTAACACAATTAGTAGGATGTATTGATGCTCATGTTGAATCATCTAATACCTGAAGGTGTTTTGAAATGATATATTTTTGGGATATTTGGTTCTTCATAGTTGTATAGTTTTTATAAGAATAGAGGGGGGAAAGTTGGATTGGGCATATTTGGGATGGAGCATTTTTCCCTCTGTGATAAACTTTCATTGTAATTTGGTATGTGTAATTCTCATTTTGAGTTTGTGAAGATTTGTGAAGATGTCAGGTGAAATGAAATGCTCCAGGTTATTATTATTGTTGTGTTGTTTCTTGTTCA

>ONT.11930.2 LG07 53985794-53997983

GCGTGCGCAGAAGGTGAAAAGGGAATTTTCAAACCTGTTCGTTGTTGTTTGAAGATGAAGGGGAGATGGAAGTTGAAAAGGCGCATGTGACTTGCTGGCGACGCTGGCAGGCGACGAGTTGCTGATGACGATGGTGAAAGTGCTGCGGAGCGGGCTGGAGGTCCAGAAAAAGACCATCCCAGAATTCCATCAAGAACCATGCAATAGTTGCTGAACATTGGATGATGATGAAGAAATCATTTTGGGGATTGATGAACAAGACATGGAAGAGAAAAATCAACAACCATTATGTGAAAATGATGTTGGCATGTCATCCTGCGTGGAAAGGAGAAGGAAGGAGCTCAATTTGAACAAGTATTTGATTTTGATGATGATTATTGAATAGTGGACTTGAATTTGGATGATAATTTTTTTAAAGTACTTGTTAGACTTTGTTAACATAATTTTTCACTTGTTACTTGTTAGTGGACTTTGTTAATATTATGTGATTTTGTGTTGTGTTTTCAATTCATGTATTACGATGTAAATACTCGATATTGTGTTTATGGATAGAACTTAGAAGTTATGACTTAAAA

>ONT.3296.6 LG02 31255608-31256145

AGACAATAGAAAAAACCACTTCTTTTCTGGTTCATCTCGGACGAGCAGAGGGAGGGGAGGGGGAGAGAGAGAGAGAGAGAGAGAGAGAGTGATGGCGAACCAGCTGGTGGAGAACCTGGTGGAGTCGATAAAATCAAAGGTCCGATCTCTGAAGAGATCGAGGAAGAAGAAGCCATACGTGATGATGGACAAAAGCTCAAGCGTCAAGGTCGAGATCCGAAGCCGAAAGGCCAGAAACCTCATCGACAAAACCCTCAAAGTCGTATATATATATATATATACACACACATATATATCTATATCTATATCTGCATATCTCTCCCTTGATTTAGATACATATGCATCTTTACCTTTCTTTTATGGATTTTGCTGTCTCTACGTCTGTGATAGATTTACAGCTTGCACTCTGATGTACTAATTTTGGTGAAAATGGAAGGAAAGATGAGCATTTTATGTTTTTT

>ONT.4722.2 LG02 66221775-66227282

ACAATGAGATTCGACGATACTTTAAATGTATGGAGTACTCACGCCATCCCTCTCTGCTATCTCCCTGCTCACGCATCCCTTCCCCCCCCCCCAAAAGAAAAAAAATCTATCATCTAGGGTTTTCTCTCGAGCTCTTCGTCTTTCGTTTCAGGGAAGGAGGAAATGTTACCCATTCCTGAAGGTATTCAATCAAATGATGCTTTATTACCAAAGGATATATAGGCAATTCCATTCAGACCGCTCTCTCAAAGACGACTGACATTTCTTCAGCTTGGGCATAGGGATAGTGGCAGCTTCTTGAGTTAGAGCAACTAAAGCCCCGGACATATCAACATCTTGGAGTTCACCCTTTTCTTGCATGTAATTCTCTACATTCTGCATGAACTCTTTAGTATTTAGTTTGATCATTGGAATTTCGTCAGGATCTTCTGTCCAGAAAGCATCCTCAATTGCACTTTCCAAACTGTATAAGCACTCTGGCTCTGGTGTTGCTGGCTCTTCAATAATAGGTTCGCACTGCATGAGTATAGACCTTGCTTGTGAGTGTGAGCTCTACTAAACGAGTCCTATTGAAGGCTAGGTTCAACCAAAATAAATTCTATGATATATGACCATGGTCTTGAAACATGAGTCCGTGTCCTCTTAAGAAAATGTCTTCTATGTAAAAGAGGGGGTCTGTTTAATTTCATCAAAGTTAATACTGTCTCATTAAAGAAAGCAAAAGTTCATG

>ONT.9880.3 LG05 104577648-104585613

GAGTACAAAGCCCAACAATAGTGGTCTCCCTCCAAAGTCACCCCTCCCAAACCCTCTCCTCCCCCCAAAAAGCCAAATCCCCTTCATCTCTCTTCCAACACCACCACCTACACTTGCCTCCCTCCCTCCAAATGACGATTCCTCCTCCATAAAGGTCGCCAATGGCTGAACCCTAACCCTTCTCATCTCTCACCCGGAAGAGGGTTCTCGCATCTTCTCTTTTGTCTTCATCTCTCCAGCATCTCCAAAGCAACGGGCCTATTCTTTGTCTTCTTCGCCTTCGTTTGTTTGACACCTCCTCTGTTTTTGTCTCTCCGGCATCTCCATGGCAACCTTTATTCATTGGATGCATCCAGTATCCAGATGCACTCTGGCAATGTGAACTGGTCTTGGAATTGAATGGACCCAGCATTCTGATTAATGGATTGGACAGCATACATTTGCATTGTCTGTAGCTTTTGTTTCTGCTAAGGTTCATGATAAGAAAAGAAGCTCTTTGGGCCTGCTTTGCAGTGGAGCCTAATTTTGAAGCAATGATTTCGGTTTCTTTCTTCTCTTGAAGCATGAAGGAATCGATGGATATGTTTTTGCTCTTTTTGTGGGTGTCCATCGGTTACTGCTGTTACTGAAGGTTGGGACTGAAGATCAATGTAATAGTGGCCAATTGCTTGAACATCTTTTTGTTTTTCATAAAAATATTGTACTTACACTATTTTGGATGCTACATTTTTACTTGAATTAGTAACAACAACGTTGCTAAACTTGTGGGTCGTATTTAATAGTTATATGTTTCCTAATATGTTGAAAGGTCATATATTGTACATTTTTCATTAGTTAAATGAGCAGACTTTACTGTA

>ONT.14983.1 LG10 61667721-61669942

AGGCTGATCCAATGGTACTTGCGATGAGTTATTTAAAGGGTAAAAAAGGGAAAATCGCCCAAAGCTTTCATCTTTGCCGCTCGTCTCTTCCTCGTCTCTTCCCCATCTCTTGTACTCTCTGCCGTCTCTCAATCGCCAATCTCCTTGCAGATGCCATGCACTATATATGGTCTGACTTGATGTGTGTTTAATGTTTTATATGATAATTTGTGGATTTATGGAAGCTCTATTTTGTAGGCTTTAAATGAAGACTGGTTGACATGGTTTCGCAGTTGCATGTGGTTGTGATTGGCATGAGAAGTTGAATGTTTGTTTTGGATGCTACCCATTGTATGGAATTAACGGCAATTGGATTTTGTAATGTTGGTGGTTGTTAGAGGTTGTTTAGTTCTATCCATTGTTTTTTATGGGATTTGATGTTGGAGGTTGTTATTAATGTAATTTGCTTAGGTCTATTGGATTTTGGGCCCGACTCCAATGTCTCGAAGTTATTTGATTTTATGTTGGATGCTGGAAATTGGTGTTAGATGTTGGATTTTGCAAATGTGCTTCGAAGCTGTTGAAATATTGCCTTTTTACAAATGGATATTCCAGTATTATAAATGAATGTATAGAATGTT

>ONT.1209.2 LG01 28764966-28813955

CGTGATCTTGAGATGGGGAGCGGGCCTTCCCGAACTAGGTTGTGGTTCACTACCCGGTTTGGCAAGAAGAAAAATGCTTGGGTGGATTCGAGTTCTCATGAAGTCTATGACGAGCTTAAGAAGCTAGAGTCTCAACCGTTAGAGGAGAGCGATCAGCATATATACGTGGTCGTGGGGCTGGTCCAAAGGCCACAACATGGACAGCTGGGCAGCGGATCTGTGTGCAACTAGAAAGGGAGAATGAGGAGTTTAGGAGGCGAGCTGAGGAGGATAGAAGGCTCTTTGAGGAGATGGAAAAGGAGAAAAGAGAGATGGCTTTACGACTAGAGTCCCTAGAGTCCTAGGTTGCCTCGCAGCAAGCACAGATGCATGAGGTGTGCAAGCTTCAGTAAAGTCTCAATTCACCACCCTCCTCCAGTAGTTCAAGGATGGAGGGCAAATTCCGATGTGATTCATAACGGGTCCAAATTCCGATGTGATTCATAACGGGTCAAAGGCCCCAGATCATAATTTTTTATATTTTATTTGAGGTTTTATTTTATTATGATGATGTAGACTTAACTTTTGCTAGTTAAATACGTTGGAATTTAATGATTTTGAATTTGAAGT

>ONT.506.1 Contig01483 17661-20043

GGAGACTTCTTCTCTTCCTCCTCTTCATCTCTTCTTGCATGTGCTACCTAAGGGAGAGATTTCAAGAATCCAAACTCACTAGTCTCTCTCTCTTCTTCCTCTACATCTTGATCCAAATTGGAGAAGGGTTTTTGGAAGTGGTTTGGCTAGGGTGTAAGTAGAGAGCTCTCCTTCCTCCTTTATCTTCTCAAATTTTATTTCTTCACTCTCTTTGGTAAAGGCAAAGGACACCTTCTACTATTACACCGACCTGGATGCCGAGGCAATCCCAGAGGATCCTCAACTGAGAAAGGAGGACGAGGTACGAGAGTGTGATGCCGATGACATAGTTTAGCGCCATCACTCCCGGCACTAGTGCGCGTTATACGGGGATATATTCTATCGTGCCACCTCATTTCATCTTTTCGGTTAGATCTTTTGAACTTGTCTATCTTAGCATTGTATATCTTTTGTACCAGGACCTAAGTTTTTGGGATGGTCCTCATGTTTATAAACTTTATATAGTTTATCTTTTGAAACTTCAACATATATAATGGAATGAAATCCATACTTGCATTGATATTCTT

>ONT.10102.7 LG06 15931429-15947193

AAAAAAAACCCAAAATCGTCTCTGGCTCTTTCAACCTTCCCTCTTCAGCTCTTCTCCCGGTCTACAGCTCTTCCGACCTAGTGACCTTCCTTCTTCCTTCGACGCTTCGACGGCTCAGGAGCAAGCCTCTACAGATATGGGATAAAGAAGTAAAGAAGACTATATGGAATAACTTCTCTGTGCTAAGTTGTGTCAACACATTCACCCTTTTTGTTCGATGAAAAGCTACATCAAAATGTTGCAAACGATGGTCCAAAGAAAAGTCTTCTAGCAGAACTAGACGAAGGTGATGGTGTAGACACTTCTTATATATGAGGAGGCTCAAAATAATATACTTATGCAAGAAAAGCAATTTGTTGCAAAGAATGCTACAAGTTAATTGGAGGGCTTTTCCACCACAACAATTGGAATCAGCTAGCAATAAACAACATTGGAGAAAGTTAAAGCCACAACAAATATGATGGGATAGACTTTTTGGTTGTTAATTTTTTTTCAGAAATGGAGTGGATGAAAGTAGCTGGAAGGTTATATGAGGTGGATGTGTGATGTATGGATATTCAAATGTTAAATATTTGAGTTCAAGTTAGTCTATTGATTTCA

>ONT.260.8 Contig00734 42534-43746

ATCTTCACTTCCTTTGCTCCTCTCTCTCTCTCTTCAAATGCTCTGTTTCTCTTCGTTTTCAATTGGATTTCTTCGAGTTAATGACGACTGAGCTCTGATTCATCTCTTCCGATCGTTTAATTGCACTGCTTTCTGTATCCAATTCGACATGGAACGCAAGATTCTGAAATAAAAAAAAGTAGTAATTCGAGATTTGGATGCGTGGTAAATGACTTTTCATTTCCTGTTTGGATGAAACTATGAAACATCTAGTTGCTGAAATTGATTGGAAATGGCGGTAAATTTGGTTCCAAGGCGGGAGAGATTCACTCCGATTTGGTTGCTTTTGATGAGTTTCGGTGTTTGGACTTTGGAGTGTCTACTCGATGGAAGGCAGGCTTTTAGTTAATAGGACTCTGGTCCGTGGGGCTAAAGCTACAGGAGCAGGCAAGTTGTTCTATCAATGAAAAAACCCAGATGAAGAAATCATCAGAAAAACAAACCCAGATTAGCACTGGGTTTGATGGGACAGAAGCAAATGAAGGAGATGCAAACGTTCTTGTACTTCCTGCCTTTCTTCTGAGCAATTACCACTGTTGAGAAGAGATTCTTGTCAATGGTTCCTGGGTTTTGATGGGTTTTTGTTATGGAGATGCTTGAGCTCTGCGGCACTGAGCACTCCGTGAAAGGGCCTTTGCAGACATCTCTCTCTCTCTCTCTCGCTCTCTCATCTGGTCTGGTTTGGCCACAAGAAGTTAGATTTGAATGGGGTTTTTGTTTGGTGGAAAGATGTCCCTTTTGTTATAAATGGGTGGCCCGCTCGGATGAAAGATCTACACCGTTGCCTAAAGTTTCTATTTCGATATTGGATTGGAAGATCCT

>ONT.3697.2 LG02 108174072-108175692

TCATCTCCAGCACCTTCCTCCAACAACCGTTCCTCCATTTTTTCTTCTTCTTTCTTTCTCTGTTTCCATTCTTTCTTCCATTTTCTCTTCATCAAAGGTGGATTTGAGGTGAGCAAGTTAAGAGGACTTCATCTTCTTCCTCTTCATCATCATCTTCATCAATTCTCTTGAGTTTTGAGTTGAATTGAAAGCTTCAAAGGGAGGATTTTATCTCAAATCCAAGTTAATCAAAGACCAAGCTTCATCTAAGGCAAGTTTGTCCGTGCCAATACCTCCGAGACCGTCCCGTCAGCGGAGGAGACCCATTCGCTACTTCTAGGAGGTTAGCCGAATGGACATGATGTGAGAGCGAACACGTGGGAATCTTTTGAGAACTTTTTAGAAATGATTTTATAGATTCATTTTGGTATTGTAAGTATATTCCCTGCATTGTATTTTAAATGTACAGTGGTTGATAGTAAGAAACTGAGTTGATGTACAGTAACTTTACTGTTCAAAGTAAAGTGTTTGATTAGTTTGATT

>ONT.4635.3 LG02 39780397-39798883

CTTCTCTAATCTAACCCGTCCCTTCTCTCTCTCTCCTTCATCCTTCGCCTCGACCGCACCAGACCACAGGTCTAATCTGAAGGAGGATTTGGTGTTTCTCAACCAAATTTATTCCTGTCTGGCCCGCCAATATCAATGACAATCAAGGAAAGCTAAGGGTAGATAATATGGTTTCAATAAAGCAATGTCTAATGTTGGTTCAAGAAATCTAGTATGGAGCTATCAAAGTGTCATAGAGATCCTTTGACATGCATTATTCTGAATTGCCGATGGTCAAGCAGGAGCAGAAGAAATGGATATATATGTGAACAGAAACTCAATACTCTTGCCTTTTCTTTGGATTCTCAATTCTCAAATGGACTATGTAGCTAGTTTGAACGTTTGTGATGTAAATTTATCTTAGCCACTTGTGTTTGGGTTTGGATTTGTTCAACTATTCGGCAGGTGGCCACTTATTAGACAGTTGTGTGTTATATTAGCATGC

>ONT.4637.4 LG02 40211387-40213049

GAGGAAAGCGAAGCTCCTCCTCCCTGCGGACTAGAAATATATAAAGAAAAGGTGCTCTCTCCCAACTTCCTTCTTTACGTCTCTAATCAAACCCGTCCCTTCTCTCTCTCTCCTTCATCCTTCTCCTCGACCGCACCAGACCACAGATTGCAGAATTTAGCGCATCACATCGATATGGGGTCTGGGGTTTGCAAGGAATGACTTCTCCAATTTACCAAATTTACCAAACAAACGTTGTGGAGATGCTTTCTTTTGTTGCTACTTGTGGTGTATTAAAGTGAGGTTTCTATTACTGGATTGAGTTTGTGAGGGGCGGTTTGCTTCATGTTACTCCTTAATAAAGCTGCAAAACAGCCAAGGGAGAAAAGGTGAGGTTTCTATCAATCTTTCATTTTTACCCTCTCCATGTGAACTGTGAAAACTTGGTAAAGTAGGTAAGATTAGAGATCACTGTCTTCATAAGTGTTATCCTACCTCTACAGTGAACAGTATTGCCGCTTGCCCGACGGGAGGGGTTTTACTTTTGGAGGAAGTCGCAAATAATTTGGCATCGCAAAATAGGATCATATTGTTTGCAAATTATGTAGATGGTGATTTCAAAAATCTCACTACCTGGTTGGGATGGTCGTACTCTTTTGCAATTCTTTTGTCCATATGGAAGGGGAGACGATAGGATTTTTAGAGAAGCTTCATCTTGTAAGAATGTGTTTGTTCTTTGCGGTTGACCTAAGGCTAGTGAACGGGCCCGCTTTTAAGGAAGGGGTTCTGTAACTTAAAGCTCGATAATATTCTTCATGACTGGGAAGTTTGTATGGAGTCTAGGAGTTTAAAAGAATGGTAGTTGGTCAGTTGGTCACCCCCTCGCAAAGGGCCTTTGAAATATAATGTTGATGGAGCAGTTAAAGGCAGGCCAGGGCTGGCAACTGGCAAGTATAGGTACTGGAAGGAGATGCATTGTCCATGTTTTCTAAGCATGTGGGAGTGAAGGACTCTAATGAGGTTGATGTGCTTGCCATTGTGGCCCTAAGGATTTATTTCTGCAGCTGCCTCGACAGGTTTATTATGGAGTAACTCTTCCAATGCCATATTATGGAGTAACTCCCTAGGGTATGACACTATATGGCTTTGATTCAAGTGGCTTTTCAACATGTTAGTAGATTTGCTAATGGGATGGCAGGCTCGCTAGCCAAGCAAGGGCTGGGTAGTACTGTTGGTTCAGTGCTCCTATCGTATAATATTGGCATTTTGGGCGTTCAACATTTAGTATGATGTGCTTGTACTGACACTCTCTTGCTTTCACCATTCTCTTTTGTCTGTACTTGTGCTCAATTTTAATAATTTTTTTTCACTGATAAAAA

>ONT.11157.1 LG07 5637397-5638877

GAAATCGTCAGACTTTTTGTCTTTCTTTTGCCTACTCTTCATCAGACTCAAATCACTTCCCCAAAATATCACTTTGTTTGCCCCTTTCCATCCCTATATAGCTCCCCGCCTCCTTCCAGGCACCGATCCTCCTAGGCGAGACTCTATCCGGCGATCGTTTTGCTCTAGCGAGAACGTCTCTCTCTTCGGTGACGCTCCTTCTCCTGCCCTGGGCTTTTCTCTCCAGCGACTGTCTCTTTGAGGGATAAGTGTCGATGGAGAAGGCATGTTGAGAGTTGACATTCTGGATCATTTTGAGTTGTTCATGAGACCTGACATTTTGGATCATTTTTTAGTGGTTGAGAGTTGATTTTATGGTTGATTCTGAGTTGTTGTATTTGGCATGTAATTTGATTTTTTGTGTGTTTGGAATAGATTTTTTTTTGGATTTGATATGGTGTGTTTCAAATTTGTGGTTTGAAATTTTTTTGTAGAAATGAATGGATGACTCGTCATGT

>ONT.15102.1 LG10 81512094-81516051

CAAAAGGTAGCAGCGTTTTTTACTCCCCCCAAACTACTTCCCCTTCACTTTTTCCTCCCTCCCTCTTTATTTTTCCCCTCCCGCGCCCTGCTCCCCGCCCCCAAACCTCTTCCCCTTCACTTTTTCCTTCCTCCCTCTTTATTTTTCCCCTCCCACTCCCTGCTCCCCTGCGCCCAAACCAAGCCCTCCCGTCGCCCCAGCCGCGTCACTGCCGGAGGCTGCCTGCGCCGGAGCCACCCAAACCCCCTCCCTCTCCCTCTCTCTTTATTTCTCTCCCTCCCTCTCCCTCCCGTGCGATTGGAGCTGCCACCAGTTCTCTCTCTCATTCCCTGCACCTGAACTGCCACTGATCCTTTCTCTCCCTCTCCCTCTTCCTCTCTCTCTCTCTCTCTCTCTCTATCTCTCTCTCTCTATTTCTCTCTCTTTCCCGATTTATATATGTATACAGTTACAGATTTTTTTTTTTTTAATTTTTTTCCTGTTGTTGTTTTTGTGTATTGAGTGTAGGAAACCCGCTTTTTGCTCGCACCATTCAATCTTGCTACAGCTGCTGGTTTGAGAGCATTGGATATACCATGGCACCCTGCACGGAACTTTTTTTTTCCAGTATTTGTAATGGATATAATTTGTTGAAACTTTTGTATTGTTTTGAATGTAATTAATATATGACTTTCTTTTGTTATTTGTAATTTTAATTGTTGGTAGT

>ONT.641.9 Contig01843 19683-38693

AGCCAGCTTCGAACTTCTAACTGCTCAATTGCAACTGCAAGACCACTTGTAAAATATGGATTCCAAGAAGGCAATATCCAGATTGGTGGGGGAGGGAACAACCCCCAGGTCCAAGGGAATATTAGTGCAGGTACTGGAGGTAACATCAATTTAGGAGTTCAAAGAGCAAATGTCAATTCGTGAAATCAAGTAAGAGGTGGTGGTAACATCAATACGGGAAATCAAGGATATGTGGTGGTGGTAACATCAATACGGGAAGTCAAGGATACGGTGGAGGTAATGCCAATACGAGAACTCAAGAATAAGGTGGTGAGAGCTGGACCCACTGAAAATTAATTTCGCCAGTTATCTATGTTTCATGTGTCTTATATAATGTTTAAGCTTTGTCTACTATCAACTATGTCCGTTTACTACGTGTGATGCAGTACAACATCATTTCTGCATTTTGTGGATTCCAAGTGGCTATTGTGTAATGGTTGCTTGCTTATTAATATAAGTTTGTGTTATATA

>ONT.8923.2 LG05 1158411-1159418

GCAGCCCCCATCTCCATTTTCTCTCATCTCTCCCGTTCTCTCCATCTCACTCTCCTCGCCGGACAACGACCACCGTCGCCACCGGTCACCCTCATCTCCGACGGACTCCTCTCCTTCTCCATTCTCTTCTCCACTCTCTCATCTCATCACTCATTTCTCTCACTCATCTCTCCCTCTTCCCAGCTCACAGCAGCCCATCCCCATCTCTTTTTCCCCTTTCTCTTCTCTCTCATCTCACCTCACTCATTGCCATCACCACTGAACCCACCTCACCATTTCTTTCATTTCCTTTCTTTATTTCTTTTTCCCCCCATCTCTGTTTCTCTCTTTCATTCTCTGTTTCCCTTTTCTTTCTTTGTTTCTCTCTCTTACTTTCTTTCTCTCTCTCCCTTGCTCCCAACGATTTTAGCCCACACCATTTCATTCCCTCTCATTTTCTCATCATCTCATTTCATTTTCTCTCATCTCATCTCATCTCATCTTCCTCACCCATTCCACCCACCTCACTCTCTCACTTATTTCCTTTCTTTCTCTCTCACTCCCCTGCCAACGTGGAACACCCCACCCACATCCATTTCACTCTCTCTCTCTCTCTCATTCATTCTCTGTATCTTTATCTCTCATTCTTTCTCCATTCTTTATTTCCTTCTATCTTCACTCTCCTTCTTGTTAATGGACCACCAAGAGCTACATCACCACTACTTAGTGGGCCACTCATCAAGTGGGCCCCATAACCTCCGATGATCACTATCATCTCCGCGTGGACCCCGCAAGCTCAATCATCTCCAATTTGCAAGCCACCACTCCATCCACCTATTTGTTTGGCTTGGTTTGTTGGACTATGGAGCTAGGATTTGTGGATTGTACACTTTCAACCAAAG

>ONT.6970.1 LG04 79595772-79600998

GTTGCTACTTGCTAGGCTTAGGAGAGGGAAAGAAGGGTCGTGGCGACTGGCGCTGGAGCTTGAGCTCGGCGACTTCAACGAGATGATGGTGGAGTAGGCGATGGCGACGGCGAACAGACGAGCGCGGCAGCGACGATGTACGATGGTGGAGGCGACGCTGCTGCTGCTTGGACTCGTCGTCAATGAAGAGCAGAGTTCCAAATGGTGCACGGTTCAAGCACATGTGATTTTGGATTACTGATACGCTGTCATTAGGTGGATTTGATGGATGGGGCTCTCCGATAGCCTAATCACCACTCTCATGAATATAGTATGTGTACTTCTATGTTGTCTGTGTGCATGCTAACAATTTTACCATTATGTAGGGTTGATCTTTGTATGGGAAGCTTTTTTTGCGCAATTTAATGGCCTACACAATTTTCTTGGATCTTACAACAGTACAGGTGTTCATATGAGGAAAAAAAAACATAATTGTCCATTACCTTATCTTGGTTTGAACTTATGTGTGACACTTATGATGCCTGGTGGCAAATGGGGAGTACTAATTGGGTTAGTAGAGGAAAAATATATCGTAACTTACAAATGAATTTATA

>ONT.2322.2 LG01 39218174-39220378

GCGAAAGAGACAAAGAACCCAAGGCAAGGCAGTAGGAAGTGGGATGTGGGAACAAACTAATCTGCTCTGAAGGATCCAAAGGAAAATGATTGAAAGCCCTGGAAACTTCCCAAGCCTCATCGAGACCAGAAATCATAATCCTTCCTCATTAGAGTAATCTGACAAGATTTTCGGCAATCCACGGCAAAATGTGTGAAGGTTCTGTGTTTCACTGTTGATTGGGCTTCATTGTTTCTCTTATCTTCATGTGTTAAAACAGAGAAGCAGTGGTGAAGATATTATGTGTGAAGCTGCCATAATCAAACTTGATGTGCTCCAGAATCATGCCAAGCATGATCTTGGTGGGGCTTGCAGGGCTCATATTTTGTACCTCTTTTCTTCCATTTTGTAATGTGGAGGTCAAAGGTGAAAAAAAAGTGCAAGTGGTTTCATTCTTTATAGTTCATATACAAAAACTGCCTTGGTTATCATAACCATATTAT

>ONT.11804.1 LG07 9331704-9333208

TAAAACCAAAATCGAGATGGGCCATCAGATGGTGTTTTCATCTCCCGCTTTCATTTCCCCCCTTTCATCTTCGGAAGACCCAAGAGAGGTTTTCTCATCTCTCCCTCTCTCGTCGAGCGCTCTCTCTTTCTACTGTTCTCTTCGCCCTCTCTCTCTCTCTCTCTCTCTCTCTCTCTCTCTCGCTTTCTATTGTTCTTCCTTTCTCCCTCTACTATTCTCGCCGTCTCAATTTGCTTGGATACCTAATCGAGGTCTCTCTACTGTTCTCCCTCTCTCGATTAGCTCTCTTTTGCTATTGCTCTCCCTCTCATGTCGTCTCTCCCGCTTTAGCGGCGCTTATGAAGTCAGATTCGTTACAATCTCCACCTGGATCCGGAAAAGGGATTTTTTTGACAAGGAGGTCGGACCATCGCTGTGTCCCAGAGGTCTGTCTCGTTGCAGCCCAGCCCCTTCGTCAGCCAGCCCCTTCCACAAGTCGGAGTGATCTTTTTTGGTTCTTTTGTGGGTTTTCGATTTTTTTGGTTTTAGTCCATTAGAACACCAGCAACAGTTTCAATTTCAAAGGATGTCAATAGATCTTCTAGTTTCAATTGATGTTTTGTGATGGATTGTGGGTTCTGAGATCTGGGTTGTTTTTAGTTTTGATTTTTTTATGTCCAGACATTTTTTGAGGGATCGCGCTATGTTTTGTAGTCTCTTCATTTGATGGAGTGTTTATTCATATTTTTCAATTTTAGCTAGTTACAATAACATCGATTTGAGTTTTCATGGAAGAAAACTGGTAGATTTGTTGTAGGGAAATTTGAGTGCTATCATAAGTTCCAAGCTAAGATTATCAATTATCATGTCATCTTCAAGTATGTGATTGAAAATCTTTTATTTATTTCTAATATGTAATTTTTATATATGTTTAGAGGTAATATGAATTTACATATTAGCTTGAGAATACGTAGGCAGGTGATTGAATGGGATCCAAAGTGCCCATGTGGATATGAGTTTATGATTCCAAAGACTCACGTACAACATCCAACCCTAGGCGCAAATTTTGGAAATGTCTAGATTTGAAGTGCAAGGAGAAACAGGTTGTGGTCTTCTAATTTAGATGGATGAAGTTGCTTCGGAATGAATTCCCAACTCAAATTCAAGACGTGACCATGCCAGATTGAATTGAAAATGGAAGAAATGAAAACCTCTATTGAAGATATGAGGAGGGAAATAAAACATCTAAGAGAGTATAGAGCATCAAAATATGGGAAGCAGACGCAAATCATTGTTTGTCATTGTGTTCTTTTTTGCTGCATTTATTTGGTGTTTGAAAAATGGACGGGAGAGTCTATTAGAGTTGATGTAATTAATGTGCTTAATAGTTAGGTGTGATTTGATGTAACTAATTCGGCCCAATTTCCAACTTAGTAGTGAAATCGAA

>ONT.4205.1 LG02 10196445-10197584

GACGACGTGATAGAGTATTAGTGGTCCACTCTCTCTCTCTGACGTTGCAATCCTCCCTCCCCATCAGTAGCGCGCTCTCTCTCTCTCTCTCTAATCAACCATCGCAGCCCCAATCAAAGCGCTTCCCACTCTCGGCATTTGATACCCCCATCGCAACAGCAGCCACGCTCCCCACGGCTCCGCCTCCACATCGCAGCGCCACGCAGCAACCCATCACTCATATCTCTCTCTCTCTCTCTCTCTCTCTCTCTCTCTCTCTCTCTCTCTCTGTGCGGATCCAGCGCAGCAACCCCATTCAAAAATCAACCGCCCTCGCAGCAACACCCCTATTTCTCACAAACACTTAACAGGATTCAGGTTGGAAGTTTGCATGATCCTGATGGTTGCTGTGACCGGCGAATAGTTTGTTGTGACTGTGTTGGAAAGGTGTTAAAATATTAAAAATATTCTTTCTCGCCATGTCTATTGCAACCTCTTTCCAGACTCTGGCCAATTTCTCATTGAAGATGGCAGAGAAGAAAGAAGGTATAAGCCAGTTGTCAATTTCAAAGCTGACCAAGGAGGAGAAGAAAGAGAGTGCAAAGTCACCCCCAAGTGCAGATTAGGAAGATAGATTAGGTAGATGAGACAGAGCCCACTGTGTTTTGCACTTGAATTTGATGGGCTGCACTCCTTCGAGACTCTTGTTTCTCATTCATAAATCACCTTGTATTTTCAATTTTTTTATTGAATTCATATCTTGATTGGGCAAGGAGAATGTACATTTGAAAATTTCTTTCAGAATTATTTCAATTGAATTC

>ONT.10157.1 LG06 43717150-43721866

GCGAAGGTCAATATGTTTTTTACCCCCTAATCTATCTCATTCGCCGGCGGATTTCACCCGCCAACTCCACCAAATGTCCGCGTCTGTATCCCCATCACCTAAGGTCGATCCCTGCCATTCATCAGAAGACCACCACATCCTTCACCTCCGAGTCTCTCTCGTCTCCCTCTGCATCTCGCTCGGTGTGCCACCTCCTCTGTTATTTTCTGTCCGGCATCTTGTTTGGTTCAGAGAAATAGAGAGAAGCTATGGCGACGAACCTGGTTACATTTCAGAAGGGATCTGGGCTTCAGATTTTCTGTAAGTAGAGAGACAGAGACAGAGAAAGAGAAAGCGATCGTTCCCCCTACAGAGTCATTGAAATCACGCCTCCGCCCAAAAACCTCGGCATTCGCTGCTTCCCCTCTGGAGTTATAACGAGTGTTGTGAAGAAAGATGTATGAGATTTTGAAGCTAAGGCCTATTGGTGAGAATTCTGATGGATACGTCTTTCCCATTGTGAATCTTGAGGCTTTGTGTGTCACATAAGGAAGGCATAAAAATCCTAACCCTTGGACAACAAGGGTCCACATCCAAAAGATGAAGGCTAAAAATTGGATGGTTAGGATCTTCCATTTTATGGTCTGCTCATCATTGTCCTGGTCTTCAATATAAAGTCTTTTGGTGAGTTTTAGATTTTTCTTTTATGGATGGTCTGCTCATCATTGTCGAGTCTGAGTGATGCTGTCCGTAATAGGATTCTTCCATATTAAACTACAGGCTTCTTCCATATTTATTGGTGTGAACTGGCACGTGGATGTCCTAATGCCTTGATAATGATAATAAAATGTTAAGTTCTGATGAA

>ONT.12301.1 LG08 4522110-4525567

AGTCAGCCCCCATCCCAAGACCAATTCACTGTTCCATTGCAATTTCCCTCTTCTTCCTCTCTCAATTTCCTTCCTACGGATGAACTGGTATTGCTACTGTGTTGCTGCTGGGATCCTTCCCTAAGAGCCCCAATTGCTGTCTCATCTTCCATCTTCCTTTCTCATTCCATTTCTCCAGCTGCTGTTGTGGTTCTATGTTAGGAGAAGCACCTCTTGGAGCAGCAGCAATTGATCTTCTTCCTCTCTGTTACTGTGCTGGGGAACTCTTGATCTGCTACTCTTCTTTCTTCTTTGTTTTCTTCTTCTTTCCCTCTCCATTTCTCTACCCTACGTTGAGCCAAGGCTGATACTGTTGCTGATTTCACTGCTGGAATTTCCTCAAATGGCAGAGCTGCAGCTGCTACCTCATCTTCTTCTCCTCCTCTGTTATTGCTGGAAATCGGGGTTCCTTCTCTTCTCTTGCACTCTTCCTCTCTATAAACCTTCTCTCCATTATTAAGGCAAAATGCTGTAACTTGCCAGATCTTTTAGATCTGGCCCACTCATTGGTGATTAGGCCATGATCCCGAGATCCCAGACGATGCACTAGCTGTAGTGGAGGCACCTGTCGCAGGAGAGTATGCGTGGGGCCCGGCCAACACGGATGTCGAGCCGGAGGGTGGAGACGATCTATATGGGCTTTGAGGGACTCCATTTATTTTGGGTTTTGAGAGGCACAATTTTGTAGCGAGACGGGTAGATTCAAATGTATTAACTTTAATTTACATTGAATCCTTTTAACTAGACTAATGTTCTGCTCATACTTAATATATATCGATACCTATTGCTTCCGCATGGTATAA

>ONT.11931.1 LG07 54455956-54459198

TTGCATGCTCGTCCCTCATCTCTCCACAACTTCTGTCCTCGAAACACTCCGTCGCTCGTCGCTCCCTGTAATCTGGGTCGACTCCCTAATAGGTCTCCTCGCTCGCGATTGCCCTCATCGTGCGACGAATTTCAAACAAGTCTTTCTTCCAATTCTAGGTTTATTTAGATTGTGAAAGTAAAATGTCAACAGACTTGGCAGGCTTCAGAGCATTCAAGCATTTGACAGACAAAATTTAGGTCGGACGTTTGGAGTGGTCATTGTTGAAATATTCAAATTGCTAAAACATATTGAATGGTCCCTGTAAAACTGTTGAACATGTTGATTGGTAACTCTTGATTGCATTTCACAAATGTATTTCTGTTGGTCGCCCTGAAAGTTACATTCATAGATAATGGTTGACGACATTTCTCATGGATATATAAAACTTTACTGGTTAATCTTTGTT

>ONT.10036.1 LG06 7103507-7120803

TCTCCCCACTCAAATCCCCACTCACTTCTTTCTCTGCCCTCACTCATCTCCGGCTCCCTCTGCTTCATCGCCCCCACCTCTCCATCGTCGTCCTCTCATCACCTCCATCCCATACCCCCCTTCGCACCCCTCCAGAACCCATTTCCATCGCCCTCACCGCTCATCCCTTTCTATCTCTCCCTCCCTCTCCAACCTCTCCCACTCGCACGACCCACTTCATCTTCCTCCGCCAGACGACCACAGCAGCACTGCCAAGCGCGCGCAGCAGTCCCCGGACGCCCTCAGAGCCTTCATCTTCCTCCTCGCGCCGCCGGACGCCTCCCCTGCACCAGATTTGAAGAAGTGCAAGTTATCTGAGGATTTGGCTCAAGATAGATTAGAATGGAGAGAGAGAATTCATGTAGCCAAACCCAACATAGTTGCAACAAGGCTTTGATGATAATAATGGTAATGATGGCTTGCCCGAAGAAGTGATCCACTCTATGACTTGCTTATTTGATCTTCATAAGGAATCTTGGTGATTTTGACGGTTTTGTTGTGTATCTTCAGTGACTAAGACATATCCTTCATAAAAACTTTATTTGACTGCTTATTCCAACTTCCTATGAATGGAATTGTGTCCAACCATCCTTTAGGTGGTGGCAGTTATGATCATCAAAGGTCACTGTTATTAATGTTCTTCCCAGGTGGATGAATTTTTTTATGGTAGGTGTTGTAAGTTTATAAACCATTTATGAATGAAAGCCTGATCTGTATCTTACTGAA

>ONT.10564.3 LG06 6396492-6400644

CTCTTCCCCTTCCCATCTCTCCGAAGGAGAAAAAAATTCCAAGGAGCTCCTCTCCCTCTCCTGCTTCCATCTAGTGTTGCTGGTGCTTCCCTTGTGTGCTGCAAATTTCATGTTCATAAGATCATCCTAACCCCTTTTATCTCTGGCGCTCGAGTGTAAAGGTTCTTCCTGAAATGAAAAGACTTCATGATAAATCAAAGATATTAGCCCAGATTGTGCACTGCTGGACATCAAGGAACCCGCTGAAGTCCAAGATCAAACCCACCATTGGATGGCCCTCCTTATGCATACTGCGTTATGATCATGAGAAAATCAAAGCCTCCTGAATCCTGATGTTTTGTATTTCCTCTTATTGTTAATGTTTTTGTAAAGGGCTGGAGTTTCTCCCTATTTGGACAACGTTGTTGTTGTTTCATTATATATATTTTATAAAGAGATGTAATCGTATGATTGGAAGGGAAGTTTATCCTTCAGTTGTATGTTTGAACTTTTAATGT

>ONT.16140.2 LG11 20997458-21003056

GCATTCTCGACACCCTTGCGCCCTTTCACCCTCTTTCCCTCTCTCCCACTACTTACCCTATCTATTTTCCCCCTTTCTCCCTCTCTCCCCCCTACGCACATATTTGATCGGCAGCGGCACCTCTCCCTCTCTCCCACTCGGTCTCTCTCCCTCTCCTCAACACCTGCAGGGGATGAATGAATGCCCCTGCAGCACTTGCTTTTGAAAACGTAATGAAACGGCAGATGGTTTCTACTTTTTACTTTCCAGTGCTATCAAGATGATTTCCTTTCGTTTGCTTCTGTCAGGGCATTTGTTGCTACTATTCCTACTCCCATTTAAATACACTTTTACCTTCTCTTCTCCATCTGGGTTACAGGCGTCAAAACATCACTCGATGATGTTATTACTGCATATGAATCTGACAGCTCGGTGGCGCAAGGTCTTTGGTCGTGATAGCTAAGGATGCATACGTGGCTTAGGCACTGTTTCAAAAACGGCCATAAAACATTCAGCTCCTTATAAAAGAGCCTTGGAGGAGGAGCATGAAAGCAATACACATCTTCAAACTGAGGTGAAAAGGTTGAAGGAGGATTAGATGGGCATACGGAGGGAAATTGATGAATTGAAACAAACTCTTACCCAAGTTGGGACACAAGTGTGACCTTCAACTTATCATGATAGTTCATCTCAGGCTCAGGATAATTCCTCATGTCAAGTGGCATGTACAAATCAACCGAACATTAATACACAGCAGGGTACGTGCAGGTTGTTGCATTACGTGCGAAAAGATATTATTGTTGCCCTTGGACGTGTTCTTGGTTTGTCAACTTAGGAGGAAGGATGTTATAGGATTGTTGTAGATGAGATCTTAAAGTTTAACATAGAGTTGTTGGGTGGAGAAAAGATGTTTGGGGTTCTCACCGTGGGTGACATCATTTCATGGCCCACATATAGAACCGTGTTTGGTTAGTTACAAAGCCCACATGATTTACTATTTAGATGACTTTATTTGTAGTATTTATTATGATGTGTGTGATCATGGTATTAGTAATACCGAACGTGAACGTGGTTTTTGTTAATTTTTTACCATTTACTGTAGTTATACTTTGATGGTTTAAATTTTAAGTGGATGGATGATTTATTAGCT

>ONT.8237.1 LG05 20912347-20914765

AGGCCTTCTACTCCATTTTTGTTTTTCCTGCAACTGTGTTAAGAAAGAGTTTCATTCTCTTCCATTTCCAGCCACCTTCTCTCCTCCTGCATGTGTGCCTCTTGAGTGCTACTTGGAGATTTGACTTCATAAATCTCCTCTCCTCACCTCTAGCTTCCCATTAGTACTGTTCTCGAAGAAAATCAGCTCGGACTACCTTCTTCACCAAGTCTTTCTTCTTGCTTAGAGCTGTGAAAGATGTCGGACCCGTAAATTTTGAGGCGGAGCACGCGTTTGAGGCGGTAGAGCCGCCAGTGGAGCTCGAGTGGCAGCCCAACGATGTCATGTAGACGTCGTGTTGGAATTTGAGCTGGGATAACATTTTGGGAACTTAGTTTATGACCTCATCAGTCTTCTTTTGGGATGTATAAATTAAATATACTTTTGTGATCCTCACAACTTTAACTAACTTTGGGTTATACCTTTTATATATTTATCTTTTGAT

>ONT.7077.1 LG04 87907107-87911388

GTGTTTGTGCACTATCAATCTAAGAACAAGAAGGCAAAAGAGCCCTCCAAAACCTGTTTCCGTTCGCGCCTCTTCTACACCCAGCAGTCTTGCCCTCGTTATCGAGCACCCGATCATCGCTGGAGCCTACTCACCGAGCACTGTGGCAACAAATGGACATTGAATATACATTCTAGCAAATGCTTCATCTTTGCACATCACCGAAAGTTGTGATGATGTTATTATTGCATATGAATCTGACAGCTCGGTGGCGCAAAAGGTAGGTAACTATCAAGATGGAGTTACATTTTGGCAGTTAACCAAGATGACCAAGATGGAGTTGAAGACTTTTTGTAGTTAACTGGAACACTTGTTTTGAATCATTTTTGTCATTTGATACAACACACCGTGAATGGATGCTTTGTAGTTATGCTAAAACTATTAACTTTGAAGTGTTTATTGTTGAAGGATGAATATTTTGAAGGGTTGTGAACTTGGATGATAGAATGTTGACTTGTTGGATATTTGATTTGAATGATATGTTGCGTCTTTGTGGT

>ONT.9746.2 LG05 93053545-93081947

ACCCACATCAAATCTAAAACTCCTCATTTGAGTCATTTCCCCCAAAAACCCATCGATTGAAAACCCTCCTCCCCTCCCAACCCTTCTTCCTCAGTTCCTCTAATCCTCTCCACACCACATCGCACACCAAAACCCTCTAATCTCTGATCTCTCCAACCCTTCTCCCTCTCCACATCGCACCCAGGTCTCCAGCAATGGCGACGACGGCGACAGTTGTGCAGAAATTCGAAGACATCCAAGAAGAGTGATCTACAGGAGAGATTATGAATCCATCTGAAGACACCCAAAATCACAGGTACCATTTATCTACGGAATGAGAATTGTGTGTGCAACTTCAATCATCTTCACCAAATGGGTGTCCTTGAATTAAAGTCAATAGTGATCACGTGCGCAGATAGAAATCCTATTGGGATATTATTACTCTAATCTATAAGAGGAGAGCTGTTGCAGGTGACGATCAAAGTGTGCCTCTCAAATCCACAATTTTTCCAAATAGTTTAAACAATTGATTGGAAGAAGAGATTCTCTAGAAAGGTGACCTTGTCTTGCAGCTTTTGAGGGAACCAATGCAGTGACCTACAGGAGAGATTATGAATCCATCTGAAGACACGCAAAATCACAGGTACCATTTATCTACGGAATGAGAATTGTGTGTGCAACTTCAATCATCTTCACCAAATGGGTGTCCTTGAATCAAAGTCAATAGTGATCACGTGCACCGATAGAAATCCTATTGGGATATTATTACTCTACTCTATAAGAGGAGAGCTGTTGCAGGTGACGATCAAAGTGTGCCTCTCAAATCCACAATTTTTCCAAGTAGTTTAAACAATTGATTGGAAGAAGAGATTCTCTAGAAAGGTGACAGTGTCTTGCAGCTTTTGAGGGAACCAATGCAGGGCAATACCATGCATGGTGCGGGAGGGGACAACCACATGATGCAAGGGAATATCAACGCTACTACTGGAGCTAACATCAACTTAGGAACTCAAGTATATAATGCAGGAGGATGAGGATTATCTAGAGCTTGAGACTGAGGATGATGAGGACTACGAACTTGTGCCATAGTACAACTATAATGATGCAATTCTGGATGGTCAAAATGAAGATGTTTAATCTATGAGGATTTAAGACATTAAAGTCAATTATGTTTAAGACTATTAGTTTATCTTGGGATTGATGTAATTAATTAATCTCATACTTGGTTCACTTGGAATTTTTTTTTGGTGATGTAACAGTTGATGGATTGTTATTTATGGTTATGAGCTTGGCTTCTTA

>ONT.12069.1 LG07 71410466-71412016

GAGATCGATTCTTCTCCCATTTCCCTTTTTCCTTCTTCCATCGGTTTCTACCTCACTTTCACCAGCGAGCATCAGAGCACCGACGACCGCCCGTACACCGGTGAGCACCAACAACCACTGGTGAAAATGGCTATGACAAGCTTATTTGGAAGGATGGGGTATTCATGGACAAGATCGAAGATGCAAATTCCGCAGTTAATGTATCTAAGAAACTTGTCATTTGTCAACGTAGGTGGAGCGCTCTAAATCAATGTTGAAGTCGGTAGATATAGAGGTGGAGCACATGAATACATCTTTGTTCAGCCTGGAAATTGTAGAGATGGTATGCAAACTTTTTCATTTTGTTCAAATTATGTAATGTATTATCTTTGTAACCTATATGTTTTTTTAACATAGGCATGTGATCATGTGTGGAACATATAAGTGATACCGAATAATCAATTAATGTAAACTTTTTTATTATAGTGAAACCTAGCAATATTTATGTCTTCATATCAGCTGGAACCCAA

>ONT.2249.3 LG01 30393859-30397463

GTGACGAAATTAAACTAGGCAGGGATGGAGAGAGGGAAAGAGGAAGAAGCAGCAGAAGATGAAGAAATTGTCTGTTTAGATGAGTATTCCTTCATCAATGACAAGTTAAAAGAACATCAAAATTTATCACCATCCCATTTCTCCTTTCAATTTGAAAAAACCCTTCTCTCTAATATCTCTTTCTTCAACTTCTTGCAGTCATCAGCTCACCACATTTACATTTGGGTCCCAAGTTCTTCAGCTCTTCTGTCTGCAGTCTTCTTCTAACGCAGATGCAGCTCCCGACCTTGAAGAGGCCCAGGAGGGAGAGGAGGGTACCCAAGAGGTACCAGCAGGACTAGAAGAGTGGCCAGAACCAGGGCCAGCCTAGCTTGTTTCCTCGGAGTGCGGAATATATTGTAGATACCATAACTCGTGTGACTAACCATTTTCATATGGTTACACGAAATTCTTTTGAATATACTTTTGGTAGTGAATGTATAGGTTCTTCAAGGGTTGTGCAGGGATTCTTTCATTTGTCCCTCTTTTGAAATTTAATATGTATATTGTTTACTCCTTTGTATCAAACTTAAATGTTTAAATTAAACTTGATGGAATTTGATACC

>ONT.10169.3 LG06 49479756-49480719

GACATGCAGCATGCCACATGGCAAGAGGCAGGGAGCCATTTATCTTGCTCTGTGCACTCTACCACTAGTTCCAATAGCACCCCAATGGCTACTTCTGCTTCCATTTCTCCCTTCCTCAAATCCAGAGTACTTGCAAGGGATCAAAAGGAATCACAGATCTGAATCCTATAATTCAGGATATATGTGGTGAATCTCTAGTTGAGAACAAAGATGAATTTCTCAAAACTTTTTCAACAGAAACTCAGTTTTTCAGGTATGTACTAATGATTACACTGAAACATTTTCGCTGAAGGTGAAGGGCAACGGTATGTGCTTATATATGTGGAGGTGAGGCACATTTGTTCAAGATTGAGTTTGAGTTTTGATCATCAGGTGGGCCACAATTTATAAAAAATGGATAGTCGAGGAGAGAGATCGAATTATCCAAATTCAATTGTACTTTTGTCCCACCTGATGAGAAAACTCCCTTAGTCTTTGAGCCAATAATCTGTGTGTTCTAGTGAAGGGACTGGATATTGTGGATGTGTGGTTGGCCCATGTCTTGCTAAATAAGTTGGCAATTTGGTCATGTATTGGGAACTTCAACTCTACCCTTTTTTTAATGTGTAATTCAAATTTCAGCCTTTCATTTGTATGTAATTCATATGGCTATCTTTACAGCTTTGTAAATTTTCGTATTGATTTACTGCTCTCAGAAACATCATCTCAAATGGAGAAGTCATAGGCTCTGAAGCTTCCAAGGGGCATGATGATGCATCTTATAGCTATTTGAGGCTTCCATCAAGGACGTGCTCTCTTAATTTTACTTTTATTCTCATGTGCATTTTGTAATTTCATGATGTTATCTGGCTCTATGTCTGGATTTTTCAGTTAAATAAGCATTTCCAAAA

>ONT.7063.1 LG04 86971424-87017016

ATCTCTCTGACCCTCTCTCGCCCAGCAATCCCTCATCTCTTTGACCCTTTCTCGCCTGCCATCCCTTCCAGCGGCTCTTTCCACTGATTCCTCCAGCGGCTCTTTCCAATCCTCCTCCTCCATAATCACGCATTCTTGAGCTGTTTTTCAGATTGAATAAAAATTGAAAAACAACTTCTGTTGAAAGAGTCATTGCTGCTAGGATGCAGCCAACATGAAGGCCAAGTATGTAGAGTAGAATGTTTGGCATTTGAATGTTGGTATAGAGTAGAATCCTTTTGTGGTGAATGACCACTTCTCTTACCCGGTGATTCTCCACTGTGTGCTAGAAGATAAATCTGATTGGGTTGACAAACCATTGTGTACTAGATATATGCTTTCTGCTTGTGTATGTTGGCTGCCCACCACTTTAGAAATCCTGCTGTGACTTTTCTCTTATCATGCATCAGTATTGTTGAGTTGT

>ONT.7976.3 LG05 7475127-7483613

GGGTGGATTTAGAATTTCTCCTCCTCTGTATACGCTGTTCTGGTCACTTGCATTGTCGAAAAGTTTCGCCCCGATACAACATTATCAAAAATCATTCCGGCTTCCGTCTCAGGCCACTGTGTTTCATCTGACGACAGATCGAAGCTTCCAGCAGGTTGCATAGAAGAATTTGAGTATATCAAATGGCCACAGGACTGGAAAATATTGGTGGAACTGAATTGGGGATCGAAAATGTGTTGTTGGTGCGACAGATTGAGCCTGGGAGGCTGAACTGTGACACCCTTCTCAACAGGTTAGCTGAGTTTAATACTTATGCAGTAACCTGCAGCCGAATGGATGATTGTCTGTTCCTTGTGTTTTGTGGTGGCACAGAAATCCGGATTGACTAGGGAGATGCCCTGTTTCCTGTCATTTCTGTCCACATTGATTGTCTGGGGTGGAACAGGGGAAAGAAAGTATTAGAAGTGGAACAGACAGCCATGGCCAATATGTTGAAACCCTAGATATCTGCGGGCATCATCGACATTGAGGCGGCAAGCTTTGGTTATGCAGTTACCAGACGAGGCTCTTGTGAACTCGATATAGCGGTTCCATTCTGATCTGTAGTTGTTTCCAGTTTCCAAAATAATTAAAGTGTCTCTGTTTGGTGTACTCCTCTTCCCTGCAAAAATGTTATCTGCCAGGCATGTTTTTATATATCTGTAATTGCAGCTGATTTCCATGGCCTGAGGGCTCTTTTCTCAGGGGGGAAATTGACAGAATGAGAATTTATACTGGGTGGGCTGGAGATTGGAAGCACCGCCTAGACTCGAAGCACATGGCCGATAAGACGTGGCATGGCTGCATGGGGCCGCTTTTGCCCTGTACAGATTCGAAATGGAGACGTGTTGGCTTTGATATGATCTCAAATTGTTAGCCAATACACCCAAAATGCCAACGTGTAAAAGATGCAGAAAACTAACTACTTAACAGCCTTCAGTTTTTCAATTTTTTGGGTAAAGTAACATCCTTCAGTCAAACAAAATGACCCCGTAGGACAGTTGGTAGGTGCTTATTGTTGCACCTCTAGGGTTGTGGGTTTGGGTCATATTACAATTTAAGAGGTGTTCAAAATTATTTTTAATAATAAGAAATTCAGTTTTATTGGATCAA

>ONT.13487.1 LG09 50210898-50212286

ACATACACCCATTTGAAGTGGGGATCTTCTCGTTTCTGGTGTGTTTTCTCTCCAAGACTCCAACCCTCACCCATTTGAAATGGGATCTTCTCGTCTGGGCTGGATTATGAAGACCATTCTCCCTTCTCCATGATTAAGAAATCGGACCGTCAACATCAAGGAGGCTATTTTGAGGGATCATCTAGCAGCCCCAAGGCATTTGCAGAGAAACTTGATTGAGAAATAGTATTTTCCCTACCATGTAAGCAAGTAGAGGTTTTGCTTGAGGGCCAGAAGGTAGAAAAAATGGGCGAATAGCCATATTAAACAGAGAAGGTAGCTCTCTCTCTCTCTCTCTCTCTCTCTCTCTCTCTCTTGAGGAGGAAAAGAATGCCCAGGGACAAGGGCACTAAATAAGAGTAAGCTTGAAGGAAGGTCGGGATTTATGCCCTAATAAGTTGGTTGGTGGAAACAGTTTGCAACTTGGTAGCACCCCACCCAATTGTTTAAAACTGAAGTGCTCTCGGATTGCAGCTTTGAGATCTAATTTTTCAATATTTGGAATTTTTTGTACTAGTTGAAGTTGTCCCTGCCCCCACCAACCCCCATCCCCCACTCCTTTTTTTTTTTGTTTTTTTTTGTTTTTTTGTAGGTTTTTCTGCTTTGGTGAGGGACAGACACCCCCACATAATATTTTTTCAGACGTTTTTCTGCTAGGGTGAGGGATAGAGGCCCCACATTTTTTTTGCTGGTTTTCCTTCTGGGTGTGATTGGGATGCCCCATTTCATCATATGCATGTTTTCTCTGCTGGGGCTGGTTTTTTTGTTTTTCTCTGCAGCTTTTTCTGTATAATCTTATTTTTCAGAATGTCATCGTCCAATAATTCAACGATTGAAATGGTACCTAAATGCCGATGTGAGAGTGATGAGATGAAACTTTTGACGTCTCACACAAAGTCGAATCCAAATCGAAAATTTTGGAGATGCCCAAGGTGGAATGATGGAAATGGCTGTGGTGCATTTATATGAAAGATAAATGTCAGAGAGAAGAAGTTATTGAATCAGGTTCAGTGTCGTGTGACAAAAATTATAGAAACAGAGATTTGCAGAATCTGACATTGGCGATCACAGACCTCAAGCTTGCAATTGAGATGCACAATAGATTATTGCATGTAACACTAGAAGAGAAAAAAGAGAAGATGAAACTGGGGAGACTAAATAATTTGTATTTGCAGAGAATGTACCATGTAATGTTGTGTATGTCATGTTTTGTAATCTTGATAGTAACTTGGACCCATTTATGATCAATGGAAGCCTTTTTATTTTGATAGT

>ONT.7795.1 LG04 87603437-87607521

AAGAAGGAGGCAAAAGAGCCCTCCAAAACCTGTTGCCGTTCGAGCCTCTTCTACACCCAGCAGTCTTGCCCTCATTATCGAGCACCCGATCATCGCCGGAGCCTACTCACCGAGCACCGTGGCAACAAATGGACATTGAATATACATTCTAGCAAATGCTTCACCTTTGCACATCACCGAAAGTTGTGATGATCTTAGTATTGTATATGAATCTGACAGCTCGGTGGCGCAAGGTGATTTAGACCATGATGTAGTTACAAAGGTAGGTAACTACCAAGATGGAGTTACTTTTTGGCAGTTAACCAAGATGACCAAGATGGAGTTGAAGTCTTTTTGTAGTTAACTGGAACATTTGTTTTGAATTATTTTTGTCATTTGACACAACACACCGTGTATGGTTGTTTTGTAGTTATGCTAAAACTATTAACTTTGAAGTGTTAATTATTGAAGGATGAATATTTTGAAGGGTTGTGAACTTGGATGATAGAATGTTGACTTGTTGGATATTTGATTTGAATGATATTTTACGTCTTTGTGGT

>ONT.11527.2 LG07 74333621-74342743

CCGCTTTGGCTACTGGAAGACGGGTGCGAAGGTAATCTTTGTCTTTTGACATCTTCCTGTAGCTGTTGGTCTTGAAAAAATGGCTTCCACCTTCGTCTCTGCTGCAGAGCAATGCAAGGAGCATGGCAAGAACCCATCTTTTGGAAAATTCAAACGCTCATGGTCGTCCAATGATTCTTCCTGCTCAGCTGGTCAGGGGACCAAGACCTGCGTCTGCGCTCCAGCAACCCATGCCGGATCCTTTAAATGCAGGCTTCACAGGGTGTAGAATGATGATATGAGTACAACGGGGCATTTCAATGTATGCATTATGCAAATGCTGCTCAATGATTGGATGCATTTTTTTGGTTTCTTCTTAATCTGCAGCTCCTTGTGTCCTCTATATATAGACCCCGAGCTACCATTACTTCAGTTACTGTTGAGAATTGGAATTGCAGTCGGTAATAAATCTGATTGGTTGGTCCGCATCTATCATCGAAGTTGGTATGATAAAGGAGCAGAAATGTTGTCAGGTTTTGCACTCAACTCCGATGTAGATGGCAGTTCGGGTGGTGAAGAGACACATTTTAGTATTGATTTATCACATATTTGGTTTTCAAGTTCCTACTCATGTTGCTTATATAATGTGTTGTCTGTGAGTAAAAGAATATCCACAGACGGCTCTGTTCTGCTTATCTTCATCAATACAAATTTTGAAATGTGTAGAATCATGCAATCGTTTTACTTTATACT

>ONT.9712.1 LG05 88209360-88238706

CTCTTTTATAATATCCAGCTACTTCCATCTCTGAAATCATCTGTGTACTTTCTACAAGTTCTGATTTCAAAATCTTTGCTAAAAACCAGTGACCTAAAGGAGAGACTGTGAACGCATCTGAAGACACTTAGGTGCTCACTGGAGCCAATCCATTTATCTACTTAGAATGAGAAATTTGTGTGCAACTTCAATCATCTTCACCAAATGGGTGTCCCTGAATCAAAGTCAATAGTGATCACGTGCACAGATAGAAATCCTATTGGGATATTATTACTCTACTCTATTAAGAGGAGAGCAACATGTAGAGCTGTTGCAGGTGACAATCAAAGTGTTCCTCAAATCTACAATTTTTTCAAATAGTTTAAACAATTGATTGGAAGAAGATATTCTCTAGAAAGGTGACCGTGTCCTGCAGCTTTTGACGGAACTGATGCAGGGCAATATTATGCATGGTGCGGGAGGGAACAACCCCATGGTCCAAGCAAGGGATATCAATGCTAATACTGGAGCTATCATCAACTTCGGAATTCAAGTAAATAATCCAGGTAGGAAGAAAAGAGGGGACAAAGGTAGTTATGACAAAAAAAGAAAGCTGATTGCACATGAGTCGAGAGACTTAGGAGGTTGAGGATTTGCAAGAGCTTGGGATTGAGGGTGATGAGGACTACGAACTTGTACCACAATATGGCTATGATGCCATTCTGGATGGTCAAAATGAAGATGTTTAATCTATGAGGATTTAAGACATTAAAGTAAATTATGTTTAATAAGACTTTATTAGTTTATATTGGGATTGATGTAATTAATAAATCTCGTACTTGGAAGTTTTTTTTGGTGATGTAACAATTGATGGATTGTTATTTATGGT

>ONT.7568.1 LG04 66230326-66245783

TTTTCGAAATTAAGGCCTTCTTTACGAAGCATCATATTTCCAAATAATCTAAAAAACAGAGCTTCTTCCCAACCTGTACATTCGAATCAGGGAAAAGCTACACTGCAGGAACGTGAAACAGGCCATATGATAGGGAGTTAAAGAGACTGTGGAGGTTCTACAACTACTCAAACCAAAAGATGGAATCAACGCACTTTTTATCTCTCCATCTCTTTGCTACGGCTATCTCAATTCTTTTTCCTTTTGTTGTAAAGGTGAAGTACAAAGGAAAACACCATGTTACAGATGAATGCGAAGAACTATGATTTATGACCGTTGGGACTTGTTGATTGTATAATATGTAATTTATGTTTTTGTTTATCTGGAATTTGGTTGTACAAATGGTTTGGATTATGGGTTGGAGGTTCTTTTGGGACTTATTTAATAATAGATTGTAAGATTGGTTTGAATGATTAAAAATGTAATTTGGGGGGTTCGTTCAGCATCTGTTTGGTTTATGAATGGTCAGAATGCTATGAAATATTTTTTCTGTGCTGCTTTTCGTGGGGAAAGAAAATTGTTGTTC

>ONT.3610.1 LG02 100520970-100527879

ATTCCCAATTTTTTCCCCTCCCTCCCGCGCCCTGTCCTCCTGCACCCTGCCCTCCCGCCGCACCAGCGACGACACCGCCGGAAGCCGCCTGCCCCGGAGCCGCCACCGGTGCTCTCTCCCTCCCTCCCTCCCTCCCCTCCCCACCCTCCCTCTCTCTCTCTCTCTCTCTCATCTCTCTCTCTCTCTCTCTCCCCCCCCCCTTCCTCCCTCTACCTCTCGCTCTGAACTGTGCCCTCCCGTCGTCGGTGCCGCCAATCCGTCCTCATCTGCGATCCCGACTGCCCTTTCTCCATCACCGCTTTCGACGCCAATCACTGTCCAGGAAACTCTCTTTTTGCTCGCAGCATTCAATCTTGCTACAGCTGCTGGAAACCCTGTTTTTGCTCGCACCATTCAATCTTGCTACAGTTGTTGGTTTGAGAGCATCGGATATACCACGGCACTCGGCACGGAAAATTTTTCTTTTTCTGTAATTGTAATGGATATATTTTTATTGAAACTTTTGTAATGTTTTGAATGCAATTAATATGAGA

>ONT.1332.1 LG01 81502717-81509437

CAACTCAGGAGAAAATTCTCTCTTTCCCTCTTCTTCCTTCACCTATTCTTTCTTGATTCCAAAGAGGTAGATTTGATAGGTGTTTGGAAGAGAAAGCTCTCGTTTAGAGCTTGGCTTGACTGTGTTTGAAGTTTGGGTTAGCTCTTGATTAGAGGCAAGTATGATTTTCTAACTTGTACCCCTGTGCGATTTCTGAGGAGGCGGTTGCTGAGCGCAAGTGACTTGTGATTGAGGTGCAGAGGTTGTCATGTAGACTCCGTTCCTTGCACATGAGACGCGAAATATCTAGGAGTATTATTTAGGGTTTATATTCTGTTTTATTAGCCTCTGTTGGCCTTTTATTTTGGAATTGTAATTAATACATCTTTTGTAATTTCTAACTTGAAATATGGGAATGAAATCTTTGGTTTACTTGT

>ONT.12315.2 LG08 5261829-5266772

TCTCTTTCTTTATTCTTTCTTTCGTTTTTCCTTTTCCAGTTTTACCTCTTGCGTATGGGGGGATCCATCTATCAATTTCATCATCATCCATTTCTATCCCATAAACCCGCGTTCTTCCCCAACCATGAAACCCTTCTCGCTGCTCTGCCCTCTCTCGTTGATCTACTGCCGGAGCTCGAGCCATCGATCGCCTGAGCCATCTCCTCTCTCAGCCACAGATCACATCCGTGACGATCACGATTGCTATATATATTCATTAAAGAAAGATGAGTTTCAAAAGAAAAATGGGTTTCAATGAGTTCATTAAGGCATAAATTCCGGTATGAGATGGCGAGGACGAGATTCAGCTTGCCATTGCGATCCTTTGCTCAAGTTGACGCTGGAAACGATACTTAATAAGATTGTTGAGGACTTCTTTCAAGGGCGTCCAATCAGCCATCAGAAGCTTAAGGTACTTCTTGGTGATCCTTGGTTACAAATCATTGCCGGGGCACTTCTTGGCATATCGGTTACTTTTATTTGTCATTAAGGTTTCATGGGTGCTGCGTAAATTGTTGAAGCAGGTACTTGGGAAAGGATGAAAAGGGTTGTGGTAGATTCATATGGAAAGATGAAGTTGACTATGATAAGAATTTAGCAGCGCTTATCGAGGATATATGAAGAAGACAATGGACAAACTAAATGAAAACATGTGTAGCAGTTGAAGAGATGAGACAAGCGGCTGAAGAAAGGAAAAAACCAAATAAATTTGTTTCTGTAGTGGTGTTTGTCGGTTGTCTGATCTGATCTGCATTATGAAAAATGTGAGGTATATTTTGAACAAGCACGGATTTTTTTTTGTGGCGATTTTAATGGCGGTGGTTTTTAATAGTATATTTTGATCAAGCAT

>ONT.16827.2 LG11 53510719-53511470

AAACCCTTGGCCTTCCTCTTCCTCAAACCCCAAATCCTCTCCAAGGGCTCTTGCAATCCCAGCCCTCCCTTTCAAGCTCTGACGCTCCGGCTCCGACAATCCGGCATGGAAAGGAGGTGTGGTTTGAAGCTTACACCATCTCAGACTCTCAATTAGCAGAGCATCTGGAACCAAAGAGTGATGGTTGAGCTTTGTCTTGTCTAATCAAATTATTCTTTTGCAACGAATAGCAGTACATATGAGATTTCCGATGGATCATCCGAAAATCTGATTTCCATCATCAAGTGTAAAGCATTGACCATCTCGTTTCCCCTATCTGGTGCAGCCCATTTAATTGTGTGGCCTACCAGATGGCAAATCAAATCCAAACAGAGAAGTTGCCCCTCTCTTGATTGAGCAAAATCAAATGTATAAGCTTGGAAAGCAGGAAATATCAGCAATTATCCTGGAAAAGAAAGCTCAAATCCATCAATGTGAACCAAAATTTCATATTTTTTCAGTGGTTCTACTTCACATTCTCAATTACAAGAACTATTAGCTTGAGAATTCCTAGAGTGTACTCTCAACTGATGGATATTAAAGTTTCCTAAATTAATTCCCAATGGGAATTCAAATA

>ONT.641.10 Contig01843 19683-38694

AAGCCAGCTTCGAACTTCTAACTGCTCAATTGCAACTGCAAGACCACTTGTAAAATATGGATTCCAAGAAGGCAATATCCAGATTGGTGGGGGAGGGAACAACCCCCAGGTCCAAGGGAATATTAGTGCAGGTACTGGAGGTAACATCAATTTAGGAGTTCAAAGAGCAAATGTCAATTCGTGAAATCAAGTAAGAGGTGGTGGTAACATCAATACGGGAAATCAAGGATATGTAAGTGGTGGTGGTAACATCAATACGGGAAGTCAAGGATACGGTGGAGGTAATGCCAATACGAGAACTCAAGAATAAGGTGGTGAGAGCTGGACCCACTGAAAATTAATTTCGCCAGTTATCTATGTTTCATGTGTCTTATATAATGTTTAAGCTTTGTCTACTATCAACTATGTCCGTTTACTACGTGTGATGCAGTACAACATCATTTCTGCATTTTGTGGATTCCAAGTGGCTATTGTGTAATGGTTGCTTGCTTATTAATATAAGTTTGTGTTATATA

>ONT.5152.1 LG02 127525256-127527473

AAACTAATCAATTCGGCCCTGATTTGACTCAAAGTGTAAATATGACAATGGAGGAGAGGACCTTATAAACTGTGAAACAACAGCACTCAAAACAGCCACCCATTTCATTCGTTCTGTTCATGTTTCAGTTTATGTGGGCTGCTGAAAGGGAAACTCAAACTCATTCCTTCTTCCTTTTCTCCTTCTCTCTTCACCATCATTCAAGGAAGAACTTGCAGGTGGAGGAAAAGGCGGACTTTAGCTTTCTCATTCCAATTACCAGCCTTTCAACTCCATAAAACCTAAAGAACCCTCTTCCTATCTCTTCTAATTGCTAGATCAGACGCATATCCCAATCCCGGTCTTAAGGAGACCGCAGAGGAAGAGGAGGAGGCCGTTGAGGTACCTCCAGGCATAGAGGAATGGACGGCTGAGGGAGCTATCTAGCTTATGTCACTAGAGAGCGTCTCATCTTTCACAAATAATGTACTTTTGGCTTATAGAATACTTTTGTGTAGATGAACAAACTATACTTTTGGTATTGATGTATATAAATATTTTTGTATTATTGAGGTAGAGTACGTTTTAAATCTCTACTTCTTACTAGTAAATGAAATCATCTTTTAATTAATGAACT

>ONT.7784.14 LG04 86662021-86663306

CTTCGAACTTCTCATTTGCAACTTCAAACCCACTTGTAAAATATGATTGGTGTGGAAGGGAACAACTCCCAGGTCCAAGGGAATATCAATGCAGATACTGGAGGTAGCATCACTTTGGGAGTTCAAGGAGCAAACGTCAATCCGGGAAATCAATTAAGTGGTGGTGGTAACATCAATACCTAAAATCAAGAATAATGGTGGAGGTAACGCCAACATTAGAACTCAAGGATAGGGGTGGTGGCTGGACCTGCTGAAAATAAATGTCGCTAGTTATCTATGTTTCATGTGTCTTTTGTTATGTTTAAGCTTTGTCTACTGTCAACTATGTCCGTTTAATTACTACGTGTGATGCAGAACAACATCATTTCTGCATTTTGTGGATTCTGTAATAGTTGCTTACTTATTAATAAAAGTTTGTGTTATATA

>ONT.7976.7 LG05 7475142-7483613

TTCTCCTCCTCTGTATACGCTGTTCTGGTCACTTGCATTGTCGAAAAGTTTCGCCCCGATACAACATTATCAAGTGGCAAAAATCATTCCGGCTTCCGTCTCAGGCCACTGTGTTTCATCTGACGACAGATCGAAGCTTCCAGCAGGTTGCATAGAAGAATTTGAGTATATCAAATGGCCACAGGACTGGAAAATATTGGTGGAACTGAATTGGGGATCGAAAATGTGTTGTTGGTGCGACAGATTGAGCCTGGGAGGCTGAACTGTGACACCCTTCTCAACAGGTTAGCTGAGTTTAATACTTATGCAGTAACCTGCAGCCGAATGGATGATTGTCTGTTCCTTGTGTTTTGTGGTGGCACAGAAATCCGGATTGACTAGGGAGATGCCCTGTTTCCTGTCATTTCTGTCCACATTGATTGTCTGGGGTGGAACAGGGGAAAGAAAGTATTAGAAGTGGAACAGACAGCCATGGCCAATATGTTGAAACCCTAGATATCTGCGGGCATCATCGACATTGAGGCGGCAAGTTACCAGACGAGGCTCTTGTGAACTCGATATAGCGGTTCCATTCTGATCTGTAGTTGTTTCCAGTTTCCAAAATAATTAAAGTGTCTCTGTTTGGTGTACTCCTCTTCCCTGCAAAAATGTTATCTGCCAGGCATGTTTTTATATATCTGTAATTGCAGCTGATTTCCATGGCCTGAGGGCTCTTTTCTCAGGGGGGAAATTGACAGAATGAGAATTTATACTGGGTGGGCTGGAGATTGGAAGCACCGCCTAGACTCGAAGCACATGGCCGATAAGACGTGGCATGGCTGCATGGGGCCGCTTTTGCCCTGTACAGATTCGAAATGGAGACGTGTTGGCTTTGATATGATCTCAAATTGTTAGCCAATACACCCAAAATGCCAACGTGTAAAAGATGCAGAAAACTAACTACTTAACAGCCTTCAGTTTTTCAATTTTTTGGGTAAAGTAACATCCTTCAGTCAAACAAAATGACCCCGTAGGACAGTTGGTAGGTGCTTATTGTTGCACCTCTAGGGTTGTGGGTTTGGGTCATATTACAATTTAAGAGGTGTTCAAAATTATTTTTAATAATAAGAAATTCAGTTTTATTGGATCAA

>ONT.4729.1 LG02 73482298-73489547

CCACTAAACAAAAGTGACGCAACAAGTTTTCGTTCCTTTCCTCGAAAGTGAAAACTTCCCACCATTCTTTCTTCCGTTCCCTATTGTTATCCCTGCGCCTCCTCCATCTGCACCAAGAGACATGCGTGGTCTGGCCGGATTTTCTGTTTCTCCAAACTCTCCCGCTTCCTCTCCAACCTCTCAGAACTCCGACACTTCCTCAAAAACCCTTCCTCTCTCCCCCCTCACCACGCCCTTCCTCTCTTTCGTTGATTTGCTTTGTGGTTCTTAAATCTGATGGGTTTTTTTGGGGTTTCTGTGTTGGGTTTTGTTAATGTTTTGATTAAAAGTCTGGATTTAAGATTTGATTTAGGGTTGAGTATTTGGGTTTCTAAGGTGTAATGTGATTGCTTGACAGGGAGCCTCTCTCCCGCTTCTCTGCATCACCATCGCCCAGCAGCCTTCTCATCTCGTATAAGGGACCATAGCATGATTGAGAAACTAGCACAGTTTACTGATATCAGATGTTGCCTTGTGGTCGGTGGGCTTTCAATGAAGGTTTGGCTGGCACTGGACCCTCCATCGAACTGGTCAGAGCCTGAACTGTAACAAGGTGAATGAAGAACTTAATTAAACGGAGATGGAATGCCTTTTGCTATATGGATCTACTAATTATGTTAAGTTAATTTTGGTTAGTTAGGGATTTTCAGAAATTGTTTACAATGTAGCTTGTCTTTTTTTGGATGCTTTTGTGAAGGTTTTGGATTATGTCGTGACGCTTTGATTACGAATTCGAAGGTTAATGTAAATAGTTTGAATTATGGATGGATTCAAATTGGC

>ONT.5289.7 LG03 18468847-18470372

CCTGATTCCAGTTTGGCTGTTGCTCTGTGCACATTCTGTCCTGTTCATGGACACCGATCACTCATGTTCTGGACATTTGCTGGGGTGGTTCCATTACCATGACACTGGCTGTTAGCAAGCAAGAAGCCTGCAACCTGAACAATCTCTTCAGTTGTGAGTTGCAGAGACCACTGCCATGTGCTCAATCAGATCCAATACTCGGTTACTGAACCAAGCCACAGAATTTCATGTTAACAGAAACATTTCAAAGGGGAAAAGGAGGCATGAGAAAAAAAGGTACGTTTAGGCTTACCTGTATCCCCACTCATTGTCGTACCATGAAATAAGCTTCATGAAGGAAGCACTCAATCCTATCCCCGCTTTAGCATCAAAGATGCTAGACCTAGCAAGTAAAAGATAGTCATAAACAGGAGAGAAAGTAACTAGAATAATCCAGCATTTTCCAGGTCAACTCCAAAAGGATGATTTTTTGTTTTTACCTCGAGTCACCAATGAAATCATTAGAGACAACATCCTCATCGGTATATCCAAGGATGCCTTTCAATTGCCCCTCTGATGCAAACCTGATACAGAGTTTAGCAGGTCATTTCACAATGCAGCTTCAAGGGTCACAAGCACTAAATACACAAAAGTGAATTCATTTTAGCAACATCATCATCATTAGAGTCTAAGAAGCACGGACACGGACACGGGACACGGACACGACACGACACGGACACGCCGACACGGCATTTCTCAAAAAAGTAGGACACGGACACGTCGGGGACACGTTAATAATAATAATAATAATATATCAAATAATATACTAAAAGATACTATCATTTTAACTTTGTAATCAATTTAGCAATCAAATAAATAAAAAATCAAGTACCACTGTACCAACTAACAAACAAATCAAATTATCAAATAGCAATACAAAATTGAAACATAATTAATAATCATTAAACATGAACTATCCATAGTTCCATAAAATTCATACAAAAA

>ONT.3520.1 LG02 65432168-65439584

GCTATTAAGACGATATTGCCATCAGTCTCTGCTCTCTTCCTCATCGATCCCTCCCTCGCTCTCTCCCTGCTCACGCCATCTCTCCCTGCTCACGCCATAAGTCTCTGTTCTCTCCCTGCTCATGCATCGTTCTCTTTGCTCGACGCCGAAAGAGATCCCTCTCTTCCTCATCGTCGATCCCTCTCTTGCTCTCTCCCTACTCACGCCATCCCTCTCTGCTCTCTCCCTGCTCACGCATCCCTCTCTTCCTCATCGATTTTCCTTATCGTGGATATAAAGCAACAAAATCGTCCCCCCAAAAAATCTATCATCTAGGGTTTTCTCTCGAGCTCTTCGTCTTTCGTTTCAGGGAAGGAGGAAATGTTACCCACTCCTGAAGGTATTCAATCAAATGATGCTTTATTACCAAAGGATATATAGGCAATTCCATTCAGACCGCTCTCTCAAAGACGACTGACATTCTTCAGCTTGGGCATAGGGATAGTGGCAGCTCCTTGAGTTAGAGCAACTAAAGCCCCGGACATATCAACATCTTGGAGTTCACCCTTTTCTTGCATGTACTCAAGAGGTAGTAAAATTAGATCAAAGACGTGCAACTTATCCAAGGATTTGATTCAAGATATAGATCGGAATGGAGAAATAAAATTCATGTAGCCGACCCCAATATAGGTGGGACAAGTCTTTGATGATGGTGATGATGAAATAACTCTGAGGAAGTAGATCGTAGCTGCATCATTATAGCGTGTAAAAGATTATGGGCATAATTTTGGAACTATTATTATTGTTTTTATATTTTTAATTTGAATGGAATTTTGAAGTGAAGATTTTATAGTTTTCCTCCTAGGGATTACCTTGTTTATTTCGATCACAGACATTTGTGGATGTTAAAGATTTTGGGAAATGATCAAATAATAATATTTGTGTATTTTA

>ONT.10102.6 LG06 15931424-15947198

AAAAAAAAAAAAACCCAAAATCGTCTCTGGCTCTTTCAACCTTCCCTCTTCAGCTCTTCTCCCGGTCTACAGCTCTTCCGACCTAGTGACCTTCCTTCTTCCTTCGACGCTTCGACGGCTCAGGAGCAAGCCTCTACAGATATGGGATAAAGAAGAGCTGTGCTTTGCAGCGATTAGAAGCTCAGAGCTAATGTGAAGAGAAGAGCTTTAGTGGAATGCTGAGCTGCAATGTTGGGAAGTGTTTTGCTCTATAGTGGTTTCTCCCAGTGCGAAGGAAGTTAAAGAAGACTATATGGAATAACTTCTCTGTGCTAAGTTGTGTCAACACATTCACCCTTTTTGTTCGATGAAAAGCTACATCAAAATGTTGCAAACGATGGTCCAAAGAAAAGTCTTCTAGCAGAACTAGACGAAGGTGATGGTGTAGACACTTCTTATATATGTGGAGGAGGCTCAAAATAATATACTTATGCAAGAAAAGCAATTTGTTGCAAAGAATGCTACAAGTTAATTGGAGGGCTTTTCCACCACAACAATTGGAATCAGCTAGCAATAAACAACATTGGAGAAAGTTAAAGCCACAACAAATATGATGGGATAGACTTTTTGGTTGTTAATTTTTTTTCAGAAATGGAGTGGATGAAAGTAGCTGGAAGGTTATATGAGGTGGATGTGTGATGTATGGATATTCAAATGTTAAATATTTGAGTTCAAGTTAGTCTATTGATTTCAATTTT

>ONT.16143.2 LG11 22173523-22182188

ATTGTCTTGTGAAAAAGCTTCTAAAGGCGTTGGACTTGGATGAGCCTCTCACAGTGAGAGAAAAAAAAAAGAGCTTCTCTGAAAATGTGTTTGTGGGGTTTTGAATATTAGACAAATGTTCCTAAAAATCAGGTTCTGAATTTCTCTTTTCTGCAATATGTAGGTATATTGACGGTGAAGAATGTTGTACCTTGTTGGGTCTAAGACTACTACTTAATCTTGTCCTAGATCTCCTTCTTCGTCAACCCAGAGACCGGGGAGCTTTGCCCGTCACATCCTCACAGACCATCCGAGTACATGGCTGGCCTCTTAATCTTCTACATACCAACCTCTCTACATTCCTGTTCAGTCGAATCAGCAAACCTGTACAGCCCCACCGTCTAGCTGTACTATATGAAATTCACATGGACAAAAGAAGGGTGAATAGATCGAACAAAACCCCCTAATTCAACCATCTGGACTCTGCATTTTTTTTATAGGAGAGAAACTTCTCCCTCTTTTTCTTTCCCAGAAATTTGCTCAAGTAGTTGTTGGGTGCGAGATGGCCTTCTGCCACCCCTTGACACCAAAGAACATGACCAAGACCTCGTTCCTCTTCCTAACTGGTCTAACCATGTTCGTGGTCGGTGCCCACTACTCTTATGTCCACGTCGAACGCCAGCAGGCCCACACCAAAGCCCGCAGTGATCTCCTCAGAGAGCATCTGCTCAAGAAATATGGCTACAACAAGAAATGGGGCGACCTTGACAAATAGCAGTTTTCTTCTCTGGTTCTCTGCGGTTCGGCGACTCGAGGACTTCAAAGTCTGGGAGGCAGTTTGGAGCTTGCCTCACTTGAAGCATATTTCATCTACCTTGTAATTTTCACCCTTTCTGCATACGGGGATATCTTGTAAATTATGACATTTTAAGATTTATATATGCCATTTAGAGGCAATGAAATTTTTGGTTGTTGAGTTTTATTCATTGTATCTCCTTTTATCTATTTGGAACCACTATTATTTTCA

>ONT.13383.1 LG09 35187725-35195133

GCTCCTCTCTACCGTTTCTCCTCTCTATAGTTTCTCTCGGTCTCTGCTTCCACTACAGTTTCTTTCCCTCGTTTCGCCCGAGACGTTGCCGATCAGAACACCGCCGGAGAAGAGATCTACTGCAATCAATCCGAGATTCACTTCTCGTCAAGTGTCATCACCAAAACCAATATTTCAGAATCCTAAGCCAGCGAGCCTCCAACCCTTGCCATCTCTTGGTTGCCAAGCCCCCACTCCAATAAGCAAGGGGCGAGTACAGGTCAGAAATCAAGAACTGTAGTAGAGGGATTACGCCCAAAATTAAAGATTGATTTTGGATATTATTGTGCTGGTTGCGGGCTCTTAGGGATAACTTTTTTTATTTTGATCACAGACATTTGTATATGTTAAAGATTTTTGGAAATTATCAAGTAGTAATATTTATGTATTTTTAGAGATTTGTGGGTTAATTTTGGTTTATGGATGTAGGATATTTGGTTGTGAATGTTATTTTTGATTTGTTAATGAATCTATTTTAAATTGGGTTGTGCATC

>ONT.9545.1 LG05 42021592-42022694

GGCTGAACGTCCGCTCACCTCTTCCTCTCTCTCAATCTCGTGCGCAGCAGCTCTCTTTCTCACTCTCGATCGCGATTCTCCTCCCTCCCCATCGCTGTGTGTCCAAACCCACACCATGGAAGAGGCACTGCAAATCTCAGAGGGCTGAACGTCCATCATCTCTTCCTTTATCTCAATCTTGTGCGCAACAAGCTCTCTTCCTCTCTCTCGATCGCGATTTTCCTCCCTCCCCATCGCTGCACGTCCAAACCCACGGCTCCGCCTCCACCAGCAGCATCGAATTGTCTCCCTCTTACCATCGCAGCGCCTCCCTTCCTCCCTCATCGCAGCGGCATTCCTCCCCATCGCAGCCGCATCCCCCTCGCAGAAAGGTTACCTCAAAATTTATGTGCGGGAGGAATGATTGGGCACAGGCGATACTTCAAACTTTTGTGGATTTGAATTGTCCCATCGCTACTTCAAACATGTTATCTTATTTTCTTTTGAGAAGAGAGATTCTGGTCATTTACAACATAATAGGCTGCTGACTTGGTAGGAAAAAAATTTATTGATCTTGCTTCAGCCAAGGAACTTAGTTTCATTTCATCTATGTTGCATAGGGACAGGTACGAGCATCAGAATTCAATAGTGTGATGCTTGCCTAACCACTAAGACGAATAATATTGCTTGCATCATTTTGTTAATGCATCACAGAAACCTTGTGTTAT

>ONT.10102.1 LG06 15931415-15947197

GGGCAGAACAAAAAAAAAAAAACCCAAAATCGTCTCTGGCTCTTTCAACCTTCCCTCTTCAGCTCTTCTCCCGGTCTACAGCTCTTCCGACCTAGTGACCTTCCTTCTTCCTTCGACGCTTCGACGGCTCAGAGCTGTGCTTTGCAGCGATTAGAAGCTCAGAGCTAATGTGAAGAGAAGAGCTTTAGTGGAATGCTGAGCTGCAATGTTGGGAAGTGTTTTGCTCTATAGTGGTTTCTCCCAGTGCGAAGGAAGTTAAAGAAGACTATATGGAATAACTTCTCTGTGCTAAGTTGTGTCAACACATTCACCCTTTTTGTTCGATGAAAAGCTACATCAAAATGTTGCAAACGATGGTCCAAAGAAAAGTCTTCTAGCAGAACTAGACGAAGGTGATGGTGTAGACACTTCTTATATATGTGGAGGAGGCTCAAAATAATATACTTATGCAAGAAAAGCAATTTGTTGCAAAGAATGCTACAAGTTAATTGGAGGGCTTTTCCACCACAACAATTGGAATCAGCTAGCAATAAACAACATTGGAGAAAGTTAAAGCCACAACAAATATGATGGGATAGACTTTTTGGTTGTTAATTTTTTTTCAGAAATGGAGTGGATGAAAGTAGCTGGAAGGTTATATGAGGTGGATGTGTGATGTATGGATATTCAAATGTTAAATATTTGAGTTCAAGTTAGTCTATTGATTTCAATTT

>ONT.7698.1 LG04 78751374-78752929

CAAATAGCCTCAGTGAAAATTTTCCAGATTGCAAGTCTTTTGGGCACAACTGCCATTTCTGCTTAGATAAAGATATATCAAGAGTCTTTTTGAGTGCTTTGAGCTATGTCTGCTCTCTATATAATGTCAATGATCTGGGTTTCTTTCCTCTGCACCAACTTAAAAGCCTACAAATTTTTATGGGGGTTTTTGAATTTTCTTATATAAGGGAGTGGTCTTTTGTTGTGTTTCTTAGTAGTCTTTGCTTGCAACAATTCAAGAATAGTGGAGGAGTTTCGGGTCTTTTTTTTTTTTTAATCTTGGTAATGGTTGGATGCTTAAGAGCAAGGAAATTTCAAATGTTTTTTGGGTTCTTGAAGGTAGTTGAGATAATTGCAATTGTTTCTGGTGTTGGAGATATTAAGACTGGGAGATGGATTAGAGGAGAGAGGTGTTAGAGCAGAGAAATCTTAGGCGAAATTTGAGGAAAGTTGAATTTTGAATTTGAAAATTTGGAAGTATCTGATGTTGTAGGTGATCCTTTGAGTTTCTCAGAAGCGTCTACTTGCAACAATTCGAGAAAATTGTAAGTCTTTTTGGGTCTTGTTCTACTTTCTTTCGAACGTTTTTCTTTTGAGTGACTGAAGAAACTGCAAGTGTTTTGGGTTGTTCTACTTCCTAAAAGGTTATCCAGCAGCAAACTGCTGTTTTTGTTAGTAAAATCAAGCAACCCAAAAGACCCAAGTGAGTAACTGAAACACCAATTTATGTTTTGGAGTTGAAAATGTTGGATATGTCTCAAAATACCTTTAGTTGTTGTATACCTCCTCCTATGTGTTTCTTAGAAGTTTCTGTTCTCAAAAGTTAAAAGAAAACTGCATTATGTTCTGTGTCTCCTTTTCACTTTTTCTGGCTGCTTAAAAGGGAAGAAACTTTAAAAAGCTTTTCTGGGTTCTGTGAGTTTCTGCTCTTCCAGTTCTTATAGCTCTCTTCTAGCACCAAACTGCTGTTATTTCAAGTATTATGAAGCAACCCAATAGACCCCAATAACCAATATACCAACATCTTTCTGCATTTGGAGCATCCATTGAGACTTGCCCAAGATCTCTAAGCCCCTGCTGACCTGTCTAGATGGGGTGGGCTGGAGGACTAGCGTGGGTGAATTACAGCAAGAAGCATTGCAGAAACACTTTATGGAGGTTGAAAGCAAAGATGAAGAAGGCTGTCAAGAACGGAAGCAAGCAGAGGGTCACATTTGGGTATGATGCTTGGAGTTACGCACTAAATTTCGATGATGGGCGTTGTGGATTCAGAAATGAAACTGATGTGCTCCGCACACTTCATGCTCCAGACCTTACTTCAAACCAAAACTCTACATGGGTTCTTGTTCTCTCTGTGAAATCTCTCTAGATGTGAACTTCTTTCTAGATCACTTCAATTTGGGTTTATCATGAACTTCTTTCTTGATTAGTTTATTGATTTGCCTATTCTATTTCCAGTTATAATGATTAGAGTGTGTTATTTCTCT

>ONT.8911.3 LG05 513806-517330

GGCATTGGAACGAGTCCGTCCGACTTCTGAACCCTTGGAACGAGTTTGCAGAGCTTCTCTCCATCTCTCGGTCTCTCGTCATCTCCATTTGAGTAGAGCCTCTCACTCTCTTTCTCTCCTCCTCTCTTCATCTCGATCTCAGGCAAGATCTTTTGATACTATTAACTTCTGCTGTTACAACAGTGCGTGTTTGATGGTATTGTTACCAGTATCTCATCAAAGATGAAGTTCATGATTTATATCAAGGAGATATTGATCGGACAACCCCATAAATGTCATAACGTATTTAGAATGAGCATTGGTTTTTTCATTGTATTAGCTAGTGAGCTAAGGTGTACGGACCTTCTAAGAGACCACTGTACGGTGAGTGTGGAAGAGTAGTTACTTGTCCATATTTTTATTAACCGTTGCCCACAGTGAAAGAAATTGAATAATGGAGAACTAGTTTCAACATTCAAGGAAAACAGCCAATTTCGACTCGACATTGGATGCAATAATAAAACTCGCTTCGTATTATGTGAAGAAATTAGAAGGAGAGTGTCCACCTGAGGTTGTGAATATTCCTTTGTTCTACCGTTTTTCAAGGATTGTGTTGGAGCACTAGATGAGGCACGTATCCCTGCATGAGTTAGACATGAAGATCAAGTCTGTTGTCACAATAGAGATGGTTGATTATCACAAAACGTGATGGTCATTGTATCATTTGATATGCGTTTCTCATATGTTTTTGCTAGATGAGTGGGATCAGCTGCGGATTCTAGGGTTCTTCAGGATTCAGTTTGGAGATGGCCCCGGAACAGGTTATGTGTTCTAACCGGTGCCTAGAACTAAAGACGACCTGTTCAACTTGCTACACAACTTTATCCAACATTGGAATACTGAGGACAAATTATTCCGAGAGGCATTGAATGAGATAATGGAAGAATATGCTTTGGTCGATGAACGAGATCACAATGAAGTAGAGGACAGTGTGTTGGTCCTAATGATGCGGATTGGCAATTCTTGACCAATTTGAGAGAACAATTGGCAAAAGATATGTGGGAAGCTAGGGGGAGGGAGTGCTTGATATGGTCTTACTTACATCAATGTTTGTAGTGTTTATATTTATGTTGTCTATTATTGATGATCTTTTGAGAATAGTTAACAATGATTAATGGACTAGTTGTGATGTCTAACGTGTGAT

>ONT.5098.2 LG02 121499348-121507803

ACCGTAAACTCTCACAGCGATCTCTCCATTTCATTTGATCGGCTGCGAAATCGCACAGGAGCTTCCAATTCTCGAATGCTCGATCTCATCAACGATCCAAACTAGAAAGAGCAATTTGCTTAGAAGTTAGGGTTTTCGAACGAATTGAGGAGAGAGAGCAAGAAGATAGGGTTTTTAATTTTGAAGCTGATCCGTGGAAGAAATCTGCTGAGAGTTTGAATTGGAGCCGATCAATAGTTCCAGCCCTTAAGTTGCTGTTTCATCTTCATTGCTGATTCTTAGAGTTCCAGCCCTTAAGTTGCTATTTCAGACGTACTTAGGTGGCATCCCTTTTCTTGTATCAGGAGTTTGACTGGGAGGCAACTGTCAAAGAAATTGATTTTGTCTGTCAGGTTGTGGCCTTGACCTCAAATGGAGAGAATTTTAGGAAGTCCAAGTCTTCTAAGCCCAAGACATTTGTTGGTTCCAGGCAATCGACTCTTGATAAGTTTGTCAGAACAGGGGGAAGATCTAGCATCCCTCATGGGAATAGTTTTGATATGCGTGACAACGGTATCTGTCCAAGAATTGACTTTTTCATCATCTTGTATAGTAGACAGGTGACATACTAGAGAAGTGAGGGATTTTATTGCTATATGTGCACATCAGTTGGAACTAAAATTTGTAAGAGTTGTGATGCCAGGTACTTCACCTATGCCCATGTTTTCTTTGTTCATTCCAAAAGGAAGTTCTCAA

>ONT.10136.1 LG06 32299590-32302688

ACCCATTTTCCCTGCAACGGTTCCAGCAAGAGCTGGAACCACTCCTCTCTCAGCCACTCTCTCTCTCTCTCTCTCTCCTCTCTCCCTTGGCTTCTCTTGATTGTAAATGGAGATTTGATCTCCTCATCATCTTCCATACCTTCCAAGCTTTCCAAAGATGTTATTTTCATGGATTTTGAGCAAAGGCAACTTCTCTTTTCAAGCTCTTCTTCTTGGTTCAAGGCTTTAAAGGGTTTTCAAGGGAAAATTTTGGGGGTTTTGGTGGGTTTGGAGCTAGAGACTTCTTTGAGGCTTTGGTGGAGTGATTTGTGCATCTTCTTATCTAAGGCAAGTTCAAATTTCCTTGTCCCGTGTTGAGTGCTTTGGTTGGCTTTGGAGCCATGTCTTGCAGGTTGTTTTGTCGGCTATGTCATGTAGACATCGAGCTTGATATCGGAGCTACGTCACATTTGGGTAGTAGTTATTAGCTTATTGTTGTATGGCTTTTGTTAGCCTTCTCATGGGTTTCTTAATTAAGGTACATTGTTAATTTGTAACTTGAACACATTTGGGAATGAAATATTCTATTACTGTCA

>ONT.6536.1 LG04 8892207-8895473

GCATTCTGCTTTCCTTTATCAAACCTTATCTCCGCAGCGCAGAGACCATCGTCTGCATCGCTCTCCCGATCTCGTCCCATGGTCATGGCTGCAGAGAGAACTCTCATCTCTCCTGTCTCGTATCTTTGTTTCTGTTTTTGCAGACAGAGTGAAGAGATCTTTCTCTCTTCTTCTCATTCTCTCTTACCTCCTCAGTCTCCTATCTCAAGAGATAGACTGCTTTTTAATATCTGCTCCAAGAAAGAGGAGGACCCAAAACACATCTGGATCATTGCACCACACCATCTATGAGATTCAGCAGCAGCAGCAGTCTGTTTTGCAGCGCTCACATTCTCCTTATCAGTATGACACGGTGCAAGGAGAAAAAAAATTGTAAAAAAAATTGTGAGATCAAACTGTAGTTTTTTCACTTACTGGATTTCTTCTTTCCAGATCCAGCAGTAGCAGCCGCCGCAGCCTGAGCCTGCTCTTTCACAGCCCTGGCTGCTTTTTTTTTCTCAGTTTTTTCTTTCTTCTGTGCTTCTGTATGGCATTCTATAAGATGCTGTACGGGCCTTTCCCCCTTAGGTTGGCTGCAGTGAGAACACTTGGAATTCATTCTGTAAATTAAGAAAAAGAAACTAATTTTAGACTGGGCATGGGATTTAGAGAGAGGTGGGGCAAGGGTCGGAGTGGGCCATTTGTGGATTAGAGGGTCCAGCAGATATAGGATTTAGATTCTGATTCGACATTCTGTTTAATCTAGATTTTCATTGAGACTTAGATTCTGATTCGACATTCTGTTTAATCTAGATTTTCATTGAGAC

>ONT.488.1 Contig01434 23150-24008

GTCTCATTGATGGCATGTCTCAACCTTCCTGTGAGAGTGACTATCATTACTTCCTCACAATGGAAGTCCAACTGTCGTATATTTCTCCCCATCTCAGATTGTAGATGAGTCGATGACCAAAGTAAGAAGAAGCTTCATCAGATGACTTGGGATGCAAACGGGTCAATTGGGGATGGAAACATGGTGGATCCGGACGTGAGCCCATTTTTATAATCTGGATCTTGTTCCCACATGGAACCTATTTTTCGTTTTGGATATTGCCCCTGCAACGATCTTTGGACCTTCATCCATAAACGGTTAAATTACTCTATGCAGAGCCACAAGATTGAAAAATAGGATCAGAACACTTACCGGCCCACTTCAACTATTACGAATTGGAGCTTTTCTCGGATGAAGATAGGACGACCAATTGAGCGGAGTTGGGATCAGATGGAGTTTGCTTTTCCACTATGTCAGATGATAATTTGTTGATGACGACATCTTCTACTTGAGAACAACTTGATAAGCAAGACATAGATAATGTGAGTTATTATAATTGAAACAAGTAAATTAAATTTGGATACATTCGGA

>ONT.14215.6 LG10 488223-492877

AGGCGAAGTTCCTCATCCTTCACTCCTCCATTCTCTTTCTCAAATCTTCAGAACTAGGACTTGCACGTGTGTTGAGAGTGTAGATTGGGACTTCCTTATCATCTTCCCTGACTTTCAAAACAATCGTGGAGCAAGAGTCTTCTTCATCAACTATCTATTTCATATAAAAGGGTTGTTGACAGACACGTTGTGATTGGTGCCTATACACGGGAGTCCAATGGCCACTGTTGGGGTTGCAAAAGACTGGCTCAAGTATTTGAATAGATCTGTATTTGAAGGGCGAGCTATGTTGAGAAGGTTATCTGTGTGTCGAGAAGCTCTTCATTGAGCTTCAAAAGCCTTATCACAACCACCTAAACAAGAAAATCACTCTTTCAAGCAAATATCAGACTATTTGACCAAAGGGTTGTATGCCCTGCATCTCTTTCTTTTGAGTAATCGGAATATTTTTTATTTACTTTTTTTTTCCTGTTGTTTGTTTGCCTACTGGTCTGGACGTTCCTACTGTGTATGGTACGGTGCTGAAATTGAAAACATGCTACGTGCAATGCTATTCTGATGATTCTGCCTCTTCATCCTCCAATCTGTATTGGCGATGTAACAAAGTTTTGTTCTCTCACCATCGCTATAATCCGAGTCACACCCCATGATCTTTGCTCACCTTATAATTATAGATTTTTGTGCCACTTAAAGATGCATGCGGAACAGGGTCCATCCCTTCAGCCTGAAATGTTTAAACTGTCATGGTTTGGGGGTGCATTCAAATGCTCTCCACATTCAATATTCTTGGAGTTTTGAATGATCTACCTCTTCCTTTTTAGAGCTGCAGTAGTTTGTTTCCGAAGTTTTATTAAGGAATTATGCTATTTCTTTTGCAGTCGAGGAGGCAAAGAGGACGGACACCAACACCTGGCTGGTACTGTGGCCTGATAACGATCCCTGCTATGGGATCACACACCTATAACCCTAGTAGAACTTTGGGAAGTTGCTTTTTGGCAGCCACTCCGGAGTCCATCAAATTACTCTCCTTGTGGCAGGATACATTATTATTATCCCTGCTTCTATGAAAGGGATCAATCCTACTCTCTGTAATACAGCAAGTGCAGTGTTTCTCCAAGAGTGAGAAGAAGCTTGAGGAGGAGCTTGTCTTGCAGCATTTCTCTGAGGCCAAGGGAGCAATAGCTTTTCTCACAATCTTTCTTCCAGTTTCTAGAGCTATTCTCAGAATTCAGTGTATTTCTCCCTGACCGAGGAGGTGTTATCACAGAGCATTTCTCATAGACCGAGGAGGAGCCCTTGGAGCACGGTTTCTGCTAGGCTGGAGGAGTCATTCCAGCAGCTGCAGTCATAGTGTGAGCTTGGGTTCTCGACCTCTATGGCTCTATCACTTGTATTCACCTTAAGGTTCAGCCATCTAGCAGTGGATATTCGAGGTTTCCTTTGCCTTGTGGTGGATTAATGATCATTGAATGCCAACTTTGGTCACCGTTCT

>ONT.641.5 Contig01843 19681-38693

AGCCAGCTTCGAACTTCTAACTGCTCAATTGCAACTGCAAGACCACTTGTAAAATATGGATTCCAAGAAGGTAACACCACTCGGCGCAATATCCAGATTGGTGGGGGAGGGAACAACCCCCAGGTCCAAGGGAATATTAGTGCAGGTACTGGAGGTAACATCAATTTAGGAGTTCAAAGAGCAAATGTCAATTCGTGAAATCAAGTAAGAGGTGGTGGTAACATCAATACGGGAAATCAAGGATATGGTGGAGCAAACGTCAATTCGGGAAATCAAGTAAGTGGTGGTGGTAACATCAATACGGGAAGTCAAGGATACGGTGGAGGTAATGCCAATACGAGAACTCAAGAATAAGGTGGTGAGAGCTGGACCCACTGAAAATTAATTTCGCCAGTTATCTATGTTTCATGTGTCTTATATAATGTTTAAGCTTTGTCTACTATCAACTATGTCCGTTTACTACGTGTGATGCAGTACAACATCATTTCTGCATTTTGTGGATTCCAAGTGGCTATTGTGTAATGGTTGCTTGCTTATTAATATAAGTTTGTGTTATATATA

>ONT.15069.1 LG10 76036900-76050943

CCTCTGTTTCTTCCTTAAACAGTAGCCAACACAAGTCTTGCTCAACTTTGCCCAATTGCTCTAGCTCTGTTTGTATCTGTTTCCCATACTTCAGATCCCATACAAAAGATCTCTCACACAATGGTGCAAAAACAGCATACACCAGCAAGCTCATACCCCATCTTTGCCATCATCTAATCAGATTGTCATGTCAACTTGGGAGCACTGTATTCTAAAGATAGGCTATGCAATGAGTTGGTGTGATGGTGGAAAACCAATACCTGTAATATATCAATGTGTGGGTGTTAACAGAGGCTGGCAACTTACAAGTCTGTTATACACACCATGTGAAACATGAAGTGTATTTACTATGTGGGATGGAGGATGAGCTACATAAACCTGCAATAGTATGCGCGTGCAACGACTCCATTGTCCGCCTTTATGACCTCCCAACATTTGATGAAAGGGGAAGAATTTTCTGTAATGAGGATGTAAGGATGATTCAGACCAGTCCAGGGGGGCTATTATTCGCAGGTGATGCAAGGGGCGGATTAAAAGTCGGGAAGATGCTAATGAAGTCAGGAACTGCTTGAATGTCCAAATGCAATATAAACTTATATTGATTATGTAAATTACAATGTATTCCACTTCAATTCTTTCCCATTGTTTTAAAGAAATTTTTTTTCCATTATATTGATTATGTACATTACATTAATGTATTCCCTCTTGGATGGTCTCTCTCTAATCATAGAGGGGCCTTTCATAAGATTGATCATGTATAAAGCAAATGTTTTTTTTTTAATGATTTATTCAAGTCTATTCTTCTGAT

>ONT.7172.2 LG04 2762687-2766143

GGGCTTTCCCTATCCTTGGTTTTGCCATAATTCTGCATCTGCAACAACCGGAGAGAGAGAGAAAGAAAGAATGGCGAAATTCGATCCATGGCCGATCTTCTTCAAGCGCGAGTGGAAGCGCAACTGGCCTTTTCTGGTGGGTTTTGCCACCACTGGCACCATCGTCACAAAACTCCACCTCAGCCTCACAGGTACGCACGCGTAATAATTTGAAACCATCTTTTATTATTTGGCAGGTGAATTCGAAAATGATGACTGATTCAGATGCTGGATCAACTCTCTTTATTTTGTTTTATGTGCTTGGAACCTGAATTAGTGACTCTTTTTTTTTTTTGGTTTATATCTTGGGCTTCAAAAACAATTGAAATGGGTGTTTTACTTTATTTCGTTATCTTCTATGTGGTTCTTAGTTGAATAAAAATGATGAACTAGCTATGTCCAAGCTTATTTGAACTTCCTTGGAATGAATATGTGAAAGCTTGCGCTGACAATAAGC

>ONT.4106.1 LG02 1698041-1705496

TCCTAAGTCCTAACCGGAAACCTTTCTCGCTGGCTGCCCCCTCTCATCGATCCACCGCCGGACCTCGAGCCATCGATCGCCGAAGCCGTCTTGTCTCTCAGCCCTAAGGATATCTTCTCACACACCATCAAAGGCCTCAGTATTGAGGTACCGTAAGAGTGAAGGCACAAGAATGGCTTTTGTGATGAGGTGAAGTCTTTGAAGAGTCTTCATGAGTTCCAGAAACAGTTAGGCCAACCAGATGGATTTCCATGGTACACATAGAAACCATGGTGTTGGCCAATCAAGTGAGACATTAACATATGTCAGAAAAGAGAAATCTGGAACACTCCTTAGTGAATACATTGCTTTTGAGGGCAATTCCTGGGCTGAATGAATTTGGCAAGGCTTTATTAGGTGAATGAAGAACTTAATTAAACGGAGATGGAATGCCTTTTGCTAAGCGGATATACTAATTATGTTAAGTTAATTTTGGCTAGTTAGGGATTTCCGGAAATTGTTTACAATGTAGCTTGCCTTTTTTTGGATGCTTTTGTGAAGGTTTTGGATTATGTCGTGACGCTTTGGTTACGAATTCGAAGGTTAATGTAAATAGTTTGGATTATGGATGGATTCAAATTGGCTATGTTTTGAATAGTTTGTTTGATTTAGTGCGTGGACGAGCAAGTACGTTCTTTAATGTGAATGGTGATTAATTTCTAATTAATGAAATGGTTCTTATTGAATGTTGCTATCT

>ONT.4637.2 LG02 40210285-40212969

CTCTAATCAAACCCGTCCCTTCTCTCTCTCTCCTTCATCCTTCTCCTCGACCGCACCAGACCACAGAATTTAGCGCATCACATCGATATGGGGTCTGGGGTTTGCAAGGAATGACTTCTCCAATTTACCAAATTTACCAAACAAACGTTGTGGAGATGCTTTCTTTTGTTGCTACTTGTGGTGTATTAAAGTGAGGTTTCTATTACTGGATTGAGTTTGTGAGGGGCGGTTTGCTTCATGTTACTCCTTAATAAAGCTGCAAAACAGCCAAGGGAGAAAAGCTTTATCATGCCTGAAATGGAAGAGGACATGATGGAAGGGATGTGGGGAGTGCACACTATGGGGTTGCCAAAGGAAAGCACACCGTAGGGTGCAAATGTTATACACTTTAAAGTATAAGCCAAGCGAGATAGAAAGATATAAATGAGGTTGATTGCGAAAGGATTTACCTAGCCTTATGGTGGGACTTATCAAGAAACATTTGTATGATTTGCAAAGTTCAAGTCGATACGAGTTCTATTGCCCCTTGTTGCTATATATTCTTGGTCTTTACATCAATTGAATGTAAACATTTTTCCCCTTCTTCCTTTTAAGTATGTGGGTACTTTGTGTTGTGCAAGTTGAAAAAGTCCTGTATGGTCTCCAATAATCTCCCAATCTTTGTTTGAGATGTCTAATAGTGCCTTGCATGCGCAAAA

>ONT.8562.1 LG05 53865725-53869632

CCATTTCCCTACAACGGTTCCAGCAAGAGCTGGAACCACTCCTCTCTCAGCCACTCTCTCTCTCCTCTCTCCCTTGGCTTCTCTTGATTGTAAATGGAGATTTGATCTCCTCATCATCTTCCATACCTTCCAAGCTTTCCAAAGATGTTATTTTCATGGATTTTGAGCAAAGGCAACTTCTCTTTTCAAGCTCTTCTTCTTGGTTCAAGGCTTTAAAGGGTTTTCAAGGGAAAATTTTGGGGGTTTTGGTGGGTTTGGAGCTAAAGACTTCTTTGAGGCTTTTGTGGAGTGATTTGTGCATCTTCTTATCTAAGGTTCAGATTTCCCGTCCCGAGATAAGTGCTTAGGCTGGCTGTGGAGCCATGTCTTGCAGGTTGTTTTGTCAGCTATGTCATGTAGACATTGGACTCGACATCGGAGTTACGTCACATTTGGGTAGTAGTTTATAGCTTATTGTTGTATGGCTTTTGTTAGCCTTCTCTTGGTTTTCTTAATTAATGTATATTGTTAATTTGTAACTTGAACACATTTGGGAATGAAATATCCTGTTACT

>ONT.10111.1 LG06 18632645-18638209

GAGGAGCCCTTGCCAAATGAGAGATGTGAGCGCATCTTCTTCTTCCCCTCATCTGAAGCTCTCTTTAGACCCTATTTTGTCCAAAATAAAGATGGGCAGCCCCTCCTACAAGCTCCTTCTTGCTGGTTGTTGGTCTTAGAGGTAGCAGGTTCTTCGCCTGAGCTGAGGGAGTGTGTTGGCTGTGGTGCCATGAGTTTGCAGGTTGTAGTGTCGGTTATGTCATGTAGACATTGAGCTTGACAACGGAGCTACGTCATATTTGGGACTCCTCAGGTGAGTACTATTACGAGGAGCAATCAGGTGAGGAGATCCCAGAGGATCTCCAACCGGTTGAGGAGGAACGAGAGTGGCGTGCAGATGACGTAGAGTAGCGTCATCATACCTGGCACGAGAGAGATTCATGGGAACTTCACTAGATTCTTGATTCTTTATTTATCTCTCGTTGAAACTTTCGTATCGTTGACTTCTTATTTAATTTAAATGGCCTTGATTAGTGGAAAAGCCATTGTAATAAATTAATTTCATGGTTTAAGTTTTATTTCGTTTCTTGTGCAACTTTAACTATTAATGAGATTTATTATTCATTGCTTCGAAA

>ONT.14758.7 LG10 26123145-26125130

AGAAGAGAATGGAGAAGAAGAGGGTCAAGAAGAGGCCATTGAGGAAGAAGTGGATGATTCTACCGATGGCGCTGTCCTTGTAAACGGCAACGAGGCTGAAGAAGAGTGGGGTACGAATAATGAAGGAACCCCGTCAGCCTAAAAGCAATTGGTTGGGCAAGTTGCTTGCGCCAAGCCATTAGACCCTGCCAAGGCAGCTGGTTCCAGCAGTAATACAGGTATAACTAAAGAGGAGGCAGCGGCCAGGGAGCGGCTGCAATATCCCTATTATTTTTTCAGGAACTGAGTTTTGTCATTGTGTAAGAAAGATCAGAGACCCCATTGATTGTTTCATATTCTTGAGAGCAGCTTTCTGTTGTGTACGCCATTCCAACATAGTATAGTTTTAGCAAAAGAGAAGAAGAAGATGGTCATTGTCGGTGTTCTCTCCAATTTTACATTTTTCTTACTAAATGGTTGTTAAGCTGTTTCTTTCTTCTTC

>ONT.17972.1 LG12 51633969-51636260

TTATTCCAAATGTACACTTTAATGTTCAAGGGGGAATAAACAGAAGAACTAGTATCAAACAACTTTGATGAAGCCATTGACCGATTGAGTTTTTGAGGCCCAGTCTTCTGTAAGTGGGAGCAGGAATGGTGGGAAAGAATTTGATGGCATGGAGTATTCAAGCAATGAAACACCGATTCTCAAACTGTCAATAAGTGGAAATGCCTAGGGAAGTTGGAGAAAGATCAGTTCCTAGATTAAGAGTGGCACCTAGGAAAGGAAGTTCCTCTGCTAGCACAACCTACTCCATATCATTAAAGCTGTAGTGCTAAGGCTAGTTTTGAGAGATTCTTCTAATTTGAGGTGGAAAGATTTGGTCATTGGGCAGGGGAGGCATCCACTTACGTTGGCATGCTGGAGGCTGAACCGAGGTGAACCATATGGTATGGTATTATAAGGAAGCTCAAGCATATGCCCCTTACCTCAGGCGCTTGCTTAAACATGAGAGAGATGCTTAAAGGCAGAGAGGTAAGATTATCCAAATGCTAGATCCTCCTTTATGCATAAACACTGCTGATGTACTTGCACTTGGTTTATATGCATAAAACACTGATGTACTTTGTGGGATCGATTTTCACATGGGAGAGGATAAGAAACTGTCTATTTTGAAGCTGGAAAAGTTTTGTTGTATGTGAACATTGGAAAAATGATGCATGCATCCTTAAATTTTAATCGCTCTT

>ONT.5831.7 LG03 3776368-3780590

AAAAAGATGGAGAAGAATGTGATGGAGAAGAAGAGGAGAAAGACCAGGGAGAGAAGAAGAATAAAGAAGCATCCCCAGCTGGCAGGCATTGGGAATGCTTTTTGGTGGGATTCAGATGGATTGATTGATTTTCTCCTCTTTGGAACTCATTGCTGTAGGGAGAACTGTAAGGAATCGCAACTGAATCAGAAAGAGAGTTTTCAGAAGTAGGCAAAGTTTGTTGCATAATACGGTCTACACCAAATTGGGTTTGAGACAAATTGCTGTTCCAAAGATTTGATTCAGGACAATTTTGATTTACTGATGCATCATAAGGCCTAGGAATCGGTAGTGAAGGATCTTCAACAACATTGTTGTAATCAGATCGGGTTTGAAGCGGATTTTTCGGAGTGCTGGTCGAAGGACTCGAGTTACCGAATTTTGAGTTGGTTTCATCTGGTTGAGATCCCCGCTTCCTTTCGATAGCCCTGTTTGTTAGGGATCGGATGGATACATCTGTATGTCCTGGAAAAACACTTTCTTTATTGTATATCTTACACAGGACGTAAGGATCACCCTGCAATGCAAAACGGAAGAATTTCATCAGTTCATAT

>ONT.16026.1 LG10 81056366-81058383

TCTCCCTTTCATTTGCTTCCTTCTCCAATTTTCTTCTTCCTCTTCACTAAGAAGTTGTGGCTTGACCGATTGAAGACAAAGGTTGGAGTTCATCTTCATCTTCTCCATTCTTCATCTTCATCCACTAAGTTGACAAGAAACCATCTCCATATCTCCCTACTCTTTTCCCCAAATTCGGATTTCCTCTTGAGACGCAGATGTAGCTCCTGGTCCTAAGGAGGCCCCGGAGAGAGAGGAGGAGACCGTTGAGGTTCCTCCAGGATTAGAAGTGTGGCCTGGGAATGGCCCTATGTAATCCGGTCCACGGAGACCGATGGACTTAATTCTTTTGAGAGCTGACTCGAGAATGTGACTTACTCCTTTTTGTGTGGGTTACATTAATTAGATCATATGTTAGAGATGATCACTCTTTTGTAATAGAATGTGGGGTGTTTTTTTTTGTAATCTCTATTGTAGTTAATGTGTAAATATGTTTTGTATAAACTTGTCACCTTAACTTTAAATAGGATTGTGCAATGAAATGTACATAGTGGCTTATTATTC

>ONT.10874.1 LG06 64158600-64167078

CTCTCGCCCAACTCCCCAGCTCCCACGCTCCACTCTGCGTTCTCTTTCGCCTCTCACTCAGCTCTGCCGCTTCTGCTCTCTTCTCCACTCTGCTCTCAGTGTTCTTCATCTCTCGCTCAGCTCTGTACTCTCTACTCTCTGCTCTGCTCTCACCGTCGCGGCGAGCCCTCGCACTACTGTCGCACGCAGCGTCTCCCTCTCTCACCCTCTTTCTCTCTTGCCCTAGCCCTCTCTCACTCTTCCTCCCTCGCCCAGTCGGCAGTCGCCCCCTGCCTTCCCCCTCTCTCACCCTCACTCTCACCCTCCTTCCATCTCCCATGTTCTCTATCTCTCTCTATTTCTAATCCCGTACTTCCTCTCCCGCTTCCTCTCCCCATCTCTCAGTCTATGCTCAGAGATACCGCTCAAGGTTTTTTTCTTTGTGAGGAAATCTTAGAGTCAATCGAGTGGCAGACATCCATAACTTCGGGATTGTGGGATACTAAGATACAGTGGAGACGTGGAGTCCAACAATGGAAGGTGCTCTCAACTTGAATGTGGAAGAGTGCTTCAAAACGAGAATGGAGAGGTGTCTGTTTTGTTTTCGAAGTCCATCGGAGTTTTTTACCAAATAAAAAAGGTCCATCGGAGTTGAAGATTCTAATGAGGCAGAGGTGCCTGCCATTCTAGAAGCTTTGGGATTATATCCCAATTCGTTCTGAGAAAGCTTAGTCGTGGAGATCGATTCAGTCAATGCTATCTCCTGGGCATTGTGTAAAGATGCGACTCCTTGATCCGCTACTATCTAAATGATGTCAGATACCTTCTTATATGATCCAGGTGTGATTTTTAATCATGTATATAGTTTGGCTAATGAGCTGACTGCTTCTTTAGCCAAGCAAGGAGTGGATGGAATGGCACCCCGTGATGGGTCTTAATGTTTGTTTGTTTCTGTTTTTGGTGAGTGGTGTTACAGTCTCCAGTATACTACTATACTCCAACCTAACCATGGGGTTGTTTCTGTATGTACATGTGTATAGAACTTTAGTATGCACTCCAGTCCCATTACTCTTTGGCTTTAACCAACAACCTTGTGGTTT

>ONT.5010.17 LG02 116694315-116695142

CACATCTTTCTAGGGTTCCTCGTTGCCTTTTCTTTCTTGTTTCTGTTTTTCTTAGATCTGCTCGTTTTCTTTCGTCTTTCGGCTGGTATGCAGTCTATTTCGTATTGATCTATCGCCGATCTACCTATTGATAGTCGATTTTCCTTCTGATTTGCACTTAGCCCTTTGTTTATTGGTTGTTTTGCAGCAGATCTAAGTCTGACGGATCAAGACTCATAAGGCGATGGAGGGGATTCCAGTTTCGCAGATCGTAGTCTCTTATAGCAGTAAAGAGACTCCTTTCGCAAGATCCGTTGCATTTTGGCGTTCCTCGTCGACGATTTCATCGGATCCATGGGTTTTTTTAGCGATCTTTCATCAGATCTACTATGTATGGATGTAAAGAAAAGTAGTTTAGTGTGAAGATCGAGGAAAAGCTTGTTATGTATGAAGCTGATAGAGAAATAGGTATGGATTAAGCGAGAGAGAGGCTTGTGGCAATGGATCTATGTCATCCTCCGACCTTTGCCATGTTGGGTGCGCTCGCATGGCAGGTCAAAAAAGCCTAATAAAATTCTCGATTTTTGGTTCT

>ONT.692.1 Contig01934 6600-22135

GAAGGCCATGGAGGTGGTACCAAAGGAGCAGAAGGAAGAAAAGGCAACCATTGAAGCACCAAAAGAAGAGAAGGTAGGGACTGCAGTAGAAACCAAGACAGGCGTTTCCTTCCCACCAAACTAGATGATGGGAAGCTGCTTTATTCCGTGGGATTGAGGAAGAAGAGTGTTCTTGGACTCGGTATTAAGATATATGGCTTCGTCCCTAAACAATAGGCAGCCTTTTTCAATTCACCCGAATTAATCCAATGGTGTTGGTTGGCCTAACCATCCCGATGAACCAGGTATACTTACAAGGCTAGACTTCAAAAAGTCATCCCATCTTTCTTGAGAAACTCTAAATTCAACACCAATGCTTCCTATCCAAAATCTTGATAAATGTATACGTGTTTCAGTGAAAACCAATGTTTAGTACTATTTTGATGTATTTTAGAAAAGATGAACCTTCATTTTA

>ONT.12805.6 LG08 3431945-3446810

GACTCGAGAGGCAGAAGCACAGAGGTGTTGGTCTTCGTGCACCTACCACTCAAAATAATGCGAGGTGCCTGGGAAACTATCAACGGAGCTTGAGCTCTACTACAGTCAGGCGCTTTTTACTTTCACAAGTAATTGGGCTTTTTTCTGCCCAAACAGGGTACCCTACCCATGTGAGCAACTGAAAAGATTCATTCAAATTTTTACTTCAATCATAAGCAGAGGTTAAAATAAGTCTTGCAAACCTTTTTCAATTACTTCCTTCTAGCTCTAGCTTGACTGGCTCTCATATACACCACAACTCATATCCGATTCATACTATCTCTCACAAACGGTTTGGGAAAACAGCAATCCGAATCAAAGTTCTGAGTATCTCCTCTTTGCTAAATTCTGATTAGGTGTGGATTGCTGGAGTTTGATAAAGAGTTATAGGGATATTGGCAACTTCTTAATGGTGTGAATAGTTGGAGTCAGATAAAGAATAGTAGGGATCTTTGCACCTTCTTATTGGTCTGGGCAATTCATGGAGTTTAAAAACAAACAAAACATTCCTTCATGATGTTGTTTGAACTCATGGTTGTGTTTTTTGAGTCGCTTTATTTGGCTTTGGTTTAACTATGAATTTACTGGTGTAGTTGTACTGTGTGGATCTTGCTTGTCATCTTTGGTTTGTTCTTATTCACTTGTTTGGAA

>ONT.1209.4 LG01 28813131-28813967

AAAGGCTTTCGCTCAGATCAGCTATTATAGTCATGATCTTGAGACGGGGAGCGGGCCTTCCCGAACTAAGTTGTGGTTCACTACCCGGTTTGGCAAGAAGAAAATTGCTTGGGTGGATTCGAGTTCTCATGAAGTCTATGACGAGCTTAAGAAGCTAGAGTCTCAACCGTTAGAGGAGAGCGATCAGCATATATACGTGGTCGTGGGGCTGGTCCAAAGGCCACAACATGGACAGCTGGGCAGCGGATCTGTGTGCAACTAGAAAGGGAGAATGAGGAGTTTAGGAGGCGAGCTGAGGAGGATAGAAGGCTCTTTGAGGAGATGGAAAAGGAGAAAAGAGAGATGGCTTTACGACTAGAGTCCCTAGAGTCCTAGGTTGCCTCGCAGCAAGCACAGATGCATGAGGTGTGCAAGCTTCAGTAAAGTCTCAATTCACCACCCTCCTCCAGTAGTTCAAGGATGGAGGGCAAATTCCGATGTGATTCATAACGGGTCCAAATTCCGATGTGATTCATAACGGGTCAAAGGCCCCAGATCATAATTTTTTATATTTTATTTGAGGTTTTATTTTATTATGATGATGTAGACTTAACTTTTGCTAGTTAAATACGTTGGAATTTAATGATTTTGAATTTGAAGTTATGATTCAATA

>ONT.7797.1 LG04 87727831-87731587

AGGGATCATAGCTTCCTTTCTTGAGTGGAGAATGACATTTCTACAGCTTTTCCATCCAAGATGACTTTCACTTTATGATAAATTCTCACTTTATGATAAATTTGCGCAGAGAGCCATGACAATCTTATCAGCCATTTCATCATCCACCCTCCTCACCATCAATCTCTCTCACACAGCTTCAACAGACATGGAAATCTCTCTCCAACAGCTTCAACAGGCATGAAAATCTCCATAAGCCCATCTCCTCTTTCAGAATCAAAGCTGCAAAGCTGCCTCTAGGAACTCTACAGTTGATGGAAGATATATAAAAACTTCAAATGGGTCATGGAATTTTCAGGTGCAAATGCCTAAAGTAGAGCCCCAGCTCACTCCCCATTTCTTGGATTTACTAAAACAGCTGAGATATGGAATTCCAGAGCATGTATGATTAGCCTCATTGGGACTTTCATAGTTGAGCTGGCGGGTTCGTTTTTTACCATTTTATGATTTAATACCATTTTTAAGATTGCAATTGAATACTGGGGGCAGAGAGTGAATTGTTATCCATTCATTGTTTGGAGTTAAGCTGAATTATGAAACCACAAGAGAAAGAACCCAACCTTTTTGCCTTTTCTTGCCCCCAAATTATAGTAAGTGCATATCGGAAATGGAAGCAGTTTGTCGAAATCTCTCTGTTTTAATGTTTCTGTTCCACTGCGTTCATATCTAGTTTCATGATTGAGTACATTGTCAGAACTTGAAGTGATTATGTTATCAATTTCTCTCTTGGTTTGGATTCTTTTGA

>ONT.10669.1 LG06 38763041-38795030

CCGCAACCACTGCCAAATACACCCAGGAGCACTCTCCATTCTCCAGTGCAAGGCTCCAAGTGGAAAGCAGATATTGGACACAAGTAGTCAAGGGGAGCTTCAGTTGGTGATATCTGATGTACCATTGCGTATGTATGTTAGTTGAAGTTAATATATATTGGAAAAGACACTAGAAGAGCAGTAAGAAAAGACTCCAGAATAGCAGCAGCTTCAAGTTTATTCATGTAGGTAAAATGGAGCAACAAGTAAATTGCTCTTATCTAGCCAGCCTATCACCAGAGGATTTCCAAGATCTGGAGATTGATATTCAATCAGCAGGTGAGTTTTCTAGCCCAGTGGATATTGATCTTCTCGAGTGGAAAGCTGCTTATGATGGAAGATATGCATGCACTTAACAAGGATAAAACATTGGATATTTGTTGATTTTGTAGAAGAAATGTGTGTGGTAGGATGTAAGTGGGTGTATACTGTGAAGTGTGATTTGGATGGTATAATTCTATGAAAAATCAAGGCTAGTGGTAAAAGAATTTACTAACTTATGGT

>ONT.10048.2 LG06 8840721-8842000

GTCAGACTTTTTGTCTTTCTTTTGCCAACTCTCCATCAGACTCAAATCACTTCCCCCAAAATATCACTTTGTTTGCCCCTTTCCATCCCTAGCTCCCCTCCTCTTTCTAGGCACCGATCCTCCCAGGTGAGACTCTATCCGGCGATCATTTTGCTCCGGCGAGAACGTCTCTCTCTTCGGTGACGCTCCTTCTCCTGCCCTGGGCTTTTCTCTCCAGCGACTGTCTCTTTGAGGTTGTGACGAGGTTGGAGAAAGGTTGGGTGGGATGGGATTGATTGGAGTCTAAGTTTCCTATTTTGTTAGGTTGTGACGAGGTTGGAAAAAGGTTGGGTGGGATGGGATTGATTGGAGTCTAAGTTTGAGATGGGCTCAAGGGGATGGCTTGGATTCAATTTTCCTGTAGATTTTCATTCTCTCTGTGATCATCAGTTTTCTCCCTCTGCAACCGGTAGAAATGGAAGCCTGGGTTTTATTGTTTTTAATTTCTTTTATGTGATTGATCTAAGTCTTTGAGTTAGAGATGGGTTTAAAGGGAGGGTTTGGATGGGTGTCCTGTCCTGTCCCAATCTTCAAACACCAATTGCTATCCTGTCCCCAAGTTATCGCTAAAAAGAGGAGATTTGAGATTTTGCCCCATTTCAGTATACATGTTTTGATTCAAAGGTGATAAAAACTTGTAATCCAAAGGTGAGGAACACTCCGGCTTGTTGGAATTCTTAGAAGATTGCACTTTGTTTCCATGTGAAAATGGTTGGGGGAGTAATTATATGAACCTCTATTATGGATATGAATGTATATTCTGGTAGTGAATATGTACATCCAAGGCCCAAAAATTGGCATTTCAAACTGTTGAACTGAAAGGCCAGACTATGAATAAGGATTAGGGCTATGTTGATGGGTGAACGGTGAATCTGGTCGAAAGAGGGTTCGGTTGATGTCTAGATTTCAGTATTCCCATTGTAGTTTCAAAGGTTGACATACTTTCTTTTAGAGATTCTCCATGTTCTGGTTTTTGTAATTTTGCGGGTTGTAACTTGTAACATTCATGGCGCTGCTGTGTATCACATTATAGCGGTTCCTGTAATTTTTTGGGTTGCATATGAGTTGTAACAATGGCGGTTCTTGTAATTTTATGGGTTGCATATGAGTTGTAACATGTTCGTTCCTGTGAAACTTATGAAATTTGTGTTGTATTGAA

>ONT.13151.2 LG08 62254610-62260544

GAGAAGCTCGGTAGCGAGAAGAGGAGGAACTCCAATGGCGGAAAAACCCGAAACGGAGAGAGAGATCGCAAATTTAGCGTACCGAGCAGCAAAGGCTAACAAGAACGTGCCAAACGCTGCCTCTTCCGATTATGTTTCCTACTCCTAATAGCTGAAGCTGGAAACCCTAGAAAAAGAGCTACATCAGAAGTCAGAACCCTTCAAGCTACGTCAATCATGCACAGGGACATTGCCTCGGTGAATGCCAACTTGGCTCAGGCCTCAGATGTTAGTCAAGGACAATATCTGTAAATTTGTCAGCACTCGGACTCCATACATGGCCAATGAATACTGCTCCTTTTGGTTTTTCCCTGAACTGGTTTTGGCCATATGTGATGTTGGCGGGAGCATTTTATGCTATTACAGTTGTCGAGAATCTAACAGAATTTTCATAGTTGTCTGAGGTTATTGTATTTGGAGTAGTAACTGAAAGATCCTTTCCCTTTATTTCCCCCCTATTCGATGCGTCTTTACTGCTATTAGAAGTTGAGAAAATTGTCCGGGCAGGTTCCTTTTCTTCTAAAAAAATTCATATTCAGAGGTACCGGTGTCTTGTATATGCAGTTGCTGGAATCTGCACAACTGAGTATCCACATATAAAGTCTTGAGCAACTATTCTGCTGAATTGTACTGGTTGCTGATTATCT

>ONT.3031.3 LG02 12742807-12744468

CTTCTTTTGCAAAAATTAAGAGGAAAAAGAAAAAAGAGTCACCCCAACACGGGTGTTTGTTATATGTTACTCTGAGCTGTTCTTGCAAGAATCCTTTTCTATTCTCAGGAATCCAAGGTTACAAATGGCTCTCCATGGTTGAAGTGGATCTTGTTAGGGGAGAATGAAATGGGGCTCAGACTCATCTGAAAAGGTACCGGGGCCTGCCTAAATGAGTGGCTTCAATTTCTCACATATGTTAAATGCAACTAGGAGATTCAGGCGGGTCAGGCAGGCTTATCTGAAACTATTCTTTGAGCTTTGAGTGGTCTATGGGATAAGGAGAAGCTTTGCAACTAACTGAACACAAGCTTGTACAGCATCCAATCAGGATAGCTCCCAAGAAAGCAGCAAAACTGAGAAAGTTCGAAGAAAAGCAACTTCCTCCAGCAAATGAGGAAACACAAACTACTGGTCACCTATGGTTCAAAATGTCACTGATAGTTTGTAATTATGTCTGTACAGAACATTTACATTGTATCGGCCAATATGGTCAACAGTATTGATCAAAATGACTTTTTTGATGTCCCGTACGTAAACATATCCTATGTGCTAACACAGTAAAGCATTATAATATGATACGATATTCAGTTTTTTTTT

>ONT.6896.1 LG04 74819281-74821254

ATTCGACTGTGGCTTGTTTGGTTGAGGAGAAGGACCTTTCTTCCTCTTTTTTTCTTTCTTTTCCTTTGCTCTTTTCCTCTCTCGATCATGAAAGGAATTGTGTATGAACATGTGGAGGTTAACTTGGATTTATTCTTCATCTTCTCCATCTTCTCCATCTTCCTTCTCTACTCTTGGTTCTTCCTTCTCTACTCTTGGTTGACAAGAACCCATCTCCATCATTCCCTACTCTTTCCAAATTCGGATTTATTGTTGAGTGGATTTGTGGTTAGGGTTTGATATTTGGGGGATTTTGGTTCATTTGAGCAAAAGATTAGAGAAAGGGCTTGGAAAGCTAATTGAGGCAAGTATAAGCTTTATTTGGTTTTACATTTTGTCTATTTCTTGATTGGTTTCAGATTCTATTATGGTACACTTCTAGCCTCTAGTTAAAATTAGAAATGAATATGAAATGGGTTGGATTTCTTTTGATGAAATGGTGGATGAATGGCTTGGTGGTGTGAAGGAGAGCATGTAGGCTTTATTTTCTGCAGGGATGTGGTTTGGAGAAGCTTAAAATGAGTTATGGAAGTGTAAAAATACTCCTCTCAATGGAGACCAAGGCCCAGAAATTGTTATAGAAATTCCCAGAGAATGTTCACCTAATTTACATTAAGAAAAATTAATGGGAGACCTTTTAAGGCTATCATTTGGCACCTTGATCATTCTATGTCCGACGGAAACCTCTAAAAGCATGAGATATCTAGAGAGGCTAGGATAACTCATGTTTCCTACCGGATGTTCTATCACCATGCCATGTATGTTAGGACCTTTTGTATTTAAATGCATAATGTGACACTAAATAGAATAGTAATACATGGGTTCTCTAAATTTATGTCGAATTGGACGGTGAGGAACAATGGATAGGTAGATTAGGATTGGCTAGTAATTTCCTTGATTCCTTAAATTCAATGCATTTCATATTGAGTTAGACTTTATCATTGACTTCAAAGTTGTTTTATCAATGCATTATATATTGGTGTATTTTAATTCGATTACTATAATTGCATTTGATTCTTTTGAAACGATTATGAATGTGAACAATAACAATTACTATTCGATTCGATTCAATTTGAGATGAAATAAATTGAAACTTTATTCACATTCACTTATTATTCGATTCGATTGAGTCGGCACTACTTAGTTTCAACCTTATGATACCTGACGGGGTTAAACTCGGTTTATTATTCGAGACTACTGTAACAAGTCATCTGGAGCACTGGAACTCCCAACCTAGGTAGCCGGGTAGCTTTTGGCCGAGCCGCTGGTGACATCCTAACTAGATTCGCTCTCTTTTGAGAAGATGAGCTCAATGTAGTAGCCAAATTAGTATACTTATTCTCCATGTGATTCTTAAAGCATGAATTATTCGATTTTGGCACCATTGCCTAGTTATTACTTGCTATTGACATCTTGTAATCAATTGTCATTTAATTCGATTTGAAACATCATGTATTTCTATATTTGCTAAATTGTCGTATTTGAGATAATATTCCTGGCGTCACAGACGCTGGTGCAGCTCTCGATCCTGAGAAGGGCCAGAAGGAAGAGTAGGAGACCGAGGAGGTTCCTCTCGCATTAGAGGAGTGGCCAGATAATGGCCCTATGTAGTCTGGTCCTCGGAGAGTGATGTGGAAACTTTTGAGAACTTAATTGGAGAGTGTGACTGACTCATTTTTGTGTGGGTCACACTAGATAGATAGATGCAGAAATATATTTTGGAAATATGTTTTGTATAGAGTTGTAGGGTGATTTTTGTAATCCCTACTTTGGTTCTAATATGTAATTATGTTTTGTATTTGACATTTAACTTTAAATAGGATTGTTCAATGAAATGGT

>ONT.10694.1 LG06 46888454-46889762

AGAAAAGAAAAAAAGAGTTGAACGCGCCCAAATCGAACGGAAGGGAGGAGCGACTTTGCCATCTCGATCGCCCGGAGCCAAATCGTGCGATCGATCCCTCTCTCCCGCCGTCTCATCATCTCCTCTCTCGTACCTCTCCTTTTCAACGTCCCATTTCTCTCTACTCCCTCGCGACGACCCGGTGGCATGGCAAATCCCCAAATCTGGTCGGGGTTCTTCTCTCTCTCGAGATCATCTCACTCTCTCGCTAGGCGTCGCATCTCACCCTCCCCTCTCGGTCTCTAATGCGGCTTCTCTCCGGAACCTCGGGCATCTCTTCCTAATTTCTCGCCTGCCAAACCAGGGGGACTGTTGATCTACATGCGGACATGCCAAGAATGCCAGAAGCTCCATCCGATACACCTGTTGAAGGAAGCTCTTTAGATGTGTCCGGTTCAAATGTCAACCCAAGGACATGGCATTTGGTTTATATTTGATGTTGAATGGCCAAGTTACTTTTGTCAAATTTAGTATTTAATTTTATTATGAATTTGATGCCCAGTTTAAGATCAA

>ONT.7976.2 LG05 7475127-7483157

GGGTGGATTTAGAATTTCTCCTCCTCTGTATACGCTGTTCTGGTCACTTGCATTGTCGAAAAGTTTCGCCCCGATACAACATTATCAAGTGGCAAAAATCATTCCGGCTTCCGTCTCAGGCCACTGTGTTTCATCTGACGACAGATCGAAGCTTCCAGCAGGTTGCATAGAAGAATTTGAGTATATCAAATGGCCACAGGACTGGAAAATATTGGTGGAACTGAATTGGGGATCGAAAATGTGTTGTTGGTGCGACAGATTGAGCCTGGGAGGCTGAACTGTGACACCCTTCTCAACAGGTTAGCTGAGTTTAATACTTATGCAGTAACCTGCAGCCGAATGGATGATTGTCTGTTCCTTGTGTTTTGTGGTGGCACAGAAATCCGGATTGACTAGGGAGATGCCCTGTTTCCTGTCATTTCTGTCCACATTGATTGTCTGGGGTGGAACAGGGGAAAGAAAGTATTAGAAGTGGAACAGACAGCCATGGCCAATATGTTGAAACCCTAGATATCTGCGGGCATCATCGACATTGAGGCGGCAAGGTGAACAAACTTTATGCGCAAAGGTAGCTTCCCCAGTCACCTTTTGTTGGTCAATACAAACTAACAAACGGTATTTCAGCTTTGGTTATGCAGTTACCAGACGAGGCTCTTGTGAACTCGATATAGCGGTTCCATTCTGATCTGTAGTTGTTTCCAGTTTCCAAAATAATTAAAGTGTCTCTGTTTGGTGTACTCCTCTTCCCTGCAAAAATGTTATCTGCCAGGCATGTTTTTATA

>ONT.14996.1 LG10 64677191-64680344

ATCCATTTCCCTGCAACAGTTCCAGCAAGAGCTGGAACCACTCCTCTCTCTCAGCCACTCTCTCTCTCCTCTCTCCCTTGGCTTCTCTTGATTGTAAATGGAGATTTGATCTCCTCATCATCTTCCATACCTTCCAAGCTTTCCAAAGATGTTATTTTCATGGATTTTGAGCAAAGGCAACTTCTCTTTTCAAGCTATTCTTCTTGGTTCAAGGCCTTAAAGGGTTTTCATGGGAAAATTTGGGGGTTTTGGTGGGTTTGGAGCTAGAGACTTCTTTGAGGCTTTTGTGGAGTGATTTGTGCATCTTCTTATCTAAGGTTTCTTGTTCTTCCCTGCTTTTCAGATGTGTGGATCGGGGGAGGCGATAGAGCCACCGGTCATCGTGAGAGCCTACTTAAGGATATCATATAGATGTCAAGGTGGGCAACAGAGTTCGTAACAACTGTTGGGATTAGTATTTGTTTAGACATTTTATTTTGGGTATGGCTTTTATTAGCCTTTTCTTGGGTTTTGTTAATGAAAATACTTTGTTTTAATTGTAACTTGAATATTCATGGGAAATAAGAATATCCTGTTACTGTCAG

>ONT.10166.1 LG06 49015150-49023129

GTCTTCTTTGCTTTCCATCATCTCCTCCCTGCTTCCAACCAAGTGCTCAGCTGCTTCTACAGAGCGTCTACTTCTACAACAGTAAGAGGTTTCCGCGCAATTTCTATGTTGCTTCCTGAGAGTTGAAAATTGTGTGTCCTGGCAAGGTCCCATGGCCCAGCCCGGCACACTTGGATTGGAAATAAACGTTGCCTACCAGTACCCTCTCATTTTCATCACTCGCCTTCAGTTTTTGTATGCACTGTTTCGCTAAGCTCTCTCTCGCACTAAGTTCAATTAGTTTTTTCTCCCATACCAAGTTCAGTTAGTTTTGAAAAGATCAATATCAAATCATCAAGCCCCAACTCCAAGACGCTCTTTCTCTCTGTGTCAAGTCTTCTTCACACTCGAAGCTGATAAATCACATCTTTGCTCATACGAATCTACCCAAGCAAAAGTGGCGACGATGATAGAACTGTCTCTGCTATGTAACATATGCTGGGATGCTGTTGTAGAGGAGGCTGATTTTGATGCCATTTTGCAATAGAATAACTAAAAAATCTACTTTCATATACCATGCATGTATAATAAATGAACGTTATAAGATTAATTAAATTAGACAAGATGGCCAAGCACATAA

>ONT.14171.1 LG09 62943462-62951734

CACTTTCCCGTGGTAGGGAAGGACGATCAGCTTCACCATCTCCATCTCCATCTCCATCTCCCTCTCTCTCGTTGTATTTCTCTCTCCCTCCCTCCCTCTCCCTCTCTCCCTCCCACCATCGGTTCTCTCTCTTTCTCACTCCTTGTGCTGGCGCCGCCACCGGAGCTCTGCATTGTCCCGCCGCACTCTCATCTCGTGAGCTCGATTTGGTTTTTCTTTGCTGCTATTTCGATCTCAAGAAATCTCTCTGCCTTAAGAGAGCAGGTGCAGAGCAGAGCAGAGATTGGTTCACACTTCGGTGCTCAGTTGCATTGGCACTAGTTTCACATGATTGTGCAGAGATCAAACCTGGGAAGGGTCCATAGCATGATTGAGAAACTAGCACAGTTTACTGATATCAGATGTTGCCTTGTGGTCAGTGGGCTTTCAATGAAGGGAGCATGAATTTTATGGACGAGTGGCTTTCTTCTCTTAAGAGCAGCTGAAGTGAGATAAATCTCCGCTTGGCCCTTTCTGAAGTTGTTTCTTCCTTTTTCTTCTGATGAAGATCGTAAAGATCCTTAGCTATTAACCAAACTCCAATCTAAGCTGAAAGTGGCAAGCAGTGTGCTGGATTCAAAGAATATATCTGAAAATGGAATGTGTGATACCAACCATGTTTCTAGAGGAATGAGTTTAAAGGCTGAAGGGAAGGAATGAAGCAGCGTCCAAGGTTGTGATTTAAGTTCAAGTTCAGACATTCAAGAAATTCTATTTGTTTTCCTCCCTCTAGAAGTTCAAATTAGCTGTGCTGAATCTTGGGTTTTGCTTCAATTTCAGAACTCGAGCATTTTAAAAATGGGAAACCAAAGGGTCAATCATCTACCCAATTGTCTTATGGATCAGACCGAGAGAAGATCAAGTTATGGGCCGCTGAGGATTGGACCCATATATTAAATGGGCACACACTATCGGTCGGCTTGTCTTTAGCTATTATGGAATATCATCATTTCACTCAGATTCAGTGGTTTGGACTGGCTGGAACAGCTTAGTTTTGGACCAAAGTGAATCCACAGCTGGGCATGATGATGAGTGGTTCTGATCTCAGGCAGATCTGATTGAGTGAATTGTGTAACATGATTGATGAAACGATGCACATAAAGGTGACCCGGTGAGATTACTGAGTCTGCTTTACTCAGTTTGATCTCAGTGAGTTTTTTTTTTTTTTTTTTTTTTGAGTTACTATAAGGCTTTCAGGTTGGATTTGGGCAGTGTCCAGGCCCGAGTCCATTTGCAATTTAGGAATCAAAGCTCGGGACTAAGTCTGATATAGGCCCTATAGTTCAGATTGGAGCTATCGGACCTACTTTTCCAATGGAAAAGTTCTGAACTTAGTGGTGTTCTACAGAATTTTGTCCACATTTGTAATGATCTTTTACATGGAGAACTGATGTAAACTTTGAACTTGATTATGAATCACTGCTTTTCCCTTCAA

>ONT.7059.11 LG04 86525879-86716220

AGCAATCCGTCACCTCTTTGACCCTTTCTCGCCTGCCATCCCTTCCAGCAGCTCTTTCCACCGATTCCTCCAGCGGCTCTTTCCAATCCTCCTCCTCCATAATCACGCCTTCTTGAGCTGTTTTTGAGGTATTGGTGACTCAACTCGGGCCAATTTTCAAAACACGGAGCTAACAGACAGCAGATTGAATAAAAATTGAAAAACAACTTCTGTTGAAAGAGTCATTGCTGCTAGGATGCAGCCAACATGAAGGCCAAGTATGTAGAGTAGAATGTTTGGCATTTGAATGTTGGTATAGAGTAGAATCCTTTTGTGGTGAATGACCACTTCTCTTACGCGGTGATTCTCCACTGTGTGCTAGAAGATAAATCTGATTGGGTTGACAAACCATTGTGTGCTAGATATATGCTTTGTGCTTGTGTATGTTGGCTGCCCACCACTTTAGAAATCCTGCTGTGACTTTTCTCTTATCATGCAACAGTATTGTTGAGTTGTAAACTGCATCATGGTTCTGGCCTAATTTTCAGTTTATTGACCGGTTGTTCGAAGGCTTTCTTCAGGATTCCTCTTTGTACCTTTTCTTTTAATCTAAT

>ONT.15801.2 LG10 33733199-33738878

GGCCCTCGTCTCCATCTATCGTTCTTATCCAATTGCTCCTAGGCGTGCGAACCCGATTCCCTCATCTCTCTCCTCGGCATCATCTCCATCCCTCACCTCCATCACCACTCACGCAGCCCACCCACTATCTCCACCCCTCTTTCCATTTTCTGTTTTCTGTTTTCTTTTCTCCTTTTCTTTTACTTTCTGCCATGGTTTTAGGTGGAGGCCGAGTGGGCTGAGACCGGAAGGAGGGAGGAAGGAGAGGGAGGAGAGAGAAGGGGAGCTCGCCAGCTGGTGGCCCAGGTGGAGGCCGAGTGGGGAGCAGGGAGAGTGACAGCAGAGTGAGGGGAGAAGGAAGAAGGGAGCTCGCCAGTTAGTCAAGGTGGAGGCTAGGCGCAATCAGGAGAGTGAGGGAGAAGTCACCAGTGAGGAGATGGAAAGTGGTGAAGGCGGAAATCGAATGGGTCTTGTGTTGTGTGTGCTCATTTGATGCAGAATGGGCCATATGTACGAGTGAATGTAAACGCTCAAGGGTCTTCTTATTCTAGCAAGTGATCTTAGATTTGAGGGGACAAGAGACTTGGACGGGGGTTTGTCAGATGGTGCTACTATGCAAGGCATACGAAGAATCTCATTAACAGAGTTGGAGTGTATGGTGGCTGTCGATTACTTTTTGATTCACTTATAAAAACCAGGGAAGAGCTTGGCTTGTGCAGCAGAAAATGATAATTCAGAAGTGATTGATATTTCTTTTGCAAGAGCATACATTCACATTTTCACCTCACAGAATGCATTAAGGAGATGATGACTGCAGCCTAGAAAGAGTGACATCTCACCATCTCTAGGGGAAATTTTAAAGAGACTAGTGATTTTCTTAGTTTCCTTTTTTGTGAATGCTATTGTTATTGTTTTTGTATTTATCTAAGTTGTAGTGAGGATGTAGGCAGATATTTCTGTGCCTCATATGGGCTGATATTTGTA

>ONT.13868.1 LG09 41319527-41322577

TCATCTCCCTCTCCCACTACCACACAAGCCATCTCCTCTCCCAGGCGCAGAACCCGGCCCTTATCCTCCATCTCCCAGGCGACCTCATCCCCATCTCCCTCTCCCTCTCCCTCTCATCAACTCCGTCTCACGATCCCTCTCATCACCTGCACAGACCTGCCCAGCAGCCCCCCATCTCCATCTCCATCTTCTTCCTCCATGCACAAACCTCTCCATCACCATTCCTCTCTCACGCCTCACCCTCTCCGCGCCTGTGTCTCCATTACCGACCCATCTCACATCCCAGGATCTGCCACAGAATGTAGATCCAGACTCCGGTCACCACCACTACGCTGCCCTACAATGAGGAGAGAGGAACAGAGAGATGGGAGAAGAAGAACGGTTTGGTTATGTATTTCTCTGATCAGGAGCAGAAATCGGAGACTGTCAAGGAATGAAATCTCTACACGCAGGTCGGCGAAAAAGACGATCGTTGGAGATCTCTGCACACAGATCTCAGCACGTTTGGGGCATATGGGAGGCTGGTGGTTGCGACTTAAAAATCAGCTGGAGCAATTAAGAAATGATCTCATACATATATACTGGGGAAAGAAGGCATTCATCTGATCGGCTCTTTCATGGTTTGGTTTTGGCGAACTTCAGATCTGATCTCGGATCCATGAGCTTCATTTTTTTTTTTCCAGCATAGGAGTAAAACCGAGAATACAAGGGCCAGCAGTGAGACATCGTGATCTATTTCAAGTGAGCATGTCACTGATGGGGCTAAAGATTCTTCCTCTGATCATGGATCTGAAAAATCAGAGATGAAATTACTATATTCATATTCTGGTTGTGGGGCGATGTCAATAGGCTTGTGCCTTGGACCTAATATTGCAGGGATCAATCTTGTCAAAGTAAGCCTCTGATTCTTGCCTATTCTGTCTTCAAGTTTTTGTTGTGTTTACATGTAATTTACTACTTACATTGGCATCTATAGAGGTGGGTTGTGGATCTAAATGAATATATGCTTGTCAGAGCCTTAAAGAGAACAACCCTGAGACAGAGGTATATTTATTCTTCCACAGCGGCTGTTGTTACATTTTTTTATATTATTATTGTTGTTGTTTTTTAAAATTCTCCCAGATGATTGGCCTTACATAATTTTAAAATGTTGCATCAAAATTTTATTTAGGAAGATTGCTCAAAAGCTTGTCTTTTATTTCTTGCTTCACTTAAAGTTTAAACTTTACTTCACTTAAAATTTTAACACTACAAGAAAATAAATTGTTGATGGGTTGGTTGTAGAATAGAGATTTAGAGAGATCTTACCATTTAGTATATGCATGATAGGTGTTTGTGACAATGCCTAATACTAAAGATAAATAGAGGAGGAAGAAGATGTTTTGTTTGAGGAACCTGATACTAAAGATAAAATAGAGGAGGAAGAAGATGTTTTGTTTAAGGAAAGCCAGACTCAAAATGTTATAATCACCAGTTCAAAAAGACAAATAGAATAGGAAAAAGATGTTAGTGTCTGGTGTTTGTGGTGTTTTGGTCTTGTGGGGTGCTGGGCTATTGTGGTCGAAGTTGGTGTTTGATGATACTTGTTTCTTGTGGTATCCTATGGGTGGTTGCTGTTCAGGTGTCTTTATGTGGTTTTGATGTTTTAGGGCTTTTTTTAAAGGTTGTGTGATGTGCTGTAGTCTGGACTATTACTACTAGTATAATAAAGTGTGATGACTGTCATAGAAGATTCAACC

>ONT.10045.2 LG06 8489953-8499581

CAGATTCTTTCTTCTTCTTATTCCAATCTCCCAATTCGGAGACTCTCACAGCTCTTATCAGCCTACGGCTATTGATGCTCGCATTTCCGGAAACCTGAATCTGATTCCTACGTTTGATTCCACGCTCCTTCATGGGAAAGTTGGATTTTTGCGGCAGTTGCGATTTTTAGCGTTCTTCTGAAATCTGAAATCGCTAAATTTGTTCGAATGGGGAAGTGAAATCAGGGTTGAGAGAATTGGAAGAATCGAATCTGGAATTTGAGAGAGAGGGTCTGTAAGTGCAGAAAATATAACTCACAAGATTAGAGGGTGCCTTAGAGGACTTATCCAATCGACTATGGAGAGCTATCCGCTTCAGGAACCTGAAAGGAAAGAAATAGTAGTCAAAGAACTCTGGCTGTGGGCAGCGCAGGGCTCTCCAACGCCTAAGTTAGAGCAGGGACCAAGATCGGAGAAGAAGAGAGGCAAAGTAATGACTAGGAGATTGCATGCATGTGTGTGTACCTCGTTTTTTTTTGTGGTCATTCACCTTTTATGAGAAAAACTCTGGATGCCTGTTAGCGGGCCTAATGATTTGACTGCTAAGGTCAGTAATTAATGAGGATCCATCATAGCCCACATTGCATTAAATGAAGGTC

>ONT.17087.1 LG12 29209413-29215307

AATCAACCCATCTCCAACACGCGCATAGTAAACCCTCACATCGCCCTTGCCCTCTGCATCTCCCTCTCTCTCGGTCCCTCGCGCATCTCTCTCTCAACACACGCAGCGCCAACCCATCTCGCCTCCATCCCTCTGTCTCCCTGCGCAGTTGATAACGGACTGATTCACAAGTCTCTCTCGATCCCTCCGTCTCTCTCACCTTTGCGTCTCGCCCGTCTCCCAATTCGGTTCTGGTTTCATAACAGATCGAAGAATAGAGGAGAAGAGAGTCTGACTTGCTCTTTGAAGGACTGCAGTCCCCAATCGTCGCTGCCGGAGAAGGACGGGGCTGCCCGCCATCCATACCTCGCCGTTCGTCCCACCAGAGAAGAACGAGGGCCTGGACTGTTGTCGACCTATTGTCGCCTTGATCCTCGCCCCCCTTCTCGAATCTGCAGAATTGAATGAAAATTGAAAAATAAATTTTGTTAAAAGAGTCCATGTAGCTAGGATGCAGCCAACATGAAGGCCAGGTATACAGAGTAACACATTGCTATATGTTAAAATGAGATGTATGAAGTTTTTTAAAACAATGGTACAAGTGTTGGAATGGTGCAACAAGGAAATGATCATATATGAATATGTAAATTTTCATTAACATTTGAAGGTTGGTACAGAGTAGAATGTTATCAATTGTTTTAATTTTGTAATGGTAATGGTAAAGTGCTTTCAATGTTGAAAAGATGTTTGAATATTAATATGAAGTGGAATGAATTTTCTTATTTCATTTTTTGATG

>ONT.5010.16 LG02 116691434-116695143

ACACATCTTTCTAGGGTTCCTCGTTGCCTTTTCTTTCTTGTTTCTGTTTTTCTTAGATCTGCTCGTTTTCTTTCGTCTTTCGGCTGTCTGACGGATCAAGACTCATAAGGCGATGGAGGGGATTCCAGTTTCGCAGATCGTAGTCTCTTATAGCAGTAAAGAGACTCCTTTCGCAAGATCCGTTGCATTTTGGCGTTCCTCGTCGACGATTTCATCGGATCCATGGGTTTTTTTAGCGATCTTTCATCAGATCTACTATGTATGGATGTAAAGAAAAGTAGTTTAGTGTGAAGATCGAGGAAAAGCTTGTTATGTATGAAGCTGATAGAGAAATAGGTATGGATTAAGCGAGAGAGAGGCTTGTGGCAATGGATCTATGTCATCCTCCGACCTTTGCCATGTTGGGTGCGCTCGCATGGCAGGTCAAAAAAGCCTAATAAAATTCTCGATTTTTGGTTCTCGGGGGAGTTCGCATGGGCACTTTCGCCTGGGTTCTCCCCCTCCTGTGTGTCCTCTCTTTCTTTTCGTTTCTCCATCTCTTTCTTTCTGTGCGTTTGATTAGCAATATCTGCAACTCGACATCACGCTTGCGAAACCGAGCAGAAGAAAGCTACGATGTGGAAATCTCTCGTTATATCATGGCAACATCACAAAGGTACCTACCATCGTGCTTGAAGTTTATTTCTTTAAATTATTCTAAGTTCATGCAGCCACAATTAAAATGGTTATAGCACTCACCCCACCAAAAAAAAAAAACATGTTCACGATGTTCTACTTGATAATGTTTTTGGAATCGTAGTTTAGTTGTAAATTCATAGAGAATTATGTGGCTAGGTTGATGGCATCATGAAAGGGGAAAAAGATTGTGGCTTGTAATCTCAACATATCTTGTTGACCAAGCTGTATTTTGTTGGGCTTCAGATCTTATGAGCTCATCACATCATCTTTTGCTGGGACTGACTCCTAATGTGGTTATAGTCTCAGTGGATTTTTTTATAATTTTTATGAAGCTCGCATGAACAGCTTGTTGACTTGGTGGTGTTTGATTAAAAAGCTCACCTTTTTATTCTTCCATGGTCCGACTATGTCTTTAAAATTTACTTCCTAGAAATTATTGAAATTATTTCCAAAAGGTTCATAATGGAATCATCAAAGAATATTCTCATCTATGCAATATGGATGAAGGTGATTTGAAAATGAATACCCAGACCTTGCCTTAGTAAATCAAATGAGATGAAAGTTATGGGCATACGATTTCATATCCGAAAGAGTGGGCTTAACAGTAAGTTTTCATATACCTCACTATTTCTGGTGATATCTTTCTTATCAAAATGTCACAATTATTGTAGACAATTGAATTTAATGGATTCAGCACTGGGTTTTTAATGTCTGCAACAAAAACAATTGAAATTTAATAGTCTTTATATCTAAGTAGTACAATAGGCCTAATCTGCCAGTTGTTGGTGCATTTAACTTACATCATGCAACTACCCCTGTTATTGAACACTGTAAGATTGAATTTACTGATTGAATACTTCTCAAATGACTAATCATCATCTCTGCTGGATTTCTGATGACCTGGGCATTGAGAAGAGTTAGGATGTCAATACAAAATATAACATTGCAATATCAGTGGGCAAATCATTTTTTAAGACGATAGATGTTTTTAAGACATTGGCCTCTTTGTGATGGAATCGGTTGGGTAGATTGGGATTGGTCGGTGAGCTGTTGTGCTATGAGAGGATTCTGTTATTGAGGGAAATTGTTACGTTCCCACAGTTTCCCAAGTTGGAAGGGATAAAATTATGCTTTTAAGTACTTTTAATTCACTATTGAATCCTCTCGTTGCATAACACTTGTGCACAAGTAAGTCTTCAATGTAGTTGGTTTTTTTTTTTTCCGAATGTTGGAACGGAGAAAATTTAGCTCAATTTCTAGTTCATTTTCCTTTGTAGGTTTTAACATGACCAACTCTTTCCACGAACTTGTGGCAGAAAACTTTAAGAATCAGTAAGAACTTTGCAAATGCAAGATTTCTAAAAAAGAAATTTTTATGGGTTGAAATGCCGTGATTACTTTCAACTGACCTGTTGGTAATGAAAATTTTGCTAGTCCTGAATTTAAATCGTCGCCCGTCCAGCCCTGTCGATTATAAGCATACAATTAGCATATGATGCCTTCACATGTCAGCTACTTTATGCTTATCGACAACAGCTTATGCAACTCATGAAAATTTTTAGGAGGAAAGGTTAAGTTAAGAAAAGTGTTGGCCGTGGTAAACAAAAATGGGTAATTAGCAAGAGCAACAAAGAAAAAAATTAGTTATCCCACATATCATTTCATTAGTTCCACGTTCCCCAGCACGATAATAGCAATTTTTTTTTCAGTTGATTATGGTTGAAAGTTCATTGAATGAGATATATGCATGTTATGGAATTATCTAATTTGATGATATGTGCAATTGATGGGAGTCCCACGGATAATTCATATTAAGTTCTCATATTTATATTCTGGTTCCATGCCTTGTTTCAATGCACTCCTTTGTCATGGCTTGTTTTGCTTTGTTATTGGGGACACTGAAATATTTATCGTGTATGGATTTTTTGTTTTTTCAACCTTGTAAAGAACTATTTTGTTGAGGTGTGTGCACATGATGTACGGTTAAACTATTTTTAGTCCTAGTGTAGCATAAATTCTGTAGGGCAGTTGGGTAATTGTCTGCTTCCAAGTTTAGTCTTGTTTGGGGAGAGAGCATATGCAACGGTGGAGCAGCTGATCAGCAAGAGGAAAGGTGAAGGTGTATTCTTTGTTGCTGCTGGACGTGACTGATAGGAGGCTCCTTGGGGATTGGATGATTTCTTTGCTGTTGGTGGACCTGAAGAACAGTAGTGACGGAATAACTTTGGTTCGATGAATTCTTTGCTGCTGGTTGACCCGTTGATGGTTCACATTGCAATCCTCGGTTGGATGAATTCTTTGGATGTGGACGACATAGGGCAACGGGGATGAACTGTGTTTTATCAATTCATTTCTGCTTTTGCATCTTTGCCTTCCAGACAGACCCTCTCACCCGTAGGAGTTTAAAAAATCAATGCACGAGCAGGCTTATTATGCTGGTTCCTTGTTCGTAGGTTATGGCCAAACCCATGGCGTTTGTTTTCCTTCAAATAATATGCCTCTCGACAATGCTTTAACTAACAGGTCGGCGGCTTTTTACCACATGGTGGTGCAGTGGAGTTTGCGCGCTATCTATTCAAGCTTTGGAGTTCGTCTTTTGTTCTGGACTGTTTTTATCATGCGTTTCCAGAGTTGTTGCTGAATATTATTCCAGACTTCTTTTC

>ONT.10314.1 LG06 63340831-63344455

GATTTTTTTTTGTGTCGATCGAATGTGCAATGCAGATCGTTGCTTGCAGCAGAGGCGGAAGGAAGAGAGAGAGCAAAAGAGAAGATGATTTACAGAAGATGGAGTCTGCTCATAACCCCACCGATCATCGTAGGGTCGATCGCATCCGTCACTCTCTTCTACAACTTCTTCTTGGGCGGCAACGAACGGTTTTTCAGTAAGTATAAGCAGCTGGAATCATCATCAACAACCACCGTTTCTACTGAGAATGCATAAAGAGCAGTAGCCATGACTTGAGCTTTTCAGTATTCTGTGTGTTTTCTTGTGAAAACTTATGTTTGAAGTAGCAGATGCCTGAGAATGGATATTTGACCTGAATAAAATTCGGCAGCATCAATAACAACTTTTGTGTTTTAGCGAAAAGATATTGTAGGCCATGGTGATGGAAATCAATTGCAAGTCCATAATTT

>ONT.13855.1 LG09 37834251-37859108

GAAAGACTCAGACGTGCAAGAAGTGAGAAACCCTATAAATATATAGGAAGCGAGAACTCACATGCTGGGAAACTACTCACCTCTCATCACCATGATGTATTTTCTTAATCACGGACAATAAATGCTGTGAACCCATCTTCCTTGTACTGGCCTGGCGGTTTCGTTCCTGTCGAATTTCAGAGGGCTGTATAGAAGCCAGGTGTAAGGGGGAACAAACACTCGTTCCTTTGAAGCTAGAAAGGTAGTAAGGTGTTGTAGTTTCATGCTTTATGCTTTTGGATTCACAGAATTCGTTAAATTCTCTTGAGTTACTGTATCTGTTGTTTTCTTCCAATTTGAAAGGGGACATTAGATTTTGACCTTTGAAGTCATCAGCGGGTTTGAGAGCATCGGACATACCACGGCACCCGGAAATTTTTTTTTTCTTTTTCCTCTAAATGTAATGGATATATGTTATTGAAACTTTTGTAATTAATGTTTTGAATGAAATTAATATAAGATTTTATTTTTTTATTTGTGATTT

>ONT.641.7 Contig01843 19682-38688

GCTTCGAACTTCTAACTGCTCAATTGCAACTGCAAGACCACTTGTAAAATATGGATTCCAAGAAGATATCCAGATTGGTGGGGGAGGGAACAACCCCCAGGTCCAAGGGAATATTAGTGCAGGTACTGGAGGTAACATCAATTTAGGAGTTCAAAGAGCAAATGTCAATTCGTGAAATCAAGTAAGAGGTGGTGGTAACATCAATACGGGAAATCAAGGATATGTGGTGGTGGTAACATCAATACGGGAAGTCAAGGATACGGTGGAGGTAATGCCAATACGAGAACTCAAGAATAAGGTGGTGAGAGCTGGACCCACTGAAAATTAATTTCGCCAGTTATCTATGTTTCATGTGTCTTATATAATGTTTAAGCTTTGTCTACTATCAACTATGTCCGTTTACTACGTGTGATGCAGTACAACATCATTTCTGCATTTTGTGGATTCCAAGTGGCTATTGTGTAATGGTTGCTTGCTTATTAATATAAGTTTGTGTTATATAT

>ONT.6981.1 LG04 80612182-80617251

ACAAACCCGTTCTGCATCTCCCACCCTCTCGGCCCCGTCACCCATCTCGATCCCTCATGTCTCTAAGACTCTAACCCATCCCTCTCTCGACCGCCATCCTCTCCACCCGCCACCCTGCGTCTCGATCCCTCCGTCTCCCCTGCGTAGAGAATCCACATCACATGCGCAGACCACCATCTCCCTCTCCCACGCAAGCCATCTCCCTCACGACGTCAACCCTCTCCTCCATCTCCCAGGTGACCTGGTCCCCCTCTCCTTCTCATCATCTCCCTCTCTCGATCACCCTGTATCTCGCGGCTGTGCTTGAATATTTCCCTGCTGAGCATGTTTGGTTGGTCCCAGAAAGATGTGTACATTGAATGCAAGCACACCTGCACTTGCCATTCCCAATGTGAGCTCTCAAGGTTTCTTAAAGGTGGAAGATTTCCTGATAAAGTTGTAAAAATCCAAGAAGTAGAGCCTCTGAGTGAATATGACACATCCATTTGTTCTCTAGGTGTTGTGAGGTGTCACTCATATAACAATCTTGGATTAAGTGAACCCATACACAGTTCTTTTGAGGATCAAATGAAAATTGAAGAAAGTTTTTTGTTGAAAGATTCATGCAACTAAGATGCTTCAAACATGAAGTAAAAATGGAAGCAGACTAGCATATTACTATATGTTAAAATAGGATGTATGAAGTTTTTTTTAACAATGTTGCAGGTGTTGGATTGGAGCAACAATGAGATGATTGTATATGAAGATGTAAATTTTCATTGGCATTTGAATGTTGGCAAAGAGTAGAATGTTAGCATGAATTTTTATCTTGTAATGGTAATGGTAAAGTGCTTTTAATATTGTAAGGACATTTGAAGATTAATATGAAGTAGAATGAATATTTGGAATTGAA

>ONT.5832.1 LG03 3866850-3871058

AGGCAAAAAGAATATTGAGAAGAATGAGACGAAGAAGAAGAGGAGAAAGACCAGGGAGAGAAGAAAAATAAAGAAGCATCCCCAGCCGGCAGGCCTTGGCGTAGAGACCTCGTCTCTCTGTTTGGCAGCCAGGATCATACATACTATCGTCAAATTCTAGTAACCCTCAAAACCTTGCCCAAGCTCCTTATGCAACCTTAAAACGGGAACGTTTTTTGGTGGGATTCAGATGGATTGATTGATTTTCTCCTCTTTGGTAAGTATGACAACCAGTGAAGCTTATTCCTTATTTCAAAAGATTTGATTTATTACAATCTCTTATTTTGAGTTATAATCTCTTATTCAGTAGAGTTTCTAAGGGGTTTTGAGCTTTATTTATGTGCAGCATGTACATGGGTCATCTAGCACACATTTATCGATAAGTCATGCTGAAAATCAAACCATAATAGATATTTATAGAAACTAAACTTCAACAAGCAA

>ONT.10874.2 LG06 64158618-64167054

ACGCTCCACTCTGCGTTCTCTTTCGCCTCTCACTCAGCTCTGCCGCTTCTGCTCTCTTCTCCACTCTGCTCTCAGTGTTCTTCATCTCTCGCTCAGCTCTGTACTCTCTACTCTCTGCTCTGCTCTCACCGTCGCGGCGAGCCCTCGCACTACTGTCGCACGCAGCGTCTCCCTCTCTCACCCTCTTTCTCTCTTGCCCTAGCCCTCTCTCACTCTTCCTCCCTCGCCCAGTCGGCAGTCGCCCCCTGCCTTCCCCCTCTCTCACCCTCACTCTCACCCTCCTTCCATCTCCCATGTTCTCTATCTCTCTCTATTTCTAATCCCGTACTTCCTCTCCCGCTTCCTCTCCCCATCTCTCAGTCTATGCTCAGAGATACCGCTCAAGGTTTTTTTCTTTGTGAGGAAATCTTAGAGTCAATCGAGTGGCAGACATCCATAACTTCGGGATTGTGGGATACTAAGAGTGGAGACGTGGAGTCCAACAATGGAAGGTGCTCTCAACTTGAATGTGGAAGAGTGCTTCAAAACGAGAATGGAGAGGTGTCTGTTTTGTTTTCGAAGTCCATCGGAGTTTTTTACCAAATAAAAAAGGTCCATCGGAGTTGAAGATTCTAATGAGGCAGAGGTGCCTGCCATTCTAGAAGCTTTGGGATTATATCCCAATTCGTTCTGAGAAAGCTTAGTCGTGGAGATCGATTCAGTCAATGCTATCTCCTGGGCATTGTGTAAAGATGCGACTCCTTGATCCGCTACTATCTAAATGATGTCAGATACCTTCTTATATGATCCAGGTGTGATTTTTAATCATGTATATAGTTTGGCTAATGAGCTGACTGCTTCTTTAGCCAAGCAAGGAGTGGATGGAATGGCACCCCGTGATGGGTCTTAATGTTTGTTTGTTTCTGTTTTTGGTGAGTGGTGTTACAGTCTCCAGTATACTACTATACTCCAACCTAACCATGGGGTTGTTTCTGTATGTACATGTGTATAGAACTTTAGTATGCACTCCAGTCCCATTACTCTTTGGCTTTAA

>ONT.10662.6 LG06 33950235-33960468

GGAACTGTAGGCGGAGGGGTTTCAGTCGAGAGGGAGAGAAATGAGGAAGAAGGAGGCTTGGGGTCTTATATACTGAGGGTCTCTTCTCATACCTAGGGCTCTTCTTCCCACTACATCATTCTGTTTGTTAGAGGGTTTTCTCGGTCACGCTCACCCTTCTTCTCAGACCCCGGATATAAGTTGGAAAGGTATACAGCTAATGACTCACGTATTATGGTAGATGTTGTATATAATCCAAAGTGGAACTTTCCTCATGCTCTGCCAGGTAAATATTATGTAATTGACACAGGGTATCCTAACACACAAGGTTTCCTTTGACCTTATAACCACCGTCGCTCTCATATTCAAGACTTCCGCCATGATGCTGGATCTAGAGATAATGCGGAGCTCTTTAATTTCTATCATTCCTCACTAAAGAATGTAATAGAAGTATAGAACGTTGCTTCAGGAAGCTAAAGGCGATCTTCCCAATATTGAAGGAAATGACTCCTTGCCCATTTCCTACTCAAATGTATATAATCGTCACTACAATGACAATGTATAACTTCATCCACCAGTAAGCGATAATTGATACCTTGTTCAA

>ONT.10128.1 LG06 27624870-27627973

GAGAGAAGTCTCTTCTCCCCTACATTACCATCGTCCAGCAACAAAGAGCTCTGTCTTCCCCTTTCTCATTTCTTCTCCTTTTCCATGGTGGAGATCATCTACTAGTGGACCCTGCCTGCAAGATCCGACCCCGCCAAGTGATTCTACATCCTCTCCTTCACAAGATACATCGTTTTCACGAGGTTCCGTGACCGGCCATCCCATGATCCCTCTTCTTCTCCTTCTTTGAGCTTCTTAGAGGGGCTTTTTGGAGGTGGATTGTGAAAGGTTTGAGGAGGCGTTCCAGCTGAGATTTGGACCATTCGTTGAGTGCTTGGACTGCTTTGGAGCGATGCCTGGTGCCATAGGTATGATTTCCTGACATGTACCCTAGTACGGTTTTTGAGGAGGCGGTTGCTGAGCCACAAGTGACATGTGATCAAGATGCAGAGGTTGTCATTTAGACTCCACCCCTTACACATGAGACGCGAAATACCTAGGAGTATTATTTTGGGTTTTGTATTATGTTTTCCTGGCCTTTGTTGGCCTTTTATTTTGGAGAATGCAATTATTACTTCTTTTGTAATTTCTAACTAGAAACATGGGAATGATTTATCCTGTTTACTTGTCTTGCTCTATT

>ONT.2954.2 LG02 6898917-6908151

CCATGAAACCCTTCTTGCTACTCTCCCTCTCGTCGATCCACAGCCGGAGCTCGAGCCATCGATCGCAGGCAATAATGTTTTGAGGAGGTCGCAGTTGATTTGAGTACTTTGTTGGACAGAATCAAGCTCTCAAGCATTACTTTGTTGATCAAATTACGAGGACTTGGAGGGAAGTGGTTGCAAGTTTATCAATGCGGTATTTCCAAGGAGGATTCATAAATAGGTTTTGAATTCCAAGAGAGTATTTATTCCATGGAAAAGGAATGAAAACCATACTCTCTACATCAATTCTGCAAAGATTCTTGGATGTATTGTTATCAACGATTCAAGAGAGGAACTTACACAAGCAAGTACATACCCATTATCGCACGTGTTAGCGAGCATCAACCTCCAACCTGGCCATCTTATTTTATGGACATCAATGTTTGGGTGTTTTTAGCTCCAGCTGGAATTATTATTCTTCATCAAACCCATGTCTAAATCGGAGGGACTCAAGGATTTCTCAGATTCTTCATCTCGGGTACCTCTCACTAGTATTTAAAAACAGTAATATTTTATATTATCAGCGACCCAAC

>ONT.222.2 Contig00654 56345-57627

GTTTATCTGTTATATTGGAGTGAAAACCCTCTTCTTCTTCTTGGAAACGATGGTTGGGTACTTGGGTTGGGGAATGTGGGTTGGAGAACAGGGAGATGAATGATAAGGTTAAAGGGATTATCTGTCCAACTCTACACACAAGAGGTGGAAAGAGATTTAGTGCGAAAAATGATTCATCCATTTCTCGATCTTTATGGGTTGGACAGATGGCTCGTCCATCCATCATCCGAACTGGGTTTGAATCAGATCTGTTTATTGCCAATAATCTTCTGCTTATGTATTGCATTTGCGGTTTCATCTCAGATGCACTCCAACTGTTTGATCTTATTTTTTTTATTTTCCATTCTATTTGTTATTAGTGTATATAATGTTGTTTTTTTTTTGAATTTGATAAATTGGAAGAAGGGATGGCTTTCTTAAAAAAGTTTTGCAGCCTGGTTTGATAGTTCTACTTGTTTTTTACAATGGATGCCTCACTAGTTTTTGACAATGGAGAAATATTTTTCTATTGGTTTACTCACTGAGAATGATTGGTTGTGTATCTCAGAAACAGGCAACTACTGCTCTGGGGGTCCATTCATGAGGAGTTCCATATCAGCTGTTGGCCTCTAATTGATCATCGTCAATGGATTCAGGCTGGTGACTAAAATAGTAAGGCATGGACCTATTCAACCCCAGACAATCCACTTCTTGTTACTAATGCTTGGGGTCTGTGAAATTCTTCAATGCACCCCTTCCGAGCAAAATTGGAGTTCATTTGGGCCATCTGCGTGTATGTTAAAGGCTACACTTGCATTTGGATAATCTAACTACACTTGCATTTGGATAATCTAACTTTATAATTCTTAACTAGGCAGATGTAGCAGAGTTACAGGTAAACCAGGTCACAAGGTCTCTGAGCTCCAAGGATCCAAAATGGGAGTTCAGTTAAAAACTGCAGGCCCAGATCTAGCAGACAATTCAAACCCAGATGGAAACAATAGGAGGATGGCATGTGTTTTATGTTCTAGTTAATGTTGTTCCCTCCTATCATAACTTGTGTACAGATAGAAGGATGCACTTGAACAGCTTATCAACCTGTGTAGCAGCTTGACTGAAATCTGTTTTTCAATTTTCATAGCTTAGTAATTGTGTTGCTTTATTTAAACTAAAGTCCCTCCTTTC

>ONT.13408.1 LG09 42820086-42822435

GAATGATTTCGTTCTTCTTCTCTTTGCCAACTTTCTCCTTTCCCGCCTTTTTTCTCTTTCTCCTCTGCTCCCTCCTCTCTATTGCCCTCATCATTCCTATTTAAAGGTCGAATAACTCCCTAAATCTGCTTAGAGAGTGAGACATCATCAATTGGATCATCAGGTGAGATGAGCTTCAGGAAAATGGATGACAAGAAGCGCCCACGTGGGTTTGTATCTGAAGCTGATTTGCATGGAATACAGTCATTCCTTCTGATTTTGATAAGTGGAAGACGATCGTAGAGGGTATTCTTCAGAAAGAGGGAGGTGGTCTGCAGGCATGGTATAGCCTGTCCTGAATAAGGTTGTGGAATTGGGTTGCAGTCATAGACAAGAAGCCAATGTCCATCCTTGTTTGTGCATATTTGGTTGATGGGAGGGTTATCGCTACCGGTTGTACCATAGTTGTTGATGTTGCTACTATAAAGTTCATCATTGTTTATATTTCCCCTAAAGACATAGACATGACAGTTGTGCTCTAAATTTGTTATTGCGTTAACCATTTATTATATCGTCATTGAATCATTGTTGCACTCGCATCGTTCTGGATAAAGTGGTCTATGTAGTAATCAAGTTATAAGTTTGGAAGGCCACAATTGTTGAA

>ONT.6772.1 LG04 50068351-50069255

GAACTCTGAAGCCTTACCATCCCTTCTCAGTTCTAGCAAAGAAGAAGATGGGTATGACATGGAATGAGAGTGGGTTTCTTGGAGCTTGAGGACTAGGGGTATATGGAAAGCACCTTTCTTACTTACAAAGAAAGAAAGAAGAAGTTTTGCTTTCCTATACCACCGAGTCCTTTGGTTTCACGAGACTCCTCTCTTTCCTTCTCTACCCTTATTACTGTTGACGTTCACTGCATGTCAAGTTGCGTATCTCAGATCAGATAGTGAGGATTGGAGGGGCATGAAGGAGGAGCTGTCTTCATCCTTTTTGGGAATAAAGGAGGCCCCAATGTTCTTCCTTTCGTGATCATGATGGATATTTGATGTGGTGGTTGTCAATATCTTTTATTTCAGTCCATTGAATTGGGGCCCATCTGATGTAGTCCACCTTTTGAGAAATATTGGCAAAATTTAATCTTTTTAATCAACTCAGATCAAACCATTTGATCAGGTGAGTTCTGCGAATCTCATTGGTATCAATTGGTCAGCTGTGAGTTTATGTAATAAGTGGCTTAAATCATTAATTGTTTGCATTCA

>ONT.14958.1 LG10 49230969-49233616

GCCCTCCACGAGATGGAGCACGCCCAGCCCTGCAACAACAGCTCCCTTCCCTTGTTTTTCTCACTTCGCTGCTTCCAAACTCAAGATCTATGCTACCAATTACCAAATGAAGCTTGCCATTGCATTCTCCTCTATCTCTTCTTGCTCTAGACACCATTTTTGTGGGAAACCATGAATGACAGCCCCCTCATTTAGTTATTCCTTGCTGGTTGGACCACTTATATGGATTTTTGTGTAGATTGGTGATTTGAGCTTAAGAGTGTGATTAGAGGCTTGATTTGCTATCTTAGACCAAAGGCAAGTCCAGACTTCTTCGTCCCGAGCTGAGTGCTTTTGTAGGCTGTGGAGTCATGATTTGCAGGGTTTTTTTTCCGACTATGTCATGCAGATATTAAACTTGACATCGGAATTGCATCACATATGGGTAGTGGTTATTAGCTTGTTGTTGTATGACTTTTATTAGCCATTTCTTAGGAGATTTTAATTAATGCACATTGATAATTTGTAACTTGAACATTTGTGGGAATGCTTATCTTGTTACTATC

>ONT.13855.2 LG09 37834261-37860879

ATTTCTGCTTGATGCAGTTGCACGCTTTTTAAAGCTGGTGTCAGTTGTGACCACCATGTGTAGCCGGTCCATGCACCCAATGAAGAAAGCCTAGCTGATGGTGCTATGACGCATGAGAAAGACTCAGACGTGCAAGAAGTGAGAAACCCTATAAATATATAGGAAGCGAGAACTCACATGCTGGGAAACTACTCACCTCTCATCACCATGATGTATTTTCTTAATCACGGACAATAAATGCTGTGAACCCATCTTCCTTGTACTGGCCTGGCGGTTTCGTTCCTGTCGAATTTCAGAGGGCTGTATAGAAGCCAGGTGTAAGGGGGAACAAACACTCGTTCCTTTGAAGCTAGAAAGGTAGTAAGGTGTTGTAGTTTCATGCTTTATGCTTTTGGATTCACAGAATTCGTTAAATTCTCTTGAGTTACTGTATCTGTTGTTTTCTTCCAATTTGAAAGGGGACATTAGATTTTGACCTTTGAAGTCATCAGCGGGTTTGAGAGCATCGGACATACCACGGCACCCGGAAATTTTTTTTTTCTTTTTCCTCTAAATGTAATGGATATATGTTATTGAAACTTTTGTAATTAATGTTTTGAATGAAATTAATATAAGATTTTATTTTTTTA

>ONT.7482.3 LG04 24334948-24347053

GTTCCCAATTTTTTCCCCTCCCTCCCGCGCCCTGTCCTCCCGTGCACCAGCGACGACACTGCCGGAAGCCGCCTACCCCGGAGCTGCCACCGGTGCTCCCTCCCTCCCTCCCTCTCTCTCTCTCTCTCCCTCTACCTCTCACTCTGAACTGTGCCCTCCCGTCGCCGGTGCCGCCAATCCGTCCTCATCTGCGATCCCGACTGCCCTTTCTCCGTCACTGCTTTCGACGCCAATCACTGTCCAGGAAACCCCGTTTTTGCTCGCACCATTCAATCCTGCTACAGCTGCTGGTCATGTGGTCGACGTTTGTTCCCTTTATTCCCAACAGCATTTTGCCTGGGCTCCTCACATTCATTGCAGCAGTGGCTGCTGTTGTTATGGACACCAGACGCTGGCAGCGGCCACAGGGCTAGACACGACTTCGGTGGAGAGGGCAGTCATGATTTAACAGGCTCGAGTGATCCTGATCAGGTTTAAGAGCATCGGATATACCACGGCACAGGGCAATTTTTTTTTTCCGTTTTCCTGTAATTGTAATGGATATATTTTATTGAAACTTTTGTAATGTTTTGAATGCAATTAATATAAGACTTTCTTTTTTTATTTGCAATTTTA

>ONT.2161.3 LG01 21842020-21848412

ACTGCAAGGCTGCAAACTCGTCAAATAGGTCTTTCATTATCTGGCTCTTGAGGACTGGAATGAGATGCAAAAGTTTTAAACCCTAGAAAAGGGTTTCGTAGCACTGCTCTCTCTGAACTTCTGTAACTGTTACATATAAATCGACGAGGTGAATGAGAAGGGATAGTGTTGAAAGCGCCAAATCTGCACGAACACGGTAAGATTGAGAGAATCTGGGGGGGTGGGTTCGAGACGATGGAAAGATTCACGCAAGGGCTTCGTTCTCTAGCTCAAGAGTCGAGCAGAAACGCTAGCACTTCTTTCAGCGGCTCTGATTTATCTTCCGATCACGCTCAACTAGTTGTTCAAAGAGCTAATCGGCAAGTTGTTTCTCTGTGGACATGTTCTAAGGTTTGTGCCATCAGCTTTGCTGTAGGCGTCTTTGTGGGCTTCACGTTAAAGCGACGTTGCAAGCGTTGGGTCACCAAATTGCTCAAGCGATTGAAGGATGATTGAAACTTGTGTTCTAAGTTGAATTTTGTCGTATGTTGCCATTATCATTATGGTAGACAAGTAAATCCTTGTAACCTTTTCTTTCTCATCATGCTTACTCCTACCAACCCAACAGAGGCCTTCCCCCAAGATCAGATTTACCC

>ONT.12408.1 LG08 24158979-24162284

ATCCATTTCCCTACAACGGTTCCAGCAAGAGCTGGAACCACTCCTCTCTCAGCCACTCTCTCTCTCCTCTCTCCCTTGGCTTCTCTTGATTGTAAATGGAGATTTGATCTCCTCATCATCTTCCATACCTTCCAAGCTTTCCAAAGATGTTATTTTCATGGATTTTGAGCAAAGGCAACTTCTCTTTTCAAGCTCTTCTTCTTGGTTCAAGGCTTTAAAGGGTTTTCAAGGGAAATTTGGGGGTTTTGGTGGGTTTGGAGCTAGAGACTTCTTTGAGGCTTTTGTGGAGTGATTTGTACATCTTCTAACCCAAGGTTCAGATTTCTTCGTCCCGAGATGAGTGTTTGGTTGGCTTTGGAGCCATGTCTTGCACGTTGTTTTGTCGGCTATGTCATGTAGACATCGAGCTTGACATCGGAGCTGCGTCACTTTTTGGGTAGTGGTTAATAGCTTATTATTGTATGGCTTTTGTTAGCCTTTCTTGGGTTTCTTAATTAATGTACATTGTTAAATTGTAATTTGAACACATTTGGGAATGAAATATCCTGTTACTGTCAGCTC

>ONT.8213.1 LG05 19007403-19015403

GCTCATGTTTGTGCGAGTAGCCTTCTTTCTATTTCGAGCAATATTATACAAAGCTCTCTCTCTCTCTCTCTCTAACAAAATATATAAGAAGAGGCAGATGAAGAAGAAAAAAATGGTGATGAAGAGAAGAAAGAAGAAGAAAAACGTCGCCGTCTTTTCGGGCTTCGATTCTCTCCATCCAAACAGGATGAGTAGGATGGAGGTCCCATGGTGGGGCCAGGAGAGTATGATGAGGGGAACAGTGCTGAACTTCCTTTAGTCAATGCTTGTTGCAAATCAATGGATATTGGCGCATACGGCTCAATCTGTTTCCTGTTTTTTGTCTTTCTTTTTACTACTGCAATTTTACGTTTTTCCATGTTTCTCTTATTTTTCTCCCCCAATGTGTTGAGCATTTGAACATTTTATTTCTATTTCCTATTTCAGATGTGTATTCTACCTGGGATACCAAAATAATAAATTTTAGATATATATGGCTCC

>ONT.7565.1 LG04 63109333-63117467

AACGCAAACCTCTTTATTTTTTTTCCCTCTATTGTTTTCCCTCATCTGCTCCTCTCTCTCTCTCTCTCTCTCTCTCTTTCTGTCTCTCTCATCTGCTCATCTCTCTCTCTCTCGATCGGCGCTCTCTCTCTCTCTCTCTGTCTCTCTCGATCTGCTCTCCTCTCTCCTCTCTCTCTCTCTCTCGATCTGGCTCATCTCTCTCTCGATCTGCACCGTCTTGGACCAAAGCAACTGCTATCTCACCGCACTGCAGCACCACTGAGCCCACCCTCCTCGTCCCTGCAACCCAGCTGATCTCTCTCACCAGCTGCCTTCCTCTTCCAGGTTATTGGTAACAAATGGGATACTTATCTTGATCTTCTTCAAGCTGATTACACAGAAGGATTGATGGACGCTTTGGGGTCGGTTGCAGACTAGCTAAATGTGTTTTAATTTTTTAGTTTATTTGTATATGTATATTGGACAAATATATGAGATTACATCATTATTGATTGTAATAATTGTTTAATGAGATGAATGATTTAATGAAGATAGTTGTTT

>ONT.7556.4 LG04 61082117-61088089

GATACTAATGAACCGAATTCTCAATCCCTCATCTCTCTCTGACCCTCTCTCTCACCTGCTATCCCCTGCGTCTCGATCCCCTTCTCTCCTCCATCTCGATCACCCTAATCCCATCCCAAAAACCCCGCATCTCCATCTTCTTCCTCCCTGCACAGACCTCCCCATTACACCATTTCTCTCTCACGCCTCACCCTCTCCACGCCTCCCCTGTGTATCAAGCATCTCCATCGCCCATCTCACTGCATCCCCTGCGCATCAAACATCTCCATCGCCCATCTCGGTTTCTCCTCTATCCCCATCACCTAAGGCCGATCCCTGCCATTCATCAGAAGATCACCACATCCCTCACCTCTGAGTCTCTCTCGTCTCCCTTTGCATCTCTCTCGGTGTGCCACCTCCTCTGTTATTTTCTCTCCGGCATCTTCAGAGACATAGAGAGAAGCGATGGCGACGAACCTGGTTACATTTCAGAAGATTTTCTATAAGAAGAGAGACAGAACGAAAAAGCGATCGTTACCCCTACGGTGTCATTGAAATCACGCCTCTGCCCAAAAACCTCGGCATTCGCTGCTTCCCCTCTAGGGGAGTGAAATCTACAAAATGTCAACACCCAGCAATAAAGTTTGCATTTATAGTTGGGAAAGGAAAGTTTTGTCAAAGGGGTGCAGCTAGAATTGGTTAAGAACCATGCGGTTAAGCAAATTGCTGGTTTGGTAAAGGAGGGACTTCACAATTATATATACCCAGCAATAAATTTTGTTGATTAGGCCTTCACTTGGGCAAGTTCGTCACATCAAGATCCCTCATCGGAAGCCTGGCTAATTTTGTGGAAGTGTTGTCTCCATTAATCGTCTCAGTCTCAGACAGGCCACGCATGCGGATTCTATTTTCCAACGGCAACCAGATGAGATTCTCATTTAAAGTTGAGATCTTTTTTGAAGCAGAGAATTGGGTTTGTCGTTTGGTGGGGCTTAAGTAGATATAAGAGGTTTTGTTTGGGTTTTGGAGTTTTAGGAAGGCAACATATGAATGTTCTTCAAGCTTGATTTTTTTTTCTTCTCTTTTCTCAGCCATGTGAGGGGTTTACTTCAGGTGAGATTAGGTCCTGTAGTATGATTTAACTGCAGTTGTATTTATGTAATAAGATTTATCTTTTCTTGCTCTTTT

>ONT.674.2 Contig01889 381495-383597

GCGCCAGGATTGTTTACTAGTACCAATCCGGATACTCAATCGACAAACCCATTCTGCATCTCCCTCCCTCTCGGTTCCGTCACCCATCTTCATCCCTCATGTCTCTAACCGATCCCTCTCTCGCCCGCCATCCTCTCCACCCGCTACCCTGCGTCTCGATCCCTCCGTCTCCCCTACGTAAAGAATCCCCATCACATGCGCAGACCACCATCTCTCCCACGCAAGCCATATCCCTCGCGACCTCAACCCTCTCCTCCATCTCCCAGGCGACCTGGTCCCCCTCTCCCTCTCATCATTTCCCTCTCACCGCTCCCTCTCACCACTCCCACTCTTGATCACCCTGTATCTCGCGGCTGTGCTTTAATTATTCCCTGCTGAGATGTGTACATTGAATGCAAGCACACCTGCACTTGCCATTCCCAACGTGAGCTCTCAAGTGATCCCTTATGGAGGTTTTTGTTCATACATGAATCTGCTCTGATTGGAACATTTTTTTGGAAGCTGACAGCTTGTGATTGTTGTGGTTTTTTGGGGGAATTTGTGTCGTTTGGTTCAAGAAGACGAGGATTTGTGTTATTTTTTTAGTTAGGTTATGGAAAGATGGATTGGAACTAGGAATTGCGATTTGAGTTGCTGAAGGTTTTGAATTCTGATTGTTTTGAGATTCTTTCATGTCTATTGTAGGAGATGAGTTTTTGAAGTGAAGTAATTAGGTTCTTCTGTTGTTCCTCTTTTTTTTTTTTTTGTAATGTGACTAATTCATATATGATTTGCATTCATTGTTCAAGGACGATTGAGCTTTCTGGTGCTGCAATTCACCAACAAGGGTTTAGTTC

>ONT.17053.2 LG12 14990130-14993295

ATCCATTTCCCTGCAACGGTTCCAGCAAGAGCTGGAACCACTCCTCTCTCTCAGCCACTCTCTCTCTCCTCTCTCCCCTCTCCCTTGGCTTCTCTTGATTGTAAATGGAGATTTGATCTCCTCATCATCTTCCATACCTTCCAAGCTTTCCAAAGATGTTATTTTCATGGATTTTGAGCAAAGGCAACTTCTCTTTTCAAGCTCTTCTTCTTGGTTCAAGGCTTTAAAGGGTTTTCATGGGAAAATTTAGGGGTTTTGGTGGGTTTGGAGGTAGAGACTTCTTTGAGGCTTTTGTGGAGTGATTTGTGCATCTTCTTATCTAAGGTTTCTTGTTCTTCCCTGCTTTTCAGATGTGTGGATCGGGGGAGGCGGTAGAGCCACCGGTCATCGTGAGAGCCTACTTAAGGATATCATATAGATGTCAAGGTGGGCAACAGAGTTCGTAACAACTGTTGGGATTAGTATTTGTTTAGACATTTTATTTTGGGTATGGCTTTTATTAGCCTTTTCTTGGGTTTTGTTAATGAAAATACTTTGTTTTAATTGTAACTTGAATATTCATGGGAAATAAGAATATCCTGTTATTGTCAG

>ONT.7059.13 LG04 86710702-86716244

CTGTCGCCCAGCAATCCCTCATCTCTTTGACCCTTTCTCGCCTGCCATCCCTTCCAGCGGCTTTTTCCACCGATTCCTCCAGCGGCTCTTTCCAATCCTCCTCCTCCTCCATAATCACGCCTTCTTGAGCTGTTTTTGAGGATGACTCAACTCGGGCCAATTTTCAAAACACAGAGCTAACAGACAACAGATTGAATAAAAATTGAAAAACAACTTCTGTTGAAAGAGTCATTGCTGCTAGGATGCAGCCAACATGAAGGCCAAGTATGTAGAGTAGAATGTTTGGCATTTGAATGTTGGTATAGAGTAGAATCCTTTTGTGGTGAATGACCACTTCTCTTACGCGGTGATTCTCCACTGTGTGCTAGAAGATAAATCTGATTGGGTTGACAAACCATTGTGTGCTAGATATATGCTTTGTGCTTGTGTATGTTGGCTGCCCACCACTTTAGAAATCCTGCTGTGACTTTTCTCTTATCATGCAACAGTATTGTTGAGTTGTAAACTGCATCATGGTTCTGGCCTAATTTTCAGTTTATTGACCGGTTGTTCGAAGGCTTTCTTCAGGATTCCTCTTTGTACCTTTTCTTTTAATCTAATGTTATACTTAACTGCCAATTTTTC

>ONT.692.2 Contig01934 19661-22140

AAAGAGAAGGCCATGGAGGTGGTACCAAAGGAGCAGAAGGAAGAAAAGGCAACCATTGAAGCACCAAAAGAAGAGAAGGTAGGGACTGCAGTAGAAACCAAGACAGGCGTTTCCTTCCCACCAAACTAGATGATGGGAAGCTGCTTTATTCCGTGGGATTGAGGAAGAAGAGTGTTCTTGGACTCGGTATTAAGATATATGGCTTCGATCACCAAGAAACTAGAAAACATTTTCTACACAAAGGGAGCATACCAACCAATTGATATTGCTGAACATGCAGGGATGTTTGTGGACAATTAGAAGCTAAAAGTTCTTCTGAAATCAAAGATTGAGAAGGCTCCCAAGCAGCCCCCTCACCGAATGCCCCAAAAATTCTTTGTTCCCAGTCAGGGTCAAGGATCAAGGATCCAACTTCCAAGTTCTCTGTCCTTTATTTAAAGCATGTGCACATTGTACCTATCTAATTTAAGTTTCAAAATCCATATTCTGCCGCTAACATGGCCGTGACTGGTCCACTCATGGGTCACCCTTT

>ONT.12838.4 LG08 7195207-7204724

CCCAGGCGACTTCATCCTGCACAGAACCACTCACCAGAGCCTTACCCTCTCCACGCCTGTCTCTCCATTACCGACCCATCTCACAGCCTCCCCTGCGCATTAAACATCTCGGACTCTCCTCTATCCCCATCACCTGAGGCCGATCCCTGCAACCCATCCCTCACCTCACCTCTGCGTCTCTCTCGTCTCCCTCCGCATCTCTCTCGGTGTGCGTCTCCCAGGTTATGAAGACGAGAGGTTGCTGCAAATGTTGCTGAAAGAAGCCATCCATGTGATCAGCTGTGTGGCTATGTGGCCAAGACAGAATGGGGCAAAGAGGCATTTTACAAAGTTATTGGAAGTTGGGCTTCGAAGAAGTTTATGACAGGATGGTGGGCATCATTTCTCAATTTGTGTTTGATCTTTATCTTTCACTCAGTGTTAAAACTGATCTTTTCTTACTGTCATAGATATTGTTTATCTATTGTCCCTTTCCGAAGTTTTTTCCTTGATTATGATATTTTGTGTTTTCTCTTTTGAAATCATGGATGTTCCTTCTTAATTATGATGTTTTTGATTTATTATACTTTTATTCCAGTCGAATGTTACAAAGGGTCATAATCTACTCAGTGAACTTCTTT

>ONT.7297.5 LG04 8975790-8981021

GAGACTCTGTCACGTGTGCGTCACAGCGGATCAGATCTACTAGATTTCAACGAAGAAAACCCTAGGGCCTTGTCCGCCTGGCTTTCTCAATCCAACCTTTACCCCCCTCTCCCTCCCTAGCAGATCAGATCTACGAGATTTCAACGAACACAAGCCCTAGGGCCTTGTCCGGCTGGCTTTCTCAATCCTACATTTACCCCCTTCCTCCCTCTCTCCCTCCTCAACAAATTCATTCCAGCTTCGATCAAGCACACACCTTACCTACCTGACTACCTCTGTCTGTCTGAGTCTATCCTCTCCTCTCTCGTTGATGCCCTTCTTTTCAATATGATATGTCTGAGTCTCTCCTCTCCTCTCTCGAGTGGCCGCAAGAAGGAAGGTCTCTCTCTCTCTCTCTCTCTCTTTTTTTGCTCTTGATTGGTTATCAGATTACAGGTTCATGTTGTGATGAAAGATATGTATTATGATACCTTCTATTTTCTTTTTGGTATTTTTCAGTTGTACGCCAACATAGTACTCTTTGAGCTATCATGTCTTCATGGACTCTCTTGATGAATGCCATTTGATTTGTTTATAAA

>ONT.15413.1 LG10 11277803-11286408

CACTAGCCGAGAGGGGAGATGAGGCGGGAAAGGTGTGGAGAGGAGGAGGAGATGAATAGGGAATAGGGAATAGGGAAGAGGAACGAGAAGGAGGGAAAGAGAGAAAGATGGGAGGAGAGGTGGAGAAGTAGAAGAGGAAGGATAACGATGCAGCAGCAGCAGCCGGAGATGGATGCCAGATGCAGCCCTTGAGGTCCAGCTGTCCAGTCCATTTCACAGATAAGTGCTGAAAAAGGATGACTAGGAGGATGATTTATATGGCTCATGTTGCTTGCATACTTGTAGGGATGGCTAGAAGTTAAGAACTGAAGATTTTTGGGACACCGCAGTCTGTTGCGCGCTTTCCTTGGATACAATGAGTATACAGAATTATAATTGAAGTTGCTGTTGAAACCTGTTTGTGAAGCAGGAGCTTTTATTATTTGTTTGCTGTGGTAAAAAGCTCCTGAGATTATCTTATGCTGCTAATCAAAGAGGATTCGAACTCAAATCACTGTCACATACAAGAGGAACTCAATTTCTGAGATTTGTCTTCTGGGTCAATCTTGTGCTGCTGGCCAAAGTGGTGGAAGGTGCCCATTGTGCCCTCTCGGGTTCCGCGGCATCACCCCAACGGAGACAACATATGTTTTGCATACAATTAAGGAGTGCAAATCTAAATTGAAAGAGAAAGTAACAAGGGGTAGAATTGTAAAGAACTCTAAATTGCAAAAGAGAACTCTTTTTTTTTTTTTTAATAGTCCTTGTCCATGTTTATGACTTTGGGAACTAGATTAGAGGTTTGAATGCTGTAAAGTATGTTTGACTAAAGTAAAGGAATCTTATTTTCCAGTTT

>ONT.3606.1 LG02 99541726-99553582

GGTGTTGAAAAGGGGACCTGGCGTCGCTCTTTTCTCATCTTCTCCTGTCGGCGCCACCACTCCTCCGACTCAACATGTCATTGTCGAGTCATTTTTCTTCTCGTTTCGGAGCTCTCAACCTCAGGAAGGATGTGCAAGGCTATAAAACAAGAGAACTTCATTTTCGCATTGCCACTACAACATGCCTAAGGATTCAGTGGTATAGGAAAGAAGTTGAAAGATGGATCCCGGTCCTTTGGATAAACATATCCTTACTGATCAAGATAATCATATATCAAAAGTGAAATGGGAAGGGCAGGAGCTTGGCCCACTGAAGTGCATCCCAGCTGACCCTTGAAATGAATTCTGGGAATTAAATGAAAGTCAAGAAGAAATTGTCTGTATGGTCGGCCTGCACAATCTGGCCTTAATTGGTAAAATGAAAATCCACCATGGCATTATATGGGCGCTGGTGGAGAGATGGAGACCCGAGACTAATACTTTCCATATCACCTTGTCAGTTAATATATGTTGTGAGCAAATGGCACAGAAAGAATCT

>ONT.7707.1 LG04 79777713-79784793

CAAGGAGAGCCCCCTCCTCTTTACTTAAGCTTTTCATGTAAATTGAGAGCAATTGTAGAGAGAAAGTGAGGAAAGCCTTGTAAAATTTGAAGATTTATAATGAAAGCTTTGTCTTCTCCAATGGGTGGCTAATCACTTTGATTGGATCTTCTAGATGAAGCTAAGATGACGAGAAAACAAAAAGGAGGGAAAATACAAAAACATTGACAAGGATTCAAAAGCGGAAGGAGATAGATACTTCTAGGGTGTCGGTAGAAATCACAGACGATGGAGAAGACAATGGGAGCAAAGAGAAGCAGCTGAGATGCAACTTGGAAGAGGCCAAGCTCGAATTAGCATAGCCTGGCTCAGCCAAGACTGAGATATGGGTTGGACCTAAAACTCCAAATTAGGAATGCAAACAGGTTGGGTTAGGGGTGGATGTATTTTTATAGATTTGGTTTTAAATTTGAATGCAGATCCGAAGCAGAATCTATGTGTTGCATTCAGTTCTTGTACCAACTTAGAAAAACAGATCCTAATCTAAAATAAATTGTAGAGTGTATAAGTTTAATTGGAAGTAAACTAAAAGAGTTCC

>ONT.1318.1 LG01 63930732-63933132

AGCAGATTTTTCCAGCAAAGAGAGGGATTCCAGCAGCCCTCTTCTCCCTTTTCTTCTACATGCATGTTCTCCCAAATCAAGCCCAACTCTCTTAAAATTGGAAATCCAAGCTCATGGTTGTATTCCTCACCTTCCCTCCCTACAACAAGCCTATTTTTCCTACAATTTCAGCTAGGCAGCGCTTTCCTTGAGCAAGTTCTCTCTAATTGGAGCTCTTACAGATTGGATTTCGTGTGTTACTTTGCCCGTTCTACAGTGGGACTTTCGTGTGATGCCCAGCTATACTGGATTACTATAATTATAAGCTCATGTGCTCCTGTCACTTCGTGTGACGGAGGAATTATCGTTTGTGCTCTTGTCACTTCGGTATCAGGTCCTCTATCCCTATTTGGAGTGTGTGCAATGGGCTATGGAGCCTTGTATTTCGGTAAGTTTGTTGTGCTGACGATGTCATTTAGGCGTCATGCTTAGCACCCGAGCTTGTCACATAATTAGGGTAGTATTTTGTTTAGTTAGTTATTTTGTGTGGCTTTTATTAGCCTGTTTTCTTTGGGACTATAATTAATATACATTTGTAAATTGTAACTTGAACATAAATGGGAAATGAATCGTCCTGTTACTATCAG

>ONT.2161.1 LG01 21841863-21848464

GTGGCCGAGGACCTGTCCATAAAATGAAATAATCAATCGCTCTGGCTCGCACACTGCAAGGCTGCAAACTCGTCAAATAGGTCTTTCATTATCTGGCTCTTGAGGACTGGAATGAGATGCAAAAGTTTTAAACCCTAGAAAAGGGTTTCGTAGCACTGCTCTCTCTGAACTTCTGTAACTGTTACATATAAATCGACGAGGTGAATGAGAAGGGATAGTGTTGAAAGCGCCAAATCTGCACGAACACGGTAAGATTGAGAGAATCTGGGGGGGTGGGTTCGAGACGATGGAAAGATTCACGCAAGGGCTTCGTTCTCTAGCTCAAGAGTCGAGCAGAAACGCTAGCACTTCTTTCAGCGGCTCTGATTTATCTTCCGATCACGCTCAACTAGTTGTTCAAAGAGCTAATCGGCAAGTTGTTTCTCTGTGGACATGTTCTAAGGTTTGTGCCATCAGCTTTGCTGTAGGCGTCTTTGTGGGCTTCACGTTAAAGCGACGTTGCAAGCGTTGGGTCACCAAATTGCTCAAGCGATTGAAGGATGATTGAAACTTGTGTTCTAAGTTGAATTTTGTCGTATGTTGCCATTATCATTATGGTAGACAAGTAAATCCTTGTAACCTTTTCTTTCTCATCATGCTTACTCCTACCAACCCAACAGAGGCCTTCCCCCAAGATCAGATTTACCCAACTTTCCAGATGTCAATTTGTTTGTGGGCTGGTGGAATCCCCGTAACTGTAGTTAGTTGCTTTTTTTCTGGTATTGTGTTAGGGTGAGTGCTCAGATTTCTTTGCCATGTGATAATGAGTAGCTTTTGCAGTTAATGATTCTTTTTTTAAGTGAAA

>ONT.13354.2 LG09 17884169-17910021

GTAACTTTCTTTTCTCCTTCCTTTTGATTTTCTTTCTTTCTTTTATTTCCTACACCACCTTGTTGCCAAATGGATCATCAAACCATCATCCTCGTAATCCAACACCGCCCATCATCATCGGAAGAGTCCAAGGTTCATTTTCATACAATTCTCTTGGGATTTCCCCTTCTTCATCATCCTAGGTCCCGTCGTCGGTTATTCCGAGGAACACGAGGAGGCAGTCGGTGAACTGCAGCAGTTAGTTGAGTGGCATGCTGATGATGTCATGTAGACATTAGATTTCTAGAGAGAACATTTTGGGAAAACTTTTAACCATTCTTCTTGGGGACTGGTATTTTGTTTATATTGGCCTTTGTTGGCCTTTATTTTTTGGAATGTAATTAAATGATCTTTTGTATAAAAACTTTAACTTACTTTTGGGTAATATGCTATCGATCATTGCTCATTCTGATATA

>ONT.8342.1 LG05 28534014-28535955

ATTCGACTGTTTCCAGCAAAGAAAAACCCTAGGCTCTGTCTTCTTTACTTTTCTTCATTTCTTCTTCGTTAATTGATCAAACTTGGAGCTTTACAAGTGAATTCCAAAGAAGAATTGTGTTAGCTTTATCTTCTCCATCATCTTCTTCACTTTTCTTTGGAGAAAGAGATAAAGAAAAGCTGTGATTTGCTCTTTGTTGAGTTACAAGCACAACTGCCCATGCCGGTCCTTTTGAGGCCACAGCGCTAGCGGAGACGTCCGATCCGGTTTATTCAGGAGGACTAGTTGAGTGGAAGTGAAACAATATCATATGATTATGATATTGAAACGAAATTTGAAAAGAAAAAATTTTGTTATGGAAATTGGTTGGAAACGTTTCACACTTTAAATCCTTGCATTGTATTTAAATATGCAAGTGATTGGTTTTATAAATGGTATAAATAAGTTTTGTTTATCTTGTAAATGAATATTTTGAGCAATGTATATTTGTTGATATTCTGTATAATGTGTATTTGATA

>ONT.517.1 Contig01548 21551-25265

TCTAAACCCTTCTTCTGAATTTCTTCTTCTTACAACTGCAGCAAGCCAAACCCTTATTCCAAATCTTGTTCTTCTTGCAACTGCAGCAAGCCAAACCCTCCTTTGGAATCGTCTTCTTCTTGCAACTGCAGAAATCCAAACCAATCTTCGGAATCGTCTTCTTCTTGCAACTGTAGAAATCCAAACCAATCTTCGGAATCGTCTTCTTCTTGCAACTGCAGCAAGCGAAGGTAACAATGAGAAGGGGAGATTGATAAGGACTTTAAAGAAAATGTAGCTAGAAGTGACGTGTCAGATTTGGGAGTGACTAGGAAAGGGAGGAAAGTAGAATTAAGCAATGCAACTCTAGATAGATGAGATAAGCCTTCAAGTTATAATGATGACAATATGTAGATTATTAATTGCAAACAACATTGGCATGCATACTGTTTTGATGCATGGTCTTTATGTCTTTTCTATTATTTATCTTTCATCAATGTTAACTGTAACAGTCTATTTTAACTGATGCTTAGTATGTTGATTTA

>ONT.15958.1 LG10 72253913-72257453

CCCCACAGAGATGGACTGCATAGGCTTGTGCCTTGGAGAGAATTTCTCCTCATTCCTACTCCGACCTTATATTAATTAGCCAGCTGCCGCTTTCCCTTCTCCCTTCTCCCTTCTCTGATCGATCGGCTCTCTCTCTCTCTCTCTACACTACAGCATCTCTGCCTTTTTCAACTGTTGTAAACACGTTCGTGGCCCTTAACTACCCTCAGCATGTCAGCTTTTGTAAGATTTGGAAGCCTTGCACCCAAGACTAAGAACTTCATTGTGGCTGGAGGTTTGACAACCTTTGTTTTTGGGGTGTATTTCTACACCATGAGGGCTGTTGGAGGTACTGATGAGCTGCAGGTGGCAATTGATAAATTTGAAGAGCAGAAGGACAAAAAAGAGATTGATACGAGTAGATTATCAAAGTCCTGAATCATCGCCTTTTTCACTTTATTGGCATTTTCTTGTTAGAGTCATGTAGCAAATACTGTTGAAAAGAAAAATAAAGGAGTGAACTTTTGCATTTCCTTCTTTTTGCTGTTGGGCTTGGCAAGCTCTTGGTCTAAAAACTTATGTTGAAAGAGCTTGCAGCGACTAACTCGTTCATGAGTTTCTCTGCTGGAATATATTACACTAAGGTTATTTCAATGAGTAAAGATGTAGATAACCATATC

>ONT.16110.1 LG11 3697515-3765205

GTCGATTGGAAATCATTTACACAAAAGTTTCTTTCTCTCTCTCTCATCTTTCTCTTTCTCTCTCTCTGTCATGGTATCAGAGCTTAGGATATGATCAATCTTCTTCTCTCATTGATCCACTGAGTTTTCCTGGCGTGGATATTCTTCCTGGCGTGCTGATCTTCTCTGTTACTGTCACTGATCAGCAGTGGCCCACCACGGATCATCCTCATATCTCCCAGATATCACTACAGTTGTGATCGACTATCCTTGAGGATCTTTTTAAGGATTTTTATGGAATTTTTCCATCAGCTTTGAGGTAATCTTACTGCGGGTGGGATAAATCAGGATGGCGCTCAGCACGTAGATTTTGGCATGCGTGGCTGAAGAGAGAGAACGAGAAATATTTATATGAGTGACAGAAGAATTAGCTTTTATTTAGCAATTGATTTAGTGTTTAGTACATTATTAATTTGAGTTAGAATATAGGGATTTGGATTATTCAGAGATTTTTGATATTGTTTGACTTTATTTAAATTAATTGGAATTATAGTTGATTTA

>ONT.6143.2 LG03 51763750-51773375

CTTCTTCTGTTCTTCGTTCTTCTTTCTTCTGTTCTTCTTCTTCACTCCTCTAACTCTCAACCTCAGCCTGCCCCTTCTGGCTTCTTGGCTTCTCTTCGGATGCTTGTGAGGCTGTAACAATCATTCGTCGTTCGGCTTCAGATACAGATACAGTTATAGCTCCAGCTCCTGAGGAGGCCCAAGAGGGAGAGTGGGAGAGCAGGAGACCCCAGAGATTCCTTCGGTACTAGAAGAGTGGGTTTTGTTTAGGGGTCTTTTGGGACGTTAGTCCTCGATTCACAGAGCATCATGTGCTTTGTTTAGGGGTCTTCTGGGAAGTTACATCGTGCCATCTTACATTACTTTTTGTTATGAATGTAATGGTGGTATTTGTTGGATATTCGGGGAAATTGTATAGACGCCATATACCTTTTGTATAACTCTTGTATGGTGTTTTTTTGGAATTCCCTGCATTTGAGTAAAATGAACTTTATATTTTGTAT

>ONT.14891.4 LG10 36987010-36992403

AGAAAAACTTCTTTTTTTTTCTTTCTTTTTTTCCTTTTCCTCTCCTTCCATGGCTTGTTGAAGAACAACTTCTTGGAGACTCTCCTTGCAAGATCCAGCTGGGCCAATCAACTCCTCATCATATTCTGAGCCACATAGTTTGTTTTGGTGTGAATCTAGCAGTGACAACCCCCTCTTCCATCACCTTCTCCTTTCTTTGATCGTCTAAGAGAAGAAGGTAGCAGAGAAGGGCAAGAGGGAACTCTTCTGAAGAGCAACAGAGGCCAATCAAGCTTGTGGGAAGGAAGAAGAAGCGGGAACGCATAGTGGATGCAGCGATTGAGTTCTCACCTCGACCGTGCTACGATTTACTACACTAGGAGGTATGCACCGTTTGGGGGAGCATGGCAACGGAGATAATCAGCCATTGGACTTCAATATGCGTGGCTGATAAGAGAGCGTCTGAATGAAACCGAACTAGATATCAGTTTATTTCTTTAAATAAATGTTGAAGTTCTAGGATTTTGATTGAAAGACTTATTTATGTTTCATGTCTAGTTAAATTGATACCTCAAATAAATGTTTTCGTTGCTCAGTTCCTTGTTTTAATTTGATTTATGAATGATTTAGATTCTTTTTAGTAAGACCCGGGATCCTGATGAGCTCCGGGATTCAGGGTGTTACACTCGCCCTGCTTGACCATTGGATGTACCCAGATCAAAAGAGGGTTTTTCGGTTTAGGTGCGAGCATTTAGTTGCCTTGCCCTTCCAAGTTACCTAGATCACCTTGCCTTTCTTCCACCCGTTTTGGGTACCCTATTTTTACCTGTTACACTGCCCGTTATTTTATAGAATTTCCCGGACTTTGAAAATATTGTTTTAAAACCCGTTTTAACTTGATTTACTATTTTAAGCGAGACTTAACTTGGCTTACTGTATTAAAAGCCGAATCAACACTGGTTTCCGTTTTTAAGCATTCTAATTGCTTGTCAACCCCCATACTTACACATATATCTCTTGAGTAATCAAATTACTAATTAACTCCTAATTAAGCACCGAACCCTGCAATCTGTATTGATTCTCGAGTTGGCACCGCCAAGATGTTTTTCCAGTATTATTACCTGGTTACATATATATACACATTGCTTGTTGCAGTTTGAAGTCTTGTATGTGACCGGTGTCACTTAAGTACCCATCCTGTTTGGGGCAGACTTGGTACCCCTATGATAGGGAATCTAGGACGAGTTCCAAGGTACCCTCAGCCAATTCGTTAGGGGCTAGCAATTGCTCCCCCTTCCCAGGAATTATGCGAAGTGGTTGCCAAGTGGCCAATAATCTAAATTAATCATTAGTTATGATTCTATTAATTATATTCTCATGCATTAGCCTACCTAGCTCACTATCGCTAATATTCAGATTAACCATATGTATTTATATTCTGCTTTGTGCCTGCAAACTATGCCTTATATCTTAAATACTTGCCAGACTCATATCTAATCTATGCTTGATGCCGCAGGTAACGAGCTCACATACGTGCCCGATTGGACTTTGCGGCCATCCAACAGGATCTGCCAGTTACACAAGAGTGAGCAGCCGACAACATAGAGTAGTGTCCAAAGCTGCAGAACTCGTAACATTATCTTTTGTGTTTTTTGTTATTTTTAGGGAATTCATCTTTTGTACTTGTAATCAATGGCCATTGTTTTTGTACGGCTTTATTCTGAGATGTAACTAAATTTAATCTTTTGTTACACCTGAATGCTAATTACGAAATGAAATCCTTTTTGCTATTCGGTCTAATGATGCC

>ONT.16720.3 LG11 46784848-46792009

ACGTTCCTCGAATCCTCTCTCTCTCTTCTTCTTCACTTCCATTTCTAGGGTTTCGTACTTCAATCGCTAGGGTTTCTCTCATCCCTTTGTTGTTTCCTCTGCAGAATTCGATCGCATAGAGACTCTGCGTCTCATCTTCTCTAAGGGTTTATTTACTGGTGCTAAACTAAAAGAAGCAGATGGCAGCTCAAAGGATCTCTCATGCCACCTTGAAAGGACCCAGTGTGGTCAAGGAGATCATTATTGGTGCCACGCTCGGCTTGATTGCTGGGGGGTTTTGGAAGATGCATCACTGGAATGAGCAGAGGAGAACCAGGGCCTTCTATGATATGCTAGAGAAAGGCGAGATCAGTGTTGTAGTAGAAGAATAGGTTCTCGTTTCATTCTATTTTCCTCTTCTGATGGTCGACCTTGTTTCTCTGTTACCTTGAATTGTCTTTTGAGAACTTAAGCTGCAAATGTGCAGTATCGGGTACTTCATGAAAGAAATAAGCCATTTGTGTATTGAAACTTGAGAACCGCATTGCTGTGTTTAACAGTCTTAATTATTTCTTCTCTTTTTTGTTTA

>ONT.641.14 Contig01843 19686-80133

AGCCAGCTTCGAACTTCTAACTGCTCAATTGCAACTTCAAGACCACTTGTAAAATATGGATTCCACGAAGGTAATACCCAGTCTGGTGGAGGAGGGAACACCCCCCAGGTCTAAGGGAATATCGGTGTAGGTACTGGAGGTAACGTCAATTTGGGAGTTCAAGGAGCAAACGTCAATTCGGGAAATCAAGTAAGTGGTGGTGGTAACATCAATACGGGAAATCAAGTAAGAGGTGGTGGTAACATCAATACGGGAAATCAAGGATATGTGGTGGTGGTAACATCAATACGGGAAGTCAAGGATACGGTGGAGGTAATGCCAATACGAGAACTCAAGAATAAGGTGGTGAGAGCTGGACCCACTGAAAATTAATTTCGCCAGTTATCTATGTTTCATGTGTCTTATATAATGTTTAAGCTTTGTCTACTATCAACTATGTCCGTTTACTACGTGTGATGCAGTACAACATCATTTCTGCATTTTGTGGATTCCAAGTGGCTATTGTGTAATGGTTGCTTGCTTATTAATATAAGTTTGTGTTAT

>ONT.3533.2 LG02 76289434-76291313

GCCGAATTCTCTCGCTCTCCCATCCCATTCCTTTTCCATCGAGCTCCAGAATCTCCATACCAAAATCAATCTCAGCCAGCGTCAGAATCTCCATCGCTAGTTTCTCATCTCTCTTTGTCTCTCTTAGTCAGATCCAGTAAACCCAACCTAACCCTTTTCCATCGAGCGCCAGAATCTCCAACGCCAAAATTAGCAGTTTGAGCTTCAATCTCAGCCAGCGCCATAATCTCAACTCTCTCTCTCTCTCTCTCTCTCTCTCTCTCTCTCTCTCTCTCTCTCTCGGTCAGATCCAGCGCCAGAAATCGGGCCAAAGCCAGAGTCACCAGTTCGGTGAGATTGAATCTCAGCTAAGCGTAATTGACATGACTCTTTCGTTCCTCTCCCATCCCAACCCTTTTCCTCCCCATCTCTCATCTCTCTTTCTCACTCGGATCCAGCGCCAGAATCTCCATCGCCAGAATCTCTCTTAGAACGCTGACAGAGAACCAGGTCAAGCTTCAATCTCATAAATCCCCCATCCCCAAGCATGAAGACCTCCATCGAATGTAGGAAAACCAATACCTCATCAATCCCCCATTGCCAGAATCTCTCTCCAGCTCCATTAAGCACTCATCATGTACTCTGCCAATATTACAAGAGATGGTGGTCATAGAATGATAATATTTCTCAAGTGTTCGGAGAAGAAGATTAGAGCAGGGCTGGATTTCTATGTGAAGTGTTGGAAGTGGAAGCCTTCTGAGGTTGCAATTCATCATATTCTTCTAATGTCTAGTTTGGAAGAAAGATTGATTCCAAGAAGTAAGGTTTGGAAGTTGCTATTATTTAAGGGGTTGGTTGATAAGGGATAGGCGAAATGGTAGACTGAATTTATTATTATTTTTTTACTTTGATTAATGGATGCTGCTATTATGGAAGTGGACTCTCTGCTGTTGTATGAATCGAATGATTTTTCAAGTTTTCTGTGCTTATTGGGTTTTTATCCAGGTTCTCA

>ONT.674.3 Contig01889 381495-383906

GCGCCAGGATTGTTTACTAGTACCAATCCGGATACTCAATCGACAAACCCATTCTGCATCTCCCTCCCTCTCGGTTCCGTCACCCATCTTCATCCCTCATGTCTCTAACCGATCCCTCTCTCGCCCGCCATCCTCTCCACCCGCTACCCTGCGTCTCGATCCCTCCGTCTCCCCTACGTAAAGAATCCCCATCACATGCGCAGACCACCATCTCTCCCACGCAAGCCATATCCCTCGCGACCTCAACCCTCTCCTCCATCTCCCAGGCGACCTGGTCCCCCTCTCCCTCTCATCATTTCCCTCTCACCGCTCCCTCTCACCACTCCCACTCTTGATCACCCTGTATCTCGCGGCTGTGCTTTAATTATTCCCTGCTGAGATGTGTACATTGAATGCAAGCACACCTGCACTTGCCATTCCCAACGTGAGCTCTCAAGGACGATTGAGCTTTCTGGTGCTGCAATTCACCAACAAGGGTTTAGTTCCAATTAGAATTGGCTAAATGTTCGATTCAAAGGTGAATATTTAACACTGAAATATTTCAATATCGCAGTACAGAGATTGATACCCAATCTTGTAGTGTCTAGTGCCTCTGTGATTGGATGGCTACTAATATGAAGGTGTATGATGTTCATATATTAAAGATTTATGATATTAGGACTATTCAGTGGCTCCAAAACATCCAGACCCTGTATAAATGGCCTTTTTTCTTTCAGGATTTAAATGTAATTGGTCTTTTAAGTACTACTGCAACTATTCATTGGCAGTGAAATAAGAGTCTAGTTCATTACTAA

>ONT.2321.1 LG01 39096031-39115413

GACCTTCCCTCACTTGAGTTGTGAGTTCCGACCTTCCCTCTTCCTTCGACCTGAACGGCTGAACCCTTCTTCCTTCATGTGCACGGATCTGTCGCACTTCTCGTCGGACATCTACGAGGATCTTTGAGCTTCAGGTTTGAAGAGGAACATAGAGAATTTGGAATGGAAAAGAATGTGGTGAATGACTTACATACATTGGTGAAGCTTGGTTCTCAAGTGTACATGCAAGATGATGTTGGGCGACTCGGTCTTCTTCACCTCCGTGGTATTTGGCAGACATTATTGAAGAGCTGCTTTAGTGGTTAGGTGATTTGAACGTTTCGTTCAATCACATTATGAGATCAGCAAATGCGAAGGCGGATAGCAAAAGAGGGAGTTCTTAAACCTTCTTTACTCATTTCTGGCCCTAATTAAGTTCCTTTTCTTTTGTTGGTGCCCAATGTTTTTGGCTCGCAAATCTTTTTATACAACATTCCCCGTGTTTTGTGTAGGGGGGCCAAAAACAGCCCTCTGATACTGTACAGTCTTTGTGCAATGAAAAATCAACTTTTTCAAACAAAAAAAAGTTTGTGTAAAGAAAATGCAAGAGTGAAATACTATAACCCACCAAATTTTCCAAATTGAACATTAAAAATAAGAAACTAAAAGAACAAAAAATAGAAAAAGGTGAAGACGATTAAAAGAAATAAACATTTACAAAAAACATAAATTTCTAAACATACCATACCATAAATAAAATTAAGTTACTAAGATAGATAATAAAGAAAAGAAAATACAGAATATTTAAAATAATGTTCTTCCACCAATAAAGAATCATT

>ONT.17200.2 LG12 42331714-42336212

GCAAAACTACCTGCCATAGAAAGTTGGGTCTAACCATGGGTCTTTCTTGTTGCTCATTTTCCGATGAAGGAGGTCCGGGCATGATGGGATTTCTCATGGTTGTGGTCATTGCTCTAGTGCTTGTGATTATCTGCACCACGCCGCGACGCCGCTTCGTTGTATGTCGTGTGTGCTGAAATTGGTCAGATTTGGAATTTTATTTCCTCTCATTTTCCCTAGTTATGTATTGATCATAATCCTTAGCATGTATTTGCTTTCTACCCATTTTGATTTCTGGTTGCTAGTGAGAATGGTGAATGAATTATAAATAGTTGCTAGATGCAGGGGAGACTGAAGCTCCCTCCACAAGGTTTTTGCTTTTATGCTTTGTGTGGGTAGGCTTTCTTTGCTGCAGCTTCATGAAACATCATTTTAGCAGTCCTGTAATTACATTCATTGTAATTGTGATCTATTCCAGGGTCAAAATGATTCTAATAAAGAAGTGAATTGGCATCTGTTCAACT

>ONT.4106.3 LG02 1698054-1705503

GTTGGCCTCCTAAGTCCTAACCGGAAACCTTTCTCGCTGGCTGCCCCCTCTCATCGATCCACCGCCGGACCTCGAGCCATCGATCGCCGAAGCCGTCTTGTCTCTCAGCCCTAAGGATATCTTCTCACACACCATCAAAGGCCTCAGTATTGAGGTACCGTAAGAGTGAAGGCACAAGAATGGCTTTTGTGATGAGGTGAAGTCTTTGAAGAGTCTTCATGAGTTCCAGAAACAGAATACATTGCTTTTGAGGGCAATTCCTGGGCTGAATGAATTTGGCAAGGCTTTATTAGGTGAATGAAGAACTTAATTAAACGGAGATGGAATGCCTTTTGCTAAGCGGATATACTAATTATGTTAAGTTAATTTTGGCTAGTTAGGGATTTCCGGAAATTGTTTACAATGTAGCTTGCCTTTTTTTGGATGCTTTTGTGAAGGTTTTGGATTATGTCGTGACGCTTTGGTTACGAATTCGAAGGTTAATGTAAATAGTTTGGATTATGGATGGATTCAAATTGGCTATGTTTTGAATAGTTTGTTTGATTTAGTGCGTGGACGAGCAAGTACGTTCTTTAATGTGAATGGTGATTAATTTCTAATTAATGAAATGGTTCTTATTG

>ONT.2229.1 LG01 28127554-28129578

CACCGAATTTCCTTTATCTTCTTCTCCACCCTCTCTACTTCATTTCCACTTCCCTATCTCCAAGCATGAATCCCACAAGGTTTCCAACCCCAAACAAGGACAAGCTTCCCTTGAGCACTGCCATCATCTTCTTCTTCCCCATTTTCACCTTCTAGACATGTGCTATTGAGCATTGGATTAGAGTGGGTTGTGGATGAGCATGAGCAAGATCTTGGGAAACCCTAAGCACCAAAAGGAGGGTTTTTGTGGAAACTACCAAAGGCAATGGAGCCCCATTGTGTAAGTGGCTGGGGTGGTCCCACAAGATTCGCCGTGTTGTTGGATTAATACCTGAGAGCCTCACATTACATCCCGGTTATATGAGCATGGGTGCATCTCTTGTAGTGTTGGACCTTCATTAAAACTATAATGCATATTCTAATTGAACTTTAAATAAATACTTCTGTCACTAGTACCAACTTTGTTATTATGATTCATTGTATTGGTTTA

>ONT.5010.14 LG02 116691431-116695143

ACACATCTTTCTAGGGTTCCTCGTTGCCTTTTCTTTCTTGTTTCTGTTTTTCTTAGATCTGCTCGTTTTCTTTCGTCTTTCGGCTGGTATGCAGTCTATTTCGTATTGATCTATCGCCGATCTACCTATTGATAGTCGATTTTCCTTCTGATTTGCACTTAGCCCTTTGTTTATTGGTTGTTTTGCAGCAGATCTAAGGTATTTCAACGTTTCGATCCTCTTCTTCTTTGTTTCTTCTTGGTTTTCGCGGTTTCTTTGCCTAATCTTCTTTGATTTTTCTTGAAATCGTATGGTAAACGAGGTCTAGATTTGTCATACCTTTCTTTTTACTGTTTGTGTTTGTCGATCTTCCTTTCGTTTTCAGATTTGAGGTAGGCTTGTTCTTTTTGTTGTAACTTAGGGTCTGGTGCTTGCGATTGCAATATTTCTTCTTTTTTATGGTTGTTTTGAAGCAGTCTGACGGATCAAGACTCATAAGGCGATGGAGGGGATTCCAGTTTCGCAGATCGTAGTCTCTTATAGCAGTAAAGAGACTCCTTTCGCAAGATCCGTTGCATTTTGGCGTTCCTCGTCGACGATTTCATCGGATCCATGGGTTTTTTTAGCGATCTTTCATCAGATCTACTATGTATGGATGTAAAGAAAAGTAGTTTAGTGTGAAGATCGAGGAAAAGCTTGTTATGTATGAAGCTGATAGAGAAATAGGTATGGATTAAGCGAGAGAGAGGCTTGTGGCAATGGATCTATGTCATCCTCCGACCTTTGCCATGTTGGGTGCGCTCGCATGGCAGGTCAAAAAAGCCTAATAAAATTCTCGATTTTTGGTTCTCGGGGGAGTTCGCATGGGCACTTTCGCCTGGGTTCTCCCCCTCCTGTGTGTCCTCTCTTTCTTTTCGTTTCTCCATCTCTTTCTTTCTGTGCGTTTGATTAGCAATATCTGCAACTCGACATCACGCTTGCGAAACCGAGCAGAAGAAAGCTACGATGTGGAAATCTCTCGTTATATCATGGCAACATCACAAAGGGCAGTTGGGTAATTGTCTGCTTCCAAGTTTAGTCTTGTTTGGGGAGAGAGCATATGCAACGGTGGAGCAGCTGATCAGCAAGAGGAAAGGTGAAGGTGTATTCTTTGTTGCTGCTGGACGTGACTGATAGGAGGCTCCTTGGGGATTGGATGATTTCTTTGCTGTTGGTGGACCTGAAGAACAGTAGTGACGGAATAACTTTGGTTCGATGAATTCTTTGCTGCTGGTTGACCCGTTGATGGTTCACATTGCAATCCTCGGTTGGATGAATTCTTTGGATGTGGACGACATAGGGCAACGGGGATGAACTGTGTTTTATCAATTCATTTCTGCTTTTGCATCTTTGCCTTCCAGACAGACCCTCTCACCCGTAGGAGTTTAAAAAATCAATGCACGAGCAGGCTTATTATGCTGGTTCCTTGTTCGTAGGTTATGGCCAAACCCATGGCGTTTGTTTTCCTTCAAATAATATGCCTCTCGACAATGCTTTAACTAACAGGTCGGCGGCTTTTTACCACATGGTGGTGCAGTGGAGTTTGCGCGCTATCTATTCAAGCTTTGGAGTTCGTCTTTTGTTCTGGACTGTTTTTATCATGCGTTTCCAGAGTTGTTGCTGAATATTATTCCAGACTTCTTTTCTCT

>ONT.7077.2 LG04 87907127-87911385

TAAGAACAAGAAGGCAAAAGAGCCCTCCAAAACCTGTTTCCGTTCGCGCCTCTTCTACACCCAGCAGTCTTGCCCTCGTTATCGAGCACCCGATCATCGCTGGAGCCTACTCACCGAGCACTGTGGCAACAAATGGACATTGAATATACATTCTAGCAAATGCTTCATCTTTGCACATCACCGAAAGTTGTGATGATGTTATTATTGCATATGAATCTGACAGCTCGGTGGCGCAAAAGGTGATTTAGACCATGATGTAGTTACAAAGGTAGGTAACTATCAAGATGGAGTTACATTTTGGCAGTTAACCAAGATGACCAAGATGGAGTTGAAGACTTTTTGTAGTTAACTGGAACACTTGTTTTGAATCATTTTTGTCATTTGATACAACACACCGTGAATGGATGCTTTGTAGTTATGCTAAAACTATTAACTTTGAAGTGTTTATTGTTGAAGGATGAATATTTTGAAGGGTTGTGAACTTGGATGATAGAATGTTGACTTGTTGGATATTTGATTTGAATGATATGTTGCGTCTTTGT

>ONT.13019.1 LG08 53456822-53462834

AAGAGAGGTTTTCTCATCTCTCCCTCTCTTGTCGAGCGGTCTCTCTCTTTACTGTTATCTTCGCCCTCTCGATTAGCTCTCACTCTCTCTCTCGCTTTCTACTGCTATTCCTCTCTCCCTCTATTGTTCTCGCTGTCTTGATTTGCTTGGAGACTTAATCGAGGTATCTCTACTGTTCTCCCTCTCTCGATTAGCTCTCTCTCGCTACTGCTCTCCCTCTCGCTTCATCTTCTCCCACTGCAGCGCCGCCTCATGACGTCAGATTCGCTACAATCTCCACCCGAATCCGGAAAAGGGATGTCTTTGACAAGAAGGTCGGACCATCGCTGTGTCCCGGAGGTCTCTGTCGCTGCAGCCCAGCCCCTTGTCATGCAGGCCCTCCACAGGCCTTGACCCAGAGATCCCTGAGGATGCACTCGCGCTGGTAGAGGCGCCGACTGTCGGGGATTATGGATGGGGCCCGGCCGACCCGGATGTCAAGCCAGAGGGCGGAGATGATTTACCTGGGTTTTGAGAGCTCATCTTATTTTGGGTTTGAGCGAATTTTGTAGTGAGTTGGGTAGTAATCAAATATGTATTAACTTTTGATTAACAACTGATACATTTAACTTGACCACTGTTCCGCTCTT

>ONT.12805.1 LG08 3431764-3446805

GAGAGGCAGAAGCACAGAGGTGTTGGTCTTCGTGCACCTACCACTCAAAATAATGCGAGGTGCCTGGGAAACTATCAACGGAGCTTGAGCTCTACTACAGTCAGGCGCTTTTTACTTTCACAAGTAATTGGGCTTTTTTCTGCCCAAACAGGGTACCCTACCCATGTGAGCAACTGAAAAGATTCATTCAAATTTTTACTTCAATCATAAGCAGAGGTTAAAATAAGTCTTGCAAACCTTTTTCAATTACTTCCTTCTAGCTCTAGCTTGACTGGCTCTCATATACACCACAACTCATATCCGATTCATACTATCTCTCACAAACGGTTTGGGAAAACAGCAATCCGAATCAAAGTTCTGAGTATCTCCTCTTTGCTAAATTCTGATTAGGTGTGGATTGCTGGAGTTTGATAAAGAGTTATAGGGATATTGGCAACTTCTTAATGGTGAAGTTTAGGCTGTGTCATTTCAAGTTTAATAAAGCATTACAGGTGTGAATAGTTGGAGTCAGATAAAGAATAGTAGGGATCTTTGCACCTTCTTATTGGTCTGGGCAATTCATGGAGTTTAAAAACAAACAAAACATTCCTTCATGATGTTGTTTGAACTCATGGTTGTGTTTTTTGAGTCGCTTTATTTGGCTTTGGTTTAACTATGAATTTACTGGTGTAGTTGTACTGTGTGGATCTTGCTTGTCATCTTTGGTTTGTTCTTATTCACTTGTTTGGAACTAAAATGATTCTCTATTTTTACCTTCTGTATGTGAGGAAATTATATAGACCATGGATGTAATCCTAATGACTTGATTATCTTCAAGTGGGGCCCATGATTTTGTCATGGAGAGTGCTGATGAGATGAAATATTTGTGGAAAATTCATATCCTAAGAAATCTTGCCATGGGCAACTCGTAT

>ONT.4746.1 LG02 82710370-82712048

GGGGAAAAAGAAGAGGGAAAAGGAGGGGAAGAAAAAGGAGAAAGAAGAGAAAAATCGAGAAGATGAAGCAGCAACAGTAGCACATCTAGCGATTGTAGCTGGAGCGTCAGCAGATCTGCGAGGACCCAGCGATTGTAGCCGGAGCTTAAGTAGATCTGTGAGGACTAAAGACCGTGACATCTAGGAAATGAAAAAAAGGGTCCCACACATGCACATGTGAGGTCACACATGTGGGGCGTCCAAGAGGGGGGTCCACTTTCTTTGCACAAAATCCAAAGTTCATTTTTTGAAGATAATTCAAGATAATCAAAGAAAATCCATGAAGGTCCAAATCAAGAAAGAAGATTTCCAAAGAGACAAAAGTGGAAAGAATCTACACATATTGGGCATTCAATTTTCTCTTGATTCCAACTTAGAGTTTGCCACCTCATGCATCTCTCATAAATGTCAAGTTAAAGGAAGATTTGCTAAAGAAGGAAATATGACAAGTGCATGAAGATAGAATGGAGAAAATAAAGGAGAAGACTTACCAAGTGAAGAAGATTCTTGCAACAAAGTAGGGACTAAAAAGAAAGAATAGGGCAGCCCATACCCTATTTAAAGAAGCCTAGGACGTCCACTCCCTTTCATGAAGGAAAAAACCCCCTCACTGGTGTAATTCCTCCACCATTATACTCATCTCCTTTCTCTACCATAGCTCATCTCTCTTTAAGAAGGCTTTTCCCAACTTGATGCTTCAACTAGAGAGGTTCCTTTCATCCTAGTATGTAGTTGTTTTTTTTTTTTTTTTTATTTCTCCATCTTTTATGTAAAAACATTGCATATTAGTAAAAGTGTGATTTTT

>ONT.10127.1 LG06 26830298-26831208

GAAATGCCCTAATCTCAACAGATCCCTCCTTTTCATTCTCCTCTGTCCACTCCTTCTCTCCATAGCCAGCCAAGGCGATCACCGGGGAAGACGCATCTCTCCCCCTCCGCAGGGAGACGGAGGTCTCCATCTCGCCATTGCCAAGAGATTCTCCATCTTCCTTCATCTCTACCTCTCTCCACAGCTAGGCCAGACGATCCAAAAGGGGAAGACGTTGCAATCCCCTCCCAGCCCAATATCTCCCTCCCATAGAGAGACTCATCTCTCCCTCTCGCCACTGCCAAGAGGGAAAAGCCATGAGCCTTGCCGCTCTCCTTCGTATCTCCCTCTCTACAGCGATCTGCGCTAGCCCAGCTACGTTTGCCATCGCACAATCGATCAACCACGTGCTGGCCGGCCATTGCTATATCTCTCCTGCTTGAAGACTGTCAACCCAGTTGAAAGTGCAGATGTTGAAATCTGAGTGTTCAAACTGTTCATGTGGATTGGGGATGTTTGAAAAAAGAGTAAGTTGCATGTACTACAAATTTTACAGGTTTTCAGTTCTTTCGTGCCACACTTTGTTGTATTCTCTTTCGCATGGGATCAAAGGATTGGAGAAAGCTTCAAGCAATATTCAGAAATTGTAGTCGCTGAAAATTGTATTCACAGTTGTTAAATTATATTCAGAGATTTTTCTGGAAAAAATGTATTCTCTCAAATTTTTATCGAGGAATCTGTTTAGAAATGTATATTTGCAGTCACATTTGTACTCAGATTTTAATTAG

>ONT.6141.1 LG03 51709438-51710661

GATCTAATCGACCCTCTCTCTCTGCAGGTTCTCTCTCTCTCTCTCTCCCCCCCCCTGAATCAGCGACTGCAAGAAGCTTCTCTCGGACATCTCTCCCTGCCAGACGGCTGCCTCTCTCGTCTCTCTCTCCCGGACTGGAAGAAGCTTCTAATAGAGGGTTTTTTTTCTCTCTCTCTCTCCCCCGTTTTTCTCTCTCGCTTCCCTCTCACTCCCCTCTGCAAATCGCCTTTCGAGCTTGCTTCTGCCCCTCCAGACTACGCACGACCCTTCTTCTCTCCTCTCTTGACCAGACCAGAGAAAAAACTCATTCTCCTTTAAGCACTCTTTCCCTCCCTCTCCTGTTCTCTCCTTTCCTCTCTAGTTCTCTCCCTTCCTGTCTTTCTCCCTCTCTCTCTCTTTCAATAGTTTTTCTTCCTTCCCACATATCCTAATCTCAGATATCTACTCACAGATTGGGGTTTGCATTTAGATTTGGGATTCGGGATTTTAGGGTTTCTTATGGAGATTTGATTTCTGTGATCTGGAGTTTCTGTTGCAGATCATGTGAAAAAATTTCAATTTTTCTCAGTTTTAGAAGCCCGTTCTAAATTTGATCTGTATTTTGATGGTTTGATTTCTCTGCCATCCTTTGTACCATTGCCCACAAATGTGGTGTTAGGCGATTGAATGGAATAAGGGGGCAGTTTGGGAAATCAGTGGTGCACTTACCATTCGACTGCCCAAAGAAGTGGGCATGGAGGAGAGCTGTGGTAATGCCGGTTTGGGTTGAAAAAAAAAATCAAAACAGCATCCCATCTAATGTGATATTTGATTTGAAGTCCTTACTAATGCAGGCTCTGATTTTGAAATGCTTGGATTAAAAAATTGGTAAAGGTTGTGAAGGTAAAATGTCAACAAATTGTGCAGGCTTCAGAGCATTCAAGCATTGAACAAGTGAAGAATTTTAGGGCATGGGTTTTGTTGGTTTTGTGATTATGGATGTCTGTTTTGGTTATGAACTTGTGAGTGGTGTTTTTGAACTATTGCTTGGTCACTGTTCACTGTAACAGAAATGCATGAAGAGAATGAGGTTTCCTATGTATGTTCATGTTGACAAACAAGACAAGTAAAGAAAATATGAAGAGAATGAGGTTTCCT

>ONT.15413.3 LG10 11281029-11286409

GCACTAGCCGAGAGGGGAGATGAGGCGGGAAAGGTGTGGAGAGGAGGAGGAGATGAATAGGGAATAGGGAATAGGGAAGAGGAACGAGAAGGAGGGAAAGAGAGAAAGATGGGAGGAGAGGTGGAGAAGTAGAAGAGGAAGGATAACGATGCAGCAGCAGCAGCCGGAGATGGATGCCAGATGCAGCCCTTGAGGTCCAGCTGTCCAGTCCATTTCACAGATAAGTGCTGAAAAAGGATGACTAGGAGGATGATTTATATGGCTCATGTTGCTTGCATACTTGTAGGTTGAATGCAGAACTTGAACTTGTGTGTGTGTGTGTTTTTTCTCGATTGGTACTCTCCCGAATGTAATACTGCTTTTCAACGTGTAGCAGAGGTGGATGGCTAGAAGTTAAGAACTGAAGATTTTTGGGACACCGCAGTCTGTTGCGCGCTTTCCTTGGATACAATGAGTATACAGAATTATAATTGAAGTTGCTGTTGAAACCTGTTTGTGAAGCAGGAGCTTTTATTATTTGTTTGGTAAGGTTACCAAAAGGTTTGTTTAATTGCAACCTAATCTTTCATTTTCATACTTTTACATTAAAGTGTGTTGTATGCTTGTTCTACAGAAAATATTCATATTTGTGTTTGTAACTAGGTTTACATGTTTAGTGCTTCCTTGCCGGCATAAGAGACTGCTGTTTTGACCTTTCGGATCTGGGTAGTGTGAGGTTTTCTTATTGATAGTTCAGGGTTGTGGCATTTGATTTTGCATACTTTGCCAAAAATTAGGTGGGTGGTTGTGAGAGTGATTTGATCTGATTTACCTATCCTTGTTTGTCACACTGCATGGTGCAAGAATTTCTGTTTCGTCAAAATGTGTATGGCACATGATCTTGTAGCAACAGATTTCGGCAAAAAATGGAATGGTCTAATGAACTAAATGAAGAGTAAAGGATGACTACTCTCTTCTGGCCAGCAGGAAGAGGTATTGGTGTCTCAATCTGCTTAGACGCGGG

>ONT.15742.1 LG10 27899263-27903358

AGCCTCTGAAGTCTGTGAGCTGCTGCCAGTGCCACCATGTCTTGTCCGACCGCTCTCCTCTCTAGGGTTTCTAATTCTGCCTCTCGATCTCCTTCTCTCTTTCTCAGATTACGAACCAAAACCCTAACCCCCCAATTCTCCCCTTCTTCTTCTTCTCCCATCTCTCGATCGCGACTCCCCGATTCCACCAAACGATTCTATCTCATTTCCAGATTACCTTTGAATTTGCGCTGTTTGGGATCGATGCTGCCATTGCACAGTGCCATTGCTTCCGCCCGTCTTAATTCAATCCTTTCTGCGGAATCCCAGAGCTGGGGTTTGATTCCCCAAGGCAATTCAATGCCTTTATGATGGTATTGCAACATTACCTATTTGGCACCTGACAGATCACTGTGTTTGGTGGGACAGATGATCCGCAAGACCTTATTTACTCAATTTTGTTTTTAAAACTTAACAAATGCATTATTATGGCTGTGATGATTTACAGTTCTTTATTCCTTTGTTTATCTCTGAAACCCTCTCTTGATTATATTCAACTTTGTTATGGGGAAAAGATTTAACCATGAACTTTTTTAAGTGAATGTGAAAATCAAAAGTGAATGTGAAAATCAAGATGTTTTCCAGC

>ONT.7797.2 LG04 87730725-87731612

GTGACTGGGTACATTTCTTACTTTCAGGGATCATAGCTTCCTTTCTTGAGTGGAGAATGACATTTCTACAGCTTTTCCATCCAAGATGACTTTCACTTTATGATAAATTCTCACTTTATGATAAATTTGCGCAGAGAGCCATGACAATCTTATCAGCCATTTCATCATCCACCCTCCTCACCATCAATCTCTCTCACACAGCTTCAACAGACATGGAAATCTCTCTCCAACAGCTTCAACAGGCATGAAAATCTCCATAAGCCCATCTCCTCTTTCAGAATCAAAGCTGCAAAGCTGCCTCTAGGAACTCTACAGTTGATGGAAGATATATAAAAACTTCAAATGGGTCATGGAATTTTCAGGTGCAAATGCCTAAAGTAGAGCCCCAGCTCACTCCCCATTTCTTGGATTTACTAAAACAGCTGAGATATGGAATTCCAGAGCATGTATGATTAGCCTCATTGGGACTTTCATAGTTGAGCTGGCGGGTTCGTTTTTTACCATTTTATGATTTAATACCATTTTTAAGATTGCAATTGAATACTGGGGGCAGAGAGTGAATTGTTATCCATTCATTGTTTGGAGTTAAGCTGAATTATGAAACCACAAGAGAAAGAACCCAACCTTTTTGCCTTTTCTTGCCCCCAAATTATAGTAAGTGCATATCGGAAATGGAAGCAGTTTGTCGAAATCTCTCTGTTTTAATGTTTCTGTTCCACTGCGTTCATATCTAGTTTCATGATTGAGTACATTGTCAGAACTTGAAGTGATTATGTTATCAATTTCTGTA

>ONT.712.5 Contig01982 110531-117066

AATCTAATCTTCCGTCGCCAAACCACAAGTAGAGCTGCCGCCCTCTCTCCTCATTCCGTCTCCGACTCTCTCTCCTGCTCCGGCTCCAGCCCTTTCTCCCGTTTCCAGCTCCGGCCTTCTCTCCTCATTCTTGATTTCGCTACTGTAGTGCTGTTACAGAAGAAGCATTAATGTAGTGAATGTAAGGACGGATGAAGTCAACATAAGGACTCAGCGGTATAGGAAAATGCATTTGATGCGCTCCATTCTAAGGGTGTTGGAAGCAAAGCAGGCAATTAGTCTGTCTCCACTTTTGTAGTTATATGCTGTTCAAATCTAACACAAAGTTTATATCTATCTAAAGAACTTCTGATGGAACTGGAGAGGAGAGAGTAAGAGAGAGGAATACTTCCTGATCCTGATCTGGACACCTACATGAAGGCTACATCGGTTGAGGACTCAGCGGCAAGTCTTCAAAGTGACTATATTTGGAAGGTTCTTGGACTGGAGTCTTGCTTGGGAGTGATTTTAGTGTAGGTGATGCTATGAGACGAGGTGTATCAGGTGGAGAGAAGAAACGAGTTACTCAAGGGGAAATGATAGTTCAATCTTTTCGATGAAATTATCATAATGGCAGAAGGGAGGATCATCTACCATGGTCCTTGTGATTGTTTTTGAGTTTTTTTGAAAATTGTGGTTTCAGGTGCCTAGAAAGGAAGAGCCCTGCCAATTTCCTCGCTGAGGTGATTTCGAAGAAAGATCAAGCGCAATACTGGGCTCACTATGGTCAACCATATCATTATGTCTCTGTTGAACAATTTGCAGAATTATTCAAGGAACATTATTTGGGTCAAAAGTTATCAAAAGAGCTTTCACAGCCATGCTCTTTTCTCAGAAGTTGCAAGGTGCTTTATCCTTTACTAAGTACTCTCTGAGTAAAAAAGCAACTTTTGAGAGCTTGCACTAGTAGAGAATGGCTTCTTGTGAAGAGAAATTTGTTTGTATATGTTTTCAAATCGATCTAGGTAAATATCATAAGTGAATAAAAGAATCCTTGTCAAAAGAATCCAGGTTATATGAGCTCTCT

>ONT.1635.11 LG01 118121160-118122172

ATCCCAAGGTCTCTGGTCTCAAACCTCTCAAAATGGTGAGTCATAGATCTTTTACCATTGTCTTCCTGGTGTTGTTGGGTGCAGGCATATGTTCTGCAGCTAGAACGCTCCTTGCCTTTGAAGGAGCCCATGGTGCGGGCCACTTCGTCGACAGTGCCGCATCTGGAGGCTACGGTGAGGGTGGACCGGCCGGTGGCTATGGAGCTGGTGGCGGCGGTGGAGCTGGTGGTGGCCATGGCGGCTACACTCCATGAGAAGATAAGCATGTTTAAGAAGAAGGTGGTACTTCTAAGCAGTAGTATTAATAAGAAGTGTGATTATGATAAGATGGTCAGTACGTAAGTGTAAAAGTTTCAAGCGCAGTGTGATAAAAGTATCTCAGTGCTGCATTGTAATATCTATGTGATTGTATGAAATGAAAGATCTCTGTAGTGCTTGCTTGATTTCTGCTA

>ONT.159.2 Contig00448 275583-276516

AGGCAGTTTCTAACTTCTCAATTACAACTTCAAGACCACTTCAACTGAAGCTGATTTTCCATGGACATTCAATTTAGTCTTGGGAGCTCTGATAAGTCACCACCGCCCCAGCCTAATAACACGACCAAAAATGTCCAGACTGGTGGGGGCAAGTACAACCCACAAGTCCAAGGGGGGAACAAAGCAGATGGAGGTGGCAACATCAATATGGGACCTCAAATATCTGATGGTGGTAACTTTACTATTAATTGAAAAAATATCTTCCCCCTCTAAATGAGGATGTTGAAGCTACTACCATCCGTGCTCAAGTCTTTAAGCTTTGTCTACTTTCAACTAAGTCAGGGTCCTCCAGTTACTGAAAGCGCCTCCCGCCTCCCAAAGTCACAAATAATACTGGAAGTAATGTTAGCCTCTGTTTACACAAAATAAATAAAAAATAAAAAAATAGTTATCTGAATCGTTTCTGTACTTTTGTGGATCTCAAGTAGTTATTGTGTAATGCTTGCTTGCTGGTAAGTCAAAGTGGTGTTATAT

>ONT.7801.3 LG04 88007019-88007946

ACAGGACAACCTCTTACTTCTCTGTGGCAACTTCAAGACCACTTGTAAAATATGGATTGCAACACGGTATGGTAATAATTCGAGAGGGAACAACCCCCAAATCCAAGGGGGTAACATCAATCAAGGCAATACCCAGACTGGTGGGGGACGGAACATCAATACGGGAAATATAGTACGTAATCGTGGTAACTTCAATTCGGGAAATCAAGTGCGTCGTGGTGGTGAAATCAATACGAGAAATGAAGAGAATGGTGGTGATAACAACATTTCGAACAATGAAGTCAGTGATGGTGGTAACATGAATTCGGGAAATCAAGTAAGTGGTGGTGGTAGCATCAATTCGGGAATGGGTAAAGCTACCGGCTGCTGTATGATAGTATGATACAATGCAAGTGGGTGGAGCCCAACATCATTTCTGCATTTTGTGGATTCCAAGTGGCTATTGTGTAATGGTTATTGCTTGCTTATTAATAAAAGTTTGTATTAT

>ONT.5138.4 LG02 124639765-124640485

ATCCAGTGGAGTAAGGGTTGTGAATATCTATGTAGAGGTGCTGAAGCAAGAATGCGCAAGTTGCACATGTGGGAGCAAACCTTTAGTGTGATCTTATGATGATGACGACGACGACGAAGACCATTATGATGAAGAAAAAATGGAGTGGTTTGGTTGAGAATTCTGGTGGCAGCTGGTGTTGTGGAAACTGAAACAGCTCCCCAAGGAACCACCACTTTGTGTACTGTGTTGTGTACATATAAGAATCCCTTTAGATATGTCTTTGATTGCTGGAGGGTCATGAGGAAGTTTCTGCTCAAAAGAGAGCTTGTAATTAAAACAGATTTTGGATACTATGTTCTCTATATCAGTTAGTTTGTGCTTTTAACTTCTCAGGCATAATACATGAATTGCCTTTGATGATTTAAATTATAATAAGGTCTTGACTCTTG

>ONT.8641.1 LG05 84992225-84994417

AACATTCATTTCACTCAACAACCATAGCACAGTAGCCGACTCTTCTTCGCTTCCTCTCTTCCTTTCATTTTCTTCTTCTTCGTCGACGAGCACCGGAGCTCCGGCAAAGGAAACCTAGCCACACATCATCTTCCTCAAGCCTTCAAGATCACAACCATACTCTTTTCTTCTTGTTTCATCCACAAACCATTTAAAGGAGCTAGTTGGGTTTGGAGAGGTGTTTCACCTTTTTGGAGCTTCAAAGTGTTTTCCTTCTTCACTTTCTTCTTGATTTTGAGTCTAGCACATCACAAAGGTACCGAGCTTTACTATCTCGAGGCTGGAAGAGACGACGAAAAAAGTTCAGGCAGATCAACTTGATGACTTCCTAAAGTATAGTACGGATGACATTGTATAGCCGTGATTACCCCGTACACGAGAGTTTCACGATTATCATCCTAGCTTCATCTTTTGGTTTCTTTATACTTTTGTTTAGGCCTAGTTTTAGTTTGGCCTTTTTACTTGTAATTAATATGTTGTAAAGAGTCTTAAACTTTAACCCTAACTTTGATATTCAATATATTGCTCGTAAACTACACATATTTAAG

>ONT.1507.2 LG01 109766853-109798878

GTCTCTCTCTCTATGTGGGGTAACCCGTATCGTCTCAAGTGACTGGAATCGAAAAGAGGAATTCATTGCAGAGGGGGTTTTCTCGAAAGCGAGAAGTGAAGGTTCTGTTGGTTCCTTGGGAAGTTGCTAGTATTGTTTTGAGCTCTGATGCCCTGATACGACATAGATACAACATGGACATGCCAAGGATATGATAGGTGGATTACTTGATAGAAGGGACAGGGGTGGCTGCTGCCATATTGCTTTTTGGGTTTTGAGTCAGGACGTGGATAAATTTCAACAGAAAGCTCAAAATGAGAAGTTCTGCACTTTCTTCCCTGGTGGCAAAGCTGAAAGAAATGTTCAACAGAGAAAACGGATAATTGTAAATGTTGATGTGATTTATACTATTTTCAACAGATGCATGTATTTCATCTCCCCAGTGGTTGGATGAGAGTTCCTAATTCTGAGTTTTCCCTAATATCTGTTTATCAAAGTACTTGTTTTACATGTTCTTTTGTTGAAAGACATGACACAACTATCAGGGACCCTTTTGTAGGCCATTTGCAAGGATAACCAACTTTCATAAATGTTCAATGCTCGCTCATTGCTATC

>ONT.12803.2 LG08 2374452-2399814

GCGCTGAAAAGAAAGGGCTTTGGAGAACCCTTTTGCTGCTGCTGGCCAACGAAGCCGAGGAGGAGGGGCGAAGGTTCTATACGCCGCGGCTGTCTGGCTGCTGCCTGCTGCCTGCTGGAGGTGACGCTGCTGCTCAAGTGGTGGCGACGAAGCAGGTGATAGCACCGCTACCCTCGCCCCGGAGGATGAGACAGTCGACGAGGCACCATCAGGTTTTTAGCTGAGTGGACCTCGAATGATGTCATGTAGACAATTGCTTGGGCGCGAGTGACCTCACATTACTTCATGGAGATCCAGAAGAAGAAAATTGATGCAGGTCTTATTGGGAGATCAGTAGATAGAAGCGTGAAATGAACTCAAATGGTGAGGAGAATTGGGCATAGGTGATGGTGGGGAGATTTGGTCATAGGTGAAGTCCAATGGAGATTGGGTCTATAATGGTGGCAGTGGCGGACCTAAGTGTCCGCAGGCGTGTGCAGTCGCACCCGTGAGATTCGAGAAACTCTTTACATAATACCTTAAAAGTTTTTATATTATTATTTAATATTTTCACTTGATTATTGTCCGCACCACCCAAATGAAAATCCTGGTATGCCGTTGACCGGTGGTGGTGATGGAGACTGATGGAGAACTTAGATTTTTAAAAAGTTTTACATCAGAATTGGTGTAATGGGGAATTATTGAAGTGAGTGAATATTTGAATAATTCTTTGACTGGGTATTGTACAAAGGAAATCCTATAGTTGTTAAAGATGTCGGTGTTTGCTAGAGGTACAA

>ONT.8960.1 LG05 3642341-3649217

GTTTAGAATTTAAGGAAAAAAAAAAATCCTCGGAATTCTACACTCTCAAATCCAACATGTACATGTGCGTCAGGGACCCGAGCCGACATGTTTACATCCCGTAAAGCAATGGATCCTTGTGTCCTTTTCAAAGGCGAGTTTCTCAATCCTCTGGCTCTTCCTCCGTGACTTCATCTTCTTCGCCGTATCCTTGTGCGCAGAGTCGCAATTCAATCCTTGTCAGCCATCGATAGAAAGTGAATCCATTGCTTAAACATATATACGGAATGTGAGGTCAATCCAACTCCCTCCAAACCCCAGATAACGCAAATTTAAGAAGATGGCAGAGATGGGAAGTTAGTGTCCATGATCAGTACTCAAATCTTTGGCAGCTGAATCAAGACTCTTGTACACTTCTTTCTCAACTTTCAATTTTCAAGTCCTCCATTGGAATTATTGGTCAAGAGAGACCAATGAATGACTCAAGAGTTTTGGTTGGTGTGAATGTGCTTGAAGATGCAGCCGTCCGGACAGTGTACAGTCCTATAAACAATGTGTGGTTGGTTAGAAGATGTTGTTCAGACCTTGTACATCTTGCTGAAGCATTTTATTGGAAGGGCATATAAGTTTTATTAAGCAAACTTATAATGTTGTTCGTATTGTCCCTTTTTGTAAGGACGAGCCAGCTTTTTTTTTGGGCTATATGTTGGAAACTTTTTCAATGATGATAATAATATATATAATGTTGTCTGTATTGTCCCGT

>ONT.12458.1 LG08 47469363-47476649

GAGTACTCACGCCATCCCTCTCTGCTCACTCCCTGCTCATGCATCCCTCTCTTCCTCAGCGATTTTCCTTATCGAGTAAATACTCCTCGCTGGATATAAAGCAACAAAGATCGCCCCCCCCCCCCCACACACACAAAAAAATCTATCATCTAGGGTTTTCTCTCGAGCTCTTCATCTTTCGTTTCAGGGAAGGAGGAAATGTTACCCATTCCTGAAGGTATTCAATCAAATGATGATTTATTACCAAAGGATATATAGGCAATTCCATTCAGACCGCTCTCTCAAAGACTCAGATGATTGCCTGATGACTGAATGATGAAGATGAAGTGAGAGAGGAAGGAGGAGAGAGTGACTTGTGGGATTGAATCTTTATGAGAGACATCCAGAGGGCGGGCCATGCACCGTGCAGAAGGTGTTGAGTCCCACCAAAGTTTTCCAACGACAGTTCTTTATGCAATAAAACAATGTGTCCACAAAATGAGCATGTGCTCTAGCAAACTGAAGCTTGCAGTTGGGAGGTTCCCATGGTCCTCATGTTCATGGATTATAAACATCAACTTTGCTGGTTAATTGACACCAAAAGAGTTTATAAAAACAGCTCTTCCATCACAACTGAGGAAGATCTCCATGAGGAAAAAAAGACGACTGACATTTCTTCAGCTTGGGCATAGGGATAGTGGCAGCTTCTTGAGTTAGAGCAACTAAAGCCCCGGACATATCAACTTCTTGGAGTTCACCCTTTTCTTGCATGTACTCAAGAGGTAGTAAAATTAGATCAAAGACGTGCAACTTATCCAAGGATTTGATTCAAGATAGATCGGAATGGAGAAACAAAATTCATGTAGCCGACCCCAACATAGGTGGGACAAGTCTTTGATGATGGTGATGATGAAATAACTCTGAGGAAGTAGATCGTAGCTGCGTCATTATAGCGTGTAAAAGATTATGGGCATAATTTTGGAATTGTTATTATTATTTTTATATTTTTAATTTGAATGGAATTTTGAAGTGAAGATTTTAGTTTTCCTCCTAGGGATTACCTTGTTTATTTCAATCACAGACATTTGTGGATGTTAAAGATTTTGGAAAATGATCAAATAATAATATTTGTGTATTTTAGAGA

>ONT.455.1 Contig01277 57148-58211

GTACAGGCAGTTTCTAACTTCTCAATTACAACTTCAAGGCCACTTCAACTTAAGCTGATTTTCCATGGTCTCTCTCTCTCTCCCTCTTCAATTACAACTTCAAGGCCACTGCAACTTAAGCTGATTTTCCATGGGCATTCAATTTAGTCTTGGGAGCTCTGATAAGTCACCACCACCCCAGCCTAATAACACGACCAAAAATGCCCAGACTGGTGGGAGCAAGTACAACCCACAAGTCCAAGGGGGGAACAAAGCAGACGGAGGTGGCAACATCAATATGGGACCTCAAATATCTGATGGTGGTAACTTTACTATCAATTTAAAAAATTTCTTCCCCCTCTAAATGAGGATATTGAAGCTACTACCATCCGTGCTCAAGTCTTTAAGCTTTGTCTACTTTCAACTAAGTCAGGGTCCTCCAGTTACTGAAAGCGCCTCCGGCCTCCCAAAGTCATAAATAATACTGGAAGTAATGTTAGCCTCTGTTTACACAAAATAAAAATAAAAATAAAAAAATAGTAATCTGCATCGTTTCTGTATTTTTGTGGATCTCAAGTAGTTATTGTGTAATGCTTGCTTGCTGATTAGTCAAAGTGGTGTTACATATATATGCCATATAGCAGC

>ONT.5302.1 LG03 23748879-23782624

AGGGGATCCACTTCCTGCGAAGCTGCACACCCTCTGCTCGAATCATGACCTCTTCTTCTGTTCCGTTTATTTCCATCGCTTTTTTAACTTTTCCATCTTTCCTTCTTCTTTCTCTCCTGTCTGTCTCGAAGTAAAATCATGAGATCACGTTCTTCACTTGCAAAACAAAATCCCCATCAATCTTTTGCTCTCATCTTCTTTACAAATCTGAGATTATCATTAGAGAAAGAACAGACAATTAATTACAATATGTTGATGGGTATTATTTCCCTTCAGTCTCTGATTGACATGATTTTAGCAGGGATTTCTCTCATGTTTGGATTGGGAATCTTCGCTTTCATAGCTGCCATTCTCTGTTCTGCTGCTTTCTTGCAAAATGCAAAGAACGTCTCTTAGAGAATCACACTTCGAGGTTCTATTTCCTTCGTCCCGAGATAAGAGTTTTTGGTTGGCTATGGAGCCATGTCTCGCAGGTTGTTTGTCGGCTATGTCATGTAGACATTGGGCTTGACATCGGAGCTACGTCATATATTGGGTAGTGGTAAATAGCTTATTGTTGTATGGCTTTTATTAGCCTTTTCTTGGGATTTTTAATTAATGTACATTGTTAATTTGTAACTTGAACACACTTGGGAATGAAATATCCTGTTACTGTC

>ONT.11301.1 LG07 24094564-24106959

ATCCAACAACATAGGATTTAATCTTGTTTTCTCTTTCTCATTTCTTCTCCCCTTCCATGTTGGAAATCATCTACTAGTGTGCCCCGCATGCAAAATCGACCCTACTAAGCAATTCTATGTCATCTCCTTCACAATATACAACGTTTTCGCGAGGATCCATGAAAGGCAGCCCCTTGAACCATCTTCTCCTCTTTCTTTGAGCTTAATAAAGGAGGCGGTTGCTGAGCTGCAGGTGACATGTGACTAAGGTGCAGAGATTATCATGTAGACTTCATTCCTTACACATGAGACGCGAAATACCTGAAAATATTATTTTGGATTTTATATTTTGTGGACTTATTGGTCTCTGTTGGCCTTTTAATTTTTGGGAGTATAATTAATGCATCTTCAGTAATTTCTAACTTGAAATATGAGAATAGTTTATCATGTTTACTTGTCTAGC

>ONT.4106.2 LG02 1698052-1705505

TTGTTGGCCTCCTAAGTCCTAACCGGAAACCTTTCTCGCTGGCTGCCCCCTCTCATCGATCCACCGCCGGACCTCGAGCCATCGATCGCCGAAGCCGTCTTGTCTCTCAGCCCTAAGGTACCGTAAGAGTGAAGGCACAAGAATGGCTTTTGTGATGAGGTGAAGTCTTTGAAGAGTCTTCATGAGTTCCAGAAACAGTTAGGCCAACCAGATGGATTTCCATGGTACACATAGAAACCATGGTGTTGGCCAATCAAGTGAGACATTAACATATGTCAGAAAAGAGAAATCTGGAACACTCCTTAGTGAATACATTGCTTTTGAGGGCAATTCCTGGGCTGAATGAATTTGGCAAGGCTTTATTAGGTGAATGAAGAACTTAATTAAACGGAGATGGAATGCCTTTTGCTAAGCGGATATACTAATTATGTTAAGTTAATTTTGGCTAGTTAGGGATTTCCGGAAATTGTTTACAATGTAGCTTGCCTTTTTTTGGATGCTTTTGTGAAGGTTTTGGATTATGTCGTGACGCTTTGGTTACGAATTCGAAGGTTAATGTAAATAGTTTGGATTATGGATGGATTCAAATTGGCTATGTTTTGAATAGTTTGTTTGATTTAGTGCGTGGACGAGCAAGTACGTTCTTTAATGTGAATGGTGATTAATTTCTAATTAATGAAATGGTTCTTATTGAA

>ONT.15060.1 LG10 74763605-74791718

GTCTTTGCCGCGAAAACAATGAAATCGTTGCTCTCTTCCCGCCAGCTTCTCCAGCTAGGGTTGGGTTAGGTTAGGGCTTCGTGTAGAAAAAAGAGAGACTGGAAGATGATTGAATTTGAGGTGGTGTTGTAAGAGACGACACGATCGTCGATCAGAAGAAGCTGATGCTGCACAGACCGGAATGAGAGAGAGAGAGAGAGAGCAGACAAGATCCTAATCCACAACTTGCATTGCATGCAACCGATGGAGCTCTTCTATAACTACTAGGTACCAGATGCTTTCAATTGCAGGTCTGTTCTGGGCATGTAAACGTTTGCAGAGTATGTAGCATGGAATTGTCAGTTGCAGATGGCAATTTTCTTTCATTGACATGCCAACGTCAAATAGAGTATGTAGCATGGAATTGTCCGTGGCTGATCTTTTTGTAAAATAGTATCAACCAGTAATAGTTCCCTTTGATAAAGTATCATCATAGATCTTGAATTGGAGTTAGAGTTGAGTACTGTAAACATGTAATGAATTTTTTAGTTGATCTATCAGATAAACAGGCTGGATTAGATCCTGGCAGTACAAGAACCTTTGCATCCTTGTCCTTCTGAGTAGTACTGTATTATGATCTGTCTAATGTGATATTACCATCATGATGTAAAGCAATCACCTTAGCTATGCAGATGGAGGCCATATGGGGTGAGGCCCATTTACA

>ONT.15066.1 LG10 75828875-75830019

GTTTTAGATAAAGCTTCCGAGTAGACCTACGATTCATTTGTAGTGAATGAGCGTGGGTGCATCAGATCCAAGCGAAAACAATATATCAGATTTCAAACACTTGTGCTCTCCACTTTCTGCAAAAATGTAGCTGCACGAATGTCATAATGTGGTACCAAAGAAGCGCGGAAAAGAAAAAGGACAGAACGGCCACTTTTCCTCATGTAAAAGCGGAAAAGGTAACTGAGTTTCCTGCGGTTCCATGGAAAACCAGTTCCTGTGGAGAACCTTCTCTGCAGATCTGTGTATCTGTAGTGCTGCCCGTTCTCTATCCAGCTCTACGTTCGTCATCAGGCCAGGTCCCTGAAAGTTGGTCAGTCTCCAAATAGCAAGTCCAAACTTCAATGTCATAAGCAAGCAAGCATATTGGTCCTTCACTAAAGTTTAACTAATCTCCTTTGTGATGCCGCACACGCCAACCTGCCCTTCAATATACAAACAGGTCCAGTTCTAGGCCAAACATTAAGTTGGACCCATTCTGTGACCTGATTCAAAAGATCAGGAAAGCCAGGCAATCATTACATTGGCATTGAGGAATCCAAATTTTAATGCAGGGATCCAACTGTCAAGTTGTCTTGTAGACTTGATTTTTGGACAGATCAGATCCGATGCAGTGTACACCAGTTGAGCGGCTCGTGTATGTAAACACCATTGTCCATTGATGGTACTCAGAGGAAGTATAAGGTAGAAGACTAAAAAGACCAAGGGAGTGGAGCTATAAAAGTTTTCAAGTTACTAAAGTACAGTGCAAATAAACTTCTGTAATGGAAATTCATACATATTTGTGCTCTTTTATCCCAGAGAATAATGGTAATGAGGCACTGTTTATCCTCATAAAGGAATTGTTTCTC

>ONT.9479.1 LG05 36980435-36983792

CATTTCCTCCACCCCAACGTCTGCAACTCCATAGCTACAGTGCCAGCAGCCCCTCTTCTCCATTTCTTCCATTTCTCTTGCTCTAATGGAGCCTCCAAGCCACCAATCCGACGAGGGAACTCCGGCAACTTCTTCTCCTTCATCTCCTCTCTTGTTTGACACCATTTTTGTGGGAAACCAAGGTGGGCAGCCCCTATTGCAAACTTCTTCTTGTTGATTCATGACTCTAGAGAGATTGTTGATCTTGAGCTTGAGATGTGATTAGAAGCTTGATTTGCTAACTTGGGACCCAAGGCAAGTTCAGACTTCTTTGCCCCGAGTTGAGTGTTTTGTTGGCTGTGGAGTCATGCTTTGCAGGTTGGTTTGTCGGCTATGTCATGTAGACATTGAGCTTGACATCGGAGCTACGTCACATTTAGGTAGTAGTTAATAGCTAGTTGTTGTATGGCTTTTATTAGCCTTCCTTGGGATTCTTAATTAATGTACATTGTTAAATTGTAACTTGAATATATTTGAAAATGTAATATCCTGTTACTGTCAGCTCTGTAA

>ONT.11926.1 LG07 53091462-53093718

ATCATTTGAGTCATTTCTCCTCAACCATGCCAGACCTAACCTTTCCTTGCTAGCCTCAATCCACTCGCCCATTCAAGTGCCTTACCCATTCCCTCAATCCCGTCGCCCTCAGGACGCCACATTCGACTACGCCCACAATCCCCTCTGCCAAGATCGGTGATGCCGCCCTCCACGCCCACAAGCTCCTCTCCCAAGGTCAGCAATGCCACCTTCGACTGCTACGGATTCCAGCTGTCCTAGCCCAAAGAAGAGCCACCTCGAGAGCCTTCATGAACGAGCGATCTCGACGGATTCCAGTGGTGTCCTAGGGACAAAGAGGTTGAGGAGGTCTTCTAATTTGGATGGATGAAGTTGCGTTGGAATTGATTCCTAACTCTAATTCAAGATGTGGCCAAATTAAAGCGAAAATGAAAGAAATGAAAAGCTCTATTGAAGAAATAAGACTTGAAATTCAACGTCTAAGACGGTTTAGAGCATTAAAATATGGGAAGCAGACGCAAACATTGATTTCCGTCATTGATTGTCATTGTTCTATTTTTTTCTTCATTTATTTGGTGTTTGAAAAATGGATGTGACAGTCAATTGGAGTTGATGCCATTATTGTGCTTAATGGTTAGGCTTGCTTTGATGTAACTAATTTGACCCGATTATGTTAATAGATCCAGCTTGAAATTTCT

>ONT.10669.2 LG06 38763043-38781369

GAGTCGCCTGCCCCCATCCACTTGCATTATGAAAGAGAGTGGCCCCCTTAAATCAACATCAGCCAGCTGAGCTCCGCCTCCACTTCAAACCCTAACATCGCTCCGCGAACCAAAATCCCGCAACCACTACCAAATACACCCAGGAGCACTCTCCATTCTCCAGTGCAAGGCTCCAAGTGGAAAGCAGATATTGGACACAAGTAGTCAAGGGGAGCTTCAGTTGGTGATATCTGATGTACCATTGCGTATGTATGTTAGTTGAAGTTAATATATATTGGAAAAGACACTAGAAGAGCAGTAAGAAAAGACTCCAGAATAGCAGCAGCTTCAAGTTTATTCATGTAGGTAAAATGGAGCAACAAGTAAATTGCTCTTATCTAGCCAGCCTATCACCAGAGGATTTCCAAGATCTGGAGATTGATATTCAATCAGCAGGTGAGTTTTCTAGCCCAGTGGATATTGATCTTCTCGAGTGGAAAGCTGCTTATGATGGAAGATATGCATGCACTTAACAAGGATAAAACATTGGATATTTGTTGATTTTGTAGAAGAAATGTGTGTGGTAGGATGTAAGTGGGTGTATACTGTGAAGTGTGATTTGGATGGTATAATTCTATGAAAAATCAAGGCTAGTGGTAAAAGAATTTACTAACTTATG

>ONT.12803.3 LG08 2397860-2399814

GCGCTGAAAAGAAAGGGCTTTGGAGAACCCTTTTGCTGCTGCTGGCCAACGAAGCCGAGGAGGAGGGGCGAAGGTTCTATACGCCGCGGCTGTCTGGCTGCTGCCTGCTGCCTGCTGGAGGTGACGCTGCTGCTCAAGTGGTGGCGACGAAGCAGGTGATAGCACCGCTACCCTCGCCCCGGAGGATGAGACAGTCGACGAGGCACCATCAGGTTTTTAGCTGAGTGGACCTCGAATGATGTCATGTAGACAATTGCTTGGGCGCGAGTGACCTCACATTATGGATAGTTGCTTTTTTTTCATAGCATTTGTTATTGGCCATTGTTGGCCGTTGTTCGATCGGGATTGTAACTAAAATACTATTATAACTGTTTGAATCTAATGCTTCCACAC

>ONT.11369.2 LG07 62850372-62860400

CAAATCCCTCATCTTTCATTTTGCGCCCTAAATACCGATGTATGTCTCTCGCTGGCGTCTCATGTGAAGGCACTCAGACCTCTCCCTTCCGACAACTTCTCATGGAGACTTCAGATCTGAATCAGAAAAGGAGGAGTAATAGATCCAGGCTAAAACTTCTTCGAAGCCATGGCAAGGTGAATGGTCACTATGAAAGCTGAACACAGAGTCATCATGGGTGGTTGCTGGTATGCAAAAATGTGTTAGCAAAGCAGGTTTTTGAGGGAAGGGCCTCAGAGAGTCTATAAGATCGTTGCTATATATTGATTGGTGGCCGATCTTTGGAATCAAAACTGCTTAAGAATATTGTCCCACCTTGTTCTACCATCAAAGAAAAATTCACTACAATTAAAGGTCTGTCCATTCTCAAAATTATCACATCTAAAAATTGCGATTTGATTCTTTAAACATGATCAATCTTATGGTGGTCATAATTCTAAGGGTAAGTTTGTTTAATGAAAGGATTCTGGGATAGAGGAAAATTGTTTCGATCTGTCATGCATCCCACTCTTTCATCCTTTGGTGCCAAGTTTTTAGGATGGGGCAAAAGAAAGAAAATCAGGATTAATTTTCCTCTATAGAGACAGTATCCATTTCCCAAGGGAGGGGGAATGCAAGTTCCTCTTTCCCATCGGGTTGGAATTCTGTGGGGTGTGGAATCCTCTTGTTACACAATACACCCTAATTGGAGTGAAGTTTTCCTTGATAATAGAAGAAGGCGGGCACTGTTTTTGAGCAGTTTGAATTTTAAATAGTAGGCCGAACAGTCGGAATAACACACATGGACCTGTACTCCTCCACCCTCGAGTCTGAGACTTCTGAGGGGGGCCAAATATTCTCTGAGCTCATTGCTACATACCCATCAGTAGGCCCAGCTTTGGAATCCAAACCGCATAAGGTAATGTCACCATTGCCCTTGAGGAGGAGTGCGAGGAGGTGGCACAAGTGCCATAACCCGTAGTCGAATGGAGTGCAGACGATGCCATTTAGACATTAGGGACTGCTCGCGAGTTGAGGATCATGTTTTGAGAGATGTCTTCGTATTTTGGGAATGTATTGGTCTATGTTGACCATTTTATTTTGGAATTGTATTTAGAACTTACTTTATGTAAATATTCAACTTAAATGCTA

>ONT.68.1 Contig00205 90954-93127

GCCCAAAGCTTTCGTCTTTCCCGCTCGTCTCTTCCCCATCTCTTGTATCTCTGCCGTCTCTCAATCACCAATCTCCTTGCAGGTGACGGCTCTCATCCCTTTCTCCTTCCAGGCGAGACATTGTAGAGGTCAACATCTGATGACAAGGCATTGAACAATTGCAGCTCTATTTTGTAGGCTTTAAATGAAGAGTGGTTGACATGCTTTTGCCGTTGCATGTGGTTGTGATTGGCATGAGAAGTTGAATGTTTGTTTTGGATGCTACCCATTGTATGGAATTAACGGCAATTGGATTTTGTAATGTTGGTGGTTGTTAGAGGTTGCTTAGTTCTATCCTTTTTTGTTTTTTTATGGGATTTGATGTTGGAGGTTGTTATTAATGTAATTTGCTTAGTTCTATTGGATTTTGGGCCCGACACCAATGTCTCTAAGTTATTTGATTTTATGTTGGATGCTGGAAATTGGTGTTAGATGTTGGATTTTGCAAATGTGCTTTGAAGCTGTTGAAATATTGCCTTTTTACAAATGGATAGTCCAGTATTATAAATGAATGTATAGAATGTTGATTGTGTCTC

>ONT.641.8 Contig01843 19682-38693

AGCCAGCTTCGAACTTCTAACTGCTCAATTGCAACTGCAAGACCACTTGTAAAATATGGATTCCAAGAAGGGCGCAATATCCAGATTGGTGGGGGAGGGAACAACCCCCAGGTCCAAGGGAATATTAGTGCAGGTACTGGAGGTAACATCAATTTAGGAGTTCAAAGAGCAAATGTCAATTCGTGAAATCAAGTAAGAGGTGGTGGTAACATCAATACGGGAAATCAAGGATATGTGGTGGTGGTAACATCAATACGGGAAGTCAAGGATACGGTGGAGGTAATGCCAATACGAGAACTCAAGAATAAGGTGGTGAGAGCTGGACCCACTGAAAATTAATTTCGCCAGTTATCTATGTTTCATGTGTCTTATATAATGTTTAAGCTTTGTCTACTATCAACTATGTCCGTTTACTACGTGTGATGCAGTACAACATCATTTCTGCATTTTGTGGATTCCAAGTGGCTATTGTGTAATGGTTGCTTGCTTATTAATATAAGTTTGTGTTATATAT

>ONT.6085.1 LG03 48804030-48811439

GGATTCGCGCTTCTTCTCCTTTCCGCCAAAAGAAAAGACCACCGCCACCCCTTCCCCAAAACCCTCTCACCCCACAAACTCCCACCTTTTCTGTATCACACCACCACTATTGATGAAATGAAATCCACCACGCAAATGCATAAGACATAGAGAGGAAAATTTGAAAAAAAAGAAGAAGGAGATCCTCTTCTTCTTTACAGTGGATGGATAGTAGAAATGAATTCTCTCTTGTATTTTGTTTTGTTTTCTCTCTTATCATCAAATCAAATGAGAAAATAAGCTTTTGGGGGGTTTTAGGTTTCATTCACATCTTTCCAAGTTATCTAAGAGGGCTGCTGCTGCTTCTTCTTCTTCTATCGATGAAGAGTTCTTCTCTACATGTGCAGCTCTTAGCCTGGATGGGAAGCTCAGAGAAAGATGGCTCCTGCCTCAAAGATTCGGAGATGAAAGAGATGCTTACTGCAGGATTATGCATTCTTCGAGCATTATATACCAAAGAATGTTCAAGTCCTTTTAATATCGGCAATGCATAAACAAAGTAAGGCATCTTTTGGAGTAGGGAATGAAGCAAGTGGTCCGCATAGCATTAAAAATGGTCATATATTGACAATCCAACTCATGGAGTTAGCAACACATTTCTTTTTGCCTGGAGTTTTCTGATTCCTATATTTTGATCTTCTATCTGATTCATTTGTATTCCTTTTGTATTTCTATCTTGTATCCTTTAGTGTTTGTATACTTGGAATGCCCTGTACGCTAATTCACCAGAACTAAATTCTTTCCAAGTTCTTTATTTCTGTATTGTGGAACAATGCACAATTGTCATATTGCTTTAATTTT

>ONT.11245.1 LG07 11519806-11536567

CATTTCCCCCCAAATCCGTTTCCTCCTTCCTCTCTCCCTCACCGACTTCAGTTTAGCGCATCTCTCTCCCGGTCGTCGCGCACCTCTGTCCCGGCCAGGCCCGCCCAGATTTCCCTTTTCTCCCAAATCCCCTCCTCCCTCTCTCCCGTTTTCCTCCATCTCTCTCGCTCACCGAACCGGCCACGCATCTCTCTTCCGGCCAGCGCCAGCGCCGGTGTACCAGATGGACGGCGAAGATCCATTTGACAGTATACAGCTAGATTTGCAACTCTGGCTTTCGGACGGTCTCGCAACTTCCGGTCCCCTCTGAGATTCAGAACCCGTCACAATCGACGTTTATCCCCAGCTCCTCCTTTCAAGTTCCTATTTTGGGCAGTCAAAGGTGTCTGGAGCATGGCATTTTGGGAAAAAGATGCTTCTCATAGAATCTTTGCTACTGATTGATGTGATAAAAGATGGGCAGTAGGGACTACTATATTCTGTAATGAGTCCTGTTTAGTCACCATCAACTTTTTTCATGATAGTGATATCCTGATTTGTGACCAACAACTTTATTCTTAATGGTAAATAAATCAAGTTGTTGCTGTTG

>ONT.13757.1 LG09 2845186-2847657

CAAACATCGGGCTTGAATCGATGAGGAGGAGAGTCCGAAACCTGCAACGCCCGGGATTCGGCTCCTCTGCGCATTTCTTCTTCGACGATTAAGTGATCGCAACATCTCATCTTTCTCTTCTTCTTCTTCTTGTCCCCTCTCTCCTCCGATACACAGAAAAATCTTTATCTTCAACGAAAAGGGAGGAAACATCTCTTATTTGATGAAAAAGGGGAATCTTTCGCCGTCTTGCTTGAATCGCTCCCCTCCCCCCCACCCCGCCTCAAAAATCCGATTTGACCTAGATCTGAAGCCGAGGCTCGTCTACCCGTGCCGGTCCCTCGGAGACCGCCCAGGCAGCGAAGGAGGCCCATGCGCTACCTCCAGGAGGTCTAGCGGAGTGGACGTGATTAAGAGATATGAATATAGAATCTTTTGGAAATTCTTTTGGAACTGTCTTGTATATCTATTTTGGTATTGTAAGTAATAGAAGGATATTCCCTGCATTGTATTAAATATGCAGTGGTTGACTTTAGGTGTACGAAACGATGTAAATGGACTTTATGATTCATTTTGTTAAATAGGTTGTCAATGTA

>ONT.14981.1 LG10 60806882-60827445

TCTTCACACCCTTTGTTTCCCCTCCCGCGCCATATCCCTTTCCTACCACGCCAAATCCCTTCCCTGCCCTAGCGTCGGAGCTGCCATCGCCGGAGCCACCTTCGCCGAAGCCGCCAAGTTTTCTCTCTCCCTCTCACTCACTCTCTCTCTATTTGTCTTACATCACTTGGGTCTCTCTCTCTCTCTGTATGTGTGTGTGTAAGATCCTAAACCAAGGACGAGCTCATAGCGCAGAAGACTTTGCAGAAAATCCTTAATCGAAGGTTATGGAGTTGGGCAGCATAATTACATACACCACCAAAGAAATAGATAAGATCCGACTCGAGTTTATCGACTTCATCCAAGGATTTCTTCCTTTATAAAGGTGAATAAAGAACTTAATTAAACGGAGATGGAATGCCTTTTGCTAAGTGGATCTACAAATTAGGTTAAGTTAATTTTGGTTAGTCAGGGATTTTTGGAAATTGTTTACAATGTAGCTTGCCTTTTTTTGGATGCTTTTGTGAAGGTTTTGGATTATGTCGTGACGCTTTGGTTATGAATTCGAAGGTTAATGTAAATAGTTTGGATT

>ONT.6073.4 LG03 48137246-48138285

CCTGCCCTGAGTTATTGCTTTCTGGTGTCTGTTTGCGGCCACATACCTCGCCCTCTTCCTCTTCAACGTCTCCGTGCTAGGGTTCCAATTCGCCAACGGATCCATCGACGCCGCGGAAGCGGCCTCGGTCTCCGTCTTCTCCGTCGCCGGCCTCGCAGCGGCCGTCGGACTCTTCCTCTGGAACACGGGACTCCGTCCGACTCGGCACGCGTGGATGCTCTACGCCTTGTTCAGCCTCTCCGCCTTTGGCCGATCCGCGCTTCTATTCGCCCGCCACGAGTGGTTTTTCTCCTTCTGTTTCGGGCTGGGATCTGTCTACTGCTTTCTGCTCACGTTCTGCGCTTGGAATGTTCTCCAATCAATGGATCTGACCGACCCGGTAAAGAAATCGGCCATGTTCGTCTGATCATGATGATGATGATGATATGATTATGATCATATGCTGGTATTTCTTGCTTTCTCCTTCTCTTATATGTCTGCTTTATTAGGATTGATGTGTTTAACTAGCCTTTATAAGGCTGGAGACCGATGTGTGTTAAGTATACAAGTGTTTTTTTTTTTTTTTTCCTACGGTTTTAGGGCTTTTTATAAGAAATAATGAAATTGGGTTTTGCTCCTTCGTGGAAGGTGATGGTGGGGCCAATTAGTGGAAAGTCTAGCTTGCTATCCCCACAGCTCACTTCGTGAAAGACATATTATCATTTGTCGAATTCCCTTGCAGTTGGATCACATATATTTGGTGTAAGCTTATCTTTGCTAACCCGTCGGCCACTTTCTTCTTAGATCGGCTATAGAATTTTATATCCCACCAATCCAGAGTTGAAAGAGAGTGAAGAAGTTAGTTAAATGCATTGTGTGCAGATGTATAATTCCATACACTTGTTAGTGATCCTTAGCTCTTAATTACATGCCAACAGCTTTCTCCAG

>ONT.260.7 Contig00734 42183-43746

ATCTTCACTTCCTTTGCTCCTCTCTCTCTCTCTTCAAATGCTCTGTTTCTCTTCGTTTTCAATTGGATTTCTTCGAGTTAATGACGACTGAGCTCTGATTCATCTCTTCCGATCGTTTAATTGCACTGCTTTCTGTATCCAATTCGACATGGAACGCAAGATTCTGAAATAAAAAAAAGTAGTAATTCGAGATTTGGATGCGTGGTAAATGACTTTTCATTTCCTGTTTGGATGAAACTATGAAACATCTAGTTGCTGAAATTGATTGGAAATGGCGGTAAATTTGGTTCCAAGGCGGGAGAGATTCACTCCGATTTGGTTGCTTTTGATGAGTTTCGGTGTTTGGACTTTGGAGTGTCTACTCGATGGAAGGCAGGCTTTTAGTTAATAGGACTCTGGTCCGTGGGGCTAAAGCTACAGGAGCAGGCAAGTTGTTCTATCAATGAAAAAACCCAGATGAAGAAATCATCAGAAAAACAAACCCAGATTAGCACTGGGTTTGATGGGACAGAAGCAAATGAAGGAGATGCAAACGTTCTTGTACTTCCTGCCTTTCTTCTGAGCAATTACCACTGTTGAGAAGAGATTCTTGTCAATGGTTCCTGGGTTTTGATGGGTTTTTGTTATGGAGATGCTTGAGCTCTGCGGCACTGAGCACTCCGTGAAAGGGCCTTTGCAGACATCTCTCTCTCTCTCTCTCGCTCTCTCATCTGGTCTGGTTTGGCCACAAGAAGTTAGATTTGAATGGGGTTTTTGTTTGGTGGAAAGATGTCCCTTTTGTTATAAATGGGTGGCCCGCTCGGATGAAAGATCTACACCGTTGCCTAAAGTTTCTATTTCGATATTGGATTGGAAGATCCTAACCATCTAATTTCAGGATCAGTTGTAACCTTTCATTTTGATTGCTGATAAGGGTTAATCTTGTGGGACAGGCAAGGTCCAGCCAGGAATTCACCTAGCTCAAGTCCTACCCAAATTTCTTTTGTAGGTTAGCCCGAGCTTGGGCCCAACGCCAGATTGTCTTGGAATTTCGAGTCCAAGCCTAAACCCCAGCAATTTAAATCTAAACTAGTAAGATAACCCCTGCTAAGCACACATGAACCACATGGCTAGGTCTCGACTCTTGTTTAACTACTTTTGATATTGTTTTAACAAATAATAACATA

>ONT.15896.1 LG10 51715480-51728780

GGCTGGGAAGGCCATCTGTAGAGCTTATCTCCTTACAAGCTGGCATCTGTAGAGAAACTTGGGCGCCTCTTCCTCTCTCTCTCTCTCCCTCTCCCTCTCCCTCTCCCTCAGTTATTCCAAAGAAATGCCTACGTCTGCCCTCGCCGGAGCTGAAGATGGATTGTCCATTTTCTCTCTCTCCAATCTATTTTATGCTGTTGTTTGTTGAAGCTTCCCCTTTTGTTTTATTTTGAGATTTCAATATGTTTGTAGTTCAGGTTCTTTGGGTTTGATTGTGATGTGGCTTGGGTCTCCTCTCCATTCCACTTCTTTGCACCCCTAAACAGGAGATTCATAAACACCACCGCATGAAACCCTATTTCCTCTGTTTTGTGCTTCAATGCCATTTTAATCTCCTTCTGATTCAATCTCCAACAAAAATGCTCTATGGAAAACATATCAGGAGCTTAAATTTAGACCTATTTCACTGGTATAAATCCACGCAAGTTGACATCTTTATGCGATTTGTTTTGAGAAAACTCGGTTTTATATGTATTTTACTTTGATCTTCCTATGTATTTTACTTGATCTTCCTACTTTAAATCAGAAAAATGCATCATCTGGGTTTTCATGAAATTGCTTTTGGATTTGTGATGTTTGTGTTGTCCTTCATTCTTATGGTTTGTTGGCATGATGTAAGACTTTGGTTTTAATGGGTTTCTTTTTGTTGAAAGTTTGTTGATTAATTTTCCATTCACCGGGATACTCATTCTGCTATGTGTGGTTATGAAGGATGTCATAGTCTAGAACTTCAAGACTTAAGGGGAGTTACCACTAATGTGTATTCAGTTCTTGGATTATTGTTTTTGAGTTGCAGAAATAAAACTTGTTTTGGCCAGGGACATAAACTCCAATCGTATATGTCGTGTGGAAAGATCAGAAGATTAAGAGAACATCAAGAGAGCTGATCAGGAATCAGAAATGCCAACTCCATCTATTGCACCAACTATCATTAGGAGACTAAACAATCTCTCATTTTTCTTGTAAAAAGATGTTGACTCTTCATCTTCCCTTAGTTTGATGATGGTCATGTTTTGGAGGTTGGCTGTAAGTGGAACATCTGTTGAAAGAGTTCTGATATTAACAGTAAAGAAAAGTTTTTGTAATTAGGAATATTTTATCCTAAATGTGTACATCTGCATTCAGTTTTTT

>ONT.266.2 Contig00740 1510-3242

ACTGTTTTCCTCTCTAATTTTTTTTCCTCTCCCGCACTTTGCCCTCCCGCCGCCTGCCGGAGCTCGCCTGCGCTGGGGACTGCCTGCAGCTCTCTCTCTCTCTCTCTCTCTCTCTCTCTCTCTCTCTGTGCACTGTGCCCTCCCACACCCCTGCCGGAGAGGGAGGCTCCGGAGAGGGCACGCCCCTGCCGGAGCCTCCCTCTCCCACTCCCACTCTCTCTCTTTATTTCTCTCTGCGTTGTGCTCTCCTGCGCGATCCACACTATCCCGCCGCCGTAATCGCGAGCTCGATTTTGCTTTTCCTTGCTGCTATTTCGGTCTGAAGGCGAGCTGTGTGAAAAGTTTGCGTGGATTGGTTGAATGAGGATCATCATCTCACTTTATTCCTTTTTGTACTCTATAAATTGAAATTGGTGTGAGAGCATCGGATCCATCACGGCACTCTGCACAGACAATATGTTTTTTTTTTTTTGCTAGAATTGTAATGGATATAATTTTGTTGAAACTTTTGTGATGTTTTGAATATCATTAATATAAGACTTTGTTTTG

>ONT.17490.1 LG12 907529-918299

ACGCGGCCCCTAACACACCCACCCCCACTTTTACTTCCCACGTTTTTTTCCTCTTTCCATCCCACGTTTTTTTTTTCCTCTTTTCCCCTCCAGGGCTGCGCTCGATCTGCTCTCCCGCTGCACCAGCGACGATTCCGTCGGAGCCCACCTGCGCCGGAGATCTCTCCCTCCCTCCCCCTCCCTCCCTCCCTCCCTCCCTCCCTCTCTCTCTGCACTGTGTCGCCCTCCCGCGCGCCCGCCGGAGCCGCCACCGGCTCTCTCCCTCCCTCCCTCTCCCTCTCCCTCTCAATCTGCACTGTGCCGCCCTCCCGCGCGCCGGAGCCGCCACCGGTTCTCTCCGCCATTAAAACCTCGAAGACGAACAACAACTTCTGAAACAAGCTTCTTTCCCTACGTTCTGCATCAAAATCATTTCCAAATGCAGTTGGATGTTGATTCACTGGGCCCTTTCTCTATCCCAAGCACAGCTATTCAGGCATTGCTGATTTCTCACCAGATTGGGCCTTTTCTGGGTTTGAAACAAAGCATTGATTTACGTACATTTTTGGGAAGTATGGAGAATCATAATACCATAAGGTGGTGTGGAGTTGTATGTTTGGTGAATTCGAGTCTTCTGTTGAAGTTCTGGGAACTAATGCCCTTAGATGTGTTAAAACTTAAAAGCGAATCTGAATAGGAATTGTGCCTTCAAATACGGCTTATTGCTCAAGTGGAGAAATGTGAGACAAAAATCCTAGAAGGCATTGACGCAAAAGCTGCTGAATGACAGTGCCAAATATTTTGGCTGCTCATGAAGTGGCAAAGTACCAAATATTTTGGATGACAAATGCCAGCAGCCACTCTTGATATGAATGGGCAATGAGTCCCATAGTTGCTACTGGCATAAGTTCCAGTTTCAGACATTCATGTTGATTGCTTCATTGGGCTGCATTTTTTGGTAGGTAAGAATGCCACTTTCTTTCATGCACACGATGTAAACTAGCATGTACAGTTTAGAATGCATCTTTTGTATTAAGTATATTTGCGGTTGAATTTCTTTTATATTCAAAGACCAGTTTTTT

>ONT.16349.2 LG11 47923123-47925063

GTAGCTATTGCCTTCTCCGTTTCCTTCCCAATTCCTAACAAAGAGGAGGAAGCAGAAGAGAAGGAGAGAATTTGCTGCTGCATTTCCTTTTCATCAGACAAGTTGTGATGGCTGTTGCTACCTCAATTCCAGCCACAGGATTCAAAGGCATGTGCTCGTCAGTTTATGGGGGGTGGGCCACTTCACTATGTGGTGAAGACTGAATTGTTCTAATGGCAAAGCAATCTCCTCCGTTGGTTCGAGTGGGAAGGCCCACTCGGTGCAGACCCATGATGTGCATGAAGGGAAGGGGGTGTTTGCACCGTTAGTGATTCTGACCCGCAATATCATTGGGAAGAAACGCTTCAATCAGCTCAGAGGAAAAGCCATTGCCTTGCATTCCCAGGTCTCTCTAACTACCTCTTTACTAGGGTTGCAAACATGGCGCAGATGGGAAACAACGGCAGGGATTGATTCTAATGGAGAAAAAGAATGGTGAATGGCTTGGATTTCTCGCTTGATTATAGGTGTACTCCATTTTTTTGTTTTGTTTGTAAGGCCTGCTGGTGTAGAGGTGAATAGGTGAAAATCCATGGAAAACTTGTGTTTGGTATTTTCCTTCTAGTGAGTTCTGTACATACACCATTTTATGTCTATGTGCCCCTTCATTCAACATAATTAGTACCAAACTTCTATGTTCAAGGCTTTAAGAGATGCTAATGATTGTAGTCATTAATACCAAACTTCCCTGGATCTGGTGTAATTTTTCTACTATGTGTGCATTACCATAATGATGGTGCTTTTCTAAAATCAAACTTCATTGTTCAGCATCTTTGTC

>ONT.16720.5 LG11 46784860-46792031

CTTCTCTACTTGAGTTTTTGAATTCGATCGCATAGAGACTCTGCGTCTCATCTTCTCTAAGGGTTTATTTACTGGTGCTAAACTAAAAGAAGCAGATGGCAGCTCAAAGGATCTCTCATGCCACCTTGAAAGGACCCAGTGTGGTCAAGGAGATCATTATTGGTGCCACGCTCGGCTTGATTGCTGGGGGGTTTTGGAAGATGCATCACTGGAATGAGCAGAGGAGAACCAGGGCCTTCTATGATATGCTAGAGAAAGGCGAGATCAGTGTTGTAGTAGAAGAATAGGTTCTCGTTTCATTCTATTTTCCTCTTCTGATGGTCGACCTTGTTTCTCTGTTACCTTGAATTGTCTTTTGAGAACTTAAGCTGCAAATGTGCAGTATCGGGTACTTCATGAAAGAAATAAGCCATTTGTGTATTGAAACTTGAGAACCGCATTGCTGTGTTTAACAGTCTTAATTATTTCTTCT

>ONT.1507.1 LG01 109766843-109798860

GCTGCAATCTGTCTCTCTCTCTATGTGGGGTAACCCGTATCGTCTCAAGTGACTGGAATCGAAAAGAGGAATTCATTGCAGAGGGGGTTTTCTCGAAAGCGAGAAGTGAAGGTTCTGTTGGTTCCTTGGGAAGTTGCTAGTATTGTTTTGAGCTCTGATGCCCTGATACGACATAGATACAACATGGACATGCCAAGGATATGATAGACCATCCCGTACAGGTGGATTACTTGATAGAAGGGACAGGGGTGGCTGCTGCCATATTGCTTTTTGGGTTTTGAGTCAGGACGTGGATAAATTTCAACAGAAAGCTCAAAATGAGAAGTTCTGCACTTTCTTCCCTGGTGGCAAAGCTGAAAGAAATGTTCAACAGAGAAAACGGATAATTGTAAATGTTGATGTGATTTATACTATTTTCAACAGATGCATGTATTTCATCTCCCCAGTGGTTGGATGAGAGTTCCTAATTCTGAGTTTTCCCTAATATCTGTTTATCAAAGTACTTGTTTTACATGTTCTTTTGTTGAAAGACATGACACAACTATCAGGGACCCTTTTGTAGGCCATTTGCAAGGATAACCAACTTTCATAAATGTTCAA

>ONT.641.4 Contig01843 19681-38693

AGCCAGCTTCGAACTTCTAACTGCTCAATTGCAACTGCAAGACCACTTGTAAAATATGGATTCCAAGAAGGCGCAATATCCAGATTGGTGGGGGAGGGAACAACCCCCAGGTCCAAGGGAATATTAGTGCAGGTACTGGAGGTAACATCAATTTAGGAGTTCAAAGAGCAAATGTCAATTCGTGAAATCAAGTAAGAGGTGGTGGTAACATCAATACGGGAAATCAAGGATATGTGGTGGTGGTAACATCAATACGGGAAGTCAAGGATACGGTGGAGGTAATGCCAATACGAGAACTCAAGAATAAGGTGGTGAGAGCTGGACCCACTGAAAATTAATTTCGCCAGTTATCTATGTTTCATGTGTCTTATATAATGTTTAAGCTTTGTCTACTATCAACTATGTCCGTTTACTACGTGTGATGCAGTACAACATCATTTCTGCATTTTGTGGATTCCAAGTGGCTATTGTGTAATGGTTGCTTGCTTATTAATATAAGTTTGTGTTATATATA

>ONT.16581.1 LG11 28743467-28745956

TCTTCTCCAAGGTATGACTCCTCTTTTCCTCTCTCACACCCTAATAACAACAACATTAAAAAAAAAAAACCCTTACATCGCAAGAGGCAGGAGCCCTGCCGCAGGAGCACCCCCCGCCCAATTCTCAGTATTTTGTCGCTTGGTGGGCCTTGATAACTACCATGTTCCACTTGGATGGATATGAATCGATCATCCCCAACAAGAAGAAATCATGCATCCGGGCCGACTTTTAAGAGACCTTTCTTCACATTTTGATGGCTATATGAGGTCCTTTTGAAGTATTTTTTAAGATGAAATTCGATATTATAGAAAAAAATAATAGCACATTTACTGCTCGGCCATCGGATGACAAACCAGAGGACTTATAGGCGGACCTAACAGAAGGACATGTGGAAAGGGTGATGTACAAAACTTCTTGATGGCGTGTAGACATGTGAGAGTTAAAGAGATTATGAAATGGTGATGTTATATTTTGGGTTTTGAGAACCTCAATCGTTCAACTGATTTTTTGTTTTTGTATATATTTGTTTTGCTAAGGTCGTTCAACTGTTTTTGTTAAGTGGATTTTGTATATCATCTAGCCACGCCCCAAGCTCGCTCACTTGAAATGGAGATATTGTCATTTTATTGAACTATATTAGTTGAATGTGATGGGAAACTTAAAGTTGAATGTGAGTTTTTACATTAAAATCAATAAGAGAATATGGAACTGTGAA

>ONT.160.1 Contig00448 38762-44644

GACAAACCCATTCTGCATCTCCCTCCCTCTTGGTTCCGTCACCCATCTTCATCCCTCATGTCTCTAACCCATCCCTCTCTCGCCCGCCAACCTCTCCACTCGCTACCCTGCGTCTCGATCCCTCCGTCTCCCCTACGTAAAGAATCCCCATCACATGCCCAGACCACCATCTCCCTCTCCCACGCAAGCCATATCCCTCACGACCTCAACCCTCTCCTCCATCTCCCAGGCGACCTGGTCCCCCTCTCCCTCTCATCAACTCCCTCTCACCACTCCCTCTCTCGATCACCCTATATCTCGCGGCTGTGCTTGAGTTTTTCCCTGCTGAGAAAGATGTGTACATTGAATGCAAGCACACCTGCACTTGCCATTCCCAAAGTGAGCTCTCAAGGACGATTGAGCTTTCTGGTGCTGCAATTCACCAACAAGGGTTTAGTTCCAATTAGAATTGGCTAAATGTTCGATTCAAAGAGTGAGATGGAGGATGGCTATGGTGGCTGTTTCATGTTGCTCACCTTGATGTCATAGCGTCACTTCGCCCATCAATCTAGATCAGCATGGTGTGTCTTACTTACTGCTGGAATTTTCAAACATTTACAAAGTTCTGGGATCGTTCTGATGTTGAAACATTTAGAAAATTTGGATGGCTTAGCAACTTATCATAATTTTTGTTCTTGTTCTTGTCCTTGTTCTTAATCAGGAAGACTCATGGGTCTTGTTGTACACAAGTAATGTTAGAAACAGAGATCTAAGGCCAATAATTTTTTTGTTTTGGGGGCGGCACACACACACACATATATATATATATATATATATATATATATATCATGTGATAGATCTTCAATGATATCAATCATAAATGCTGGCCCTATTACTTTCAAATCCAAGATCCAAGCTTATTGCTCTGGACCTTGTGTCCTCCTCTTATATTCCTCCAAAGACTTTTATTTTCATTCTGTGATATATTTTTTCTATGGACTTGATTTAATGGAATCTCTCTTTTCTCGGGC

>ONT.11163.1 LG07 5836768-5839582

ATTTATTGCTTATTACATCTTGAGAAAGGGAGAGAGTGAGAGAGATTTCTTCCTCCTTTTTCCTTTCCATTCCTCTTTCTTTTCCCAAATCATTTTGTGCATATGGTTGAGAGACAAGGATTTAGAGTCTTCTTCTTCTCCATCTTTATCTTTATCTTCATCTTCTCCATCTTCCTGTTAAGCCTTGTTGACAAGAACCATATCCTACAACTTTCATTTTCGGATTTCTTATTGAGGGTTTGTTATTTGGGGGTTTTGGGTTTCCTTGAGCCATAGCGAAGAGAGACCTTGGAAAAGCACCAATTGAGGGAAATAAATGTCCATTGACAGGTGTCAGTGAGTAATCCTCACATGTAAAATGTGGAGTTTTCTCATTCGTGAATTCGGATTTGGTTTGGAACAGGTGCCAGTTCACGGGAGTACGCGTGTCACACCTTGGGAACCACCCTAAACAAGGAATTCTCGGGTCTAGGATGTGAGAAGATATGAACATTCCTTGTTGGTCCACATCTACTTGAGTGTGGCTTTCTTGGGATCTAGCCACTCAAAATGAAGGCACTACCCTGTAGCGACTCTTCATTATAGATTTATAGGACATGTGGGCATTCCAATCAAATCTTGAAAGAGGTGTGGGCCCCATCAATAGATGGGAATACTAAATTAAGGGCATCCACCTTGACCACCATATGTGGGAGGGGAAAATCTCCATTGGTGAATTATATGGGATGTCTCAAGTTCTACCATTTTAAAGCTGATCCCACTGTAAGATTGAATGTATGATTAATACAATTTATCAATATGAATACTAAGGGTGTCATTTCATTTCA

>ONT.260.9 Contig00734 42639-43746

ATCTTCACTTCCTTTGCTCCTCTCTCTCTCTCTTCAAATGCTCTGTTTCTCTTCGTTTTCAATTGGATTTCTTCGAGTTAATGACGACTGAGCTCTGATTCATCTCTTCCGATCGTTTAATTGCACTGCTTTCTGTATCCAATTCGACATGGAACGCAAGATTCTGAAATAAAAAAAAGTAGTAATTCGAGATTTGGATGCGTGGTAAATGACTTTTCATTTCCTGTTTGGATGAAACTATGAAACATCTAGTTGCTGAAATTGATTGGAAATGGCGGTAAATTTGGTTCCAAGGCGGGAGAGATTCACTCCGATTTGGTTGCTTTTGATGAGTTTCGGTGTTTGGACTTTGGAGTGTCTACTCGATGGAAGGCAGGCTTTTAGTTAATAGGACTCTGGTCCGTGGGGCTAAAGCTACAGGAGCAGGCAAGTTGTTCTATCAATGAAAAAACCCAGATGAAGAAATCATCAGAAAAACAAACCCAGATTAGCACTGGGTTTGATGGGACAGAAGCAAATGAAGGAGATGCAAACGTTCTTGTACTTCCTGCCTTTCTTCTGAGCAATTACCACTGTTGAGAAGAGATTCTTGTCAATGGTTCCTGGGTTTTGATGGGTTTTTGTTATGGAGATGCTTGAGCTCTGCGGCACTGAGCACTCCGTGAAAGGGCCTTTGCAGACATCTCTCTCTCTCTCTCTCGCTCTCTCATCTGGTCTGGTTTGGCCACAAGAAGTTAGATTTGAATGGGGTTTTTG

>ONT.5010.7 LG02 116691291-116695143

ACACATCTTTCTAGGGTTCCTCGTTGCCTTTTCTTTCTTGTTTCTGTTTTTCTTAGATCTGCTCGTTTTCTTTCGTCTTTCGGCTGGTATGCAGTCTATTTCGTATTGATCTATCGCCGATCTACCTATTGATAGTCGATTTTCCTTCTGATTTGCACTTAGCCCTTTGTTTATTGGTTGTTTTGCAGCAGATCTAAGGTATTTCAACGTTTCGATCCTCTTCTTCTTTGTTTCTTCTTGGTTTTCGCGGTTTCTTTGCCTAATCTTCTTTGATTTTTCTTGAAATCGTATGGTAAACGAGGTCTAGATTTGTCATACCTTTCTTTTTACTGTTTGTGTTTGTCGATCTTCCTTTCGTTTTCAGATTTGAGGTAGGCTTGTTCTTTTTGTTGTAACTTAGGGTCTGGTGCTTGCGATTGCAATATTTCTTCTTTTTTATGGTTGTTTTGAAGCAGTCTGACGGATCAAGACTCATAAGGCGATGGAGGGGATTCCAGTTTCGCAGATCGTAGTCTCTTATAGCAGTAAAGAGACTCCTTTCGCAAGATCCGTTGCATTTTGGCGTTCCTCGTCGACGATTTCATCGGATCCATGGGTTTTTTTAGCGATCTTTCATCAGATCTACTATGTATGGATGTAAAGAAAAGTAGTTTAGTGTGAAGATCGAGGAAAAGCTTGTTATGTATGAAGCTGATAGAGAAATAGGTATGGATTAAGCGAGAGAGAGGCTTGTGGCAATGGATCTATGTCATCCTCCGACCTTTGCCATGTTGGGTGCGCTCGCATGGCAGGTCAAAAAAGCCTAATAAAATTCTCGATTTTTGGTTCTCGGGGGAGTTCGCATGGGCACTTTCGCCTGGGTTCTCCCCCTCCTGTGTGTCCTCTCTTTCTTTTCGTTTCTCCATCTCTTTCTTTCTGTGCGTTTGATTAGCAATATCTGCAACTCGACATCACGCTTGCGAAACCGAGCAGAAGAAAGCTACGATGTGGAAATCTCTCGTTATATCATGGCAACATCACAAAGTCCTAGTGTAGCATAAATTCTGTAGGGCAGTTGGGTAATTGTCTGCTTCCAAGTTTAGTCTTGTTTGGGGAGAGAGCATATGCAACGGTGGAGCAGCTGATCAGCAAGAGGAAAGGTGAAGGTGTATTCTTTGTTGCTGCTGGACGTGACTGATAGGAGGCTCCTTGGGGATTGGATGATTTCTTTGCTGTTGGTGGACCTGAAGAACAGTAGTGACGGAATAACTTTGGTTCGATGAATTCTTTGCTGCTGGTTGACCCGTTGATGGTTCACATTGCAATCCTCGGTTGGATGAATTCTTTGGATGTGGACGACATAGGGCAACGGGGATGAACTGTGTTTTATCAATTCATTTCTGCTTTTGCATCTTTGCCTTCCAGACAGACCCTCTCACCCGTAGGAGTTTAAAAAATCAATGCACGAGCAGGCTTATTATGCTGGTTCCTTGTTCGTAGGTTATGGCCAAACCCATGGCGTTTGTTTTCCTTCAAATAATATGCCTCTCGACAATGCTTTAACTAACAGGTCGGCGGCTTTTTACCACATGGTGGTGCAGTGGAGTTTGCGCGCTATCTATTCAAGCTTTGGAGTTCGTCTTTTGTTCTGGACTGTTTTTATCATGCGTTTCCAGAGTTGTTGCTGAATATTATTCCAGACTTCTTTTCTCTGATTTCACGGAATTATGGTGGGCTGATTGGTAATCATACTTCTCATAGAACTGCAATCTGCTTTTAACTACAAATTTTATTTTTACATGTAGCGTTCATTCGTTTATATTCATATTATGCACTGGTATCTTTTTTCTTTA

>ONT.15547.2 LG10 17634782-17640483

GAGCGAGAAAGAAAAAGAGAGCTGATAGACAAAAAGGAATTCCTCTCAGCAGACGCATTTCCAAGAAATCCATCCGATTTCATCGAAAATGGCGGAGGAGAAAGGCAGCAGCTAGGGTTTCGAGTACCAAAAGCTCCAATCTTTTCCACCTGCATTGCCTCAGCTACCTTCAAAAACAACCATCTCTCCTTCGAATCTACCCAAACCCAAACAAATCCCCTGAATTTGAAGAAAACCAAGTGGAAAAAACGCCTCTTCTCTGCTCTTTTCGGAGGAAAATCGGAGAAAGAGAATGGCTTGATGATGAGAACGAGTGTTGGAGCTTCAATTCGTTCGAAAAACATCAAATGAAGAAGAGATTTCAGAGAGGTCAGAGACTGAATCTCGACCGTTGGATCCTGATTCCTTGTCGTGGGATTTGAGATTCAGCATCTTTCTTCTTCTTTTTCATCTTCAGGGGGGAATATGGCTTCTAGTACATGGACCTCTATGGAACTCTCTCGCATGATGGGATTTTCCTTTCGATCCTTTGACAAGTGGGGTGGTGGTGGAATGCCAAGGCCAATAATATACCATGACTTGGTGCATGTCATCTTCATGTACTTGTTTTATTTCTTGTTAGCCATTTGGTAAAGGTGGTTTCAGCCAATGAATGTGGTGATATTGGCGGGACGGCTCAATCTCTTTTAAGCATATGCTTAGCTACTAAATGCATACTTGCAGTTAGTTTTTCCTAATACTTTAAAATTTTTTTA

>ONT.424.3 Contig01206 74268-103069

TTCTTCTCCTCTCCTTCTATGGCTTGTTGAAGAACAACTCCATGGAGGCTTCCATTGCAAAATCCGATATGGCCAAGCAACTCCCCATCATCTTCCCCGTGACATAGTTTGTTTTGGTGCGAATCCACAGTGGCAGTCCCCTTCTTCCATCACCTTCTCCTTTCTTTGACCTTCAAAGAGGAGATTTTGGTGGGTTTGTTTGGGAGAGAAAGCTCTTGGTTTGGAGCTTGGATTGAACGTGGTTTAAGGTTGAATTAGCTCTTGATTAGAGGCAAGGGTTCAGGGAAACCAAGAGGCTTAGTGACGACGAGATCATTATCAACCTGGGATAGGATGCTAAGGCACAGGTGGTGTCTGTTGGAGTAGTTACTTTATGCTTTTCAAATAATAAGTTGATTTTGTCAGACATTTTATATGTACTGTCTTTAAGGCAGAACTCAATTTCAATTTCAAGTTGAGTGAATAAAATTTATTCTGTTGACATTGGT

>ONT.3506.1 LG02 57439180-57465994

GGCCAGTTGATTCCCTGTCATCTACTACGCGACATAATATTTTTCAGCACAAATCCAGTATGGCAGCCCCTTCTTCGACCACCTTCTCCTTTCTTTGATCCTCTAAGAGGGAGTTTTGGAGGTTTTGTTGGGAAGAGAAAGCTCTTGGTTGGAGCTTGGATTGCTTGTGGTTTGAGAGTGGATTTGGTTCTTGGTTAGAGGCAAGTTCTGTTTTCTTCGTCCTGAGATGAGAGTTTTTGTTGGCTTTGGAGCCATGTCTTGCAGGTTGTTTTGTCGGCTATGTCATGTAGACATTGAGCTTGACATCGGAGCGACGTCACATTTGGGTAATAGTTAATAGCTTATTGTTGTATGGCCTTTATTAGCCTTTTCTTGGGATTCTTAATTAATGTACATTGATAATTGTAACTTGAATATATTTGGGAATGCAATATCCTGTTACTATCA

>ONT.6085.2 LG03 48804031-48811439

GGATTCGCGCTTCTTCTCCTTTCCGCCAAAAGAAAAGACCACCGCCACCCCTTCCCCAAAACCCTCTCACCCCACAAACTCCCACCTTTTCTGTATCACACCACCACTATTGATGAAATGAAATCCACCACGCAAATGCATAAGACATAGAGAGGAAAATTTGAAAAAAAAGAAGAAGGAGATCCTCTTCTTCTTTACAGTGGATGGATAGTAGAAATGAATTCTCTCTTGTATTTTGTTTTGTTTTCTCTCTTATCATCAAATCAAATGAGAAAATAAGCTTTTGGGGGGTTTTAGGTTTCATTCACATCTTTCCAAGTTATCTAAGAGGGCTGCTGCTGCTTCTTCTTCTTCTATCGATGAAGAGTTCTTCTCTACATGTGCAGCTCTTAGCCTGGATGGGAAGCTCAGAGAAAGATGGCTCCTGCCTCAAAGGTCTATTCGGAGATGAAAGAGATGCTTACTGCAGGATTATGCATTCTTCGAGCATTATATACCAAAGAATGTTCAAGTCCTTTTAATATCGGCAATGCATAAACAAAGTAAGGCATCTTTTGGAGTAGGGAATGAAGCAAGTGGTCCGCATAGCATTAAAAATGGTCATATATTGACAATCCAACTCATGGAGTTAGCAACACATTTCTTTTTGCCTGGAGTTTTCTGATTCCTATATTTTGATCTTCTATCTGATTCATTTGTATTCCTTTTGTATTTCTATCTTGTATCCTTTAGTGTTTGTATACTTGGAATGCCCTGTACGCTAATTCACCAGAACTAAATTCTTTCCAAGTTCTTTATTTCTGTATTGTGGAACAATGCACAATTGTCATATTGCTTTAATTT

>ONT.15012.1 LG10 68290298-68292323

GCCTCTAAAACCCTACCGCCACGCCCATCGCCCTCCCTTCCTCTCTCTCCGCCTCCTCTCCTTCGCCAAACCTTAAGAGTCCGTATGCGCATTAAGCCTCCCCTTAAATCCCTCCGCCCCTAGCAGCCCATCTCCCCTCCCCTCCCCAGAAACCCAACGCCCCCAACTTCCCCAACACCGTGCCTCCCTTGCCTGCAACCGCCTCACCCTCATTCGCGAGAAGGACCTCGATGAAGCCTACCTCCTCGTCCGCCACTCCATCTACTCCAACTGCCGCCCCATCGTATTCACCTGCAACGCCGCCCTCCTCTGCCAATCCTGCTACTCCGACCTCCTCTCCCTCCACTGCTTCATCACCCAAGCCTCCGTCTAGGTTTTGCTGGGAAATCCGTAACCGGGATGTGGGCCGACAAGAGTATGGAGACAACCAGCCCAAAGACTTTTGGCATGTGTGATTGAAGAGAGAGATTTAAGACTCATTAATTCAGGGGATTAGCTATATAGTTGATGCTTAATTTAGAAAACTTTAATTGGTGGATTGTGTGGTACTTTTTATTAGTATTGGAACTTGTTTACTTTTTTTTGTTGGTTTGTGATTTAATAAAAGATTGTGTGTTTAAGGTTTTTGTGTT

>ONT.10022.1 LG06 5832645-5845741

ATTTCAGTATGTAGACGAACAAAACCCTATCCCTAAAACCTTGTACAGCAACACGAGATGCGAAGATGCATATCTCCGTTCTTTCCCTCGATCTCCCGACTCTTCTGCAAATCGTCTTATAGCCATTCAACTCCTCCACTTGGTGAAGGTCACGGCCTCCAGATCATCATATTGTTAGTGGGCAAAATTTCATGTGGACGCGACTACATCCTCATAGCTTGAGAAGGTGGAGGACCAGTGACACGAAGGCCCTGATTTGATTTCCCAGATGGGACATTTAAGTCAAAATAGCTAATAACCAATTCTTCTGTTTCATTATTGAAGAAAAAACTTTACTAAACTGAGCAGCACATGTGTACAATCATTTGAGCTTGGTGGATATGAGCAAGCCCTATCCTAGTGAAGCAATTTGTGAG

>ONT.17053.1 LG12 14990130-14993294

ATCCATTTCCCTGCAACGGTTCCAGCAAGAGCTGGAACCACTCCTCTCTCTCAGCCACTCTCTCTCTCCTCTCTCCCCTCTCCCTTGGCTTCTCTTGATTGTAAATGGAGATTTGATCTCCTCATCATCTTCCATACCTTCCAAGCTTTCCAAAGATGTTATTTTCATGGATTTTGAGCAAAGGCAACTTCTCTTTTCAAGCTCTTCTTCTTGGTTCAAGGCTTTAAAGGTTTCTTGTTCTTCCCTGCTTTTCAGATGTGTGGATCGGGGGAGGCGGTAGAGCCACCGGTCATCGTGAGAGCCTACTTAAGGATATCATATAGATGTCAAGGTGGGCAACAGAGTTCGTAACAACTGTTGGGATTAGTATTTGTTTAGACATTTTATTTTGGGTATGGCTTTTATTAGCCTTTTCTTGGGTTTTGTTAATGAAAATACTTTGTTTTAATTGTAACTTGAATATTCATGGGAAATAAGAATATCCTGTTATTGTCA

>ONT.560.4 Contig01698_ERROPOS594908 418863-420676

GAAACAGGCAACTACTGCTTTTGGGGTCCATTCATGAGGGGTTCCTTATCAGCTGTTGGCCCCTAATTGATCATCGTCAATGGATTCAGGCTGGTGAGTTTTATCTAGGTTTTGGGTTTCTGGAATTCTTGCCCAGCTGTATAGTTGTTGTGCACAGAGTTGTCAGACTCTTATGGCCCTTAGTTGTGTTATCATAGTATGCTCAGGTGGTGGAATGTTATGTGGAATGAGGAATGGTTTGGTGCCAAGGAAGCTTGCATCTTGAAATTGCATTGGGAGACAACTATTAAAAGGAAGGCAAGTGCACTATTCATTACTGATGTTATCCTCATGCCTAATTTTCTTTATGAGTGCTTATGTTCAACCTTTTATTTAAATCATTTGTCATGATAGGTTGTGGTTTGTTTTTATATTATATATGTATGTATTTCTAAATATTATAAATTATATATTTGAATTCAAGCTAGTATATTGATTCTAATTTT

>ONT.6452.2 LG04 4579161-4581899

GGGAATACTATGTTCTATGTTTGAAGCAGCTGGCGGAATTAGGACATAGCCTTGATACCGATCCAATGCCTTTGGTCGGTTCCAACTTGTTAATGTTCATGACCAGGTGCTTTCGTCTGGTGAAGCTTTTAGTAAAAAGAAGAATAGGAGCTGGAGCAGTGTATTGTTCTCAATCAGTTTGAAGAAGGTAGTGGTGTCATAAATGGTATGGATGGGCTCCTTTCTTGATTGACAGTGTATTGTTTTCGACCAGGTCTTGTGTAAAGGGGCCCGATTTTGAAGCAGCAGCAGCAGCTGAAGAAAAAGTGCAATGTATTGTCTTCTCTTCGACCAGATTTTGTAAAAAGAGACCCTGTTCATCAGGAGTAGGCGGTAACGTGAATGGCATGAATGGGCTTCTTTCTAGCTTGAATAGCAGTATTGTCTTCAACCATATTTTGTGTAGGGAGACCCAATTCATAATAAGAAGA

>ONT.8803.2 LG05 101866899-101869370

GAAACGTACAAGGCATTCAAGAGATTGAGGTCGGAGAAGGGATGTAAGAAGCAGAAGAGGCTCTTTTCTGCAGCAGTTAGCGGCAAAACCATGGCCTTTGGAAGTTACCAATCTCAAGTCTCCAATACGATTCAAAGGGACGTTCTTTCAGAAGCCAGGAACTCCTGCTACAAGATGTATAGATTCTCTTGAAATTGCTGCACCAAGGCTTCCTGCAGTGCATGGTTGAGACATCCTTTCTTCATTGGATTGGTTTGCATTCTGCTTGGTTTCAGCTGAGAGTCATAGAACTTCACTCATCCAGTGCCGAAGGACATAATCTACCATTCTAGAGGATGAAATCTAGCTCAATGACATGGGGTACTAAGGACAAGGGTGTTGGTTAGAATTTAGAAATGCAGGGTGTTGTCTTATATATTCCTCTTATATTTCTTTCGTTTGAGTTACGAAGCAAACCTTAACAATGAGAAATCAGGAGAACTTTTATCAAGAGTGCCTTCGTGGCTTATTACCTTTTGTTTGTTGGCGCATACTTCTACACATTTTACGTCTATTACGTAATGTATACCACTACATTGATGGTTACAGCTTCTAAGAGATACAATCGATCCAGGGGGACCAGGGCAGTTGTTACCATGAGATTTATTTTATTCTTAAGCAGCGACAAGTTTCATATGCATACAGTTACAATCATCCACCTTGCCAGTGTGCCTATGATGCAGCAGCTTCCTGACTTCTAGTTAGCATGTATAATCTTGTGAGAATACAGTATGGTTGGATCCATCCAAAGATGTATGTTTAAAAGTATTAGCTCCAAGGAGGAATTTTTAGAATCTTATAAGTTTTTCAGGAATTGGACAAGCGCTGATTTTTCGAGAATGGAAATCTTATGATATGTTAGGTAGAGATCTCTTATCAAAATCATCCATGCCCGAAATGTATCTTGATGCACAAAAGTTGGATAACATGCCCCGTCGATAAGGATTTTGTTGCCCTTGATTTCTATGAAAATACAAGCTTCTCATTGAATGATTAAAAAGAATCCTCAAGGAAGGTGCCATGGCAGCAGAATTCAATGTAAGAAGATGTTCCGCTCAACTGTTGTAGTTAATTAGATTTTTTGGCCCCTTGTTTCACCACTAAAATGAAACCCTGTGGCTATTATGTGCTCCTTCTGTCTTCTACTCCAAGGATTCAGACACCACGTTTGGATTCTTTTGTTTGTTAAATGATGTATTTTTACATTTGCTAGAACTTTTTTATACCTCCCATTTCGGTAAATTCTGAAGCAAGAGCATTTGAGA

>ONT.3307.1 LG02 32299980-32307182

ATAAGAAAAGGTAGGTACCCTTTTATCATGAAGCTGTCCTCTTCCTATTTCATCTCCAGCGTCCGTCCCTTGTCCCAGGCTCTCCTGCTGCTGCTCCTCTCTCCTCTCTCCTCTCTCAGCCCTCCTTGCTCCTGCAAAAATAACCATCTCCTCTCTCTCTCTCTCGTTCTCCTATGCCAAGCCCTCAGCGCTAGGGCTCCCTATCCTCTCCACGCTCTCCTGAAGGTTTGTTTCATCCCGTGACAAAGAAGGATGATGTATGGTGACCCGCAGCAGCAGGTTGGTGATTTCCATAGGGGGCACCTATGCGTCAGCCCTCGGCTTCTTCCAATAATCTCCAGCCAGATTATCTTCATTCGAGCGCCCCTCCTCTTCCTCCTACTCCTTATGTAGGTGCTATACTATACAAGATAAACATGTGCTATGATATGTTCCACGGGTACTCCCTCAATACAATCTTGAGATATAGGTATGGTACTCCTATGGAGCAACTGCATCTTTAGCTTTTACAGTTCAGAAGAGTCAGCTCACTGTGTGATTAGGCCAGAGAAGGTTTGATCATGGAATAGTTAAAGAGTCTTGGAAGAGCCCTCCCATGCTTGCTGGACGTGGATTTCCTGAAAAGAGAACAGTTTGAAGCCAGCTTGAGAATTTTATTCAAGTTCAGCTTTATAGTTTCTGGTGCAGGTAAACTACCAGGCATGAGAAGGTTTAATCATGTAAGAGGTATTCTCGAGGAACTATTGACAAAAAAAGGGGAGTTAAATCTCAGGGAGCCCTCCCGTGCATGCTGGACGTCGGTTTGGATTAGGTGATAGATGGGTCCCACCCATAGTTATCACGGTTTGAACCATGAGATAAGTTCTCACCAGATTATTTTCCTAAATATAAAATCCAAGAAGACAACTGCTTGGAACAATTTGAGTCAGGGTTCACTGACTATACAATTAAGGCATTGGTATTGTGGCTTTTATGGTGTGTTTTTTTTACCCCATTTGAGATATATAAACCAAATTAAATA

>ONT.11810.1 LG07 9747509-9748189

AAAAAAAATCCCAGAATCTCTTATCATTCAATCTAATTGCTTCCCATCATCCACAGCTATTCTTCATCTTTACTTCCTTTCTGTGAGCAATACTTGCACCTTTTCAGAGCTAGTTCTTTCTCACCATGTCTATTGCAAGCTATTTCCAGGCTTCTGGCCGATTTATCACTGAAGAAGGCAGAGAAGAAAGAAGGTATAAGCCAGATGTCAATTTCTAATCTGACCAAGGAAGAGAAGAAAGAGAGTGAAAAGTCACCCCAGGTGCAGAATCGGAAGATGAGACAGAGCCCATTGTGTTTTGCACCTGAATTTGATGGGCTGCACTCCTTTGAGACTTTTGTTTCTCATTCATAAATCACCTTGTATTTTCAAATTTTTTTTATTGAATTCATCTATTGATTGGGCAAGAAGTATATACATTTGAAAATTTCTTTCAAAATTATTTCAATTGAATTCGGCATAGTTTATTTCTAATTTAAGTCGGTTGATGTGCAAGTGAAATCTGATTTATGATTGTTGGGATGTAATTGAAATTAACATTTCCCACATATGGTTCGTTAACATTGTCACGCTGGGTGTTCT

>ONT.8756.8 LG05 97423347-97423975

GGAGATTTTTCAAGGGTTTGCTGCAGCAGAGGAAGCTGAAATGGTCGTCTTTTAGATACAATGCATTTCATGTTGTAGGAATGGGGAGCCATCTGTCCACTGAGGCGGCTCTTTGGTTTGGTTCTTTCTTTGTAAGTGATATTCATGTCCCTTTCCATACATACAAACTTGCTGCCAGTGCATTATCTACCTTCCTGAGACACTGATAGATTTATGGACTAAACCGAACCATCATGGCCCAATCCGACAAAAAGTGAATGTTGGGTTAGGCTTGTTAGGTTCGGGTCAGGCTTGGGCTTGGATGTTGAAACCCACATGGAATTCAAGTCAGGTTCGGGTTAGGGCTTTGGGTATCCAATGTATCTATAAACATATGTATAGTGTACATAATACATGGTATATACACTGTTATATTCATTTTGTATACACTTTATAAAATTTGTATCTTTCGAAAAGCAAACCAAACC

>ONT.12315.1 LG08 5261820-5266756

GTCTTTCTCTCTCTTTCTTTATTCTTTCTTTCGTTTTTCCTTTTCCAGTTTTACCTCTTGCGTATGGGGGGATCCATCTATCAATTTCATCATCATCCATTTCTATCCCATAAACCCGCGTTCTTCCCCAACCATGAAACCCTTCTCGCTGCTCTGCCCTCTCTCGTTGATCTACTGCCGGAGCTCGAGCCATCGATCGCCTGAGCCATCTCCTCTCTCAGCCACAGATCACATCCGTGACGATCACGATTGCTATATATATTCATTAAAGAAAGATGAGTTTCAAAAGAAAAATGGGTTTCAATGAGTTCATTAAGGCATAAATTCCGGTATGAGATGGCGAGGACGAGATTCAGCTTGCCATTGCGATCCTTTGCTCAAGTTGACGCTGGAAACGATACTTAATAAGATTGTTGAGGACTTCTTTCAAGGGCGTCCAATCAGCCATCAGAAGCTTAAGGTACTTCTTGGTGATCCTTGGTTACAAATCATTGCCGGGGCACTTCTTGGCATATCGGTTACTTTTATTTGTCATTAAGGTTTCATGGGTGCTGCGTAAATTGTTGAAGCAGGTACTTGGGAAAGTTCATGTTAGTGAAGTACAGGATTTTACCCCGTACCACCATCTCCAAAAAACCCCATACCCTCTACCGAATCCGAAATCCTTCCCAAACCCCAGTCCCATAAAAACACACTCGTATATTAGGAAGTTTTGGAGATGTCCTGATTGGAAAGATGAAAAGGGTTGTGGTAGATTCATATGGAAAGATGAAGTTGACTATGATAAGAATTTAGCAGCGCTTATCGAGGATATATGAAGAAGACAATGGACAAACTAAATGAAAACATGTGTAGCAGTTGAAGAGATGAGACAAGCGGCTGAAGAAAGGAAAAAACCAAATAAATTTGTTTCTGTAGTGGTGTTTGTCGGTTGTCTGATCTGATCTGCATTATGAAAAATGTGAGGTATATTTTGAACAAGCACGGATTTTTTTTTGTGGCGATTTTAATGGCGGTGGTTTTTAATAGTA

>ONT.5955.1 LG03 37548013-37549065

ATCTGGACATGCAGCATGCCACATGGCAAGAGGCAGGGAGCTATTTATCTTGCTCTGTGCACTCTACCACTAGTTCCAATAGCAACCCAATGGCTACTTCTGCTTCCATTTCCCCCTTCCTCAAATCCAGAGTACTTGCAAGGGATCAAAAGGAATCACAGATCTGAATCCTATAATTCAGGATATATGTGGTGAATCTCTAGTTCAGAACAAAGATGAATTTCTCAAAACTTTTTCAACAGAAACTCAGTTTTTCAGAAACATCATCTCAAATGGAGAAGTCATAGGCTCTGAAGCTTCCAAGGGGCATGATGATGCATCTTATAGCTATTTGGAACCAGAGGCTTCCATCACGGACGTGCTCTCTTAATTTTACTTTTATTCTCATTTGCATTTTGTAATTTCATGATGCTATCTGGCTTCTATGTCTAGATTTTTCAGTTAAATAAGCATTTCCAAAAGTAATGTTCTAGACTTGACCAGAATGTTTGTGGTTATGATATTTCATCCTGCAGCTGATTGATATGGTGCTCTTTGTAC

>ONT.11369.1 LG07 62850370-62860417

GCCAAATCCCTCATCTTTCATTTTGCGCCCTAAATACCGATGTATGTCTCTCGCTGGCGTCTCATGTGAAGGCACTCAGACCTCTCCCTTCCGACAACTTCTCATGGAGACTTCAGATCTGAATCAGAAAAGGAGGAGTAATAGATCCAGCAGGCTAAAACTTCTTCGAAGCCATGGCAAGGTGAATGGTCACTATGAAAGCTGAACACAGAGTCATCATGGGTGGTTGCTGGTATGCAAAAATGTGTTAGCAAAGCAGGTAATGTCACCATTGCCCTTGAGGAGGAGTGCGAGGAGGTGGCACAAGTGCCATAACCCGTAGTCGAATGGAGTGCAGACGATGCCATTTAGACATTAGGGACTGCTCGCGAGTTGAGGATCATGTTTTGAGAGATGTCTTCGTATTTTGGGAATGTATTGGTCTATGTTGACCATTTTATTTTGGAATTGTATTTAGAACTTACTTTATGTAAATATTCAACTTAAATGCTATGGGATGCCATTTTTAG

>ONT.504.1 Contig01473 41574-44299

CAACCTCCGAACAAACAGAGGAGGAAATATCACTCTAAGTTGGGCATAGATCTCCTGCTCGTGCACAGTTGAGAGGGGATATGCTGCTCAAAGAAAAAGCAAAAGGGGAGAGTCTTGGGAGAAGTTTTTAGATATTCACTTGTTACCATGCCAAAGTCAGAGATACTAAGCATGAAACTCTTACCTATTACATTATGATTGCAACAGTAACTTCCATTTCACCATGAACATCACATACTAATACTAACCGTTAGGATAGGGATTCAGGTTTGTGATCTTGTATTACTTATCTTAATTTTTGCATTGACACTTGACAGATTTCTTATGATGAATCAACTTGTTTTGCTTCAAGTTCACTTTTATTTTTTAATTTTTTAGGTTAGGAGTTTAAGTGCTTCCATTTGTGAAATTACACAGATAAAGAAAAAGGAAGATTTGCTTCTCTCTATAGTGCAAATCCTTACTTTGGTAGCTTGTCACCAAGTTTGTTTCTCGATTTCATTAATGTTTTAGTGAATTGCTCTGCACTCTGTTATCTGAATTAGACCTTTTAAGCATCCATCAACATTTGTATGAAGAAAAACATGCATCAGTTCTTTTTCTTTT

>ONT.293.2 Contig00884 106852-108258

AAACTTCTCAACTCTCTTAGCTTGTTTTTTTCTGTTCTCTAATTCATTTATGGCTGCTGTTGCAGGACTTCCAATTCAGTCTTTTTGGCTCTAAGAAGACCCCACCACCTCAGGCTAATAACAAAACTAAAAATGTCCAGACTGGTGGGGGACAATACAATCCACAAGTCCAAGGTGGTAACAAAGCAGATGGAGGAGGAAGCATTAACATGGGACCACAAATTAGCGGAGGTGGTGGCTTCACTATTAATTAAAATTGAAGCTATCATCAATAATTCTACTTCTACCAATCAGTCGTCCATGTCTCATGTTTCATGTAGTGCATAAGCTGTGTCTTGTATCATCTATGTCATGGTACTTGAGTCAGGAGAAGTCTCTCCCACTATCGTGTTACAATAAATAATGTTAGTCCCTGTTTAAGGAAGATAATCA

>ONT.15186.2 LG10 87483630-87487500

AAAGGAGAAAACACTCTCCTCTTTCCTTTCCTCTTCTCTCCTCTCCTTCCATGACATCTTGAAGAACTTCACATGGAGGCTCCTTTTGCAAGTCTAACCCAGCCAAGCAACATAATCTATTTTGGTGCGGATCCAACAGTGGCAGCCCCTTCATCAGTCACCTTTCCTTTCTTTGAGCTTCAAATAGGTAACGGGCTTGTCTACGCACCAAAACTGAACCTTGTGGTGGTCCAGCAGGATTCGCTAGTTATTCAAGAGTGGGAGCTAACGACACAGAGTAGCGTCTAAAGCTGCAGAACTCATAACATCATCTTTTGGGTTTTTGTTATTTTGGGGAATTTCATCTTTTGATGATTATAATTATTGGCCAGTGTTTTTTCACGGCCTTATTCTGGGATGTAATTAAACTTAATATTTTGTTAGACCTGAATACTAACTGATAGAATGAATTCCTTTATGCTATC

>ONT.16179.1 LG11 34766444-34768424

ATTCCATTGTTTTTCAACCTAAGAGAAGGAGAGCTGAAGCCAAGGTTCTTTCTTCTCCCTTTCTTCAATCTCTTCAAGAGTTGGAGCTTTGCTAAGTGTTTTTGAAAAAGAGTTGCAGCACTTCATCTTCTTCACTAAGATCTTCCATTTGTTTGAGAGTTGAAGCTGGAGAAGGGGCTCAAAGTTCCTCCCTTGGAGCCACAAGCTTGGAAGAGGGTTTCATTGAAGGGATTTTGTCTCAAGGTCAAGCTAAACAAAGGTGCTTTTTTGTCAATTAATCTTCAAACAAGGCAAAGGCCGCACAACTAGCGGATGAGGCCAATCAAATTCCTCCAAGAGCATTGATTGAGTGGACGTGAACAGAGTGCGAAGGAATAGAACAGTTTGGAAATGTTTTGTGAAATTCTTTTGTATATTCGTTTTGGAATTGTAATGGATGTCACCTGCATTGTATTTTGAATATGCAGGGGTTGACTTAATTAGGATAAATGAGAAGTATGTATAAACTTTACTTTTATTGTAAAGTACTTGTTCAGTTAATTGTTTCGTCAATGTATATATTCTGTAAAAATGTACATTCGACAGGTATGAATGTATTGTTATCCCTTGTGGGTTTCGTTTT

>ONT.6896.3 LG04 74819281-74821270

ATTCGACTGTGGCTTGTTTGGTTGAGGAGAAGGACCTTTCTTCCTCTTTTTTTCTTTCTTTTCCTTTGCTCTTTTCCTCTCTCGATCATGAAAGGAATTGTGTATGAACATGTGGAGGTTAACTTGGATTTATTCTTCATCTTCTCCATCTTCTCCATCTTCCTTCTCTACTCTTGGTTCTTCCTTCTCTACTCTTGGTTGACAAGAACCCATCTCCATCATTCCCTACTCTTTCCAAATTCGGATTTATTGTTGAGTGGATTTGTGGTTAGGGTTTGATATTTGGGGGATTTTGGTTCATTTGAGCAAAAGATTAGAGAAAGGGCTTGGAAAGCTAATTGAGGCAAACGCTGGTGCAGCTCTCGATCCTGAGAAGGGCCAGAAGGAAGAGTAGGAGACCGAGGAGGTTCCTCTCGCATTAGAGGAGTGGCCAGATAATGGCCCTATGTAGTCTGGTCCTCGGAGAGTGATGTGGAAACTTTTGAGAACTTAATTGGAGAGTGTGACTGACTCATTTTTGTGTGGGTCACACTAGATAGATAGATGCAGAAATATATTTTGGAAATATGTTTTGTATAGAGTTGTAGGGTGATTTTTGTAATCCCTACTTTGGTTCTAATATGTAATTATGTTTTGTATTTGACATTTAACTTTAAATAGGATTGTTCAATGAAATGGTACCTAGTTGTGTATTA

>ONT.1731.1 LG01 123347375-123349512

ATCCGTTTACAGGTTGGTGTGGAGAGAATCCTTTCTCTCTTCTTTTCTTTTTCATTTTCTCTTCTTCCTCTTAATTTCTTCTTGCTCCAATCCTTGTGCAAGTGAAGAATAAGTGTAGAGATCACCTCTTCATCAACTCCTTCATCTTCTCCACCTTGCTTCAAGAGACCAATTGAGCTCTCCAACTCTTAATTGTGGGGTTCTTTGTCAAGGGTTTGAACCAATTGGGGATTTTAGGATTGAAACCACCAAAAGAGTAAAAGAGATTGAAAAGCTCAAAATCTAGGCAAACACAGATGCAACCTCCGGTCCTGAAGAGGTCCAGGAGGGAGAGAAGAGCACCCAGGAGATACATTCAGGATTAGAAGAGTGGCCAGAACCAGGGCCCGTTTAGAAGTCATCTTCTCGGAGTGCGAGCTACACTTGTAGATACCATAGTTCGTATGATTATTCTTTTCATATAGTCATGCGAAATTCTTTTGGAGTTACTTTTGGTAGAGATTGTATAGTGTTCTTTCGTTTACACCTCTTTTGTAACTTAATGTATATGCTGTTTATTCCTTTTGTAATACATTTCAAATGTTTAAATAGTATTTGATGGAATTTGATACCTATATAAATGAATTACGACTTCCG

>ONT.12658.1 LG08 62678450-62680234

AATGAAACCATTTCAACCCCTTTCCATCTGAAACCTCTCTCTATTAACGAAGCCCTTCTTCTCTTTTCCCTCCATCCGAAGCTCCCTCTCCCTCCAGCACCGACGCCGACGCCACGTCCGCCAGCTCTAGTCTCTCTCCAGCGATCGTTCGCCAAGCCCTCTCCCTCTGCATCTTGAGCTCTCCCTGCTGCACCTTTCCCCCTCCCGCGATCGTCCGCCAGGCCCACTCCCCCTCCATCCCAAGCTCTCCCTACCGCACTTCTCCCTCTTCCGTGATCAGATGCCAGGTTTATGGTGCAAGGCTGGTAGGGAGATGGTTAGAGAAGATATTGGTGACATAGCTATCAAAGATGCTCATAAAAGAGGAGAATCAGGAGATAGATGAGAACTCAAGGGGAAGGAGTTGGTGTATAGGGTGGAGAAGAATAAAGGTTTGGTGAATGCTGTTACAGGGCAAGTCGGATATCTTGATTGATCTTCCAAATAGGCCAAAGAATAATGCTGCTTTAGGTGGTGAAGAACATGAAGATAGAAGCTGGCGACTTGGATAATGAAATGGAAGAGTAGAGAAAAGTTTGCAAAAGAAAGTCACTGTGGTGTTTTTGTTTTTGTATTGTAGGGCTAAAAGATTCAAATTTGAACTCGCCAAGTTAGTGATAAATGTTTTCTGCAGGGGACCAGAATTTAACCTTCAAGAGTCATGCACAGCTGCACTCCCATAGTTAACAAGAAGCACTTCACAAGGCACTGTAATGGGAGAATAAGTGATCTCTTTTGTATACCATTGTCATGGATAAGTGCTAGTTTTCCAATTGAACAGTTTTGCTGC

>ONT.7791.2 LG04 87025626-87026956

TTTGTGCCAAATTCCTAAAGCAAGACTTCCACTCATGTTAGCCTTATCTTCAGACATTTATTTATGATTATTTTGCATTGTCTCCCTTGCATTTTTCTATATAAATTGGATCGGTGGCCTTATGATCTAAAATCCAGCTTCTAACTTCTCAATTGCAACTTCAAGACCACTTGTAAAATATAGATTCTAAGAAGGGCAATACCCAGGGTAATAATTCCGGAGAGTTGGGTACTCGAAATTGAATCCATAAACAAGTGAGACACACATGATGGTAGCAAGTTGTGTTTGGCCAATAGAAAGGAGACAGTATAAGACATAGGTGGAGTTCATTTTATCATTGGCCTGGCAGTATACTTATCAAGTTGTCCGTACAAATAACGGCTCCACATCCGAGTGATAAATAGCGACAATTCTTACCCGCATGTCTGGGTTCCTACTTTCGGTACGTCGTTGCTTCTCATTTATATGGTCAATGTTGCAAGGCAATACCCAGGATAATAATTCGGGAGGGAACAACATAGGGGGTAATAATTTGGGAGGGAACAACCCCCAGGTCCAAGGAAATATCAGGGCTGGTGATGGAGGTAACATCAATTTGGGACTTCAACGAGTAAACGTCGGGAAAAAAACGTAAGTGTTGGTGATAGCATCAATACGGGAAATCAAGGATACGGTGGAGGTAATGCCAATACGGGAACTCAAGATTAGGATGGTGGTAGCTGGACTAGCTGAAAATAAATGTCGCCAGTTATTTATGTTTCATATGTCTTATGTAATGCTTAAGCTTTGTCTAATATCAACTATGTCCGTTTACTACGTGTGATGCAAAACAACATTTCTGCATTTTGTGGATTCCAAGTGGCTATTGTGTAATGGTTGCTTGCTTATTAATATAAGTTTGTGTTACAT

>ONT.678.2 Contig01889 42556-44700

TCACAAACCTGCCTCTCTCTCTCTCGTCTCTCTCTCTGACTCTCGACTCTCGACTCTCTTTCGCTTCTCTCTCTCGGACGACGACGATCGGACGGCGACACCCAAAAAAACCCATCTCTCTCTGTCTCTCGCCTGCTCTCTCTCTCACGACGACGACGATCGGACGACGACTTTGAGATCTCTCTCTCCCACGATCTGTCTGTCGCCTGATCTCTCTCTCTGCGAAGCTCTCTCCCTCGCAGTCTCCCTCGCCTGATCTCTCTCTCAGCTCAGGTCCCCGCTGCTGCTCATCTCTCTCCCCCTCTCTGCGACGAAGCCCTGCTGCTGCGCACATCTCCCTCTCTCTCGCCTGCATCCGTCCGATTCACACGCCTGCAGCTGAACTTTTGAAATCTCTTGTGGTAATTGTTTGTTCTTCTTTTCAGCTATAATCAAAGTTCCTGTAGAATCCCTAATGATACCTCCAATTTCATTTCGACTGTTAATGTGAAGGGGTTATAAGACCCATCAAATTTCAATCTCACATATGATTGCAGGGAGGAGATATCGATCAGGACTCCTTGTCACAACCCTCTCTGTGGATTAAAGGGCTTATAAACAACACCAGTGACTCTGAGTATCAGATCATCTATCATCCATTGATTTGAAACGATAAGTTGGTCAGGTGGGTCACATGTAATTAATGGGATTGGAGAACCAGAAAACGAATACAACAAGTGCTTATTAACCCCGATGAGACCATGATGGGGAATTTTGTTATCAAGAACCAAACCAACCCGGTAAACTTGGATTCTCTAACGATTTGTGAGTTGTGACCTTTATCATAACAAATTTTTCCTCTGTCAAGGGAAGAGGAGATTGAAGCTTTTAGGGATAGTTTGTCTTTTACTTACTTGATATGAAAATCAAAATTTAGAACAATAGGTCCACAGTTGACTGCTATAACCACTTATTTATGCTTTTGGTTTGAAGCCTTGGTTGGAATGCCTAAATCACCATTAGAGTTGGTTTGCATAGGAATTCCGGGTTGGAATGCCTAAATCTTCTTTTTGTTTCCACGGTCTGTGATGTTAGATCTTTGATCTTGGGTTAGGGATCCATGAAGAAATTCAATGTGAGAGGACAATGTATGTTTGACGTTTCAGAAAATCTTAAGATGCTTCTACAAAATAATTTATAAGTGTTTTATGTGGGGACTCATAATGTGAAGAAAACCAGTCAGATGGAATTGTTTGCAACCTTTTCTTTTCCAGAGGACACGCCCAAATTTTTGTTTCGACAAAACGAGAGGAAAGGGAGAGACAATGGAGAAGAAGAATGTGGTCTCATCTACTCCAAGCACTAGCAGCAAAAAAGTGGTCATCACCTCTGAGAAGGCCTGAAGGGTTTGAAGGATGGTGTGGAGGATTTGGATCAGGTGGAGTTTTGTGAGAAACTGAGTCCAACTTGAAAGAGGCTGGGGATCATGATTTAATGGATTTTATGTTTCTTTATATCTTTTTCCTTGGAGCATTACCTATTATTCATGGACTTCAGATTGTACACAACAACACAACACTCTCTTAATAAAAATGTATGTTGATTGAGATCAACAA

>ONT.10955.14 LG06 69528930-69556585

ATCATCAAGGCCAATCGATCTCCAACTTCTTCTTGATCCTATATACCTTCAACTCCACCTTACTTTCATCTTCCGCAGCCATGAACAACCAATCTTGCAACAAATACACTCAGACAGGTGGGGGCCAGAACAATCCTCAAATCCAGGGGGTTGGCAAAATCGCCAACCAGGGCGATATTAATATGGGGTCCAGCACCGGTGCAACAAACAACTTCAACAACCAGGGAAACCGTGCTAATAAGCAAATCCAGGGTGTGAACGGTGGCTCCATCACGATGTAAACCATGCGTGAAGACAAAGAATTGGATGGATGAGAAGGTGGTGTCACCTTTCATTCTGACCTATTCTAGTCTTCCAGTTTACACAATCTTATGTTAATGTAATGGACTCGTTCTGCATCAGGGTCTGTATTCCCTGAAATAAATAAACAAATAAAATTGGGTC

>ONT.4900.1 LG02 109295946-109300361

TGTTGGAGATGGGAGAGAGCTTAATGAAATTAATGGAAATGTGATGGACTCTGGGGACATGAGGAGATGAAAAGGCCCACCACATCTTCCAACACCAATCTCACCGTTGGTCAGAGCCTATAGGAGAAATTCACCAGCAGTGCACCACACTACCAAGGAGTCATGAACATTGAGCAGTAGAGATGGTGAGGCCTTACAGAACCAGGCCAGGGACACATGGATATAGTATGGTGTAGAAGCCAGAAATGGAGAAGAGCCAGAGAGAACTGAGTGCTCGGAGCCTGTGGAAGTAGTCAGCTATGGCCAAACAGCATCTGGCAGCCTGAGATGGTGTGAGAATCAGGTGAATCCGATGAAGGGTTTGCTGCCTCAGATTATCAGCCTGTAAGAGAAATTTCTTCAAATAGCATTACATAATTTTTAATTAAAAAAAAATTCTACAGTGTAGAAGGCTATGATGATGAATAAGACTCTGATTGATGCCTGTGATTCTACAGTGTAGAAAATGATGAAATTGAACCCATACTTTCATGTCTTTATGCAGAATCTGGTAACCCCAAGAGTAGAACTTCTAATGATTGTTGTAAATATTGAGAAATTAAAAGATCAGGGTCTTTTTTTTTTTTTTTTTTTTGTCATTTTACATATACGATGGGCCATGGGCAGGTCCTTGGCAGTTGGCAACAAATAAAAAATGACCTTAGACAGTCCAAA

>ONT.12454.7 LG08 47133244-47135116

CTATTTCCCTTCACAGAAACTTACCGAGACCTGGCTGTTGAAGAAAGTTGAGAAGCCAAACATCATGTTGTTGTTGAAGCTCAAATCCCTTACCATTTTCGTCGCTGAAGGTCCTTTGCTCAGTTGCTTGAGGCACGCTCCTCCTGCCGCTGCTTGCTACTCCTTCCCCTTCTTCTCGCTTCTTTTTCCTCTTCTCCTCCTCCTTTCCATCCTCTCTCCTTCTTGACCCTTTTTCCGCATGAAGGAAATGAGGCAGCAGCAGATTTTCTTTCCCGAAAGGCATCCCCTCCCTATGGCGTTCTGAAATTCAATGTGGATGGTGCCGCTCATGGTAAACTGGACTGGCTGGGGTGGGTGGTGTCCTTAGGAATCATAAAGTTGAGGTGTTATTTATGTTCTCCAAGCATGTGGGAGTTAGATTCAAATGAGGGTTTTGGGCATTTTGTAAGATCTCTGGATGTATTTTTAGTCTGTCCATAATAGTTTGATTTTGGAAAGTACTTCATCCAACGCCATTAGGTAGATGAATTTGTAATGGGGCCCTTGGAAGATGCATTTCCATTTTAATGAGAATAAGCGTCTATCTTCAAGACGTTGGGTGTTGTTTCAGCATAGTAGTAGGTCCGCTAATGGCATGGCTGATGCCCTAGTTAAACAAGGGGTGAATCATTTTTTGTAATCCGAGCGCCTTCATGGTGCAGTTTAGCTGTTATGCTAGCACTTTGCTGTTCTGATTGGTTGTGTTGTCCTGTAATTTCTTTCTGTCATTAGTGAAGTTACCTGTTATGATTAAAAAAATGTATACTCCTGAAACATCTACTGTTTGTTTGTCTGCAGGCCGGCAGGGATCTTAGATTTCTTAGTACAACAATGAGTGGATGTCTTCTTTTTGGTGGCCTTTTATTAATGCTTTTGAGCAACAAAGGGTTTGGTTTGCGGGGCTTCTGGTGGGCACTTGCAGGATTTCAATGGGCTCGGTTTTCTCTAGCTTTACAGAGGGTTACTTCTCCTCAGGGTATGTTGTACTGTGATGAGTTCCAACAGCCTGAGTTTGGTAAGCTAAAAGCCACTTAGAGGAGCAGATATTTATAACGTCCTACAGGAGACGTTTGTGGTGGGTGAGCATCTTCTCCGTTCCACTGTGCAGTGGCCTCCATGAAGCAATGTTCCTGCATCCTCATAGTTGGTATGGATGCAATTGTCCAAGCAAGAGGTATAGATCAACATGATTAAGTCCAAACGGCTATGGGAATTGTTTCTTTTAGTTGAATTTTATCTTTCAATAGTTTCTTCCTTCCTAATTTTGCAGCATCTGTAGTTCAACTTAAACCATTTATCACATTGTAGATTCCTGTTCTTTAGTCGATTGGATATTTTT

>ONT.4706.1 LG02 57934408-57937440

ATCTTCATTTCCCTCTTCTCATTCTTCACTCCACACACAGCAGCCCCCATCCCCATTTTCTCTCATCTCTCCCGTTCTCTCCATCTCACTCTCCTCGCCGGACAACGAGCACGCCGCCACCGGTCACCCTCATCTCCGGCGGATTCCTCTCCTTCTCCCATCTCTTCTCCACTCTCTCATCTCATTACTCATTTCTCTCACTCATCTCTCCCTCTTCCCAGCTCACAGCAGCCCATCCCCATCTCTTTTTCCCCTTTCTCTTCTCATCTCTCAATCATCTCTCCCTCACCCAGCCATGCTGCCCATTGTTCCGGCGCCATCACCGTCGCCGCCGGACCCACCTGCACCGTCGCCGAGCTTCGCATCTTCGGCATTTTTCCCTGTTTGTTTTCCCACTTCATCTCTCTTTGTTTTCCCCCATTTTTCCCCTTCACCGTTCAGTCCTTTCATTCTCTGTTTCTCTCTTTCATTCTCTCTGTTTTCCTTTCTTTCTTTGTTTCTCTCTCTTCCTTTCTTTCTCTCTCATTCCTCTTGCTGCCAACGATTTTAGCCCATACCCATTTATTCCCCCTCATTTTCTCATCATCTCATTTCATATTCTCTCATCTCACCTTTCCCTCACCTCACTCCACTCACTGTCAACTCACGTTGACTTCTCACCCCCATTCCACCATTTCTCCCTCTCTTTCTTTAATTGTTTTTATTTTCTCCCATGGCTGCCAACATGACTATCCCACTCCACCATTTCAATTCTCACTACACTCACTGTCCACATGGGTTGACTTTTCTCCCCTCACCCATCTCACCATTCCACACTCTCTCTCTCTCTAATTCAATATCTGTTTCTTTCTCTCTTTATTCTCCATTCTTTATTTCTTTCTATCTTCACTCTCCTTTTGTCAATGGACCACCAAGATCTATATCACCACCAATCAGTGGGCCACCAATCAAGTGGGCCCCATAACCTCCGATGATCATTATCATCTCCGTGTGGACCCCGCAAGCTCAATCATCTCCAATTTGCAAGCCACCACTCCATCCACCCATTTATTTGGGTTGGTTTGTTGGACTATGGAGCTAGGATTTGTGGATTTGTGCAATTTCAACCAAAGGTACCGGGTACATCTACCTTTGGGAAAGTGGAGGCGTCGCTTCAGGCAGTTTAGCCTGATGGTGAGTACGAATTGTGTGTCGACGGTATTGTCGTCATGACCAACACACGAGTGCCTTCATACTTTGGTTAGTCGTATTTATTCTCTTTTGGCTTAGGTTTTGTTAGAAGCCATTAATTAATATACTTTTGTTACCTTTTACTAAACTGAAACTATAGAAAAGAAAGATGTGCATACTCTGAAATGTTATATTGTACCATCTGTTTAG

>ONT.10108.10 LG06 17581352-17582542

AGAAGTGGTGGCAGAAGCGGCGGTAGATTTGGTGGCAGAGACAGTGGTGGTCGATTTGGCGGAAGGCGTGGAGGTGGCCGAGGTGGTGGTGGACGTTTTGGTGGAGGCGGCAGAGGCCGTGGAATGCCAAATAGGCCAAGCATAGGTGCAGATTGCAGGAAAGAAGACTACATTTGGTGATGACTAGAGGGATGGTGGATGTGCAATATCACCTTTCATTTATTTTCTGCGTGAAGAAGAATATTAGTTCTTTATAAGGCTTAATTATGCATAGTTTATCAAAAGATTGTTCTGGGATTTGTTTTAATTAATGTTTTGACCGGTTTTTGAGCAAGGTGAACGGTTTAACTTGGACCAAACCGTTCAATGCACCGGTTCCATATTGTGTTTAATTACTATTTAGGTTTGTGTTCT

>ONT.3745.2 LG02 110461891-110462472

AGAAACCCCTCTCTCAACCCCACAACAAACAAAACCCATACAATAGAAATCTTCATTTTCTTGTGCTTCTGTTGCTCTACCCCAAAACACACAAGCACCATAAAATGGAAGGTCAGAAGATGAGATTCTCCTTTGCCATTGTGTTGATGGCCATAGCCATGGCCTCTTCCATGGTCCCACTTGCTGCAGCAGCTGACCCACCTGCACCCAGTCCCACATCTGCCTCTGCTGTGTTTGGCCCAGCTCTTTTTGCCTCCTTCTCTGCCCTTCTCTTTGGGTTCTTTTTCTGCTGATGTTTATGATCTTATTATACCTGGGTTTTGGTTATTTTTGATGGGTTAGAGAGAGGGAGAGAGAGATGGGGTGATTGATGTATGATTCTTTCCTGTTCACTGGTATTGTATCTTTGTAATTGGTGTGTGTTGTAGAATATATTTATATCTGTTATATAATAAAATTCTTTTTTTTTCT

>ONT.1301.1 LG01 48975925-48976815

AATAGTTTCTTGGCCTCCATCACTAAACAAGAAAGAACAGGCAAAAGAGTTGTAAACCCCACGTGCGTTCCAAGCCTCTGTCATTTCAAGGTGCTTCTGTCACTTTAAAGAAAGAGTGGTGGAGTCAGCAAAGAGCTCTCATTCCCGCCATGAACGATGATCTTTCAGTCACACAGGTCGTCAGATACAGGTGAGAAGAAGATAGGAGGAAGTTCCAAGGCCAAATGTGGTGCCGCAACTTGTCCAAGCAATCCCAAAAAAGTTCGCAAAGGGGCCATTGAGTGCATGATCTGTTACGATTTGGTCCGGAGTTCTGGTGCAATTGGCCAGTTGACAATGTTGAATTTATGTGTTTTTTTAGTTGATCTGACTTGTTTGATAGCACAGAGTACACAAATTGGATCATAACTTAATTACAATATGTAGACATTGTTGAGATACACTCAAAATTTCAGTTTAGATGTGACCTGAATTGC

>ONT.6379.1 LG04 656424-657673

GACAAGAATTCACATGCTTTTTTCCACACCTTACTGCCCTAAAAGTTAAAGCCCCACTTTCCCATCCTCAATTTCAATTGAAAAAGCAATAGACTGAAACACCAGAAAGGAAGATGGCAGTGGATGTGGGGAAGTTTCTCCTGGAGAAATTGCACGAATTCCTGCTCTTATTGCAACATCTTTGGGCTTGCGTGCTCAAGAAATTCAATGAGGTGTTCCCCCCTGAGACGAGGGCCGAGAAGATAAAGCACTGGCTCCAAATAGGATGGCCCGTCGGCTTAGCCCTAGCCGCTGTGCTGCTCCTATTATGCGTTTGCTGCTTTTGCCCTCGTTGGCTGAGGTCCTGTTGCGGTTCCTGGGTTAGGTGCTGTCGCCGATGTTTATGCTGCTGTTGTGGAAGGAGGGGAAGGATGATGAAGGCTCCAGGAAGGGACTACTTGATGCTTAGAGATGATTTCGAGAGCGATCCGAGATCCTACTTCATAGATCTGCGTAGTAATAGGTAATAGATAGATGGCTCCTGTTTGGGGTCTGGCTATATACTTGGTGTGTTATTAGTGTTTTAATCTTAATATGACTTGCTTTTTTACTTGTATTGTATTGTACGCATGTAGAGAGTGCTTAATTGGGCTTCATGGGGCTTGTTATAGTGTGTTGGGTGGTTTTATGTTGCATTTGGCATCATATATAGAGGATGCAATGTTATTGTGGTTGT

>ONT.14215.3 LG10 488222-490514

CAGGCGAAGTTCCTCATCCTTCACTCCTCCATTCTCTTTCTCAAATCTTCAGAACTAGGACTTGCACGTGTGTTGAGAGTGTAGATTGGGACTTCCTTATCATCTTCCCTGACTTTCAAAACAATCGTGGAGCAAGAGTCTTCTTCATCAACTATCTATTTCATATAAAAGGGTGAGAGCTCTGGCTACAATTTAGATTTTTGGGTTTAATTGGGTCAAAGGATTTCCCTACACTACATATACAGGTGCAGCTCTCGGTCATGAGGAGGCCCAGGAGAGAGAGAGGAGGAGACCGAGGAGGTTCCTCCCGGATTAGAAGTGTGGGCAGCTAAAGGCCCTATCTAGTCCTTTTTCCCGGAGAGCGACGTCTGTACTATTTTGGGATATTTTGTAGTGGGTGTAGGTGATGTCCATAGATAGTGATCACCCTCTTTGGTGGGACTCACTTGTTAGATATTGGAGGCACTTTTGAATATACTTTTGTTTATCTATGTAGGGTGTACTTTGTAATCCCTACCTATGTAGTTAATTGTAAATATCTTTTGCGTCAATATGGAATGTTAAATGGACTGATCATGAAATGATACCAACTCTGGGAA

>ONT.5303.1 LG03 24715330-24716978

GATACGTTTTGTAGAAAAGAGGTCTCTTCATCTCTCATGGTGTCACCCATCTCCATCTCAATCTCCCTCTCCCTTCTCCGCCTTGGTATCAAAACAAATTCTTGCAAAATGTCTGATGTTTTTCGGTTTGCCAAATTCCCAATGCTTTCCACCTTTCTTTTTCGCTCCAAAACCTTAGAAAAAACCCTAAAGAAAATTTTCTCTGGTTGAAGAAGCTACAACAACTGCAACGGCACACACAAGGCTGCCTTCCCTTCTCAAGCAATCTTGGGTTTTCCAGAGCTAAAGATTGCTTCTTTCATCTATCTTGAATTAATTTGTAGGTGGGTTTTATTCAATTTCTTATGGATTATGGATTTTGTGGGTTTTATTCAATTTCTTATGGATTATGGATTTTGTGGATTTTAGTTGCCACCTTTTTTGTTCTTTATTTTGATCTTTCAGTGGCTGACAAGGTATCATTCTCCTGCTGCTTCTTTTCTACTTCCCAATTGATTTACTTTCTTCATTTCTTATTTTTATAATTATTTCTAACTTATTGTTCTTATTTATTATGCAGAGACTTGAAGAGATATCACCTGATTCTGCAAATTTCAAATGAGATTGGGAACTGTACTTCTCTGAAACACCTATTTCTGTTTGGGGTTAGTGATTTAAAAAAATTTGTATGTGTTCTTCCTGTTAGACGATGGTGGTCCCATGACCGCACCTCTAATAATCATAGGGCACCATTTTAAGTCAAATAATATGCTTTCCCCTTTTAGTTTTGATTTGATTAAATCTTTCTTGGATTCAATTTAGAAAAGATTAGAAAAGCCTCATCAATCAAGATTCTTGGTTGACCAATCATTGAGGAAACTAAGGAAACTTATTGCTAATTGATTTCTCCGATGTGAAGAAATCTCTCCTGTGTTATTTTATAAAAAGGGTTTTTTGGGCCTCTCTCTTTAACTCATGTTATTTTCAAATTACCCATGGACTCTCACACATGTTGGAGTGAGTGACAAGGGAAATAGGAGAAAATTCAAAAGCTGTTGATCTCACAGCGGGAAAAGTTTTAGATCAATCCATACCGCTCATCATCAAGATCATGGCTCAAGGGGTTTACCAAGTAGCATGCTTTACAGAGACATCGATCCCTTCTGCGACCTCTAAGCTTAAACAACTTGAGGAATTGTAAGGCTTTCAAGAACAATAGGCTAATAGGGGTCTCATTCCTTCAACTCTGTCACATATTCCAAATTTGAAGAGTATGTGAGTTCTCTTGAATTCAAGGATCATCTCTTGGATAATGTTGTAAAGTTGTATCATTCATGAATTCCATCTGGGTCAAGTTTGATAGATATTTTGGAGCTTTTGGTTGA

>ONT.5955.2 LG03 37548029-37549065

ATCTGGACATGCAGCATGCCACATGGCAAGAGGCAGGGAGCTATTTATCTTGCTCTGTGCACTCTACCACTAGTTCCAATAGCAACCCAATGGCTACTTCTGCTTCCATTTCCCCCTTCCTCAAATCCAGAGTACTTGCAAGGGATCAAAAGGAATCACAGATCTGAATCCTATAATTCAGATAAGCATTTGATACTTATTTTATGGTTTGTTTGATATTGGCTACTGGTGTAATGAACAATTATTGTTTGCCAGGATATATGTGGTGAATCTCTAGTTCAGAACAAAGATGAATTTCTCAAAACTTTTTCAACAGAAACTCAGTTTTTCAGAAACATCATCTCAAATGGAGAAGTCATAGGCTCTGAAGCTTCCAAGGGGCATGATGATGCATCTTATAGCTATTTGGAACCAGAGGCTTCCATCACGGACGTGCTCTCTTAATTTTACTTTTATTCTCATTTGCATTTTGTAATTTCATGATGCTATCTGGCTTCTATGTCTAGATTTTTCAGTTAAATAAGCATTTCCAAAAGTAATGTTCTAGACTTGACCAGAATGTTTGTGGTTATGATATTTCATCCTGCAGCTGATTGAT

>ONT.10128.3 LG06 27624879-27627995

CTCTTCTCCCCTACATTACCATCGTCCAGCAACAAAGAGCTCTGTCTTCCCCTTTCTCATTTCTTCTCCTTTTCCATGGTGGAGATCATCTACTAGTGGACCCTGCCTGCAAGATCCGACCCCGCCAAGTGATTCTACATCCTCTCCTTCACAAGATACATCGTTTTCACGAGGTTCCGTGACCGGCCATCCCATGATCCCTCTTCTTCTCCTTCTTTGAGCTTCTTAGAGGGGCTTTTTGGAGGTGGATTGTGAAAGGTTTGAGGAGGCGTTCCAGCTGAGATTTGGACCATTCGTTGAGTGCTTGGACTGCTTTGGAGCGATCTTAGGCCAAGGTATGATTTCCTGACATGTACCCTAGTACGGTTTTTGAGGAGGCGGTTGCTGAGCCACAAGTGACATGTGATCAAGATGCAGAGGTTGTCATTTAGACTCCACCCCTTACACATGAGACGCGAAATACCTAGGAGTATTATTTTGGGTTTTGTATTATGTTTTCCTGGCCTTTGTTGGCCTTTTATTTTGGAGAATGCAATTATTACTTCTTTTGTAATTTCTAACTAGAAACATGGGAATGATTTATCCTGTTTACTTGTCTTGCTCTATTATCCTCTGTTTATATTCATACC

>ONT.13457.1 LG09 48086849-48089206

ATTTCATTCGTTTGATATAAGGGACTGTGGAGTGAGTGGAAAAACAAAACCCTTTTTCCTCTTCTTTCTTTCCATTCCCTTCCTTTCTCCAAACCTTTTAACCTTGCACAAGTGAAGACAAGTGGAGGGCTTCATCTCCATCATCTTCTTCTCTTCTTTGCTAGCAAGAAAGATAACCCCATCATCTCCCATCTCCTTCAAATTCACAATTATTACTAGAGGGTTTGACTCAAATGGGGATTTTAGGATCTTGACCATCAAGAGTTTAGAAGAGGTGTGAAAAGCATAAATTGAGGCAAACGCAGATGCAGCTCCCGGTCCTGAAAAGGTCCAGGAGAGAGAGGAGAGCACCCAGGAGGTACAGTCAGGATTAGAAGAGTGGCCAGAACAAGAGCCAGTTTAGAAGCCATCTCCTCGGAGTGCGAACTACACTTGAGGTCTGGCATAATAACAAGAACAGTCCCCTGAACAGCTCTAGTGATTTGACTATTCATAAAAAAAGAAAAAAGAGGGGCGGGATGCAGTAAAATTGATATTTATACAATTTAGTACTACTTTTCCCACCAGTAAATTTGATATTTATACAACTTAGTTCTACTTTCAAAGTACCAA

>ONT.13855.3 LG09 37834263-37860858

ACGCTTTTTAAAGCTGGTGTCAGTTGTGACCACCATGTGTAGCCGGTCCATGCACCCAATGAAGAAAGCCTAGCTGATGGTGCTATGACGCATGAGAAAGACTCAGACGTGCAAGAAGTGAGAAACCCTATAAATATATAGGAAGCGAGAACTCACATGCTGGGAAACTACTCACCTCTCATCACCATGATGTATTTTCTTAATCACGGACAATAAATGCTGTGAACCCATCTTCCTTGTACTGGCCTGGCGGTTTCGTTCCTGTCGAATTTCAGAGGGCTGTATAGAAGCCAGGTGTAAGGGGGAACAAACACTCGTTCCTTTGAAGCTAGAAAGTTACTGTATCTGTTGTTTTCTTCCAATTTGAAAGGGGACATTAGATTTTGACCTTTGAAGTCATCAGCGGGTTTGAGAGCATCGGACATACCACGGCACCCGGAAATTTTTTTTTTCTTTTTCCTCTAAATGTAATGGATATATGTTATTGAAACTTTTGTAATTAATGTTTTGAATGAAATTAATATAAGATTTTATTTTTT

>ONT.5307.2 LG03 25766648-25769789

GTTCCAGCAAGAGCTGGAACCACTCCTCTCTCTCAGCCACTCTCTCTCTCCTCTCTCCCTTGGCTTCTCTTGATTGTAAATGGAGATTTGATCTCCTCATCATCTTCCATACCTTCCAAGCTTTCCAAAGATGTTTTTTTCATGGATTTTGAGCAAAGGCAACTTCTCTTTTCAAGCTATTCTTCTTGGTTCAAGGCCTTAAAGGGTTTTCATGGGAAAATTTGGGGGTTTTGGTGGGTTTGGAGCTAGAGACTTCTTTGAGGCTTTTGTGGAGTGATTTGTGCATCTTCTTATCTAAGGCAAGTTTCTTGTTCTTCCTGCTTTTCAGATGTGTGGATCGGGGGAGGCGGTAGAGCCACCGGTCATCGTGAGAGCCTACTTAAGGATATCATATAGATGTCAAGGTGGGCAACAGAGTTCGTAACAACTGTTGGGATTAGTATTTGTTTAGACATTTTATTTTGGGTATGGCTTTTATTAGCCTTTTCTTGGGTTTTGTTAATGAAAATACTTTGTTTTAATTGTAACTTGAATATTCATGGGAAATAAGAATATCCTGTTACTG

>ONT.2364.3 LG01 74634860-74641180

GCCCTCAACAAAAGATCCCTCATCTCCAAAACGAAACAAGGGTTTGGTTTTAGAGAAAGGGTGACGACGATACATGAAACATCCAGGGACTAAACGTTTTCCGTTACGAACGAATTCGAGTATGTCGAGAAGTTGGAAAAGTGGTAGTCACTTCATCAAAATTCTCATGGGAGATTTCTCTCAGCGTAGGCACAGCTGCTCTTGCCGATCCTTCTAAGACCACAGCGCCTCCGGAGACGTCCGGCGCGGTTCTGCCAGGAGGACTAGTTGAATGGAAATAAAACTTTGAATTGAGATGTGTTTGAAATGTTTCACAATTTTGATTCCTTGCATTGTATTTAAATATGCAAGTGATGGAGTTGTATATAGTATAAAGAACTTTTGGGTTTCTACCAGCAATGTTTATTTGTGAATGTTTCCGCATAATGTATAATGATAGCCCT

>ONT.85.3 Contig00262 32474-33233

ATCTGAATCCCAACGTAGAGAGAGAGAGAGAGAGAGAGAGAGATGGCTAGGTTTTATTGTGTAATGTTGGTGCTTGCACTAGTGCTTGTTCATACCACTGCAGCTAGGAATGTACCTAGCAGTGAGGTGGTCGACACAAGCGCCAAGAAAACCCCAAGTAACGCCGGTCTCAAGGACGGGAAGAACTTCGTCTTCGGCGGTGGCTTTGGTGGTGCTGGTGGAATTCTTCCTTGATTTTGGTTGAGTTGGTAGTGTGAGAAGTTGGGTTTGTTTTAGTTAACTTGTTTGGTGGCTATGTAATAGTCCAAATCAATAATGTGTTGATGGTGCTGGTTTTTAAGATTAGTAATGTTTTAAGAATAGTTGTTATGTTCTAATATTTCATGATTTGCAAATGTTGCATTAGTTACGTACTCAACAATGTTTTGAGGAGTTGTCATGTTCTAAGATTTCATGAATCAGTTATGCATTACTCATTCCACAA

>ONT.7804.2 LG04 88140472-88146625

GTACAGGCAGTTTCTAACTTCTCAATTACAACTTCAAGATCACTTCAACTACAGCTGACTTTCCATGGACATTCAATTTAGTCTTGGGAGCTCTGATAAGTCACCACCACCCCAGCCTAATAACACGACCAAAAATGCCCAGACTGGTGGGGGCAAGTACAACCCACAAATCCAAGGAGGGAACAAAGCAGATGGAGGTGGCAAGATCAATATGGGACCTGAAATCACTGATGGTGGAAACTTCACAATTAATTGAAAAATAGCCCCTACTCAATGAGAGTGCTGAAGCTCCCTCAGCGCTTGCTGTTACTTATGCCTTATGTAATATGAAGCTCTACCTACTATCATCCAAGTCAGGCTCTCCAGATGTAATAATACAATAATTAATGTTAGCCTGTTTTTACTAATGATAATAAGAAGATAGTATATGTTGGGATCTCATTCTCACC

>ONT.459.2 Contig01280 23052-41377

GTTGTTCCAAGCTCTTGCAGCTATTGCAATTTCTGCAGCTGCGATTAGATGTTGCTGCTTGCTGCGACTGTCGTGGCTGGTGGTAGCTGCTGCTGGTGCTCCTGCTCTCCAGATTTTGAAGGGATATATCTGAGAAATAGCCAAGTGGATCATCTACAAAGAGCACAAGTGGGAACAACACAAAGAATGAGGGGTGCTTCTGTTTGTTGTAAAAGCCGCTAAAAGTTCTCTTTGCTGGGGATCATCTTGCCAATAGAGTATATGGGGACTCTACTGGACTGTTAATTTACGAAGGCTACAACTGGTTTTCATATTTTAGATCTACTTGCACCTTACCAAAGAGGTGGAAAGAATGGATTGTTTGGCGGTGCTGGTGTCAGAAAGACTCTGCCTATCATGGAACTCATTACAATGTTGCCAAAGCTCATGGTTGTATAATTGTAATGGAATTCTGTGTTGGAAATACATAACTCTGTTTATACTTATGAAAAGTCATTGGGTTATTCTAGTTATGTTAATATCCATCTTATAGGGACCTTTCTCATTGAGATAGTATGATTTTCTTGGTTAGGTTATGGTTATGTCAATGAAGAATTTAAATTGTTTTTTGGATGCTACTCTTGTCAACAACTACTGCTTGTTACCTAAACTTTAAGAAGGGTGAGAGTGCTGGGTGTTTATA

>ONT.456.1 Contig01277 64145-65008

AGGCAGTTTCTAACTTCTCAATTACAACTTCAAGATCACTTCAACTTAAGCTGATTTTCCATGGACATTCAATTTAGTCTTGGGAGCTCTGATAAGTCACCACCACCCCAGCCTAATAACACGACCAAAAATGCCCAGACTGGTGGGGGCAAGTACAACCCACAAGTCCAAGGAGGGAACAAAGCAGATGGAGGTGGCAAGATCAATATGGGACCTGAAATCACTGATGGTGGAAACTTCACAATGAATTGAAAAATAGCCCCTACTCAATGAGAGTGCTGAAGCTCCCTCAGCGCTTGCTGTTACTTATGCCTTATGTAATATGAAGCTCTACCTACTATCATCCAAGTCAGGCTCTCCAGATGTAATAATACAGTAATTAATGTTAGCCTGTTTTTACTAATGATAATAAGAAGAGAGTATATGTTGGGATCTCATTCTCACCAAA

>ONT.11891.1 LG07 24976279-24979432

ATCCTTTTCCCTGCAACGGTTCCAGCAAGAGCTGGAATCACTCCTCTCTCAGCCACTCTCTCTCTCCTCTCTCCCTTGGCTTCTCTTGATTGTAAATGGAGATTTGATCTCCTCATCATCTTCCATACCTTCCAAGCTTTCCAAAGATGTTATTTTCATGGATTTTGAGCAAAGGCACCTTCTCTTTTCAAGCTCTTCTTCTTGGTTCAAGGCTTTAAAGGGTTTTCAAGGGAAAATTTGGGGGTTTTGGTGGGTTTGGAGCTAGAGACTTCTTTGAGGCTTTTGTGGAGTGATTTGTGCATCTTCTAACCCAAGGCAAGTTCAGATTTCTTCGTCCCGATATGAGTGTTTGGTTGGCTATGGAGCCATGTCTTGCAGGTTGTTTTGTCGGCTATGTCATATAGACATTGGGCTCGACATCGGAGCTACGTCACATATGGGTAGTAGTTAATAGCTTATTTTGTATGGCTTTTATTAGCCTTTTATTGGGGATTCTTAATTAATGTACATTGATAATTGTAACTTGAACACATTTGGGAATGTAATATCCTGTTATTGTCAGCTC

>ONT.17201.4 LG12 42404408-42405412

ATCCAATCCCTTTAGTTGCCTCTGGGTAGCTTGCAACGAGGCTACTTACTACCTTTCTTACTCTGTTCCTGTAAGAGTTTGAGGGAGAGTTATGCCACCTCCTCCACCTGGACCTGGCCCTGGTTGGGGCCCTGGGCCTGGAGGCCCGCCCGGCTGTGGACCTTGCTGCGGTCTCTGTGACTGTTTGTATAGTACAGTATCAACTTGGTGGGTATATGTATTTTGTTTGGAGCTTTGCTATGTCATTCAAGGGATTTGAGTTGAGTGGAAATTCAAAACTCACAAAAGGTGACCCAGATAGATGAGATAGCGCTTCGATGTTAATACAGTGGATAGGCCCAAAACAACTTCAGATGCCCATTATATCTCCTGTTTGGTGCATCTCAGAAACATTGCTCTTGATTTTCTTTGTTCTGTGGGAAAATTATCATCTTCTACTTCTTGTGCTGTTGCTGCTTGTTTCAAGAGTGCTGCGGCCCATGGTTCGGAGGGCCTGGTGGGCCCGGCGGGCCACCTGGTGGGCCTGGTGGGCCTCCTCCATTTTGAAGGGGCACTTGGACCTGTTGGACTTGTACTGGAATAACCTGTATGTGCTTCATTGTATAGGATAGGAGTTGATGTCCACTGCTCAGTTTGTTGGTGTTTTATGAATACATATGATGCATGTCTAGTTTCCTCCAAGTGACTTTGTTGAGTGTGGGTGTATGCCAGATAAATGACTTTGCGTAATATAATCAGCCCTAAGGGCATATTTGTTTATTTAAAA

>ONT.2644.1 LG01 117252989-117261438

AGCTACAACTCACTCTCTCATCCCTTCCCTTCCCCACCCCGCAAAAAAAAAAAAAAAAAAAAAAAAAAATCTATCTCTCAATTTCCCTCCAATTCTCTCATCTCTTCCATCTCTCTCTAAAACCCTAGATCTAGAACCCAAACAAGAAGGAGAAGATTATCTACTACTAGCTACAGCCACAATCTCTTCTAAAGAGCTCTCTCATCTTCTGTATCTCTTCTCCCTCTTCTAATCAAGGAGTTAAAGATTTCTCTAAATTTGAGTTTTGGGGCTGGTTTGTGTGTTTGGAAACCATAGGACTTTTGGATTGCATTTTTATAGCTTGGATCTCTCTTTCTCTCTCTACCTTCTTACAATTGAATCAAGGCAAGTTTCGACCTCGATTCCGAGGACGAAGCGCTCGCGCTAGTTGAGGCACCCATAGTAGGGGACCAGCAAACCAAGATGTCGAGTTAGAAGTTGGAGAGCCAGCACATGGGATATGAGAGTTCTTTATTATTTTGGGATTTTAAGTGATTTTTGTAGTGAGCAGGGTAATTATTAGAAATTGTAATAATCTTTTGACCTTTATAACTTTTACACTTTAGGTTGGATTTCAGCCCTGCTATATTTGTTATAGACTTATGATCCCTTGTCATTGCTTC

>ONT.673.1 Contig01889 39596-42103

CTCACAAACCTGCCTCTCTCTCTCTCTCGTCTCTCTCTCTGACTCTCGACTCTCGACTCTCTTTCGCTTCTCTCTCTCGGACGACGACGATCGGACGGCGACGCCCAAAAAAACCCATCTCTCTCTGTCTCTCGCCTGCTCTCTCTCTCACGACGACGACGATCGGACGACGACTCTGAGATCTCTCTCTCACGATCTGTCTGTCGCCTGATCTCTCTCTCTCTGCGAAGCTCTCTCCCTCGCGGCTGTGTCTCCTTCGCCTGATCTCTCTCTCTCAGCTCAGGCCCCCGCTGCTGCTCATCTCTCTCTCCCTCTCTGCGACGAAGCCCTGCTGCTGCGCACATCTCCCTCTCTCTCGCCTGCATCCGTCCGATTCACACGCCTGCAGCTGAACTTTTGAAATCTCTTCTGAGGACACGCCCAAATTTTTGTTTCGACAAAACGAGAGGAAAGGGAGAGACAATGGAGAAGAAGAATGTGGTCTCATCTACTCCAAGCACTAGCAGGTTCACTGACAATGGGAGTATAATAGTGGACATGCAAAGGAGTAGATAGTTGCAAAACTTTGAAGATTTTGAAGTCTTCAATGATACATGTTGAAGGGGTTGAATGTTGAACGTTTATGTAAAAATGATGATTCATCTGTTTTTTTTTTTTTTTTTTTTTTTCATTTTTCTTGTAGATAAAAAATGATGTATCAGATTCTAGTGTTGAATGTATCAGATTTTCCTGTATGTGATATGCTGTAGCATATTTTATTATTTGTCATAA

>ONT.7804.4 LG04 88145655-88146625

GTACAGGCAGTTTCTAACTTCTCAATTACAACTTCAAGATCACTTCAACTACAGCTGACTTTCCATGGACATTCAATTTAGTCTTGGGAGCTCTGATAAGTCACCACCACCCCAGCCTAATAACACGACCAAAAATGCCCAGACTGGTGGGGGCAAGTACAACCCACAAGTCCAAGGAGGGAACAAAGCAGATGGAGGTGGCAAGATCAATATGGGACCTGAAATCACTGATGGTGGAAACTTCACAATTAATTGAAAAATAGCCCCTACTCAATGAGAGTGCTGAAGCTCCCTCAGCGCTTGCTGTTACTTAGGCCTTACGTAATATGAAGCTCTACCTACTATCATCCAAGTCAGGCTCTCCAGATGTAATAATACAATAATTAATGTTAGCCTGTTTTTACTAATGATAATAAGAAGATAGTATATGTTGGGATCTCATTCTCACCAAA

>ONT.9613.1 LG05 54846917-54869400

GCAACCGGCCAACAAACTTGAGAGAATTTCTCTCTTTTCTCTTCCTTTCTTCCCTTCTCCTCTCCTTCCATGGCTTGTTGGAGAACATCTTCTCGGAGGCTTTTTCTTGTCCAATCCAATCCGGCTAATCAACTCCCCATCATCTCTTTTGCAATATATTTTGTTTTGATTTGAATCCAACGGTGGTAGTTCCTCATGCAACCACCTTCGATCTTCTTCCTTAATCTACTAAGAAGATGGATTTTTGTTTGGAGAGATAGCTTCCGGTTGAAGCTTGGATTGACTGAGGTTTGTTTCCCCGACTCATACCTTGGTGTGGTTTTCAAGGCAGCGGTTGCTAAGCAGTTAGCGAAGTGTGATTTTTGTGTAAAGGTTGTCATATAGACTCCACCCCTTTCACCTAAGACGCAAAATACATAAGAGTATTATTTTAGGGTTTTGTATTCTATTTTACTGACCTGTGTTGGTCTGTTATTCTGGGAATGTAATTAATATATCTTTTGTAATTATAACTTGAATTCGGGAATAAAATCTCTAATTACTTGTCT

>ONT.7482.4 LG04 24334950-24347053

GTTCCCAATTTTTTCCCCTCCCTCCCGCGCCCTGTCCTCCCGTGCACCAGCGACGACACTGCCGGAAGCCGCCTACCCCGGAGCTGCCACCGGTGCTCCCTCCCTCCCTCCCTCTCTCTCTCTCTCTCCCTCTACCTCTCACTCTGAACTGTGCCCTCCCGTCGCCGGTGCCGCCAATCCGTCCTCATCTGCGATCCCGACTGCCCTTTCTCCGTCACTGCTTTCGACGCCAATCACTGTCCAGGAAACCCTCTTTTTGCTCGCAGCATTCAATCTTGCTACAGCTGCTGGAAACCCCGTTTTTGCTCGCACCATTCAATCCTGCTACAGCTGCTGATGCAGGGGAAATGAATTTATATTAATGAAAGGAGCATTTGATTTCTTTCGCTTAATTGGCTGGTCTCCACTGTAGGTCATGTGGTCGACGTTTGTTCCCTTTATTCCCAACAGCATTTTGCCTGGGCTCCTCACATTCATTGCAGCAGTGGCTGCTGTTGTTATGGACACCAGACGCTGGCAGCGGCCACAGGGCTAGACACGACTTCGGTGGAGAGGGCAGTCATGATTTAACAGGCTCGAGTGATCCTGATCAGGTTTAAGAGCATCGGATATACCACGGCACAGGGCAATTTTTTTTTTCCGTTTTCCTGTAATTGTAATGGATATATTTTATTGAAACTTTTGTAATGTTTTGAATGCAATTAATATAAGACTTTCTTTTTTTATTTGCAATTT

>ONT.4679.1 LG02 48590600-48598916

CAGCAACAAGATCCTACTTTCCCGTGGTAGAGAAAGAAAAGCTCCCGCTGCACTTTCCCGTGGTAGAGAAGGACGATCAGCTTCACCGGTTCTCTCCATCTCCCTCTCTCTCGTTCTATTTCTCTCTCTCTCTCTCTGTCTCTCTCCCTCCCTCCCTCCCTCTCCCTCTCCCTCTCTCCCTCCCGCCACCGGTTCTCTCTCTTTCTCCCTCCCTGCGCCAGAGCCGCCACCGGAGCTCTGCATTGTCCCACCGCACTCATCTCGTGAGCTCGATTTGGTTTTTCTTTACTGCTATTTCGGTCTCAAGGTCGTCAGGCTCTGTGCCAAATCAAGAGAAGCTGGAAGCTCCCCTGTTGAGCATTTAACGCTGCACTATCAGGTGATTAGGATAGTGATTATAGTGGCATGTGGAACTATTTTTTACAGCACTAGACAAAGAAAGACCAGGTAAGCAGGTTATGTGAAGGAGGACTTTGAAGAGGCTGCAAGACTGGAAAAGGCTATCACAGAAGCAACATCAAAAGACACAGTGGCAAAAAGTATGCCTTTTAGCATTAGGATAAGTGCAACATCATCTACCTATTTATGTATTAGGTACGAAACAGATTAGCTTCCTCTCCATGGAGTCCTGGGTTTCATACAAATCAACTGCAGGACTCTTCTGTAGAAATTTGTCATTGTATAATCTCCTACAACTTCTAGTTCATTGCTCCTTAATTGTCTAGTCTGCACAAACAAGGCAGATAGATATTGAGTGGAGTTTCTGTTGTAAATATTGTAAGCTGCAATACTTATAGAGTAGCAGCATATCAAACCACTTGAGTAGAAGAAATGAGGAGGTTGAAATAGTACAAGTATCAGTTGGAACTCTTCTTATCAGTTGTTTGAAGATTTAGATTGCC

>ONT.15932.3 LG10 68586920-68602056

ATGGTCTGGTCCCTCTTATCTTTATGGAGTGCCTCTCTGTCATTCTACTTCCCTCTGGTTCTTCCTTAACAGTAGTCAACACAAGTCTTGCTCAACTTTCCCAATTGCTCTAGCTCTGCTTCTATCTGTTTCCCAAACTTCAGATCCCATACAAAAGATCTCTCACACAATGGTGCAAAAACAGCTTACACCAGCAAGCTCATACCCCATCTTTGCCATCATCTAATCAGGTATCTGAGTTCTGAGGATCCAAACAAGGCCCTTGATCTCCTATTTGTCATGACAGTTTGGTGACATTTAAACATCTTTCCAAGATTGTCATGTCAACTTGTGAGCGCTGTGTTCTTAAGATAGGCTATGCAATGAGTTTGTGTGATGGTGGAAAATCAGTATCATTAATATATCAATGTAAGGCTAAGTTTGGAGACAATCTATTCTGCTACTTGTGAAATCATGTGTGCATAATAGAACTAATTGTGGATTCTGTAAACTGTGGATTCTATAATTTGAAGTTTGAGAGTGCATAAATCCATGGAAGGGGAAAAAATCATATATTTAATCCTT

>ONT.477.1 Contig01353 37808-51427

GGAGTTTGGACAGTGGACTGTAAAAAAGAACTTCCCCACCTTCTCTCTCTTACATGCACGCAGAACCTCGAAATCTCTGTAGCAATGGCGGCATGCGCGGCTGCGGCTCCACCTTCTCTCTCTTTCATACACGCAGAACCTCGAAATCTCTGTAGCAATGGCGGCATGCGCTGCCGCGGCTCGTCAAGCCGCGTCTCTATCTCGGCTCTCATCTCCCAAATCGGCTGCTCAAGCCTCAAATCTAGTGCAAAGACGCCGTCTTGCGGGAGGTGGAGATCATCATGGATCCCTCAAGGTTCACTGCTGGCAGGACCCATTAACTCCATCTAGATGGAAGGAAGAGCATGTGTGCATCGTTTGGGGGAGCTTGGCAACGGGGAAAATCAGCCATTGGACTTCAATATGCGTGGCTGATAAGAGATCGTCATAATGGAACTGAAATAGATACTAGCATTACTTCTTTCAATAAATGTTGATTTTATGATTTTTGGCTGGCAGACTTGTTGATGTCTCTTTTCTAATTAATTTGATCCCTCAAATAAATTATTACGTTGCT

>ONT.424.1 Contig01206 74265-103067

CTTCTCCTCTCCTTCTATGGCTTGTTGAAGAACAACTCCATGGAGGCTTCCATTGCAAAATCCGATATGGCCAAGCAACTCCCCATCATCTTCCCCGTGACATAGTTTGTTTTGGTGCGAATCCACAGTGGCAGTCCCCTTCTTCCATCACCTTCTCCTTTCTTTGACCTTCAAAGAGATTTTGGTGGGTTTGTTTGGGAGAGAAAGCTCTTGGTTTGGAGCTTGGATTGAACGTGGTTTAAGGTTGAATTAGCTCTTGATTAGAGGCAAGGGTTCAGGGAAACCAAGAGGCTTAGTGACGACGAGATCATTATCAACCTGGGATAGGATGCTAAGGCACAGGTGGTGTCTGTTGGAGTAGTTACTTTATGCTTTTCAAATAATAAGTTGATTTTGTCAGACATTTTATATGTACTGTCTTTAAGGCAGAACTCAATTTCAATTTCAAGTTGAGTGAATAAAATTTATTCTGTTGACATTGGTACT

>ONT.8406.1 LG05 34454441-34481314

GAGGATGACGACTTTAGTCATTTCCTCATCACAAGGAACTGAAATGGAAGCCACCAAATGGCCCCTTCTAAGAAACTGGTGGAATTTGGGGAGATCTACTGAAAGAAGAAGAAGAAGAGGCCTCTCCAAGACAACTGGCATTCTTCACCTTGGGCATAGGGATAGAGGCAGCTTCTTGAGTTCGAGCAACTAAAGCCTTTGACATATCAACATCTTGGAGTTCACCCTTTTCTTGCATATAGCTCCTGTGTGGAGGGCCACTGAAACCCAAGGAGGATCTCAGAATACGAATCCAACTCCGTCGTAAGGAGAAGGATATTGGCTCCAGGAGGAGAAGAAGAAGAACAATAAAGTTTCTATAGAGCTTTGATGTAGAGAGACATAGTAAGGTGCTCTTTTAAGTTGAGATGGCACTTCTCATCCAACAGACATCTATATATAATGAGTTGTTATTGCTAAAAATGGGAATGACTGAGAGGGGTGCATGAACCTATGGTGATAAGTTGGCATTGCTAAATATGAAAAGGATTTTAATTGGATTACTTGCATTTGGATTCTCTACAAAGTTTAGTATTGTAGGATAAGGATACAGATTTTCTGTTGCCTGTAAATTAACTATTCTTGCACATGAATCCAGTACAAAAGATTTTGTCCACCTAGGAGACACAGAGAAGGATTAGAGCCATTGATTTATTATTA

>ONT.7482.1 LG04 24334947-24347052

TTCCCAATTTTTTCCCCTCCCTCCCGCGCCCTGTCCTCCCGTGCACCAGCGACGACACTGCCGGAAGCCGCCTACCCCGGAGCTGCCACCGGTGCTCCCTCCCTCCCTCCCTCTCTCTCTCTCTCTCCCTCTACCTCTCACTCTGAACTGTGCCCTCCCGTCGCCGGTGCCGCCAATCCGTCCTCATCTGCGATCCCGACTGCCCTTTCTCCGTCACTGCTTTCGACGCCAATCACTGTCCAGGAAACCCCGTTTTTGCTCGCACCATTCAATCCTGCTACAGCTGCTGATGCAGGGGAAATGAATTTATATTAATGAAAGGAGCATTTGATTTCTTTCGCTTAATTGGCTGGTCTCCACTGTAGGTCATGTGGTCGACGTTTGTTCCCTTTATTCCCAACAGCATTTTGCCTGGGCTCCTCACATTCATTGCAGCAGTGGCTGCTGTTGTTATGGACACCAGACGCTGGCAGCGGCCACAGGGCTAGACACGACTTCGGTGGAGAGGGCAGTCATGATTTAACAGGCTCGAGTGATCCTGATCAGGTTTAAGAGCATCGGATATACCACGGCACAGGGCAATTTTTTTTTTCCGTTTTCCTGTAATTGTAATGGATATATTTTATTGAAACTTTTGTAATGTTTTGAATGCAATTAATATAAGACTTTCTTTTTTTATTTGCAATTTTAA

>ONT.249.2 Contig00712 5890-95867

GGAAAGGAAGGATTTTTTTTCTCTGTAGTTTGGAGTTTGGACAGTGGACAAAAATAACTTCCCCACCTTCTCTCTCTTACATGCACGCAGAACCTCGAAATCTCTGTAGCAATGGCGGCATGCGCGGCCGCGGCTCGTCAAGCCGCGTCTCTATCTCGGCTCTCATCTCCCAAATCGGCTGCTCAAGCCTCAAATCTAGTGCAAAGACGCCGTCTTGCGGGAGGTGGAGATCATTATGGATCCCCCAAGGTTCACTGCTGGCAGGACCCATTAACTCCATCTAGATGGAAGGAAGAGCATTTTGTGATTGTTTCTTTATCTGGTTGGGGGTTACTTTTCTATGGAGGCTACAAATTCTTCTCTGGTGGCAAGAAAGACAAAATTTCATAAAATCACAGGAGCACTCCACTAGGCTTGATGGTGCAGTGTGACAAGTTTCGTTGTTGAGTCACATTTGAAACTAAATTATTTTCCATAACTTTGCAATATGTAATGAACGTATTTTGGTTATGAGATGTCGATTTTGAGTGACTAGTTTACATAACCCCTCGTTTATGGTTGAAAAATGCTGAGACCATGTTGCATGTCCCGGTCATGTGGGCAGCCATTGTTGATCCTTTCTTATTTCTGAATGGTTGCAGTGTTCTACCTTCTTTTATGGTGTATTTGGATGCTAGGATTT

>ONT.10662.3 LG06 33950157-33960468

GGAACTGTAGGCGGAGGGGTTTCAGTCGAGAGGGAGAGAAATGAGGAAGAAGGAGGCTTGGGGTCTTATATACTGAGGGTCTCCCTACTCATACCCTTCTTCCCACTACATCATTCTGTTTGTTAGAGGGTTTTCTCGGTCACGCTCACCCTTCTTCTCAGACCCCGGATATAAGTTGGAAAGGTATACAGCTAATGACTCACGTATTATGGTAGATGTTGTATATAATCCAAAGTGGAACTTTCCTCATGCTCTGCCAGGTAAATATTATGTAATTGACACAGGGTATCCTAACACACAAGGTTTCCTTTGACCTTATAACCACCGTCGCTCTCATATTCAAGACTTCCGCCATGATGCTGGATCTAGAGATAATGCGGAGCTCTTTAATTTCTATCATTCCTCACTAAAGAATGTAATAGAAGTATAGAACGTTGCTTCAGGAAGCTAAAGGCGATCTTCCCAATATTGAAGGAAATGACTCCTTGCCCATTTCCTACTCAAATGTATATAATCGTCACTACAATGACAATGTATAACTTCATCCACCAGTAAGCGATAATTGATACCTTGTTCAATTATTCATATTATATATGAAGCGAACTGGATCCAATTGAGAAATTGAACAATTGGATCCAATTGATTTTGACCATTTT

>ONT.15025.1 LG10 70458969-70461588

GGTGTTGCATGACTCCCAGTCCAAGCTGAGCCTCATTCTTCTCCTTCCTTGCTTCTCTTCTCCACTGATCAACCATGATTCGTCACCGTTTGAAAATCCGATTTCGCCATTGTGTTCCCCACCTCTTTAGCTCCCTATAGATGCAATGTTTATGCAAAACAAAGCTGGGGCAGCCCTCCATTTGAACTCTTTCTTGCTGGTTTCAGGTCCTTCAGGTGTGCTTATGTCTAATTGGTGAGATTATGGGTAATCTTGGAGTGCTAGAGGCCAAGGCAAATGGCAGATTCTTCGTCTTGAGCTAGTACACGTGTAGATGGCTAAGGAGCCGCTGGTTGCAGTTTTTTTGTGTCATTGATGTCATGTAGACGTTGTGCTTGACACCGGAGTTACGTTTCACCTTTGGGTAGTATTTGTTCTTAGCTGGTACTTTTAGTATGGCTTTTGTTAGCCTTTTTTTCTTTGGGATGTTAACTAATGCACTTTGTATAATTTGTAACTTGAACATGTTTGGGTAATGATTAGCCTATTACTGTCAGCTCCGT

>ONT.2325.1 LG01 40501670-40503938

TGCTTCTTTCTTTTATCTCTTTTTCTCTGTTACTCTCTCTCTCTCTCCGGGACTTCTTACTCTTTCACTGTCTCTCCCTTGCTGCCTACTCAACTGAAGCCCCACCAAGCTAAGAGGATCACCGTCATTGTCCTCCCCATGCTCCAATGACCTTGAGCATCCCTTTCTTCAAGCCATCCATTAATTCTCCATCTCTAAGTTGTTTTGGGTTTGAAAGGTTCTTCCCCAATTCGAATTCAAATAGTGGGTTTTCTACTTTTCCCCTATTTTGGAGTGTACTCACCAAGGCAAGTACAAAGCCCTTCTACCTCGAGAAGGATATGACGAAAGAGATTCAGGCGGATCAATCTGATAACCTCCTAGATTATACTACGAACGACATTGTATAGTCGCATCAACCCCGTACTCGAGAGCCTTCGCGTCATATTGGATAGATTCATCTTTTGATTAGGCCTCACTTTTAGTGTGGCATTTTTATGTAATTATTACTTTTGGTTAACTATTTCAACTTGGACTTTAGTTAATATATTTCCGTAATTGCTCTCAAA

>ONT.14891.3 LG10 36987007-36992386

AAGAGAAAAACTTCTTTTTTTTTCTTTCTTTTTTTCCTTTTCCTCTCCTTCCATGGCTTGTTGAAGAACAACTTCTTGGAGACTCTCCTTGCAAGATCCAGCTGGGCCAATCAACTCCTCATCATATTCTGAGCCACATAGTTTGTTTTGGTGTGAATCTAGCAGTGACAACCCCCTCTTCCATCACCTTCTCCTTTCTTTGATCGTCTAAGAGGTAACGAGCTCACATACGTGCCCGATTGGACTTTGCGGCCATCCAACAGGATCTGCCAGTTACACAAGAGTGAGCAGCCGACAACATAGAGTAGTGTCCAAAGCTGCAGAACTCGTAACATTATCTTTTGTGTTTTTTGTTATTTTTAGGGAATTCATCTTTTGTACTTGTAATCAATGGCCATTGTTTTTGTACGGCTTTATTCTGAGATGTAACTAAATTTAATCTTTTGTTACACCTGAATGCTAATTACGAAATGAAATCCTTTTTGCTA

>ONT.641.3 Contig01843 19680-38694

AAGCCAGCTTCGAACTTCTAACTGCTCAATTGCAACTGCAAGACCACTTGTAAAATATGGATTCCAAGAAGGCGCAATATCCAGATTGGTGGGGGAGGGAACAACCCCCAGGTCCAAGGGAATATTAGTGCAGGTACTGGAGGTAACATCAATTTAGGAGTTCAAAGAGCAAATGTCAATTCGTGAAATCAAGTAAGAGGTGGTGGTAACATCAATACGGGAAATCAAGGATATGGTGGAGCAAACGTCAATTCGGGAAATCAAGTAAGTGGTGGTGGTAACATCAATACGGGAAGTCAAGGATACGGTGGAGGTAATGCCAATACGAGAACTCAAGAATAAGGTGGTGAGAGCTGGACCCACTGAAAATTAATTTCGCCAGTTATCTATGTTTCATGTGTCTTATATAATGTTTAAGCTTTGTCTACTATCAACTATGTCCGTTTACTACGTGTGATGCAGTACAACATCATTTCTGCATTTTGTGGATTCCAAGTGGCTATTGTGTAATGGTTGCTTGCTTATTAATATAAGTTTGTGTTATATATAT

>ONT.10128.2 LG06 27624870-27627973

GAGAGAAGTCTCTTCTCCCCTACATTACCATCGTCCAGCAACAAAGAGCTCTGTCTTCCCCTTTCTCATTTCTTCTCCTTTTCCATGGTGGAGATCATCTACTAGTGGACCCTGCCTGCAAGATCCGACCCCGCCAAGTGATTCTACATCCTCTCCTTCACAAGATACATCGTTTTCACGAGGTTCCGTGACCGGCCATCCCATGATCCCTCTTCTTCTCCTTCTTTGAGCTTCTTAGAGGGGCTTTTTGGAGGTGGATTGTGAAAGGTTTGAGGAGGCGTTCCAGCTGAGATTTGGACCATTCGTTGAGTGCTTGGACTGCTTTGGAGCGATCTTAGGCCAAGGCATTCATGTCTGATCTACGCCTGGTGCCATAGGTATGATTTCCTGACATGTACCCTAGTACGGTTTTTGAGGAGGCGGTTGCTGAGCCACAAGTGACATGTGATCAAGATGCAGAGGTTGTCATTTAGACTCCACCCCTTACACATGAGACGCGAAATACCTAGGAGTATTATTTTGGGTTTTGTATTATGTTTTCCTGGCCTTTGTTGGCCTTTTATTTTGGAGAATGCAATTATTACTTCTTTTGTAATTTCTAACTAGAAACATGGGAATGATTTATCCTGTTTACTTGTCTTGCTCTATT

>ONT.14491.1 LG10 13140902-13147089

GCTCCTACAATGATTTGGGTAAAGACCATAACTTTCCAAACCTAGAAACCCTAAATTAAACACAGAGCAAAATCGTCTCCATCGGCGTTGTCGTTGATTTTAACAGAGAAATGGTTGGCAATGTAGAAGGTAGCTGAACCTGCTTACGCGTCAACAGCTCAAGCAAAATTCAAGTTTTATTTAAAGCAAACAAGTTCTTTATAGGTTTTACACATTGACGCATACCAGGGTATGGCATCTATAGCATTGGCTAGATTCCCCACCACCGAAAAACGTGTCTAGACTTGACAATTGATTGACTGTGTGTATCAATATGATTCGCTATAATGTAGTTAGTGTCTTTGCTTTAGAGAGCAAGCGCAGTGTGTCACAATTTCTGCCACTGTTGAGAAAATCTGAAGCATCCAAACCATCTATCATAAACAGAGCAGGAGCGTGGACGCACCTAAAGGCTGTCATACATGCATGGATAGTGAGACAGAGAATCTGCATTGTGGAACATAAAACCTGAAGCAAGCTCCATTATTAATCACCACAGATAACTCAGGCTTGAAGATTGGTCCACTGAAGAGAATACGGAAGGTGGTCCATATTTAGCTATCAATAGTCCTGAGAGGCTTGTTTAGTATATAGAATTATAGATGACTTTTGTTTCATTGTCTACCCATCGTCTGTCTCATTAAATGTTCTTGACTGAGATTTGTCCAAGTTACTATATGGAAATCTTAGCCCAAATGC

>ONT.493.1 Contig01453 24142-34392

ATCTTTAATTTCAAGATTGAGATGCTTTGCTTGACATGTTGCCCTCTATGCTCCAATGTAGCTCTTCCTCCCCCTTCCTACAGCCAAACTCATCATGTACTTGAGATGGCTCTTGCTCCTACTTCCTTCATCATGCACTTGAGATGGTTCAAACTGGGTCTTATATTGGGTTGCTATGGAGTAGAGACCACAATCAAAATTGTTTCAGGTGACGTTGCCTTTGAGGAGTCCGGATGAAGCTTTTGAGCCAACCCCAGTCAGAGAGTGGCAACTCGACGATGTCAACTAGACGTCGGGCGGGATCTTGAGTTTCGCTTCACTCAATCATGGGTTTCATTTTGTTATTTTGAGAATTGTATGGGCCTTTGTTGGCCTCTTATGGGTTGTAACAAAAGCTTAACCTACTTTTGTATTAGCCTTGAAACTTGAATTTAATGGGAATGTTATGATATGA

>ONT.6376.2 LG04 592916-597758

ACTCACTAGAGCAGAGCTCTCTCTTTCCCGGCATTCACGAGCGGGACAAGGGGGCTTGGAAGAGGACGAATAACTCCAACATCTTCTTCCATGCATCGATCGTCTGCATCCTCCACCGCACTACATCCATTGGTGCCTGCCAGAAAAACCCCAAATCCCAAAAAGCCCACTCTCTCTTCCCTTTCTCTTCTTTCTTTGTCCACGCCTAACACGCCCTTCGTTCCCCGGGTATGGCTAAAAAGAGGAAGGATTGACTACTCTGATTGAAAGCCGGTTGTAGGACTTCTTCTCACTGTGGAACCCACCTGGATTCATCCAAAACCAAATCTATCTCTTCTTCAAGATATTTTTGTTCCAACTTACAAGCTTCTAAAGATTGATTTTGGATATTATTGTGCTGGCTGCGGGCTCTTAGGGATAACTTTTTTTATTTTGATCACAGACATTTGTATATGTTAAAGATTTTTGGAAATTATCAAGTAGTAATATTTGTCTATTTTAGAGATTTGTGGGTTTATTTTGGTCTATGGATGTAGGATATTTGGTTGTGAATGTTATTTTCAATTTGTTAATGAATCTATTTTAAATTGGGTTGTGCATCAA

>ONT.7784.15 LG04 86662021-86720645

AACTTCTCAATTGCAACTTCAAGACCACTTGTAAAATATGGATTTCAACAAGAACAATAAGCTGACTAGTGGGCAATCGCACAAAGAGGTGCAACGGGGTATCAATGCAAACAAGAAGATTGGTGTGGAAGGGAACAACTCCCAGGTCCAAGGGAATATCAATGCAGATACTGGAGGTAGCATCACTTTGGGAGTTCAAGGAGCAAACGTCAATCCGGGAAATCAATTAAGTGGTGGTGGTAACATCAATACCTAAAATCAAGAATAATGGTGGAGGTAACGCCAACATTAGAACTCAAGGATAGGGGTGGTGGCTGGACCTGCTGAAAATAAATGTCGCTAGTTATCTATGTTTCATGTGTCTTTTGTTATGTTTAAGCTTTGTCTACTGTCAACTATGTCCGTTTAATTACTACGTGTGATGCAGAACAACATCATTTCTGCATTTTGTGGATTCTGTAATAGTTGCTTACTTATTAATAAAAGTTTGTGTTATATA

>ONT.3594.1 LG02 97768140-97769448

AGGATCCCCTGTTTGGTCCGAACAGGAGTCTCCACATGGGGGCAGCCCCCCCCCCCCCCCCCCCCCCCCCCGAAAGCTCCCGAAAGCTCTCTTTGCCTCCCATCTTCATCGACCACCCGAAAGGAAGTTCAAGCATCATCCCCGTCTTCCTGAGATTAGGTTAGGATTCACTACATAGGACAGAGTGGTGGTAGTTTGTTTTGAAGAAAAGGGTTTGAAGCATGTTAAGAAAGATGGGATCACAGTGGGATAGGACGTTGAGATGAATAGAAATTTTATAGTTGTAATTGATCTGTAGCATGTTCCGAAACAAGGAATGAAAGTGGGGTTTCAATGCATCTTTGGAAGTGAAATATGAGATTTTGATCTATAGAGATGGGTGTTTGTGTTTGTAGAAATTGGTGGTTTGACACTGTAGAAAAAAGTTTGTTTGTATTTGTAAAAATATGTGATTTGTATTTGAAGAAATAGATGATTTGAGTGTTGTAATGG

>ONT.435.1 Contig01232 84228-96285

GCTCTAGGTATTATAGAGCTCCTGAGCTTATCCCCTTTCCCACCATCCCTATTCGGTCCAACCCATCTCTGCCTTTTCCAAAACCTCTCCAGCCCCACCATCCCATATTCAGTCCAACCATTATCTGACTTTTCCAAAACCTCTCCAACGGTCCAAGTGTCCAACCATTCTCTGCCGTGACTCGTGAGTCAATCGCCCATCCATTGAAGTTGTTGCTGTTGATATATCCGTCGCCGATCGCACGGATTGTTGCGTGGAACAGTGAACACACCGACTAGGCGACTAAGACTCAGGGCCTTGGAGCAGGTTACGCTCAAAACTATAAGGAGAAGGAAAGTTGGCGCACAGTACTAGGGCCTTACCTGAAATAAATATCATCACCAATGGTGAAGAAGGGAGATCACATTTTGTTTTTTAATGCAGTTTGTGTTATATGGAGCTTGTATAAGTTGCTACGAATTGCTGACTTCGATCTGTTTCTTGTAAGCTTAATACTCTGAAGCTCGTTGCAGTCAATTGACCTGTGAGTCTGGTATTTACTAATTTTAAGGGATGATGATGATATAATCCATCTGACAA

>ONT.4498.1 LG02 29621468-29628588

ACAATAGCATCCACAAAAGTTTCGGAGGATATCCGGTTGGGAAACTTCCATTGTTGCTCGGAGGAGCTTTAATCCAATCACCTCTGCAAATGCACCCCATCAACATTAATCCTCTGTAAGAGTTCTTCTGACATGCCGATTAGGAGGAGTTCTTGAAGAGTAGTAAGGTACTGCATGCCCTCAGATTCAATTTAGGGATTGCAAAAATCGACAACACCTTGAGTTTCAAGAAGCATCCATGACGGAAGCGCAATTCCTCCCCAACATATGCCGTTATAAGATAAAGGGACATGAGATTGGGCAATGCTTCAAGTGAAGACAATGGATCTTCTTTCAAATGAGACCATAGTAACCCCAAGTGAGCGAGGTTCTGAAGGGAACCAATCCATGGAGGTAGCCTCTCCAAACGCCCATCTAACCAGAGATTTGTGAGATGGGTTGGTGGAGAAGTTACTGCATCCAAATCAAGAATTTCTTCCTTGCTTATGACAGCTACTTCCAAACTGAAAAGGGCTTCCATCTTCTGTAGGGAGGCAGAGAGTTCTTTTCCATGATTTCTTTTCACATTTTCAATTTCCAATTTTCTTAGTTGAGTCAAGTTTCCCACTTTTCTAACTGTATCATCGTTTATACCAATAATTGATAAACTCTGTAAGCAATTCAAATTGAATATTCCATCTGGAGCTTGTACGCCTGATGATGTAAACATGTGCCTCAATTTCTTCAACTTTC

>ONT.1635.33 LG01 118121171-118122171

TCTGGTCTCAAACCTCTCAAAATGGTGAGTCATAGATCTTTTACCATTGTCTTCCTGGTGTTGTTGGGTGCAGGCATATGTTCTGCAGCTAGAACGCTCCTTGCCTTTGAAGGAGCCCATGGTGCGGGCCACTTCGTCGACAGTGCCGCATCTGGAGGCTACGGTGAGGGTGGAGGAAGTGGCAGTGGAGCCGGGTATGGAGCTGGAGCTGGTGGTGGCCATGGCGGCTACACTCCATGAGAAGATAAGCATGTTTAAGAAGAAGGTGGTACTTCTAAGCAGTAGTATTAATAAGAAGTGTGATTATGATAAGATGGTCAGTACGTAAGTGTAAAAGTTTCAAGCGCAGTGTGATAAAAGTATCTCAGTGCTGCATTGTAATATCTATGTGATTGTATGAAATGAAAGATCTCTGTAGTGCTTGCTTGATTTCTGCT

>ONT.11157.2 LG07 5637522-5638880

ATCCTCCTAGGCGAGACTCTATCCGGCGATCGTTTTGCTCTAGCGAGAACGTCTCTCTCTTCGGTGACGCTCCTTCTCCTGCCCTGGGCTTTTCTCTCCAGCGACTGTCTCTTTGAGGTTGTGACGAGGTTGGAGAAAGATTGGGTAGGATGGGATTGATTGGAGTCTAAGTTTGAGATGGGTTCAAGGGGATGGCTTGGATACAATTTTCCTGCAGATTTTCATTCTCTTTGTGCTCTTTAGTTTTTCTCCCTCTGCAACGGTAGAAATGAAAGCCTGGGTTTTATTGTTTTTAATTTCTTTAATGTGATCGATCTGAGTCTTTGAGTTAGAGATGGGTTCAAGGGGAGGGTTTGGATGGGTGTCCTGTCCTATCCCAATCTTCAAACACCAACTGCTATCATGTCCCCAAGTTATTGCTAAAAAGAGAAGATTTGAGATTAAGCCCCATTTTAGCATACATGTTTTGATTCAAAGGTGATAAAAACTTGTAATCCAAAGGTGAGGAACACTCCAGCTGGTTGGAATTCTAAGAAGATTGCATTTTGTTTCCATGTGAAAATGGTTGGGGAAGTAACTATATGAACCTCTATTATGGATATGAATGTATATTCTGGCAGTGAATATGTACATCCAAGGCCCAAAAATTGACATTTCAAACTGTTGAACCGAAATGCCCCACTATGAATAAGGATTAGGGCTGAGTTGATTGGTGAACGGTGAATCTGGTCGAAAGAGGGTTCGGTTGATGTGGGTTTATTAGTAGTTCTAGCACATAATTTTAGGTTAACAAGTGAAGGCTAGAACATTGGACCAAGCCGGTCACTTGATGAGAGCAACAGTTTGGAGTACTCTGTTTCAGATGTGTAATAATGGGAAGGGCGTTAGGTGATTTTCTATATATTAATCTAATGTATACCATGCACTATGGGTTGTTTATTTAACAATTTCTTTTATGTTTGTTTACTGTGCATAGGATTATGGGTTGTTGATTTAATGTTTTCTTTTTATGATTTCACAGGGATAAGTGTCGATGGAGAAGGCATGTTGAGAGTTGACATTCTGGATCATTTTGAGTTGTTCATGAGACCTGACATTTTGGATCATTTTTTAGTGGTTGAGAGTTGATTTTATGGTTGATTCTGAGTTGTTGTATTTGGCATGTAATTTGATTTTTTGTGTGTTTGGAATAGATTTTTTTTTGGATTTGATATGGTGTGTTTCAAATTTGTGGTTTGAAATTTTTTTGTAGAAATGAATGGATGACTCGTCATGTAAC

>ONT.51.2 Contig00149 61336-66508

AGCGTCCCTGGCATCTCTCTCTTTCTTCATCTTCAACTCTACTGCATTGCCTTCTTACAACCCTCTTTCTCTTTTGCATTAGTGCGTTTGATTTCCTCCACCAGCGTCGACGCGTTGCAGCCCGCCGTTGCTCGAGAAGATGGCCCCCTCGAGATCTGGCTCGTCTCTCCCGGCTCCGTCGAATGGGTATTCCATGTGTCCGCAAACTATCCAAGGCAATCCCGATTCTAGGGTTTTTTTCCCTCGTCGATCCTCATCCAGCATCGACGGTTCGATTTCAGAGTGGGATCTTTTCTCCTTGAAGCAAAAGGTTTGATGATGTTTTACTGCATACGAATCTGACAGCTCGGCGGCGTAGGGTGATTTAGATAATGATGCAGTTACAAAGGTTCAAATGAAAATTGAAGAAAGTTTTTTGTTGAAAGATTCATGCAACTAAGATGCAGCGAACATGAAGGAAAAATGGATGCAGACTAGCAGATTGCTATATGTTAAAATAGGATGTATGAAGTTCTTTTTTATCAATGTTGCAGGTGTTGGATTGGTGCAACAATGAGATGAAGATGTAAATTTTCATTGGCATTTGAATGTTGGCAAAGAGTAGAATCTTAGCATGGATTTTTATTTTGTAATGGTAATGGTAAAGTTCTTTTAACATTGTAAGGATATTTGAATATTAATATGAAGTGGAATGAATATTTGGAATTGAAA

>ONT.7556.5 LG04 61082119-61088097

GGATTGTTGATACTAATGAACCGAATTCTCAATCCCTCATCTCTCTCTGACCCTCTCTCTCACCTGCTATCCCCTGCGTCTCGATCCCCTTCTCTCCTCCATCTCGATCACCCTAATCCCATCCCAAAAACCCCGCATCTCCATCTTCTTCCTCCCTGCACAGACCTCCCCATTACACCATTTCTCTCTCACGCCTCACCCTCTCCACGCCTCCCCTGTGTATCAAGCATCTCCATCGCCCATCTCACTGCATCCCCTGCGCATCAAACATCTCCATCGCCCATCTCGGTTTCTCCTCTATCCCCATCACCTAAGGCCGATCCCTGCCATTCATCAGAAGATCACCACATCCCTCACCTCTGAGTCTCTCTCGTCTCCCTTTGCATCTCTCTCGGTGTGCCACCTCCTCTGTTATTTTCTCTCCGGCATCTTCAGAGACATAGAGAGAAGCGATGGCGACGAACCTGGTTACATTTCAGAAGATTTTCTATAAGAAGAGAGACAGAACGAAAAAGCGATCGTTACCCCTACGGTGTCATTGAAATCACGCCTCTGCCCAAAAACCTCGGCATTCGCTGCTTCCCCTCTTGAGATATGATATGCAGAGGGGAGTGAAATCTACAAAATGTCAACACCCAGCAATAAAGTTTGCATTTATAGTTGGGAAAGGAAAGTTTTGTCAAAGGGGTGCAGCTAGAATTGGTTAAGAACCATGCGGTTAAGCAAATTGCTGGTTTGGTAAAGGAGGGACTTCACAATTATATATACCCAGCAATAAATTTTGTTGATTAGGCCTTCACTTGGGCAAGTTCGTCACATCAAGATCCCTCATCGGAAGCCTGGCTAATTTTGTGGAAGTGTTGTCTCCATTAATCGTCTCAGTCTCAGACAGGCCACGCATGCGGATTCTATTTTCCAACGGCAACCAGATGAGATTCTCATTTAAAGTTGAGATCTTTTTTGAAGCAGAGAATTGGGTTTGTCGTTTGGTGGGGCTTAAGTAGATATAAGAGGTTTTGTTTGGGTTTTGGAGTTTTAGGAAGGCAACATATGAATGTTCTTCAAGCTTGATTTTTTTTTCTTCTCTTTTCTCAGCCATGTGAGGGGTTTACTTCAGGTGAGATTAGGTCCTGTAGTATGATTTAACTGCAGTTGTATTTATGTAATAAGATTTATCTTTTCTTGCTCTT

>ONT.15179.3 LG10 87054917-87085204

GGACCACTTCTTCTTCATCTTCTCCAATCACCCAAAGCCACCGGGACTACTCTCTATCTCTCTTCCTCGTCGTGAAATGGGTTGCACAAGAAAAAAAAAAAAAAAATAGAAAGCTCTTCCATGGTGTCTTAGCTCATCTCCATCTCCTTTCCCGTTCTCAACCATTCCATCTCAGTCTCTCTTTCTTAGTAATCCATCTCCATCTCTCTCTCATGAGCCCATCTTGGATGCACAAAGGTGATTGATATAACCTGCAACGGGGCTACTGCTGTGTCCAATATCGGTTTGTTGAGCAGAGATGAGGCGAAGTGGAAGTGGAGGTCCATCATATTTCTCTTGCCCTTGTTGCTATGAGAAATATGACATCACCACTCTTTTTCATGTTGGAGTAGTAGTGAGTGTTGAGAAATTAATTGTTTTTCTTCTTTGGCTCTTGAAAGAACCAATATTCTCTATCTGAATCTATCTGAATCTCTTATTTTGTTCTTTTAGAGAAGGAATTGTTACTGATATCTAAA

>ONT.7059.12 LG04 86525891-86716171

CCTCTTTGACCCTTTCTCGCCTGCCATCCCTTCCAGCAGCTCTTTCCACCGATTCCTCCAGCGGCTCTTTCCAATCCTCCTCCTCCATAATCACGCCTTCTTGAGCTGTTTTTGAGGATGACTCAACTCGGGCCAATTTTCAAAACACAGAGCTAACAGACAACAGAATTGAATAAAAATTGAAAAACAACTTCTGTTGAAAGAGTCATTGCTGCTAGGATGCAGCCAACATGAAGGCCAAGTATGTAGAGTAGAATGTTTGGCATTTGAATGTTGGTATAGAGTAGAATCCTTTTGTGGTGAATGACCACTTCTCTTACGCGGTGATTCTCCACTGTGTGCTAGAAGATAAATCTGATTGGGTTGACAAACCATTGTGTGCTAGATATATGCTTTGTGCTTGTGTATGTTGGCTGCCCACCACTTTAGAAATCCTGCTGTGACTTTTCTCTTATCATGCAACAGTATTGTTGAGTTGTAAACTGCATCATGGTTCTGGCCTAATTTTCAGTTTATTGACCGGTTGTT

>ONT.12838.3 LG08 7191860-7204728

ATCTCCCAGGCGACTTCATCCTGCACAGAACCACTCACCAGAGCCTTACCCTCTCCACGCCTGTCTCTCCATTACCGACCCATCTCACAGCCTCCCCTGCGCATTAAACATCTCGGACTCTCCTCTATCCCCATCACCTGAGGCCGATCCCTGCAACCCATCCCTCACCTCACCTCTGCGTCTCTCTCGTCTCCCTCCGCATCTCTCTCGGTGTGCGTCTCCCAGGTTATGAAGACGAGAGGTTGCTGCAAATGTTGCTGAAAGAAGCCATCCATGTGATCAGCTGTGTGGCTATGTGGCCAAGACAGAATGGGGCAAAGAGGCATTTTACAAAGTTATTGGAAGTTGGGCTTCGAAGAAGTTTATGACAGGATGATTGAATGAACATTGAAAAACAAATTTTATTGAAAGAGTCCATGCAGCTAGGATGCAGCCAACATGAAGGCTAAGTATGTAAAGTAACATTGATATATGTTAAAATGGGATGCCTGAAGTTTTTTTAAACAATGGTGCAGGTGTTGGAATGGTGCAACAATGAAATGATCATATATGAAGATGTAAATTTTCATTGGCATTTGAGATTTGGTACATAGTAGAATGTTAGCATTTGTTTTAATTTTGTAATGGTAATGGTAAAGTGCTTTCAATGTTGAAAGGATGTTTGAATATTATTATGAAGTGGAATGAATTTTCTTATTTC

>ONT.11163.2 LG07 5836775-5839582

GCTTATTACATCTTGAGAAAGGGAGAGAGTGAGAGAGATTTCTTCCTCCTTTTTCCTTTCCATTCCTCTTTCTTTTCCCAAATCATTTTGTGCATATGGTTGAGAGACAAGGATTTAGAGTCTTCTTCTTCTCCATCTTTATCTTTATCTTCATCTTCTCCATCTTCCTGTTAAGCCTTGTTGACAAGAACCATATCCTACAACTTTCATTTTCGGATTTCTTATTGAGGGTTTGTTATTTGGGGGTTTTGGGTTTCCTTGAGCCATAGCGAAGAGAGACCTTGGAAAAGCACCAATTGAGGGAAAATTCAAGAAACCAAAGAGAGTTACTTTTAACCATTCAAATTTGAGATTAATTGGTAAGCTTAACGATTAGCTATGACTGCCGGAGTACGAATCAGAAGAGTAACCTTGAGTTTGAGACTAGATAAATGTCCATTGACAGGTGTCAGTGAGTAATCCTCACATGTAAAATGTGGAGTTTTCTCATTCGTGAATTCGGATTTGGTTTGGAACAGGTGCCAGTTCACGGGAGTACGCGTGTCACACCTTGGGAACCACCCTAAACAAGGAATTCTCGGGTCTAGGATGTGAGAAGATATGAACATTCCTTGTTGGTCCACATCTACTTGAGTGTGGCTTTCTTGGGATCTAGCCACTCAAAATGAAGGCACTACCCTGTAGCGACTCTTCATTATAGATTTATAGGACATGTGGGCATTCCAATCAAATCTTGAAAGAGGTGTGGGCCCCATCAATAGATGGGAATACTAAATTAAGGGCATCCACCTTGACCACCATATGTGGGAGGGGAAAATCTCCATTGGTGAATTATATGGGATGTCTCAAGTTCTACCATTTTAAAGCTGATCCCACTGTAAGATTGAATGTATGATTAATACAATTTATCAATATGAATACTAAGGGTGTCATTTCATTTCA

>ONT.11844.1 LG07 15344132-15345689

CTCCTCAACTGCAGTTGAGGAGAGCCTCCGCTGGAAGCGTGCGACGGTAACGTGTGGAGTTATAATGGAATGGGTCAAACCGCCCAAAAATTCAGTTCCCTCCTTCCAGACCGTCATTCATCTCCAGACCTAGAAACAGATTTCCTCACATGGCCTCTTCTCCCTGCTCTCTCCCTCCCATCGCTTCTCTCTCCCATCTCTGAAGCGTGCGATCTCTCTCTCCCTCTCGCCTGCGATCAGACATCGTTTCTCATCCCTCCCATCAAGCTCCACTCTGCGCTGCGATCTCTCCAGTTATTCCGCTGCGATCTCTCCAGCTGCGATCTCTCTCACCCATCTCGCCAGTGTTTGATCTCTCTCTCAAGAGGGCTCTCTCCCTCAAGACTTCCTCCCTGCAAAGCCCCTGCTTTAGAAATCTGAAAACACTACATGCAAAAGTGAAAACCCAAAGAGGGCTCTCTCCCTCAAGACTTTTCCCTGTAAAGCCCCTACAACTCTCCCACCTTTTTCATTTACCGCCCAAGCCGAAAGAGAAAACCAGTTTGAAGAAGAACAAGAAGAAAATATCTGGTCTCCTCTGCGACTGGCCTCACCGGCTGCATATCATCATCTCTCGCCATAACTCCACTCCTGCTCGTTTCTCATATCATGCTCATCTTTCTACATTTTCTTCTGGAGAAAACAGAGAAATAGAGAGAAATGAAGGAAAAGGAAGAGGTGGGGAGAATCGTCCACAGCCCTGCATATAACTGGAATTATGCTCTCAGATATCTCATCGGCAGTCATCAGTACGCAAAGAAGAACATGGGTTCTTCTGTACATCTAACGAGAATTTCAACATCTGTTCATTCATCCTCTTCTTAGAATCCGAAGAATCGTCGAAGAAGGTGATGCTCATCAGCTGTTGGGGAGCAAACTGACTACTATACAAAGAGATGGAATCCTTCTAATAGAAAGTCTCTCTTGAATTGATATTTTCAATTTGTATAATTGTAATCTTTTTATCAGATGCAAATGTAAGACTTCTGTTTGAATGTCATTCCATTATATTTGAATGGGCATTCACCAGTTTGTAAACCTCTTATGGTATATGAATTCTTAATGTATAAATTAGCATATGAGGATCATCACCAATTA

>ONT.8871.3 LG05 107669624-107707210

ACGAAACCCTAAACCCTAAGCCCTCTTACTCTCTTCGTTATCGCCTCCTCTCGTTCTGGTCTGCGCCTCTGCCTCTCTGCGAACTGCTCTCTTCTCCCACTCAGCTCTCCGTGATCTTCATCTCTCGCTTAGCTCTCTGCTCTCTACTCTCTGCTCTGCTCCCGCCGTCGCTGCGAGCCCTCGCACAGTCGCACGCAGCACGTTGACGCCGCCACTCTCGCACCTCGCACCGACGCACGCACGCCGCACGCCGCACGGTGACGGCACGCCGCACGGTGACGCTGCCCTTCTACACTCTCTCTCCCTACCTCGCCTCGCCCAGTCTCCCTCTCTCACCCTCTTTCTGTTAATCTGCTGGGCCCAAGGTGATTCGATGAAGCAGGTGAGATCTGCTCTCTTTTACATGCATTCCGTTTTTTATTTATAATATTGTAAGTAATTTATAAGATACAGTTGGGTGGATTTTGGATGCTATGGAATAAAATGGAAGCTCATATCCAAGTTTAATCTTCCATTTAATCATATCAAGATGTAATCACTTAGGTCGCACCAGACTTCTGACTATAATTGCAAAGAGGATCCGACTATAAGAAGTGTAATGCCAATCAAGATATGAAATTCAAAACCCAAGTTCTGATTATACTAAATTGCAAC

>ONT.7784.10 LG04 86543101-86720143

GAGAACAACCCCCAGATCCAAGGGGGTATCATGGCTCCCCTAGTCAAAAAGGACAATACTACAACGGGAAGGAGCTTCTTCGACGTCCAAGCGGATATTAGTGCAAATAATGGAGGTAACAGTAATTTGGGACCTCAACCAAAAAACGTCGGGAAAAAAAAGTAAGTGTTGGGGGTAGCATCAATACGAGAAATCAAGGATACGGTGGAGGTAATGCCAATACGGGAACTCAAGACTAGGGTGGTGGTAATTGAACTAGCTGAAAATAAATGTCGCCAGTTATTTATGTTTCATATGTCTTATGTAATGTTTAAGCTTTGTCTAATATCAACTATGTCCGTTTACTACGTGTGATGCAAAACAACATTTCTGCATTTTGTGGATTCCAAGTGGCTATTGTGTAATGGTTGCTTGCTTATTAATATAAGTTTGTGTTACA

>ONT.15632.1 LG10 22429474-22430530

CCCCCATACTACTACAAGTCCCCACCTCCTCCATACAACTACAAATCACCACCTCCACCCTCACCATCACCACCTCCTCCATACTACTACAAATCACCACCTCCGCCTTCACCATCTCCACCACCTCCATACTTCTACAAGTCCCCACCTCCACCATCACGATCCCCATCCCCACCTCCTCCATACTACTACAAATCCCCAACTCCACCATCACCATCACCACTTCCTCCATACTACTACAAATCTCCTCCACCCCCATCACCTTCACCTCCTCCTCCTTACTATTACACTTCTCCCCCACCTCCCAAAGGCCACTAATAGAAATGCCATCAGTTCCATTTCATTGGTGAAGGTCTTGAGAATAAGGATGATGGAGTGATTACATTAGCTACGTACAGTCTACAGTGGGATGCATTTGAGAATAAAAGAATTGAAAGAGGCTATTCTTAGCAATTCATTCTTATGTAATATACACTGTAATATGGGTTGTGTTGGGTCATATGAAATGATTGTTTGACATTTTGTTTCTCACGTCGTTTGTTCAATCAATGAAATTAATTTCAATAAACTCTATGCAGCGCATTTTCCT

>ONT.5831.5 LG03 3776347-3780635

GCATTGACCCCATTTATATCTGGGGAGAGAGAAAGGAAAGTCGGCAAAAAGATGGAGAAGAATGTGATGGAGAAGAAGAGGAGAAAGACCAGGGAGAGAAGAAGAATAAAGAAGCATCCCCAGCTGGCAGGCATTGGTATTCTCTCTCCCAGAAAAGAGTGTTCAAATACACTCCCAGGAAAAAGTTTCAATTTTTGGAATGCTTTTTGGTGGGATTCAGATGGATTGATTGATTTTCTCCTCTTTGATTTTCGATTGAATAATATTGAAGCATTGATGGCCAACTGAGCCTTGCATCCATCTCGTGAAGGAACTCATTGCTGTAGGGAGAACTGTAAGGAATCGCAACTGAATCAGAAAGAGAGTTTTCAGAAGTAGGCAAAGTTTGTTGCATAATACGGTCTACACCAAATTGGGTTTGAGACAAATTGCTGTTCCAAAGATTTGATTCAGGACAATTTTGATTTACTGATGCATCATAAGGCCTAGGAATCGGTAGTGAAGGATCTTCAACAACATTGTTGTAATCAGATCGGGTTTGAAGCGGATTTTTCGGAGTGCTGGTCGAAGGACTCGAGTTACCGAATTTTGAGTTGGTTTCATCTGGTTGAGATCCCCGCTTCCTTTCGATAGCCCTGTTTGTTAGGGATCGGATGGATACATCTGTATGTCCTGGAAAAACACTTTCTTTATTGTATATCTTACACAGGACGTAAGGATCACCCTGCAATGCAAAACGGAAGAATTTCATCAGTTCATATATGAACAAGATATTCATATTT

>ONT.11321.1 LG07 44439623-44459484

AACTGCGTGTGTTTGAGAGAGAGTGAAGGCCTTTGAAGAAGAGAGTTAGCTGCACTTGTTGTAACTCTGTGTGTGTGTTTGAGAGAGAGAGATGGGTATTTGGGATTTGTTGTGCTCTGCCACGGACATGCTCAAGCAAAACGCTCCCGATCCGGCCACAGTTGCAAATGCCTGGCAAAATAACTTTCCCGGTCCTCAAACTCAGGAGAAGATCAAAAGGATCGCCACAGAAGTAGCCGTCCCTGAGTTGCTTAGATTCACAGGCATCCCAGGTCTGGTGAAATGCTATAAGATTTTATTCGAAGATCGTTCATGACGGCAAGAAATTTGAACATCAGGCAAAAGAGTTGAAAAGAAATGCAAACGAAAAATGAGGACTTGTGGGAAGAAGTTGACAAAATCAAACGCAATAATAAGATCCTAAGAGAACATATTCTATGATATCGTATTAAGGTGTGATGGTGTGATGGACAAAAATTGTTTCCAACTAAATGTTATTTTCTTTGCTGTGGTTTTTTTCTTTTT

>ONT.317.1 Contig00931 109891-119566

ATCAAATTTCAGGCAGAGAAGTAATGGCCCTAATGCAACCCAGGCAGAAAGTTAGCTCTTCCTTTCCTCTTACCCAACACAAAACGCACGCAAGTAATGTCACGATCGTTTTTTCTAACGTGAGATTATCTCACGCGTTTCTCCTCATCATAGATCTCATTCGTTGGTTGCATTTTTGTAACGTCCGCTTGCAAAGGCTCCTCATTATTTTTGTTTTAACGATCAGAAGTTTCTCCCTGCGATTCCGGGGAATCATGGAGAGCTCCACTGCCAAGTTGCAAACCTCCTCTCCCCTCTTTTATATTTCTCTCACTTTAAGGTCTTTTTCTTCTGTAAACATTTGTAAAGTCCAATTGCAAAGGCTTGACTACACTTTGGTTTTTGCTGCAACATTTATCCAATCACCAGGATAGAGGTACCGAGCCTTTCTACCCCGAGGAGGAGGAGGCGGCGGAAGAAGTTCAAGCAGATCAGACTGATGACTTCCTAGAGTATAATACGCACGGCATTGTATAGCCGTGACGACCTCGTACAGGAGAGCCTTCACTATTCATCATGCTAGCTTCATCTTTTGATTTATTATACTTTTGTTTAGGCCTAGTTTTAGTATGGCCTTCTTCTTTGTAATTAATATTTTGGTAACAGTCTCAAACTCTAACTCTAATTATATTATTCAGTATTTTGCTCTTAA

>ONT.12805.2 LG08 3431786-3446805

GAGAGGCAGAAGCACAGAGGTGTTGGTCTTCGTGCACCTACCACTCAAAATAATGCGAGGTGCCTGGGAAACTATCAACGGAGCTTGAGCTCTACTACAGTCAGGCGCTTTTTACTTTCACAAGTAATTGGGCTTTTTTCTGCCCAAACAGGGTACCCTACCCATGTGAGCAACTGAAAAGATTCATTCAAATTTTTACTTCAATCATAAGCAGAGGTTAAAATAAGTCTTGCAAACCTTTTTCAATTACTTCCTTCTAGCTCTAGCTTGACTGGCTCTCATATACACCACAACTCATATCCGATTCATACTATCTCTCACAAACGGTTTGGGAAAACAGCAATCCGAATCAAAGTTCTGAGTATCTCCTCTTTGCTAAATTCTGATTAGGTGAATTGTGGCATTTGAAGTTCAATAAAGTATTAAAGGTGTGGATTGCTGGAGTTTGATAAAGAGTTATAGGGATATTGGCAACTTCTTAATGGTGAAGTTTAGGCTGTGTCATTTCAAGTTTAATAAAGCATTACAGGTGTGAATAGTTGGAGTCAGATAAAGAATAGTAGGGATCTTTGCACCTTCTTATTGGTCTGGGCAATTCATGGAGTTTAAAAACAAACAAAACATTCCTTCATGATGTTGTTTGAACTCATGGTTGTGTTTTTTGAGTCGCTTTATTTGGCTTTGGTTTAACTATGAATTTACTGGTGTAGTTGTACTGTGTGGATCTTGCTTGTCATCTTTGGTTTGTTCTTATTCACTTGTTTGGAACTAAAATGATTCTCTATTTTTACCTTCTGTATGTGAGGAAATTATATAGACCATGGATGTAATCCTAATGACTTGATTATCTTCAAGTGGGGCCCATGATTTTGTCATGGAGAGTGCTGATGAGATGAAATATTTGTGGAAAATTCATATCCTAAGAAA

>ONT.386.1 Contig01130 241976-252612

ACCATAGAACTTTGTTGAGGCTTTTTCTAAATTGCTTCTTGCTCTGCTCGCTTCAAATTGCGAAGCGAGAAAACCTTTTCTCTGCAGAAATCGAACTCTACTCGCTTCAAATTGCGAAGAAGGAAAACCTTTTCTCTACACAAATCACAAGGGGGAGAAGCTAAAATTAGTCCCTCCAGTCCCTGCAGGCTAGCCTCCTGAGCAGCTTCATTCTCTCTTGGAGTCTCTTCAAAGGCAGAGGTCAGCACAAGTCTTGCAAACCTTTTACAATTACTTCCTTCTAGCTCTAGCGTGACTGTATCTCATATATGCCATAACTCATATCCGATTCATAATATCTCTCACAAGCGGTTTGGGAAAACAGCAATCCGGAGTAAAGTTCTGCGTACCTCCTCTTTGCTAAATTCTGATAAGCTAATTCTCACTCTTCTTACCAAACTGAGTGGGAATTTTTGATCCGACCCATCTTTTGGGAGAGTGGATTGACCTTGGGCACAAGAAAGGGAAGAGGGGCAGCGGCTACGTTGGGGAAGAAGGAGAAGGGGTTGAGCAAGCCCTACGGTTTAAAAAGGGTTCTTCGTCTCTCGCTTGACCGGATCATAAACTCAGTTGATCTAGTTTAGGTTCCGTCTTGGGTGTGAAGACATGCCCTGAGTACTCGCTTGAACAAAAAAATTGCTTGCTGTAACGAATTCTCATTCTGCCTGGGGTGAAATGATGTACTGGCAGACCACATTTTGGGCATTTTGATAATCACAAAAAACGATATTC

>ONT.12458.2 LG08 47469386-47476661

GCTCACTCCCTGCTCATGCATCCCTCTCTTCCTCAGCGATTTTCCTTATCGAGTAAATACTCCTCGCTGGATATAAAGCAACAAAGATCGCCCCCCCCCCCCCACACACACAAAAAAATCTATCATCTAGGGTTTTCTCTCGAGCTCTTCATCTTTCGTTTCAGGGAAGGAGGAAATGTTACCCATTCCTGAAGGTATTCAATCAAATGATGATTTATTACCAAAGGATATATAGGCAATTCCATTCAGACCGCTCTCTCAAAGACGACTGACATTTCTTCAGCTTGGGCATAGGGATAGTGGCAGCTTCTTGAGTTAGAGCAACTAAAGCCCCGGACATATCAACTTCTTGGAGTTCACCCTTTTCTTGCATGTACTCAAGAGGTAGTAAAATTAGATCAAAGACGTGCAACTTATCCAAGGATTTGATTCAAGATAGATCGGAATGGAGAAACAAAATTCATGTAGCCGACCCCAACATAGGTGGGACAAGTCTTTGATGATGGTGATGATGAAATAACTCTGAGGAAGTAGATCGTAGCTGCGTCATTATAGCGTGTAAAAGATTATGGGCATAATTTTGGAATTGTTATTATTATTTTTATATTTTTAATTTGAATGGAATTTTGAAGTGAAGATTTTAGTTTTCCTCCTAGGGATTACCTTGTTTATTTCAATCACAGACATTTGTGGATGTTAAAGATTTTGGAAAATGATCAAATAATAATATTTGTGTATTTTAGAGATTTATGGGTTTA

>ONT.13049.1 LG08 56615112-56620183

AGCCTGTGCCAAAGTTGAAGGTGCCCACTCTTGCATTCATGAAGTTGTGGGTTCAAGTCCTATTATTTGTCCCCAAGTGGAGAATCTCTGCAGGAAGATTTACGATGCTCGTTGTCAATTAATCTAACAGTTCATGGAGGATTCAACACACTGAGCTGCAGCATGACACATAATATTCATCTGGCAGAACCCAAAAAGGCTGAACCATGTAGCAAAGAGTCACAAATTCAAGCTCCATTACCTATACCCAAGAAACTGAGACGATCAAGGAGACTCCACAAAGATTGAGTAAGTGGAATCCTGATGATGTAGTTTGATAATCTGGCTCAAAGAGTTCGTCACATGGGATAGTGTCTACGTGGTTGGATGTATTGACCTTCCAGGTCTTTAGTCGGAAGAGTTTTTGAAGGACAGATGTGATAGACTAGATGTTTTTGGATTGTATACAAAACTCTTTTATTTATGTTGTAAACTTAAAACACCCTACGTTGTAAATATTCTGAGACTGTGTTTGCTCTGCTTACAAGATGTGTTATCT

>ONT.183.3 Contig00516 72976-100483

ACTTCATCTCTGAAACCATCTGTGCACTTTCCTCAAGTTCTGATTTCAAAATCTTTGCTAAAAACCAGAGGAGAGCTGTTGCACATGACAATCAAAGTGTTCCTCAAATCTACAATTTTTTCAAATAGTTTAAACAATTGATTGGAAGAAGAGATTCTCTAGAAAGGTGACCGTGTCTTGCAGCTTTTGACGAAACTGATGCAGGACAATATTATGCTTGGTGCGGGAGGAGAGAACAACCCCATGGTCCAAGCAGGGGTTATCATAGTTCATGATGGAGGTAACCTCAACATGGGAATTCAAGATAATGCAGGAGGGTGAGGATTTACTGGAGCCTGGGATTGAGGGTGATGAGGACTTCGAACTTGTGCCACAATATGACTATAATGATGCCATTCTGGATGGTCAAAATGAATATGTTTAAACTATGAGGATTTAAGACATTAAAGTTAATTATGTTTAAGACTTTATTAGTTTATATTGGGATTGATGTAATTAATTAATCTCGTACTTGGTTCACTTGGAATCTTTTTTGGTGATGTAACAATTGATGGATTGTTATTTCTGGTTATGAGCTTGGCTTACTTAATATATG

>ONT.7424.1 LG04 17884422-17890019

AAATTTGATAGAAAGGGGAAAACGCAGAAAGGGAGGAGAAAGGGGAAAATGGCGACCTCAATTATTTCCTCTCTGTAAATCTGAAGAAGGCTGAGCTCATCTCATCCTCTCTACAAATCTCATACACGGGAGCTCATCTCTTCCTCTTTCTCAATCTCGTGCGCAGATTCTTTCTTTCTCTGTCTTGATCATGAGGCGCTGCGAATCTCAGCTTGGTTGAACGTCCCTCATCTCTTCCTCTCTCTCAATGTTGCAGTAGCTCCTCCCTTCCTCCCTCATTGGATCCAGCATCCCTCCCCATCACATCCAGTATCCCCCTCGCAGGGTGAGAAAAGTGTTGCATTTGAGGATAGCTCTTAAAGGGCTCGAACATGAATGTTCAGGCCAGAGGAATTTCCTTAGGACCATGAATGTTCCTGTCAAGCCCTTGCATTTACAGGGCTTTGTGCTGATCGTGTTTGAGACTTTCAGTCTACCTTTTTTACATAGGCAGCATGAGAGGGAAGGGAGATGGTGATGGCAAGATGCCAAGGCAAAAAAGTGTGGACTCCACAAAGGAAACATTGAACAGGAGAAGGCATCCCAACTTAATGGAAATAGAAATGGTCTTGCACAGGAGAAGGCATCACATTACTGTGTAGTGTGTACCCTTACTGTGTAGTGTTATATCATTAACTATGAATGGTTGTTTCAAATGAATGGATTTTT

>ONT.12899.1 LG08 17847019-17851733

CCGGATAAAATGGGAAGTGAAGAGGCGCAAATCCTTTCATGATCTCCATCTCCCGGTAGAAGCGGATGCTCAAAGTCGATCTTGAGGATTTAGGGAATGAACTGTATATTGGGATTTGATTGGTGTAATCATTCCAAGAAACACTAATACAATCAATAATAAGTAGATGGACTGTTTCGAATGTGTCAAGTGTAATGAGAAGAATTGAAGAAAGAATCTGCCTGATGGTCAAATTGTTCTATGTGTTTGTAACATACACTTAAAAAAATATGCCCTTTTCCTTCTCTGTAGCATTTCAACTCTCAAGGGGCACCAGATGGGAAGCATCACAAATGGTTGAGCGGATTCATGAATTCCTTGTTGAGGAATGTTTGAACTTTTAAGACGTGTTGCAATGAGACCCTAATTTGACAACAGTAGGATGGTTTGCAGAAGTTGCTGTGCTTACTTCTCTACACCTCCATGGGGTCCATCTAAGATGCATGTTTGCGTTGTCATGCTTGCAAAATAAATACGTGGGCCTCACTTAAGATGCATGTTGCCTTGTCATGCTTGATGGACATGCAAGAATCACCTGCTCTCCCTGAACCGACTTCTCAAATCTCCAAAAACATTATGTGAGAAACTCTCTGCATACAAGAGTTCTCCTGGTTTCCCAGGGCCGATTTCTCCAAATAATCATTTCTCATTAACGATGTTCCCTCTCACAGATGCTTATCTCCCATCTCTCAAATTCTAATCTGTTTTGCCATCATACTATCAGCAAGGTTTGAATGAAACTTCAGTAAATCCCTAATTTCTCTCCATTTTCTTGGGAAAAAAAAGAGGAGTGGCATTGGAAGGGGGACTTGTTCTCCATCTCGATGATGTAAATGGCGTTTCCTTCTCTTCCTGAACATACCCACTTGCCAGATCTAGCTCTCAACTTCAACAAGGTGTTACAAGTTTTCCCCTCTCCATCGACTTTCTGCTGATTTCTCTTCCTGTTATGCTCCTGGCCTAATTCTCAACATTTCCATGAAACTCATTTAATTGCTTTTGTAATTTTAGGTACCTGTACGGTAGGTTGATGCCTTTAGGTAGGAATCATGAGTCCTATTTTTTGTCATGTGCTTGCTTATAAATCATAGCTCATACACATAAGGTCATGACATGTGTACTTCTGAAGTTAAAAAACTCCAATTTATAGTAACATGATGGGTTGATTGTACAAA

>ONT.7480.1 LG04 23869471-23873539

TTGGGAAGAGGTCGGGCTACGTAAAGGGGTATGAGATATACTCGAGGACCTCTTCCTCTTCCACAGTCTCGGGCACCGGACCCTGAGGTGGTCGCCCTGCGCGAGCAGCTAGCTGAGTAGGGTAGGAGGCAGGCTGAGCAGCTAACTCAGCAGGACTGGAGGACACGCCAACTTGAGGCCCTCGTTCAAAGGCTGGCAGCTATGGCAGGAATAGGTCCGGCCGCCTTTCTTGACACCACGGGAGAGAGCACTTCTGCTAGGGATGGCACAGCTCCTGGTGGATCATGATGCGACTCTTTGATTGATGGACGCGTTCTGATCGGATGCACACTTTGGTGTTGTTTTGTTGTTTTTAGTTTATTTGTATATGTATTTTGGACATTTTTATTCAACTGTATTTTTATTAATTTAGACATTTTTATTCGACTGTATGTTTATTAATAGTACTGTTTATTTAAGGAGATGAATGATTTGATGAAG

>ONT.10955.16 LG06 69555765-69578300

ATCATCATCAAGGCCAGCCTCCAACTTCTTGATCCTACTACCTCCCAACTCCATCTATCTTTAATCTTCTGCAGCCATGAACAACCAATCTTGCAACCAATACACTCAGACAGGTGGGGGCCAGAACAATCCTCAAATCCAGGGGGTTGGCAAAATCGCCAACAAGAAAGATATTAATATGGGGTCCAGCACCGGTGCAACAAACAACTTCAATAACCAGGGAAACTATGCTGATAAGCAAATCCAGGGTGTGAACGGTGGCTCCATCCAGATGTAACCATGCGTGAAGACAGAGAATTGGATGGATGAGAAGGTGGCGTCACCTTTCATTCTGGCCTATTCTAGTCTTCCAGTATGCACAATCTTATGTTTTATGTTTTCTGTTAATGTAATGGACTCGTTCTGCCTCAGGGTCTGTATTCCCTGAAATAAATAAATAAATAAAATTGGGTCCTTTATTTTGTGTTGCCTACTCA

>ONT.13427.1 LG09 45489624-45503916

CACTCCAGAGGCTTCTCTCCATCTCCTCCTCTCTCTTTCTGAACCAAAAACCTCGAAATCCCTCGAGCGATGGCAGCATGGGCAGCGGTGGCTCGTCAAGCCGCGTCTCTATCTCGGCTCTCCTCTCCAACATCGGCGGCTCAAGCCTCCAAGCTAATCCAACGGCGGCGGCCTTGCAGGTGGCGCAGATCCTCATGGAACCCCCAGGGTTAATTGCTGGTCGGACCCACTAAGCCCAGCTAAATGGAAGGAAGAGCATTTTGTGATCGTTTCTTTATCTGGTTGGGGCCTGCTCATCTATGGAGGCTACAAATTATTCTCTGGTGGGAAGAAAGAAAAGAAGGAAGAGCAAACTGTAGGAGCATCTCCCCCGGCTTGATGGTTCAGTATGACAAGTTGCTTTCATTGTTCGCAGTTGAAAAATAAAGTATTTGCTAAGACACTGCAGTTTTAAGGGGAAGGTATTTCTTGGTTTTGATGTCACTTTTGAGTGGTTTATGTGACTCCTGGATAATGGAGAGAAACACAGAGTTGCATTTGCACATCTTGTGTGTTCCTTCTAGTTCAGTTTCATCTTTTGAACTGTTCAAAAACTGTTCAGATGCTTTCCTTTCTTTGAATGACCTCAATAGTTTCTTCTCATCCAGAGTACTTGTTGATTCTATATTTTCTTCGGATGTTATTGCCATGGATGCATGGGTTC

>ONT.15862.1 LG10 43763300-43764746

CTGCTCCCATGCTCGCTGCATCTCCTTCTTCGATCCTCTGCATCTCCTCATTGCTCATCTCTCTCTCCCTCCCTCCTTTTCCTCTCTCTCTCTTCTTCTCTCTTCTCTCTTCTCTCTCTCCTTCCCTTCTCTCCCCCTCTCTCTCTGCCAGCGCCCTACTTCTCCGGTCGTTTGGAAGATTTGAAAGATTTTACAAGCTCGTTGTCATAGATTTCTTCCAAGCAAGGAAAGTGATTAAAGAAAGAAAGATTCGAGCTTGTTTTCGTTTTCAGGATTTTTATTTTTTTGTGCTCTCTGGGGTCATCAGCTCAGCCTATCAATCAAGGGTTGTAACAGGAAATATGCAAGAGATTATTTCCTGTTTCAAAACCCTGTCTGAGAATCTCAAGTTTTATACCTGACCTGAAGGACTGGAAAATGGCATCTAGGCATGATGTTTGTGTTTTTGATGGACGATGATGTTGCATTATCTGTTCTTTCTTTCTTTTTGTTATTTGTTATTGTCTTTGGTCGGATTATGTGTCATAACTCCATAAGCTGGGGTGCTGATTCAACTTGGTTTGGAGGGTCACTGAGTCAACTCAAATCAATGACTCCACTGATTGTTGGAGTTGTGATGATGGAAACTATGTATAAAATATAGCTGAATCTTGCCAAATTATTTATGGGTCTTACTCACTTTTCCTA

>ONT.7556.1 LG04 61080150-61088041

CTCTCACCTGCTATCCCCTGCGTCTCGATCCCCTTCTCTCCTCCATCTCGATCACCCTAATCCCATCCCAAAAACCCCGCATCTCCATCTTCTTCCTCCCTGCACAGACCTCCCCATTACACCATTTCTCTCTCACGCCTCACCCTCTCCACGCCTCCCCTGTGTATCAAGCATCTCCATCGCCCATCTCACTGCATCCCCTGCGCATCAAACATCTCCATCGCCCATCTCGGTTTCTCCTCTATCCCCATCACCTAAGGCCGATCCCTGCCATTCATCAGAAGATCACCACATCCCTCACCTCTGAGTCTCTCTCGTCTCCCTTTGCATCTCTCTCGGTGTGCCACCTCCTCTGTTATTTTCTCTCCGGCATCTTCAGAGACATAGAGAGAAGCGATGGCGACGAACCTGGTTACATTTCAGAAGATTTTCTATAAGAAGAGAGACAGAACGAAAAAGCGATCGTTACCCCTACGGTGTCATTGAAATCACGCCTCTGCCCAAAAACCTCGGCATTCGCTGCTTCCCCTCTTGAGATATGATATGCAGAGGGGAGTGAAATCTACAAAATGTCAACACCCAGCAATAAAGTTTGCATTTATAGTTGGGAAAGGAAAGTTTTGTCAAAGGGGTGCAGCTAGAATTGGTTAAGAACCATGCGGTTAAGCAAATTGCTGGTTTGGTAAAGGAGGGACTTCACAATTATATATACCCAGCAATAAATTTTGTTGATTAGGCCTTCACTTGGGCAAGTTCGTCACATCAAGATCCCTCATCGGAAGCCTGGCTAATTTTGTGGAAGTGTTGTCTCCATTAATCGTCTCAGTCTCAGACAGGCCACGCATGCGGATTCTATTTTCCAACGGCAACCAGATGAGATTCTCATTTAAAGTTGAGATCTTTTTTGAAGCAGAGAATTGGGTTTGTCGTTTGGTGGGGCTTAAGTAGATATAAGAGGTTTTGTTTGGGTTTTGGAGTTTTAGGAAGGCAACATATGAATGTTCTTCAAGCTTGATTTTTTTTTCTTCTCTTTTCTCAGCCATGTGAGGGGTTTACTTCAGATTGAATGAAAATTGAAAAAACAAATTTGGTTAAAAGAGTCCATGCAGCTAGGATGCAGCCAATATGAAGGCCAAGTATGCAGAGTAACACATTGTTATATGTTAAAATGAGATGCATGAAGTTTTTTAAGACAATTGTGCAGGTGTTGGAATTGTGCAACAAGGAAATGATCATATATGAATATGTAAATTTTCATTAGCATTTGAATGTTGGTACAGAGTAGAATGTTACCCGTTGTTTTAATTTTGTAATGGTAATGGTAAAGTGCTTTCAATGTTGAAATGATGTTTGAATATTAATATGAAGTTGAATGAATTTTCTTATTTCATTT

>ONT.5622.4 LG03 54694446-54701592

GAAGCAACCTCTCGAGTTTTCTCGCTCTTTCAACTGGCACAGGTTATGTGAAGGTGGGGAGCTTTTGGATAGGATACTCTCTAGTATTGATTCAAATTTTGATGAGCATCTCAAGGAAACCAAACTCCATTGATTTGTACTCTTATGATGGACAAGTGGGAGCAAGTATGATGAACCCATGAGGGGTATGCATGATTCAAGTTGGGACTTGTGGTTGTGGTTGGTGTGGATGGGGCAGGAAAGAGCAGGCATTGGAATATATCATGAACCTTTGAATGTCAAGGGCATTGTGGTTGTGGTCTGCTCCTCACATAAATCATGAACCTTTGAATGTCAAGGGCATTGTTTTAACATGTAAATAACTATTCATCTGTAGTTTTATGCCTATGTCAGATATGTATGTTTATAGTAGTCAGTAGATACATAATTGGAACATCATTTCTTATGTGTTTCTTGACAATGTTGTAAAAATATTGCATGGAAATGCTGATTATATTTTTCCTCTGCCCTATATTATGTTACTGAAATTCAAATCAATATGTTGAA

>ONT.10691.4 LG06 45928645-45932204

AAAACATCTCCTTCGAAAACCCTACGTCTTCGTCTTCAAGTCCTCTCCAACTCCTCTGTCTCTCTCTATATGGAAAAAAAATCCAAATTGCAGTGGCAAATCAGCACACCATGACCATCTCTCTTTCTCTCTCGTGGGCAGAAAACCCCCAAATCGTAGAATGAGAAAAAACCCCAAATCCCAGCAAAGAAGGCGGTGATAGCTGCAAGGAATGGTGTGGGCTTTCTTCAAATCTCTAATAGGATGTATTCTGGCGTCCTCACTTTGCATCCTCTCTCTCTCTCTCTCTCTCTCTCTCTCAGGAAGGATTGACTGCTTTGATTGAAAGCCGGTTGTAGGACTTCTTCTCACTGTGGAACCCACCGGGATTCATCCAAAACCAAATCTATCTCTTCTTCAAGATATTTTTGTTCCAACTTACAAGCTTCTAGAGATTGATTTTGGATATTATTGTGCTGGCTGCGGGCTCTTTAGGGATAACTTTTTTTTATTTTGATGACAGACATTTCTATATGTTAAAGATTTTTGAAAATTATCAAGTAGTAATATTTATCTATTTTAGAGATTTGTGGGTTTATTTTGGTCTATGGATGTAGGATATTTGGTTGTGGATGTTATTTTCGATTTGTTAATGAATCTATTTTAAATTGGGTTGTGC

>ONT.92.1 Contig00285 40611-42128

AAAGCAGAAGCTAACTCACCCATCCTGCAAGATGAGGGGGATACTTCTTTTATCATTTCTTTGCTTCAATCTTCTTCTCATTAATGTTCTTGCAAGAGATTTTGCTAGGGTGAATAAGGACGGTGTTGATGAGAAGAAGTTCCTTGGCCTTTCTAAAGGTGGTGCCTTTGGTTTGGTTTGGAGGAGGTGGTGGGTTCGGTAGTGGAGGAGGTGGTGGGTTCGGTAGTGGAGGTGGTGGAGGGATTGGAGGACATCACTGAGGCTCCACGTTGCATGGCCACCTTCTCAGGCCTCCGTCAAACGAATGTGGATTGCTCTTAATTACATGCAGTGAATGATTTTGCAGGCTCGCGTTAGTGCCAAATAAATGTGTTACTATCTTGTGTGTGATTTTTTTTTTTTTTTGGACGAATGTTGTGTTTGTATTAATTACATAGTAAAATCAAATGTATGTGAC

>ONT.712.3 Contig01982 110522-117062

TAATCTTCCGTCGCCAAACCACAAGTAGAGCTGCCGCCCTCTCTCCTCATTCCGTCTCCGACTCTCTCTCCTGCTCCGGCTCCAGCCCTTTCTCCCGTTTCCAGCTCCGGCCTTCTCTCCTCATTCTTGATTTCGCTACTGTAGTGCTGTTACAGAAGAAGCATTAATGTAGTGAATGTAAGGACGGATGAAGTCAACATAAGGACTCAGCGGTATAGGAAAATGCATTTGATGCGCTCCATTCTAAGGGGACATTAGGTACAATGGTTTTGGATTTGTTTTCCTTAGAAAACATCAGTTTATATCAACCAGTATGACCTCCATGTACCAGAGATGACTGTCAGAGAAACACTAAAATTTTCAGCTCATTGCCAGGGTGTTGGAAGCAAAGCAGGCAATTAGTCTGTCTCCACTTTTGTAGTTATATGCTGTTCAAATCTAACACAAAGTTTATATCTATCTAAAGAACTTCTGATGGAACTGGAGAGGAGAGAGTAAGAGAGAGGAATACTTCCTGATCCTGATCTGGACACCTACATGAAGGCTACATCGGTTGAGGACTCAGCGGCAAGTCTTCAAAGTGACTATATTTGGAAGGTTCTTGGACTGGAGTCTTGCTTGGGAGTGATTTTAGTGTAGGTGATGCTATGAGACGAGGTGTATCAGGTGGAGAGAAGAAACGAGTTACTCAAGGGGAAATGATAGTTCAATCTTTTCGATGAAATTATCATAATGGCAGAAGGGAGGATCATCTACCATGGTCCTTGTGATTGTTTTTGAGTTTTTTTGAAAATTGTGGTTTCAGGTGCCTAGAAAGGAAGAGCCCTGCCAATTTCCTCGCTGAGGTGATTTCGAAGAAAGATCAAGCGCAATACTGGGCTCACTATGGTCAACCATATCATTATGTCTCTGTTGAACAATTTGCAGAATTATTCAAGGAACATTATTTGGGTCAAAAGTTATCAAAAGAGCTTTCACAGCCATGCTCTTTTCTCAGAAGTTGCAAGGTGCTTTATCCTTTACTAAGTACTCTCTGAGTAAAAAAGCAACTTTTGAGAGCTTGCACTAGTAGAGAATGGCTTCTTGTGAAGAGAAATTTGTTTGTATATGTTTTCAAATCGATCTAGGTAAATATCATAAGTGAATAAAAGAATCCTTGTCAAAAGAATCCAGGTTATATGAGCTCTCTTTTTTTCTC

>ONT.4291.1 LG02 15255618-15257292

ATCACCTGTAACCCTAAGCAACATCCATTTCTTCCTCCGTTTCTCTTCTTTCTTCCATTTCTTCTTCATCAAAGGTGACTTGAGGTGATTGAAGTGAAGAGGAGTCTAGCTACTTCATCATCTTCATCATCTTCTTAAAGGTTCTTGAAATCCAAGCTTCAAAGGGATGATTTCATCTCAAATCCAAGATAAACAAGGGGTTTAGTGTTTATCAAGCTTCAATTAAGTTACCCGTGCTGGTCCCTCCGACACCGTCCCGTCAGCGGAGGAGGCCCATTCGATACCTCCAGGAGGTCTAACCGAATGGACGTGATTGGAGAGCGTATACATTAGATTATTTTGGAAACTTTTTAGAAAGTATTTGTATATTCATTTTGGCATTGTAAGTATTGTAAATGATGTCTCCTGCATTGTATTTTAAATATGCAATGGTTGATGATAAGTGAAATGGAACAATGTAAATCGAACTTTACAGTTCACAGTAAAGTATCTGATTCATCTTGTTAAA

>ONT.10045.1 LG06 8489952-8496968

CCAGATTCTTTCTTCTTCTTATTCCAATCTCCCAATTCGGAGACTCTCACAGCTCTTATCAGCCTACGGCTATTGATGCTCGCATTTCCGGAAACCTGAATCTGATTCCTACGTTTGATTCCACGCTCCTTCATGGGAAAGTTGGATTTTTGCGGCAGTTGCGATTTTTAGCGTTCTTCTGAAATCTGAAATCGCTAAATTTGTTCGAATGGGGAAGTGAAATCAGGGTTGAGAGAATTGGAAGAATCGAATCTGGAATTTGAGAAATGCAAAGTCAAAAGCCCTCTAAACTTATATATCTTTCCCCAACAAACTGGGTGGGGTACATGGTGTTGAAGAGGATGTTGTCTAGTTAGGGAATTTGGTTGAATGGGTGTTGGGTGTAGTCCATGATTGAAGAGTGTTGGAGGGAGCAAACATTGATGAGCTTGGTGAGGATAAAGAGGTTGTGTTGGAGGTTGGATTTTATAGTGGTGGCATGGATTTGCTTGAGTTCTTTGAGTTTGGGAGGTGATTAAGTAACCTTTAAAAGTACTATGTAATGTTTTCTTGAAAGAAAATCTGCAAAATGACCCCTCTCAAATTAACCTAGTTTTGTAGATTGCTTTGTCTTTGACTTTTGCAGAATAAAATCTTAACAAAAAAGAGAACAAATTGCATTTATTTTCTTT

>ONT.7546.1 LG04 58316023-58321844

GGACTCCTAACCGGAAACCTTTCTCGCTGGTCTGCCCGCTCTCATCGATCCACCGCCGGACCTCGAGCCATCGATCGCCGAAGCCGTCTCCTCTCTCAGCCCTAGGAGGATAGCTTCTCACACACCATCAAAGGCCTCATTATTGAGATAATACAACGAGACGGAACCCAAATGTGAAGTTAAATCAAGGAATCCAACAAAGAAAGAAATCCAACATGAAAATCAAAGAGAGAAAGAAAACTCCATTATGTGGTGCCCACCATCCTTACACAAAAACTCTCCTACTTTTATGAGACTCAAAAACTACATGGGGAGACCAAGCCAAGCAACAAAGTTAAGACTTTGAAGTCAAATAAGGCGGGGCCCATGAAGAGTGCAGCAGCAATGAAGAGAGAAAAGAAAGAAGAAATATCAAAGGTGCCACATATAAGGAGTGGGGTCCACGAGCCTTGCATACCCAGTGGGGAGCCCAATTGTTGAAGAATTCACAAAGAAATCTTCTCTTTTTAAAGACTTTTCAAATTTGAAGGTTTTCATGGTGGCAATTTGCAAGAAAGGAAAGAAGACTATTTTCGAGAAAAGGAGCAAGAGTTGGAAATCCTTATGCAGCAAGTAAATGGAGATGGAAAGATTTTCAATAAGCCCTATCTTTTCTTCTTTCCTCCATATTTCTCTCCTTTTTTTTTTTATTTTTTTATTTTCTCTCCCTTTCTTTCCTTTTCCTTTCCTATTCTTTCTCCCCAAGTCCAAAAAGTTCATAAAAAAAAAAAAAAAACAGAAAATTAGGGGGAGTGGACCTCCAACCTATCCTATCCCATAACACAAGTCACAACACTTGCTTCCACTTTCCACTCTTTTTCACACGCCGCACGCACCTCTCCATCCGTGGCTTCTTTTATTTTTTGCACTTTATGTATATCAGTGATACGTTCAGAGATTGTGAAGATCAAGACCATTAAAAGGCTAAGGGTATTTTTATATGGTGACGAGTGAAACTTTTTTGAAATTTTATTGGCTTTGTGTGTAGACACAATTAATAGTTGAGTATCTAGCAATTTAACGTAGAAAAATAATTTATAAT

>ONT.13342.1 LG09 11062244-11063433

TGAACCGTTGCTCAAACACAGCCTCAACGAAGGTTCCTCTCTGAAATGAAACCCTCCCATTGAAACTCTCCTCTCTCAACGAAACACGTTCGCTCGTTAGCTACTCTGCCATTTCGAGGGATTGTGCTCATCGGCTGATCTGCCATTTCAAGGGACTGTGCTTGTCACTGATATGCTCGTCTCGTCTCCTTCAACGAAACCCTAAACATCTTCGGCTACTTTGTGCGACCCACTCACATCGGCACGTCTGCTCTGCCAAAGATGTGATCATTGAATAGTGGTCCTGTTCTAAACCAATGCCAATACAAGATGGTGTTGCGGAGGTCAAATTTTCTCGATGGGCTACATGAGAGGCTATTCATTAAAGTTCAGATTGCTCTGATTCTGAAATGCTTGGATACAAAAATGTATTTCGTCTAATGTGAAGTTGCTGTCCTCACATTTAAATTGGAGTTTGAAGATGTTGTCATTACTTTGATAATGGATGCTGTCAATGGAGGCTTATGAAGATGTTGAAGCTGGCATGGATTATGAGACTTCTCCACTGTTTTGTCACTGTTATTGTCTGTTTTTTATGATCTGTTAGTTGAGTGTGTTGATGCATTGTGTGATTTGATTCACAACTGTTATGAAGCAGTCTGCAACTTATGGTTTTCAATTAATGTGTTGTTATTATG

>ONT.17207.2 LG12 42664470-42665202

AATTACACGAAGAAACAAAGAGAGAGCGAGGGAAATCGTTAAACAGAAAGATCTTCGTTTCTAGGGTTAGGGTTTCAAAAGATGAGTTCTTCAGCTTCGACGAAAGGCGGTCGAGGAAAGCCCAAATCCTCAAAATCCGTCTCCCGTTCGCACAAGGCCGGCCTCCAATTCCCAGTCGGCAGGATCGCGAGGTTCCTCAAGGCCGGAAAATACGCAGAGCGAGTCGGAGCCGGCGCCCCGGTCTATCTCTCCGCCGTTCTTGAATACCTTGCTGCAGAGGTTCGTTTCTTTTGAGGTTTGGGGAGAGATGTTAGGGTTGGGTGATGTTGAGGGTATGTTTGTAAATTTTCTTGGATTTTTGGAACCTAATCTGTTGCTTTTATAGGATATATAATGCAATGGTAATAACTCATTTACAAA

>ONT.10884.3 LG06 64463833-64467640

GGTTTCTTACGTGTACTACTCACAAGCAAGAAAAGAGGAGGAGCTGCTGGCGATGGTGAGGAAGAAAAAGAAGACGGCAGCTTGCTGTGCAAGAATGACCTGATGCTGGAATAGGAGGCGGGCGTTGACGATGCCATGGTTGCTGTTGCTGTTGTTTCTGGTTGGAGAGGATTGAGCAGTAGAGATGAAGATCGACGGTTCCAGATTTTGTAGGGAGGATCCCTGATGTCTTCATCAATTGGCTCCATGCCAGGCATGATCAAATTATAGGATTTATTTGTGGGGAGGACCAGGATAATGAGGTGACAGTGGAGGATGAATCCGATTACAAACAGGTCAGGTGTAGGACAGATCAGATTTGATGATATATGTGGAAGCAACTAAATGTCTTGAGAGAAATAGACTAGTTTACTTTATATTTCAGTTGAATATTTTATTCGATATGGTTCATAATAACTTCAAGCATGGTTTGGATTTTAACAATCTAGTGTGGATGTTTAATGGATTTAGATTAATTAAATGATTTATGTTCTT

>ONT.201.1 Contig00566 89002-89884

GGACGTTTGGAATCCTCCCTCTCGGTCGCACCCCTCTATCGGCGGATTCCTCCCTCTCGTCGCTCAATCCATCTCCTCAGATCACTTCCCATGTTGTGACGCCCATCGAGTTCCTCCTAAATCACTTCTCTCTCCCTTTCTGCGACGTCCATACCTGGTTTCATCCCTCTCTCTTCTCTCCTCTGTGGAACCCCATCTCCCAAACCCTCCTCTCTAAAACCCTCTGCCTTCTCTCTCCTCTCTGGAACCCCCTCTGCCAAACCCCTCTATTGAACCCCATTCTTTTTCCCCGGAGAAAAAGAGGTTTCAGACGTTGCAGAGGAACGTCACCGGCAGGTTGCCTTGGGTGAATCTGGTGCAGACAAAATCGACAGGTTCAATGATTTGCAACAGTGAGGATTCCAAGCTTCCCACGTCTGCTTTTGGTTTCTACTTGAGCTACTCGGATTATAGGCTTCCCACGTCTGCTTTTGGATTGCAGCCAAGCATCTGAGTCTTCTTCAAGGAGAGCAACAACCAAAAAGGATCGTACCAAGACCTGAAGATTGTTTCTCCTTCACTCGCTTGCTACCGAATCAACGTCTTTATCTTTTTTTCCTTCCTCTGCATTTTTTTCTTCTGTATGCCCTTTTCCCCTTCGAAAGGCTCTGGTGAGGACCCGTGTCAATAATCAAAGCCGTTTAAATGAAGGGCCCATTGTTACATGAGCCCTTGTAAAACTTCACACTAATAGCAAATGGGGCCATTGTTAGATT

>ONT.674.1 Contig01889 381495-383389

GCGCCAGGATTGTTTACTAGTACCAATCCGGATACTCAATCGACAAACCCATTCTGCATCTCCCTCCCTCTCGGTTCCGTCACCCATCTTCATCCCTCATGTCTCTAACCGATCCCTCTCTCGCCCGCCATCCTCTCCACCCGCTACCCTGCGTCTCGATCCCTCCGTCTCCCCTACGTAAAGAATCCCCATCACATGCGCAGACCACCATCTCTCCCACGCAAGCCATATCCCTCGCGACCTCAACCCTCTCCTCCATCTCCCAGGCGACCTGGTCCCCCTCTCCCTCTCATCATTTCCCTCTCACCGCTCCCTCTCACCACTCCCACTCTTGATCACCCTGTATCTCGCGGCTGTGCTTTAATTATTCCCTGCTGAGAGATGTGTACATTGAATGCAAGCACACCTGCACTTGCCATTCCCAACGTGAGCTCTCAAGTGATCCCTTATGGAGGTTTTTGTTCATACATGAATCTGCTCTGATTGGAACATTTTTTTGGAAGCTGACAGCTTGTGATTGTTGTGGTTTTTTGGGGGAATTTGTGTCGTTTGGTTCAAGAAGACGAGGATTTGTGTTATTTTTTTAGTTAGGTTATGGAAAGATGGATTGGAACTAGGAATTGCGATTTG

>ONT.221.1 Contig00652 70823-75239

TCTCTTTCCTTTCAAATGAAAGCCCTAGCCCTACTGAAGAAAGTCTCACGCCCTCTCCTCTGCCTCAGATCCCACAGCAGCCCTCTTCTCCGGCAGCGATTCGGAACCGGATCTCTCCTTCTCGTTTGTTTGTGGTGGTGGGATCCCAATCGAAATCAACCCAGGCTTATCTCTCTCCCGATCTCTGCTTCTGCAACATTGCAAGCAGCTTCGTCTTCTCCGTCACCGACGAATCAGAAACCGATAGCACTCTATTCTTGGCCTTTCATCAGAGGCAAACCTGATCGCTTCTCCGTTTCTCTGTTTCAAGAAACCAGAGACCCTGCTGCTTTTCCAATCTTGTCCGCGTCTTCTCTGGCAACCTGATCCTGTTCTTTGGTTTAGTCTGCAACCCGAAACCCTGATCCTTTCTTTGGCTCTGGGCAATAAAACGAACAACAAGGACGAAGTTAATCCCAGGCCTAACTAAGTGTGAATCACAGGCCTAACGAAGCCCCTCTGCAGGCCTTTGGTGACTCTAGCTTATCCCTTGAACTTTCCTCATTGTGATATGGTGAAGGATGCTTATGGAGGAATCTCTTTTGTATGCAATAATACTTTTGAATATGGCTGTGATCTTGACAGATTGAAATGGGAGATGAAGAGAAAATAAGAACAAGCAACATTTTTTGACCGATTTGGATAGTGCGGATCAATTTGAGGATGCTATATTTTATCATGGAAATTGCTTGTCGTCTCTCTGTGATACATATTGAAGACCGCATTTCAATTATTGTTTCTATGTTATATTGAGACCAACTATTATATAGATTTATGTTTAACAGAATTTTATCAATATCATTCAGTAAGAATTTGTGATGAGGACAACTGATTCGTTGCACTCAAATTCAATGAATTAATTTAGTTTATGTTTTT

>ONT.7296.2 LG04 8915586-8933663

GGGTTTCTCAATCCAACATTTACCCCCTTCCTCCCTCTCTACCTCCTCAACAAATTCATTCCAGCTTCGATCAAGCACACACCTTACCTACCTGACTACCTCTGTCTGTCTGAGTCTATCCTCTCCTCTCTCGTTGATGCCCTTCTTTTCAATATGCTATGTCTGAGTCTCTCCTCTCCTCTCTCGAGTGGCCGCAAGAAGGAAGGTCTCTCTTTTTGTTTTGCTCTTGATTGGTTATCAGATTACAGGCAAAAGTATTTTGAAATGTGGTGGTATTGATCCCATTGCCAAGGATTGTATCATGGACACCAAAAACAAACAGCAACTCCATAAATGTCTTGAAAGATGAAAATGCATACGGTGGTGGAGAGGCTAGCGAACAATGCAGTGTAGAGACTCACCAAAGGCTCTCTCTCTCTCTCCGCGATCAACTTTACTGGAACTCCTTTCTTTCCTGGAACTCCTTTCTTTCAAGAGACTATTAAATATATGCTATGATGCCCAGTTATGAAAATGGACCTCAGGCCACATGTGTTGGGCCCAAGCATATATAGGAATTTTTTTATCTATTTAATTGTTGTTAGTTACTATTGATGATTTGTATCTAGGATTAGGTTTCCAATATTATTTAGTTTCCCAGTTTAATTAGGTTTCATAGGGTTTAAGTTTTCTTTTCCTATACATATGGTATCGTAGAAGATTATTTTTAGAGTTTATTAATTGAATTGAGTTTTCC

>ONT.249.1 Contig00712 5890-95852

TTTTTCTCTGTAGTTTGGAGTTTGGACAGTGGACAAAAATAACTTCCCCACCTTCTCTCTCTTACATGCACGCAGAACCTCGAAATCTCTGTAGCAATGGCGGCATGCGCGGCCGCGGCCGCGTCTCTATCTCGGCTCTCATCTCCCAAATCGGCTGCTCAAGCCTCAAATCTAGTGCAAAGACGCCGTCTTGCGGGAGGTGGAGATCATTATGGATCCCCCAAGGTTCACTGCTGGCAGGACCCATTAACTCCATCTAGATGGAAGGAAGAGCATTTTGTGATTGTTTCTTTATCTGGTTGGGGGTTACTTTTCTATGGAGGCTACAAATTCTTCTCTGGTGGCAAGAAAGACAAAATTTCATAAAATCACAGGAGCACTCCACTAGGCTTGATGGTGCAGTGTGACAAGTTTCGTTGTTGAGTCACATTTGAAACTAAATTATTTTCCATAACTTTGCAATATGTAATGAACGTATTTTGGTTATGAGATGTCGATTTTGAGTGACTAGTTTACATAACCCCTCGTTTATGGTTGAAAAATGCTGAGACCATGTTGCATGTCCCGGTCATGTGGGCAGCCATTGTTGATCCTTTCTTATTTCTGAATGGTTGCAGTGTTCTACCTTCTTTTATGGTGTATTTGGATGCTAGGATTT

>ONT.13676.1 LG09 61101252-61102887

AAAAAGAGATTCAATCGCCCAAATCAAACAGAAGAACATAGAGTTGAATCGAGCATCCTCTCTCCCAGACCTCGGCTCTCTCTCTCGTTTCCCTCGCCCCTTTCTCATGCCGTCCATCTTCCTCATATCTTTCTCGGACTCTCTTTCCCTCGATCGGTTAGACGCATGATCTCGTCCCTCTTCTTCGCCTTCCCTCTCTTAATCTTGCCCTAAATCCATTGAGAGACGAAAAAAATGGCAGGACGAGTTTTTGTTGAAATTTTTTTTTTTTCATAACTGAGCTCTGAAAGTCTGAGTTCACCCCTCTTCCTCGTCTCCCCCTCTGTCTGCCGATCACGCTCACCTTTTTCTGATTCGGACTCTCTCAATCCGTGCACAGTAAGAAAAATGGTTGTGGTGCGATTTTCATGGAGCATCGAATATAGAACTGTCCGAGGTTAATTCATCAACGTGTGAAAGAGACAATTGGACAGAGCAACCAGAGAGTTCAACAACTACACTTGAGAAATTGGAAATTGTAAATTGGAAAAAGCAGTGCGTGCAAAAATTGAGAAGACAAAATACGGACTTTGTATTTATGTAAAATGTATCATGCAAACGCATCCTTCTGTTGTATTATAATTTTTGTTTTCATATTGTATTGGGCAAAGATACTGTAAAAGGTATTGTTTATAATTTGAGGTGGCGGGTTGTAGTTTGAATTATCCTAGATATAATTTTAAATTGCAGTTTGCAGATGCAA

>ONT.6970.2 LG04 79595775-79604268

GCTACTTGCTAGGCTTAGGAGAGGGAAAGAAGGGTCGTGGCGACTGGCGCTGGAGCTTGAGCTCGGCGACTTCAACGAGATGATGGTGGAGTAGGCGATGGCGACGGCGAACAGACGAGCGCGGCAGCGACGATGTACGATGGTGGAGGCGACGCTGCTGCTGCTTGGACTCGTCGTCAATGAAGAGCAGAGTTCCAAATGGTGCACGGTTCAAGCACATGTGATTTTGGATTACTGATACGCTGTCATTAGGTGGATTTGATGGATGGGGCTCTCCGATAGCCTAATCACCACTCTCATGAATATAGGCGCGCTCGTGCACAGCAGCTGCATTCCCTCTTCTGTGGCACTTGCTTACTAGCGGGGGAGTTAGGCCGGATGTCATGCTGGAGAGGAAAATATCAGCAAAACAAAGGGCCGAAAATCATGGAAGGTCGAAATTGTTTCTGCACCAAACAGGTACAGGATGGTGCCAGAAAATCTAGTGCCTTATCTCCCTTTTCTGTGGCAACATATCGTTGCACAAACACACCCTTATTCTCTTGGATGGGAATATGATTATCTTTGATTTCTTGAAAGAGGCTTTGTACTTATGTAAGATAAATAAAATACAGAAACGGCAATTTGGATGATTAGGCTAAGGCTTGAGGTTATGTACAATGGAGGCTTCTGTTTTCTAAGACTACTCATTCTTCAACCAACCAAGCACAAA

>ONT.3438.3 LG02 41868389-41869603

CTGCAATGGAGGCGGCCAGACTCTCTTATTGGAAGAAAGATTATATCTTTCTCTCTCTGCAGCCAACAAGAAGCAGTTGCAGATCCAAAACATGAGTTGTAAGTGATGGTGAAATAACAAGGACATGTGCACTCCATCTGAGGATGATGACTCGACTTCGGAATCTCTCTGCTCTTCGAACTTCAAATGCAATCATGTTCTGGCTTCGGTGACCTTGTGCTTTATTACAAGTACTTTCCTTTGTTTTCTCTGTTATTTTTCTTTCTATATATCTACATATCTTTGACCATGAATTGTTGTTGTTATTATTATGTTTTTATTTTGGTTGGATCTGTATGTCCCCACCCTTTTCATTTTCTTATCATTCCCTTTCCATTTGCTGGTTTTGTTCAACTTTAAGGAATC

>ONT.4106.4 LG02 1698168-1705494

CTAAGTCCTAACCGGAAACCTTTCTCGCTGGCTGCCCCCTCTCATCGATCCACCGCCGGACCTCGAGCCATCGATCGCCGAAGCCGTCTTGTCTCTCAGCCCTAAGGATATCTTCTCACACACCATCAAAGGCCTCAGTATTGAGGTACCGTAAGAGTGAAGGCACAAGAATGGCTTTTGTGATGAGGTGAAGTCTTTGAAGAGTCTTCATGAGTTCCAGAAACAGTTAGGCCAACCAGATGGATTTCCATGGTACACATAGAAACCATGGTGTTGGCCAATCAAGTGAGACATTAACATATGTCAGAAAAGAGAAATCTGGAACACTCCTTAGTGAATACATTGCTTTTGAGGGCAATTCCTGGGCTGAATGAATTTGGCAAGGCTTTATTAGGAAATATGTTCTTTGTGCTAGAGTTATTGTGGGTGCTGTTTTGATCATTGTTGCCTATGCTATTTGGATGGCTAGAAATTGGCTCCTCTTTGAGAAATTGTAGTCCATCTCAGTGAATGAAGAACTTAATTAAACGGAGATGGAATGCCTTTTGCTAAGCGGATATACTAATTATGTTAAGTTAATTTTGGCTAGTTAGGGATTTCCGGAAATTGTTTACAATGTAGCTTGCCTTTTTTTGGATGCTTTTGTGAAGGTTTTGGATTATGTCGTGACGCTTTGGTTACGAATTCGAAGGTTAATGTAAATAGTTTGGATTATGGAT

>ONT.6085.3 LG03 48804073-48811439

GGATTCGCGCTTCTTCTCCTTTCCGCCAAAAGAAAAGACCACCGCCACCCCTTCCCCAAAACCCTCTCACCCCACAAACTCCCACCTTTTCTGTATCACACCACCACTATTGATGAAATGAAATCCACCACGCAAATGCATAAGACATAGAGAGGAAAATTTGAAAAAAAAGAAGAAGGAGATCCTCTTCTTCTTTACAGTGGATGGATAGTAGAAATGAATTCTCTCTTGTATTTTGTTTTGTTTTCTCTCTTATCATCAAATCAAATGAGAAAATAAGCTTTTGGGGGGTTTTAGGTTTCATTCACATCTTTCCAAGTTATCTAAGAGGGCTGCTGCTGCTTCTTCTTCTTCTATCGATGAAGAGTTCTTCTCTACATGTGCAGCTCTTAGCCTGGATGGGAAGCTCAGAGAAAGATGGCTCCTGCCTCAAAGGTTTCGGAGATGAAAGAGATGCTTACTGCAGGATTATGCATTCTTCGAGCATTATATACCAAAGAATGTTCAAGTCCTTTTAATATCGGCAATGCATAAACAAAGTAAGGCATCTTTTGGAGTAGGGAATGAAGCAAGTGGTCCGCATAGCATTAAAAATGGTCATATATTGACAATCCAACTCATGGAGTTAGCAACACATTTCTTTTTGCCTGGAGTTTTCTGATTCCTATATTTTGATCTTCTATCTGATTCATTTGTATTCCTTTTGTATTTCTATCTTGTATCCTTTAGTGTTTGTATACTTGGAATGCCCTGTACGCTAATTCACCAGAACTAAATTCTTTCCAAGTTCTTTATTTC

>ONT.8760.1 LG05 97788133-97793891

GCCAGGCCCAGCACAATGCGTACAACGTCGGCACTCTGGCCCAGCATGGCCCGTCCATTCGGCAGGCCACAGCACTTTCTCTTGATCTCTCTGTGCAAGTTTCAGGGGTTTTGTGACATGATTCTATGTGACTTGTGATTTAGGGTTTTTGTGTGATATGATTTAGGGTTTCAAATCAAAATCAAAATCAAAATCAAAATCAAAATCAAAATCAAAATCAGAGAGCATACAAGTGTTCGAATTCTGCTACCATATATCAGCAAGTGTTGGGGCTTTTTCCTGTTCTCCCTTCATGGGCTATCTAAGTGTTGTTCATATGACACCCAACTGTCTTGAGTAGAAGCTTCCAAAGGAGTTTGATGTGCGACATTTGGTCAAGAAAGGATATTCTATCCCTAGTTCTCGGCGATGAGAAGATGTCAACTGCATTCTGGAAAGAGAACTCTTAAGGTAGGCAACAAAGGCAGGCATTTCATGTGGTAACTTATACATTATCAACAACCAACCAGGTTCTGTGAAGGTGGGGAGCTTTTGGATAGGATACTTTCCAGGTGATTATTTGGTATTGTCATATGGGTATTGCAATGTGATTATATAAGATTGTTGTGCTAATTTATTATGGTCCCATTATCCCATTGTCTTTTTTGTAGTGAATGTTTGGCCTGGCAAGGATTTATTGCTACTTTTATTCTGATGGTGTTCTTTTTTGGTTTTGGAGATTGGTTTTGAATTTTATTATTCTGGCTGCAGACTCTTAGGGATTACCTTGTTTATTTTGATCACAGACATTTGTGGATGTTAGAGATTTTTTGAAAC

>ONT.1289.1 LG01 43228197-43231524

CCACAATCCACATCACAGATCGGTTTCCTCATTTTCATGGTGATGCTTGTCTCCCCTTTCTAGTTTTTAGTTTTGAGTAAAGCTTTTGGTTACAATTCATCTCACCTGTTTATTATGTGCTTGATTTCATCTTATTGTTCCTTATTTTTTATAATTTCTTTGCTTTTCTCATCTGGGTTCTCCTTTCATGCCTCTCTATTACAACCGGAGAGGAGCTTGAGATGTGTTTTTCCACTGGAAGGATTTTCTTATGGTGCCCTTATATGTTGCTAGTTATATTTATCTGTTAGAATGTGTGGTTGTAGTACAGTACATCTCAATCTGTTTATTTTGTGCTTGATTTGATGTACTATTTTAGATTATTTTGAGCTTTTTTGGCCTTTTCTCATCTGGGTTTCTCTTCTTCTTACGATGCAGTTTTCCATTGAAAGTATTTTCTTATGGCACATTTATATGTTGGTGGATGTCTTGATTTTGTATGTTTGAAAAATCTGTCTTAAAGTTTTTGATACTACATAAATACCTTGTTTCTGTATCTTCATATTTCAAGCCTAGTCCTTAAGATCGCCTTCCGTTTTCTCTCTGGAAGCGTTTTGAGTCATAACTAGGCAACATTGATTCTGGGGTTCTTCTAAGGGTTTTTCTTTGAAGCCTTGCATCCTCTACTTTTATCATTGAAGGGGGCATTTCAATTTGGCATGCATTCCTTTTTCATGTCCTAAGATCAATAGCTTGTGCTGTTTGTCTCTAGTGATTTTGTTAGGGGATTTTGTTGCCAATGTGATGAGATGGTCGCCCGAATGGTACTGGATGATACTGATTGTTTCTTATTCGTATTTCCTGAAACATTATGTATTTTATCTACCTTTGCAAAAGGAAATGAGGCAAGAGTGTTGAATCAATTAGATGTGTGAATGGAATATTTTTCCTTATGATCTCAGGCTTATTGATATTCTGACTTGCCACCCCTCAGGTTTTATTGATTTTTACAAACAGAGGTGGGCAATTTATTTTCATGGTAATATGATCCAGACCTGCAACCCACTAGTTAGTTGTGCGATCACTTTCCCACCAGAGGTGCATCATGTTAAAAATATTTTGGGGTATTTGACCAAGTAGGGGAAATCAAATTCTTGTATTATTTAAAAATTTAGCTCCCGAGCCAAGCATGACATAGCTTGAGCTCAAGGGTGGCTTGTAGTCTTTTGACTAGCTCAGGTACTCAGATAAGTCATTTGGCCTGGCTTTGCTTTGCTCCTATTGTCAATACACTTAACTCAAGCTCCTCTTTTGGAAACCTCGCCTCTCATTCAGCTGTCAAGGGATTGATCCTTAGGTATGCTTTTTTCCGTATTTGCGGGTGCTTTTTTCCAGCCACTGCATGAGTAATATGAGAAGAGAGTACTTGCACATGTGGGAAGAGAGCATGCCTAGGGATCAATCCCTCGGCACTTATATAAGTGATAGGGTTCCCAACCAACTAGCAACTCCTCGTGTCATGTAGGTGTTCACTCCATGCGTGTGCTTTCATGGGTCTCACAAAAGAAAAAGGGGCTCAACTTGGCTTGGTTTTGTGCACATATCTTTAGTAAATATCATTCAAGATATAATTTTATGTTGAATATTAGACACTACTATTAATGAACATATTTTGAAGAAATCAATTCAATTGGATTTGTAACATATCCAATTGTATGGATTTAATGGTGAATATTATACTTGTTTATTAGATAATGTCTAGCTTAATCTACATATGAACTAAAAAAATTCCTTACTTGATCCACATAGTTAATGTCTAGCTTAATCCACATATGAACTAAAAGAATTCCTTACTTGATCCACATAGGTAATGTCTAACTTAATCCACATATGAATATAAATTTTACAAGCTTAGGAACTCCTTACTTGATCTGGAACTTTTGATGGAATTATCACATCACAAATTTTCGATAGATCTATTAGATCCTGTGTCAAAAAAATGTGAAAGGGATAGCAAGAGTTGTCACATTAAGAACTCTTTGTCTATCTTTTACATTCATTTGATATGAGCCTTGCTCAATTGTATGGTGAATACTCTCCGGGTTAGGATAATTATGATGGGAAGGCTTTTGAATTTAAATATTCTTCCCTTTCCTACTTTTGTTGACTCCCTTATACAAGTTTTAATCATCATTGACATTAATGGCGGTAGAGTGAGGGATATTGTAGCATTTTACTCAATAGAAATAGTTTACAAAGAAATAAATTACAAGGGTCTTTGGCTATAGCTCAGTTGGTTAGGGACATGGGTGTGTGTAAGAGTGCGTGAGTTCGAGTCTCTAATGTAGCTAAAAAAATTGCAAGGTGTTTTTTTTTTTTTTGCTTTAAAAGGGATTCGAACTTGCGTACTCACGCACACTACACATGTCCCTAACCAGCTGAGCAACAGCTGGGGGCTATTGTGCAATTAAAATTCACATTTCACGGTAAATGTTTAGATCTGAGAGGTTGCATCCATTGCATTAATTTAAGCATACCTATTGTGACATGATGCTCATATTCAGAGAATGGAGAAGACATGGAAACCTTCCATTACAAGGTAAATCCCTATATTCTAATATACCTATGAAATAAATTGCCACTCTTAGATAGAGAAGACCTCAATCTTTCGAACATTCTTTTTTGAATTGCATGATCTTCATTGCAGACTGCAGTTACAAGGGAGACCAAATCGTATTCAGCTGAATCAAGAAATTATAAACTTGCTTCGATTCACAGCTTCTTTCTGTGCTTTCACTTTTCTTTCCTTCTCTCATGTTTTCTGTGAGAAGATTAGACAAGTTGCCATAGGGGTGGTCTTCAGTAAAGTATGAGAGAAGACAATGTTGTCTCCACATATCCTTCATTGTACATGTGATGGATGTGCCTAAGCTACTTCTTTTGTCTCAAGTCATAGTGAAGTGAATTGCACTGTGGATGTCAAATTTTGGGTGTGTGAATTTGAATAGTCAGGGGAAGAATGGAGCAGGGGGTGTGTGAATTTGAATAGTCAGGGGAAGGGGCGGCCACCTGTTTATTCTAAGTCTCAATTTACTTAATTTCATTTATAAACTACCAAATGTCCCGCACAGGTGATTCATCAAGTTATGCGTGCTGATTATCATCA

>ONT.8770.1 LG05 99020231-99090823

CATCAACCCAGCGATTTTTCCCCCACTCTCTCTCTCTATCTCTATCTCTATTTCTCTCTCTCACTCTCTCTCCCTCCCTCCCTCTCGCTCTGCACTGTGCCCTCGCTCCCGCGCGCCAGAGCCGTTGCTGACGAGGTGCTGGAACAGCTCCCTTTCTCCGAAATCCTCTCCATTGAGAAGATCTCTCTCGGCGGAAAGAATGTGAAACCCATCCGTCCTCACAGACGCGTTTTTCTTCTCTTCCCCTTTCATTTCTCCACTCGAGAGATTGTAGATTTTTTCATCTTGTGTGGAGTGGTGAGTGGAGTCATCGCCTTATGGGGGTTGAGTCAAGAACGAACTGTGCTTGTGGAATCCCTTGCTAAGTGGTAAAAACTAAAGAGAATGGGATTTGGGATTCTCGTTTTTTAACACAGAGCAATGGTGGTGGTTCGGGTTTGAGAGCATCGGATCCATCACCGCACCCTGCACGGCCAAACCCTTTTTTTTTCTTTTTCTGTAATTGTAATGGATTTTATTTGTTGAAACTTTTGTAATGTTGTTAATATAATTAATATAAAATTTTCTTTTGTTATA

>ONT.10169.2 LG06 49479755-49480782

GGACATGCAGCATGCCACATGGCAAGAGGCAGGGAGCCATTTATCTTGCTCTGTGCACTCTACCACTAGTTCCAATAGCACCCCAATGGCTACTTCTGCTTCCATTTCTCCCTTCCTCAAATCCAGAGTACTTGCAAGGGATCAAAAGGAATCACAGATCTGAATCCTATAATTCAGGATATATGTGGTGAATCTCTAGTTGAGAACAAAGATGAATTTCTCAAAACTTTTTCAACAGAAACTCAGTTTTTCAGAAACATCATCTCAAATGGAGAAGTCATAGGCTCTGAAGCTTCCAAGGGGCATGATGATGCATCTTATAGCTATTTGAGGCTTCCATCAAGGACGTGCTCTCTTAATTTTACTTTTATTCTCATGTGCATTTTGTAATTTCATGATGTTATCTGGCTCTATGTCTGGATTTTTCAGTTAAATAAGCATTTCCAAAAGTAATGTTCTAGACTTGACCAGTATGTTTGTGGTTATGATATTTCATCCTGCAGCTGATTGAT

>ONT.14954.5 LG10 48514267-48515408

AAGAACAAATACGAGAAGAATAATACTCTCCTCCCCTCTAGGTTTTCTCTCCAAAACCGTAGAAATTCTCTCATTCCAATTCTCCACGCGTCCCTGCAAATTCATCCGACATTCCTGCTTTTGCGTATCTCTGAGGTCACTCATCTCTGTTGCTGTGGGGATTTTTTATCTTGGGTTGAAAAAGCTGGGATTTCTTTTCTGGTAGGTTTCAGTTTCCACGAGGAATCTGGGATTTGTTATTTTTGCACGAGCCATTGGTCTCATGAGTATTGTTAGGGGGATGCAGAGATTGATTTGGGAGGAGCAGAAGGCGCTGGGCTGGAAGGAATCATTTAGTCAAGGGATTGGTGTCACCTTGCCCAAGGCCAGAACACGCGATAAAGGGCTGATAACATTGAATTAAGAGGGAGAACAACCGAGGGTGAGGGCTTCTGTTGGA

>ONT.10219.3 LG06 56438886-56444223

GCGCCTTTTGTTTTCACAAATGTTCCCTGCGCGCGGTAATTCACCCCTCCCCATTTCTTCTTGCGCCATTGGAGACCTCCACCATTTCTCCAATCGCATTCTCTCGTCGCCATTTTCTAAGAATCCCAAACGAGAGAGAAGGTGCGGAGGAGAAATCTAGAGAAGAAAAGATCGAGAAGAAATCTGGAAAAGAAGACGAAGCTAGTCAGAAGCTTCCAAAGGAGTTTGATGTGCGGCATTTTTTTCAAGAAAGGATATTCTATCCCTAGTTCTCGTCGTTGACAAGATCTCAACTGCATTCTGGAAAGAGGATCTTAAGAAGGTAAAGGTATTAGCTGGGGCACCCCAATAGATCCTCCGTTATATGACGATCCGAGCAACGCTCTTCCTGGCGATGGAATCCGACCCGAGATTGCAGAATCTGTAAAACAGATTGAATGAAAATTTAAAAACAAATATTGTTGAAAGTGTCTATGCAGCTAGGATGCAGCCAATATGAAGGACAAGTATGCAGAATAACAGATTGCTAAATATTAAAATGGGATGCATGAAGTTTTTTAAAACAATGGTTCAAGTGTTGGAATGGTGCAACAATGAAATCATCATATATGAATATGTAAATTTTCATTGGTATTTGAATATTGGTCCAGAGTAGAATGTTACCATTTGTTTTAATTTTGTAATGGTAATGGTAAAGTGTTTTCAATGTTGAAAGGATGTTTGAATATTAATATGAAGTGGAATGAATTTTCTTGTTTC

>ONT.12836.3 LG08 6795098-6799065

AGGCGACTTCATCCAGCGCAGAACCACTCACCAGAGTCTCACCCTCTCCACGCCTGTCTCTCCATTACCGACCCATCTCACACCCTCCCCTGCGCATCACACAGCTCGGACTCTCTATCCCCATCACCTGAGGCCGATCCCTGCAACCCATCCCTCACCTCACCTCTGCGTCTCTCTCGTCTCCCTCCGCACCTCTCTCTGTGTGCGTCTCCCAGGTTATGAAGACGAGAGGTTGCTGCAAATGTTGCTGAAAGAAGCCGTCCATGTGGTCAGCTGTGTGGCTATGTGGCCAATACAGAATGGGGCAAAGAGTTTGAACTTGGTGGCTAATTGAAGCTATTGGAGAGGGTTGCTTACATCGACACCATAGTTTATCCACTTACATCCATCCCAGGAGATGGGCATTTTACAAAGTTATTGGAAGTTGGGCTTCAAAGAAGTTTATGACAGGATGGTGTGCATCATTTCTCAATTTGTGTTTGATCTTTATCTTTCACTCAGTGTTAAAACTGATCTTTTCTTACTGTCATAGATATTGTTTATCTATTGTCCCTTTCCGAAGTTTTTTCCTTGATTATGA

>ONT.1327.1 LG01 70785170-70793245

TTTCTCTTCTCTTCTTCTTCTTGTTTCTTCTCTCCCATGGCTCGGTGAAGAGCACCTGGAGGGAGACTCTCCTTGCAAGATCCAGCCCGGCCATTCAACTTCCAGTCATCCCCTACACAACATAGTGCGTTTTGGTGTGATTCCAGCAGTGGTAGCCCCCTCTTTCACCACCTTCTTCTTTCTTGATTTTCTAAGAGGGTGGAACTTTGGGGCTTTGTTTGGAAGAGAGAGCTCTTGGTTGGAGGTTGGATTGACTGTAGTTTGAGATGTGATCTGTTCTTGATTAGAGGCAAGTATGTTTCCCTCACTTGTACCTCCCGGTACGGTTTCTAAGGAGGGGGTTGTTGAGCTGCAAGTGACGTGTGATTGAGGTGCAGAGGTTGTCATATAGACTCCGTTCCTTGCCATGAGATGCTATATACCTGGGAGTTTTATTTTATGTTTAAATCCTATTTTACTGGCCTCTGTTGGCCTTTTATTTTGGGAATGTAATTAATACATCTTTTATAATTTCTAACTTGATATATAGGAATTAAATCTCCTGTTTACTT

>ONT.10102.4 LG06 15931420-15947199

GAACAAAAAAAAAAAAACCCAAAATCGTCTCTGGCTCTTTCAACCTTCCCTCTTCAGCTCTTCTCCCGGTCTACAGCTCTTCCGACCTAGTGACCTTCCTTCTTCCTTCGACGCTTCGACGGCTCAGGAGCAAGCCTCTACAGATATGGGATAAAGAAGTAAAGAAGACTATATGGAATAACTTCTCTGTGCTAAGTTGTGTCAACACATTCACCCTTTTTGTTCGATGAAAAGCTACATCAAAATGTTGCAAACGATGGTCCAAAGAAAAGTCTTCTAGCAGAACTAGACGAAGGTGATGGTGTAGACACTTCTTATATATGTGGAGGAGGCTCAAAATAATATACTTATGCAAGAAAAGCAATTTGTTGCAAAGAATGCTACAAGTTAATTGGAGGGCTTTTCCACCACAACAATTGGAATCAGCTAGCAATAAACAACATTGGAGAAAGTTAAAGCCACAACAAATATGATGGGATAGACTTTTTGGTTGTTAATTTTTTTTCAGAAATGGAGTGGATGAAAGTAGCTGGAAGGTTATATGAGGTGGATGTGTGATGTATGGATATTCAAATGTTAAATATTTGAGTTCAAGTTAGTCTATTGATTTCAATTTTA

>ONT.7059.2 LG04 86525847-86716175

CCCTCACCCAATTAACCCCTCATCTCTCGCCCAGCAATCCGTCACCTCTTTGACCCTTTCTCGCCTGCCATCCCTTCCAGCAGCTCTTTCCACCGATTCCTCCAGCGGCTCTTTCCAATCCTCCTCCTCCATAATCACGCCTTCTTGAGCTGTTTTTGAGGATGACTCAACTCGGGCCAATTTTCAAAACACAGAGCTAACAGACAACAGATTGAATAAAAATTGAAAAACAACTTCTGTTGAAAGAGTCATTGCTGCTAGGATGCAGCCAACATGAAGGCCAAGTATGTAGAGTAGAATGTTTGGCATTTGAATGTTGGTATAGAGTAGAATCCTTTTGTGGTGAATGACCACTTCTCTTACGCGGTGATTCTCCACTGTGTGCTAGAAGATAAATCTGATTGGGTTGACAAACCATTGTGTGCTAGATATATGCTTTGTGCTTGTGTATGTTGGCTGCCCACCACTTTAGAAATCCTGCTGTGACTTTTCTCTTATCATGCAACAGTATTGTTGAGTTGTAAACTGCATCATGGTTCTGGCCTAATTTTCAGTTTATTGACCGGTTGTTCGAA

>ONT.356.4 Contig01018 76141-79026

AAAGTCCATGTAGCATTGCCCTCTCTCTCTGCCCTCCTCTCCGCAGATCTCCATTCCCCTCACGGTTCCCACCTGCTTCATCTACACCACCTCTCCACCGTCCTCCTCTCATCACCTGCATCCCATACCCCCATCGCCCTCACTGCTCATCCCTTTCTATCTCTCCCTCCCTCTCCAGACATCTCCCAGGCCCACGACCCACTTCATCTTCCTCCGCCAGACGACCACAGCAGCACTGCCAAGCGCGCGCAGCAGCCCCCTTGACGCCCGCAGAGCCTTCATCTTCTTCCTCGCGCCGCCGGACGCCTCCCCTGCACCAGACAGTAGACTTTCTGGCATTTCGTATCTATTTATTCAACGAAGAATAGAGAAATTGTATTATTGAAGAAATGAACCAAACCCTGCTGCAGAGAGGGGAAAAATGGATGAAAAAAAGAACAGGTTGCAAAAGGAGCCAAGAATTGTGGAGGGCTTCAAGCTCATTGAATTCGTAAACCCAAAAGTTGACAAGAAACCGGTTCAAGAGGTTTTATTTCTAAATAGACCATATTTTATCTCATACAATATTTGTAACTTATGTGTGCATCTTCAATTGGTATATTAATATTTTAGGAAGTTGAGTAATTTGAAATGCCTTGGGTTCTACCTGGTGCTTGCTGAGGTGACTGTGTGGGGGAGCAAACCATAGCATGGAAGTAACATTTTGGACAATGAAAGGGGGAGGAGCACCTTGTGTACTGTTGCTTTGTATATTTTTTCTTAATGACCATCCCTTTTACGTGTGAAATAGACCATCAGTATGCACTAATTTTTCTCT

>ONT.15931.1 LG10 68457970-68464620

GAGATGAGAGATTTCTAATCTCAAATAGGGCCTCACCTACAAGTCATACTAAAGTATCACAAGGGGTCACACGACCCTTATATTATTCACAATTCACAAAGTCCGGCACGCAAACACAAATTGCAATCGGCAGTTTGCTCCCTCTCCCCCATCGGCAATCTCTCTACCTTGTCTCTCTAGATGGTGAGGATGATGCAGACGCTGATGATAATGTAGAGGAGGTTCCCGATAATTAGACACTATAAGGCGGAAACCTTTTATCGCATATTAGGAAGGAACAACGGTAGGAAGAAACAATGGTCGATGAGGAACTGTAGGGATGCCAAGTGTGGTGTGCAGATGATGTAATCTAAAGACAGCGTGCTTGTGCAAGAGGATAGCTATGACTTAGTTGATTAGTTTCTTAGTTTCCATGACTATTTTGTTGGTCCTTGGTTTGGCAAAAATATTATTACTTTAATGTGTTCAGTGTTGAACTTTAAATTATTTGATGATTATATACTTTGACATTATCTTTATTAATTGAAATTAGGGATTGCTTATTCT

>ONT.14535.1 LG10 15425459-15432759

TTTTATGCCCCCCTCGTTCAGACAGCTAGAAGGAGATGACCTTGAGCTAGGGTTTATCGGCGGAACTTCTTTTTAGTGACGTGAGAACTGGTAGCTACTAAAACTGCAATTGGGGAAATGCGGACCAGCAGACATGAGGACAGCACAGAGAGCGCCTCTCAAACTTCAGCCGGGCTATATGATATCCTTATTCCTCCAGCTATGGAGGACGCTGCAGTATAGTAGTTTTGCTTTATGAATGCTGGCTGTCTGCAATTCGGCTATGATTTTTGTAAACATCTATGTACAGGAATCTTTTTTCCTTCCTAGTAATAATATAATCCACTTTCAGAGAAAAAATAAAGATTCTGGTAGTCTTTACCATGCCGATTTGAGTAGGTTTTGAAAGCGTAAGAAGTTCTACCAGTAGTACTATCTGGTACCAGTATGAGTGTCGTATGTCCGATATGTATACGTTGGGCTTGTACAAATATCCAGGTATCCTAGGGTGGTAGTTGCTCCTTGTTTGGTGGCAACCTTGTATTTTGATAAGTGTAAAGACAGTGTAATGTCTCGTTATAGTTTGAAGGTTGAGGGCTTACAGAGCATGCATGGGATTTATGATGGTTGAGAACCTTAATTAAATAATTTGGATTTGCCAGA

>ONT.17611.3 LG12 13472268-13473199

ATCTTGGCAGTTTTTCAGAGGCACGCAGGAAAAAGGCAAAAAAAAAATAAAAATAAAAAAAATAAAAATAAAAGAATCTCGATCTCTGGATCTAGGGTTTCTCGATTCCCTCGCTGACTCCCCATCTCGATCCACTGCTTCAATTTTAGGTCTTTAATTCATTTGCTGCAGTTTTTGCTCGTCTTAGCTTTTGGAAGATGATCATCACCCGATCCCAGCGGTCGAGTCGGCACAAACCCACTGCCACGGGGATAGTGCCACGGGGAGCTCGAAGCAGAGGGCGAAGAGGTGGAAGAAGAGAAATGAGAAAAGAGAACCCGCCGGTTACGCTTCAGATATCAACAGGTAAGCCGAGAGTTTAAGAACCTTTTCATGATTTGTAATCTGTTATTGTTATTGCTTTTGGTCAAGATTTGTAATCTGTAGTTAATTGTTTGAGTGTCACAGAATTGGGAACTTGATTTTCTTGGAAACCCTAGATTTATTGGATGTTCAGTCATGTGGAAATAAGTTGATTGTTACTGGTGGCATTATGCAGTGAATGTGTTGGTTTTGGAT

>ONT.1349.1 LG01 91763769-91767417

GGAAACAACATAAGCGCAACTCTCTCTTTCATATCTCTCAACGATGATTGGATTTCTTCTTCAGATCACGCCTTCTCCACGCCAAACTCCTCCCTTTTTCTCTCCTTCTTTCATTTCTCTCAACGACTTTCTTTTGTAGAGGATTTCTTCTTCTTCTTCAATTCTCAAAATCCGAGAGGTTATGAGAAATCTTCACCCTGCAAGAAGAAGAAAGAGAAGAAAACCCTTCCCTCAAAGAGGCAACACCAAGAAGAAGAAGGAAAAAGAAGCAGAGAAGGGGAAGCCTGTCTTTTGAGTGTATCGGTTTCGTTTATTGAGCAATGTCGATATAGATGGTGAGAAATCCCTAAATCTGCCGGAACACCAAATCCCAGCCACCACCTCCGCACTGCCAGTAACCCAACCATCGAAACCCTACACATGTGACCCACTAATAGAAACAGAGTCATAAAAATAGGCTTCTCTATCCGATCTTCCTCTTGCGTTTGGAGATTGGGACTGGAGATCAATGTAATAGAGGCCAATTGCTTGAAGATCTTTTTGTTTTTGATAAGAATATTGTACCTACACTATTTTGGATGCTACATTTTTACTTGAATTAGTAACGATGATATGGCTAAACTTGTGGGTTGGATTTAATAGTTATATGTTTCCTAATATGTTGAAGGAGAAGGTTTTGTACATTTTTTATTAGTTAAATGAGCAGCCTTTATTGTACATTTTATTTTAGTTGAATGAGAAGGTTTTATTGTATA

>ONT.3193.2 LG02 22961740-22973072

GTCTTCCGAAAACCCCGTGGACAATCTCCGAAGCGAAACCTCCTCGCCAAATACGAATCCCCGCTCCTCAATCCGATTCCCAAAGTCCAAAACCCTACACCCTCATCCCTCGATTTTCACTCTTCTGGGGTTTATTGGATCAATAAGTCGTATAGGGCTCCCCCTCGATCCAGACAATGACAGAGATGAGGTTCGCCATTTAGTTTTGCTTCTTTGCCAATTTGTAAATTATATTGAGGTTGCCTGTCTACTACCCCTTGCAGCATGAAAAGGAAGATCCTAAAGTTAAAGCTAATAATGTGTGTAACTTAATGGCCAGTGAGTGACTTGCAGGGGAATCTAATGCATTCTGACATTGGGCTTCCTACGAAGTGATACCATGCTGCACTAAATGGTTTGTTTTGCCAAAAGCCAACTCGGACTCCTGCCCTGTACATAGTCTTCTTGAGGATCGCAAGTTCATTTAAAATGGAAAAGAAGAGGGGAAAAAAAGCTCATGCAGATCAAGCATTTTTAATGCCTCATCCTGGAATATGTAGCTCTGGACATTGAATTATTGTTGTGGTATTCTCTTGGTTGTTCAAGCATCACTCATTTGGGCCACTGCTGTCTCTCCATTTTTTCTCTGGAAGCATTTGTAAAACCCTTTTTCTCATGGTAAATGTAATCACACATTCCAAATG

>ONT.8911.2 LG05 513764-517332

CCGGCATTGGAACGAGTCCGTCCGACTTCTGAACCCTTGGAACGAGTTTGCAGAGCTTCTCTCCATCTCTCGGTCTCTCGTCATCTCCATTTGAGTAGAGCCTCTCACTCTCTTTCTCTCCTCCTCTCTTCATCTCGATCTCAGATGAGTGGGATCAGCTGCGGATTCTAGGGTTCTTCAGGATTCAGTTTGGAGATGGCCCCGGAACAGGTTATGTGTTCTAACCGGTGCCTAGAACTAAAGACGACCTGTTCAACTTGCTACACAACTTTATCCAACATTGGAATACTGAGGACAAATTATTCCGAGAGGCATTGAATGAGATAATGGAAGAATATGCTTTGGTCGATGAACGAGATCACAATGAAGTAGAGGACAGTGTGTTGGTCCTAATGATGCGGATTGGCAATTCTTGACCAATTTGAGAGAACAATTGGCAAAAGATATGTGGGAAGCTAGGGGGAGGGAGTGCTTGATATGGTCTTACTTACATCAATGTTTGTAGTGTTTATATTTATGTTGTCTATTATTGATGATCTTTTGAGAATAGTTAACAATGATTAATGGACTAGTTGTGATGTCTAACGTGTGATATATTATGTTATTTATGTGCTTTATGTATGGAGTTGATTTAT

>ONT.9521.1 LG05 40263657-40275039

GGGAAGAAAAACCCTATCGCCGGCGATGCGATTGCCATCTTTTGGTGTGTTAGCCTAGCCGTCTTTTGCTATTGCCATAATCAGACGGATACGATGCACCTAATCTTCTCTTGTTCAACAGCACCACTGGCAAGAAGCCTAACTGTGGTGGCACTCCAAAAGCGCCCATGTAAAAGAATCTATTTAGCATTTGAAGGCTTGCTACTTGCTAGATTAGGCGTTGCAATGTGAAGATAGGCTCTAATGTGAAGATGGAAACTTATACAAATGGATGCTACAAATGGTTTCTATGATTACTTATGAGTGTGTTCAAAAGGTTGGAGTTGATGGGGTGTCTGCTGTAAAATTTCTCTTATGCAGTTGGAGTTCAGGTTGCCTTCATGTTCTAACTAGTTATTGAAATGGAGTTTCACTTTGAAGCATTGGAGAAGTCAAAGACTGATTATACAGCTTTGAAGCCATTGGCCAAAAATTGATTTTCCACTGTCTAAGGCTAACTAGTGCAGAAGAAAACTACCTTCATTACAAATGGGGAAGGGATTGCTTTTCGATGCTGAGAGTTTCGATGAACAGTTTCGGAAGATTAGTTGTTAAAATTTTCTAAGCAAGAGTTGTTCCTTGCAAAGATAAAAATCTACTCGTCTAAATTTCTTACATTAATTGATTACCTTGATGTGAAAGAAATTTCACTTAATTAATTACCTTATGTAACTTTTGAGTTTCATTTTCAAGTTAAATTAGTATCTTGTCTCAGTTTTTTATGGTT

>ONT.3793.2 LG02 112997318-113048523

CTTTCATCTTTCGCGCGCGCTCTCTCTCTACGGTGGATTCAAAAATATTTTCTTGAGCGGCACGATTTCTAAATCCCGCGAAGAGATTGAGAGAGAAGATGGATAATCGAAGGCATCTTCCTCTGTCGACGACTTTTTTCCTCCCCCATCTACACTCAACTTGGGATCAATATCTTTGGTACCTTCCATCATCACCTCTTCCATTATGATTTAAATGGTCAACGTGGAGCGACAGAACTTATATATGACATTTTTCTAAGGAAAGGAAAATAGTTCCCTGGCCAAAGGAATAAGAAAAAGCACGAGTATGAAGAGGCAAATGTGAAGATCTACGGGCCATCGTCACTGGTGCCTAATCCATCTATCTAGGGTTGGCTTTGTAGGTTCTACTTCAAAATATGTTTCCATCTTTGTTGAAAGGTGGTTAACCTCTCTACATTTGTTTTAAGTTTTCTTATTTTCTCTTTTCTTTTTATCTGCAGCAGGATCAATCAAGTGCTGTTAATTTGTTGTGTATTTGAGCATATGGCGCCTGGCCTTAGAAGAAAGAAAATGTTTATATACAGAAAATGGTGATGTGAATTTGTTAGGGAATAATTTCCACAAATTGCATCCAAACAAGTTATTTATTTTATTTGAGAACC

>ONT.11968.1 LG07 63396023-63396702

CGGGTTTTGCCAGCTCTCTTTATCATTTCTCCCTAATCAGGCCAAAGGTTGAGCCCATTTACTGAATGAATCCTTCTACATATCCAAGTCCACATTAACAGGCCCAATCCTGTCTGCCGGGAACAGGCTACTCTGCAAAATTGATGAAACCCCTCTCTCTCTCTCTCTGCATTTTGTAGTTGGTTACTTGGCTTGGTGTCAGAGAGAAGAGAAGGAAGCCATGGTCAGTGAAGAAGTAGGATCGAAGCTCGTTCGTTGGCTCTCTTTTGTAGGCGCAGGAGTGATTTGCACGGCCGCGATCAATCAATGGAGGGATTACGAGCGAAAGGTGGCCAAGCAAGCGGCAGAAGCGCCCGAAAATCCAACCACGAAATTGGTAAAAGAAGCCGTGGAATCCTAGTTAGGGTTTCCTCCTGATTAAGTAAAATGCTACTGTTGTTATTTCTGTTTCTCATCAATTTCTTTTTCAGTGGTGGTGATTTCTAATGTTGTGAATTTCAGAGCTTGGGTTTTCCTTTTTATGTTTGGAGTTGATTTTCTTGGTATCAGTAAATTGCAACAGACCATCTGAATCTTTTCTTTGGAA

>ONT.712.1 Contig01982 110429-117066

AATCTAATCTTCCGTCGCCAAACCACAAGTAGAGCTGCCGCCCTCTCTCCTCATTCCGTCTCCGACTCTCTCTCCTGCTCCGGCTCCAGCCCTTTCTCCCGTTTCCAGCTCCGGCCTTCTCTCCTCATTCTTGATTTCGCTACTGTAGTGCTGTTACAGAAGAAGCATTAATGTAGTGAATGTAAGGACGGATGAAGTCAACATAAGGACTCAGCGGTATAGGAAAATGCATTTGATGCGCTCCATTCTAAGGTACAATGGTTTTGGATTTGTTTTCCTTAGAAAACATCAGTTTATATCAACCAGTATGACCTCCATGTACCAGAGATGACTGTCAGAGAAACACTAAAATTTTCAGCTCATTGCCAGGGTGTTGGAAGCAAAGCAGGCAATTAGTCTGTCTCCACTTTTGTAGTTATATGCTGTTCAAATCTAACACAAAGTTTATATCTATCTAAAGAACTTCTGATGGAACTGGAGAGGAGAGAGTAAGAGAGAGGAATACTTCCTGATCCTGATCTGGACACCTACATGAAGGCTACATCGGTTGAGGACTCAGCGGCAAGTCTTCAAAGTGACTATATTTGGAAGGTTCTTGGACTGGAGTCTTGCTTGGGAGTGATTTTAGTGTAGGTGATGCTATGAGACGAGGTGTATCAGGTGGAGAGAAGAAACGAGTTACTCAAGGGGAAATGATAGTTCAATCTTTTCGATGAAATTATCATAATGGCAGAAGGGAGGATCATCTACCATGGTCCTTGTGATTGTTTTTGAGTTTTTTTGAAAATTGTGGTTTCAGGTGCCTAGAAAGGAAGAGCCCTGCCAATTTCCTCGCTGAGGTGATTTCGAAGAAAGATCAAGCGCAATACTGGGCTCACTATGGTCAACCATATCATTATGTCTCTGTTGAACAATTTGCAGAATTATTCAAGGAACATTATTTGGGTCAAAAGTTATCAAAAGAGCTTTCACAGCCATGCTCTTTTCTCAGAAGTTGCAAGGTGCTTTATCCTTTACTAAGTACTCTCTGAGTAAAAAAGCAACTTTTGAGAGCTTGCACTAGTAGAGAATGGCTTCTTGTGAAGAGAAATTTGTTTGTATATGTTTTCAAATCGATCTAGGTAAATATCATAAGTGAATAAAAGAATCCTTGTCAAAAGAATCCAGGTTATATGAGCTCTCTTTTTTTCTCCCTCTTCTTGATCATGACCAACAATTATTTGAAATTGATCATGACTGTTTTAAGGCTTCCAGTATTTTACAGACAGAGAGATTCATACTTCTA

>ONT.4737.1 LG02 77632524-77636373

GCTGATGAATGTCTACACTTGTTTGTCCTGGACTCTTTCCTCTCAGTATCGACTCTTTCGCCCAAACACGCAAAAAGGAAAATCCACTTCTTCCTCTCCATGCATCGCCCAAACACAGAAACAAAGCTCTACTTCTTCCTCCCAATACACCCCCCAGATGTAAGAAAAAGCATCACTTCTTGCTCTCAATACAAGGCCCTAAACACAGAAAAGGAAGGCATCACTGGTTGAGTATCATGGAGCATCGGTTTTTTTTCCAACTAATGTGATGGTTTGCCACAAAAGATGTGAGTATTGAATAAGTAAACATGGATGTGGGATTCCTCCTCTAACTGATATCTTTTTCTCTCTCTCTCTCTCTCTCTCTGTTTTTTTTTTTTCTAGAAAATGTGGATTTTACTAGAACTGTTTAGGAGGAAATCACATTAACATTGCATCTTATATTGAAGAACTACATAGTTTGGGATTGGCGTATGGATGTATGAAATGGGTTAGTATATATACTTCTAAAAGAATGTGTACCTTCTTTCTTTGTTTAAAGAGGTTAAAAATGAATCTATACGATTTATGAATTGAGATATC

>ONT.5303.3 LG03 24715340-24716973

GTAGAAAAGAGGTCTCTTCATCTCTCATGGTGTCACCCATCTCCATCTCAATCTCCCTCTCCCTTCTCCGCCTTGGTATCAAAACAAATTCTTGCAAAATGTCTGATGTTTTTCGGTTTGCCAAATTCCCAATGCTTTCCACCTTTCTTTTTCGCTCCAAAACCTTAGAAAAAACCCTAAAGAAAATTTTCTCTGGTTGAAGAAGCTACAACAACTGCAACGGCACACACAAGGCTGCCTTCCCTTCTCAAGCAATCTTGGGTTTTCCAGAGCTAAAGATTGCTTCTTTCATCTATCTTGAATTAATTTGTAGAGACTTGAAGAGATATCACCTGATTCTGCAAATTTCAAATGAGATTGGGAACTGTACTTCTCTGAAACACCTATTTCTGTTTGGGGTTAGTGATTTAAAAAAATTTGTATGTGTTCTTCCTGTTAGACGATGGTGGTCCCATGACCGCACCTCTAATAATCATAGGGCACCATTTTAAGTCAAATAATATGCTTTCCCCTTTTAGTTTTGATTTGATTAAATCTTTCTTGGATTCAATTTAGAAAAGATTAGAAAAGCCTCATCAATCAAGATTCTTGGTTGACCAATCATTGAGGAAACTAAGGAAACTTATTGCTAATTGATTTCTCCGATGTGAAGAAATCTCTCCTGTGTTATTTTATAAAAAGGGTTTTTTGGGCCTCTCTCTTTAACTCATGTTATTTTCAAATTACCCATGGACTCTCACACATGTTGGAGTGAGTGACAAGGGAAATAGGAGAAAATTCAAAAGCTGTTGATCTCACAGCGGGAAAAGTTTTAGATCAATCCATACCGCTCATCATCAAGATCATGGCTCAAGGGGTTTACCAAGTAGCATGCTTTACAGAGACATCGATCCCTTCTGCGACCTCTAAGCTTAAACAACTTGAGGAATTGTAAGGCTTTCAAGAACAATAGGCTAATAGGGGTCTCATTCCTTCAACTCTGTCACATATTCCAAATTTGAAGAGTATGTGAGTTCTCTTGAATTCAAGGATCATCTCTTGGATAATGTTGTAAAGTTGTATCATTCATGAATTCCATCTGGGTCAAGTTTGATAGATATTTTGGAGCTTTTG

>ONT.2481.1 LG01 105562610-105563678

GATCAAGCCTGGAGGCTAGAGGAAGCAGAGCTGCAGGAAAGGGTTGGCGGACAACTACTGAGGAAATCCAAAAATTTGAATCACCCAGTTGGGATTCTGAGGAAGAGGAGAATTGATCTAAGTTGGTTGCTGCTGAAAGGCTTGGAAAGATTCCCTAGAGAGAAGGAGACGAATTCCTGGAGTTGCTGCTGGATAGAAGAAAGAGGAATCTTCTTATCTTCAAAGGACAACCCTGTTTGGAACAAAGGGGGAGGAGATGGGAGGAAAGGGCGGAGTCGTCTTCCAAAAAAAGAAAAAAAAGAAGTTAATAGCCAATGGTATAAAATGAAGATGTAGATTACCACATCTACCGGTCTAACCTAGCTGACCGAAGGAGGGATGAAAGCTAAGTTGGTTTGTTGGCTTCCTTATCAGTAATAGGAAACAATCAAGTTCAATTGGTTGGGTCAAAATTGTACCCCAGCCCTTTATCTGATCCCGTTTTGAGTCTGTAAATTTATCTGATCCCGTTTTGAGTCTGTAAATGTGTGGTCAATGGTTGGACATGAAGGACTGTTTATCAGGTTTTATATGGCCAAAATTAACTTTATTTGATGATT

>ONT.2337.1 LG01 47443532-47444452

AATCTCAACAGATCCCTCCTTTTCCTTCTCCTTTGTCCTTTCCCTTTCTCCAAAGCCAGCCAAGGCGATCATCAGGGAAGACGCATCCCCCCCCCCCATAGAGAGACGGAGGTCTCCATCTCACCACTGCCAAGAGATTCTCCATCTTCCTTCATCTCTCCCTCTCTCCACAGCTACGCCAGATAATCCAAAATGGGAAGACGCTGCAATCCCCTCCCAGCCTAATATCTCCCTCCCATAGGGAGACTCATCTCTCCCTCTCGCCACTGCTAAAAGGGAAAATCCATGAGCCCTGCCGCTCTCCTTCGTATCTCCCTCTCTCTATAGCGATCTGAGCTAGCCCAGCTACGTCTGCCATCGTACAATCGATCAGCCACGAGCTGGCCAGCCATTGCTACATCTCTCCTGCTTGAAGACTATCAACCTAGTTGAAAGTGCAGATGTTGAAACCTGAGTGTTCATACTATTCATGTGGATTGGGGATGTTTGAAAAAGAGTAAGTTGCATGTACTGCAAATTTTACAGGTTTACAGTTCTTTCATGCCACGCATTGCTGTCTTCTCTTTCGCATGGGATCAAAGGATTGGAGAAAGCTTCAAGCAATATTCAAAAATTGTAGTCACTGAAAATTGTATTCACAGTTGTTAAATTGTATTCAGAGATTTTTCTAGAAAAAATGTATTCTCTCAAATTTTTATCGAGGAATCTATTCAGAAATGTATATTTATAGTCACATTTGTACTCAGATTTTAATTAGAAATGTCATGAGAATTTTTTATTCTCTCAA

>ONT.14215.4 LG10 488222-492883

CAGGCGAAGTTCCTCATCCTTCACTCCTCCATTCTCTTTCTCAAATCTTCAGAACTAGGACTTGCACGTGTGTTGAGAGTGTAGATTGGGACTTCCTTATCATCTTCCCTGACTTTCAAAACAATCGTGGAGCAAGAGTCTTCTTCATCAACTATCTATTTCATATAAAAGGGGTCTGGATTTTTGGGGGTTTTCTCTTACATTTGAGCCAAGTTAGTGAAAGAGGGTTGTAAAGCTCTATTGAGGCAAGTTGTTGACAGACACGTTGTGATTGGTGCCTATACACGGGAGTCCAATGGCCACTGTTGGGGTTGCAAAAGACTGGCTCAAGTATTTGAATAGATCTGTATTTGAAGGGCGAGCTATGTTGAGAAGGTTATCTGTGTGTCGAGAAGCTCTTCATTGAGCTTCAAAAGCCTTATCACAACCACCTAAACAAGAAAATCACTCTTTCAAGCAAATATCAGACTATTTGACCAAAGGGTTTCGAGGAGGCAAAGAGGACGGACACCAACACCTGGCTGGTACTGTGGCCTGATAACGATCCCTGCTATGGGATCACACACCTATAACCCTAGTAGAACTTTGGGAAGTTGCTTTTTGGCAGCCACTCCGGAGTCCATCAAATTACTCTCCTTGTGGCAGGATACATTATTATTATCCCTGCTTCTATGAAAGGGATCAATCCTACTCTCTGTAATACAGCAAGTGCAGTGTTTCTCCAAGAGTGAGAAGAAGCTTGAGGAGGAGCTTGTCTTGCAGCATTTCTCTGAGGCCAAGGGAGCAATAGCTTTTCTCACAATCTTTCTTCCAGTTTCTAGAGCTATTCTCAGAATTCAGTGTATTTCTCCCTGACCGAGGAGGTGTTATCACAGAGCATTTCTCATAGACCGAGGAGGAGCCCTTGGAGCACGGTTTCTGCTAGGCTGGAGGAGTCATTCCAGCAGCTGCAGTCATAGTGTGAGCTTGGGTTCTCGACCTCTATGGCTCTATCACTTGTATTCACCTTAAGGTTCAGCCATCTAGCAGTGGATATTCGAGGTTTCCTTTGCCTTGTGGTGGATTAATGATCATTGAATGCCAACTTTGGTCACCGTTCTGAATGC

>ONT.5010.2 LG02 116690998-116695135

TTCTAGGGTTCCTCGTTGCCTTTTCTTTCTTGTTTCTGTTTTTCTTAGATCTGCTCGTTTTCTTTCGTCTTTCGGCTGGTATGCAGTCTATTTCGTATTGATCTATCGCCGATCTACCTATTGATAGTCGATTTTCCTTCTGATTTGCACTTAGCCCTTTGTTTATTGGTTGTTTTGCAGCAGATCTAAGGTATTTCAACGTTTCGATCCTCTTCTTCTTTGTTTCTTCTTGGTTTTCGCGGTTTCTTTGCCTAATCTTCTTTGATTTTTCTTGAAATCGTATGGTAAACGAGGTCTAGATTTGTCATACCTTTCTTTTTACTGTTTGTGTTTGTCGATCTTCCTTTCGTTTTCAGATTTGAGGTAGGCTTGTTCTTTTTGTTGTAACTTAGGGTCTGGTGCTTGCGATTGCAATATTTCTTCTTTTTTATGGTTGTTTTGAAGCAGTCTGACGGATCAAGACTCATAAGGCGATGGAGGGGATTCCAGTTTCGCAGATCGTAGTCTCTTATAGCAGTAAAGAGACTCCTTTCGCAAGATCCGTTGCATTTTGGCGTTCCTCGTCGACGATTTCATCGGATCCATGGGTTTTTTTAGCGATCTTTCATCAGATCTACTATGTATGGATGTAAAGAAAAGTAGTTTAGTGTGAAGATCGAGGAAAAGCTTGTTATGTATGAAGCTGATAGAGAAATAGGTATGGATTAAGCGAGAGAGAGGCTTGTGGCAATGGATCTATGTCATCCTCCGACCTTTGCCATGTTGGGTGCGCTCGCATGGCAGGTCAAAAAAGCCTAATAAAATTCTCGATTTTTGGTTCTCGGGGGAGTTCGCATGGGCACTTTCGCCTGGGTTCTCCCCCTCCTGTGTGTCCTCTCTTTCTTTTCGTTTCTCCATCTCTTTCTTTCTGTGCGTTTGATTAGCAATATCTGCAACTCGACATCACGCTTGCGAAACCGAGCAGAAGAAAGCTACGATGTGGAAATCTCTCGTTATATCATGGCAACATCACAAAGGTCGGCGGCTTTTTACCACATGGTGGTGCAGTGGAGTTTGCGCGCTATCTATTCAAGCTTTGGAGTTCGTCTTTTGTTCTGGACTGTTTTTATCATGCGTTTCCAGAGTTGTTGCTGAATATTATTCCAGACTTCTTTTCTCTGATTTCACGGAATTATGGTGGGCTGATTGGTAATCATACTTCTCATAGAACTGCAATCTGCTTTTAACTACAAATTTTATTTTTACATGTAGCGTTCATTCGTTTATATTCATATTATGCACTGGTATCTTTTTTCTTTATAGTGTTTCTCAAGGGTGGTTGTGGTTGTGGTTGTATATATTTTTTTTTCATGTGGTAGAAGAAAATGAAGTTATATGTATATTAATCATTTTTTCCTTGTGCTGGGGGAATATGTTTTTTTAGAAAAGATATATACATGTAAAAACCAGGCAAGGAGTGAAATGGAGGCAAGGAGTGAAATGGGAAGGTTTAATGTACTGCTGTCGAAGATCTTATTGCTGTACCTTTTTTCCTGTTTTGTTGTATTTTTGAAACTCTGCGTCGGGTTATTATTATATTCCTTAATCTGACC

>ONT.2728.1 LG01 121435936-121437527

GTTCGAATCCTCTCCTTTCACGAAAGAAGCTGCCGGATCTGCTTAGATTCTCCCATTCCTTCTCTTCTCCAGCTCGCTACATCTGCACCTTTAGCATTCTCTGAAACCCTAGCGTTTTCAGAGCCATGATTTGTACAATATCCGGCGAGGCGCCGGAAGATCCCATGATTTCGAACAAATCTCGGCTTCTGTTCGTGTTCGAGAGGATCATGATAAATGCCCTGTTACTGGAGAGCAGCTTACAATGGATGATATTGTAACAATAAAGACAAATAAGTCATTTTTTCCCTTGCAGGCAGTCAAACCCAGACCATTACAAGCTGCAAGCATCCCTTGGTTGCCTGGAATGTTTCAGAATGTAAGCTAACAGTCAAGGCTACTGGTTTATTATTTTGATTTTATCTGCTTTCATTTTTCATGGAAATCATGGTTAGAATTTCAAATCTTCAGTTTTTCACATCAAATCTTTTGCTCCTATGAATATTTGGTATTTGTGCCCATTCCTTGTTTACATTGGTTATGAATTCGAGATTATCATT

>ONT.18024.1 LG12 53934182-53955584

CCCCGCCCTCTCTCGCACGCCCTACTCTCTCTCTCAGTCTCTTTCTCCCGCTCCTCTCTTTCTCTACATCTCTCTATTTCTAATCTCTTTCTTCCTCACTCTCTTCCCCTCTCAATCTCTTTCTTGGTCTCCGCAAAGAGATCTCAGCTCTCACTGCTCACTGCCTGTCACTGCTCGCGGCCTCTGATGTCCAAGGATATGAGCTTGAGGAGACAATCAAATCCTTAACGATCAAGTCATCCATGGTTCAAGAGGAGAGAAGAGAGAGAGTTGTTATGGGGTCCCGCGGACCATGGAAGTCTCATCAGGGCTGCGGAGGAGTATCTCAAATGCACTGGATGTCATCTCTTAGCAGAAGATGGGAGTGACCATTGGCCTCCTCCTTTTTCTTCAAAGATTCAACTTTTTTTCATAGATTATCATTCCTCAAATACAGGGGCAGGAGCAGGAGGATATGGGTAATATGTCATCAAGCCAAGAGCGACATCAAGGACTTCATCATGAATGCTGATTTCCTTCTTTTGCAGTCGCCTTCTATGTTTTTGACTTACATAGTTTATGCTCATGTAGACTCAATGACTGTCTTGTAGTCCTTTATGTGCTCAAGCTCTTCTGGTTAATATAATCGACATCTTTTCATCAAAAAAAAAA

>ONT.7059.5 LG04 86525875-86531270

GCCCAGCAATCCGTCACCTCTTTGACCCTTTCTCGCCTGCCATCCCTTCCAGCAGCTCTTTCCACCGATTCCTCCAGCGGCTCTTTCCAATCCTCCTCCTCCATAATCACGCCTTCTTGAGCTGTTTTTGAGAGCAGAGTGGTATTCAGCAGTTTCTTCCTCCCTGTGCTAGTGGTGTGGCAGTTTGGAGTCTGGTTCATTACCATGAAAACAGAGTTGCCACTCATCCCTTCTATATTTTCCCTATTACCTTTCCTATCTCTTCTCTTTCCTTTCATCTGAATCACTTCTAATTTTATTTTATTTTTTCTGTTTGGGCTTGGGTGACTCCACTCTTAGGGTGACTCAACTCGGGCCAATTTTCAAAACACGGAGCTAACAGACAGCAGATTGAATAAAAATTGAAAAACAACTTCTGTTGAAAGAGTCCTTGTTGCTAGGATGCAACCAACATAAAGACCAAGTATGCAGAGTAGAATGTTTGGCATTTGAATGTTGGTATAGAGTAGAATCCTTTTGTGGTGAATGGCCACTTCTCTTACCCGGTGATTCTCCACTGTGTACTAGAAGATAAATCTGATTGGGTTGACAAACCATTGTGTACTAGATATATGCTTTCTGCTTGTGTATGTTGGCTGCCCACCACTTTAGAAATCCTGCTGTGACTTTTCTCTTATCATGCAACAGTATTGTTGAGTTGTAAACTGCATCATGTTTCTGGCCTAATTTTCAGTTTATTGACCGGTTGTTTGAAGGCTTTC

>ONT.6297.3 LG03 58654782-58660164

GACGATGGAGAGATGGCAAAAGAAAACGATGGCAGCAAGAAGGGGCGGGAGTGCTCCTCTTGGTGCCGATGAAGACGACGCTGCTGGTAGTCGACGATGATGATGCTTGTTGGTGCACGGTATCCCACTGATGCGGACAAGCAAATGTTGGATAAACAAACAATTCTTATTATGAACCAGTAAACATTGAGTGAAGCATGCACTCAGCGGTATTTAATTCTGCAATGGAAGCCAACACGAGTGAGAACGTGCATCATGCAATTATAATGTCATTTATGTTTGAAGACTGAAGGTTATTTATGTTTGAATATTGAAGTGGGGTTTTCTTTCTTCTTTCCAATCTCCATCAAAGGCAGTAACGGAGCTTCTCAGATCTCTTCCTCTTGAAGCTGAATCTTCTCCTTCTTCTCTCACTGAGGCTCTGATGCTTTTTTGTGTGGATTTCTCCCTTTCCCTCAGTCGGTTTTTCTTCCTCGATTATTGAAGATAGCAAAGTGCAGAGAAAAGATTCTGAATTACTTGCTCTTTCTGAGGATAACTCCAACTTTGCCTGACACATTTATAAATATGGTATCTCCCATTGTAGACATACCATTTCATGTCTTCCTTGCAACCTTGGTTGGTCTTATGCCAGCGTCTTATATCACTGTCAAAGTACGTGTTTTGTCCTTGCCACATTTTCTATCTACTTACTCCTGATTTATTTCATATGTTTGGTTTTTGTATCCTAATTGGCATTCACA

>ONT.6795.1 LG04 57437336-57439455

ATTTCATCTCAACAACCAACACAGCAACCCACTCATCTCTTCTTCTCTTCCTTCCTTTCATTTCTTCTTCTCCGTTGACAAGCACCGGAGCTCCGGCGAAGAAAACCAAGCCAGCCATCATCTTCCTCAAGCCTTCAAGAGTCCAACCATACACTTTTCTAAGTGTTTCACCCACAACCCACTTAAAGGAGCATATTGGGTTTGGAGAGTTGTCTCAACCTTTGGAGCTTCTTAGAGTTGTGTTTCTCCAAGTTTCTTCTTGATTTGAGTCTAGCACATCATAAAGGTACCGAGTATTTCTACCCCGAGGCAGAGGAGGCGACGGAAGAGGTTCAGGCAGATCAGCCTGATGACTTCCTAGAGTATAGTACGGATGGCATTGTATAGCCGTGACAACCCCGTACATGAGAGCTTCATGTTTATCCTCCTAGCTTCATCTTTTGGTTTTCTTATGCTTTTGTTTAGGCCTAAGTTTAGTTTGGCCTTTTTACTTGTAATTAATCTTTTGTTAACAGTCTTAAACTTTAACCCTAATTATGTTATTCAGTA

>ONT.7800.1 LG04 87966457-87967496

GGCAGTTTCTAACTTCTCAATTACAACTTCAAGACCACTTCAACTTAAGCTGATTTTCCATGGACATTCAATTTAGTCTTGGGAGCTCTGATAAGTCACCACCGCCCCAGCCTAATAACACGACCAAAAATGTCCAGACTGGTGGAGGCAAGTACAACCCACAAGTCCAAGGGGGGAACAAAGCAGATGGAGGTGGCAACATCAATATGGGACCTCAAATATCTGATGGTGGTAACTTTACTATTAATTGAAAAAATATCTTCCCCCTCTAAATGAGGATGTTGAAGCTACTACCATCCGTGCTCAAGTCTTTAAGCTTTGTCTACTTTCAACAAAGTCAGGGTCCTCCAGTTACTGAAAGCGCCTCCGGCCTCCCAAAGTCACAAATAATACTGGAAGTAATGTTAGCCTCTGTTTACACAAAATAAATAAAAAATAAAAAAATAGTTATCTGCATCGTTTCTGTATTTTTGTGGATCTCAAGTAGTTATTGTGTAATGCTTGCTTGCTGGTAAGTCAAAGTGGTGTTATATATATATAAGCCATATAACAGCTAATCAGCTAATCCCTC

>ONT.2005.1 LG01 13410801-13416142

GAAGTTAGACTTCTTTTATACTTTTTGTAACTTGTCGAGTTACGCTTGCGTGCCTCGTCTCTCCGCCTTTTAGGTCTCAGGTCTCATAGAGATTTCTAGGGTTCTTGTTTGTTCGATCTTCGAGGTAGAAATGGCAGCTAAATCGACACTCTTCAACTTCCTTCGCCCGAGTCTCCGCCCCCAATCGACTGATATCTCTGCTGCTGTTATGTGGGGCGTCGCCGCCACCACCACCGCTATCTGGCTCATTCAGCCATTTGATTGGTTGAAGAAAACGTTTATTGAGAAGCCTGAGCCTGAAAACTAGACAAAAATGAGTTCTTTCACAAGTTTCTGATGAAGGGAAGAGCACAAACCGGGATCTTTTGAATAATGCAGTTTCCTTATTTTTCCTTCCTCCCTTCGGGAAAGATGTCCATTTCTTTCTGGTAGTTTATGCCTTTTTGGATTGATTGCTAGACAATGGAGTTGAGAAAATTTTACTGGAACCATGCTTGTGGACTACTTTTGCTAATAATGTCTGGTTTGTTGATGAAGTTGCTGTGACCATTCCATAAACCTAAGGTCTGTTTGATGCCAGGCCCACAAGTGACTTCCCTGGAACTGAGAGGTGCAACTTGTGACCCATAAATTCATTTGATAATTTGTAAGGATTCTCGAGTCTGAGAGCTCCTCTTGAAACTCTGAGTTTATGAACTTGGTAGGGATTCTGCC

>ONT.11411.2 LG07 67336480-67343673

CCCCCAAAAGAAAAAAAATCTATCATCTAGGGTTTTCTCTCGAGCTCTTCGTCTTTCGTTTCAGGGAAGGAGGAAATGTTACCCATTCCTGAAGGTATTCAATCAAATGATGCTTTATTACCAAAGGATATATAGGCAATTCCATTCAGACCGCTCTCTCAAAGACTCAGATGATTGCCTGATGACTGAATGATGAAGATGAAGTGAGAGAGGAAGGAGGAGAGAGTGACTTGTGGGATTGAATCTTTATGAGAGACATCCAGAGGGCGGGCCATGCACCGTGCAGAAGGTGTTGAGTCCCACCAAAGTTTTCCAACGACAGTTCTTTATGCAATAAAACAATGTGTCCACAAAATGAGCATGTGCTCTAGCAAACTGAAGCTTTCAGTTGGGAGGTTCCCATGGTCCTCATGTTCATGGATTATAAACATCAACTTTGCCGGTTAATTGACACCAAAAGAGTTTATAAAAACAGCTCTTCCATCACAACTGAGGAAGATCTCCATGAGGAAAAAAAGACGACTGACATTTCTTCAGCTTGGGCATAGGGATAGTGGCAGCTTCTTGAGTTAGAGCAACTAAAGCCCCGGACATATCAACATCTTGGAGTTCACCCTTTTCTTGCATGTACTCAAGAGGTAGTAAAATTAGATCAAAGACGTGCAACTTATCCAAGGATTTGATTCAAGATAGATCGGAATGGAGAAACAAAATTCATGTAGCCGACCCCAACATAGGTGGGACAAGTCTTTGATGATGGTGATGATGAAATAACTCTGAGGAAGTAGATCGTAGCGGCGTCATTATAGCGTGTAAAAGATTATGGGCATAATTTTGGAACTGTTATTATTGTTTTTATATTTTTAATTTGAATGGAATTTTGAAGTGAAGATTTTAGTTTTCCTCCTAGGGATTACCTTGTTTATTTCGATCACAGACATTTGTGGATGTTAAAGATTTTGGAAAATGATCAAATAATAATATTTGTGTATTTTAGAGATTTATGGGTT

>ONT.11930.1 LG07 53972183-53997982

CGTGCGCAGAAGGTGAAAAGGGAATTTTCAAACCTGTTCGTTGTTGTTTGAAGATGAAGGGGAGATGGAAGTTGAAAAGGCGCATGTGACTTGCTGGCGACGCTGGCAGGCGACGAGTTGCTGATGACGATGGTGAAAGTGCTGCGGAGCGGGCTGGAGGTCCAGACCCGTTGTCGATTATCTAGAGGAGGAGCCGGAGGAGGCGGTAGGTGCTCCGCAACCTCTAGTTGAGTGCCGTGTTGACGATGTCATGTAGACATCGGGTTACTACGAGAGGCGTAACATTTTGAGGAGAACTATTTAACCATTCTTTTGGGTTATTGTTTTGTTTATATTGGCCTTTGTTGGCCTCTCTTTTTGGGAATGTAACTAAATGATCTTTTGTTAAAACTTTAACTTACTTTTGGGAAACATGCTATAGATCATTGCTCGTTCTGATATAGTATTTCATACTTATGTTATTGCCTTCGTGACT

>ONT.9345.1 LG05 26065338-26067473

GCATGCTGCTGGCTGGGAGAGAAAGTGTAGAGAATTTCTTTCTCTCTTCTTCTTTTTCAAATTCTCTCTAGTTCTTCCATCACTCTAGCCATATACAAGTGAAAGTCTTCTTCTTCATCCACTTCTTCATCTTTTCCACCCTTGTTTCAAGAGACCATTTGAGCTCCAACTCCTAATTTTGGGGTTCTTTGTCAAGACACAGATACAACCCCCGGTCCTGAAGAGGTCCAGGAGGGAAAGGAGAGCACCCAGGAGATATATTCAGGATTAGAAGCGTGGTCAGAACCAGGGCCCGTCCAAAAGTCATCTTCTCGGAGTGCGAGCTACATTTGTAGATATCATATTTCGTATGATTCCTCTTTCATGTGGTTATACGAAATTCTTTTGGAGATATTTTTGGTAGAAATTGTATAGGGTTCGTTCATTTGCACCTCTTTTGTAACTTAACGTATATGTTTGTTTACATTTTGTATATCAAACTTTAAATGTTTAATTAGTACTTGATGGAATTTGATACCT

>ONT.10219.1 LG06 56438870-56444222

CGTTAAAAATTTCATCGCGCCTTTTGTTTTCACAAATGTTCCCTGCGCGCGGTAATTCACCCCTCCCCATTTCTTCTTGCGCCATTGGAGACCTCCACCATTTCTCCAATCGCATTCTCTCGTCGCCATTTTCTAAGGTTTTCGTTTCGTTTTGTGGATCTCTCACCTCCTCTACTCGCAATCCATCTCCTCCATTCCTTTATTCTTTCTCCATTCTTGCTCCTGTTTCAGAATCCCAAACGAGAGAGAAGGTGCGGAGGAGAAATCTAGAGAAGAAAAGATCGAGAAGAAATCTGGAAAAGAAGACGAAGCTAGTCAGAAGCTTCCAAAGGAGTTTGATGTGCGGCATTTTTTTCAAGAAAGGATATTCTATCCCTAGTTCTCGTCGTTGACAAGATCTCAACTGCATTCTGGAAAGAGGATCTTAAGAAGGTAAAGGTATTAGCTGGGGCACCCCAATAGATCCTCCGTTATATGACGATCCGAGCAACGCTCTTCCTGGCGATGGAATCCGACCCGAGATTGCAGAATCTGTAAAACAGATTGAATGAAAATTTAAAAACAAATATTGTTGAAAGTGTCTATGCAGCTAGGATGCAGCCAATATGAAGGACAAGTATGCAGAATAACAGATTGCTAAATATTAAAATGGGATGCATGAAGTTTTTTAAAACAATGGTTCAAGTGTTGGAATGGTGCAACAATGAAATCATCATATATGAATATGTAAATTTTCATTGGTATTTGAATATTGGTCCAGAGTAGAATGTTACCATTTGTTTTAATTTTGTAATGGTAATGGTAAAGTGTTTTCAATGTTGAAAGGATGTTTGAATATTAATATGAAGTGGAATGAATTTTCTTGTTT

>ONT.4637.3 LG02 40211366-40212967

CTAATCAAACCCGTCCCTTCTCTCTCTCTCCTTCATCCTTCTCCTCGACCGCACCAGACCACAGAATTTAGCGCATCACATCGATATGGGGTCTGGGGTTTGCAAGGAATGACTTCTCCAATTTACCAAATTTACCAAACAAACGTTGTGGAGATGCTTTCTTTTGTTGCTACTTGTGGTGTATTAAAGTGAGGTTTCTATTACTGGATTGAGTTTGTGAGGGGCGGTTTGCTTCATGTTACTCCTTAATAAAGCTGCAAAACAGCCAAGGGAGAAAAGGTGAGGTTTCTATCAATCTTTCATTTTTACCCTCTCCATGTGAACTGTGAAAACTTGGTAAAGTAGGTAAGATTAGAGATCACTGTCTTCATAAGTGTTATCCTACCTCTACAGTGAACAGTATTGCCGCTTGCCCGACGGGAGGGGTTTTACTTTTGGAGGAAGTCGCAAATAATTTGGCATCGCAAAATAGGATCATATTGTTTGCAAATTATGTAGATGGTGATTTCAAAAATCTCACTACCTGGTTGGGATGGTCGTACTCTTTTGCAATTCTTTTGTCCATATGGAAGGGGAGACGATAGGATTTTTAGAGAAGCTTCATCTTGTAAGAATGTGTTTGTTCTTTGCGGTTGACCTAAGGCTAGTGAACGGGCCCGCTTTTAAGGAAGGGGTTCTGTAACTTAAAGCTCGATAATATTCTTCATGACTGGGAAGTTTGTATGGAGTCTAGGAGTTTAAAAGAATGGTAGTTGGTCAGTTGGTCACCCCCTCGCAAAGGGCCTTTGAAATATAATGTTGATGGAGCAGTTAAAGGCAGGCCAGGGCTGGCAACTGGCAAGTATAGGTACTGGAAGGAGATGCATTGTCCATGTTTTCTAAGCATGTGGGAGTGAAGGACTCTAATGAGGTTGATGTGCTTGCCATTGTGGCCCTAAGGATTTATTTCTGCAGCTGCCTCGACAGGTTTATTATGGAGTAACTCTTCCAATGCCATATTATGGAGTAACTCCCTAGGGTATGACACTATATGGCTTTGATTCAAGTGGCTTTTCAACATGTTAGTAGATTTGCTAATGGGATGGCAGGCTCGCTAGCCAAGCAAGGGCTGGGTAGTACTGTTGGTTCAGTGCTCCTATCGTATAATATTGGCATTTTGGGCGTTCAACATTTAGTATGATGTGCTTGTACTGACACTCTCTTGCTTTCACCATTCTCTTTTGTCTGTACTTGTGCTCAATTTTAATAATTTTTTTTCACTGATAAAAAAGAAATCATGCCATGTGGAAA

>ONT.653.1 Contig01862_ERROPOS1000000_A 170417-179829

GGATATCTCTTCTCACCGAAAACCAGAGGCGACTCGTATCCCCTAACTACAAGAAAATAAGACGCTATAAGACGATATTACCACTCAAATACGGCGATCGCAGGCCTCTAATGCAAATGCTATTAAGACGATATTGCCGTCAGCTCTCTCCCTGCTCATGCATCGTTCTCTTTGCTCGACGCCGAAAGAGATCCCTCTCTTCCTCATCATCGATCCCTCTCTTGCTCTCTCCCTACTCACGCCATCCCTCTCTGCTCTATCCCTGCTCACGCATCCCTTTCTTCCTCATCGATTTTCCTCATCGAGTAAATACTCCTCCTCGCTGGATATAAAGCAACGAAGATGGCGCCCCCCACCCCACAACAAAAAATCTATCATCTAGGGTTTTCTCTCGAGCTCTTCGTCTTTCGTTTCAGGGAAGGAGGAAATGTTACCCATTCCTGAAGGTATTCAATCAAATGATGCTTTATTACCAAAGGATATAGGCAATTCCATTCAGACCGCTCTCTCAAAGACGACTGACATTCTTCAGCTTGGGCATAGGGATAGTGGCAGCTTCTTGAGTTAGAGCAACTAAAGCCCCGGACATATCAACATCTTGGAGTTCACCCTTTTCTTGCATGTACTCAAGAGGTAGTAAAATTAGATCAAAGACGTGCAACTTATCCAAGGATTTGACTCAAGATAGATTGGAATGGAGAAACAAAATTCATGTAGCCGACCCCAACATAGGTGGGACAAGTCTTTGATGATGGTGATGATGAAATAACTCTGAGGAAGTAGATCGTAGTTGCGTCATTGTAGCGTGTAAAAGATTATGGGCATAATTTTGGAACTGTTGTTATTGTTTTTATATTTTTAATTTGAATGGAATTTTGAAGTGAAGATTTTATAGTTTTCCTCCCAGGGATTACTTTGTTTATTTCGATCACAGACATTTGTGGATGTTAAAGATTTTAGGAAATGATCAAATATAATATTTGTGTATTTTAGAG

>ONT.5143.1 LG02 125349461-125361457

GCACAACACAAGATCAGGAAATCACCACCCATCAGCTGAAGAATCTCAAGTCTTTTATCTCGTTCAATCTTCCTACTGCTGGGCATATATCAGAATGTGCAGAAAATGAGAATTACAGACCTCTGTCACGGAGCCCACTTTCAAGCACATTGTCATCCTGGGCCCTGCATTGCTGAATTGAAGTAAATTGAAGAATTCACTATATGGGTCCACACACAAGCCATATGCAAATAAATCAAAGGAGGGGTCCACTCATCATGTCACTTGAAGAATCCTAACTACAGTAGAAGACTTCTCATAGGGCCCACTACTCAAACAGATGAAATGTCTCACATGCAGGGCAGGGTCCACAGTGGTTGCAAATTTGAAATTATGAATGGATAATTCAAAGAGTATCATTCTGATTAGAACAAAAGAAGAAGAGTTTGAATCATCCACATCTGATGTCCTTTCAAAAAAAAGAAAGTTGTGCAGAGATAGAATCCATTGTAAATCAAATTCTAGAAGTTAATCTCGTTTTTCCCAGGACAACTAATCTTCAAATGCTCATAACTTTCAATCCGACCGTCGGATCGATGAAATTTACCTACCGTTGGAAAGCTTATTCAGAGTAGATATCCGTGGAGCCTAAGAACGTGCAAATCAGAGCTATATTGACAAAGTTATGAGTCCGGAGATTTTCATGTTCATTTTTAGACGGGATTGCAAATTAAATCCACACTATTTGCCAAAGCTTTGCTACAAATTCAATTTCAGATTTGAACTTATCAAAGAGAGTGGGCCATAGCATGGGAAATTTCCAAGTGAAGAGAATTTAAGAGGAAGTCCTTGTTGGCTAGGGAATCTAACTAAGCAAGTCTTGTCCAAAAAGTTAACATCTCTCCAAAAGTCACTAAGAAACTCACCTTAACTTGGAAAGCAAGAAGAAGACTTATCAAAGAAGGAAGATTCTTTTGCTAAAAAAGAGGATCTAGAGAGAAAGAATAGGGAGCTCATACCCTATTTAAAGAAGGCTTTGGCTCTCAAAGAGAGAAACTCCTCCCCTTGGATGTCCATATCACTCTCTCTCCTCTTTCTCTCCTTTCTCTAATTAATAGAATTTAATTCTTATTTTTGGGTATTCTGGAGCCCTGTTCCAGAAATTAGGATTTATAGAAAAGAGATTTCTGAGAGATCATTGTACAAGGAGATGCTATCAATGAAATTACTTATGGAGTTCTTTT

>ONT.629.7 Contig01814 58986-61530

GCTCTTCAGCCGCTTCTGCTCTGCCCCAGGTCAGGAAGCGAGAAAGACAAAAAAAAAACCTCTTCGGCTCTTCTGCTCTTCTGCTCTTCCCTCTTCCCTTTTCCGCTCTTTCGCTCTTCCGAGTTCCGACCTACCCTCTTCCTTCGAGGATCGACGGTTAAGGTGCTCTCATTTGGATACATATGATGAAGGAACTATTTGGAGCATAAGGAAATAATAACGAATTTTTACATGATAATGTGTATTACTTCGTTAGAGTTTCTTTATTATTGTTGTAGTTTGCTTGTCAAATTGTGTGCTAATTTTTTGAAGGTTGTGAACCCTTTCTTTTTTAGGATGATTGGTTTGCCTTCATTTTTTGAGAGATTTTTTTGTTTTCAGTATACTTCTCTTGTACTGTAAATTTTGGAGAAATGAATAGTCATCATTTCAAACAAGAAATATATTAAGTTGATTTAGTTAAA

>ONT.4665.3 LG02 44901797-44904112

GGTTTCAATAACAACAACATTTTTCTCTCTATCTATCATCGTTTACTGCTTTCCAGGGCCGCCTTCTCTTCGAGGACGGCTTTTACTATTTATACACTCTGTATGTAGTTTGACATGCTTATATAGCATAATAGAAGAAACAGGAAAACAACAGGACAAACATGGATTTTACTGTAGCATGGAAAAGTGTTTCTACTTTCCACGCTTTTTGGCACAGCCTGGATCTGTGATCGATGCGTTAGATCAACCTCCTGAGGGATTGATAAAATCCAAGATGGATTCCATCCACTCGAGCACAATTGCAACAATGAAACACTCCATAAACTGAAGAAGCTCAGAAATCTTGATTAATTGAATAAGCATTCTTAATACAGTGTGGCCTTCTCATCCTTATATAGAGGAGTAGGCTTGATGCCTAAGCAACAATACTTTCTAATATCTGTATAATGCTAAAATAGGAAACTAACTAGTATTAATCCTAATCAAATCAAACTCCTAAATAAAGGAATGCCCCCTTGGGCCTATAGACTGGACCCAATGGGTTGGCCGACCTCAGAGGCGGACCCAGTGTATAAGCGCCCCAGGTCAAAATCCACTTTTAGTATTGCCTTGCCCCACATCAAACAGATGTCATGAAAGGAAAAC

>ONT.7617.2 LG04 71809538-71811075

AAAAGAGAGATCGATTCTTCTCCTATTTTCCTATTTCCTTCTTCCATCGGTTTCTGCCCACTTTCACCAGCGTGCATCGGAGCACCGACGACCACCCGTACACCGGCGACCACTGACAACCAATGGTGAAAATGGTTGTGACAAGCTTATTTGGAAGGATGAGGTATTCATGGGAGAGATCGAAGATGCAAATTCCGCATTTAATGTATCTAAGAAATTTGTCATTTGTCAACGAAGGTGGAGCACTATAAATCAAGATTGAAGTTGGTAGATACAGAGGTGGAGCATATGAACACATCTTTGTTCAGCCTGGAAATTGTAGAGACGCTTCGCAAACTTTTTCATTTTGTTCAAATTATGTAATGTATTATCTTTGTAACCTAGCTATATGTTTTTTTTAACATAGGCATGTGATCATGTGTGGAACATATAAGTGATACCGAATGATCAATGAATGTAAACTTCTTGATTATAATGAAATCTAATCATATTTATGT

>ONT.7076.1 LG04 87759635-87760958

GGACAGGAGTGGGTCGCTCGTGTGGGATTAGAACAAAGGGAACATTTTGTTTCGGTCTTTCGCCAAAAAATTTCATTTCCCCCTTTCAGACCCCCAAACGCTCCTCTTCTTCTCTATCTCTCGCTGCTGCGCTTCTCATCCCTGCGACCCATCTCGCCCCGTCTTCTCCATCTCTCGCTGTTGCGCTTCTCTTCCCTGCTCTCTCTCTCTCTCTCCCATCTCGCGATCTCTCTCTCCCCCTCTCGCTTGCGATCTCTCTCTCGCTGCTCCGCTGCGTCACCTCTCAGCTCTGCTATTGCGCTGCGATCCCCTATCTCTCTGGTGCTGCGCTGTGATCCCTGCTTCTCCGGTTAGTGCTGCGATCTCTGTTGCGCGACCCCTCTCTCGCTCTCTCCCCCTCTCTGCTGTGATCGCTCCCTCATCTCTCTGCTCCTCTCCCCCTCTTTGCTGTGATTCTCCCATCTCTTATTTGATTTGAAAGGATAATTCATGCAATGCACACAAGTGGAGATCTCCAAACGACCAAGCAAGCTTGCCGTGATTGACTCCCTTTCTTGTGGTGAGGACATGTTGGCATGTAGCTGCTCAACAAATTGAGCAACAGTGGCCATGGTGTCATCAGAATTGTCCATTCCTCCTGGACAATTTGATCCCCTAAGAAGCATTTGGAATATCCTTTTGTCAGGAAATAGAATTTACTTTACATGCATGCTTTGACAAGAAGGAGTCTTCAGATTCGGAAGAGGAAAAACAGATTAGAGAGCCTTTGTTGTGATGGAATTGACGTTGGGAACACTTCTCATTGTTGTTTTTACTTAAATGATCTTATGTTCAAAGAAATGCAATTCGCACAATGTAATGGATTTCATGGAAATGTAATTTTAACGATGGAATGAATTTTGGTTTAAATGTGA

>ONT.4858.1 LG02 105635217-105643837

CTCCAAACGTTACCAACCCATGATTAGATAAGCCTCACATTCTCCACTACAGTTGCTCTAAAGAGGCCTTTTCATGGTTTGTCCTGTTGCCAATTTGCCATCCTCCCAAGTTTAGTCCCATAGGCTTGGGGGGTTTTGTGCTTGGGAATTATCTCCACAACAAGGAGGGCAAAATACATGGATGAGTGATGATACATAGGAGATGTGGGGGCATCATGATTAAATCAGATGCTTGTAGTCATGTGATTATGGACCTTGAAAACAGACAGTTGGATTAGTTTGATCCAACCCTCTATTATTCTGTAAGGCCTTCCATGATTGGACCTTGTAAATGATGCATGAGCTGTTTGAACGGTGCTGTGACATCTTTGGAAATTTTGTCAGACCTTTTGATAGTACATGGCGGGGATTTGTAATCCTTTGAAGCTGTAGCTTGTATAGCTCAATGACCTTTTCACATTTTCTATTTTTGAAGGGTTTTTAATTTCCAA

>ONT.10323.1 LG06 63894120-63895869

ATTCATCTTCTGTAGCAGAGACAGCCTCTGTTTCATCCGTTTTCTTCTTCTGTCTTTCGTTTTCTTCTTCTGTCTTTCACTTCTTCTTAATCCAAGGTGAGTTAAGTGAAGAGGAGTCCATTTCTCCACCTTTCTCATCATCTTCTTAAAGAGATATTGAGATCAAAGCTTCAAGTCAAGGGATTTTCACTCAATCCAAGCTAAGAAAGGGGTTTTTGTTTCATTTCATCTTCACTTAAGGCAAGCTCAGTTGCCCGTGCCAGTACCTCCGAGACCGTCCCGTCAGCGGAGGAGGCCCATTCGCTACCTCCAAGAGGTCTAGCCGAATGGACATGATTAAGAGCGGACACGTGGAAATCTTTTGAGAACTTTTAGAAATGATTTTATAGATTCATTTTGGTATTGTACGTATGTAAATGATGTCCCCTGCAATGTATTTTAAATATGCAGTGGTTGATAATAGGAAAACTGAAATGATGTAAAGTAACTTTACTGTTCATAGTAAAGTGTAT

>ONT.11900.2 LG07 27862563-27865389

GCCCCTTCTTTCTTTATTCTTTCTTTCCCCTCCCTTATCTTCATTTCCCTCTTCTCATTCTTCACTCCACACACAGCAGCCCCCATGTCCATTTTCCCTCATCTCTCCCGTTCTCTCCATCTCACTCTCCTCGCCGGACAACGAGCACGCCGCCACCGGTCACCTCATCTCCGACAGACTCCTCTCCTTCTCCCATCTCTTCTCTTCTCATCTCACCTCATCTCATCTCTCAATCATCTCTCCCTCACCCAGCCATGCTGCCCACTGTCCCGGCGCCATCACCGTCGCCGCCGGACCCACCTGCACCGTCGCCGAGCTCCCCCATCTCCGGCGAACCAGCCCATTTCATCTCTCTTTGTTTTCCCCCATTTTTCCCCTTCTCTGTTCATTCCTTTCCTTCCTTGTTTCTCTCTATTTCCTTTTCTTTGTTTCTCTCTCTTACTTTCTTTCTCTCCCATTCCTCTTGCTGCCAACGATTTTAGCCCATTCTCATAATCTCATTTCATTTTCTCTCATCTCACTCATCTCACCCATGTCACCATTTCAGTCTCTCTCTTTCTAATTCATTCTCTGTTTCTTATCTCTCTTTAATTCCCCATTCTTTCCTTATTATGTCTTCTTCCTTCTTCCTGTTAATGGACCACCAAGAGACCCTCCATCACCACTAATTTAGCGGGCCACCAATCAAGTGGGCCCCACAACCTCTGATGATCATTATCATCTCCGCGTGGACCCCGCAAGCTCAATCATCTCCAATTTGCAAGCCACCACTCCATCCACCCATTTGTTTGGCTTGGTTTGTTGGACCATGGAGCTAGGATTTGTGGATTTGTGCACTTTCAACCAAAGGCAAGTACCGGGTATATCTACCTTTGAGAAAGTGGAGGCGTCGCTTCAGGCAGTTTAGTCTGATGGTGAGTACGAGTTGTGTGTCGACGTTATTGTCGTCATGACCCGCACACGAGTGCCTTCATACTTTGGTTAGTTTGTATTTATTCTCTTTTGGCTTAGGTTTTGTTAGAGGTCATTAATTAATATACTTTTGCTATCTTTTGATAAACTGAAACTATAGAAAAGAAAGATGTGCATACTCTGAAATGTTATATGGTACCATCTGTTTA

>ONT.14958.2 LG10 49230969-49233642

GCCCTCCACGAGATGGAGCACGCCCAGCCCTGCAACAACAGCTCCCTTCCCTTGTTTTTCTCACTTCGCTGCTTCCAAACTCAAGATCTATGCTACCAATTACCAAATGAAGCTTGCCATTGCATTCTCCTCTATCTCTTCTTGCTCTAGACACCATTTTTGTGGGAAACCATGAATGACAGCCCCCTCATTTAGTTATTCCTTGCTGGTTGGACCACTTATATGTCCAGACTTCTTCGTCCCGAGCTGAGTGCTTTTGTAGGCTGTGGAGTCATGATTTGCAGGGTTTTTTTTCCGACTATGTCATGCAGATATTAAACTTGACATCGGAATTGCATCACATATGGGTAGTGGTTATTAGCTTGTTGTTGTATGACTTTTATTAGCCATTTCTTAGGAGATTTTAATTAATGCACATTGATAATTTGTAACTTGAACATTTGTGGGAATGCTTATCTTGTTACTATCAGCTCTCTACTTTGTTGTGATGGTTT

>ONT.7296.3 LG04 8928570-8933743

GAGACTCTGTCACGTGTGCGTCACAGCATATCAGATCTACGAGATTTCAACGAACACAAGCCCTAGGGCCTTGTCCACCTGGGTTTCTCAATCCAACATTTACCCCCTTCCTCCCTCTCTACCTCCTCAACAAATTCATTCCAGCTTCGATCAAGCACACACCTTACCTACCTGACTACCTCTGTCTGTCTGAGTCTATCCTCTCCTCTCTCGTTGATGCCCTTCTTTTCAATATGCTATGTCTGAGTCTCTCCTCTCCTCTCTCGAGTGGCCGCAAGAAGGAAGGTCTCTCTTTTTGTTTTGCTCTTGATTGGTTATCAGATTACAGGTTCATGTTGTGATGAAAGATATGTATTATGATACCTTCTATTTTCTTTTTGGTATTTTTCAGTTGTACGCCAACATAGTACTCTTTGAGCTATCATGTCTTCATGGACTATCTTGATGAATGCCATTTGATTTGTTTATAAATATTGGAGCCTATGGACTGCACATTGTATTTTTATATATATGATCCTTTTCCTGT

>ONT.7791.3 LG04 87025763-87026821

AGCTTCTAACTTCTCAATTGCAACTTCAAGACCACTTGTAAAATATAGATTCTAAGAAGGGCAATACCCAGGGTAATAATTCCGGAGAGTTGGGTACTCGAAATTGAATCCATAAACAAGTGAGACACACATGATGGTAGCAAGTTGTGTTTGGCCAATAGAAAGGAGACAGTATAAGACATAGGTGGAGTTCATTTTATCATTGGCCTGGCAGTATACTTATCAAGTTGTCCGTACAAATAACGGCTCCACATCCGAGTGATAAATAGCGACAATTCTTACCCGCATGTCTGGGTTCCTACTTTCGGTACGTCGTTGCTTCTCATTTATATGGTCAATGTTGCAAGGCAATACCCAGGATAATAATTCGGGAGGGAACAACATAGGGGGTAATAATTTGGGAGGGAACAACCCCCAGGTCCAAGGAAATATCAGGGCTGGTGATGGAGGTAACATCAATTTGGGACTTCAACGAGTAAACGTCGGGAAAAAAACGTAAGTGTTGGTGATAGCATCAATACGGGAAATCAAGGATACGGTGGAGGTAATGCCAATACGGGAACTCAAGATTAGGATGGTGGTAGCTGGACTAGCTGAAAATAAATGTCGCCAGTTATTTATGTTTCATATGTCTTAT

>ONT.3327.1 LG02 33752541-33755460

GGCTTGTTTTGTTGGACATCTTGAATCTGCTTGTGTTTAGCAGAGCTTCATCGCCTTTTCCTGCTCCGTGGAGTGTTGTAACTGTTAAATAGAGACACAAGCTGAGCTTCCGTGGAACACTTTGTGACAGCTTTATTCATCCTTTTGTTTACAGTCATGTCGATGGGATGGCTGGTTGATGGTTCCAATTGCGTCTCGAAATCTGACTGCAGTTGAGTGTCTCCTAAGTACGCATGTTGAGCAGTAAAGGCCTTTGTTGTTTCAACGTGTAGGTGTGGGTTATTGATAATTCAAATCTCTGTACAGAGTACTTAATGCCTTCATTTATTTAATTTAAGAAAAAGGGGGAGGGGGTTTGTTTTTCGTCTTTTTTCTTATTTTTGATACACGACATTATTCACTTCAAATTCATGGGCAGTCTATTTTATAATGGGCCCAGTTGTTGTAACAAAAGCTGTTAGCTCTCACTGAGCATGCGCTGTGAAAATGGTTTAGTCAAATGTGTATGACTTACAATGGTATAGTTAGTTCTCTAAAGAGATTAATAAAATTTTCTGTCATGATCACAA

>ONT.6093.1 LG03 49179508-49181542

ATTCGGTTGATGCTAGGGGCTGTGGAGAAAACCCTTTTCTCCTCTCTTCTTTTCAATTCCTCTTTCTCTTTACAAACCCTTTAATCTTGTGCAAGTGAAGAAGAAGGGTAGAGATTACCTCCTTCATCATCTCCTTCATCTTCTCCACCCTTGTTTCAAGAGATTTTCAACTCTCAAGCTTGGGTTCTTAGTTAAGGGTTTGGAATAATTGGGGATTTTAGGATTGGAACCACCAAAGGAGTAAAAGAAGTTGAAAAGCATAATTGAGGCAAACGCAGATACAACCTCCGGTCCTGAAGAGGTCCAGGAGGGAGAGAAGAGCACCCAGGAGATACATTCAGGATTAGAAGAGTGGCCAGAACCAAGGCCCATCTAAAAGTCATCTTCTCGGAGTGCGAGTTAATCTTGTAGATATCACAGTTCGTATGATTAGTTTCTTTTCAATAGTCATACGAAATTTTTTTGGAGATATTTTTGGTAGAGCTTGTATAGGGTTCTTTTAATTATATCTCTTTTGTAATTTGATGTATATGTTGTTTATTTCTTTTGTACTAAACTTTGATGTTTAATAGAACTTGATGGAATTTGATACCAATATC

>ONT.2246.1 LG01 30193828-30194922

GACTTAGGCATAAGAGGGCCTTGTGCTGGCAGTAGCCAGGGTCTCTGATCCTTTGTTCCTCTTTCTTCTCCAGGTTTTTAGCTGGATCTTCCCAAAGAAGTCCTACATTACCTAAGCAGAGGAGAATGCTGCTGTCAGTTGGGCTGTCAATGGCTTGCCGCTGGTTGTCTGCATCGAGTTGGTGTCCCACACCCATTGGAGTTCCAGCCTCTTCTCCTGCCTTGGCCACAATGATTAGTTCTGTAGCAGCCATCTTGAAGTCTGTAAGTTCTTGTAACTTGGAAATTGATTTTTTTTTTTTTGGGTTTGTATAAATCTAGAGATTCTTAGTTTTGTTTCATAGTGGGTTTGATGGGGAGTGAGATATGGTCACTTTTGTGTTGAGAGTGTGTTGGGGTTACGAGAATTCGTAATTATGAAATGGCTTATTTCTGTATTTATATTTGTCTAAGAGGTTTTCTTTTTTCTGGGGTTTGTGGGAAACTTGAGTTTCTTTTTCTTTCATATTGTGTTTTATGGAGAGGATTACCGTAACTCATTTACTTATTTTGAGAG

>ONT.8550.1 LG05 49926702-49932694

ATTTCCTTCCTCTTCCCACCCTTTATTTCCCCTCCCGCGCCATATCCCTTCCCTACCACGCCAAATCCCTTCCCTGCCCTAGCGTCGGAGCCGCCTTCGTCGAAGCCGCCAAGTTTTCTCTCTCCCTCTCTCTCACTCTCTCTCTCTATTTGTCTTACATCACTTGGGTCGGTCTTTGTGTGTGTGTGTGTGTGTAGGATCCTAAGCCAAGGACGAGCTCATAGCGCAGAAGACTTTGCAGAAAATCCTCAATCGAAGTTGGCCAGCATAAAGACATACACCACCAAAGAAATAGATAAGATCCGACTCGAGTTTATCGATTTCATCCAAGGATTTCTTCCTTTATAAAGGTGAATGAAGAACTTAATTAAACGGAGATGGAATGCCTTTTGCTAAGTTGATCTACTAATTAGATTAAGTTAATTTTGGTTAGTTAGGGATTTCCGGAAATTGTTTACAATGTAGCTTGCCTTTTTTTGGATGCTTTTGTGAAGGTTTTGGATTATGTCATGACACTTTGGTTACGAATTCGAAGGTTAATGTAAATAGTTTGGATTATGGATGGATTCA

>ONT.9880.2 LG05 104577630-104585612

AGTACAAAGCCCAACAATAGTGGTCTCCCTCCAAAGTCACCCCTCCCAAACCCTCTCCTCCCCCCAAAAAGCCAAATCCCCTTCATCTCTCTTCCAACACCACCACCTACACTTGCCTCCCTCCCTCCAAATGACGATTCCTCCTCCATAAAGGTCGCCAATGGCTGAACCCTAACCCTTCTCATCTCTCACCCGGAAGAGGGTTCTCGCATCTTCTCTTTTGTCTTCATCTCTCCAGCATCTCCAAAGCAACGGGCCTATTCTTTGTCTTCTTCGCCTTCGTTTGTTTGACACCTCCTCTGTTTTTGTCTCTCCGGCATCTCCATGGCAACCTTTATTCATTGGATGCATCCAGTATCCAGATGCACTCTGGCAATGTGAACTGGTCTTGGAATTGAATGGACCCAGCATTCTGATTAATGGATTGGACAGCATACATTTGCATTGTCTGTAGCTTTTGTTTCTGCTAAGGTTCATGATAAGAAAAGAAGCTCTTTGGGCCTGCTTTGCAGTGGAGCCTAATTTTGAAGCAATGATTTCGATCGCAGCTTTCTTCTCTTGAAGCATGAAGGAATCGATGGATATGTTTTTGCTCTTTTTGTGGGTGTCCATCGGTTACTGCTGTTACTGAAGGTTGGGACTGAAGATCAATGTAATAGTGGCCAATTGCTTGAACATCTTTTTGTTTTTCATAAAAATATTGTACTTACACTATTTTGGATGCTACATTTTTACTTGAATTAGTAACAACAACGTTGCTAAACTTGTGGGTCGTATTTAATAGTTATATGTTTCCTAATATGTTGAAAGGTCATATATTGTACATTTTTCATTAGTTAAATGAGCAGACTTTACTGTATATTTTGTTTGAGTTGAA

>ONT.15413.4 LG10 11281035-11286409

GCACTAGCCGAGAGGGGAGATGAGGCGGGAAAGGTGTGGAGAGGAGGAGGAGATGAATAGGGAATAGGGAATAGGGAAGAGGAACGAGAAGGAGGGAAAGAGAGAAAGATGGGAGGAGAGGTGGAGAAGTAGAAGAGGAAGGATAACGATGCAGCAGCAGCAGCCGGAGATGGATGCCAGATGCAGCCCTTGAGGTCCAGCTGTCCAGTCCATTTCACAGATAAGTGCTGAAAAAGGATGACTAGGAGGATGATTTATATGGCTCATGTTGCTTGCATACTTGTAGGTTGAATGCAGAACTTGAACTTGTGTGTGTGTGTGTTTTTTCTCGATTGGTACTCTCCCGAATGTAATACTGCTTTTCAACGTGTAGCAGAGGGATGGCTAGAAGTTAAGAACTGAAGATTTTTGGGACACCGCAGTCTGTTGCGCGCTTTCCTTGGATACAATGAGTATACAGAATTATAATTGAAGTTGCTGTTGAAACCTGTTTGTGAAGCAGGAGCTTTTATTATTTGTTTGGTAAGGTTACCAAAAGGTTTGTTTAATTGCAACCTAATCTTTCATTTTCATACTTTTACATTAAAGTGTGTTGTATGCTTGTTCTACAGAAAATATTCATATTTGTGTTTGTAACTAGGTTTACATGTTTAGTGCTTCCTTGCCGGCATAAGAGACTGCTGTTTTGACCTTTCGGATCTGGGTAGTGTGAGGTTTTCTTATTGATAGTTCAGGGTTGTGGCATTTGATTTTGCATACTTTGCCAAAAATTAGGTGGGTGGTTGTGAGAGTGATTTGATCTGATTTACCTATCCTTGTTTGTCACACTGCATGGTGCAAGAATTTCTGTTTCGTCAAAATGTGTATGGCACATGATCTTGTAGCAACAGATTTCGGCAAAAAATGGAATGGTCTAATGAACTAAATGAAGAGTAAAGGATGACTACTCTCTTCTGGCCAGCAGGAAGAGGTATTGGTGTCTCAATCTGCTTAGA

>ONT.5405.1 LG03 44322551-44327673

CCTATTTTCCCTCTCCCGCCTCACTCATTTCTCTCTCCCCTCCTCTCCGCAGATCTCCATTCCCCTCACGGTTCCCACCTGCTTCATCTACACCACCTCTCCACCGTCCTCCTCTCATCACCTGCATCCCATACCCCCATCGTCCGCACTGCTCATCCCTTTCTATCTCTCCCTCCCTCTCCAGACATCTCCCAGGCCCACGACCCACTTCATCTTCCTCCGCCAGACGACCACAGCAGCACTGCCAAGCGCGCGCAGCAGCCCCCTTGACGCCCGCAGAGCCTTCATCTTCTTCCTCGCGCCGCCGGACGCCTCCCCTGCACCAGGTTGCAAAAGGAGCAAGAATTGTCAAGGGCTTCAAGCCTCATTTGAATTCGTAAACCCAAAAGACGACAAGAAACCGGTTCAAGAGATTGAATAAAAATTGAAAAACAATTTCTGTTGAAAGAGTCCTTGCTGCTAGGATGCAGCCAACATGAAGGGCAAGTATGCAGAATAGAATGTTTGGCATTTGAATGTTGCTATAGAGTAGAATCCTTTTGTGGTGAATGACCACTTCTCTTACCCGGTGATTCTCCACTGTGTACTAGAAGATAAATTTGATTGGATTGACAAACCATTGTGTACTAGATATATGCTTTCTCCTTGTGTATGTTGGCTGCCCACCACTTTAGAAATCCTGCTTTGACTTTTCTCTTATCATGCAACAGTATTGTTGAGTTGTAAACTGCATCATGGTTCTGGCCTAATTTTCAGTTTATTGACCGGTTGTTAGAAGGCTTTCTTCAGGATTCCTCTTTGTACCTTTTCTTTTAATCTAATGTTATAATTAATTGCC

>ONT.459.3 Contig01280 23056-41365

TCTTGCAGCTATTGCAATTTCTGCAGCTGCGATTAGATGTTGCTGCTTGCTGCGACTGTCGTGGCTGGTGGTAGCTGCTGCTGGTGCTCCTGCTCTCCAGTGCAGGGGAGATACTGTGCTGAGCTCGGGTTATTGAACTAGTTATAGTATATTAAGCCAAAAGATTTTGAAGGGATATATCTGAGAAATAGCCAAGTGGATCATCTACAAAGAGCACAAGTGGGAACAACACAAAGAATGAGATTTTAGATCTACTTGCACCTTACCAAAGAGGTGGAAAGAATGGATTGTTTGGCGGTGCTGGTGTCAGAAAGACTCTGCCTATCATGGAACTCATTACAATGTTGCCAAAGCTCATGGTTGTATAATTGTAATGGAATTCTGTGTTGGAAATACATAACTCTGTTTATACTTATGAAAAGTCATTGGGTTATTCTAGTTATGTTAATATCCATCTTATAGGGACCTTTCTCATTGAGATAGTATGATTTTCTTGGTTAGGTTATGGTTATGTCAATGAAGAATTTAAATTGTTTTTTGGATGCTACTCTTGTCAACAACTACTGCTTGTTACCTAAACTTTAAGAAGGGTGAGAGTGCTGGGTGTT

>ONT.5152.2 LG02 127525263-127527376

ACCCATTTCATTCGTTCTGTTCATGTTTCAGTTTATGTGGGCTGCTGAAAGGGAAACTCAAACTCATTCCTTCTTCCTTTTCTCCTTCTCTCTTCACCATCATTCAAGGAAGAACTTGCAGGTGGAGGAAAAGGCGGACTTTAGCTTTCTCATTCCAATTACCAGCCTTTCAACTCCATAAAACCTAAAGAACCCTCTTCCTATCTCTTCTAATTGCTAGATCAGGGTTTGTGGAATTTGGGGGTTTTTACTTGTTTTGACTGAAGAGGGAGAGTAAGTGAGAAAAGCTTGGTAAAGGCGAACGCATATCCCAATCCCGGTCTTAAGGAGACCGCAGAGGAAGAGGAGGAGGCCGTTGAGGTACCTCCAGGCATAGAGGAATGGACGGCTGAGGGAGCTATCTAGCTTATGTCACTAGAGAGCGTCTCATCTTTCACAAATAATGTACTTTTGGCTTATAGAATACTTTTGTGTAGATGAACAAACTATACTTTTGGTATTGATGTATATAAATATTTTTGTATTATTGAGGTAGAGTACGTTTTAAATCTCTACTTCTTACTAGTAAATGAAATCATCTTTTAATTA

>ONT.8567.1 LG05 56561747-56565933

ACCCATGACCCATTTCATTTCATCCCAGAGAGTCAGAGACCGAGAGATAGAGAGAGTGAAGAAGAAAGTTTTCGTCCTACAATATGTCGCTTCATATGAAGAAGAAGCCTTTCTGAGATCTTCTCCTCTGGTCTGAAGCTGCTTTCACCCATGACTCATTTCATTTCTCACCATAAGCTGTCGCAACTCACAACTCGCAATTGCCGTGCTCTCTCTCCCCCGGTCTAGCTGCCGGTTACCCCGAGGCGGAGCTTGAGGAGACGGTCGGAGAACCGCAATGTTTAGTTGAGTGGCATGCTGATGATGTCGCTTAGACATTCGGTTGCCTTAAGATACGATACACTTTTGGGGATTGTGGATAACATATTCTTTTGGGGTACTATTTGGTTGTGCTAGCCTTTGTTGGCCTTTATTTTTTGGGGAATATTAATTAACCAATCTTTTGTATAAACATTAACTTCACTTTTGGGAAAATGCTTATGAATATTTCTCACTTT

>ONT.6896.2 LG04 74819281-74821265

ATTCGACTGTGGCTTGTTTGGTTGAGGAGAAGGACCTTTCTTCCTCTTTTTTTCTTTCTTTTCCTTTGCTCTTTTCCTCTCTCGATCATGAAAGGAATTGTGTATGAACATGTGGAGGTTAACTTGGATTTATTCTTCATCTTCTCCATCTTCTCCATCTTCCTTCTCTACTCTTGGTTCTTCCTTCTCTACTCTTGGTTGACAAGAACCCATCTCCATCATTCCCTACTCTTTCCAAATTCGGATTTATTGTTGAGGGTTTGATATTTGGGGGATTTTGGTTCATTTGAGCAAAAGATTAGAGAAAGGGCTTGGAAAGCTAATTGAGGCAAACGCTGGTGCAGCTCTCGATCCTGAGAAGGGCCAGAAGGAAGAGTAGGAGACCGAGGAGGTTCCTCTCGCATTAGAGGAGTGGCCAGATAATGGCCCTATGTAGTCTGGTCCTCGGAGAGTGATGTGGAAACTTTTGAGAACTTAATTGGAGAGTGTGACTGACTCATTTTTGTGTGGGTCACACTAGATAGATAGATGCAGAAATATATTTTGGAAATATGTTTTGTATAGAGTTGTAGGGTGATTTTTGTAATCCCTACTTTGGTTCTAATATGTAATTATGTTTTGTATTTGACATTTAACTTTAAATAGGATTGTTCAATGAAATGGTACCTAGTTGTG

>ONT.14462.2 LG10 11573185-11576614

CTTCTTCTTCTTCTTGCAACTGCAACAAGCCAAAGGAGGAATCTGAGAAGAGTGACATTGACCAATCTGATGTGAGTTCCTGTCTACTGTCCCCTTTTGGTAACAATCTTTTCATCATAAGGAAGATGCTGATGCTGAAGAAGAGGAAGATCACCATGTACAAGCTTGCTGTGTCCACACCAGAACCTAAGAAACTTGCATCTCCCACAATCACAAACATTCAGGTAACTATCAAGATGGAGTTACTTTTTGGCATTTAACCAAGATGACCAAGATGGGGTTGAAGACTTTTTGTAGTTAACTAGAACACTTGTTTTGAATTATTTTTGTCATTTGACACAACACATCGTGTATGGATGTTTTGTGGTTATGCTAAAACTATTAACTTTGAAGTGTATATTGTTGAAGGATGAATATTTTGAAGGGTTGTGAACTTGGATGATAGAATGTTGACTTATTGGATATTTGATTTGAATG

>ONT.5558.9 LG03 52013971-52017198

GCAGCAGAAAAGAAGGGAAGCCGAAGAGAAAAGATCTAGTGAGAGGAGGTTGGAGAAAAGATCATGACCCAAATTTCTGGGTTTAAGAAAGCAGATGCCAACAACTAAGGACTTGCTCTCAGCATTTGAGGACAGCTGTACAGCAAAATGGGAAGGGTAAAAGGCACTGTTGGAGTTATATAGGGCACTCACTACACCTCTTGTGAAGAAGACGGCGGTTTGTAATGTGGAGTCAGTAACGGCCAGCATCTGGTTTGTAGCCAAATTGACTGAGGCTGCGATTGGGTTCAGCGGAGGGATCTTCTGAGATGAACATTCAGGCAGTTGTTGGTCCGGATTCTGATGGCAGAAGTGTCGACACTGGGGCTGAATGGGTTGAGAAAATGTGGTGTTCGCTTTATGCACTTGATGGGGATTTGAAATTTGGAATGATAATGGAGCACAAGTATTTGATGTTTTATTTGTATGGTTTGACTATGGTTGGAGGTGCAAAACAACGAAGGCTTGCAGGTATCGAGTGAGGGATAAATAATTGGTAATTTCATAACAAACAAACATGCTAATTTGATAACATATCGATATTCATTACATATGCTTCAAAA

>ONT.16527.2 LG11 2593434-2597484

ACGACGCCAGCAGCTGCAACTTTTCCCTTTCTCTTTATCTTCTCCATTTCTTTCCAAAATCAAGTATTAAAGGTCGTCAATTGGTAAGCCAACACCGCCACGCAAACCATTGGCCTCTTCTTCACCTTCTCCGTCGTTTTCACGTGTTTCCATGACCCGCAGTCCCTTCTACCCACTTCATCTTCTTCGTTGGACCTGCTAGAGGGTGTTCTTGGTGGTAAATTTTGGGGGATTCTTGTGGAGAGATTCCAGAAAGGATTGAGAGTGTTTAGAAGTGAATTTGAGTTGCATTAGAGCCAAAGCAAGTACAAACCCTGTTGCCTTTGCGGAAGAGCCATATGAGACAGTTGAAGAGCCGCAACTCGTAGGAGAATACCATGCTGATGATGTCCTATAGACATCGGGCTGAGCCATGAGATGCGTGACACCTTTTGGGGAGTGAAACACTGTTATGGGTTCATTTTGTTACGCTGGCCTTTGTTGGCCTTTATTTTTGGGGATGTTATTAACTAAACTCATCTTTTGTATAAATACTTAACACTAATGCTTGGGAATGCACAGTGATACATTGCTCACTCTGACATCATGTTTATTAGTTTTAATCATCAGTTTCTTTT

>ONT.11136.1 LG07 4901185-4910510

GACAATTCCCTCCCATTTCCTCACTTCACTCTTCTTTTCCTTTCCACACTTTTCCACCTCAACTATCCTCAGCACCTCACCCCAGAGGCTCAAAACTTTTGGAAGATAATGGTACTGTTTGGTCATCATCCTGTTTTGTAGAGCTAATAACATTAAAACCTCCAAAGTTTGATTGTTTTGTTTATTGGTTTTTTGGAGTGGCAAGGCTTGCATGAAAGGTGCTAAAGTTTCTTGGTTTATGGAATGCTACTGTGCCTACATATTTCAGATGCTACAAGTAAACCGTCCCAGCCAAGTGTTTAACTTCTGCTCCAATGCCGTGACTGTATGTCTAGGTTGAAACCCGTCATTTCTTGGCACATTGCCAGAACGCTGGACTCCCCTTCTTTTGAGATGGTATGTGCTAACTGGTTTTTATTGCCATAGTGGCAACAGTAATGTAGATATTAACAGTTATTAGATGAATAAAAAGTAGTGCAGATATTGGATGAATAATTAGGATTCTAGTTGGGAAACTGATAGTGTCTTCTGCGTCACAAAATCATGGATACTTATGCTTTGGAGGCTGAACTCAATACTGATGATAGAAAGACTGAAGCTTTGTGCTTGATTTACCAGCCAGCGACTCATTGGAGGGGGTCTCATTATAACTGCATTTCAGTCGGGAAACTATGTACTGTGTGATGTAGTGTATCTATGGAAACAAATTCAATGTGCATTTCATAACCACTGATACGTATCCTCTGTTGAAGAGAAGGCAATATTGTAGCTGAATTTTTTTTCAAGTGTATCCACCAATGTTTATATTAATTCTCATCAAATACTACCAGTACCAGGGCATTTTCACTAATTTGCAGGAATGACATCGACAAGCAGAGGATGAAGCTTCTTTACTATCTTTCCAGCTGCTGACTCTTTCAGGGATAGGAGGGATAAGCTTTATGTTGCTTGAAGGGTTACATTGTTTTTGTCAGTGTTGTTAGTATTGTTGTTGTCTGTATATTGTTGTTATATTCATCTCTGTTTCCTTGTTATTTTGGACCTTATAGTTAATAATAGGATCTGCTTTAGCAAAAA

>ONT.13481.1 LG09 49886781-49901906

ATGTTGGAACAATACCAATTTCTGTTTCTCATTTTTCTCTTCCATGCATTCCGGATTCAATGGAGGTTCTTTGAGCTTACTGGGTATGCGGCGAGAGATAACGAGGAATTTTTTAAGCTCCTATGAGGTTTGATGTTCGAAGAGTTTCTTACTTTTTTTTGCAATGGAATTGAAAGGAGAGAAAGCTCCGTCGTCTCAGTACCCCAGGTACAGCTTTTCATGCTCCCTCTCAGCCTCTTCCGACAAAATTAGTTGTGCTTTGCGGCGTTTAGAAGCTCAGAGCTGATGTGAAGAGGAGCTACAATGGAATGCTGAGCTGCAGTGCTGGGAAGTGTTTTGCTCTATAGTGGTTTCTCCCATTGCAAAGAAGTGTAGATTTCTGCTCTTATGTGGTCTTCTAATGCTTCTTTGAGATGCAGTGTAGATTTCTGCTCTTATGTGTTTTGCTCTTATGTGGTCTTGTGAAGAGAACAACTGTGCATTGTTAATTTTCTATATATGTTGTTGTTCTTCTTCTTCTTTTGTTGTATTAAGATTTATTTTCTTTAAATTAAGATCGATGAGAAATTCATGGATTAGAGACATTGTTATTTCTTATAATGAAATACCTGACATTGA

>ONT.10662.1 LG06 33950151-33960459

GGCGGAGGGGTTTCAGTCGAGAGGGAGAGAAATGAGGAAGAAGGAGGCTTGGGGTCTTATATACTGAGGCCCTACTCATACCCTTCTTCCCACTACATCATTCTGTTTGTTAGAGGGTTTTCTCGGTCACGCTCACCCTTCTTCTCAGACCCCGGATATAAGTTGGAAAGGTATACAGCTAATGACTCACGTATTATGGTAGATGTTGTATATAATCCAAAGTGGAACTTTCCTCATGCTCTGCCAGGTAAATATTATGTAATTGACACAGGGTATCCTAACACACAAGGTTTCCTTTGACCTTATAACCACCGTCGCTCTCATATTCAAGACTTCCGCCATGATGCTGGATCTAGAGATAATGCGGAGCTCTTTAATTTCTATCATTCCTCACTAAAGAATGTAATAGAAGTATAGAACGTTGCTTCAGGAAGCTAAAGGCGATCTTCCCAATATTGAAGGAAATGACTCCTTGCCCATTTCCTACTCAAATGTATATAATCGTCACTACAATGACAATGTATAACTTCATCCACCAGTAAGCGATAATTGATACCTTGTTCAATTATTCATATTATATATGAAGCGAACTGGATCCAATTGAGAAATTGAACAATTGGATCCAATTGATTTTGACCATTTTAAATCA

>ONT.641.13 Contig01843 19684-38682

AACTTCTAACTGCTCAATTGCAACTGCAAGACCACTTGTAAAATATGGATTCCAAGAAGATTGGTGGGGGAGGGAACAACCCCCAGGTCCAAGGGAATATTAGTGCAGGTACTGGAGGTAACATCAATTTAGGAGTTCAAAGAGCAAATGTCAATTCGTGAAATCAAGTAAGAGGTGGTGGTAACATCAATACGGGAAATCAAGGATATGGTGGAGCAAACGTCAATTCGGGAAATCAAGTAAGTGGTGGTGGTAACATCAATACGGGAAGTCAAGGATACGGTGGAGGTAATGCCAATACGAGAACTCAAGAATAAGGTGGTGAGAGCTGGACCCACTGAAAATTAATTTCGCCAGTTATCTATGTTTCATGTGTCTTATATAATGTTTAAGCTTTGTCTACTATCAACTATGTCCGTTTACTACGTGTGATGCAGTACAACATCATTTCTGCATTTTGTGGATTCCAAGTGGCTATTGTGTAATGGTTGCTTGCTTATTAATATAAGTTTGTGTTATAT

>ONT.3594.2 LG02 97768141-97769463

GGATCCCCTGTTTGGTCCGAACAGGAGTCTCCACATGGGGGCAGCCCCCCCCCCCCCCCCCCCCCCCCCCGAAAGCTCCCGAAAGCTCTCTTTGCCTCCCATCTTCATCGACCACCCGAAAGGAAGTTCAAGCATCATCCCCGTCTTCCTGAGATTAGGGATTCACTACATAGGACAGAGTGGTGGTAGTTTGTTTTGAAGAAAAGGGTTTGAAGCATGTTAAGAAAGATGGGATCACAGTGGGATAGGACGTTGAGATGAATAGAAATTTTATAGTTGTAATTGATCTGTAGCATGTTCCGAAACAAGGAATGAAAGTGGGGTTTCAATGCATCTTTGGAAGTGAAATATGAGATTTTGATCTATAGAGATGGGTGTTTGTGTTTGTAGAAATTGGTGGTTTGACACTGTAGAAAAAAGTTTGTTTGTATTTGTAAAAATATGTGATTTGTATTTGAAGAAATAGATGATTTGAGTGTTGTAATGGATTTGAAAATTGTTT

>ONT.15186.3 LG10 87483635-87487523

AGAAAACACTCTCCTCTTTCCTTTCCTCTTCTCTCCTCTCCTTCCATGACATCTTGAAGAACTTCACATGGAGGCTCCTTTTGCAAGTCTAACCCAGCCAAGCAACATAATCTATTTTGGTGCGGATCCAACAGTGGCAGCCCCTTCATCAGTCACCTTTCCTTTCTTTGAGCTTCAAATAGAGATTTTGGTGGGTAGTTTGGAAGAGAAAACTTTTGATTTTGGAGCTTGAAGTGATGGCTAGGTTGCATTTGATTCTTGATTTGAGGCAAGTAACGGGCTTGTCTACGCACCAAAACTGAACCTTGTGGTGGTCCAGCAGGATTCGCTAGTTATTCAAGAGTGGGAGCTAACGACACAGAGTAGCGTCTAAAGCTGCAGAACTCATAACATCATCTTTTGGGTTTTTGTTATTTTGGGGAATTTCATCTTTTGATGATTATAATTATTGGCCAGTGTTTTTTCACGGCCTTATTCTGGGATGTAATTAAACTTAATATTTTGTTAGACCTGAATACTAACTGATAGAATGAATTCCTTTATGCTATCCAGTCTGATAATGCCATTATACT

>ONT.17310.1 LG12 47505610-47507460

ATCGCCTCCCATTTCAGACGCTGGACTCTCATCTCATTTCCTCTCGGCTCTCATCGTGTCATCGCCTCCCTCTCTCAGCAGCTCCCCCTCTCTCGTCCACGAGCAGCTGCCCCGGCCCTCTCTCAGTGAATGTATCCAGGATAAAACTGGTGGTGCTGTCATCAATAGACAGGGTGGTACTATTGTGTTGTACAGAAGCAGATACTATAATCCCAAGAAAAGACCTGTGATTCTACTTATGCTGTGGAGACCCCATGAACCAGTTTACCCAAGGCTGATCAAAACAGTAATTGATGGTCTGTCTATAGAAGAGACAAAGGATATGAGGAAGAGAGGCATGTCCACCGCTTGGATGATCATGATGCATGAATAACTCTATAAATCTTTTCCTGTCATCAGGTGGTTGAACACGTGAGTGCGACTTTGCGATATAGGAATTTATACAGGCATGACAATGAACCTATGTGACCAAGCCATCTGAAATGTTGTGAAATGTGAATGATCCCTGTTTCCCCATTACCAAAGGTTTTCTTGATGCTGTAGCGAAAAAGAAATAGAAAGGGAAAAAATGATTTATTGTTGTGTGCAAGATGTAATGTAGTCAATCTGTGGATTTAGATAAAATCTTATATGAATGAAATTAATGTTGG

>ONT.4722.1 LG02 66221770-66227227

GCTATCTCCCTGCTCACGCATCCCTTCCCCCCCCCCCAAAAGAAAAAAAATCTATCATCTAGGGTTTTCTCTCGAGCTCTTCGTCTTTCGTTTCAGGGAAGGAGGAAATGTTACCCATTCCTGAAGGTATTCAATCAAATGATGCTTTATTACCAAAGGATATATAGGCAATTCCATTCAGACCGCTCTCTCAAAGACTCAGATGATTGCCTGATGACTGAATGATGAAGATGAAGTGAGAGAGGAAGGAGGAGAGAGTGACTTGTGGGATTGAATCTTTATGAGAGACATCCAGAGGGCGGGCCATGCACCGTGCAGAAGGTGTTGAGTCCCACCAAAGTTTTCCAACGACAGTTCTTTATGCAATAAAACAATGTGTCCACAAAATGAGCATGTGCTCTAGCAAACTGAAGCTTTCAGTTGGGAGGTTCCCATGGTCCTCATGTTCATGGATTATAAACATCAACTTTGCCGGTTAATTGACACCAAAAGAGTTTATAAAAACAGCTCTTCCATCACAACTGAGGAAGATCTCCATGAGGAAAAAAAAGACGACTGACATTTCTTCAGCTTGGGCATAGGGATAGTGGCAGCTTCTTGAGTTAGAGCAACTAAAGCCCCGGACATATCAACATCTTGGAGTTCACCCTTTTCTTGCATGTAATTCTCTACATTCTGCATGAACTCTTTAGTATTTAGTTTGATCATTGGAATTTCGTCAGGATCTTCTGTCCAGAAAGCATCCTCAATTGCACTTTCCAAACTGTATAAGCACTCTGGCTCTGGTGTTGCTGGCTCTTCAATAATAGGTTCGCACTGCATGAGTATAGACCTTGCTTGTGAGTGTGAGCTCTACTAAACGAGTCCTATTGAAGGCTAGGTTCAACCAAAATAAATTCTATGATATATGACCATGGTCTTGAAACATGAGTCCGTGTCCTCTTAAGAAAATGTCTTCTATGTAAAAGAGGGGGTCTGTTTAATTTCATCAAAGTTAATACTGTCTCATTAAAGAAAGCAAAAGTTCATGGTGAA

>ONT.99.1 Contig00292 58233-61194

AGGTCACAGAACCCACTCCCCTCTCTCATTCACTCCACATAGCAGCAACCCCTCTCTTTTCTCTCATCTCATTTCTCTCGTTCTCCCTCCCTCACGGCTTGCTGCCATCGTCGCCGGATGTCGTCGCCGCCGGACTCCGAGCACGCCATTAGCACCACGATCACCAAGCCCTCTCCATTCTCTTCTCCACTCTCTCATCTCACTTCATTTCATCTCTCACTCATCTCTCCCTCACCTAGCCATGCTGCCCCCTGTCCCGGCGCCATCACCGTCGCCGAGCTCCCCCATCTCCGGCGAACCAGCCCACTTCATCTCTCTTTGTTTTCCCCCATTTTTCCCCTTCACTATTCATTCCTTTCATTCTCTGTTTCTCTCTTTCATTCTCTGTTTCCCTTTCTTTCTTTGTTTCTCTCTCTCACTTTCTTTCTCTCTCACTCTCTTGCTGCCAACGTGAGATAGCTCACTCACCTCACCCCATTCCACCATTTCTCCCTCTCTTTCTTTAATTGTTTTTATTTTCTCCCTTGGCTGCCAACGATTTTAACCCATACCCATTTCATACCCTCTCATTTTCTCATCATCCCATCTCATCTCACCTCTCCCTCACCTCACTCCACTCACGTTGACTTTTCTCCCCTCACCCATGTCACCATTTCACTCTCTCTCTCTCTCTAATTCATTCTCTGTTTCTCTCTCTTTATTCTCCATTCTTTATTTCTTTCTATCTTCACTCTCCTTTTGTCAATGGACCACCAAGAGCTACATCACCACTAATTAGTGGGCCACCAATCAAGTGGGCCCCACAACCTCCGATGATCACTATCATCTCCACGTGGACCCCGCAAGCTCAATCATCTCCAATTTGCAAGCCACCACTCCATCCACCTATTTGTTTGGCTTGGTTTGTTGGACTATGGAGCTAGGATTTGTGGATTTGTGCAATTTCAACCCAAGGCAAGTACCGGGTATATTTACCTTTGGGAAAGTGGAGGCGTCGCTTCAGGCAGTTTAGCCTAATGGTGGGTACGAGTTGTGTGTCGATGGTATTGTCGTCATGACCAGCACACGAGTGCCTTCATACTTTGGTTAGTTCGTATTTATTCTCTTTTGGCTTAGGTTTTGTTAGAGGTCATTAATTAATATACTTTTGTTATCTTTTGATAAACTGAAACTATAGAAAAGAAAGATGTGCATACTCTGAAATGTTATATTGTACCATCTGTTTAATTATGTTGTTCCGCTGTGTTT

>ONT.7061.1 LG04 86827848-86828876

CTTTTCCAAAACCCCTTTCCCACCATCCCTATTCGGTCCAACCCATCTCTGCCTTTTCCAAAACCTCTCCAGCCCCACCATCCCCTATTCAGTCCAACCATTATCTGGCTTTTCCAAAACCTCTCTAACGGTCCAAGTGTCCAACCATTCTCTGCCGTGACTCGTGAGTCAAACGCCCATCCATTGAAGTTGTTGCTGTTGATATATCCGTCGCCGGTCGCACGGATTGTTGCGTGGAACAGTGAACACACCGACTAGGCGACTAAGACTCAGGGCCTTGGAGCAGGCTGAAGGAGTAGAGGTCTGGATTAAGATTTGGGGATTTTGATATTCAAGTAATGGATTTGAACTGTGGAGATGGGTTTTTGATGAGCTGCAGTTTGATGATGCAAATGCATCTTGAATCTTTACCATTGATTATGGAGATAAGAAAAATGGATTTTTCCGTTTCTTCTTCTTTACCTGTTTAAGCTATATAAGATTGCATAAATGGAGAAGGGAGGTGAGAAGAGGAAATGAATATAGTCTGGGTGTACCCCACTACTGATCTTTGATATGATCTGTTGGATTGGGGGGTTAGTCTTTTGAAATGGACTTTGGACCGTTGGTTTTTGGAGAGAGAGGAAGAAGAGAAACTAAAGCGTTTGTGACTGTTCACTGTGGAAGGAAAGATGGCATTGAATGTAGAGAATAGGAAATGGGTGCTGCCGCTGTTGCATTACAGAGTTCCATTTGGGTTTCATTATGCTCTAAAATGTTGTCTCTTCCTACACTCCTGTATTTTAACTTAACTGTTTCCAGTTAGTCTAGTTTCTTGTCTCTGTAGTCTGCAAACCATTGAGATAGCCGTGGTATAATGTTTTCCATATTAATCTG

>ONT.416.1 Contig01188 17722-19358

AGAGGAGATGTCTGGCACACAGGGATCCTCTTCCCTCTCTCTACCCTTTCTCTCTCTCTCTAGCTATTTCTTCTCCTACACGCCAAGAAGAAATGTTAAAGTTCTTATGATCGAAAACTGAGATGCATCCACGCCCGTCAGCAATACCCACCATCTTCAAAGCATTGGACATCAAATCGGGAGTCTGCCATTGAAATTCTTCAAGTGCAAATTCTTGAAAGGCAGTTTTGAAATTTGGAGCAATTCTATGGTGTAAGAATCTGAAGCTATGTGCGAATGGATTATCAGATCATTTGTGGCATTATGATCGGGTAGTTCGTATTAGAAAGTCCAGGGAGCTGAATTTTCTTCAGTGGGAGTCCCGCCATGTATGAAACCTCATCTCTGCTGTTAATGCAGAAGGGGCCCCTCTGCATTGAAGGCCAACTTGAGTTTTCATGAACGGCTCAGATTGCGATGCCCACAGAAGAAAGCAGGATGTTTAATTGTCTTTCTTTTTTTTATTGCTTTCTTATATGTAGATGAGAATCATGTATAATTCTTTTCAAGTGATGGTTAATATTGATGTTGGTGTTTAGCTATAACTTTTTATTTGTTACATTTTTTATATTTAAATTAGAATTCTGTTTTATTCGTTGCAGGTGATGGTTGATAGTTGATATTGATGTTTAACTGTACTTCTTTTATATTTAGATGAGAACTTTGT

>ONT.11869.2 LG07 19346028-19349068

GCTCCCGCGCCTCTCCCTATCTCTTGCGCCATCTCTCCCCTCCCTCTTCCTCTCTTTTCTCCTCTCTCCCTCTTCCTCTCTCCCTCCCTCTTCCTCTCTGTCGCTGAAATCGCTCCATGCTTCCACGCTCTGCCCTCCCCACCCTGCACGAGCCACGCACCCGCCGGTGAATCCGCTTGCCGCCGAGCCCCACAACGTTCTCTCCCTCTCCCTCTCTCCCCTCTCTCTCTTCTCTATTTTCTCTCTCTCGCTCCCCCTCCCTCCGTCTGCCTCTCGCTCTGTCCCGTCCCCTCCGGCTCCTGTGTTCATCGGATTCCGACCGGGATTCGCGTGAATCTATGCAGGTGCTCCGTCATCATCGATCCTCTTCTTGAAGCAGGAATAGACAAAGCTCTGTTAATGAAAAAACAATATGGGTCCACCAGATTGACTCAAAATCAATGGTTTGGATGCAGTATATAGGATTGGAAGTCTGAGGATGCCTCATATCAGCTGGATGCCTGAATTAAGTTCCCAATTTCTTTGGCACATGTTCTTGCATTCTTTTTTTCCATTGTAGGTTTATGAGAAAAAATATTGTCATTATGTCTGTTGATGATTAATAAATGGGATTTTGTGCTGGC

>ONT.5927.1 LG03 26965626-26972086

TGCAGAAGATGAAAAGGAGTTCGACTCTCTCCTCTTGATGATGGAGCCGTCGCTGGCGACAACGACTGCGATGACGGAGTTTTGTTGAAGCGAAGAGGGATCTATCTGTTCTTAGGTTCACGGTAATTGAAGAGGCTAAACGGTGAAGATGTGGATCATGCTTGTGTTAATTTCGTGAGACTTTTGGTGAATCCTTTTCAAGTGGTATGTTCCCATGTTGTACAAAAGTTATTGCAAGAAAATGGCAAAAATCTGAGGAATGGGGAGATTATCTTATTTAATTTCGGTACACCTGCTTTGTTGAAATTATTCCACGGGATGAGGCTCCACAGCGTGAACTTTGGCTGAGTTTTCTAAAGTACATTACAATTCACCCTATGAGATCCAAGGTTGATTCAAGAAAGAAACACTGGTTGTTTTAGCAGTCTGTAACTTTTGGTTGATGCTTTCAATGGGAAACCAGAATTCTTTATTATAAAAAAAAGTATGCGATCAATGGAGATTCCATGAAGAAAGGTGTTGGTCTGAGTCGGAAACTTGATTCATCTTATTTTGGTTTGAAAAGTTTCAATTATGGATTTGAATTTTTGAATTGTATTTTGGATTTTCTTTATTATGAATTTGAACAATGGAGATCTTGATTTG

>ONT.4932.1 LG02 110891333-110897836

CCCGATCTGTGCTTCTCTCACTCTGTGTCTTGCTTGCTCTCTCTGTTCTCTCTGCTATTTATTTCTCTCCCTGGCTCGATCTCTGCGCTCTCTCTCGCTCGTTCTCTCTCTCTCTCTCTACCCGAAACCGATCGTCGCTCGAGGAAGGGTCTCCCTCTCTCACGCCGCTGCCTCTGCTCGGACCCTCTCTGCCGTTGCTGCCTCTGCATCTCTCCCCTCTCTGCAGGAATTTCTATCAGAAGCTATAAAATTAAGTTATGGACGTCAACAACTCAGTTTGGGCAGGCCAAGCAACAACATGGGTCAAGTAGTTTTCGACAGTGGGAGCTCCTATACATACTTTACTAAAGAAGCGTATGCTGGTCTAATTTCTTCTCTAGAACATGTTCCCCTTGAAAGACACAAATTCAGGAAGCATTCGATCAAACGCTGCCGGTATGTTGGCGAGCTAACCTCCCAGTCAGGTCTGTTAAAGATGTGAAGAAGTTCTTTAAGCCCTTGACCTTTCACTTTGGAAGAAGATGGTGGATTAGATCCACAACACTCCTGATTCCTCCTGAAGGTTACCTGATTATAAGTAATGAAGGCAATGTGTGCTTGGGAATCCTTGATGGTAGTGAGGTACATGATGGATCAATTAATATAATTGGAGAGGAAGGATTGACTGCTCTCATTGAAAGCCGGTTGTAGGACTTCTTCTCACTGTGGAACCCACCTGGATTCATCCAAAACCAATCTATCTCTTCTTCAAGATATTTTTGTTCCAACTTACAAGCTTCTAAAGATTGATTTTGGATATTATTGTGCTGGTTGCGGGCTTTTTAGGGATAACTTTTTTTATTTTGATCACAGACATTTGTATATGTTAAAGATTTTTGGAAATTATCAAGTAGTAATATTTGTCTATTTTAAAGATTTGTGGGTTTATTTTGGTCTATGGGTGTAGGATATTTGGTTGTGGATGTTATTTTCAATTTGTTAATGAATCTATTTTAAACTGAGTTGTGCA

>ONT.3307.2 LG02 32300056-32301957

AGGCTCTCCTGCTGCTGCTCCTCTCTCCTCTCTCCTCTCTCAGCCCTCCTTGCTCCTGCAAAAATAACCATCTCCTCTCTCTCTCTCTCGTTCTCCTATGCCAAGCCCTCAGCGCTAGGGCTCCCTATCCTCTCCACGCTCTCCTGAAGGTTTGTTTCATCCCGTGACAAAGAAGGATGATGTATGGTGACCCGCAGCAGCAGGTTGGTGATTTCCATAGGGGGCACCTATGCGTCAGCCCTCGGCTTCTTCCAATAATCTCCAGCCAGATTATCTTCATTCGAGCGCCCCTCCTCTTCCTCCTACTCCTTATGTAGGTAATATGAGACCAGAAGAATGAGGATTTCTACCTTTTTCTTTTGGTTTTCTCTTTTGGGAATGGTGACTATGATGAAAATTCATGGCCTAATTGTTTCCAATGTTTGTTTTTTTTTATTTCATTTTTTTTTTGTTGCATTCTTTTTTAATTTTTTTCTTTTTGGTTAGTGGAAAAATATTGCTTAAACAAGTGCTGTTACAGTGCTGCTGAAACTCCAGAACTTATATGCGTTACCCTTTAAGTAGAAAAAAGTGTTGGGCTTTGCTGCTCATGCATCTGTTGCCGAACACAAGAAAAGAAAATGAATGTGAGTTACAAAATATTTCAT

>ONT.2954.1 LG02 6898905-6908160

GTTGTTCCACGACCATGAAACCCTTCTTGCTACTCTCCCTCTCGTCGATCCACAGCCGGAGCTCGAGCCATCGATCGCAGTAATGTTTTGAGGAGGTCGCAGTTGATTTGAGTACTTTGTTGGACAGAATCAAGCTCTCAAGCATTACTTTGTTGATCAAATTACGAGGACTTGGAGGGAAGTGGTTGCAAGTTTATCAATGCGGTATTTCCAAGGAGGATTCATAAATAGGTTTTGAATTCCAAGAGAGTATTTATTCCATGGAAAAGGAATGAAAACCATACTCTCTACATCAATTCTGCAAAGATTCTTGGATGTATTGTTATCAACGATTCAAGAGAGGAACTTACACAAGCAAGTACATACCCATTATCGCACGTGTTAGCGAGCATCAACCTCCAACCTGGCCATCTTATTTTATGGACATCAATGTTTGGGTGTTTTTAGCTCCAGCTGGAATTATTATTCTTCATCAAACCCATGTCTAAATCGGAGGGACTCAAGGATTTCTCAGATTCTTCATCTCGGGTACCTCTCACTAGTATTTAAAAACAGTAATATTTTATATTATCAGCGACCCAACGTGCTGGTA

>ONT.3193.1 LG02 22961738-22973073

CTGTCTTCCGAAAACCCCGTGGACAATCTCCGAAGCGAAACCTCCTCGCCAAATACGAATCCCCGCTCCTCAATCCGATTCCCAAAGTCCAAAACCCTACACCCTCATCCCTCGATTTTCACTCTTCTGGGGTTTATTGGATCAATAAGTCGTATAGGGCTCCCCCTCGATCCAGACAATGACAGAGATGAGGTTCGCCATTTAGTTTTGCTTCTTTGCCAATTTGTAAATTATATTGAGGTTGCCTGTCTACTACCCCTTGCAGCATGAAAAGGAAGATCCTAAAGTTAAAGCTAATAATGTGTGTAACTTAATGGCCAGTGAGGGGAATCTAATGCATTCTGACATTGGGCTTCCTACGAAGTGATACCATGCTGCACTAAATGGTTTGTTTTGCCAAAAGCCAACTCGGACTCCTGCCCTGTACATAGTCTTCTTGAGGATCGCAAGTTCATTTAAAATGGAAAAGAAGAGGGGAAAAAAAGCTCATGCAGATCAAGCATTTTTAATGCCTCATCCTGGAATATGTAGCTCTGGACATTGAATTATTGTTGTGGTATTCTCTTGGTTGTTCAAGCATCACTCATTTGGGCCACTGCTGTCTCTCCATTTTTTCTCTGGAAGCATTTGTAAAACCCTTTTTCTCATGGTAAATGTAATCACACATTCCAAATGG

>ONT.192.1 Contig00545 68217-70926

GAGTTATCTCCCTCCTTTTCTCCCTACACTCGACAAGGACCAGCTCCCGGTAGCTGGAGCTCTCATCTCTTATCTCTCTCAAGCCTCCACCAAATCCACTCAAAACCACTCCCAATCCTCTTTCTTCCTCCTCTTGCTTCTCCAAGCCTCCATCCTTGCTCCAGCCCTTTCTATTGGCGATTCCAATGCATGGCAGCCCCTAGGTTGGGCTTTTTTCGTATTTTGCTCTCTTAAGAGACATTCGTGTCAGACCCACACTTGGTGCTGTAGGTTACTAAGTTTTTGCCAATGAGCCGATCTTTGAGGCGGTCTAATAGGATCCGCCAATTATATGAAAATGGATAGTTGATGATGTAATCTAGAGCATAGAGGCTTCGGAGTTCGCGACAACATCTTTTGGGTTCTTTTTGGGAGTTTAGCTATTTTGGGGTTTTGTATATTGGCCGATGTTTTTACCGGCCTTATTTTGGGGATGGTAAATAACAAACTCTTGTTTGTTTGTCAAACTTGAATGTTAATATAGAGAAATGCGTTTCTTTCTGC

>ONT.12069.6 LG07 71410488-71412027

GAAACAAAAGTGAGATCGATTCTTCTCCCATTTCCCTTTTTCCTTCTTCCATCGGTTTCTACCTCACTTTCACCAGCGAGCATCAGAGCACCGACGACCGCCCGTACACCGGGTGAAAATGGCTATGACAAGCTTATTTGGAAGGATGGGGTATTCATGGACAAGATCGAAGATGCAAATTCCGCAGTTAATGTATCTAAGAAACTTGTCATTTGTCAACGTAGGTGGAGCGCTCTAAATCAATGTTGAAGTCGGTAGATATAGAGGTGGAGCACATGAATACATCTTTGTTCAGCCTGGAAATTGTAGAGATGGTATGCAAACTTTTTCATTTTGTTCAAATTATGTAATGTATTATCTTTGTAACCTATATGTTTTTTTAACATAGGCATGTGATCATGTGTGGAACATATAAGTGATACCGAATAATCAATTAATGTAAACTTTTTTATTATAGTGAAACCTAGCAATATTTATGT

>ONT.15839.4 LG10 39546516-39547553

GACATGCAGCATGCCACATGGCAAGAGGCAGGGAGCTATTTATCTTGCTCTGTGCACTCTACCACTAGTTCCAATAGCACCCCAATGGCTACTTCTGCTTCCATTTCTCCATTCCTCAAATCCAGAGTACTTGCAAGGGAGCAAAAGGAATCATAGATCTGAATCCTATAATTCAGGATATATGTGGTGAATCTCTAGTTGAGAACAAAGATGAATTTCTCAAAACTTTTTCAACAGAAACTCAGTTTTCCAGAAACATCATCTCAAATGGAGAAGTCATAGGCTCTGAAGCTTCCAAGGGGCATGATGATGCATCTTATAGCTATTTGGAACCAGAGGCTTCCATCACGGGCGTGCTCTCTTAATTTTACTTTTATTCTCATTTGCATTTTGTAATTTCATGATGCTATCTGGCTTCTATGTCTGGATTTTTCAGTTAAATAAGCATTTCCAAAAGTAATGTTCTAGACT

>ONT.4187.1 LG02 8154439-8156240

GTTTTCATTTCTCCTTTTCATCTCCATCACGTTTCAGCTCCCCCTTTCAGCTGCAGCACCGACCACCACGTGCAGAGGTTTCATCTCTCCCTCTCTCGTCGAGATCTCTCTCTCGCTGCTGCCTCTCTCGTCGAATCTCGGATCGTCTCGCTTCTTGCAGCCGTTTTCAACCGCGAAATCTTTATCCCGCCAAGCTCTCACACTGGCGGCATCACGAACGGCCCCCATTTCTACTTGTCTTCTCGACCTCACTGCTGCTTTCCCCCTCCTGCGATCTCCTCCCTTCTTTCCATCTGAAAAGAGCGCGACAGAGAAATCCTGCGATCTCCTCCCTTCTTGTGATCTCCCAAGTTTCTCAAGAGAGAAGGTGACATCCAGACGGTGTATCTAGAGAAGGTGCCTTTTAAGACTCAATGCTTCAATTTCTACTTCAGTTTCTGAAGTGAGATGAGATGTTCGTTCTATTTTGCAAGAATTTGAATCTAGCTAATTTCGTGTATCATGTGACAAGTTCTTTGTGGAACGTATGTAACTTTGAGTTTGTTTTCTTGTAATACTCTAATAGTTTCAGGTACTTGGATTAAGCAAGTGAAGGTCATTGCAATGACAAGGGTTCTAGATATGAAATGGGTTTTCTTTACTTATGTGTTTTTCTTTGTAATGGAATTGGTTTCCTCTGTTTTTGTTGTTTATGATCATTATGGTGGAAAACGATTCAAGATATATATGAACTGGGGATTTGATT

>ONT.13354.1 LG09 17884169-17910017

GTAACTTTCTTTTCTCCTTCCTTTTGATTTTCTTTCTTTCTTTTATTTCCTACACCACCTTGTTGCCAAATGGATCATCAAACCATCATCCTCGTAATCCAACACCGCCCATCATCATCGGAAGAGTCCAAGGTTCATTTTCATACAATTCTCTTGGGATTTCCCCTTCTTCATCATCCTAGGCTTTGGGGAAATTGATTTAGGGTTGAGCTTTTGAGCTTCCACCTAAACCAAGGCAAGTCCCGTCGTCGGTTATTCCGAGGAACACGAGGAGGCAGTCGGTGAACTGCAGCAGTTAGTTGAGTGGCATGCTGATGATGTCATGTAGACATTAGATTTCTAGAGAGAACATTTTGGGAAAACTTTTAACCATTCTTCTTGGGGACTGGTATTTTGTTTATATTGGCCTTTGTTGGCCTTTATTTTTTGGAATGTAATTAAATGATCTTTTGTATAAAAACTTTAACTTACTTTTGGGTAATATGCTATCGATCATTGCTCATTCTGA

>ONT.12283.1 LG08 1991470-1993439

GTGTTTCTAGGCAAAGCATCAATTGCAGAATTCTCCTTTTTACGTTTGGGTATTTGGGTGGTGGGATAGGATCATCAGGAATCACATCTACAGTTCAAACGATCATCCTTGCATTAGATGTGATCCTGATGATCCAATTCAACCGCTGAAACTCTCCCCATGAAGGTGGTTATTGTTTGGCTCAACACTGATTGCAGCTGTAGTAGCCTTTGATTGAAAAAGGTTGATTTGATCAGTGTTTTTATTTATTTGGGTTGATGTTTGACATAAATCTGAATTTTGATTTTACAAGTGAAGTGCAAAGGAAAACACCATGTTACAGATGAAGGCAAAGAACTATGAGTTGTGACAGTTGGGACTTGTTGATTGTATAATATGTAATTTAAATTTTTGTTCATCTGGAACTTGGTTGTACAAATAGCTTGGATTAAGGGTTGGGACTTCTCTTGGGACTTATTTAATAGTAGATTGTAAGAATGGTTTGAATGATTAAAATGTAATTGGAGGGATTTGTTCAGC

>ONT.1209.3 LG01 28813126-28813967

GGGACAAAGGCTTTCGCTCAGATCAGCTATTATAGTGTAAACTTCACTCATGGTGTTTAGAACAATTTGTTTATTAAATAAATATTATATGTGTTAACTTGTGTAATGACAGCATGATCTTGAGACGGGGAGCGGGCCTTCCCGAACTAAGTTGTGGTTCACTACCCGGTTTGGCAAGAAGAAAATTGCTTGGGTGGATTCGAGTTCTCATGAAGTCTATGACGAGCTTAAGAAGCTAGAGTCTCAACCGTTAGAGGAGAGCGATCAGCATATATACGTGGTCGTGGGGCTGGTCCAAAGGCCACAACATGGACAGCTGGGCAGCGGATCTGTGTGCAACTAGAAAGGGAGAATGAGGAGTTTAGGAGGCGAGCTGAGGAGGATAGAAGGCTCTTTGAGGAGATGGAAAAGGAGAAAAGAGAGATGGCTTTACGACTAGAGTCCCTAGAGTCCTAGGTTGCCTCGCAGCAAGCACAGATGCATGAGGTGTGCAAGCTTCAGTAAAGTCTCAATTCACCACCCTCCTCCAGTAGTTCAAGGATGGAGGGCAAATTCCGATGTGATTCATAACGGGTCCAAATTCCGATGTGATTCATAACGGGTCAAAGGCCCCAGATCATAATTTTTTATATTTTATTTGAGGTTTTATTTTATTATGATGATGTAGACTTAACTTTTGCTAGTTAAATACGTTGGAATTTAATGATTTTGAATTTGAAGTTATGATTCAATA

>ONT.10691.2 LG06 45928642-45932204

AAAACATCTCCTTCGAAAACCCTACGTCTTCGTCTTCAAGTCCTCTCCAACTCCTCTGTCTCTCTCTATATGGAAAAAAAATCCAAATTGCAGTGGCAAATCAGCACACCATGACCATCTCTCTTTCTCTCTCGTGGGCAGAAAACCCCCAAATCGTAGAATGAGAAAAAACCCCAAATCCCAGCAAAGAAGGCGGTGATAGCTGCAAGGAATGGTGTGGGCTTTCTTCAAATCTCTAATAGGATGTATTCTGGCGTCCTCACTTTGCATCCTCTCTCTCTCTCTCTCTCTCTCTCTCTAGGAAGGATTGACTGCTTTGATTGAAAGCCGGTTGTAGGACTTCTTCTCACTGTGGAACCCACCGGGATTCATCCAAAACCAAATCTATCTCTTCTTCAAGATATTTTTGTTCCAACTTACAAGCTTCTAGAGATTGATTTTGGATATTATTGTGCTGGCTGCGGGCTCTTTAGGGATAACTTTTTTTTATTTTGATGACAGACATTTCTATATGTTAAAGATTTTTGAAAATTATCAAGTAGTAATATTTATCTATTTTAGAGATTTGTGGGTTTATTTTGGTCTATGGATGTAGGATATTTGGTTGTGGATGTTATTTTCGATTTGTTAATGAATCTATTTTAAATTGGGTTGTGCATC

>ONT.14993.2 LG10 63258708-63261565

ATTTTTCTCCCGTTTCTGCAACATTTCCAGCTGTGGCTGGAACCCAGTTCCAGCCACTCTTTCCCCTTTTTGTTTCCCATTTTCTTGAAGCCAAAATGGAGGAGTGATCTCACAACTTTCTTCCCCACCTTCTAAGCTTTCCAAAGATGTTATTTTCATGGATTTTGAGCAAAGGCAACTTCTCTTTTCAAGCTCTTCTTCTTGGTTCAAGGCTTTAAAGGGTTTTCATGGGAAAATTTGGGGGTTTTGGTGGGTTTGGAGCTAGAGACTTCTTTGAGGCTTTTGTGGAGTGATTTGTGCATCTTCTTATCTAAGGCAAGTTTCTTGTTCTTCCCTACTTTTCAGATGTGTGGATCGGGGGAGGCGATAGAGCCACCGGTCATCGTGAGAGCCTACTTAAGGATATCATATAGATGTCAAGGTGGGCAACAGAGTTCGTAACAACTGTTGGGATTAGTATTTGTTTAGACATTTTATTTTGGGTATGGCTTTTATTAGCCTTTTCTTGGGTTTTGTTAATGAAAATACTTTGTTTTAATTGTAACTTGAATATTCATGGGAAATAAGAATATCCTGTTAC

>ONT.12139.1 LG07 75422673-75424253

TCTCTCCATTCCCCATTTCCATCGCTGGCGCTCTCTCCTCTCTCTCTCTCCATTCTGCCAGGAACGATCGTCATCCTCGCGCGACTGGCACCCTCTCTCCCGTGACGAAAGACGATCGATCCCAATCGTCGTCTTTATCTTGCGATTACAAAATATAATCGTCATCGTCTCTCTCCTGCCAATGCTAGTAAGTGATGGATCCCTCTCCCGAAGAGCCGTTTCTCTCAAAGAGGCTCTTCCTCCTGATGGAGGACCCAATAATTGCCTCATGCCATGTTTAAGGTTCCTCTGCTGCGGTGGTCTATTTACAAGCTGCTGCCCACCTCTATTTGAGCCGGGACCTCCGCCACCATGACTAATCTAGCACTTATTTTCTTCCTCTCCTTTTCAGCTTTTTGGGAGGAAATTTACTTGAGTGCTTATAGGTGTTCTTTGGGTTGATAATATGGAGAGGGATGCTGTTAAGAAAAGATTTTTTTGATACATTATGTCAAACTTTTGGATCATAATTGCCAGTTTTTTTCTCCAAATTAACTATGCAATTGAATTGATTC

>ONT.5010.4 LG02 116691218-116695139

ATCTTTCTAGGGTTCCTCGTTGCCTTTTCTTTCTTGTTTCTGTTTTTCTTAGATCTGCTCGTTTTCTTTCGTCTTTCGGCTGGTATGCAGTCTATTTCGTATTGATCTATCGCCGATCTACCTATTGATAGTCGATTTTCCTTCTGATTTGCACTTAGCCCTTTGTTTATTGGTTGTTTTGCAGCAGATCTAAGGTATTTCAACGTTTCGATCCTCTTCTTCTTTGTTTCTTCTTGGTTTTCGCGGTTTCTTTGCCTAATCTTCTTTGATTTTTCTTGAAATCGTATGGTAAACGAGGTCTAGATTTGTCATACCTTTCTTTTTACTGTTTGTGTTTGTCGATCTTCCTTTCGTTTTCAGATTTGAGGTAGGCTTGTTCTTTTTGTTGTAACTTAGGGTCTGGTGCTTGCGATTGCAATATTTCTTCTTTTTTATGGTTGTTTTGAAGCAGTCTGACGGATCAAGACTCATAAGGCGATGGAGGGGATTCCAGTTTCGCAGATCGTAGTCTCTTATAGCAGTAAAGAGACTCCTTTCGCAAGATCCGTTGCATTTTGGCGTTCCTCGTCGACGATTTCATCGGATCCATGGGTTTTTTTAGCGATCTTTCATCAGATCTACTATGTATGGATGTAAAGAAAAGTAGTTTAGTGTGAAGATCGAGGAAAAGCTTGTTATGTATGAAGCTGATAGAGAAATAGGTATGGATTAAGCGAGAGAGAGGCTTGTGGCAATGGATCTATGTCATCCTCCGACCTTTGCCATGTTGGGTGCGCTCGCATGGCAGGTCAAAAAAGCCTAATAAAATTCTCGATTTTTGGTTCTCGGGGGAGTTCGCATGGGCACTTTCGCCTGGGTTCTCCCCCTCCTGTGTGTCCTCTCTTTCTTTTCGTTTCTCCATCTCTTTCTTTCTGTGCGTTTGATTAGCAATATCTGCAACTCGACATCACGCTTGCGAAACCGAGCAGAAGAAAGCTACGATGTGGAAATCTCTCGTTATATCATGGCAACATCACAAAGGGCAGTTGGGTAATTGTCTGCTTCCAAGTTTAGTCTTGTTTGGGGAGAGAGCATATGCAACGGTGGAGCAGCTGATCAGCAAGAGGAAAGGTGAAGGTGTATTCTTTGTTGCTGCTGGACGTGACTGATAGGAGGCTCCTTGGGGATTGGATGATTTCTTTGCTGTTGGTGGACCTGAAGAACAGTAGTGACGGAATAACTTTGGTTCGATGAATTCTTTGCTGCTGGTTGACCCGTTGATGGTTCACATTGCAATCCTCGGTTGGATGAATTCTTTGGATGTGGACGACATAGGGCAACGGGGATGAACTGTGTTTTATCAATTCATTTCTGCTTTTGCATCTTTGCCTTCCAGACAGACCCTCTCACCCGTAGGAGTTTAAAAAATCAATGCACGAGCAGGCTTATTATGCTGGTTCCTTGTTCGTAGGTTATGGCCAAACCCATGGCGTTTGTTTTCCTTCAAATAATATGCCTCTCGACAATGCTTTAACTAACAGGTCGGCGGCTTTTTACCACATGGTGGTGCAGTGGAGTTTGCGCGCTATCTATTCAAGCTTTGGAGTTCGTCTTTTGTTCTGGACTGTTTTTATCATGCGTTTCCAGAGTTGTTGCTGAATATTATTCCAGACTTCTTTTCTCTGATTTCACGGAATTATGGTGGGCTGATTGGTAATCATACTTCTCATAGAACTGCAATCTGCTTTTAACTACAAATTTTATTTTTACATGTAGCGTTCATTCGTTTATATTCATATTATGCACTGGTATCTTTTTTCTTTATAGTGTTTCTCAAGGGTGGTTGTGGTTGTGGTTGTATATATTTTTTTTTCATGTGGTAGAAGAAAATGAAGTT

>ONT.17228.2 LG12 43714271-43715139

CCAGATCAGTTCCAATTTCCATACACACAAATGAAATGATGGCTTCTGCATTTGCTCCTCCTTCTCCACTTCTCAACTTCTTTCTCCCTCCACATTCTATCTCTTCTCCTCAAACCTTCAAAACCTCCCTCCCATCCTCTTATCATTCAATCTTCTCCCTCAAATACCCACCAAACAAAATTGTCCTCTACCCATATTCAACACAGAGAAAATTTTCACAAAAAAAGACACAGTATTGGAGAATTCTTGCAGCTACTGGAGATGTTCTTCCTCCAAACACCCCAATAGAGGAAACCCAACAGATTGTTCCCCCTGATGACAGCAGTGGCTCCACCATAATCTCTGTTCTTCTGCTCATTGCTTTTGTTGGCCTTTCTATCCTTACCATAGGGGTAAAGATGCCATAGCTGTGACTGATTTCTTGCAGAAAAGGGAGAGGGAGAAGTTTGAGAAAGAGGAGGCTGCAAAGAAGAAAAGGAGTGGGAAGAAAGGGAAGGTGAAAGCTAGGACTGGCCCAAGGGGATTTGGGCAGAAAGTAGAAGATGATGATGATTAGTTTTTCAAGTGTAAGCATCTCTCTTTCTGGTGTTGTTTCCTTATTTATCTTCTTTTCCAATTAGATGTGGAGATTAAATGAAAACTCCTACGATTCAATGTTCCTGACATGTAAAAGCTTGAATTTAATTTCCTATGTTGGAAGTTTCTTGAAAATAATGACATTTAGTTCTCAGAAATATATTTCATTTTTGTTGGTTT

>ONT.10402.1 LG06 69016114-69017149

CTCCAGGTTTTTCACAGCCAGTGTGATTTCAGCGGCATAGTCTCCAGTTTTTCACAACCTGTGTGATTTCAGCGGCATAGCCATGGGCATACATTTCAGTTGTTGGAGGCCTAGAGAGCCACCGGAACCACCACAGACTACCCAAACTACTAGCTATACCCAGACTGGTGGTGGCAGCTACAATCCTCAAATTCAAGGAGTTAATATAGCAGATGGTGGGGGAAAGATTAATATGGGACCTGTTATTAGTGGAGGAAGTACCTTCACCATTTAGCATATTTAAGGATTTCATTACTCTCTGCTGTTGTTCTCAAAGTTCAGAAATTACTAACTGGGAAAAGAAAGAATTGGACCCCTGGGAGTGGGAGCATACATGACACTTCTGGCCTGTTATTTGTTTGCTATTTGATGCTCATCTTTTGATATTGATTCATTAACCACCCTCTGTATTCAATCCCTATGAATTTTAATGCAAATAAATTAAATTAGTTTTCCACTTGTTTTATTTGTCTTTCTTTAACCATCCTTTGTATTCAATCCCTATGCATTTTAATGCAAATAAATAGAATTAGTTTT

>ONT.1502.1 LG01 109557383-109558991

TCGTTCGCTTGACTTTTATTAGATTAGAGGAGATTTAACAGAATCGTTGGTTCTGAGATCTCTCTCTCTAGGCTACACTACAGCACCATGAATTTCCCCCATTAGCCGCTCCCAGTTGCTGAGATTATTCCGGGCCTTTTTGGTATTCGCATGCGTCTACGTATGCGCCGGATTTCTCTATGACATCCTACTGCTTATCTTGAAGAGATTTCTAGGAAGCATGTACTGATGATTTCTACTCCACTGTAACTGGACTGTGGGTGGACAGGAATGGGACGATGACCAGCCACTAGAGTTTCATGGCTGAAAAGAGACGCGATTCAAGACTCAGTAGTTCAAGCCTCAAGGGGCTAGCCCTGTAGTTAGGAGTTATAATTTTAGCCAACTTTAAATGATAGTTATGTTATGGGACTTCTGATGATTGTTTGAGACATTGTTACTCTTTATTATTGGTTTGGGATTTTAATAAAAGATTGGGTTTTAGTAGGTAA

>ONT.1154.4 LG01 24839380-24845636

GTCCATTCACTTCTTCCTCAAGACAGGCACAGAGAGAGAAGGTTACTTTGAGGATAGGAGGCCTGCTTCTAACATGGATCCATACGTAGTGACTTCCATGATCGCTGAAACAACGATCCTTGGGAAGCCATAAGCAAAATAATCCACTCCTCTGCTGTGTTTGTTCTCTTCATATCTTTAGATGCCATGGCCTTTGCTGTCTCTTTCATCTTTGGTGTTTGAAAGCATTGCAGTGCTTTAGGGACATATTCATATCATTGGCGATTGCAATTGTCTGTTTTCTAGTTGTGGTTTAGAGGACTTAGTCCTTTTGGGGGGTGTTTTGAGGTAGGCAGGTGGGAGAATCTATGTTGCTTGCCATTTGATTATGCCTCATGTGCAATAACATTTTAGCAATAATAATTTAAAGGCCTATATTACATCA

>ONT.12370.22 LG08 13412664-13417389

ACGGATGCTGGCCTTCTTCTCCGGCTGTTCTGCAACAAGGATGCTCGCCCAAATCTGACGAGGAGAGCAGCAGAAACCCGCCCTACTTCTCCGGTGAGGTGATTCTCTCTGTTTTGCGAAAAGGATTCGCTCAGACGCTGGCCCTTCTTCTTCGGCGACGACAGATCTCCAGACATCCCAGGCATATTGACTGTTTGTTTGTTGTTTTTCTCTAATCTGCAAAATGCCCTGTTCTGCACCCGAACCAAACACGAGCCAATCTCCAACCGTGAGATCAGATTTGCAACAGCAAGTGGCCGGCTGTGAGATCAAATCTGCAATTGCCAGTGGCCCATCGTGAGTCTTCTTATTTTTTATTTGTCTCGAACAATCATCCCCACCGTAGCTTGGAGATGATGTTAATCCAAATGATGCAGTTGTTTTAGTCTTATTTTTATGGAGTATTCTACTAAATGGATTGCATTAGTGGTTCGGCTTATATGAAAATTTCTGTTAAAAACGTTTGTAATCAAAGTATTAAATCATCAAACCTACTGTTTTCAGCAAAAATACCTTTTGTGTTATAATAGGGGAGTCGAAAGTTGATTGTGTTAGTTATTGACCTTATTTTTCCAAGGCATTTGGGTGTGGGAAGGTGACTGCCCATGTTCTAACCCTAGGCAGCATGGTAAGAACAAGGCACCTCTTAAATGTAGTTGAACACAATACATGTGGCATCTCTATGGAGGCTTTATCTTTTGAATGTTTTGATGTTCTTATATCTCCAAATCCATGAATATGGTCGCGCATAATTTTGCAAAAAAATACTTACATGGGTGACGGGGTTAGGATGTCTTCCATTTTCTAGTTTGTAGTGCTCTTTGACTGGTTGTTTCTTGTAATTCTTTTTCCTAGTGTTCTCTGTTCATTTGTTTGCTTTTAATGAATTCTTCTTTTACCAAAAA

>ONT.13271.1 LG08 68418830-68422394

AATCAGAAGGATCCAGAAATCGTCAGCAATCCTTGCTCTAGAGATCCAGAAGCTCAGAAAGAGAGAAACCTCTGTCGAAATTCCGCAGTAGCCTCCACTACAAGGTGAGTACTACTACGAGGAGCAGGCCGGTGATAAGATCCCAGAGGATCTCCAACCAGCAGAGGAGGCACGGGAGTTGAGTGCTGATGACGTAGAGTAGCGTCATCATTACCAGGCACATGAGAGACTTACTTTGGGACAAACATTAGATTTGATTTAATTCATTGTTATTCTTCATTGAACTTTTCTTTCATTGACTCTATGTTGTTTAATAGCCTTAGTTTTTGGGATGGCCATTGTAATAAATTAACTTCATTAGTTAATTTTATTATTTATTTATGCAACTTTAATTATTAATGAGATTTATTATTCATTACTTTGAAATTTAA

>ONT.9542.4 LG05 41695556-41705604

GCGCTCAAGCTCTCTCGGTGCCCGGCGTTCATGGCTTGGGTACTCTGCACCCCTTTCTCAAGACCCGCGGTCTGTACTTCAGCAACCAAAACAGAGGAGCTTGAGCGATTGTTATATCTGATGTTAGGCAATTGTTCTGATGTTGGCGTTGTAGGAACCACCAACGGCCAAAGGGTTTGCCTTTGAACAGAGTTTCAAGCCTAATAGCTGCCAGGCCTGTGGTGAGAATGGTGACATCCAACAGTGCGATTAGAGGCATATGTAGTGTGAACAGGGAGTTGTTGAAGCCGGGCAGACATCCTTGTGGACCTGATTATAGTTGCCCAATTATTTTTTCTACTATCCATATAACAATATCTGGTTACTGGGTGGGCCCTGATATTGATGATGGGTGGGGATATGTGGAGGCTTCTGTGAATCAAGTTTCTGTTTGTTGACATGTTGTTTAAGTTGTCTCTTTTACAAGTTAATGAGCATTACATGAATTTACTGTTT

>ONT.4620.2 LG02 38077415-38082738

TTTTACCCTGCTCTCTTCTCCCGCGCGCTTTGCATCTCCGATTCTCCGTCTCCCGCGCTCTCCATCGCCTCCAACCCTCATCTCTCTCTCTCTCTCTCTCTCTCCCTTTCCTCTCCCTCTTCTTCTCTCTTCTCTCCCTCTCCTTCCATTCTCTCCCTGCGTTCACCCTCTCTGCCCAGCCGCCAGCGCCCGCGCTCTGCATCTCCGTCTCGCCTCCAACCCTCGATCATCTCTCTCTCTCTCTCCCTTTCCTTTCTCTCTCTCTCCTCTTCTCTCTTCTCTTCCTCTCCTTCCCTTCTCTCCCTGCGTTCAACCTCTCTGCCCAGCTGCCAGCGCCCTGCTTCTCCCATCCCACTGCCTCTCTCTCTCTCTCTCACTTCATTTCGACATCTCATCATTCCTCTTTTCCCAACCAAATCGCTTTCTCACTTCGCACTGAATCGGAAACTGATTCGAACGAATGTCGTCCTTCTTCTTCAGATCTCGAGATCTGGTTTTCTAGGGTTAGGGTTTCCTCATTTCCACTGAAGAAAAGGGAAAACAAACGCAACATCTACTTCAGTTTCCCTTCTTTCCATCTCTGACTTCGAAGAGGATGTTCAAGAATTTGAGCTTATCGAACGCGGGAATTCTGAAGTCTTGAACTGCAATAAGAGAGAACTGTTATCTGATCAGTTTTGTTCCCCTTGCTATAAATCGAGTACTCATTTGTGGGTCTTTCGCAAAAATTGTAGCATCAGACGCTGGCAGCGGCCACAGGGCTAGACATGACTTCAGTGGAGAGGGCAGTCATGATTTAGCAGGCTCAATTGATCCTGACCAGGTTTGAGAGCATCGGATCTACCACGGCACCTGCACGGACAAATTTTTTTTCCTGTAATTGTAATGGATATAATTTATTGAAACTTTTGTAATGTTTTAAATGTAATTAATATAAGACTTTATTTTGTTA

>ONT.6376.1 LG04 592916-597756

ACTCACTAGAGCAGAGCTCTCTCTTTCCCGGCATTCACGAGCGGGACAAGGGGGCTTGGAAGAGGACGAATAACTCCAACATCTTCTTCCATGCATCGATCGTCTGCATCCTCCACCGCACTACATCCATTGGTGCCTGCCAGAAAAACCCCAAATCCCAAAAAGCCCACTCTCTCTTCCCTTTCTCTTCTTTCTTTGTCCACGCCTAACACGCCCTTCGTTCCCCGGAGGAAGGATTGACTACTCTGATTGAAAGCCGGTTGTAGGACTTCTTCTCACTGTGGAACCCACCTGGATTCATCCAAAACCAAATCTATCTCTTCTTCAAGATATTTTTGTTCCAACTTACAAGCTTCTAAAGATTGATTTTGGATATTATTGTGCTGGCTGCGGGCTCTTAGGGATAACTTTTTTTATTTTGATCACAGACATTTGTATATGTTAAAGATTTTTGGAAATTATCAAGTAGTAATATTTGTCTATTTTAGAGATTTGTGGGTTTATTTTGGTCTATGGATGTAGGATATTTGGTTGTGAATGTTATTTTCAATTTGTTAATGAATCTATTTTAAATTGGGTTGTGCATC

>ONT.16121.3 LG11 10056347-10077789

ATCCATTTCCCTGCAACGGTTCCAGCAAGAGCTGGAACCACTCCTCTCTCTCAGCCACTCTCTCTCTCCTCTCTCCCTTGGCTTCTCTTGATTGTAAATGGAGATTTGATCTCCTCATCATCTTCCATACCTTCCAAGCTTTCCAAAGATGTTATTTTCATGGATTTTGAGCAAAGGCAACTTCTCTTTTCAAGCTATTCTTCTTGGTTCAAGGCCTTAAAGGTTTCTTGTTCTTCCCTGCTTTTCAGATGTGTGGATCGGGGGAGGCGGTAGAGCCACCGGTCATCGTGAGAGCCTACTTAAGGATATCATATAGATGTCAAGGTTCAGACTTCTTTGCCCCGAGTTGAGAGTTTTTGATTGGCTATGGAGCCATGTCTCGCAGGTTGTTTCGTCGGCTATGTCATATAGACATTGGGCTTGACACCGGAGCTACGTCACATTTGGGTAATAGTTTATAGCTTATTGTTGTATGGCTTTTGGTAGCCTTCTCTTGGGTTTCTTAATTAATGTACGTTGTTAATTTATAACTTGAATACATTTGGGAATGAAATATCATGTTACTGT

>ONT.9740.2 LG05 92035201-92049842

CTCCCAGAGCTTATCATTTCTTCCCTTTTTCCTATTTTTCCTCTCCCACATTTTTCTCTCTTTCTCCTATGCTCCCTGCTCTCTCTTTCCCTCTACCTACGTCTCGGCTGGGCTGCTGTTCTCTCTTTCCCTCTCCCTCTTTGCTCCTTGCTCTCTAAGAGATTCTGGTCAGATCCTCCTGTGAATCCGCCGACTGCTCTTCTTCCATTTGAGATTTCAAAAAAAAAAAAAAAAAAACTCAGTGATAAAAGAGACTTCACCGTACGTCGATCCAAACTCACCACTCAAGTGCATTTGCTGAGAAACTAGTAAGTTTACCATTGTGCTCATTCTATTCCACCACTACTTGCAGGAGGGTGAGGATTTGCTAGAGCCTGGGATTGAGGGTGATGAGGATTGCGAACTTGTGCCACAGTATGACTATAATGATGACATTCTAGATGGTCAAAATGAAGATGTTTAATCTATGAGGATTTAAGACATTAAAGTTAATTATGTTTAAGACTTTATTAGTTTATATTGGGATTGATGTAATTAATTAATCTCGTACTTGGTTCACTTGGAAGTTTTTTTTGGTGATGTAACAATTGATGGATTGTTATTTATGGTTA

>ONT.13402.1 LG09 41228287-41239430

GCAGTTTGGGATGTGGGAGGCTGCTGAAGCACATCACCTCTCCAACCATGCTGGTCTGATAAAACTGAGATCTGGTGATATGGGTGTGATGGAATTGACAACCTGATTTTTCTCACTTCTGATTTACTTTGTTGGACTACATCTGCAACATTGACACATTCATTTCAGTCATCGTGTTATCCCTACCCACACATTCCTCCCAGTAGCCACAGCTGCCCATGCCGGTCTTTCTGAGACCTCAGTGCCAGCGGAGGAAACCACTCCGGTTCCTCTGGGAGGATTAGTTGAGTGGAAATGAGGCGATAACCTTTGATATGAATTTTATAGTGAAAACTTTTGATAATGGAGTTCGTTTTGGAAACCTTTCATACTTTTGGTTCTTGCATTATATTTAAATATGCAAGTGATTTGTATTGTACATTGTGTAAATGAAATTTTGTTTTTATACATGAACAATGTATATTTGT

>ONT.4598.1 LG02 36092777-36094618

GGAAAAAGACTCGCCCAAATCAATTGGAAGCCCCAAATCGGACGATCTCTTCCTCCTCCTTCCGACGCATCTCTTAATCCTCATCTAAGCAGGATTCCGCCGAGAGAGTCATCTTCCTCCTCCTCCCCTCTCGTCGACGTCTCTCCCTCGTCTCCATCACTTCCGTCGTGGAGCCATCCCATATCCTAGGGCTCGATCTCCCTCCGCACCAAAGCAGAACATCGATAGGGGGACGATCTCATCCTCGATGAGCTCTGCATCCCCTTTTGCCTGTCATCTCTGTCGCAGCACTCCCGGCCTAAACCAGAATGTCATCTTCTAATACAATCCGTTCTGAATGTACTCCAAAATGCCGATGTGAAGCTGGAGACATGAAATTATATACTTCTCGTACGCAATTGAATCCAAATAGAAAATTTTGGAAATGCCCAAATTGGAATGATGAAAGTGGCTATGGTACATTTCTTTGGAAAGATGAAATAGTGAATTGGCAGCTTGGTGAAGCTAATTCAACGATGTGTGAAAGAGAAAACTAGAGAGACTAGAAAAGAGAAATTTGATAGCGGTCATTGACAAGTTAGAAAGTTCAGTGTGTGCTTTCATTGAAGTAGAGAAAATTCGAAATGCGTATCTGCGAAAAATTTATCATGCTATTTTAGTTGCATGCTGTATAATAATTTTAGTTTTCTTGTTCAACCATACATGAAATACATGCTATGGGGATATGTTTTCTCTAAAGGTTTAAAAACTTGTCTTCTGTATGTTGGAACATAGAAGTTATGAATGGGCATATGTTCTCTATGAATGT

>ONT.17171.1 LG12 40491973-40493129

ACATAAAGACCAAAATAAAACAAGTGCCTCATTTCAGAGCAAAAGATAAGGGAAGGTTAATGCCATGCACACATTCATCACAAATGTTTCCAACTCCATGTCGACGATCGGAATATTTGGCGGGGATCGAATCTTCACCCTTGAATTGATAATGGTCCACAAATCATTGTCACTATAGGTGCTATCCTACCTGAGCTTCTCTATTTCTCCACCCTATTCATCTTCCCTGCCTTCCTTTTCCAACTTTCCTCCTCCGCACTCTTTCCATCCTTTCTCCTCCTTCTCAGCAATATGCTTTTCTTTTCTTGACCTTGTAAGGCCTTTTCTTGACCTTGTAAGACCCTACGTCAGAGACGTTATCTTAGTACTACGCGATATCATGAATCCTCAATCAGGCGGTTTTGATTGAGAGCGTAGGTTCTACTACTATTCCATCTCAGCCATCCATTCTTCTCTACCTCTATCTATCCCTCCTGAGCTATTCAAACCCAACCACATTTCCGGCTAAACCAATCAAGAACATGGGTTCTCTCCTATATGCAAAGTTGCAGAGGAACAAGAAGAGAGTGAGACAGTCGTCTAATTTCTAATCCCTGGTTTCTTTGTGCGATTAGTGCTTGTTTTGTATTATTTGTGCAGAAATATGCTTTGCAGGCATCAGTTTCGTTGTTTCATGTGTTGTTTGGATGAGCAGACAGTAAATATTCTCTTAACTCGCAATGCAATGTCACCTCGTGACTGTTCATTAGAGTGTTGCTGTTACTTTCTGTTGTTCTCTTCTCATATTTGTTTTTGTTGCTTACCGTATTTCAAGAAAGAACATAAATTTTGTACATGACATGAGCGATTTGACTGTCTCGAAATAGCTTTTGAGATAGATTCACCAGTTTCTTCTTGTCATCT

>ONT.5010.1 LG02 116690992-116695141

ACATCTTTCTAGGGTTCCTCGTTGCCTTTTCTTTCTTGTTTCTGTTTTTCTTAGATCTGCTCGTTTTCTTTCGTCTTTCGGCTGGTATGCAGTCTATTTCGTATTGATCTATCGCCGATCTACCTATTGATAGTCGATTTTCCTTCTGATTTGCACTTAGCCCTTTGTTTATTGGTTGTTTTGCAGCAGATCTAAGGTATTTCAACGTTTCGATCCTCTTCTTCTTTGTTTCTTCTTGGTTTTCGCGGTTTCTTTGCCTAATCTTCTTTGATTTTTCTTGAAATCGTATGGTAAACGAGGTCTAGATTTGTCATACCTTTCTTTTTACTGTTTGTGTTTGTCGATCTTCCTTTCGTTTTCAGATTTGAGGTAGGCTTGTTCTTTTTGTTGTAACTTAGGGTCTGGTGCTTGCGATTGCAATATTTCTTCTTTTTTATGGTTGTTTTGAAGCAGTCTGACGGATCAAGACTCATAAGGCGATGGAGGGGATTCCAGTTTCGCAGATCGTAGTCTCTTATAGCAGTAAAGAGACTCCTTTCGCAAGATCCGTTGCATTTTGGCGTTCCTCGTCGACGATTTCATCGGATCCATGGGTTTTTTTAGCGATCTTTCATCAGATCTACTATGTATGGATGTAAAGAAAAGTAGTTTAGTGTGAAGATCGAGGAAAAGCTTGTTATGTATGAAGCTGATAGAGAAATAGGTATGGATTAAGCGAGAGAGAGGCTTGTGGCAATGGATCTATGTCATCCTCCGACCTTTGCCATGTTGGGTGCGCTCGCATGGCAGGTCAAAAAAGCCTAATAAAATTCTCGATTTTTGGTTCTCGGGGGAGTTCGCATGGGCACTTTCGCCTGGGTTCTCCCCCTCCTGTGTGTCCTCTCTTTCTTTTCGTTTCTCCATCTCTTTCTTTCTGTGCGTTTGATTAGCAATATCTGCAACTCGACATCACGCTTGCGAAACCGAGCAGAAGAAAGCTACGATGTGGAAATCTCTCGTTATATCATGGCAACATCACAAAGTCTTGTTTGGGGAGAGAGCATATGCAACGGTGGAGCAGCTGATCAGCAAGAGGAAAGGTGAAGGTGTATTCTTTGTTGCTGCTGGACGTGACTGATAGGAGGCTCCTTGGGGATTGGATGATTTCTTTGCTGTTGGTGGACCTGAAGAACAGTAGTGACGGAATAACTTTGGTTCGATGAATTCTTTGCTGCTGGTTGACCCGTTGATGGTTCACATTGCAATCCTCGGTTGGATGAATTCTTTGGATGTGGACGACATAGGGCAACGGGGATGAACTGTGTTTTATCAATTCATTTCTGCTTTTGCATCTTTGCCTTCCAGACAGACCCTCTCACCCGTAGGAGTTTAAAAAATCAATGCACGAGCAGGCTTATTATGCTGGTTCCTTGTTCGTAGGTTATGGCCAAACCCATGGCGTTTGTTTTCCTTCAAATAATATGCCTCTCGACAATGCTTTAACTAACAGGTCGGCGGCTTTTTACCACATGGTGGTGCAGTGGAGTTTGCGCGCTATCTATTCAAGCTTTGGAGTTCGTCTTTTGTTCTGGACTGTTTTTATCATGCGTTTCCAGAGTTGTTGCTGAATATTATTCCAGACTTCTTTTCTCTGATTTCACGGAATTATGGTGGGCTGATTGGTAATCATACTTCTCATAGAACTGCAATCTGCTTTTAACTACAAATTTTATTTTTACATGTAGCGTTCATTCGTTTATATTCATATTATGCACTGGTATCTTTTTTCTTTATAGTGTTTCTCAAGGGTGGTTGTGGTTGTGGTTGTATATATTTTTTTTTCATGTGGTAGAAGAAAATGAAGTTATATGTATATTAATCATTTTTTCCTTGTGCTGGGGGAATATGTTTTTTTAGAAAAGATATATACATGTAAAAACCAGGCAAGGAGTGAAATGGAGGCAAGGAGTGAAATGGGAAGGTTTAATGTACTGCTGTCGAAGATCTTATTGCTGTACCTTTTTTCCTGTTTTGTTGTATTTTTGAAACTCTGCGTCGGGTTATTATTATATTCCTTAATCTGACCCTTCCC

>ONT.8024.2 LG05 9574849-9576633

GTCTCAGTCCCAGCAGAAAACCCACCCAAAAATCCAATCCCTCGTCCATCTCTCCGCCAATACGAACTCCGCAAAGTCCTCAAAATCTATATATCCTTTCGAGAACCCTAGAAATTGCCTTCTCCAGACTCTCTGCATCAGAACTGGAGCTCGAAAACAATGGAGTTCTGCGGCCCTACCAAGGGGCTGGGAACCAAAAGAGAGTCTGCACACATACGGGAAGGTAGCCCACCAAGTGGGAAGGAGGCCCGCACAAGTGGGAAGAGGTGACATACCTAGGGTTCAATCCTGGGCAGCCAGGTAAAAACTGACGCTCCCTACCACCCGGGTAAGGAGTTGTTCTTAGTCCCATTTTTAATTGAATCTTATGATTCAACTATGAATATTACAATTTGATACTTATTCTAATAGAACTTATGATTTAAACATTTCATTTGAGTGAGAATAGCCACAATTGAATTGTTAATGTTTCCTTCTCTTTTTTTTATTTTCTAATTTTAAAAGGTTGGTTATCTAGTGATTAATGTAACTACTTATTTTTTATTTTATTTTTGGGAGGTTATTTATT

>ONT.15779.9 LG10 31020787-31022132

TCGAGGAGGGAAAAAAGCCCTCTTTTTTTTTTCGTTCTTCCTCTGCGCGAGAGTGAGAGAGAGTAGAAGACGAAGATCGAGAATTCCTTCGAAATTTCTTCGATTCGATCGATTTCAGAGCTCAAATCCTTCTGAGTTGCGGCCCATTTGAGTCATTGGGGGCTGTCTGAGCATTCCAGGGTTGGTTGTTTCAGGGAGCATTGTCGTCACTTCTCTTTTACTTTGACCTTTTGTCTGTCAAAACTTAAATCTAAAATGGCTGAGTCGGAGACGGCATCAGAAGATGTTGCTGCTGCAGTGCCAGATAATCAAACGGGTCCGCCCAAAGATGCTGGGGAAACCATTGTTGAA

>ONT.12910.1 LG08 20580686-20582601

ACATTTCCCCATTTCAGCTGCAGCACCGACCACCACGTGCAGAGGTTTCATCTCTCCCTCTCTCGTCGAGATCTCACTCTCTCTCCCTGCTGCCTCTCTCATCGAGATCTCTCTCTCTCTCCCTGTAGCCTCTCTCATCGAATCTCAGATCGTCTCGCTACTTGCAGTCATTTTCAACTGTTGATGCAAAGTCCCGCCAAGATCTGACATTGGCGGCATCACGAGCGGCCCCCATTTCTGCTCGTCTTCTCGACCTCACTACTGCTTTCCCCCATTTATCGTTTTCCTCCCTTCTTTCCATTTGAGAAAAAAAAAAAAAAAAAAAAAAAAAAAAAAAAAAAAAACAAAGCGAGTGACAGAGAAATCTTGCGATCTCCTCCCTTCTTGCAATCTCCTAAGTTTCTCAAGAGATAGCATCAATGGCATCTCTACAGCAAAGCTTTGATTCCTTAATTTGTGAAGTATATGTGCTATGGATGATAGTAATGTTTATTCACAGTGGTTGGAAATGAGGTTGGTACTAAAAGCACTCCATGAACAACATCAACTCATACAGAAGCTCTAAACTCTATGAATGTTCGATAACGTTCTAGAAATGGTGGTTCGTTCTAGAAATGGTGATTGAGCGATTGAGCTCACAACGTCTTCTTTCAATCTGTTGACTGGCTTTTTGGAGAGGAATGAATGGAATGGGAAGGAGAAGGCAACAGAGACATGTAGTTTGACTATTTAGTAATCTTATAATGAACCCATTGACAGATTCTTATTTATTTCGTGTATTGGAGTTTAAGGACACCTGAAGACCAAGAGTTATGATACGTAAATAATGATTGTATTAATTTGTAATATTGTAATTTATAATTGGAACAGATTTTAGTTAATTGAAAATAGTTTATCAAACTAA

>ONT.10651.2 LG06 27906867-27909331

ATGTTGCACGGCCAGAACTCCTTCCAGCCGCCTCCTCTCCTTCCTCTCTTAATCTTCCACCGAAAGATCATGATACTAAAGGTGTTTCTCATCAAATCCAAGCCTAGGGAGACGTTTTCCATCATCTTCTTGCTTCCTCTCCATCGTTTTGGTGGAGAACTGCACGTGGCAGCCCCTTAACTACACTTCAACTTCTTCATTGGGATCTCTAGAGATCTGGGATCTTCTTGCGGGGATAGTTTTCTGCGATTTCTGGTTGTGTGAGAAGGTTTTTGGATTTGCTTGGAGCTCAAGGCAAGTATCGAACTCCTTGTGCCCAAACAGGACATTGTGGCGGTCCAACATGATCCGCCGTTTGTACGAGTGGTAAATTGATGACATAGAGTAGATCGTCTGAATCTGCAGAGTCACGATAATATGTTGGGTTTTATCTTAGGGGTTTTACTTTATGGGCTTTAATAATAGACTTTGGAGATTGTTTAGTTTGTGAACGTTGGCTAATGTTTAGGCCTTTTTATTTGAGACTTTCAATTAAGATTTATTCCTCTTTTTATTTCTGTTTAAACTTGAATCTTAAGTTATGGAAATAAGTTTCATTCTGACATCAGTCTATTAATGC

>ONT.3520.2 LG02 65432168-65439590

GCTATTAAGACGATATTGCCATCAGTCTCTGCTCTCTTCCTCATCGATCCCTCCCTCGCTCTCTCCCTGCTCACGCCATCTCTCCCTGCTCACGCCATAAGTCTCTGTTCTCTCCCTGCTCATGCATCGTTCTCTTTGCTCGACGCCGAAAGAGATCCCTCTCTTCCTCATCGTCGATCCCTCTCTTGCTCTCTCCCTACTCACGCCATCCCTCTCTGCTCTCTCCCTGCTCACGCATCCCTCTCTTCCTCATCGATTTTCCTTATCGTGGATATAAAGCAACAAAATCGTCCCCCCAAAAAATCTATCATCTAGGGTTTTCTCTCGAGCTCTTCGTCTTTCGTTTCAGGGAAGGAGGAAATGTTACCCACTCCTGAAGGTATTCAATCAAATGATGCTTTATTACCAAAGGATATATAGGCAATTCCATTCAGACCGCTCTCTCAAAGACTCAGATGATTGCCTGATGACTGAATGATGAAGATGAAGTGAGAGAGGAAGGAGGAGAGAGTGACTTGTGGGATAGAATCTTTATGAGAGACATCCAGAGGGCGGGCCACGCACCGTGCAGAAGGTGTTGAGTCCCACCAAAGTTTTCCAATGACAGTTCTTTATGCAATAAAACAATGTGTCCACAAAATGAGCATGTGCTCTACCAAACTGAAGCTTGCAGTTGGGAGGTTCCCATGGTCCTCATGTTCATGGATTATAAACATCAACTTTGCCGGTTAATTGACACCAAAAGAGTTTATAATCTTCATAAACAGCTCTTCCATCACAACTGAGGAAGATCTCCATGAGGAAAAAAAGACGACTGACATTCTTCAGCTTGGGCATAGGGATAGTGGCAGCTCCTTGAGTTAGAGCAACTAAAGCCCCGGACATATCAACATCTTGGAGTTCACCCTTTTCTTGCATGTACTCAAGAGGTAGTAAAATTAGATCAAAGACGTGCAACTTATCCAAGGATTTGATTCAAGATATAGATCGGAATGGAGAAATAAAATTCATGTAGCCGACCCCAATATAGGTGGGACAAGTCTTTGATGATGGTGATGATGAAATAACTCTGAGGAAGTAGATCGTAGCTGCATCATTATAGCGTGTAAAAGATTATGGGCATAATTTTGGAACTATTATTATTGTTTTTATATTTTTAATTTGAATGGAATTTTGAAGTGAAGATTTTATAGTTTTCCTCCTAGGGATTACCTTGTTTATTTCGATCACAGACATTTGTGGATGTTAAAGATTTTGGGAAATGATCAAATAATAATATTTGTGTATTTTAGAGATT

>ONT.6742.1 LG04 28680842-28685861

GACCGTCTTGTCTATTGTAGGTGGCTTCCTCAAAACCCCTTCTGCATCTCCCTCCTTCTTGGTCTCTCTCACATCCTCTGCATCTCCGTCTCGTCCAGCCTCGAGCATGCGCCCATCTCGCCTCCATCCCTTATCTCTCTGACCCATCCTGGCCTCTCTCGCCCGCTATCCTCTCCATCGCCACGCAACGCAGAGAATCCACGATCCCCATCTTTCTCGATCCCTCTGTCTCCCTGCGCAGAGCACCACTCTCGTAGAACGCTGCACATCCCTCACCTCGCACCTCTCTTTTCTCCCTCGGCTTCTCTCTCTCACGCTCACAGTCTCCCTCGACAGCGCATCCTCTCTGTCCCTCTCTCGCAGCTGGACTCACACTCACAGATTGAATGAAAATTGAAAAACAACTTTTGTTGAAAGAGTCCATGTGGCGAAGATGCAGCCAACATGAAGGCCAAGTATGCAGAATAGAATGTTTGGCATTTGAATATTGGTACATAGTAGAATCCCTTTGTGGTGAATGACCACTTCTCTTACCTGGTGATTCTCCATTGTGTACTAGAAGATAAATCTGATTGGATTGACAAACCATTGTGTACTAGATATATGCTTTCTGCTTGTGTATGTTGGCTGCTCACCAATTTAGAAATCCTGCTGTGACTTTTCTCTTATCATGCAGCAGTCTTGTGAGTTGTAAACTGCATCATGGTTCTGGCCTGATTTTCAATTTACTAACCTGTTGTTTGAAGGCTTTCT

>ONT.249.4 Contig00712 5920-15670

AAAAATAACTTCCCCACCTTCTCTCTCTTACATGCACGCAGAACCTCGAAATCTCTGTAGCAATGGCGGCATGCGCGGCCGCGGCTCGTCAAGCCGCGTCTCTATCTCGGCTCTCATCTCCCAAATCGGCTGCTCAAGCCTCAAATCTAGTGCAAAGACGCCGTCTTGCGGGAGGTGGAGATCATTATGGATCCCCCAAGGTTCACTGCTGGCAGGACCCATTAACTCCATCTAGATGGAAGGAAGAGCATTTTGTGATTGTTTCTTTATCTGGTTGGGGGTTACTTTTCTATGGAGGCTACAAATTCTTCTCTGGTGGCAAGAAAGACAAAATTTCATAAAATCACAGGAGCACTCCACTAGGCTTGATGGTGCAGTGTGACAAGTTTCGTTGTTGAGTCACATTTGAAACTAAATTATTTTCCATAACTTTGCAATATGTAATGAACGTATTTTGGTTATGAGATGTCGATTTTGAGTGACTAGTTTACATAACCCCTCGTTTATGGTTGAAAAATGCTGAGACCATGTTGCATGTCCCGGTCATGTGGGCAGCCATTGTTGATCCTTTCTTATTTCTGAATGGTTGCAGTGTTCTACCTT

>ONT.5123.4 LG02 123638518-123639789

ATGAAGAAGCAGGGCAGATACCCATGGCGTATGTGGTCAGGAAAGCCGGCAGCACCCTTACTGAAGCACAAATCATGGATTTTATTGCCAAACAGGCATGTAGGTTGCACCATATAAAAAAATACGCCGAGTCGTATTCACCAACTCAATACCGAAATCAGCTGCAGGAAAGATCTTGAGAAGAGAGCTGATTAATCATGCCATTTCTAGTAGCACAAAGTCGAGATTATGATTCCAGTTGTATAATTACTTTGAATCCATAAAAGCAGCAGTAATTGGTGAGGTCATTCCATCCACTCAAGGCAGAGTAAGTTGCCCTTATTTCATGATGATGTTGCTTACAGTGGGACGGGTCCATTTAGAGGAACTATTACCCGGTCCGGAGGTGGCCGCAGTATAAAGGAGCATGCATTCTTTGTTTATAATCTGTTGTTAAGTACACCGTTACTTCTTAAAATGAGTAATTATTATCA

>ONT.3574.1 LG02 94237120-94256367

TCTCTGCAGCGCCGAACCCTTCTCTTCCAGAAGACGAAAACCCTCAACCTATCACATTTAGTGTTTTTGGTCGGTAGGTGACGGTTTGAACCGGACATTACGATGGATAAAAGTTGGATGCATAAACCAGAAGACAATTTTGAGGTAATCATTTCTAGGTGCGCGTAGTGATTTTGGAGAAAGGAATTCCTACTCAGTGTTTCTTTCACCTCTGCATATGTACTCCTCTGTTGTTGGACTGCTTACATGAAACAGAGAAAAGCAACATAGAAAAGTTGGATTACCTGTATTTGTGTTTTTAGAACATTTTAGTTTTGTCTGGGTCTTTTGATGCTGGTGTTCTTGAGGGGAAGTTCCCACTCGAACATGTTGCTGCTAAGGTGACTTCGTTTCCGTTCTGGCCCCAATAGATGGCAATTCCTCATGCATGAGCCTTCAAGGCTACTGCAAGCATCATAACTAAGCAAAGTAGCAATGGTGGTGTCAGCTGCTTTTTGGCCATGGTGAGCTACGTGTGCTTCTATGCTAAATGCTAGCCTTAAAATCACAGGATGTCTCTGCCTAAGAAGTTTTACAAAGGATGTCTCTGCTCGAGAATGGAGGCTGAGGATCCATCTTATAGTGAGAGGTATGACCCACCGTGATCGTGATCACTTGTGCTGATGCTTGTGGAGTCAGGTGTAGTGTGCGTGGCTTTTGGGTCACTTGATCTCTGTTTTGTTGTTTGTTGCTGTTAGTGTTCCATGACTAGATTTCTTCTGCTCTGTTTGAACGCAAACGTTTTAATATAATAAGAAGAAAATATC

>ONT.2246.2 LG01 30193841-30194650

GCTTTGAAATCTTCAAAATTTTCGGAAATCTGCAGATCTGAAGCTCGAATCGGGAAAAGGTCCAAGGAGAATGCTGCTGTCAGTTGGGCTGTCAATGGCTTGCCGCTGGTTGTCTGCATCGAGTTGGTGTCCCACACCCATTGGAGTTCCAGCCTCTTCTCCTGCCTTGGCCACAATGATTAGTTCTGTAGCAGCCATCTTGAAGTCTGTAAGTTCTTGTAACTTGGAAATTGATTTTTTTTTTTTTGGGTTTGTATAAATCTAGAGATTCTTAGTTTTGTTTCATAGTGGGTTTGATGGGGAGTGAGATATGGTCACTTTTGTGTTGAGAGTGTGTTGGGGTTACGAGAATTCGTAATTATGAAATGGCTTATTTCTGTATTTATATTTGTCTAAGAGGTTTTCTTTTTTCTGGGGTTTGTGGGAAACTTGAGTTTCTTTTTCTTTCATATTGTGTTTTATGGAGAGGATTACCGTAACTCATTTA

>ONT.249.5 Contig00712 5942-15672

TAAAAAATAACTTCCCCACCTTCTCTCTCTTACATGCACGCAGAACCTCGAAATCTCTGTAGCAATGGCGGCATGCGCGGCCGGCGGCTCGTCAAGCCGCGTCTCTATCTCGGCTCTCATCTCCCAAATCGGCTGCTCAAGCCTCAAATCTAGTGCAAAGACGCCGTCTTGCGGGAGGTGGAGATCATTATGGATCCCCCAAGGTTCACTGCTGGCAGGACCCATTAACTCCATCTAGATGGAAGGAAGAGCATTTTGTGATTGTTTCTTTATCTGGTTGGGGGTTACTTTTCTATGGAGGCTACAAATTCTTCTCTGGTGGCAAGAAAGACAAAATTTCATAAAATCACAGGAGCACTCCACTAGGCTTGATGGTGCAGTGTGACAAGTTTCGTTGTTGAGTCACATTTGAAACTAAATTATTTTCCATAACTTTGCAATATGTAATGAACGTATTTTGGTTATGAGATGTCGATTTTGAGTGACTAGTTTACATAACCCCTCGTTTATGGTTGAAAAATGCTGAGACCATGTTGCATGTCCCGGTCATGTGGGCAGCCATTGTTGATCCTTTCTTATTTCTG

>ONT.13740.2 LG09 64923516-64932569

ATGAAATGTTTTATGTTGGTGTTTCTTCTCTTCATCTTCTCTTTTCTCCCTTTGCGCGGCCCGCCCGACCAGCCCGGCCGTTATTTGGGATCTCGTATTATCGTGTTCTTCTCAACCCGACCTCTCTCTCTCTCTCTCTCTTAAAAAAAAAAAAGGCTTTTCGGACTTTAGAATTAGGGGGATTTCTTTTCTTTTTTTTAGGGTTTCCTTCATATCATAATTGAGAGTGAGGGTTTCTCTCGGTTCCAACTCCTCCTCCTCCTCATCCTTTACTATTTTTGCTTTCAGTTTGAATACAACAAGTCAAAGATTATTAAAAAAAAAAAAAAGGATTTCCAGCCTCTCTTACTCTACACAACAAGGGGGGATTTCTATTCTCAGTTTTCTCTTTTTTTTTTTTTCCTGAAGATCTCCTCGGAAAAATATTTATTTTATTATGACAATGCATTTTGCATGGCCTTTAGTGGATGCCCTTGCTTCAGAACTCAGTTTCATCTGTGGTGAAATCCATTAGTGCAGTGCAGTGCTCAGTCGTAATTGTCAGAGCTCGTAGAAAAGGTTCAAGGGTTTACAAGTTGGAAGGATTCGGTACAAGTAGTAGATTTTGGTGATTCCAGTAACATGCTCACAACTTGATTTCATGATGAGGAAGCTATGATATGCTTCTCTGGTTTATCACCCTTGTAAAGGTGCTTGCTGCATGAGAGAACCAAAATCGCATAACAAGCCATTCATATTCTCATCGACAGTACGCTTAAAGCTGATGGAAATATTGGGCCTATAACATTCAGTGAGACAACTGACAAGTAAGAATATCTGCACAAATGTTAGGGGTCCAATTTGGTGATCATCTGCGCATGTTTTTAGTAGCAATCTCATGTTTGGGATTCTTGCTTGCCTTTTCAACTTTGTACCCTGTTTGGCGTAGATATGCTTGTTTGTAGCCAATATCTGATGCTTAGATGGCTCGACATTATGCTTTACTTGTATATCCAGGGTCTTATTCTGCATAGAAGGGAAGTACATGTAAATGAATAAAGTATATCTAGTTTTTATTTGGTGCTTAATTGATGTTGATGTTTGAGACCTGAGTTGTAATTTTATCCTGTTATTCCTGTGGAAGTTCATATTAAAATTTTTTTCCTTCCAATC

>ONT.6833.1 LG04 69089943-69091190

ACCCCAAATCGTTTGAAAACCCCTTTCTCTCCAACCCTTCTCTAGTTCTCCCTCTCCAGTCTCCACATCGAAGTGATCGCACCCAGGTCTATCCAGCAATGGCGACGATGGCGATTGGCAACCGTCATGCAGAAAATCGAAGACATCGAAGAAGAGGGTGGGTGGTAGAATATCTCTGTCATTGGTTGAGTCTTGGACTAACATCAGCTGTTGGACAAAGAGTCTTGGACGACATCTGTTAGGCAAGCGCTGCCCATATGAGTCTCCCTTTCTTGTCCAGAAAAGTCCACAGGCACGACCCATGTGATCTTCTCGACCACATGCAATATTGTCTACAAGCTTCATCCAAAAGAAGGTACTCTGTAAAGACTGCCTTGTGCACTTACATGTCTTAATAAATTGATCAATCTTTTATTGTAGTGGAATCTTTTGAGGATCCATAGAGTTTTAATAAGTCCCTCAAGAGGATTTTAGGACTCTTA

>ONT.7296.1 LG04 8915580-8933743

GAGACTCTGTCACGTGTGCGTCACAGCATATCAGATCTACGAGATTTCAACGAACACAAGCCCTAGGGCCTTGTCCACCTGGGTTTCTCAATCCAACATTTACCCCCTTCCTCCCTCTCTACCTCCTCAACAAATTCATTCCAGCTTCGATCAAGCACACACCTTACCTACCTGACTACCTCTGTCTGTCTGAGTCTATCCTCTCCTCTCTCGTTGATGCCCTTCTTTTCAATATGCTATGTCTGAGTCTCTCCTCTCCTCTCTCGAGTGGCCGCAAGAAGGAAGGCAAAAGTATTTTGAAATGTGGTGGTATTGATCCCATTGCCAAGGATTGTATCATGGACACCAAAAACAAACAGCAACTCCATAAATGTCTTGAAAGATGAAAATGCATACGGTGGTGGAGAGGCTAGCGAACAATGCAGTGTAGAGACTCACCAAAGGCTCTCTCTCTCTCTCCGCGATCAACTTTACTGGAACTCCTTTCTTTCCTGGAACTCCTTTCTTTCAAGAGACTATTAAATATATGCTATGATGCCCAGTTATGAAAATGGACCTCAGGCCACATGTGTTGGGCCCAAGCATATATAGGAATTTTTTTATCTATTTAATTGTTGTTAGTTACTATTGATGATTTGTATCTAGGATTAGGTTTCCAATATTATTTAGTTTCCCAGTTTAATTAGGTTTCATAGGGTTTAAGTTTTCTTTTCCTATACATATGGTATCGTAGAAGATTATTTTTAGAGTTTATTAATTGAATTGAGTTTTCCCATTAA

>ONT.12805.5 LG08 3431863-3446805

GAGAGGCAGAAGCACAGAGGTGTTGGTCTTCGTGCACCTACCACTCAAAATAATGCGAGGTGCCTGGGAAACTATCAACGGAGCTTGAGCTCTACTACAGTCAGGCGCTTTTTACTTTCACAAGTAATTGGGCTTTTTTCTGCCCAAACAGGGTGAATTGTGGCATTTGAAGTTCAATAAAGTATTAAAGGTGTGGATTGCTGGAGTTTGATAAAGAGTTATAGGGATATTGGCAACTTCTTAATGGTGAAGTTTAGGCTGTGTCATTTCAAGTTTAATAAAGCATTACAGGTGTGAATAGTTGGAGTCAGATAAAGAATAGTAGGGATCTTTGCACCTTCTTATTGGTCTGGGCAATTCATGGAGTTTAAAAACAAACAAAACATTCCTTCATGATGTTGTTTGAACTCATGGTTGTGTTTTTTGAGTCGCTTTATTTGGCTTTGGTTTAACTATGAATTTACTGGTGTAGTTGTACTGTGTGGATCTTGCTTGTCATCTTTGGTTTGTTCTTATTCACTTGTTTGGAACTAAAATGATTCTCTATTTTTACCTTCTGTATGTGAGGAAATTATATAGACCATGGATGTAATCCTAATGACTTGATTATCT

>ONT.9743.2 LG05 92704097-92713211

GGGAATCGAAGAACCAGTTCCTTTCTCGTCTCTCCAGCTCCCCGTAGGAACGATGCTTGAAGGTAGAAGTGATTTTGGACTTGCCGAGCTACTGAATGGAGGATGATCTGACACCTTTGATAACAATTAACGACTACTTCATGTGAGACTTAAACTCTCTTTACTGCCAGCGACAATATATATAATTGTGGTTCTGTAAGGGAGGTCTTATGCAATGCCAAAGCGCTGGCTCACATCTAGCTAATGTTCTCTGTTACTTTATCTTGCCCATGGTCTTATTTTCTTTTGCATTGACAGATGGAATGATGGATATATATATCAATTATTTGGTTAAGATACACCAGACAGAGAGCACTGTTTGAATGGGTTGAGCCTTCAACTTGGGGTTGTATTTTGTTTGGTCAATCTGTTCTTCCCCTTGGGACCGAAGTGATGATTGGGTTTGTCTACTTTATCTGTACTAAAATGTATTCTAAATTTGAGTACCGTGTTTTTAGCAAGATTGATGTATATGTAAGTTGATTTCTAATTTATTTTTATATATGTGGAAGAGTAGTGCTGATGACTTATCATCCGC

>ONT.17239.4 LG12 44341213-44342899

GAGGAATGGAAGAAGATGGTCCGTCTGGTAGTGCTGGGGCTGGTCCCAAGATTGAAGAGGTTGACTAAGGAAATCAGATGAGCCAATGCCGCCTGCTTTTTTTTTAACCTTATGGATGATGATGATGATAACAATGATGACAAAGGAGATAACCACTCAATCTTGGGCTTTGGCTTATGGATGATGATGATGATGACAATGATGACAAAGGAGATAACCACTCAAGCTTGGGCTTTGGCTTGATGTATATTTTGGCTCAGCCAACCAATTCGGGTATTATCCAAGGTCAGTCATTGATCTAGGGAAATCTTTGGTTTGTTATAAACCCAGATTGCCCTGTTCCAGTTACACATCTTTGCATTTTGGGAAAGTTCCTTGATTTTGATTCTGAAGGATGCAAATACCGTGAAAAAAGTCCTTGACTTGAAAGGAGCATAAGAGAGGGAGAGCTTGAGTTTTTCTTTCTCAAGAGGAATAGAGACAATCTCAAATGTAAATCATCATGTATGGTGCTTTTCAGCGCAATCATATATTTTCACATCTAATAATACCCATCATTTTCAGAGCTTCGTCCAA

>ONT.10072.1 LG06 12134391-12136134

CCAGAAGTCTCCTCTCTGAGCCCTACCCTCATCCCCGAGAGAGAAAAGCTCCCGCTCCCACGTTCGCCCACCGGCCTTTCTACCTCTCTCCTGCGATCGTCCATTCGGAAAGGCCCCAACATCGAGCGAGGTTTGAAGCGATGCACGCCCAAATCATCGATCCCTCTCCTACGATCATCTGTTAGGAAAGCACGCCCAAATCGACGATCTGTCTCATGCGATCATCTATCCAGAAAGCTCTGCAGCGCGTGCCAGGACAGAGTTGTACCTTGGACTATTGTGTACTCTCTTGAATGTACTAGTAATTTGTTTAGGGCTGTCGTATTTTCATTTGGATGGATAAAGTCAGAGCTCAATGTGTAATGATGTCAAATGTTTGTGAAACCCAAATTGCAGTGAGAATGGAAGAAATCAAGACTCAATTGAAGAGCTGTGCCTAAGAGTAAGACGTCTGAGTGAGTGAAAGCACAGAAACTCAAATTAAGGGAAGTAGATGCAAAATTGGCTGTACATATTTTATTGCAATTCTTCTCTTTGCATCGTACATGTGATGTGGACAAAATGGACGTGCCTATTTCTTTGAGTTGATGTAGACATTGTTCTCATGTGGGAGGTACAAGAGCGTTTTGTTTTATTTTGCTTGTTGGGTAAGGATGCTTTGTTTAAGAAAGAACAAAATTATTTGTTTGGATCATTTGGAACATGACTGATCAGTTTGATTTTGTGATTTGTCTTTAGCTTGTTTGTAGTTAAGAAGTTCTAGTGTAGTTTTAATGAAAAAAAAAATAAAAATAAAATGTTGGCATGGT

>ONT.11297.1 LG07 22121381-22133265

ACAGAAGTTGCCCTTGCCCCACTTTTCAAGGCCTGAAGCACCTTTGTATTCTACCTTCAGTCCTTCACTTTCGGGTCGTTACGCACGTCTGCGCTTGGGGATTGCTATGGAGGGCCAAGGAAAATTCACCCAAGAGCTCCTCCTTATCTTCTGTCTCTGCCATGGCTTCCCTTATCGACATGGACAAGGGGTTGGAGAGAGGTCCATGAAGCATTCTTTACCGAGTATTTGGTTGAACTGCCAATGGAACTGAGTTTCTTTTGTCAGTTGCAGTTTTTAGTTAAGATGGTCCAGTTTCTTTACAATAGGGTCATTTCACAGATTGCTTGTGCTTATCCTGCACATATTTGGAACTTATATGCATTGCTGGAAGGCATTTGAAAGAAAAATATAATTGGCATATACTCTAGAATCTACCTACTCTAATTATTATGTTTTGTTCATTTCTTTC

>ONT.4598.2 LG02 36092784-36094615

AAAAGACTCGCCCAAATCAATTGGAAGCCCCAAATCGGACGATCTCTTCCTCCTCCTTCCGACGCATCTCTTAATCCTCATCTAAGCAGGATTCCGCCGAGAGAGTCATCTTCCTCCTCCTCCCCTCTCGTCGACGTCTCTCCCTCGTCTCCATCACTTCCGTCGTGGAGCCATCCCATATCCTAGGGCTCGATCTCCCTCCGCACCAAAGCAGAACATCGATAGGGGGACGATCTCATCCTCGATGAGCTCTGCATCCCCTTTTGCCTGTCATCTCTGTCGCAGCACTCCCGGCCTAAACCAGGATGAAAGTGGCTATGGTACATTTCTTTGGAAAGATGAAATAGTGAATTGGCAGCTTGGTGAAGCTAATTCAACGATGTGTGAAAGAGAAAACTAGAGAGACTAGAAAAGAGAAATTTGATAGCGGTCATTGACAAGTTAGAAAGTTCAGTGTGTGCTTTCATTGAAGTAGAGAAAATTCGAAATGCGTATCTGCGAAAAATTTATCATGCTATTTTAGTTGCATGCTGTATAATAATTTTAGTTTTCTTGTTCAACCATACATGAAATACATGCTATGGGGATATGTTTTCTCTAAAGGTTTAAAAACTTGTCTTCTGTATGTTGGAACATAGAAGTTATGAATGGGCATATGTTCTCTA

>ONT.14215.8 LG10 488227-490522

GAAGTTCCTCATCCTTCACTCCTCCATTCTCTTTCTCAAATCTTCAGAACTAGGACTTGCACGTGTGTTGAGAGTGTAGATTGGGACTTCCTTATCATCTTCCCTGACTTTCAAAACAATCGTGGAGCAAGAGTCTTCTTCATCAACTATCTATTTCATATAAAAGGGTGCAGCTCTCGGTCATGAGGAGGCCCAGGAGAGAGAGAGGAGGAGACCGAGGAGGTTCCTCCCGGATTAGAAGTGTGGGCAGCTAAAGGCCCTATCTAGTCCTTTTTCCCGGAGAGCGACGTCTGTACTATTTTGGGATATTTTGTAGTGGGTGTAGGTGATGTCCATAGATAGTGATCACCCTCTTTGGTGGGACTCACTTGTTAGATATTGGAGGCACTTTTGAATATACTTTTGTTTATCTATGTAGGGTGTACTTTGTAATCCCTACCTATGTAGTTAATTGTAAATATCTTTTGCGTCAATATGGAATGTTAAATGGACTGATCATGAAATGATACCAACTCTGGGAATATTTGAG

>ONT.14215.5 LG10 488223-492871

AGGCGAAGTTCCTCATCCTTCACTCCTCCATTCTCTTTCTCAAATCTTCAGAACTAGGACTTGCACGTGTGTTGAGAGTGTAGATTGGGACTTCCTTATCATCTTCCCTGACTTTCAAAACAATCGTGGAGCAAGAGTCTTCTTCATCAACTATCTATTTCATATAAAAGGGTGAGAGCTCTGGCTACAATTTAGATTTTTGGGTTTAATTGGGTCAAAGGATTTCCCTACACTACATGTATGTCATCTTATTTCCATCCTTGCATGAATTCTTGTTAGGGTCTGGATTTTTGGGGGTTTTCTCTTACATTTGAGCCAAGTTAGTGAAAGAGGGTTGTAAAGCTCTATTGAGGCAAGTTGTTGACAGACACGTTGTGATTGGTGCCTATACACGGGAGTCCAATGGCCACTGTTGGGGTTGCAAAAGACTGGCTCAAGTATTTGAATAGATCTGTATTTGAAGGGCGAGCTATGTTGAGAAGGTTATCTGTGTGTCGAGAAGCTCTTCATTGAGCTTCAAAAGCCTTATCACAACCACCTAAACAAGAAAATCACTCTTTCAAGCAAATATCAGACTATTTGACCAAAGGTCGAGGAGGCAAAGAGGACGGACACCAACACCTGGCTGGTACTGTGGCCTGATAACGATCCCTGCTATGGGATCACACACCTATAACCCTAGTAGAACTTTGGGAAGTTGCTTTTTGGCAGCCACTCCGGAGTCCATCAAATTACTCTCCTTGTGGCAGGATACATTATTATTATCCCTGCTTCTATGAAAGGGATCAATCCTACTCTCTGTAATACAGCAAGTGCAGTGTTTCTCCAAGAGTGAGAAGAAGCTTGAGGAGGAGCTTGTCTTGCAGCATTTCTCTGAGGCCAAGGGAGCAATAGCTTTTCTCACAATCTTTCTTCCAGTTTCTAGAGCTATTCTCAGAATTCAGTGTATTTCTCCCTGACCGAGGAGGTGTTATCACAGAGCATTTCTCATAGACCGAGGAGGAGCCCTTGGAGCACGGTTTCTGCTAGGCTGGAGGAGTCATTCCAGCAGCTGCAGTCATAGTGTGAGCTTGGGTTCTCGACCTCTATGGCTCTATCACTTGTATTCACCTTAAGGTTCAGCCATCTAGCAGTGGATATTCGAGGTTTCCTTTGCCTTGTGGTGGATTAATGATCATTGAATGCCAACTTTGGTCAC

>ONT.11718.1 LG07 3579774-3581871

ATTCCATTCGTTGCAGGTTGATGGCTGGGAGAGTATGAGTAGAGAAAATTTTCTCTCTTCTTCCTTTTCAACTTCTCCCTCTTTCATCCATTACTTTAACTTTATACAAGTGAAAATCTTCTTCTTCATCATTTCCTTCATCTTCTCCACCCTTGCTTCAAGAGACCATTTGAGCTCCAACTCTTAATTTTGGGTTCTTTGTCAAGGGTTTGAAACAATTGGGGATTTTAGGATTTGAAACCACCAAAAGTGTAAAAGAGATTGAAAAGCTCAAAACTTAAGGCAAACACAGATACAACCCCCGGTCCTGAAGAGGTCCAGGAGGGAGAGAAGAGCACCCAGGAGATATATTCAGGACTAGAAGAGTGGCCAAAACCAAGGCCCGTCTAAAATTCATCTTCTCGGAAGTGCGAGCTAACCTTGTAGATATCATCGTTCGTATGATTATTCTCTTTTCATATAGTCATACGGAATTCTTTTGGAGATACTTTTGGTAGAGATTGTATAGGGTTCTTTCGTTTACACATCTCTTGTAACTTAATATATATGTTTGTTTACTTTTGTATATCAAACTTTAAATGTTTAATTCGTACTTGATGGAATTTGATACCTATATCAATGAATTATGACTTCCGCTAT

>ONT.9544.1 LG05 41841159-41844693

GGTTCAAACCTCTTCTCCTTGTCCTGTTTGCTGAGAGGGAAAGAAGAGGGTGGCACTGCTCCTCTTTGATGTTTGAGGTCAATGGCGGTGGCAGTGTCCGACATCGACGGTGATGGCTGCAATGGGGCTTGGCGAGGCTGGAGGGTGTTACGAATCTTACGATGGCGTTGATCAGAGAAGACAATGCTGCTGTTGCGGGGTTTTGGTGCACGATGCATATTGTAGAGATGGATTTCAGAGTTGAAGATGAATAGAATTGTGGGTTTTATCTCTTGAGGTGCGTCCTCATGTACGAAAGTCTTTGTAGAGACTATTATTGAGCTTTGGCACATTTCCACACAAGCTCCAACACTAGAGCCTACATCAGTATGAAGTAACGGTCACATTTGGTCACCAAATTCTGAGGAAAAGGTTTCTACTTTCCATAAAGTCGTGGAAAACCCATGTTTGATAATTGTAAATCCCTTTGAGGAAAAGATTTGGAAATAGATTTGTGAATTTAAACCAACTTTTCT

>ONT.1161.1 LG01 25312840-25326897

CTCTCACAATGGGATTTTGGGTTTTGGTTGGTTCCTGTTTATCTGGGTTCTTCTTTGGTCTTTTTTGGCCTTTTGAGTGTATCTAAAGGAGGAATGCTTATCTAGGTTTGGTTGGCACCATCTATATGTGGGAGGGAAGAGAGCCTAGTGTTGGGAACTTCCCCCTGGTACTTGCATCTCAGCATTTCAAGGGAAAAAAGCTCACTGTTAATGTTTATTTCTCATCATCACTTGCGCATGGTGTGGAACCATCGTACTAATGTCATCTCTCAAACACCCCCTATCTGGCGAGACACCTCATCCCCAACTTCCCTCTCAAACAACCCTATCTGTGGAGATGACCAAAACGGACACTGGCACTTGGATTTAATATCTTGTTCTTGAGATTGATGGACGCTTTCTGATCATATGCACACTTTAGTGTTGTTTTGTTGTTTTTAGTTTATTTGTATATGTATATTGGACATTTTTATTCGACTGTATTTTTATTAATTTAGACATTTTTATTCGACTGTATGTTTATTAATTATACTGTTTATTTAAGGACATGAATGATTTGATGAAGAATGGGGATGATTTTGTTTGGATG

>ONT.1451.1 LG01 105615216-105623048

ACAAAAAACATCCCTCCCAACCCTCCCCTTCCGCCAAAAATGGAAATCCCTCACCTCCCAATCCCTCCTCTCTTCCAACACCACCATCTACGCTAGCTCCCTCAAATAGACGATTCCTCCTCCATTAAGGTCGCAAATGGCTGTACCCTAACCCTTCTCATCTCTCACAGGATACTTCCATCTCTTCCTTCGTCTTCATCTCTCTAGCATCTCCAAAGCAATGAGCCCATCTTCTCTCTTCTCCGTCTTCGTTTTTCTGAACCTCCTCCGTCTTTGTCTCTCCGGCATCTCCAAAGCAGCGAGCCCATTCCCTGTCTTCTTCCTTTTCATTTTTCTGGAACTCCCCTGTTTCTCTCTCCGGCATCTCCAAGATTGATGGACGCTTTGGGTCAATTGCGGACTAGCTCAATGTGTTTTGTATATGTATATTGGATTGTATATTTGTTTAATGAGATGAATGATTTGTTTATGAAAAATGGAATATATTTGTTTAATGAGATGAATGATTTGTTTATGAAAAACGGACTTTGTTT

>ONT.14772.1 LG10 26912049-26922447

GTGGACTCAGAGATGGATATTAGAACTGTCACATTTGCAGAAAACTGAAGCCATAAGAAGTTGTGCATCCCTCAGACGAAGAAGGGGGAAAAGTGAAGGATCTTGTAGATAGACATGTGGTCTGGTTGCTTGGTTTGTTAAGAGGAGGGAGCAAAACAGCGAAGAGAAAATGACACAATGATGTTGCGAACATTTATTGGCGTGGCTGTAAGAAGATAAAAGATGCGAGGAAGAGGAGATGAAGAATATCTGAAGGCACAACTTTCACTTGCAAACACAGGAGCGAGCTTACCCAGTGGAGACTGAAAGAGAACAGAAAGAAGAAGATGGGAGCCAATGTTGCAATTCCAGTCCTGTTAGGGATGAAGTATAGCCATTTTGTTTGTTTTTTTTTATTACCTGAAGAATGTTTTATTTGGATCTTGAACTGGAGGGGGTGTGGCCAGTAGATTGGGAGTTTTCTC

>ONT.11136.2 LG07 4901188-4910502

AATTCCCTCCCATTTCCTCACTTCACTCTTCTTTTCCTTTCCACACTTTTCCACCTCAACTATCCTCAGCACCTCACCCCAGAGGCTCAAAACTTTTGGAAGATAATGGTTGAAACCCGTCATTTCTTGGCACATTGCCAGAACGCTGGACTCCCCTTCTTTTGAGATGGTATGTGCTAACTGGTTTTTATTGCCATAGTGGCAACAGTAATGTAGATATTAACAGTTATTAGATGAATAAAAAGTAGTGCAGATATTGGATGAATAATTAGGATTCTAGTTGGGAAACTGATAGTGTCTTCTGCGTCACAAAATCATGGATACTTATGCTTTGGAGGCTGAACTCAATACTGATGATAGAAAGACTGAAGCTTTGTGCTTGATTTACCAGCCAGCGACTCATTGGAGGGGGTCTCATTATAACTGCATTTCAGTCGGGAAACTATGTACTGTGTGATGTAGTGTATCTATGGAAACAAATTCAATGTGCATTTCATAACCACTGATACGTATCCTCTGTTGAAGAGAAGGCAATATTGTAGCTGAATTTTTTTTCAAGTGTATCCACCAATGTTTATATTAATTCTCATCAAATACTACCAGTACCAGGGCATTTTCACTAATTTGCAGGAATGACATCGACAAGCAGAGGATGAAGCTTCTTTACTATCTTTCCAGCTGCTGACTCTTTCAGGGATAGGAGGGATAAGCTTTATGTTGCTTGAAGGGTTACATTGTTTTTGTCAGTGTTGTTAGTATTGTTGTTGTCTGTATATTGTTGTTATATTCATCTCTGTTTCCTTGTTATTTTGGACCTTATAGTTAATAATAGGATCTGCTTT

>ONT.15088.2 LG10 79253518-79254421

ATCCACGCCTCTTTTCTTCTTCCCCAAAACCCTAATATCTGAGATCTCTCTTTCCCATAACCCTAATCTATACATTGGTTGATTTGTTGGGAGCTTCCAGCAGGTTTGAGGTTTTAGACCTAATTGACATCGGAAAAGAAGAAGCAGAAGCCAAAACCCTACTTCGTTGCATTTTTCGGTCGATCTTCGATCTTTGAGATCTCTCTTCCCCAAAACCCTTATCTTTCTTTGGAGCTTCCAGCAGTTGAGACCTACTTCCTGCATATTTTCGGTTCTTCGACTATCTACAGAATCTCCCATTCCTTCTCTTCTCCAGCTCGCTGCATCTGGACCTTTCGCATTCTCTGAAACCCTAGCTTTTGCAGAGCCATGATTTGTGCAATATCAGGCGAGCGCCGGAAGAGCCCGTGGTTTGTAAGAAATCAGGGCTTCTGTTCGAGAGGCGACTGATCGAGCGACATGTAGCAGTAAGAACCTCTGACTTTTAGCTGCTGATAGGGTTTTCTTTTGCTATCTCGTTGGTTTTTGTAAGGGAAGCTTGGAAGAAGTTCAGCTTCAAGTGTAATGGCTATGGTGGAGTTATTGTGTCCCTATCTGTGTATTTGAGCTGTTTTGTTGTTCTCATCCGCTCTTGGTTACTCTGTTTTGTTTGTAAATACTAATGGCCTGTTTGGTTGGATTCGATTGAAATGGGAATCCTGGATTTCGTGTAAAACAGTTGGTTTTTTTTTTGTTTGAACGCTACTGTCTCGTGAATGGAATGATCAGATGCTTTCATTTTGAT

>ONT.12345.1 LG08 10352825-10353792

CTCTTTCCTTCATCCATCGGTTTCTACCTCACTTTCACCAGCGAGCATCGGAGCACCGTCGACCACCGACAACTACTTTTCAGACTTTGCAGAATTGGTGGGGTCTGCTTAAAGGAAGTCATGATTCTTCTATTTCCAGTCATTTTCCAACCCTGTTGGGTTTAAACTTGAAATTACTCTGAAGATGGATCCCGGTATATGCAGAGTTGACAAGGTTTGCTAAATTGAAGTCATTCTTCTTGTTTGGTTGAAGTTTCAAGTTACTTCAAATTCAGAATATATTTCTCTCCAGCAACCCCCTTGACCGTGAATATTTGTATCTGGCTCACTGGCTGTCCAAAAGATGTATATATCAGCTGATTTATCGGCTGGGTTGATGTGATTTATTTTTGAATTGAAGATAGATTTAAGCAATATTGTTAATATTGGCAGATTTTTTAGAAGATGTTGTCAGGAATGTATTTGGGAGAGATTGTACATAGAGTTTTACTTAGAATAGCTTAAGAAGTGCTATTTTT

>ONT.16553.1 LG11 19308184-19337757

TTGGAGTGGTTGACGGGTGGCAGGAGGACGAGACTCACAACCCCGATGTTCCGGCAGTTTCACATAGGAGAGGGTTAACACCTTTTGTATTTGTTCCATTTGAAGAGGTGGAAACATCGGTGCTGAATCTTCCAGTTGACAAGATGGATTACTTTGTTCCGGGTTGATGAGCCAGTAGTACAACCTAACACCTTCTAGTTATGTGAGTGTTTGTTTCATCTCTTATCTGCTTCGTTATATCCAGTGCCGAGGTTTCAGTAACTCATGATTCAACCATTTCTCTGGACTAGTCGCTGCTCATGTATGCTGTATACTGTCTGCAGCACTGTTTGATTTGGATTCCTGTATATAAAAAGTAAAACTTTTCTTTTCTGTAGTATTGTGGTAAAACTGTCTTGCTAATCTTGTTCCCCTTTGATCTCTGTTTGTTAATAAATTTTCACTCTTTAATAGTGATCTCATGCGTATGTA

>ONT.14891.1 LG10 36987007-36991190

AAGAGAAAAACTTCTTTTTTTTTCTTTCTTTTTTTCCTTTTCCTCTCCTTCCATGGCTTGTTGAAGAACAACTTCTTGGAGACTCTCCTTGCAAGATCCAGCTGGGCCAATCAACTCCTCATCATATTCTGAGCCACATAGTTTGTTTTGGTGTGAATCTAGCAGTGACAACCCCCTCTTCCATCACCTTCTCCTTTCTTTGATCGTCTAAGAGAAGGTAGCAGAGAAGGGCAAGAGGGAACTCTTCTGAAGAGCAACAGAGGCCAATCAAGCTTGTGGGAAGGAAGAAGAAGCGGGAACGCATAGTGGATGCAGCGATTGAGTTCTCACCTCGACCGTGCTACGATTTACTACACTAGGAGGTATGCACCGTTTGGGGGAGCATGGCAACGGAGATAATCAGCCATTGGACTTCAATATGCGTGGCTGATAAGAGAGCGTCTGAATGAAACCGAACTAGATATCAGTTTATTTCTTTAAATAAATGTTGAAGTTCTAGGATTTTGATTGAAAGACTTATTTATGTTTCATGTCTAGTTAAATTGATACCTCAAATAAATGTTTTCGTTGCTC

>ONT.14994.2 LG10 63324168-63330041

CTTTTTCCTTCTTTCAACTCTCGGTCGTCTCAGGACGCTCCACTCAGCGTCACACTCGGAGAAGAAAAGAAAAGAAGACGGAAGAAAAAGCACGCTGCTTCCGATCAGATCTCCAACTCCAAAGCATTCCTCCCATACAACAACAACAACAAGAAGAAGGGTAAGGTAGCAGCGGAAAGGGAGGATTTTGGATAATCTACATAGTGGAATCTAATCTCTATGGCTATGTGATCCCTGCTTACTAGTGGCACTTCCAGTTTGAATTGGATTGAGGGACTCAAGCGAGGAGGACAAGAGGCCCAAATCGATGCATTGCTCTCACTAATCAGCGAGAGCGCATCGTGGTAGAATGTAATGAATATGGGCAGCCCATTGGAAGGAATGAGAGACTTTTGTCTTCATATCTTGGCGTCATTGCAAGAGATGGGCAAAAAGCTCCCCTCAATTATTCTGATTGGAGGAGAATGCCTGCAGAGCAGACAACAGATATGTGGCAGGCTATACAGATTTCCGGTGCTGCTACTAATGATATCCATCATGATGGTCCTGTTTAATGAACCGTTAGCCCATGTACACTAGATCCAACATTTCCCATTAATCAATAAGAAGCAGCAATAAGAAGCAATGGCTGGGCAGCAGATAGGAGTAGCAGAGAGCCTCTCATTGGATCTCTCTTTCTAGTTACTTCATCAATTCATCTTATGGAAAAAAAAGGCATCGGGTCGATTTCAGCCATTTCATCATCCTCCATCCTTCCTACCAGTGCTCTACATACAACCTCTCTTTCTCACCATCAATCTCTCTCCAACAGGCATGGAAATGCACAGTGCCTACTATGAGGTTCTCTTCTCAGATATTAATGCTTATGAGGTGGATTTGGGAACAGATGCAAAGCACTGCTTTTGTCGCAAGGGTGATCTGCTAGCTGTGGTCGACTAGACGTGGAGCAGAATGAGGGGGAGATTATCAACCTGTGCTTTCTAATAACAGGGACTTTTTTTTTGCAATTGTCTTGGAAGTGTACAATTTGAATCCTTCATGGGTTCCCACTTCCCAAACGGTTATTTTGTTGGTTCCATCTTGATGATGCATCACTTTCCATGCATTCTCACAGTATCAGCCTTGTTTGGTGATTAATGTACTTTTCATGCTTTCTGGATCGTTCTGGGCTGCTACTTTAGATGGTATGGGGACTACTTGTAACAGTCAGCCGACTAGCAACGGTTCTGATGAGCTTACCTACCAGTAATATTTGTAGTTATGAGGCTCATCCTTTATTAGGCTTTTCACAATAAAATTTTTGTGGGAGAACTGATGTAAACTTTGAACTTGATTATGAATCACTGCTTTTCCCTTT

>ONT.4890.4 LG02 108476513-108483777

GGAGAACCTGGTCACCACTTCTCCTCTTCCTTTTTGGCTCTCTTTGTGAAATGCCCAACGAAAAGGAAAACATCATCGCTTCCTCTTATTACTCTTCCAATCTCTCTCAAGAATTAGAATTTGGCCATGGCTTCGAAGAGGATCGTCAAAGTGCAGCTCAAGGGTCTGCAAAAAGATCCTCCAACTTCCAGGGGCGGGAAGGGTGAAGAAGATAAAAATAGCTGGAAGTAATTGGTGGGTGCTTTTCTTTCCTTCCTATTGAACATATATTTCCATTTGGAAATCTTTGGCAATGACCATGTGACCTTTTGGAAGTTATATGGACAATCTTGTATGGCAGTTAGTTGTTGGAAATGGATACTTAATTGATTAAAAATTGTGATGGTCTTGGTCCTATTTTTGGTAGATGTTACAAAAAAGAACAATGGTTGTTC

>ONT.10140.1 LG06 34082795-34140599

AGAACTGAGGAAGGGGCATTCTTCAAACGCATTCTTCAAACGCGTCCACTGTGTCATCTTCTTCGTCCTTCCACAGCGGATTTGAGGATGGGGTAGATGATTGGTGGTGTTAGTGCGAGCAATGGCCGCTACATTCTAGATTTGAGAAGGACAGAGTCAAAAGTCTTGTATACTACAAGTCTACAAATATTGATGACAATAAATTGCTGTTGTGGCATCATCATTAGAGTCAAAGTGTTACAATAAAGTACTCCATTGAGTTCTGTTCCACCAACGGTATTTGGATTCAGTCTTTGTCCATGTTTCAAAACATCAACAAGACAAATTGGATCTTAAGGCAATCAAGTGCATAGTTGTATGATATCCCAGTTCCCAGAAAGGCTACAAATGCTATCTTCTCGAGAACAGTGGTCATCTTTATGTTATAATGGATGTAACTTTTCATGAAGATATTCCTTTCTACTCATCAAGTAAGGAATTGATTCATGAAGATAATGAAGGGGAACACCTACCTCTCCTTGAATTGGTATGCCTACCAATGTTTCAAGGACTAACAAATGAGGCAAGTCAGAAATCATAGAATCTTGAGACAAATAAACTAACTGACGTGCCAGGGGAGCCATTGCAAGTGTGCTACAGGAGAAGAAAAGAAGCAAGACCATTGTTCTCATCTGCACCATCATCAACCAAAGCTCTGAATTTAGATCCACCGATGATCCAGATCCTCGACCGCAATCAGAAGTTGTCACACAATCAGGCATGCGTAGATTTTTTCTAATCCCGCTCTTCATCATGTGATTGCCTTGCGCAGTTGGAGCAGAAAGATTTGTTTGAATGATCAATTTATTTCCCTGCTTTTTAGCCTTGACCAGTTGATATTTGTTTTGGTTAGGGAAGAGTAGGTGCATGTATTATGATACAGTGGTTACATTGAGGATATGCTTGGCTGTTCCCAAATTGAAGGTGGATTGATTTTTCTTGTCCTTTTGGGGTAGTTCAAACTTTTAAATCTAGCGTGTGATAATCTGTCAATACTTTTCTTATACTATCAATGTTCAGATGCCGACAACTATTTGATGTAGATATTTATGTTATTTGCTTGTCGCCCGAAATGGATTGGAAAGCATATACAATAATCAAATCAGTTATAGTT

>ONT.10068.3 LG06 11842890-11850129

ATCAAAAGACAAAACGAGAAGAAGGGCGCAAACAGACGACAAGGCCAAGGTCGAGAAGAAGATGGGAACAAGAAACAAAGGTTGGAGACAAGGATATTCAGTTCGTTTGGTGTAGACGAACCCAGGTCTCTCTCTGCTCACTGTTCTCTCAAACTGCTCTCTCTACTTTTCTCTGCGAAAATCTCAAGATTGAATGATAATTGAAAAACAAATTTTGTTGAAAGAGTCGATACAGCTAGGATGCAGTCAACATGAAGGCCAAGCATGCAAAGTAATAGATTGCTATATGTTAAAATGATATGCATGAAGTTTTTTAAAACAATGGTGCAAGTGTTGGAATAGTGCAACAATGAAATGATCATATATGAATATGTAAATTTGCATTGGCATTAAAATGTTAGTACATAGTAAAATGTTAGCATTTGTTTTAATTTTGTAATGGTAATGATAAAGTGCTTTCAATGTTGAAAGGATGTTTGAATATTAATATGAAGTGGAA

>ONT.9743.1 LG05 92701590-92713211
[truncated: 280,406 more chars]
