# Supplementary material for: Full-length transcriptome sequencing reveals the molecular mechanism of monoterpene and sesquiterpene biosynthesis in Cinnamomum burmannii
Source: Front Genet. 2023 Jan 6;13:1087495. doi: 10.3389/fgene.2022.1087495 (PMC9852720; doi:10.3389/fgene.2022.1087495)
Supplement: Supplementary file 4 [file Table2.DOCX]

Table S2. Information of primers and qRT-PCR systems

| ID | Sequence(5’- 3’) | Product Length(bp) |
| --- | --- | --- |
| Actin.F | GACTCTGGTGTTCCTCGCC | 139 |
| Actin.R | CCATCTTTCCTCCTGTTATTTC |  |
| Cbur03G002680.F | AAAGAGTTTGTCAAGGTGGTGG | 106 |
| Cbur03G002680.R | GGATGTCATTGCGGCTGC |  |
| Cbur0G021000.F | AGGGCGCAATGTCATACC | 148 |
| Cbur0G021000.R | CCCAAGACTTCCAACCGA |  |
| Cbur03G002300.F | ACCTGTCCTTCTGGTCCATG | 167 |
| Cbur03G002300.R | GTGCGTCACCTCTGTCTTTC |  |
| Cbur09G005010.F | TCCACTCAGGGTATTTGCC | 233 |
| Cbur09G005010.R | CCTCTCTCTTGCTCAGACTTGT |  |
| Cbur09G005020.F | GAGAGAAATGATGCCCTGC | 163 |
| Cbur09G005020.R | TCCAAAAGTAACACTCGACGA |  |
| Cbur09G005210.F | GTGACTGCTACTGCTTCTCC | 190 |
| Cbur09G005210.R | AATCTCCCTCAGCAAACCAT |  |
| Cbur10G002890.F | CTGGCACATCTGCTTCCC | 188 |
| Cbur10G002890.R | CCTCTGTATACCCTGGCTTCTT |  |
| Cbur12G014500.F | GACTTATCCGAAGATGTTGGG | 137 |
| Cbur12G014500.R | AAGCAATGAAATCTGTCAAAGC |  |
| Cbur07G020680.F | CAGCAGCGACTTCTACTCTC | 102 |
| Cbur07G020680.R | CAAGGTGATAGCTTGATGCC |  |

| Bestar® SybrGreen qPCRmasterMix | 5 μl |
| --- | --- |
| PCR Forward Primer（10μM） | 0.25μl |
| PCR Reverse Primer（10μM） | 0.25μl |
| cDNA | 2μl |
| ddH_2_O | 2.5μl |
| Total | 10 μl |

qRT-PCR systems
