# Supplementary material for: Full-length transcriptome sequencing reveals the molecular mechanism of monoterpene and sesquiterpene biosynthesis in Cinnamomum burmannii
Source: Front Genet. 2023 Jan 6;13:1087495. doi: 10.3389/fgene.2022.1087495 (PMC9852720; doi:10.3389/fgene.2022.1087495)
Supplement: Supplementary file 5 [file Table5.DOCX]

**Table S5** Mapped clean reads against the reference genome of *C. burmannii*

| Sample | Total Reads | Mapped reads | Mapped rates % |
| --- | --- | --- | --- |
| FA01 | 3,316,638 | 3,284,474 | 99.03 |
| FA02 | 2,617,126 | 2,593,905 | 99.11 |
| FA03 | 2,973,141 | 2,947,056 | 99.12 |
| FB01 | 2,956,111 | 2,932,678 | 99.21 |
| FB02 | 2,957,802 | 2,931,647 | 99.12 |
| FB03 | 2,737,808 | 2,702,886 | 98.72 |
| FC01 | 2,623,869 | 2,603,891 | 99.24 |
| FC02 | 2,951,221 | 2,928,954 | 99.25 |
| FC03 | 2,706,518 | 2,686,137 | 99.25 |
| FA01 | 2,080,083 | 2,066,207 | 99.33 |
| FA02 | 2,167,850 | 2,153,705 | 99.35 |
| FA03 | 1,935,584 | 1,920,760 | 99.23 |
