# Supplementary material for: Full-length transcriptome sequencing reveals the molecular mechanism of monoterpene and sesquiterpene biosynthesis in Cinnamomum burmannii
Source: Front Genet. 2023 Jan 6;13:1087495. doi: 10.3389/fgene.2022.1087495 (PMC9852720; doi:10.3389/fgene.2022.1087495)
Supplement: Supplementary file 6 [file Table3.DOCX]

**Table S3** Statistics of clean reads generated by Nanopore sequencing

| **Sample name** | **Number of raw reads** | **Number of base pairs (bp)** | **N50** | **Mean length**  **(bp)** | **Maximum**  **length (bp)** |
| --- | --- | --- | --- | --- | --- |
| CBS11 | 3,941,015 | 4,395,094,219 | 1,150 | 1,115 | 11,541 |
| CBS12 | 3,158,401 | 3,878,772,599 | 1,329 | 1,228 | 11,272 |
| CBS13 | 3,598,604 | 3,931,212,335 | 1,136 | 1,092 | 11,200 |
| CBS21 | 3,498,621 | 3,833,936,087 | 1,144 | 1,095 | 20,253 |
| CBS22 | 3,617,045 | 3,960,187,226 | 1,137 | 1,094 | 18,254 |
| CBS23 | 3,336,851 | 3,846,278,207 | 1,216 | 1,152 | 66,250 |
| CBS31 | 3,199,713 | 3,700,748,717 | 1,240 | 1,156 | 10,750 |
| CBS32 | 3,481,448 | 3,873,857,207 | 1,165 | 1,112 | 11,201 |
| CBS33 | 3,302,780 | 3,847,621,135 | 1,224 | 1,164 | 13,787 |
| CBS41 | 2,468,910 | 2,440,139,466 | 1,092 | 988 | 19,832 |
| CBS42 | 2,623,618 | 2,662,338,404 | 1,126 | 1,014 | 9,999 |
| CBS43 | 2,341,602 | 2,660,599,873 | 1,216 | 1,136 | 18,659 |
