# Supplementary material for: Full-length transcriptome sequencing reveals the molecular mechanism of monoterpene and sesquiterpene biosynthesis in Cinnamomum burmannii
Source: Front Genet. 2023 Jan 6;13:1087495. doi: 10.3389/fgene.2022.1087495 (PMC9852720; doi:10.3389/fgene.2022.1087495)
Supplement: Supplementary file 8 [file Table4.DOCX]

**Table S4** Numbers and percentages of full-length reads

| **Sample name** | **Number of clean reads (except rRNA)** | **Number of full-length reads** | **Full-length percentage (FL%)** |
| --- | --- | --- | --- |
| CBS11 | 3,725,516 | 3,316,638 | 89.02% |
| CBS12 | 2,984,934 | 2,617,126 | 87.68% |
| CBS13 | 3,408,336 | 2,973,141 | 87.23% |
| CBS21 | 3,314,325 | 2,956,111 | 89.19% |
| CBS22 | 3,400,107 | 2,957,802 | 86.99% |
| CBS23 | 3,149,801 | 2,737,808 | 86.92% |
| CBS31 | 3,007,629 | 2,623,869 | 87.24% |
| CBS32 | 3,305,170 | 2,951,221 | 89.29% |
| CBS33 | 3,116,684 | 2,706,518 | 86.84% |
| CBS41 | 2,346,526 | 2,080,083 | 88.65% |
| CBS42 | 2,467,315 | 2,167,850 | 87.86% |
| CBS43 | 2,218,026 | 1,935,584 | 87.27% |
