# Supplementary material for: Full-length transcriptome sequencing reveals the molecular mechanism of monoterpene and sesquiterpene biosynthesis in Cinnamomum burmannii
Source: Front Genet. 2023 Jan 6;13:1087495. doi: 10.3389/fgene.2022.1087495 (PMC9852720; doi:10.3389/fgene.2022.1087495)
Supplement: Supplementary file 9 [file Table6.DOCX]

**Table S6 Statistics of consensus reads**

| **Sample name** | **Number of consensus sequences** | **Number of base pairs (bp)** | **N50** | **Mean length**  **(bp)** | **Maximum length (bp)** |
| --- | --- | --- | --- | --- | --- |
| CBS11 | 34616 | 42608146 | 1449 | 1230 | 5700 |
| CBS12 | 34687 | 45937499 | 1576 | 1324 | 7362 |
| CBS13 | 37176 | 41812726 | 1314 | 1124 | 4962 |
| CBS21 | 34974 | 39210518 | 1319 | 1121 | 5494 |
| CBS22 | 36765 | 41841470 | 1341 | 1138 | 5678 |
| CBS23 | 33136 | 39898177 | 1442 | 1204 | 6926 |
| CBS31 | 29437 | 37100380 | 1497 | 1260 | 6828 |
| CBS32 | 30445 | 35730403 | 1398 | 1173 | 5708 |
| CBS33 | 30429 | 37526026 | 1468 | 1233 | 5742 |
| CBS41 | 20428 | 22094467 | 1257 | 1081 | 5173 |
| CBS42 | 20205 | 23281876 | 1350 | 1152 | 5431 |
| CBS43 | 23587 | 27734281 | 1390 | 1175 | 5609 |
