# Supplementary material for: Full-length transcriptome sequencing reveals the molecular mechanism of monoterpene and sesquiterpene biosynthesis in Cinnamomum burmannii
Source: Front Genet. 2023 Jan 6;13:1087495. doi: 10.3389/fgene.2022.1087495 (PMC9852720; doi:10.3389/fgene.2022.1087495)
Supplement: Supplementary file 10 [file Table10.DOCX]

Table S10 Sequences of mono- and sesqui-TPS genes detected in the present study

>Cbur12G001610.1 gene=Cbur12G001610

ATGCCTCTTGTTTTGGGCTCTGGCCATTCTGATGTCCCAAACTCAAACCAATCACAGGGAAATGGAAAATCCAATGTAGA

AAGGAAATCTGCAAATTATCACCCTGACATCTGGGGTGATCGTTTCATTAAAGCCTCCATTGATGACTTGAAACCTGATG

AATTAACTCAAGAACGGGCTAATGAGTTGAAGGAAGAAGTGAATAGAATGTTTATCAATGTCAATGATCATTTGCAAGAA

CTGAATTTGATCGATGCCATCCAACGCCTCGGAGTGGCGTATCACTTTGAAACCGAGATTGCTGAGGCCCTGCTCAGGAT

ATACAACACCTATGATAAGGAGGATGATAGTGATGATTTGCACACTGTCGCTCTTCGTTTTCGACTGCTAAGGCAAGAGG

GGTACAATGCCTCACCCAACGTGTTCAACAAGTTTATAGATGGAGAAAGCAAGTTCAAGAAACCGTTGGCTACTGACATT

CGCGGGATTCTTAGTTTATATGAAGCAGCATATATGGGAATACCTGGAGAAGATATATTGGATGAAGCCATTGCTTTTAC

TAAAGAACATCTTAACTTGGCACTTCCTCATCTTGAATCACCTCTCTCAACCCTAGTTACGCTTGCCCTAGAGTTACCTT

TGCGCAAACATATTGAGAGGCTACAGTCAAGGTATTACATCTCAATCTATCAACAAGAGAAGGCAAAGAACAACATCTTA

TTAGAGTTTGCAAAGTTGGATTTCAATATATTGCAGTTATTGCATCGAAAGGAGCTAAAGGAAATTTCAATGTGGTGGAA

GAAGTGGGATTTTGGTGTGAAGCTACCATTCATTAGAGAAAGAGTGGTGGAGTGCTACTTTTGGGTAATGGCAGTATATT

TTGAACCACAATATTCCCAAGCTAGAATCATAACAACTAAAATTTTATTGTTAGCATCAGTGATGGATGACACCTATGAT

GTATATGCTACAACAGATGAATTGGAACCATACACAGATGCAATCCAAAGGTGGGATCGAAGCGCCATAGATCAGCTGCC

TGATTACATGAAGTTACATTTTTGTGCACTCTTAGACACTGTCTATGAATTTGAAGAGGAACTTTCTCATGAGGGGAAAG

CCTATCGTATTTCATACTTGAAAGAAGTTTATAAGGAACTCTCCAAAAACTATTATATTGAACAACAGTGGACACATTCG

GGATATGTGCCAACATTAGAAGAGTATATGAAAGTAGCTTTAATCACTGGTGCATACCATATGCTGACACTAGTTTCATA

TGTTGGCATGAGAGATGTTGCAACTAAAGAAGCCTTTGAATGGGTGAAAAATATGCCAAAACTGGTCCAAGCTGCTTCTA

TAGTTTGTCGATTCAAGGATGATATCCAATCAAACCAGGAGAATGGAAGCACATATGAAGAGGCTTGCGAAAAGTTTAAA

AGTATGGCTGCAAATGCATGGAAAGACATCAATAAGGAATGTCTGAATCCTACAGTGGTTCCTATGCCTCTCCTCATGCG

GACTGTAAATCTCGCACGTGTTATTGAAGTCCTATACCAACACAGAGATGGCTACACTAATTCCACACATGAGACCAAAG

ATCAAATCTCTTTAGTGTTAGTGGAACCCATTCCTATTTAA

>Cbur09G005020.1 gene=Cbur09G005020

ATGGCTCTTGTTCCTAGTTCTGTCCACTCAGATGTTCCCACGACTAACCCAGTTCGAAAATCTGCCAATTATCACCCTAA

CATTTGGGGCGACCTCTTTTTGCAATCCTCTCCACATGACTATATGCTTGATGAAGCGATCAAAAGAAGGGCTAAAGGAC

TGAAGGACCAAGTAAGGAGGATGCTCCAAAATGCTAGTGATCCATTGGTAGAAATGAATTTGATTGACTCTATCCAACGG

CTTGGAGTGGCCTATCACTTTGAAACCGAGATCGAAGAAAAGTTAGAAAGGTGGTACAACGCATGGGTTGATGGGCTAAA

TGATGGCGAAGATTTGCATGCTGTTGCTCTCCAATTTCGACTCCTGAGACAACATGGTTACAACGTCTCACCTGATGTAT

TTTGCAAGTTTAAGGAAAACGCGGGGAAATTCAAAGAAAATTTAGCTCATGACATTCGAAGTCTCTTAAGTTTGTATGAA

GCATCCTCTCTGGGGACATATGAAGAAGACATCTTGGATGAAGCCAATAAATTTGCTAGAGATCATCTTCAGTTAGCAGT

TCATCATCTGAGCTCACCTCTTTCCACTCTAGTTAAGCTCGCTCTAGAGTTGCCTCTGCAGAAACGAGTTCAAAGGCTAC

AAGCAAGGTGCTATATTTCAATTTATGAAGAGGAGAGAAATGATGCCCTGCTGGAATTTGCAAAGTTAGATTTCAACATG

CTTCAGTCATTGCACAAAAGAGAACTAGGAAACATCTCACTGTGGTGGAAGAAGATTGATTTCACCAGAAAGCTTCCTTT

CATAAGAGATAGACTCGTCGAGTGTTACTTTTGGATCTTAGAAGTTTATTATCAACCACAGTATTCTCGAGGAAGAATGA

TGGCGACAAAGATAATATACCTGACATCAGTGATGGATGACATTTATGATGTGTATGCTACCCCAGAGGAACTTGAGCCT

TTCACTGATGCAATCCAAAGGTGGGATCGTGGGGCAGCAGATCAATTACCAGACTACATGAAAGTGCATTTTCTTGAAGT

AATAGATTGTGTTGATGAATTTGAGAAGGAATTAGCAGAGGAAGGACAATCATATCGTATATACTACTTAAAAGAAGCTT

TCAAAGAAGTCTCGAAAGCCTACAACAAAGAAGCCCAGTGGTTCCACTCAGGGTATTTGCCATCATATTCTGAGTACATG

AGACTTGCCTTGGTCACTAGTGGCTACCCTCTGGTGTCAGTAGTTTCAATGGTTGGCATGGGAGAAATAGTGACGAAGGA

AGCCTTGGAATGGGCCATGAGCACACCTCAGCTCATCAAGGCCTGTTCCGCCATTGCCCGAGTCAAGGATGACATCCAAT

CAAACAAGTCTGAGCAAGAGAGAGGACATGTTGCATCAAGTGTTCAGCTATACATGAAGGAGAATGAATGTACATATGAT

GAGGCATGTGTGAAGTTACAAGGGACGGTTGAAAGAGCATGGAAAGACATAAACAAAGAATGCCTGAAGCCTACACCAGT

TCCTATGCCTTTCCTCGTACGCGTCGTGAACCTCGCACGTGTGATTGAAGTCTTATACCAGAACAGAGATGGTTATACTG

ATTCAACACATGAGACCAAAGAAAGGATCTTATCCGTGCTTGTTAATCCTATCCCAATTTGA

>Cbur09G007320.1 gene=Cbur09G007320

ATGCATGCCAAAATCTGCAATCAATCTACTTGCCTCAACCATCCTTCATACCTCAATCACGGCCGTCCATCCCCAGCATC

CTTACATCATCGTCGTCACACAATCACACTCATCCGATGCAGTTCCTACCAACCTCCTCCTAAAACCCTAGTTTCTTCAA

ACCCTCAACCCGTTCTCGAATCCCTGAAAGCCTTTGCCCCTGCCACCGTCGCCAATCTCGGCCCTGGCTTCGACTTCCTC

GGCTGCGCCGTCGACGGCCTCGGCGACACCGTCACCGTCCGAGTCGATCCGACCGTCAGTCCGGGCACCGTTTCCATCTC

CTCGATCGACGGCATCGGATCCGCTTCCAAGAAGCTCAGCTGCAACCCGCTCTGGAACTGCGCCGGGATCGCCGCCATCG

CCGTGATGCGGATGATCGGCGTCCGCTCCGTCGGCATATCGCTGTCGTTGGACAAAGGCCTGCCGCTCGGGAGTGGCCTC

GGCTCCAGCGCCGCTAGCGCCGCCGCCGCCGCCGCCGGAGTCAATGCGCTGTTCGGCATGCCGCTCTCGGAGAGGGACCT

CGTGCTCGCCGGACTGGAGTCGGAGGCGAAGGTCAGCGGCCACCATGCGGACAACATTGCTCCTGCGATCATGGGCGGGT

TCGTGCTTATTCGCAGCTACCACCCGCTCGATGTAATGCCGCTGAGATTTCCGGCTGATCGGGACCTTTTCTTCGTCCTC

GTGAGCCCCGAATTTGAGGCTCCCACGAAGAAAATGCGCGCGGCGCTGCCCAAGCAGGTGGGGATGAAGGACCATGTTGC

CAATTGCAGCCAAGCAGGCACGCTGGTTGCTGCTGTGCTCCAGGGGGATGCGAGGATGCTTGGGGCTGCGCTGTCGTCGG

ATGGGATTGTTGAGCCCGTCCGGATGCCATTGATTCCGGGGATGGCTGCGGTGAAGAAGGCGGCATTGGAAGTAGGGGCT

TTTGGCTGTACAATAAGTGGTGCTGGCCCTACTGCAGTGGCGGTGACGGATGATGAATTGAAGGGAAGGGAGATTGGGGA

GAGGATGGTTGAGGCATTTTGGGAGGAGGGGAAGTTGAAAGCGCTGGCGTCCGTCCGGAGTCTTGACCGGGTTGGGGCCA

GAGTGATTGGTGGCTGTTCCAGTTGA

>Cbur09G005050.1 gene=Cbur09G005050

ATGCCTCTTGTTTCTAGTTCTGGCCACTCAGAGGTTCCAAATGCATACTCCTCTCAGAGCAGTGGAAAGACTAACCCAGT

TCGAAAATCTGCCAATTATCACCCTAACATTTGGGGCGACCTCTTTCTGCAATCCTCTCCCGATGACTATATGCTTGATG

AAGCGATCAAAATAAGGGCTGAAGGACTGAAGGACCAAGTAAGGAGGATGCTCCAAAATGCTAGTGATCCATTGGTAGAA

ATGAATTTGATTGATTCTATCCAACGGCTTGGAGTGGCCTATCACTTTGAAACCGAGATCGAAGAAAAGTTGGAAAGGTG

GAACAATGAATGGGTTGATGGGCTAAATGATGGCGAAGATTTGCATGCTGTTGCTCTCCAATTTCGACTTCTGAGACAAC

ATGGTTACAACGTCTCACCTGATGTATTTTGCAAGTTTAAGGAAAACACAGGGAAATTCAAAGAAAATTTAGCTCATGAC

ATTCGAAGTCTCTTAAGTTTGTATGAAGCATCCTCTCTGGGGACATATGAAGATGACATCTTGGATGAAGCCAATAAATT

TGCTAGAGATCATCTTCAGTTAGCAGTTCATCATCTGAGCTCACCTCTTTCCACTCTAGTTAAGCTCGCTCTAGAGTTGC

CTCTGCAAAAACGAGTTCAAAGGCTACAAGCAAGGTGCTATATTTCAATTTATGAAGAGGAGAGAAATGATGCCCTGCTG

GAATTTGCAAAGTTAGATTTCAACATGCTTCAGTCATTGCACAAAAGAGAACTAGGAAACATCTCACTGTGGTGGAAGAA

GATTGATTTCACTAGAAAGCTTCCTTTCATAAGAGATAGACTCGTCGAGTGTTACTTTTGGATCTTAGAAGTTTATTATC

AACCACAGTATTCTCGAGGAAGAATGATGGCGACAAAGATAATATACCTGACATCAGTGATGGATGACATTTATGATGTG

TATGCTACCCCAGAGGAACTTGAGCCTTTCACTGATGCAATCCAAAGGTGGGATCGTGGGGCAGCAGATCAATTACCAGA

CTACATGAAAGTGCATTTTCTTGAAGTAATAGATTGTGTTGGTGAATTTGAGAAGGAATTAGCAGTGGAAGGACAATCAT

ATCGTATATACTACTTAAAAGAAGCTTTCAAAGAAGTCTCGCAAGCCTACAACAAAGAAGCCCAGTGGTTCCACTCAGGA

TATTTGCCATCATATTCTGAGTACATGAGACTTGCCTTGGTCACTAGTGGCTACCCTCTGGTGTCAGTAGTTTCAATGGT

TGGCATGGGAGAAATAGTGACGAAGGAAGCCTTGGAATGGGCCATGAGCACACCTCAGCTCATCAAGGCCTGTTCCGCCA

TTGCCAGAGTCAAGGATGACATCCAATCAAACAAGTCTGAGCAAGAGAGAGGACATGTTGCATCAAGTGTTCAGCTATAC

ATGAAGGAGAATGAATGTACATATGATGAGGCATGTGTGAAGTTACAAGGGAAGGTTGAAAGAGCTTGGAAAGACATAAA

CAAAGAATGCCTGAAGCCTACACCAGTTCCTATGCCTTTCCTCGTACGCGTCGTGAACCTCGCACGTGTGATTGAAGTCT

TATATCAGAACAGAGATGGTTATACTGATTCAACACATGAGACCAAAGAAAGGATCTTATCCGTGCTTGTTAATCCTATC

CCAATTTGA

>Cbur0G006370.1 gene=Cbur0G006370

ATGTCTATTGTTTTGAGCTCCAGCCTTTCAGATGCTCCAAACAAACACCATCCGAAAGGAAATAAAACTTCTGAGGTAGT

TCGTAAATCAGTGAACTATATTCCGGAGATATGGGGTGATCGCTTTGTTGCATCCTCTCCGGATAACTTGAAACCTGATG

CACAAACCCAACAAAGGGCTAATGAGTTGAAGGAAGAAGTGAGGAAAATGCTGAGGAATGTAGAGGATCATCTGCAAGAA

CTGAATCTGATCGATGCTGTCCAACGTCTAGGAGTGGCCTACCACTTCGAAGAAGAGATTGCACAAGCTCTACTCCGGAT

GTACAAATCAGGGAGGGATTACGGTGATGATTTGCATGCAGTTGCTCTCCAATTTCGACTCTTAAGGCAAGAAGGTTACA

ATGTGTCACCCGATGTATTCATGAAGTTTAAAGATGAAGAAGGTAAATTCAAGAGAACTCTAGCTGGTGACACAAGAAGT

TTGCTTAGTTTATATGAAGCAGCACATATGGGAACGCATGGAGAAAACATATTAGATGAAGCCATTGCTTTTACAAGAGA

GCATCTTGACTTGGCACTCCCTTGTCTGAACCCACCTTTTTCAACCCTAGTTGAGCTCTCACTAGAGCTACCTCTTCGTA

AGCGCCTAGAAAGGTTACAGACAAGGTATTACATCTCTATCTACCAAGAAGACGAGGACCGAAGCAATATCCTACTAGAG

TTTGCAAAGCGAGATTTCAATCTTTTGCAACTTTTGCATCGACAAGAGCTAAAAGAAGTCTCAATAGCTAATGAGTTGAA

GGAAGAAGTGAGGAAGATGCTGAGGAATGTGGAGGATCATCTGCAAGAACTGAATCTGATCGATGTTGTCCAATCTCTAC

TCCGGATGTACAAATCAGGGAGGGATTACGGTTTGCATGCAGTTGCTCTCCAATTTCGACTTTTAAAGCAAGAAAGTTAC

AATGTGTCACCCGATTTATTCATGAAGTTTAAAGATGAAGAAGGTAAATTCAAGATAACTCTAGCTGGTGACACAAGAAG

TTTGCTTAGTTTATATGAAGCAGCACATATGGGAACTCATGGAGAAAACATATTAGATGAAGCCATTGCTTTTACAAGAG

AGCATCTTAACTTGGCACTCCCTCGTCTGAACCCACCTTTTTCAACCCTGGTTGAGCTCTCACTAGAGCTACCTCTTCGT

AAGCGCGTAGAAAGGTTACAGACAAGGTATTACATCTCTATCTACCAAGAAGACGAGGACAGAAGCAATATCCTACTAGA

GTTTGCAAAGTGGTGGAAGAGTTGGGATTTTGCTGCAAAGTTGCCATTCATAAGAGATAGAATTGTCGAGTGCTACTTTT

GGATAGTAGGAGTGTATTTTGAACCACAATACTCTCGAGCCAGAAAGATGATGACTAAAATTATATCATTAACATCAATC

ATGGATGACATGTACGACGTGCATGGTACGCTAGAGGAACTTGAAACATACACAGATGCAATCCGAAGGTGGGATCGAAG

CATCATCGATCAGTTTCCTGACTACATGAAGCTACATTTTTCCGCTCTCTTAGACACTGTTGAAAATTTTGAAGAAGAAT

TGGCTCTAGAAGGGAAACCATATCTAAATGGTCCGACTGGGGTGCCCCGGACGGACCCCGGGTCGGGGTGCCCCGACGCG

CTGGGGGCCGTGGCGTCATTTGTTGGTATGGGGGATGTTGCAACCAAGGAAGCCTTTGACTGGGCAGTAAGCATGCCTAA

GCTCATTGAAGTTGCTGCTGCAAATGCCAGACTCAAGAATGACATCACATCAAACCAGCTTGAACAAGAGAGAGACCATG

TGGCGACTACTATCCAGATCTACATGAATGAGAATGGCAGCACATACGAAGAGGCATGTGAAAAGTTTAGCAGGATGGCC

GGGGATGCATGGAGAGATATAAACAAGGAATGCCTGAAGCCCCCTCCAGCTCCTATGCCTATCCTCATGCGAATTGTAAA

TCTCACACGTACGAGTGAACTGATTTACCAACATAGAGATGGCTACACCAATCCCACATATGAGACCAAAGAACGTGTCT

TGTCAGTCCTTGTTAATCCTATTCCATTTTAA

>Cbur09G004970.1 gene=Cbur09G004970

ATGTCTATTGTATTGACCTCCAGCCTTTCAGATGCTCCAAACAAACACCATCCGGTAGGAAATAGAACTTCTGAGGTAGT

TCGTAGATCAGTGAACTATGTTCCGGAGATATGGGGTGATCGCTTTGTTGCATCCTCTCCGGATAACTTGAAACCTGATG

CACAAACCCAACAAAGAGCTAATGAGTTGAAGGAAGAAGTGAGGAAAATGCTAAGGAATGTAGAGGATCATCTGCAAGAA

TTGAATCTGATCGATGCTGTCCAACGTTTAGGAGTGGCCTACCACTTCGAAGAAGAGATTGCACAAGCTCTACTCCGGAT

GTACAAATCAGGGAGGGATTACGGTGATGATTTGCATGCAGTTGCTCTCCAATTTCGACTTTTAAGGCAAGAAGGTTACA

ATGTGTCACCCGATGTATTCATGAAGTTTAAAGATGAAGAAGGTAAATTCAAGAGAACTCTAGCTGGTGACACAAGAAGT

TTGCTTAGTTTATACGAAGCAGCACATATGGGAACTCATGGAGAAAACATATTAGATGAAGCCATTGCTTTTACAAGAGA

GCATCTTAACTTGGCACTCCCTTGTCTTAACCCACCATTTTCAACCCTGGTTGAGCTCGCACTAGAGCTACCTCTTCGTA

AGCGCATGGAAAGGATACAAACAAGGTATTACATCTCTATCTACCAAGAAGACGAGGACCGAAGCAATATCCTACTAGAG

TTTGCAAAGCGAGATTTCAATCTTTTGCAACTTTTGCATCAACAAGAGCTAAGAGAAGTCTCAATGTGGTGGAAGAGTTG

GGATTTTGGTGCAAAGCTACCATTCATCAGAGATAGAATTGTTGAGTGCTACTTTTGGATATTAGGAGTGTATTTTGAAC

CACAATACTCTCGAGCCAGAAAGATGATGACTAAAATTGTATCATTAGGATCAATCATGGATGACTTCTACGACTTGCAT

GGTACGCTAGAGGAACTTGAACCATACACAGATGCAATCCAAAGGTGGGATCGAAGCATCATCGATCAGTTTCCTGACTA

CATGAAGCTACATTTTTCCGCTCTCTTAGACACTGTTGAAAAATTTGAAGAAGAATTGGCTCTAGAAGGGAAATCATATC

GTATACCCTACTTCAAACAAGCGTTCAAGGGACTCTCAAAAGCCTACTTGGTCGAAGTGCAATGGTCAAATTCAAGTCAT

GTCCCAACATTAGATGAGTACATGACCAATGCTTTAATGAGTAGTGAATACCCTATGCTTTCCGTGGCGTCATATGTTGG

TATGGGAGATGTTGCAACCAAGGAAGCCTTTGACTGGGCAGTAAGCATGCCTAAGCTCATTGAAGTTGCTGCTGCAAATG

GCAGACTCAAGAATGACATCACATCAAACCAGCTTGAACAAGAGAGAGACCATGTGGCGACTGCTATCCAGATCTACATG

AATGAGAATGACAGCACATATGAAGAGGCATGTGAAAAGGTTAGCAGGATGGCCGGGGATGCATGGAAAGATATAAACAA

GGAATGCCTGAAGCCCCCTCCAGCTCCTATGCCTATCCTCATGCGAATTGTAAATCTCACACGTGCGGGTGAAATGTTTT

ACCAACATAGAGATGGCTACACCAATCCCACATATGAGACCAAAGAACATGTCTTGTCAGTCATTGTTAATCCTATTCCA

GTTTAA

>Cbur03G002310.1 gene=Cbur03G002310

ATGGCTTTATCCACAGGATCCACATTCCCATGTTTGAATCGCCCATCCTTCTTCCTGCACATATTTCATTCCTCTCCTAA

GACTTTCCTATCGGCTACAAAAGTAGGCAGGCCCCTCAACTGCATTCCGAGCTCTGTAATCTATGAGACTGAAGTTTCAA

GAAGAAGAGCCAATTACAAGCCTAACATTTGGGACCATGATCTCCAACAGTCACTACGAAGTGATTACCAAGTAAGACTT

TGCAAATTCAATGTATCCCATTCCTCAATTAAGACTTCCCTATCACTTCCCAAAATCAACAGGCCCTTGAACTGCCTTCC

AAGCTCTGATGAAGCATATGCCAAACGAGCTGAGAAGTTAAAGGAAGACGTAAAGCACTGCATGCTCCAAGAAGCTGTGA

ACCCATTGGCTCAACTGGACCTGATCGATCCCATCCAACGTTTGGGGGTGGGACATATCTTTGATAAGGAAATCAAGGAA

GCCCTCAACATCATATGGGATAGTCACAATAAGAATGACAACAAAGGAGGATTTGGGGATAATTATGATCTCTATGCTAC

TTCTCTCCTCTTTAGGCTCCTCAGAGAACATGGGTATCATGTATCGCAAGATGTTTTCAATAAGTTCAAGGACGAGGGAG

GTGGTCTCATCACAAGTGTCAGTGAAGACATCAATGGAATCCTTAGCTTATATGAGGCTTCTCATCTTGCTCACCAAGGA

GAGACTCTCTTGCTTGAATGCAGAACAATCACAAGCACGTACCTCAAAGCTATCAAGGAAAGTGTCGATATTATACTTGC

AAACAAACTAGGGCATGCCCTGGAGCTTCCTATCCATTGGAGGACACAAAGATTAGAAGCTTTGTGGTATTTAAACATAT

ATGAGAATGAAGAGAACATGAACCCCACTTTGCTTGAACTGGCAAAGCTTGATTTCAATATGGTGCAGGCCACACACCAA

AGGGATCTTAGGAAAGCATTAAGTTGGTGGAGTAGTCTGGGTCTTGGAGGAAAGCTGAGCTTCTCCAGAGACAGGATGAC

GGAATGCTTCTTTCTGGCACTAGGAGTGATGTTTGAGCCACAATTTGGTTATCCAAGGGTAGAACTTGCAAAAGTCTGTC

AGTTGATCACAACCATAGATGATATTTATGACGTCTTTGGATCGTTGGATGAATTAGAGTGCTTCACTGATGCTGTTGAC

AGATGGGACATTAAATCAATAGATCGGCTGCCTGAGTACATGAAGATATGTTTCCTTGCTCTCTACAATACTACCAATCA

AATGGGATATGAGATCCTAAAAGATCATGGCATCAACATCATCCCATACCTACAAAAAGTGTGGGCAGATCTTTGCCAAG

CAATGCTAGTGGAGTCCAAGTGGTACTACAGTGGATACAGACCAAGCCTAGAAGAGTATCTGAACAACGGATGGGTATCA

TCATCAGGACCAGTCATTCTGGTACATGCACTTCTGCTTGCAAAGAAAACAATATCAACAGAAGTGTTGGATGGCTTGAA

CAAAAACCCAGATCTAATAAAATGGCCATCAATGATATTTCGACTTTGCAATGATTTGGTAACATACAAGGATGAGAGAG

TCAGAGGTGATGCACCATCTTCTATTGATTGTTATATGAAGGAAGCCAATGTTTCGGAAATGGATGCTCGCAATCATATG

GAAGACCTGATTTTTGATGCTTGGAAGAAGTTGAATGAAGAACTGAGCTCTACTTCTTCTACATACCCCATTCATTTCAT

CAACTCTGCCTTCAATCTTGCTAGGGTGGTCCACTGCATGTACCAGCATGGTGATGGCCATACTGTTCAAGATCGCAGCA

CCAAAGATAGACTCACATCATTGCTGGTTCAACCCATTCCACTGGTGGTTGCAATTGAATAA

>Cbur03G002160.1 gene=Cbur03G002160

ATGGATGCCATCCAATGTTTGGGGGTGGGCCATATCTTTGATGAGGAGATCAAGGAAGCCCTCAACACCATATGGGTCAG

TTGCAACAACAATGGCAACAAAGGAGGAATAGAGAATTATGATCTCTATAGCACCTCTCTCCTTTTTAGGCTCCTCAGAG

GACATGGGTATCTTGTATCACAAGATGTTTTCAATGAGTTCAAGGTCAAGACAGATAGTCACATTGTAAGTTCCGTTGAA

GACATGAAGGGAATCCTTAGCCTGTATGAGGCTTCTCACCTTGCTCACGAAGGAGAGACTATAATGGTTAAATGCAGAAA

TTCCACAAGCATGTACCTCAAAGCTTTTAAAGGAAGTGTAGACATTAAACTTGCAAACAAAATAGAACATGCCTTGGAAC

TTCCCATGCATTGGAGGGTGCCAAGAATAGAAGCTCTGTGGCACCTAAACATCTATGAGAGCGAAGAGCACTTGAACCCC

ACTTTGCTTGAACTGGCAAAGCTTGATTTCAATATGGCGCAGGCCGCACACCAACTTGATCTTAGGAAGGCATCAAGATG

GTGGAGGGATCTCGGTCTTGGAGGAAAGCTGAGCTTCTCCAGAGACAGGATGATGGAATGCTTCTTTGTGGCCCTGGGAG

TGATTTGTGAGCCACAATTTAGTTATCCAAGGGTAGAACTTGCAAAAGTCTGTCAGTTGATCACAACCATAGATGATATT

TATGATGTCTTTGGATCATTGGAAGAATTAGAGTGCTTCACTGATGCTGTTGACAGATGGGAAATTAAATCAATAGATCG

GCTGCCAGAGTATATGAAGATATGTTTCCTCGCTCTGTACAATACTACCAACGAAATGGGATATGAGGTCCTAAAAGATC

AGGGCATCAACATCATACCACACCTACACAAAGTGTGGGCAGATTTTTGTAAAGCAATGTTAGTGGAGTCCAAATGGTAC

TACAGTGGATACAAACCAATTCTAGAAGAATATCTGAACAATGCATGGGTATCATCATCAGGACCGATCATTCTGGTCCA

TGCACTTCTCCTTTCAAAGCAAACAATATCAACACAAGTGCTGGATAGTTTGGACAGAAGCCCAGATCTCATAAGATGGC

CATCAATGATTTTTCGACTTTGCAATGATTTGGGAACATATAAGGATGAGAAAGTCAGAGGTGATGCACCGTCTTCTATT

GATTGTTATATGAAAGAAGCCAATGTTTCGGAAATGGATGCTCACAATCATATTGAAGAACTGATTTTTTATGCTTGGAA

GAAGCTGAATAAAGAACTGAGCTCTACTTCTCCATACCCCATTCATTTCATCAACTCTGCCGTCAATCTTGCTAGGGTGG

TCCACTGCATTTACCAGCATGGTGATGGCCATACAGTTCAAGATTGCAGCACCAAAGATAGACTCACATCATTGCTGGTT

CAACCCATTCCACTCAATGTTTGGTAA

>Cbur03G002300.1 gene=Cbur03G002300

ATGGCTCTATCCACAGTATCCACATTCCCCTGTTTGAAACACCCATCCTTCTTCTTGAACGTATTTCATTCCTCTACCAA

GAATTTCCTATCGGCTACAAAAGTAGTCAGGCCTCCCAACTGCATTCCAAGCTCTGTAACCTATGAGCCTGAGGTTTCGA

GAAGAAGAGCCGATTACAAGCCTAATATTTGGGACCATGATTTCATACAGTCACTACGAAGTGATTACCAAGACGAAGCA

TATGCCAAACGAGCTGAGAAGCTGAAGGAAGAGATAAATTGCTTGCTCCAAGAAGCTGTGAACCCATTGGCTCAACTGGA

GATGATTGATGCCATCGAACGTTTGGGGTTGGGCCATATCTTTGATAAGAAGATCAAGGAAGTCCTCAACACCACGTGGG

TTAGTCACAACAACAATGAGAACAAAGGAGGAACAGAGAACAAAGATCTCTATGCCACTTCTCTCCTCTTTAGGCTCCTC

AGAAAACATGGGTATTATGTGTCACAAGATGTTTTCAATAAGTTCATTGACGAGAAAGGCGGTCTCATCGCAAGAGTCAG

TGAAGACATCAAGGGAATCCTCAGCTTATATGAGGCTTCTCACTTTGCTTACCAAGGAGAGACTCTTTTGAGTGAATGCA

GAACTTTCACATGGTTGTACCTCAAAGCTTTCAAAGGGAGTGGAGACATTGTACTTGCAAAGAAAGTAGAACATGCCCTG

GAACTTCCTATGCATTGGAGGATAGGAAGAATGGAAGCATTGTGGCATCTAAACATATATGAGAGTGAAGAGCACATGAA

CCCCACCTTGCTTGAACTGGCAAAGCTTGATTTCAATATGGTGCAGGCCACACACCAAAGAGATCTTAGGAAGGCTGATA

GATGGTGGAGAAGTATCGGACTCGGAGCAAAGCTGAGCTTCTCGAGAGACAGAATGATGGAATGCTTCTTTGTGGCTTTG

GGGATGATGTCTGAGCCACAATTTGGTTATCCGAGGGTAGAGCTTGCTAAAGTTTTTCAGTTGGTCACAACCATAGATGA

TATTTATGATGTCTTTGGATCGTTGGATGAATTAGAGTGCTTCACTGATGCTGTTGACAGATGGGACATTAAATCAATAG

ATGGGCTCCCTGAGTACATGAAGATATGTTTCCTTGCTCTGTACAATACTGCCAACGAAATGGGATATGAGATCCTAAAA

GAGCAGGGCATCAACATTATTCCATACCTACAAAAAGTGTGGGCAGATCTTTGTAAAGCAATGTTAGTGGAGTCCAAGTG

GTACTACAGTGGGTACAAACCAAGTCTAGAAGAGTATCTAAACAATGGATGGATAACATCATCAGGACCTGTCCTTCTGG

TCCATGCATTTCTCCTTTCAAAGCAACCAATATCAACACCAGTGTTGGATGGTTTGGACAAAAACCCAGGTCTTATAAGA

TGGCCGTCAATGATTTTTCGACTTTGCAATGATTTGGCAACATATCAGGCTGAGAAAGACAGAGGTGACGCACCATCTTC

TATCGATTGTTATATGAAGGAAGCCGATGTTTCCGAAATGGATGCTTGCAATCACGTTGAAGACCTAATTTTTGATAGTT

GGAAGAAGTTGAATGAAGAAGTGAGAACTGCATCTCCATACCCTCCCCATTTCATCAACTGTGCCTTGAATCTTGCAAGG

GTATTCCACTGCATTTACCAGCATGGTGATGGCCATACTGTTCAAGATCGTAACACCAAAGATCGACTCACATCGTTGCT

GGTTCGACCCATTCCACTGATTCTCAAATAA

>Cbur0G021000.1 gene=Cbur0G021000

ATGGCATTGCAAATGACTGTTCCATTTCCATCCTCCTTCCTCCCAAATCCACGACACAGACCCACAGCTCATGGTTTCAT

ACCCCAGAAACGTGTCTTAAAGCATATTTCATGCTCCACTACTACACCAAGCTACTCAACCACAGTACCTAGAAGATCAG

GGAACTACAGGCCCAGTATATGGGACTATGATTTTGTGCAGTCACTAGAAAGTGACTACAAGGTAGAAACACATGCGCCT

CGTGTGGAGAAGTTGAAGGAAGATGTAAAGTATTTGTTGAACGACGCAGACAGGTCTTTGGCTCAAATCGAACTAATTGA

CAAGCTCCATCGTCTAGGTGTGAGTGGTCTCTTTGAAAATGAGATTAAACAACTGCTATACACTATATCATCAGACAACA

CCAGCATAGAAACGAAGGAAGATCTTCATGCAGTATCAACTCGATTTAGACTTCTTAGACAACATGGGTACAAGGTCTCG

GCAGATGTTTTTAATGACTTCAAAGGCGAAAAAGGTGGTTTCAAGACAAGCCTTTCATCGGACATAAAGGGAATGTTGAG

CTTGTATGAAGCTTCACACCTTGCCTTTCAAGGGGAGACTATCTTGGATGAGGCAAGAGCTTTCACAAGCACGCATCTCA

TGGATATCCGGGAGAACATAGACCCAGTCCTTCATAAAAAAGTAGAGCATGCTTTGGACATTCCTTTGCATTGGAGAATA

GAAAAATTAGAGGCTAGGTGGTACATGGACATATATATGAGGGAAGAAGACATGAATTCTTCTTTACTTGAATTGGCAAA

GCTTCATTTTAACAATGTGCAAACAACATTCCAAAGAAGTTTAAGGAGTGTGTCAAGGTGGTGGAAAGATCTGGGTCTTG

GTGAGCAGTACTCTAGCTTTGCTAGAGACAGGTTGGTGGATAGTTTCTTTTGGGCCACTGCAGTGACATCCGAGCCACAG

TTTGGGCGTTGCCAAGAAGCTGTTACGAAAGTTATTCAATTCATATCAACAATTGATGATATCTATGACGTGTATGGTAC

GGTGGATGAGCTAGAACTTTTTACTAATACGGTTGATAGATGGGATCTTAAGGCAATGGAGCAACTTCCTGAATATATGA

AGACCTGTTTCTTAGCTTTATACAACAGTATCAATGAAATAAGTTATGAAATTTTGAAAGAGGAAGGGCGCAATGTCATA

CCATACCTTAGAAATGCGTGGACAGAAATGTGTAAAGCATACTTAGTAGAGGCCAAATGGTATAGCAGTGGATATACACC

AACGCTTGAGGAGTTTCTGCAAACCTCATGGATTTCGGTTGGAAGTCTTGGGATTCTAATGTATGTTTTTGCTTTACTTG

GGCAAAATCTAGCACCTGAGAGCACTGATTTCGTTGAAAAGATCTCAGATATCTTACCATTGGCAGGGATGATGGTTCGA

TTTCCAGATGATTTGGGAACTTCAACGGATGAACTAAAGAGAGGTGATGTTCCAAAATCTATTCAGTGTTACATGCATGA

AGCAGGTGTTACAGAGGATGTTGCTCGCGACCATATAATGGGTCTGTTTAGAGAGACATGGAAGAAAATCAATAAATCCC

TAGTGGAAAGCTCTCTTCCCCTGGCTTTTATCAATTGTGTTATGAATCTTGGGCGTGTGGCCTACTGCACTTACAAACAT

GGAGATGGATTTAGTGATGGATTTGGTGATCCTGGGAGTCAAGAGGAAAGGAGATTCATGTCTTTATTTGCTGAACCTCT

TCAAGTACGTACTGGATGA

>Cbur03G002680.1 gene=Cbur03G002680

ATGTCTTTATTTGTTGAACCCATTCAAGTTGATGAAGCCAAGGCCCATGGTTTCATACCCCAGGAACGTGTCTCAAAGCA

TATTTCATGCTCCACTACTACACCAACCTACTCAACCACAGTACCCAGAAGATCAGGAAACTACAAGCCCAGTATATGGG

ACTATGATTTTGTGCAGTCACTAGTAAGTGACTACAAGGTAGAGGCACATGGAACTCGTGTGGAGAAGTTGAAGGAAGTT

GTAAAGAATTTGTTGAAAGAAACAGATAGTTCTTTGGCCCAAATCGAACTGATTGACAGTCTCCATCGTCTAGGTGTGAG

GTGGCTCTTTGAAAATGAGATTAAGCAAGTGCTATACACTGTATCATCAGACAACACCAGCATAGAAATGAAGAAAGATC

TTCATGCAGTATCAACTCGATTTAGACTTCTTAGACAACATGGGTTCAAGGTCTCCACAGGGGAGACTATATTGGATGAG

GCAAGAGCTTTCACACACGCACATCTCATGGGTATCAAGGAGAACATAGACCCAATCATTCATAAAAAAGTAGAGCATGC

TTTGGATATGCCTTTGCATTGGAGGTTAGAAAAATTAGAGGCTAGGTGGTACATGGACATGTATATGAGGGAAGAAGGCA

TGAATTCTTCTTTGCTTGAATTGGCCATGCTTCATTTCAACATTGTGCAAGCAACATTCCAAACAAATTTAAAGAGTTTG

TCAAGGTGGTGGAAAGATTTGGGTCTTGGAGAGCAGTTGAGCTTTTCTAGAGACAGGTTGGTGGAATGTTTCTTTTGGGC

AGCCGCAATGACATCCGAGCCACAATTTGGACGTTGCCAGGAAGCTGTAGCGAAAGTTGTTCAACTCACAACAACAATTG

ACGATATCTATGACGTGTATGGTACGGTGGATGAGCTGGAACTTTTTACTAATGCGGTTGATAGATGGGATCTTGAGGCA

ATGGAGCAACTTCCTGAATATATGAAGACCTGTTTCTTAGCTTTATACAACAGTATTAATGAAATAGGTTATGGAATTTT

GAAAGAGCAAGGGCGTAATGTCATACCATACCTTAGAAATGCGTGGACAGAATTGTGTAAAGCATACTTAGTGGAGGCCA

AATGGTATAGTAGTGGATATACACCAATGCTTGAGGAGTTTCTGCAAACCTCATGGATTTCGGTTGGAAGTCTACCCATG

CAAACGTATGCTTTTGCTTTACTTGGGCAAAATCTAGCACCGGAGAGCTGTGATTTCGCTGAGCAGATCTCAGATATCTT

ACCATTGGCAGGAATGCTAATTCGATTTCCAGATGATTTGGGAACTTCATTGGATGAACTAAAGAGAGGTGATGTTCCAA

AATCCATTCAGTGTTACATGCATGAAGCAGGTGTTACAGAGGATGTTGCTCGTGACCACATAATGGGTCTATTTAGAGAG

ACATGGAAGAAACTCAATGAATACCTTGTGGAAAGTTCTATTCCCCATGCCTTCATCGATCAAGCTATGAATCTTGGGCG

TGTCTCCTATTGTACTTACAAACATGGAGATGGATTTAGTGATGGATTTGGAGATCCTGGCAGTCAAGAGAAAAAGATGT

ACATGTCTTTATTTGTTGAACCCATTCAAGTTGATGAAGCCAAGGGTATTTCATTTTGTGTCGATGGTGGATATGCCTGA

>Cbur10G002890.1 gene=Cbur10G002890

ATGCCACTGAGATGGGCAATGAGAAATCTGTGTTCCAAAACGAGATTTTACAGAGGAGTGACTGCAGATGGGGATCTGGG

AATCTTATCCACGACACATTCTTACATTTCTTCTTCTAGTAGAAAGCCTCCTTCAAAGGTTTTTGATGGCAGAGAAAGTC

ATTGTTATCATTCAAATGCTTTCCATGATGTCAGGTATCAAATTCATCAAGAGAGAAGCTCTGTGGTTGAGGAACCTTTG

GATCCATTTTCTCTTGTTGCTGATGAGCTTTTGATCCTAGCTAATAGATTGCAATCTATGGTGGTTGCTGAGGTACCTAA

GCTGGCATCAGCTGCTGAATATTTCTTCAAGATGGGTGTTGAGGGAAAGAGATTCCGTCCCACGGTGCTATTGTTGATGG

CATCAGCCTTGGATACGTCCATACCCAGTTCAACTACTGGCACTGTGTTGCATGGTATTCCAAAAGAAACACGTGCAAGG

CAGCAGTGTATTGCTGAAATCACTGAAATGATTCATGTTGCGAGTCTACTTCATGATGATGTCCTAGATGATGCGGACAC

AAGACGTGGGATTGGATCTTTGAATTTTGTAATGGGTAATAAGCTTGCTGTACTGGCAGGAGATTTTCTGCTTTCCCGAG

CTTGCGTGGCTCTTGCATCATTGAAAAACACAGAGGTTGTGTCATTACTGGCAACTGTCGTGGAGCATCTGGTTACTGGT

GAAACAATGCAAATGACACCTACATCTGATCAACGTTGTAGCATGCAGTACTATTTGCAGAAAACATTCTACAAGACAGC

CTCATTGATTTCTAACAGCTGCAAAGCTATTGCCCTTCTTGCTGGGCAAACAGCAGAAGTTTCAATGTTTGCTTATGATT

ACGGTCGAAATCTGGGATTGGCGCATCAATTGATTGATGATGTACTTGATTTCACTGGCACATCTGCTTCCCTTGGAAAG

GGTTCCTTGTCTGACATTCGCCATGGTATCGTGACAGCTCCTATATTATATGCCATTGAAGAATTCCCTCAACTACGTCC

ACTAGTTGATCGGGGATTTGACAACCCATCAGATATTGATCTTGCCCTTGACTACCTTGGAAGAAGCCAGGGTATACAGA

GGACAAGGGAACTTGCAGCAGAACACGCCAGCCTTGCTGTGGAAGCAATTGATTCTTTTCCTCAAAGTGATGATGAGGAT

GTCCGACTATCCAGGAGGGCGCTCGTTGATCTAACCCAGATAGTCATTACAAGAACAAAATAA

>Cbur0G019030.1 gene=Cbur0G019030

ATGTTTTCGTTGAAGCTGACGATTTTAGAATTGGAACCGCCCCTACTCTGGAAGATGGGCCACATCATCAATGGCTGCCA

TCCTAAACCATTTCACAAGCAATCCAATATTCCTCTCCTCCCCCTAAACAAGCTAAAACCCCTCTCATCTTCTGCAAAGA

CCTTTCAAGTGACCACTACATACAGTAACCCAACCAACTGCTCCACCATAAAGGCAGACGTGGACGCCCGCCTCAAGCAG

GCCCTCCCAATCCGCGACCCCCTCGACGTCCATCGACCTATGCACGATCTTGTCTTCTCCGCGCCCAAGACCTTGGCTCC

AGCTCTCTGCACTGCAGCATGCGAGCTCGTGGAGGGGAGCGTAAAGACGGTTGTGGACGTCGCCTCGGCCCTAAACCTCA

TGCTGGCTGCTTCCTACACCCATCAATACCTCCCCAACCTTCTACAGAGGCCAAATTCCCCATCCTTTCCTCACAACATT

GAGCTTCTTACAGGGGATGGCATCTACCCTCTTGGGTTCGAGCTGCTAGCCAACCCTGATGGTGTGGGGGGTGATGCTGA

GCGCGCTCTGAGGGTAATTTTGGAGGTCGCACGTGCCATGGGTGGGCAGGGGTTAGTAGAGGCACAGTACATGAAGGTGA

AGTGGGCCAAGGGGGAACTGGACGGTGACTGCGTGTTGATGCGTATGTGCGAGAGAGGAGAAGGCAGTGTTTACTCATGT

GCAGCGGCATGTGGAGCTATTGTAGGTGGGGCCTGCGAAGAGGATGTGGAAAAGTTAAGGAGGTACGGGTTTTACGTGGG

TATGATCCATGGGCTGTTGGATGGTGTTGGCAGAGAAGGCGATGTGGAAGAAGAAGTGCAAAGGCTTAGGTCGTTGGCTC

TCAAGGAGTTGGAAGGGTTTGATGCAGAGAAGGTCGGGGCCATTTCCAGCCTAGTTGATGCTGATGCCTCTCGCTGTGCA

CAGTAG

>Cbur12G004970.1 gene=Cbur12G004970

ATGGCTGCCATCCTAAACCATTTCACAAGCAATTCAATACGTCCTCTCCTCCCCCTAAACAAGCTAAAACCCCTCTCATC

TTCTGCAAAGACCTTTCAAGTGACCACTACATACAGCAACCCAACCAACTGCTCCACCATAAAGGCAGACGTGGACGCCC

GCCTCAAGCAGGCCCTCCCAATCCGCGACCCCCTCGACGTCCATCGACCTATGCACGATCTTGTCTTCTCCGCGCCCAAG

ACCTTGGCTCCAGCTCTCTGCACTGCAGCATGCGAGCTCGTGGGAGGGGAGCGTAAAGACGGTGTGGACGTCGCCTCGGC

CCTAAACCTCATGCTGGCTGCTTCCTACACCCATCAATACCTCCCCAACCTTCTACAGAGGCCAAATTCCCCATCCTTTC

CTCACAACATTGAGCTTCTAACAGGGGATGGCATCTACCCTCTTGGGTTCGAGCTGCTAGCCAACCCTGATGGTGTGGGG

GGTGATGCTGAGCGCGCTCTGAGGGTAATTTTGGAGGTCGCACGTGCCATGGGTGGGCAGGGGTTAGTAGAGGCACAGTA

CATGAAGGTGAAGTGGGCCAAGGGGGAACTGGACGGTGACTGCGTGTTGATGCGTATGTGCGAGAGAGGGGAAGGCAGTG

TTTACTCATGTGCAGCGGCATGTGGAGCTATTGTAGGTGGGGCCTGCGAAGAGGATGTGGAAAAGTTAAGGAGGTACGGG

TTTTACGTGGGTACGATCCATGGGCTGTTGGATGGTGTTGGCAGAGAAGGCGATGTGGAAGAAGAAGTGCAAAGGCTTAG

GTCGTTGGCTCTCAAGGAGTTGGAAGGGTTTGATGCAGAGAAGGTCGGGGCCATTTACAGCCTAGTTGATGCTGATGCCT

CTCGCTGTGCACAGTAG

>Cbur12G014500.1 gene=Cbur12G014500

ATGAGTTCTTCAGTGCATCTAGCTTGTACCCTGGTTCCTCAAACCTGCTCCACATTTACTCATTCAAAGGAGGTGAAATC

TACAACCCATTCCAACTTTAATCAATGGAGATGGAGATTACAAACACAACTCAATCAGCCCAAGTTTCTGTCAGGGATTA

TAGTAAAGAAAAGGGCACTAAAGACCCCTTTCTTCACTGCAAAAGCACATGTGATACAAAGAGAAAAGGAAAATGATTCT

TCCTATTCCAAAACTAGCCCTCCCTTTGATCTCAAAACCTACATGCGCCAGAAGGGCAATTCCGTCAACAGTGCCTTAGA

AGAGGCCGTCCGCCTCCGCTCGCCGGAGGCCCTGTACGAGCCCATGCGCTACTCCCTCTTCGCCGGCGGCAAGCGCGTAC

GGCCGCTCCTCTGCATCGCCGCCTGCGAGCTCGTCGGCGGCAACGAGGCCACAGCCATGCCCTCCGCCTGCGCGGCGGAG

ATGATCCACACCATGTCGCTCATGCACGACGACCTCCCCTACATGGACGACGACGACCTCCGCCGCGGGAAGCCCACCGC

CCACAAGGCCTTCGGCGAGACCGCCGCCGTCCTCTCCGGCGACGTCATGCTGCTCCTCGCCTTCGAGCACGTGGCTGCGG

CGACGCCCGGAGCGCCACCCGACCGCGTGGCCCGGGCAGTCGGAGAGCTGGCGCGGGCTGCCGGACGGGACGGGCTGGTG

GCGGGGCAGATAGTCGACGTGTGCTCTGAGGGTTCGGGGGAAGTGGATCTGGAGCGGCTGGAGTATATTCACTTGCACAA

GACGGCGGCGCTGCTGGAGGGGGCGGTGGTGGCGGGGGGAATGCTGGGTGGCGGGACGGAGGAGGAGATAGGGCGGCTGA

GGAGGTTTGCGAGGTGTATAGGGCTGCTGTTTCAGGTGGTGGATGATATATTGGACGTGACGAAGTCGTCGACGGAGCTG

GGGAAGACGGCGGGGAAGGACGTGGCGGCGGAGAAGGTGACTTATCCGAAGATGTTGGGGTTGGAGGGGTCGAGGGAGCT

GGCGGAGGAGCTGAGGAAGGAGGCGAAGGAGCAGCTGATGGGGTTCGATCCGGAAAAGGCGATGCCTTTGGTTGCTTTGA

CAGATTTCATTGCTTGCAGAAACAACTAG

>Cbur12G014320.1 gene=Cbur12G014320

ATGAGCGGTCCAGATCTCACAAACGCATACCACGTGAATAATAGTGGAGGCAGACACAGCTCCATTTTCCCACACCAGCA

CCAAACATCAGAAAACGACAAAACGCGCGCTCGTAGTAGAAAAAACGGATGGCTACGAGATAAAATGCGCATCACACACA

CACACAACTTACAACTGCGACGGCATCTTCTTCGTCCCTCTGCTAACGTGCGCCTGCTTCCATCCATGGCGACGGCTCTG

CAATTCACGCTCTCCACCCACCGCTCCGACCTCCTCCTCGCAAGAGCTGCTTTTGGGATCCGATCCGGGACCAGGAAACC

AATCGCCACCGTTCGATGCTGCAGCAGCAGCAGCAGCGCCAGCGGCACCGAATCCGCCGTCGAGTCCGAGTTCGACGCCA

AGGTGTTTCGGAAGAACCTGACACGAAGCAAGAACTACAATCGGAAGGGTTTCGGACACAAGGAGGAGACGATGGAGCTG

ATGAATCTGGAGTACACGAGCGATGTGGTAAAGACGCTGAGGGAGAACGGGAACGAGTATACGTGGGGAAACGTAACGGT

GAAGCTGGCGGAGTCGTACGGATTCTGCTGGGGTGTTGAACGTGCGGTCCAGATCGCGTATGAGGCAAGGAAGCAGTTCC

CGGAGGAGAAGATCTGGATTACCAACGAGATTATCCATAATCCAACTGTTAATAAGAGATTAGAAGAGATGGACGTCAAG

AATATTCCAATTGAGAATGGGAAGAAACAATTTGATGTTGTTGAGAAGGATGATGTTGTGATTTTACCTGCTTTTGGAGC

TGCTGTGGAAGAGATGTTGACTTTGAGCGAAAAGAACGTACAAATAGTTGATACAACTTGCCCATGGGTGTCTAAGGTCT

GGAACACTGTTGAGAAACACAAGAAGGGGGAGTACACTTCAATAATTCATGGTAAATATTCTCATGAAGAGACAGTTGCA

ACTGCATCTTTTGCAGGGAAGTATATTATTGTTAAGAACATGGCAGAGGCAATGTATGTATGTGATTACATTCTTGGAGG

TAAACTTGATGGATCTAGTTCAACGAAAGAAGAGTTTATGGAGAAATTCAAATATGCAGTTTCCAGCGGGTTTGATCCAG

ACATTGATCTAGAAAAGGCTGGCATTGCAAATCAAACTACAATGCTTAAGGGAGAGACTGAAGAGATTGGTAAATTGGTT

GAGAAGACAATGATGCGCAAGTATGGGATAGAAAACATCAATGAGCACTTCGTGAGTTTCAATACGATTTGCGATGCTAC

ACAGGAGCGACAAGATGCAATGTACAAGCTGGTGAAGGAGAAACTAGATCTCATGTTAGTGGTTGGTGGATGGAACTCCA

GTAACACCTCTCATCTACAAGAGATTGCAGAGCACTATGGCATTCCATCTTACTGGATTGATAGTGAAAAGAGAGTAGGA

CCAGGAAACAGAATAAGCTACAAGTTGAATCATGGAGAGCTGGTTGAGAAAGAGAACTTTTTACCAGAAGGTCCCATCAC

AATTGGGGTAACTTCAGGTGCTTCGACTCCAGATAAGGTAGTTGAGGACGTCCTGAACAAGTTGTTCGACATCAAACGTA

AAGAAGCTTTACAGTTAGCCTAA

>Cbur11G007560.1 gene=Cbur11G007560

ATGGCTACTGGGACAGTTCCGGCTTCAATTTCTGGTTTGAAGACCAGGGATCATGGTTTAAGCTTCGCAAGAAGTGTAGA

TTTTGTGAAGGTAACATGCTTGCCTTCACAGAAGATCAAGTCTCAGAGAGATAATATATCTGTTATCAGAAACTCAAAAC

AAGGTCCTGAAACCATTGAGTTGCAGTCTGCTTCTGAGGGAAGCCCTCTGCTAGTTCCTAGGCAGAAGTACTGTGAATCA

GTGCACAAGACTATCAGGAGAAAAACTCGGACTGTGATGGTAGGGAATGTAGCTCTTGGTAGTGAGCATCCTATAAGGAT

TCAGACGATGACTACTAGCGATACAAAAGATGTTGCTGGAACTGTTCAGGAGGTAATGCGGATAGCGGACAAGGGAGCAG

ATATTGTTCGGATCACAGTTCAGGGGAGGAAAGAAGCAGATGCATGTTTTGAAATAAAGAACACCCTAGTCCAGAAGAAT

TATAACATTCCTCTGGTGGCCGACATTCATTTTGCTCCTCCTATAGCTTTGAGAGTTGCTGAATGTTTTGACAAGATTCG

AGTCAACCCGGGAAATTTTGCTGATAGGCGGGCTCAGTTTGAGACGCTGGAGTATACTGAAGATGATTATCAAAAGGAAC

TTGAGCATATTGAGCAGGTTTTCTCTCCATTAGTTGAGAAATGTAAAAAGTACGGAAGAGCAATGCGCATTGGAACAAAT

CATGGAAGTCTTTCTGACAGGATTATGAGCTACTATGGGGATTCTCCTAGGGGGATGGTTGAATCTGCCTTTGAGTTTGC

AAGGATTTGCCGCAAGTTGGACTTCCACAACTTTGTCTTCTCAATGAAAGCAAGCAACCCAGTTGTCATGGTTCAAGCAT

ACCGCTTACTGGTTGCAGAAATGTTTGTTCAGGGTTGGGACTATCCTCTACACTTGGGAGTCACTGAAGCTGGTGAAGGT

GAGGATGGACGGATGAAATCTGCAATTGGCATTGGAACCCTTCTTCAGGATGGTTTGGGGGACACAATCCGTGTTTCCCT

CACAGAACCACCAGAGGAAGAGATAGATCCTTGCAGAAGACTAGCCAACCTTGGCATGCAAGCTTCAAAGCTTCAGAAGG

GGGTGGTACCATTTGAAGAAAAACACAGACGTTATTTTGATTTTCAGCGTAGAACTGGTCAATTACCAATTCAGAAAGAG

GGTGAAGAGGTAGACTACCGAGGAGTCTTGCACCGCGATGGTTCTGTTCTTATGTCTGTGTCTCTAGATCAGTTGAAGAC

ACCCGAACTCCTCTATAAATCACTTGCAGCGAAACTTGTTGTCGGCATGCCATTTAAGGATCTGGCAACAGTTGATTCAA

TTCTTGTGAGAAAGCTTCCTCCAGTAGAAAACACTGATGCTAGGCTTGCACTCAAAAGGCTGATAGATATAAGCATGGGA

GTTTTGACTCCATTGTCAGAGCAGCTTACGAAGCCCTTACCTAATGCCATGGTCCTAGTGAATCTTAAGGAACTGGCAAG

CGGTGCCCACAAACTTTTACCATATGGCACGCGCTTGGCAGTATCTGTTCGTGGTGATGAACGCTATGAGGAGCTGGATA

TACTTAAAGATGTGGAAATAACAATGCTTCTACACAATCTACCATTTTCTGAAGAGAAAAACAGCAGAGTACATTCAGCA

AGGAGGCTGTTTGAGTATCTGTCAGACAATTCTCTGAACTTCCCTGTAATTCACCACATGCAGTTTCCTAAAAGGATACA

CAGAGATGATCTAGTGATTGGTGCTGGAAGCAATGTGGGAGCCCTTCTGGTGGATGGCCTTGGAGATGGCTTATTCTTGG

AGGCTCCTGATCAGGACTTTGATTTTCTGAGGAACACATCCTTCAATTTGCTCCAAGGTTGCAGAATGCGAAATACAAAA

ACGGAGTATGTTTCATGCCCATCCTGTGGTCGTACACTGTTTGACCTCCAAGAAGTGAGTGCTGAGATAAGAGAGAAGAC

AGCCCATTTGCCTGGTGTTTCGATTGCAATCATGGGTTGCATTGTTAATGGGCCAGGAGAGATGGCAGATGCAGATTTTG

GGTATGTTGGAGGTGCTCCAGGAAAGATTGACCTCTACGTTGGGAAGACTGTGGTGAAGCGAGGGATTGAGATGGCACAT

GCCACGAATGCATTGATTCAGCTGATTAAAGACCATGGCCGCTGGGTGGATCCACCAGCTGATGAGTAG

>Cbur10G025900.1 gene=Cbur10G025900

ATGGCTGCTGGAACAGTTCTAGCTTCAATTTCTGGTCTGAAGACCAGTGATCATGGTTTAAGCTTTGGGAAAAGTGTAGA

TTTTGTGAAGGTTACCTGCTTGCCTGCTCAGAAGAATAAGTGTCGGAGAGATTATGTATCAGTTATAAGAAACTCAAAAC

CAGGTCCTGAGACCATTGAATTGCAGCCTGCTTCTGAAGGAAGCCCTCTCCTAGTCCCCAGGCAGAAGTACTGCGAATCA

GTGCACAAGACTGTAAGGAGAAAGACACGGACTGTGATGATAGGCAATGTGGCTCTTGGTAGTGAGCATCCCATACGAGT

TCAAACGATGACTACCAGCGACACAAAAGATGTTGCTGGAACCGTTGAGGAGGTAATGAGAATAGCTGACAAAGGAGCAG

ATATTGTTCGGATTACAGTTCAGGGTAGGAAAGAAGCAGATGCTTGTTTTGAAATAAAAAACACCTTGGTCAAGAAAAAT

TATAATATTCCTCTGGTAGCAGACATCCATTTTGCTCCTCCTGTCGCTTTGAGAGTTGCAGAATGTTTTGACAAGATCCG

TGTCAACCCAGGAAACTTTGCTGATAGGCGGGCTCAGTTTGAGAAGCTGGAGTACACGGAAGAAGATTATCATAAGGAGC

TTGAGCATATTGAGCATGTTTTCTCTCCATTAGTTGAGAAATGTAAAAAGTATGGAAGAGCAATGCGTATTGGAACTAAT

CACGGAAGTCTTTCTGACCGTATCATGAGCTATTATGGTGATTCTCCTAGGGGAATGGTTGAATCTGCCTTCGAGTTTGC

AAGGATTTGCCGCAAGTTGGACTTTCACAACTTTGTTTTCTCAATGAAAGCAAGCAACCCAGTAATCATGGTTCAAGCAT

ACCGCTTACTGGTTGCTGAAATGTATGTTCAAGGTTGGGACTATCCTCTGCACTTGGGAGTCACTGAAGCAGGAGAGGGT

GAAGATGGACGGATGAAATCTGCAATTGGCATTGGCACCCTTCTTCAGGATGGTTTGGGGGATACTATCCGTGTTTCCCT

CACAGAACCACCAGAGGAAGAGATAGATCCTTGCAGGAGACTAGCCAACCTCGGCATGCAAGCCTCAAGTCTTCAGAAAG

GGGTGGTACCATTTGAGGAAAAACACAGACACTATTTTGATTTCCAGCGTAGAACTGGTCAACTACCAATTCAGAAGGAG

GGTGAAGAGGTAGACTACCGAGGAGTCCTGCACCGCGATGGTTCTGTTCTTATGTCTGTGTCTCTAGATCAATTGAAGAC

ACCTGAACTCCTCTACAAATCACTTGCAGCTAAGCTAGTGGTTGGCATGCCTTTCAAGGATCTGGCAACAGTAGACTCAA

TCCTTGTGAAAGAACTTCCTCCAGTAGAAGACACCGATTCTAGGCTAGCACTCAAAAGGCTGATAGATATAAGCATGGGA

GTTCTGACTCCATTGTCAGAGCAGCTTACCAAGCCGTTGCCAAACGCTGTGGTCCTACTTAATCTTAAGGAACTGGAAAC

CGGTGCCCACAAACTTTTACCAGAAGGCACTCGTTTGGCAGTATTGGTTCGTGGTGATGAACCCTATGAAGAGCTGGATG

TACTGAAAGGTGTGGATATTGTAATGCTTTTACACAATCTTCCATGTTCTGAAGAGAAAAACAGCAGAGTGCATGCGGCA

AGGAGGTTGTTTGAGTATTTATCGGAAAACTCTCTTAACTTCCCTGTTATTCACCACATGCAGTTTCCTGAAGGGATTCA

CAGAGATGATCTAGTGATCGGTGCTGGTAGCAGTGTGGGAGCTCTTCTGGTCGATGGCCTAGGTGATGGTTTATTCTTGG

AAGCTCCTGACCAGGATTTTGATTTTCTGAGGAACACATCCTTCAATTTGCTCCAAGGTTGCAGAATGCGGAATACAAAA

ACGGAGTATGTTTCGTGTCCATCCTGTGGTCGTACTTTATTTGACCTCCAAGAGATAAGCGCTGAGATAAGAGAGAAGAC

AGCCCATTTGCCTGGTGTTTCGATTGCAATCATGGGTTGCATCGTAAATGGGCCAGGAGAGATGGCAGATGCAGATTTTG

GATATGTAGGCGGTGCTCCTGGAAAGATTGACCTATACGTGGGGAAGACTGTAGTCAAGCGAGCAATAGAGATGGCACAA

GCCACTGATGCATTGATTCAGCTGATTAAAGACCATGGACGTTGGGTGGATCCACCGACTGATGAGTAA

>Cbur03G016350.1 gene=Cbur03G016350

ATGGCGATGGCTTTCATGTCTCTAATCCCCACCCAAACCCGCTCTACTTCCCTCTCCTTTCCTTCGTCCCCCATCTTCTT

TTCCTCGCAATCTCCCTCTCATCGTCTCCTCCCTCGCCCCATCAACCCTTCTTCTCCTCCTCCTCCTCCTCCTTCGGTCT

CAATCCGAGCCTCCATCTCTCCAAACCCTCCAAGAGCTGCAACCACTGCTTTTGATCTCCAGAAAGATGTCGCCCTCGAA

AGATCAGCTCCTTCTCTGCCTTTTCGGATAGGACACGGCTTCGACCTTCACAGGCTCGAGCCAGGGTACCCTCTCATCAT

CGGAGGCATTAGTATTCCTCACGACAGGGGCTGCGAAGCTCATTCAGATGGTGATGTCTTACTTCACTGTGTGGTGGATG

CGATCTTGGGGGCGTTGGGGCTTCCAGATATCGGGCAGATTTTTCCGGACACCGACCCCAAATGGAAGGGGGCTGCTTCC

TCTGTATTCATGAAAGAAGCGGTTAGGCTCATGCATGAAGCAGGCTATGAGCTTGGAAACTTGGATGCTACTCTGATTCT

GCAGAGACCGAAATTGAGCCCGCACAAGGAGTCCATCAGGGCAAACCTATGTGAGCTTCTTGGAGCAGACCCATCCGTTG

TTAACCTCAAAGCAAAAACTCATGAGAAGGTTGATAGCCTTGGAGAAAACCGAAGCATTGCAGCACATACTGTTGTTCTT

CTGATGAGGAAGTGA

>Cbur06G015920.1 gene=Cbur06G015920

ATGGCGCTTCGATTCGATGTTCCACTCTCTTTTCCTCTCATCAACAATCTCCATTCCCACCCAAAATCCCACCGATTTCT

TCCTTTTCTCGCAAATCCCAATCCTCCTCCTTCACCATTTCTAAGATTACAACGTCCAATTCCAGGCCACCAAGATGTGT

TCAGGAGGAGAGTGAGAATTAATTGCTCCGTGGCAAAAACCCCAGAAAAGAATGAGGTTTCTCAGATTGTGAAAGAGAAG

AGCGTTTCAGTGGTTCTGTTGGCGGGAGGAAAAGGTAAAAGGATGGGGGCAAGCATGCCGAAGCAGTACCTCCCACTCTT

TGGCCAACCAATTGCTTTGTATAGTTTCTACACTTTCTCTCGCATGGTTGAAGTGAAGGAAATAGTTGTAGTGTGTGATC

CATCCTACAAAGACATATTTGAAGATGCCAAAGCAGAGATCCATGTAGACCTTAAGTTTGCTCTTCCTGGGAAGGAAAGA

CAGGATTCTGTGTTCAATGGATTTCAGGAAATTGATGCAAGCTCGGAGCTCGTTTGCATCCATGATTCAGCGAGGCCTTT

GGTATTATCTGGAGATATTGAAAAGGTGCTGAAAGATGGGCTGATGAATGGTGCAGCTGTGCTTGGTGTTCCAGTTAAAG

CAACAATCAAAGAGGCAAATAGCAAATCCTTTGTAGTGAGAACTCTGGACAGGAAAACGCTTTGGGAAATGCAAACTCCT

CAGGTCATCAAGCCTGACTTGCTTAGAAATGGTTTTGAGCTAGTGCAAAGTAAAAGTCTTGAAGTCACTGATGATGTGTC

CATTGTGGAGCACCTTAAGCATCCTGTCTACATCACGGAAGGATCTTACACAAACATCAAGGTTACAACCCCAGATGATC

TGTTACTTGCAGAGAGGATATTGACGAAGCAGGAGAGCCTTTGA

>Cbur09G010280.1 gene=Cbur09G010280

ATGGCTTTGAAATTCCCCCTGCAGGCAGACATCGGAGGAATCTCCTTCTTGGATTCCAGCAAAGGAAGCCTTTTGAAGCT

CAAAGGAGGATTTTATTTGAAGAGGAAGGAGAATGGAATGCCACATGTAAGGTTGACTCGCTGTTCTGCCCAGGTGCCCC

CACCGGCATGGCCTGGACGAGCCGTTGTAGAGCCAGGACGGAAGGTGTGGGATGGTCCCAAGCCTATCTCGATTGTTGGA

TCCACTGGTTCCATTGGAACTCAGACTTTGGACATAGTAGCTGAGAACCCGCACAAATTCAGAGTTGTTGCACTGGCAGC

CGGTTCAAATGTGACTCTTCTTGCGGATCAGGTGAAGACGTTCAAACCTCAACTGGTTGCTGTTAGGAATGAGTCTTTAG

TTGATGAACTTAAAGAGGCTTTGGCGGATGCTGAATACAAGCCTGAGATAATTCCTGGAGAGGAGGGTGTCATTGAAGTT

GCACGTCACCCAGATGCTGTCACGGTAGTCACAGGAATAGTAGGATGTGCAGGTTTGAAGCCTACAGTAGCTGCAATTGA

GGCTGGAAAAGACATAGCATTGGCAAACAAAGAGACTCTGATTGCAGGCGGTCCCTTTGTACTTCCTCTTGCACAAAAGC

ATAAAGTAAAAATACTTCCTGCTGACTCAGAACATTCTGCTATATTCCAGTGTATTCAAGGCCTGCCAGAGGGTGCACTT

CGGCGCATTATTTTGACTGCTTCCGGAGGGGCTTTCAGGGATTTGCCTGTTGAAAAACTCAAGGAAGTGAAAGTTGCTGA

TGCTTTAAAGCATCCTAACTGGAATATGGGAAAGAAGATCACAGTGGATTCTGCCACCCTCTTCAACAAGGGTCTTGAAG

TTATTGAAGCCCACTATCTGTTTGGAGCTGAATATGATGATATTGAGATTGTGATTCATCCCCAGTCAATCATACACTCA

ATGGTTGAGACCCAGGATTCATCAGTTCTTGCTCAGTTGGGATGGCCTGATATGCGCCTGCCAATTCTTTACACAATGTC

GTGGCCAGAGAGAATTTATTGCTCTGAAACCACCTGGCCTCGGCTCGACCTTTGCAAGTTGGGCTCTCTGACATTTAAGG

CTCCTGATAATGTGAAATACCCATCCATGGATCTTGCTTACTCCGCTGGGCGTGCTGGAGGTACGATGACTGGAGTTCTT

AGTGCAGCTAATGAGAAGGCTGTGGAGATGTTCATTAATGAGAAAATCTGCTACCTGGACATTTTCAAGATTGTGGAACT

CACATGTGCCGAACACCAGAAAGAGCTAGTAACCAGTCCCTCCTTGGAGGAGATCATTCATTATGATTTGTGGGCTCGGG

AGTTTGCTGCAAATTTACTTCTGTCTTCTGGGAGAAGACCCGTCCTGGCTTAA

>Cbur04G023450.1 gene=Cbur04G023450

ATGGCATCTTCTGCAGTTCTGATTAGGAATTGGATGCTTCCACTGCCTGATGTTGGTGGGAGTGAATTCTCATGGAGGAA

GCTTATTCACACCAGAAGATCTCTTCCCATTAAGAAACCCAAGCAGCTATATGCAGTTGCCAAGGCCAAGGACAACAGCT

CAGGTGGGGAGAATAGCATCTCTCTAAGGAATGAGCAGCAACAGATCCCTAAGACAATTGATTTCTCTGGAGAAAAGCCT

CCCACTCCAATCTTAGACACCATCAACTACCCAATTCACATGAAGAATCTCTCTGTCAAGGAACTGGAGTTGTTAGCGGA

CGAGCTGAGAGCAGAGATCATATACACAGTGTCGAAGATCGGTGGGCACCTAAGTGCGAGCCTAGGGGTGGCAGAGCTAT

CTGTGGCCCTGCATCATGTGTTCAACACCCCAGATGATAAGATCATTTGGGATGTTGGCCATCAGGCTTACCCACACAAG

ATTTTGACGGGTAGGAGATCAAGACTGCATACAATCAGACAGACTTCAGGGCTAGCAGGCTTCCCTAAGAGGGATGAGAG

TGTGCATGATGCATTTGGAGCTGGCCACAGTTCTACAAGCATCTCAGCTGGTCTAGGAATGGCAGTAGGTAGGGACCTAC

TAGGGAAGAAGAATCATGTAATTTCTGTCATCGGCGATGGAGCCATGACCGCCGGACAGGCCTATGAGGCGATGAATAAT

GCAGGTTATCTTGATTCAAATATCATAATCATCTTAAATGACAACAAGCAAGTTTCACTTCCCACGGCCACAGTGGATGG

ACCTGCACCCCCTGTAGGAGCTCTAAGTAGAGCTCTCACCAGGCTCCAATCCAGCACCAAGTTCCGCCAGCTTCGTGAAG

CAGTTAAGGACATTACAAAGCAAATTGGAGGGCAGACGCATCAGGTTGCAGCAAAAGTTGATGAATATGCAAGGGGTATG

ATAAGCGGGAATGGAGCAACATTTTTTGAGGAGCTGGGGCTGTACTACATTGGTCCAGTTGATGGACACAACATCGAAGA

CCTTGTGCACATCTTAGAGAAGGTGAAGGCCACACCCGCGCCCGGTCCTGTTCTCATCCATATTGTCACAGAGAAAGGAA

AAGGCTATGCCCCAGCTGAAATCGCCGCAGATAAAATGCATGGTGTTGTTAAATTCGATCCAAAGACAGGGAAGCAATTT

AAGGCTAAAAGTCCAACCAAAGGATACACTCAATACTTTGCAGAGGCTCTAGTCGCAGAGGCAGAGAATGATGATAAAAT

TGTAGCAATCCATGCGGCCATGGGAGGCGGCACGGGTCTTAATCTATTCCAGAAACAATTCCCTGACCGATGTTTTGACG

TTGGGATCGCCGAGCAACATGCTGTAACATTTGCTGCGGGCCTAGCTACAGAGGGGCTTAAGCCTTTCTGTGCAATCTAT

TCTTCTTTCCTACAAAGAGCTTATGATCAGGTGGTACATGATGTGGACCTTCAGAAGCTTCCTGTGCGATTCGCCATGGA

TAGGGCCGGCCTGGTAGGAGCAGACGGTCCCACCCATTGCGGGGCCTTCGATGTTGCTTACTTGGCATGCTTACCCAACA

TGGTGGTCATGGCCCCTTCTGATGAAGCTGAATTGATGCACATGGTCGCTACGGCTGCAGTCATCGACGACAGGCCAAGT

TGCTTCAGGTACCCAAGAGGAAACGGTGTTGGATCGCCCCTCCCGCCAAATAACAAGGGGGTTCCTCTAGAGATTGGAAG

GGGACGGGTTTTGAAGGAAGGAAGTAAGGTGGCCATTTTGGGTTTTGGGGCGATAGTACAGAACTGCATTGGAGCAGCAG

AGCTCCTCCAAGAGCACAAGGTTTCTGCCACTGTCGCCGATGCCCGCTTTTGCAAACCCCTCGACAGCGAGCTGATTAAA

AGACTTGCTCGAGAGCATGAGATACTCATAACGGTTGAAGAAGGATCGATTGGCGGTTTTGGCTCCCATGTCTCCCACTT

TTTGGCCTTGAATGGGCTCCTGGATGGAAACCTCAAGTGGAGGGCCATGACGCTGCCAGACCGATACATCGACCATGGAT

CTCCAAAGGACCAGATTGAAGAGGCAGGGCTTACTTCCAAGCACATTGCGGCCACAGTATTGTCTCTCATCAGTGGTAAC

AAGGATGCTCTCCATCACCTTCGGGTGTAA

>Cbur05G010500.1 gene=Cbur05G010500

ATGCATGCATCGAAATTATTTTTAGTGCCTACCCACAAGGATAAGAATCTTACGAGGGATTTGAAGAGATTTGATAATTT

GTCACTTTCTCCAAACCGTGCCTCATGGCTATATAGTACAAGACCTCGTCCTAGTTTCCTGTCTGCATCTCTCTCATTGC

CGGTTACTACTTCAAAGAAGCTCAGCATTCCGTCAGCAATGGCAGTGTTTTTGTCTCCCATGTACAGACTTGGAACTTTC

TCTCCATTCCTGCAACCTCCATGCTCACCACCCAACTGTAAAAGACAGTACTATGTGAGAGCTGCTGGCTCGGATGATGA

GGGGAAGGTTGTTACAAGAAAAGAAAAAGGTGATTGGAATATTGATTACTCTGGAGAGAAGCCTACAACTCCCTTGTTGG

ATTCAATCAATTACCCACTTCATATGAAGAATCTTTCTACAGAGGATCTTAACCAGTTAGCTTCAGAGCTTAGAGCAGAG

ATAGTTCATACGGTGTCCAAGACAGGTGGACATCTGAGTGCCAGTTTAGGGGTGGTGGAGCTGTCAGTGGCTCTGCATCA

TGTATTCAACACCCCTGCAGATAAGATCATATGGGATGTTGGTCATCAGGCATACACACATAAGATTTTGACTGGAAGAA

GGTCTACAATGCATACCATAAGACAAACTTCAGGGCTTGCAGGGTTTCCAAAAAGGGATGAGAGCATTCACGATGCCTTT

GGTACAGGGCACAGCTCTACAAGCATTTCTGCAGGCCTTGGAATGGCAGTGGCAAGAGATCTACTTGGGAAGAGCAACCA

TGTTATCTCTGTAATTGGAGACGGAGCTATGACTGCTGGACAAGCGTATGAGGCCATGAACAATGCTGGATACCTTGATT

CCAATCTAATTATAGTGTTGAATGACAACAAGCAAGTATCCCTTCCAACGGCCACGCTCGATGGCCCTGCAACTCCAGTG

GGAGCTCTCAGCAGAGCTCTTACTAGGCTTCAAGCAAGCCCTAAATTCCGCAAGCTACGCGAAGCGGCAAAGAGTGTCAC

AAAGCAGATTGGGGGGAAGACTCATGAGGTTGCAGCTAAGGTGGACGAATATGCAAGGGGATTCATCAGTGCCACTGGGT

CTAGTCTGTTTGAGGAGCTTGGGCTGTATTACATAGGTCCTGTAGATGGGCACAATGTTGAAGATCTTGTTACTATATTA

GAGAAGGTTAAGACAATGCCTGCACCAGGGCCTGTTCTAATCCACATTGTCACAGAAAAAGGAAAGGGATATCCACCTGC

AGAAGCAGCAGCTGATAAAATGCATGGTGTTGTCAAGTTTGACCCAAAAACTGGGAAACAATTTAAAAACAAGTCCTCTT

CACTTTCATACACCCAGTACTTTGCAGAGTCCCTAATAAAAGAAGCAGAGGAAGATAGCAAGATCGTAGCGATTCATGCT

GCTATGGGTGGAGGAACAGGACTTAATTTATTTCAAAAGAAGTTCCCAGATCGTTGCTTTGATGTGGGGATAGCAGAGCA

GCATGCAGTTACATTTTCTGCAGGTTTAGCGACAGAAGGTCTTAAGCCTTTCTGTGCCATCTACTCGTCATTTCTTCAAC

GGGGTTATGATCAGGTGGTACATGATGTGGATCTTCAAAAACTGCCTGTTCGATTTGCATTGGACCGGGCCGGTTTGGTC

GGGGCAGACGGGCCTACTCACTGTGGAGCATTCGACATCACATACATGGCTTGCTTGCCCAACATGATTGTAATGGCTCC

ATGCGATGAAAGTGAATTGATGCACATGGTAGCCACAGCAGCAGCTATTGATGACAGACCAAGCTGCTTTAGGTTCCCAA

GAGGTAATGGCATTGGAGTTGATCTCCCTTCTGATAATAAAGGCACGCCATTGGAGATCGGCAAGGGAAGAATTCTGTTG

GAAGGCACCAGGGTTGCAATTCTTGGATTTGGATCTATAGTCCAGAGTTGTGTTGAAGCTTCCATTATGCTTAAGTCCCA

TGACGTCTCTGTGACGGTTGCTGACGCTCGCTTCTGCAAGCCTTTGGATGCAGATCTCATCAGAAGATTGGTAAATGAGC

ATGAGTTCCTGCTCACAGTTGAAGAAGGTTCAATTGGAGGCTTTGGTTCTCATGTTCTTCATTTCCTAAGCCTCAGCGGC

CTTTTAGATGGAAATCTCAAGTTTAGAGTAATGACTCTTCCAGACAGATATATAGACCATGGATCTCCCCAAGATCAGAT

TGAAGAAGCAGGACTTTCTCCAAGGCACATTGCTGCAACAGTAATGTCTCTTTTGGGGATGCCCAAAGAAGCTCTTCAGT

TCAAGTGA

>Cbur02G033440.1 gene=Cbur02G033440

ATGGGTTCTGCTTCTGTTCAGTACCCATCTGGGATTAATACTCTGTTGCAAGGGAATTTCAGGGCTCTATCTCCCTCTCC

CAAATTGGATTTCTTAAGGGTTTCTCATCACATAGGAGGGGAATCTCCCAAGATAAATCTCTACCCATGTTCTACTTTTA

GCAGTTCTTCCAAGGGCTCTGTTGTTCGAGTTGGTTCTCTCCCCAACCTTGATGATTTTTTCTGGGAAAAGGATCCAACA

CCAATGTTGGACATGGTTGAAACTCCAATGCATTTGAAGAACATGTCTCACAAGGAGCTGAAACAGTTAGCAGATGAAAT

CCGTTCAGAGATAATATTTACCTTGTCGAGAACGAGGAGACCCTTTAAATCTAGCCTTGCAGTCGTGGAGCTGACCGTTG

CAATACATCATGTTTTCAATGCCCCAGTGGACAAGATACTATGGGATGTTGGTGAACAGACATATGCACATAAGATTCTC

ACTGGCAGGCGATCTCTTATGCATACGCTGAGGCAGAAAAACGGCCTATCAGGCTTTACATCTCGGTCTGAGAGTGAATA

TGATGCATTTGGTGCAGGGCATGGATGCAACAGTGTCTCTGCTGGACTTGGCATGGCTGTTGCACGGGATCTTAAGGGGA

AGAAGGACTGCATTGTTACTGTGATAAGTAATGGGACAACTATGGCTGGCCAGGTCTATGAGGCCATGAGCAATGCTGGG

TATTTAGATTCCAACATGGTTGTGATCCTGAATGATAGCAGACATTCTTTACAGCCAAAGCCAGAGGAAGGATCAAAGAT

GGCGATCAATGCTCTATCGAGCACTATAAGCAAGATCCAGTCGAGTAAATCCTTCCGGAAGTTTCGGGAAGCTGCCAAGG

GTGTTACTAAGAGCATTGGTAAGGGGATGTATGAATTGGCAGCTAAAGTAGATGAGTTTGCACGTGGTATGATTGGTCCA

TTGGGGTCGACTCTCTTTGAGGAGCTTGGCTTATATTACATTGGCCCAGTTGATGGCCACAACATCACTGACTTGATTTG

CGTACTCAAAGAAGTTGCATCTTTGGATTCAACTGGACCTGTATTAGTTCATGTTATCACAGAGGAAGGCAAGGGGTGGG

AAGATGACCATAAAAACGAGATGGTAGTCAAATCCCAAGCTGGGATCTCTAGCTCTAGCTCCTTAACTTCATACCCAAAA

ATTATGAGCAGCAGCCTCTCACGGACTTACAGTGATTGCTTTGTCGAGGCTTTAGTAGCAGAGGCTGAGAAGGATAAGGA

TATTGTCGCGGTTCATGCAGATATGGGAATGGATCCATCACTCCAACTTTTTCAGGGAAAATTCCCGGACAGGTTTTTTG

ATGTGGGAATGGCAGAGCAGCATGCTGTAACATTTTCTGCGGGTTTGTCTTGTGGGGGACTGAAGCCATTCTGTATAATT

CCATCCACATTCTTGCAGAGAGCATATGATCAGGTTGTGCACGATGTGGATCTGCAAAATATACCAGTCCGGTTCGTAAT

CACTAGTGCTGGACTGGTTGGGTCTGATGGTCCTATGCACTGTGGAGCATTCGACATAGCCTTCACTTCATGCTTGCCAA

ACATGATTGTCATGGCACCGTCAGATGAGGATGAGGTCGTTCACATGGTTGCCACTGCCGCTTGCATTGATGACAGGCCT

GTTTGCTTCCGCTATCCTAGAGGTGCCATTGTTGGGATTAACATCCCTTTATACAATGGGATACCCCTTGAGATTGGAAA

GGGGAGAATTCTTGCAGAGGGGAAAGATGTGGCTTTACTTGGGTATGGGGTGATGGTTCAGAACTGCCTGAAGGCTCGCT

CCCTTCTTGCATACCTTGGCATTCAGGTGACTGTTGCTGATGCAAGGTTCTGCAAGCCACTTGATATAGAGCTTGTCAGG

CAGCTATGCAGACACCATGAGTTCCTGATTACTGTTGAGGAAGGCACTGTAGGAGGCTTTGGGTCGCATGTTGCACAGTT

CATTTCTCTTGATGGACAGCTTGATGGAAGAATTAAGTGGCGACCAATTGTTTTACCAGACAAGTATATTGAGCATGCAT

CACCCAAGGAGCAGATGGTTCTTGCAGGCCTGACTGGACATCACATTGCAGCAACGGCGTTGAGTCTATTAGGGCGCACC

AGGGATGCCCTTTTATTGATGCGCTGA

>Cbur09G005220.1 gene=Cbur09G005220

ATGCAGTCCTCTGTGGCCTCCACCGTTTTGCCCCGACCCAAGTGTCCATCCAGTATGAAAGAAGATGAAAACCATGAAAC

AGCTGAAAACAGTGTCCTCCAAGTTGCCACATTCTATTGCCAAAGCAGCACCCAAGATCAATTATCGACATCTTCTAGCC

TTAGCTTGGATGGGTTCAGCATCAAGCATGTGGAGAAAGTAAATCAAGTGATGGCCATTGTTCAAAATCTGAAAGAACCA

TCTGAGCGATTGGTTATGATTGATGATCTTCGACGGTTAGGGATCGACTACCACTTCCAGGAAGGGATTGAATCAATTCT

ATGCGGACTTTGTGAAAATTATGGTGCACTTAGCAGTTTATCTGATGTTGCACTGAGCTTTCGTCTGTTAAGAGAACATG

GTCACTATGTATCTCAAGATGTGTTTAAAAGGTTCATGGACGAAGAGGGGAGATTTAAACTTCAACTAAGCACTGATATA

AAGGGAATGATGAACTTGTATGAAGCTTCAAAACTCGCAATTGAAGGAGAGGACATCATCGAGGAAGCTAATGACTTCGC

TACCAAGAATCTCACCGCTTCTGTAAAATTTTTGGAACCATGTCTGGCAAGAGTAGCGAGGCATTCTTTGGAGAATCCTT

TTCACATGAGCTTGCCAAGGTTCAACACTAAGAACCATCTCAACAATCTACGGGAAACTGATAGGAATACGGAAGCCATA

CAGGAATTGGCGAAATTGGAGTTCAACATTGTCCAATCCATGCACCAAAGTGAGCTCAAAGAGGTCACACAGTGGTGGAG

AGACTTGGGTTTGTCACAAGAACTAAGGTTTGCAAGAGATCAACCTTTGAAATGGTACATGTGGTCCTTAGCAGTTCTAC

CAAATCCTAAGTTCTCCAAATATAGGATTGAGCTTACTAAACCCATCGCCCTTGTGTACATAATTGATGACATCTATGAT

GTTTATGGGATGCCTGATGAACTCGTCCTATTTACTGAAGCCATCAACAGATGGGACCCTTCTGACATCAGTCAACTTCC

AAGATACATGAACATATGCTTCATGGCCCTCCATAGTGTCACCAATGAGTTGGCTTGTATGGTACTTAAGGAACATGGAT

GGAATCCCATAAACTCCTTAAGGCAACAGTGGAGAGACCTGTGCAATGCATTTCTTGTAGAAGCAAAATGGTTTGCAGAG

GGAGAATTACCAAAGGCAGATGAGTACTTGAGAAATGGAGTGACTAGTTCAGGAGTGGTAGCCGTGTTGGTACACTTGTT

TTACCTTGTGGGCCATGGGATCACAAGAGAGAGCGTGGATCTTGTGGACAGTGTTCCAAAGCTCATATCTTGCCCAGCAA

TGATTCTGCGGTTGTGGGATGATTTGGGAAGTGCTAAGGATGAGAACCAGGATGGATACGATGGATCATATCTAGAATGC

TATATGAAAGAAAATGCAGCTTCTCTCGAAAGCACACAAAGACATGTAAGACACTTGATATCCAATGCATGGAAGGAACT

CAACAAGGAATCCCTCTCGCCATATCCCTTCTCCCAAACATTTGCACAGGCTTCACTAAATACAGCAAGGATGGTTCAAG

TAATGTATAGTTACAACCAAGATCAACGTCTTCCGATGCTAGAGCAGCACATCATTTCATTGCTTAAAGAAAGGATCCCT

TTGGATGAAATCCCAAAGAAAGCTAACAGATAG

>Cbur09G005210.1 gene=Cbur09G005210

ATGTTGTCCTCAGCGGCTTCTTCTTCATTGGCCCCTCTGAATTCTGATCCTTTCATTCCAAAGAAGAATCACAACCACGA

AACACCTAATGCTCCATTCGATCCAAGCAATGATCAAAACCATGAAACACCAAATTATTCTCTATTTAATACCATTCCCC

AATTAGCTACAGACCATTTCCAAAGCATCACCCATCACCAATTCTGTACATCTTCTACCTTGAGCTTGGATGGCTTAAGC

ATAAAGCAGAGGGAGAAAGTTAATCAAGTGAGGGAAATAGTCCAAAATCTGAAAGACCCAGCTGAGCAAATGGTTATGAT

CGACAACCTTCAACGGCTAGGCATTGACTACCATTTCCGGGAAGAGATTGAATCAATTCTATGTAGCCTAAGTGAAAATG

ATGATGCCATTAGCAGCATTCATGATGTCGCCCTTCGCTTTCGACTGCTAAGAGAACATGGTTACTATGCATCTCCAGAT

GTGTTTAACAGCTTCAAGGACAAAGAGGGTAGATTTAAACTACAATTAACTACAGATATAAAGGGATTAATGAGTTTGTA

TGAATCTTCAAAACTCTCAACTGAAGGAGAGGACATCCTTGATGAAGTTAATGACTTTGCTAGCAAGAACCTCATTGCTT

CAATGGAATTCATTGAACCAGATCTAGAAAGAGAGGTGAGACATGTCTTGGAGCATCCTTTTCACATGAGCCTGCCAAGG

TTCAACATCAAGAAACACCTAAAAGATCTACAAGGAAAGGATGTAAAAACTGATGCCCCCATACAGGAATTGGCAATATT

GGACTTCAATATTCTTCAATCCATGCACCAAAGTGAGCTCAAAGAAGTGACAAAGTGGTGGAGAGACTTGGGTCTGTCAC

AAGAACTAAGATTTGCAAGAGACCAACCATTAAAATGGTACATGTGGCCCCTAGCTGTTCTACCAAATCCTAAATTCTCG

AGATATAGGATTGAGCTTACTAAACCCATTGCTCTTGTCTACATAATTGATGACATCTATGATGTTTATGGGACACTTGA

TGAACTCGTCGTATTTACTGAAGCTGTCAATAGATGGGACCCTTCCGACATCAATCAACTTCCAAGAAACATGAAGCTAT

GTTTCATGGCTCTCCATAACATCACCAATGAGATTGCATACATGGTCCTTAAGGAACATGGATGGAATCCGATAAACTCC

CTTAAGAAAACGTGGACAGACCTGTGCAATGCATTTCTTGTAGAAGCAAAATGGTTTGCTGAGGGAGATTCACCAAAAGC

AGATGAATACTTGAGAAATGGAGTAACTAGTTCAGGAGTGCCAACTGTGTTGGTACACTTGTTTTTCCTTGTGGGCAATG

GGATCACAAGAGAGAGCGTGGATCTTGTGGACAGTATACCAAAGCTCATATCTTGCCCAGCAATGATTCTTCGGTTGTGG

GATGATTTGGGAAGTGCTAAGGATGAGAACCAAAAAGGTTACGACGGATCATATGTAGAGTGCTACCTGAAAGAAAATGC

AACTTCTTCTCTCGAAAGTGCCAGAAGACATGTAAGACACATGATTTCCAATGCATGGAAGGAACTCAACAAGGAATGCC

TCTCACCATATCCCTTCTCTCCAACTTTCATAGAGGCATCACTAAACACAACTCGAATGGTTCAAGTCATGTATAGCTAC

GACAATGATCAACGTCTTCCAGCTCTTGAGCAGCATATCACCTCCTTGCTTAAAGAAAGTATCCCTGATAAAGAAATCTG

A

>Cbur09G005020.1 gene=Cbur09G005020

ATGGCTCTTGTTCCTAGTTCTGTCCACTCAGATGTTCCCACGACTAACCCAGTTCGAAAATCTGCCAATTATCACCCTAA

CATTTGGGGCGACCTCTTTTTGCAATCCTCTCCACATGACTATATGCTTGATGAAGCGATCAAAAGAAGGGCTAAAGGAC

TGAAGGACCAAGTAAGGAGGATGCTCCAAAATGCTAGTGATCCATTGGTAGAAATGAATTTGATTGACTCTATCCAACGG

CTTGGAGTGGCCTATCACTTTGAAACCGAGATCGAAGAAAAGTTAGAAAGGTGGTACAACGCATGGGTTGATGGGCTAAA

TGATGGCGAAGATTTGCATGCTGTTGCTCTCCAATTTCGACTCCTGAGACAACATGGTTACAACGTCTCACCTGATGTAT

TTTGCAAGTTTAAGGAAAACGCGGGGAAATTCAAAGAAAATTTAGCTCATGACATTCGAAGTCTCTTAAGTTTGTATGAA

GCATCCTCTCTGGGGACATATGAAGAAGACATCTTGGATGAAGCCAATAAATTTGCTAGAGATCATCTTCAGTTAGCAGT

TCATCATCTGAGCTCACCTCTTTCCACTCTAGTTAAGCTCGCTCTAGAGTTGCCTCTGCAGAAACGAGTTCAAAGGCTAC

AAGCAAGGTGCTATATTTCAATTTATGAAGAGGAGAGAAATGATGCCCTGCTGGAATTTGCAAAGTTAGATTTCAACATG

CTTCAGTCATTGCACAAAAGAGAACTAGGAAACATCTCACTGTGGTGGAAGAAGATTGATTTCACCAGAAAGCTTCCTTT

CATAAGAGATAGACTCGTCGAGTGTTACTTTTGGATCTTAGAAGTTTATTATCAACCACAGTATTCTCGAGGAAGAATGA

TGGCGACAAAGATAATATACCTGACATCAGTGATGGATGACATTTATGATGTGTATGCTACCCCAGAGGAACTTGAGCCT

TTCACTGATGCAATCCAAAGGTGGGATCGTGGGGCAGCAGATCAATTACCAGACTACATGAAAGTGCATTTTCTTGAAGT

AATAGATTGTGTTGATGAATTTGAGAAGGAATTAGCAGAGGAAGGACAATCATATCGTATATACTACTTAAAAGAAGCTT

TCAAAGAAGTCTCGAAAGCCTACAACAAAGAAGCCCAGTGGTTCCACTCAGGGTATTTGCCATCATATTCTGAGTACATG

AGACTTGCCTTGGTCACTAGTGGCTACCCTCTGGTGTCAGTAGTTTCAATGGTTGGCATGGGAGAAATAGTGACGAAGGA

AGCCTTGGAATGGGCCATGAGCACACCTCAGCTCATCAAGGCCTGTTCCGCCATTGCCCGAGTCAAGGATGACATCCAAT

CAAACAAGTCTGAGCAAGAGAGAGGACATGTTGCATCAAGTGTTCAGCTATACATGAAGGAGAATGAATGTACATATGAT

GAGGCATGTGTGAAGTTACAAGGGACGGTTGAAAGAGCATGGAAAGACATAAACAAAGAATGCCTGAAGCCTACACCAGT

TCCTATGCCTTTCCTCGTACGCGTCGTGAACCTCGCACGTGTGATTGAAGTCTTATACCAGAACAGAGATGGTTATACTG

ATTCAACACATGAGACCAAAGAAAGGATCTTATCCGTGCTTGTTAATCCTATCCCAATTTGA

>Cbur12G006720.1 gene=Cbur12G006720

ATGGCTGCGGCGCCAAATGGAAAGGCAGAAGATCGGCGTTCGGCGTTTCTGGGGGTGTACGATCGCCTAAAGGCCGACCT

CCTCCAAGACCCAGCTTTCGATTTCACCGAAGATTCCAAGAACTGGGTCGATCGAATGCTGGATTATAATGTACCAGGAG

GGAAGCTGAACCGCGGGTTATCTGTGATTGATAGCTACCAATTGCTTAATGGTGGAAAGGAACTGACAGCAGAAGAATAC

TTTGATGGATCTGTTCTTGGTTGGTGCATCGAATGGCTTCAAGCTTATTTCCTGGTCCTTGATGATATTATGGATGAATC

TTATACAAGGCGTGGCCAGCCTTGTTGGTTCAGAAGACCAAAGGTTGGTATGATTGCTGTGAATGACGGTATTGTACTTC

GTAACCATATTCCTAGAATGCTCAATAAACAATTCAAGGGAAGGCCATACTATGCTGATCTCCTTGATTTGTTCAATGAG

GTTGAGTTCCAGACAACTCAAGGACAAATGCTTGATCTGATCACCACTCTTGATAAAGAAAATAATCTGAACAAATACAG

CTTGCCAGTTTATCGGCGCATTGTAACGTACAAAACTGCCTTCTATTCATTTTATCTTCCAGTCGCATGTGCTTTGCTTC

TGATGGGCGAGAGCTTGGACAAACACCTTGATGTGAAGAACATTCTCATTGAAATGGGAGTTTACTTTCAAGTTCAGGAT

GATTATTTGGATTGCTATGGTGATCCTAAAGTAATCGGCAAGATCGGAACTGATATTGAAGATTACAAGTGCTCTTGGTT

GGTTGTGAAAGCTCTTGAAAGGGCCAACGAGCACCAAAAGAAGATACTATATGAGAACTATGGAAAAACAGATTCAGAGA

AGGTAGCCAAAGTGAAGGCTCTTTACAATGAACTTGGTCTCCAGAATGTATTTTTGGAGTATGAGCGAGAGAGTTATGCA

CAGCTCATCTTGTCCATTGAAGCCCAACCTAGTAAAGCAATGCAAGAGGTGCTGAAGTCCTTCTTGGGCAAGATCTACAA

GAGGCAAAAGTAG

>Cbur10G020080.1 gene=Cbur10G020080

ATGGCAGCTGCTATTAGTGTTACTGCTTCTTCTTCTGATGCTCCAAACGGGAAAGCAACGGACTTGAGATCTACATTTGA

GCAGGTCTACAATCGCCTCAAATCCGAACTCCTCGAAGATCAAGCCTTCGATTTCACCGATGATTCTCGCCAATGGATAG

AACGAATGCTGGACCACAATGTGCCTGGAGGGAAGCTGAACCGAGGGCTATCTGTAGTTGATAGCTACAGGTTGTTAAGA

CAAGGAATGGGAATAAGCATAGATGAAGTCTTCCTTGGATGTGTGCTTGGTTGGTGCATCGAATGGCTTCAAGCATATTT

TCTAGTCCTCGATGATATTATGGACGACTCCCTCACACGACGGGGTCAACCCTGCTGGTTCAGAGTGCCTAAGGTTGGTT

TCATTGCTGTTAATGATGGTGTCCTACTCCGTAACCATATTCCTAGAATTCTTAAACGGCACTTCAGGGAAAAGCCTTAT

TATGTGGATCTTCTGGATTTATTCAATGAGGTTGAGTTTCAGACAGCTTCAGGACAGATGCTAGACTTAATTACTACACA

TGAAGGAAAAAAAGATCTTTCCAAGTACAAAATGCCAGTTTATCGTCGCATTGTTCAGTACAAAACAGCTTATTATTCAT

TTTACCTTCCGGTTGCTTGTGCATTGGTTATGTCAGGTGAGAATTTGGAAAACTTCATCAATGTCAAAAACATCCTGATC

GATATGGGAACCTACTTCCAAGTGCAGGATGATTATCTGGACTGCTTTGGCGATCCTGAGGTGATTGGTAAGATAGGAAC

AGATATAGAAGATTTTAAGTGCTCGTGGTTGGTTGTGCAAGCCCTTGAACGTGCTGATGAAAATCAAAAGAAAATATTGT

CTGAGAACTATGGGAAACATGTTGCTAGAGTTAAAACACTTTATAAAGATCTTGAACTTCAGGCTGTATTTCTTGAGTAT

GAAAGGGAAAGCTATGAGCGGTTGACTGCCTCCATTGATGCCCTGCCAAGCAAATCAGTGCAGGAGGTGTTGAAGTCCTT

CTTGGGCAAGATTTACAAGAGGCAGAAGTAA

>Cbur11G002320.1 gene=Cbur11G002320

ATGGCGGTCTCGAATGGATCTGAGCCATGGATGTTGATGGTAACGGCTAGATCTCCGACGAACATTGCCGTGATCAAGTA

CTGGGGAAAGCGCGACGAAACCCTCATCCTCCCGATCAATGACAGCATCAGCGTGACGCTGGATCCAGACCATCTGTGCA

CGACCACCACCGTCGCGGTTAGCCCGGCGTTTGATTCTGATCGCATGTGGCTCAATGGAAAAGAGATTTCTCTCAGCGGA

GGGCGGTACCAGAGCTGTTTGAGGGAGATCAGGAGGAGGGCATGCGATGTAGAAGACGAGAAGAAGGGGATTCGGATTAG

GAAAGAGGATTGGGAGAGGCTACGGGTGCATATAGCTTCTTACAATAACTTCCCTACTGCGGCCGGATTGGCTTCCTCTG

CTGCAGGATTTGCTTGCCTGGCATTCGCGCTTGCAAAACTGATGAATATTAAAGAAGAAAATGGAGAACTTTCATCCATT

GCAAGGCAAGGTTCAGGCAGTGCTTGTCGCAGTCTATATGGCGGATTTGTAAAATGGGTTATGGGAAATGATGCTTCTGG

ACATGACAGCATTGCAGTTCAACTTGCAAATGAGTCACACTGGGATGATCTTGTTATTATTATTGCAGTAGTAAGTTCAC

GACAGAAAGAAACAAGTAGCACTGCAGGAATGCGCGAGAGTGTTGACACAAGTACTCTATTAATTTATAGATCCAAGGTG

GTGGTTCCACAGCGCATATTGCAAATGGAAGAAGCCATTAGAAATCGGGATTTTCAGTCTTTTGCAAGGTTGACTTGTGC

AGACAGCAATCAGTTTCATGCTGTTTGCTTGGATACCTCTCCTCCCATATTCTACATGAATGATACTTCCCGCAGGATAA

TAAATTGTGTGGAAAGATGGAATCAGTTCGAAGGAACACCACAGGTGGCTTACACTTATGATGCGGGGCCCAATGCAGTT

CTTATTGCGCGCAACAGAACAGCTGCTAGTCTTCTGCTACAGAGGCTGCTCTTCTACTTTCCTCCTCCCCCAGACACTGA

TCTAACTAGCTATTTATTAGGTGATAAATCAATATTGGAAGAATCTGGTTTGCAGACAATGAAAGACGTGGAAAACTTGC

AGGCACCTCCAGAAATAAAGGGCAGCATTTCTGTTGATAAAAATTCTGGCAGTGTCAGCTATTTCATCTGCACGAGACTT

GGGAGAGGTCCAACATTGCTTGTTGATGAAGGCCAAGCCCTCATTGATCCCAAAACTGGGATTCCTAAGTAA

>Cbur06G009090.1 gene=Cbur06G009090

ATGGCACAAGTAGTTGCGTCTGCTCCGGGGAAGGTTTTGATGACAGGTGGGTACTTGATTTTGGAGAGACCTAATGCTGG

AATTGTTCTCAGCACGACTGCCCGTTTCTATGCAATCGTGAAGCCGTTTTATGAAGAAGTTAAGCAAGATAGCTGGGCCT

GGGCATGGACAGATGTGAAAGTAACATCTCCTCAGCTTTCTAGAGAAACCATGTACAAGTTGTCTCATAAGAACTCAACC

CTCCACTGTATCTCTTCAAGGGACTCTGCCAACCCTTTTGTTGAACAAGCAGTACAATATGCAGTTGCAATTGCCACCAC

AATCTTAAGTGATAGGGGAAAGAAGGATGAGTTACAGAAACTACTCTTGCAAGGCCTTGATATCACAATATTGGGTTGCA

ATGACTTTTACTCATATCGAAATCAGATTGAAGCACGTGGACTTCCTTTGAGGCCAGAAGCATTGGCCTCACTTCCATCC

TTCTCATCAATTACCTTTAATGCAGAGGGGTCTGATGGGACTGTTACTAGAGATAATTGCAAACCTGAAGTTGCAAAAAC

TGGACTAGGTTCTTCAGCAGCAATGACCACGTCAGTGGTTGCAGCATTACTTCATTACCTTGGTGTTGTTCGTCTGCCAT

CTTCAGCTAAAAGTCCTATTGAAGAGAAGCTGCTCAATTCAGACCTCGACTTGGTGCATATTGCAGCCCAAACTGCCCAT

TGTATTGCACAAGGGAAAGTAGGCAGTGGTTTTGATGTTAGTGCTGCAGTCTATGGTAGTCAACGCTATGTTCGATTTTC

TCCTGGAGTTCTTTCTTCTGCTCAGGTGGCAGTGAAAGGAAAGCCATTACAAGAGATCATTGCTGGTATTCTGAAAGAGA

AATGGGATCATGAGAAGATTAAGTTTTCGTTACCACCACTGATGACTCTTCTACTAGGGGAACCTGGAACTGGAGGATCA

TCCACACCATCTATGGTGGGTGCTGTGAAGCGGTGGCAAACATCTGATCCTCAGAAAGCCTTGGAATCATGGAGAAAGTT

AGCAGAGGCAAATTCGGCTCTTGAAATGCAACTCAAAATGTTAAGTAAATTTGCGGAGGAGCAGTGGGACATGTACCGGT

CTATTATTAGAAACTGTAGCGAGTATACATATGATAAGTGGATGCAGCAAGCAGCTAATACATACCAGGAAGCAATTGTG

AAATCACTGTTAGGGACAAGAGATGCTTTCCTTCACATCAGATTTCATATGCAACAGATGGGCCAGGCAGCAGGTGTACC

AATAGAACCTGAATCACAAACTCGACTTTTGGATACCACAATGAATATGAAAGGAGTTCTGTTGGCTGGAGTTCCTGGAG

CAGGTGGCTTTGATGCAATTTTTGCTATTACCTTAGGAGAATCCGGCAATAACATAGCCGATGCCTGGAGTAAACTGAGC

GTTTTGCCCATGTTGGTAAGAGAAGATCCTCAAGGCGTTTCTTTAGAAAGTGGTGATCCACGAGTGAAGGATATCTCATC

TGCCATTTCTTCAATTCATATTTGA

>Cbur09G007390.1 gene=Cbur09G007390

ATGGAGATTCGAGCACGGGCGCCCGGAAAGATCATACTTTCCGGCGAGCACGCCGTCGTTCATGGAGCCACGGCGGTGGC

CGCGTCGATAGATCTCTACACGCATGTTTTGATCCGATCATCAACTTCCTCAGGAACCAATGATGGGCTTTTAGAATTGC

AGCTCAAAGATATGGGGTTAGTGTTTTCGTGGCCAGTTCAAAGAATCAAAGAAACACTTCCAGAATTGGGTACTGCTCTT

TCTCCATCATCAACATCTTTCTTGCCAGAGTCCATGAAATCCATTGCTACTCTTGTTGAGGAGCAGAATATTCCTGAGGC

TAAAATTGGACTCTTTGGCGGAGTTTCTGCCTTCCTCTTGCTGTACACCTCCATCCAAGGGTTTAAGCCTGCAACAGTAG

CAATCAGCTCTGATCTTCCTATGGGTTCGGGTTTGGGTTCATCTGCTGCATTCTGTGTTGCGCTTTCAGCAGCTTTACTT

GCATATTCAGGTGCAGTTAGATTGGACGCCAATAACTATGGTTGGTTAACTCTAGCGGAGCTTGAGCTTGAATTGGTTAA

CAAATGGGCCTTTGTAGGTGAAAAAATCATTCATGGCAAGCCTTCTGGAATTGATAATACTGTGAGCACTTTTGGCCACA

TGATCACGTTCAGGTTGGGTGAATTAACTCGCATTAGATCTAATATGCCACTGAGAATGCTAATCACCAACACTAAGGTT

GGAAGGAACACGAAGGCATTAGTTGCTGGTGTTTCTGAGCGAGCATCCAGGCATCCTGATGCTATGGCTGCTGTGTTTTC

TGCTGTGGATTCCATCAGCAGGGAACTTTCTACCATTATCCAGTCGCCTGCTTCAGATGAACTCTCCATTACAAAGAAAG

AAGATAAGTTAGAGGAGCTTTTGGAAATGAATCAAGGTTTGCTCCAGTCCATGGGAGTTAGCCACTCTTCTATAGAGGCT

GTGATTCGAACAACTCTGAAGTACAAGTTAGCTTCAAAGCTGACAGGAGCTGGTGGTGGAGGCTGTGTTTTGACATTGTT

GCCAGCATTGCTTGCTGATGTGGTTGTCGATAAAGTGATAGCTGAGCTTGAGTCATGTGGATTTCAGTGCTTGATAACTG

CAATTGGAGGTAAAGGTCTTGAAATTTCTTCCTCTTGCAGCTCCTCAACTTGTCATCCATCTGGAGAAATTTAA

>Cbur05G000450.1 gene=Cbur05G000450

ATGGCACATTCCAAACCAGATCCCGAATCTCCGCCCAACATCCGCCGGAGATACTCCTTTTCGGACGGAGACTCCCCCGC

CCCGCCCCCTCCGGCTCATACGTCCGATGCCCTCCTCCTCTTCCTCCGCCTCGCCAACAAGCTCTTCCTCCCTCTATTCT

TCGTCGCCGCCTACTTCCTCATGCAGCGGTGGCGCGAGAAGGTCCGCACCTCCACCCCTCTCCACCTCCTAACCTTCTCC

GAGATCACAGCCATCGTCTCCCTCATCGCCTCCTTCATCTACCTCCTCGGCTTCTTCGGCATCGACTTCGTACAGCCCCA

CATCTCCCGATCCCCCGATGACGATTCCGACGTCGTCCAACACTTCATCCGCCCCACACCCACTCCCAAGCGACCCATCT

CCACCGGCACCACAGCTCCGTTCGTCTCTCCCGACAATGCCGACATCGTCTCCCAGGTCGTTACTGGATCGATACCCTCG

TATTCTCTCGAATCCAGGCTGGGGGACTGCAAGCGGGCGGTGGTGGTGAGGCGTGAGGCGCTGCAGGTGAAGATGGGGCG

GTCGCTCGACGGGCTCCCGGTGGATGGGTTTGATTATGATTCGATTCTGGGGCAGTGCTGTGAGATGCCGGTGGGGTACG

TGCAGGTTCCTGTGGGGGTCGCGGGGCCGCTGTTGCTGGACGGGAGGGAGTTTTCGGTGCCGATGGCGACGACGGAGGGA

TGCCTGGTGGCGAGCACGAACAGGGGTTGCAAGGCGATCTACAGGTCGGGAGGTGCGACGAGCGTGCTTTTGAGGGATGG

GATGACGAGGGCGCCCGTGGTGAGGCTTGCGAGCGCCAAGAGAGCGGCGGAGCTCAAGTTCTTTTTGGAAGATCCAGTTA

ATTTTGAGACTTTGGCGATGATCTTCAACAGGTCTAGCAGATTTGCAAGGCTTCAAGGAATTCAGTGCTCAATGGCAGGG

AAGAATCTTTACATGAGATTCACATGTAGCACGGGAGATGCAATGGGGATGAACATGGTATCCAAAGGTGTACAGAATGT

CTTGGATTTCCTCCTGCATAATGACTTTCCAGACATGGATGTAGTCAGTTTATCAGGCAATTTTTGTTCAGACAAGAAGC

CCGCTGCAGTTAACTGGATTCAAGGACGTGGCAAATCTGTCGTTTGTGAGGCAGTTATCAAGGAGGAGGTGGTCAGGACA

GTCCTCAAGACCACAGTGCCTGCCCTGGTGGAGCTGAACATGCTTAAGAACCTTGCTGGGTCAGCTGTTGCTGGAACCCT

GGGGGGTTTCAATGCTCATGCCAGCAATATTGTCTCGGCCGTCTTTATTGCCACTGGCCAAGATCCTGCACAGACTGTTG

AAAGTTCTCACTGCATTACCATGATGGAAGCTGTTAATGATGGAAAAGATCTTCATATCTCTGTTACCATGCCTTCTATT

GAGGTGGGTACAGTTGGTGGGGGAACCCAGCTTGCTTCTCAGTCAGCTTGTTTGAATCTACTTGGTGTGAAGGGTGCAAG

CATGGAATTACCCGGAGCAAATTCCAGGCTCTTGGCCACCATCGTTGCAGGCTGTGTCTTAGCAGGAGAGCTCTCTCTCA

TGTCTGCACTTGCAGCAGGGCAGCTGGTCCAGAGCCATATGAAATACAACCGACGGAGTAAAGACTGTGCAAAGCTTGCA

GCATCCTAA

>Cbur02G029110.1 gene=Cbur02G029110

ATGGCTACTTTCTACAAGAAACCTGCCGAGTCTCCGGCCAAGATCCGCCGGAGATTTTCCCTCCCCTCCGGGGATTCCGC

CCGCGGGCCGTCGTCGGCCGCCCAGGCCTCGGACGCGCTCCTCCTCCCTATCCGCCTCACGAACAAAGTCTTCTTCCCCC

TCTTCTTCTTCGCTGCCTACTTCCTCCTCCGCCGGTGGCGCGAGAAGATCCGCACATCCACCCCCCTCCACCTCCTTACT

TTCTCTGAGATCGCCGCCATCGTCTCCCTCTTCGCCTCCTTCATCTACCTCCTCGGCTTCTTCGGCATCGACTTCGTCCA

GTCCTTCATCTCCCGTCCCTCCGACGAAGACCCAGACGACGTTGTCGTCGCCGCCCCCGCCCCCCGCTTCCTCCCTCCCA

AAACCCCCCTCCCTGCTGCCGCTGCTGACTCCCCCGACGCCGACATTGTCGCCTCCGTCCTCTCCGGCGCCATCCCCTCC

TACTCCCTGGAGTCCAAGCTCGGCGACTGCCGGCGAGCGGCCTCCATCCGCCGCCAGGCGCTGCAGGCGAAGATCGGGTG

CTCGCTGGAGGGGCTCCCATTGGAGGGTTTCGACTACGATTCGATTCTGGGGCAGTGCTGCGAGATGCCCATTGGGTACG

TGCAGCTGCCGGTGGGGATCGTCGGGCCGCTGCTGCTCGACGGGAGGGAGTTCTCGGTGCCTATGGCGACAACGGAGGGT

TGCCTTGTGGCGAGCACGAACAGGGGGTGCAAGGCGATTGCGATGTCGGGTGGAGCGACGAGCGTTCTGCTGAGGGACGG

GATGACGCGGGCACCGGCGGTGAGGTTTGCGACGGCGAAGAGGGCGGCGGAGTTGAAGTTCTTCTTGGAGGATCCTATTA

ATTTCGATACATTGGCTGTGATCTTCAATAGATCAAGCAGATTTGCCAGGCTTCAAGGGATTCAATGTGCAATGGCTGGG

AAGAATCTCTACGTAAGATTCACCTGCAGCACTGGAGATGCAATGGGGATGAATATGGTATCCAAAGGTGTTCAAAATGT

ATTGGATTTCCTGCGGAATGATTTCCCTGACATGGATATAATCAGCATCTCAGGAAATTTCTGTTCCGACAAGAAGCCGG

CCGCAGTGAATTGGATTGAAGGGCGTGGCAAGTCTGTGGTTTGTGAGGCAGTCATCAAGGAGGAGGTAGTAAGGAGTGTC

CTGAAGACCAGTGTATCTGCACTGGTGGAACTTAACATGCTTAAGAACCTTGCTGGATCGGCCGTGGCTGGTGCCTTGGG

AGGTTTCAATGCTCATGCTAGCAATATTGTCACTGCGATCTTCATTGCCACCGGCCAAGATCCTGCACAGAACGTTGAAA

GTTCCCACTGCATCACCATGATGGAACCTGTTAATGGTGGAAAGGATATCCATGTCTCTGTTACAATGCCTTCCATTGAG

GTGGGGACAGTTGGGGGTGGGACCCAACTTGCTTCTCAGTCAGCCTGCCTGAATCTACTTGGTGTGAAGGGTGCAAGTCT

AGAATCACCTGGAGCAAATTCCAGGCTCTTGGCCACCATAGTTGCAGGATCTGTTCTAGCAGGAGAGCTCTCCCTCATGT

CCGCTCTTGCTGCAGGGCAGCTTGTCAAGAGCCACATGAAATACAACAGATCAAGCAAAGATATATGCAAAGTTGTCTCC

TGA

>Cbur07G003780.1 gene=Cbur07G003780

ATGGAGTCGCAGCAGAAGAACGTGGGGATTCTGGCCATGGACATATACTTCCCTCCTACTTGCGTTGATCAGGAAGCTCT

GGAAGCCCATGATGGAGCAAGTAAAGGGAAATACACTATTGGACTTGGGCAGGATTGCATGGCCTTCTGCACAGAGTTGG

AAGATGTTATTTCAATGAGCTTGACAGTTGTTAAATCTCTTCTCAAGAAATATGAGATCAATCCAAGACTTATTGGCCGT

CTGGAAGTAGGGAGCGAGACAGTTATAGACAAAAGCAAATCCATTAAGACTTGGCTAATGCAAATCTTTGAGGAGCATGG

GAATACTGACATTGAAGGAGTTGACTCAACAAATGCATGCTATGGCGGAACTGCAGCATTATTCAATTGTGTGAATTGGG

TGGAGAGTAGCTCTTGGGATGGACGCTATGGCCTTGTTGTATGCACTGACAGTGCGGTATATGCAGAAGGACCAGCCCGT

CCAACTGGAGGTGCAGCTGCCATTGCAATGCTTATTGGACCAAATGCTCCTATAGCATTCGAAAGCAAGTTTAGAGGGAC

TCACATGGCTCATGTTTATGATTTCTACAAACCCAATCTTGCTAGTGAATACCCGGTTGTTGATGGGAAGCTTACACAAA

CTTGCTATCTCATGGCACTTGATTCTTGTTACAGACGCTTTTGTAGTAAGTTTGAGAAATTGGAGGGAAAACAATTTTCA

ATTTCTGATGCAGATTATATTGCTTTTCATTCTCCATACAACAAGCTTGTTCAGAAAAGCTTTGCTCGGATGTACTTCAA

TGACTTCTTGAGGAATGCCAGTTCTGTTGAGAATGATGCAAAAGAAAAATTGGAGCCATTTTCATCCTTGTCCGGTGATG

AAAGCTACCAGAGTCGTGATCTTGAAAAGGTATCGCAGCTAGTTGCTAAGAGTCTTTATGGTGCAAAGGTACAACCTTCT

ACTCTGATACCGAAACAAGTTGGCAACATGTACACTGCTTCACTTTATGCCGCATTTATATCTATTCTTCACAGCAACGG

TAGCACTTTGGAGGGTAAACGGGTAGTTATGTTCTCATATGGTAGCGGTCTGTCTTCGACAATGTTTTCGTTTAGGTTTC

AGGAGGGTCAACATCCTTTTAGCTTATCAAACATTGCTAGTGTGCTGGATGTTTCTGGAAAGCTGGAGTCAAGACATGTT

TTTCCACCGGAGAAATTTCTTGACACATTGAAACTGATGGAGCACCGTTATGGGGGGAAAAATTTTGTAACTAGCTCAGA

CACGAGCTTACTAGCTCCAGGGACATTTTATCTGACCAAGGTTGATTCCATGTACCGGCGATACTATGCCCAGAAGGTTG

GAGATACTGCTACATTCAGCGCTTCTCTCATCCATGATAACGGTTCACTGCCCAATGGCCACTGA

>Cbur01G026590.1 gene=Cbur01G026590

ATGGGGTCGCAGCAGAAGGATGTCGGGATTCTCGCCATGGATATCTACTTTCCTCGCACATGCGTCGATCAGGAAGCCCT

GGAAGCTCATGATGGTGCAAGTAAAGGCAAATACACTATTGGGCTTGGGCAGGATTGCATGGCTTTCTGTACAGAGTTGG

AAGATGTTATTTCAATGAGCTTGACAGTTGTTACTTCCCTTCTCAAGAAATATGGGGTTGATCCAAGACTTATTGGTCGC

CTTGAAGTAGGAAGCGAGACTGTCATCGACAAGAGCAAATCCATAAAGACTTGGCTGATGCAAATCTTTGAGGAGCATGG

GAATACTGACATTGAAGGAGTTGACTCAACAAATGCATGCTACGGGGGAACTGCAGCATTATTCAACTGTGTGAATTGGG

TGGAGAGTAGCTCTTGGGATGGACGCTATGGTCTTGTTGTCTGCACTGACAGTGCGGTCTATGCAGAAGGACCAGCCCGT

CCAACTGGTGGTGCAGCTGCCATTGCAATGTTGATTGGACCAAATGCTCCTATAGCATTAGAAAGCAAGTTTAGAGGGAC

TCACATGGCTCATGTCTATGACTTTTATAAGCCCAATCTTGCGAGTGAATACCCGGTTGTTGATGGCAAGCTTACACAAA

CTTGCTATCTCATGGCACTTGATTCTTGTTACAGACGTTTTTGTAGCAAGTTCGAGAAATTGGAGGGAAAACAGTTTTCC

ATTTCTGATACGGATTATTTTGCATTCCATTCTCCATACAATAAGCTTGTTCAGAAAAGCTTTGCTCGGCTGTACTTCAA

TGACTTTCTGAGGAATGCCAGCTCTGTTGGGAAGGATGCAATGGAAAAATTAGAGCCATTTTCATCTTTGTCTGGCGATG

AAAGTTATCAAAATCGTGATCTTGAAAAGGTATCTCAGCTGGTTGCAAAAGGCCTTTATGATACAAAAGTAAAACCATCA

ACTTTGTTGCCAAAACAAGTTGGCAACATGTACACTGCATCTCTTTATGCTGCATTTGTATCCATTCTCGATGGCAAGCC

TAGCGCTCTGAGAGGTAAACGGGTAGTTATGTTCTCATATGGCAGTGGTCTATCTTCCACAATGTTCTCATTCAGACTCC

GGGATGGTCAGCATCCCTTCAGCTTATCAAACATTGCTTGTGTGCTGAATGTTTCTGAAAAGCTGGATTCTAGACATGTT

TTTTCACCTGAGAAATTTGTTGAAACAATGAAGCTGATGGAGCATCGTTATGGAGCCAAGGATTTTGTAACCTGCTCAGA

CAAGAGCTTACTAGCTCCAGGTACATTCTATCTTACTAAGGTTGACTCAATGTACAGGCGATATTATGCCCAAAAGGTTG

GAGAAACGGATAAGTGCAGCACTGCTGCCAGCCATGAGAATGGTTCACTGCTCAATGGCCACTGA

>Cbur10G017760.1 gene=Cbur10G017760

ATGGCTTCAATCTCTGTTCCGTGCCCTAAATGCTCGGCATTTGCTTCATCCGGCGTGCGACTGCAGAATCTGCAGCAGCA

TTCGAAGGTCTCATTTTCACCGATTGCGTATCGGAGGCATTCACTGATAGGTGGATCTTCACTAGAACAGCCACGCTTTA

CACGGTTTCAGGGCCCTAAGAGGAATCATTCTACTGTTTGCAAAGCCCAGATTAATGAGGTTGCTGTCGAGAAATCTTTG

AACTCCACCTCAGCCACCCCAACCAATTCTGAGGCACCATCATCTGAAGTTAAAGGTGTAGAACCAACTAAAGAACCTGG

AGTTCCTGATTCATCTGTCGCTGCCTTCATGTCCGAAGTGGCAAGCCTTGTCAAGCTTGTGGATTCAAGAGATATTATGG

AGCTAAAGCTGAAGCAAAGGGATTGTGAGCTCATTATAAGGAAAAAGGAGGCTTTCCCGCAACCAACCGCCGCTCCTATT

GTTATGAGGCAACCCCCTTCTGCACCTATGCTTCCTTCTCAACCAGCACCGGTGCAGACTGCTGCTAGGCCTGCTGCTTC

AGCACCTGCATCGACAGCTCCTCCTGCTCTCCCTGCCCCAGCAAAGTCAAGCAAGTCATCACATCCACCACTGAAATGTC

CCATGGCTGGAACATTTTACCGATGTCCTGCACCTGGGGAGGCACCATTTGTTAAGGTTGGAGACAAGGTACAAAAAGGT

CAGGTCATTTGCGTTATTGAGGCCATGAAACTGATGAATGAGATCGAGGCGGATCAATCTGGAACAATTGTGGAGATACA

TGCTGAGGATGGTAAACCAGTCAGTGTTGACACACCGCTAATGGTTATTGCGCCCTGA

>Cbur05G014680.1 gene=Cbur05G014680

ATGGCTCCAGCTGCAGCTTCAGATTCCATTAAGCCTCGAGATGTATGTGTTGTTGGTGTTGCTCGTACACCTATGGGAGG

GTTTCTTGGCACCCTTTCTTCTTTATCTGCCACCAAGCTAGGTTCTATAGCTATTGAATGTGCACTTAAAAGGGCTAATG

TTGATCCTAAACTCGTACAAGAGGTCTTCTTTGGAAATGTTTTGAGTGCAAATTTGGGGCAGGCTCCTGCTAGGCAGGCT

GCCTTGGGTGCTGGAATACCTAATACAGTTGTCTGCACGACTATTAATAAAGTCTGTGCATCCGGGATGAAGGCAACAAT

ACTTGCGGCACAGACTATCCAGTTGGGAATTAATGATATTGTTGTGGCTGGTGGCATGGAAAGCATGTCCAATGCACCCA

AGTATCTATCTGAGGCAAGAAAAGGTTCTCGATTTGGACACGATACTGTCGTTGATGGAATGCTCAAAGATGGTCTATGG

GATGTTTACAATGATTATGCGATGGGAATGTGCGCTGAGCTCTGTTCAGAACAACATACAATAACAAGAGAAGAACAGGA

TACTTATGCTATACAAAGCTTTGAACGTGGTATTGCTGCTAAAAATGCTGGTGCCTTTGCATGGGAAATAGTACAGGTTG

AAGTTTCTGGGGGGCGAGGAAAACCATCCACACTTGTTGATAAGGATGAAGGCCTAGAAAAGTTTGATCCCGTAAAACTA

AGGAAGCTCCGGCCAAATTTTAAGGAGAATGGAGGTTCTGTTACTGCTGGCAATGCATCTAGTATAAGTGATGGTGCTGC

TGCATTAGTGTTAGTGAGTGGAGAAAAAGCTCTTAAGCTTGGGCTGCAAGTCATTGCGAAGATCACGGGATACGCTGATG

CGGCACAGGCACCTGAGTTATTTACAACTGCTCCAGCCCTTGCAATACCAAAAGCCATTTCAAATGCAGGCTTGGAGGCT

TCTCAAATTGATTACTATGAAATAAATGAAGCTTTCTCTGTTGTGGCTCTTGCAAATCAGAAGCTGCTTGGGCTTCCATC

TGATAAAGTTAATGTGCATGGCGGGGCTGTTTCCTTAGGGCATCCTCTAGGTTGCAGTGGAGCTCGTATATTGGTCACTC

TTCTAGGGGTATTGAGACAGAGACAAGGGAAGTTCGGAATTGCTGGCGTTTGCAACGGGGGAGGAGGAGCATCAGCACTT

GTCCTAGAGCTAATATAA

>Cbur05G014600.1 gene=Cbur05G014600

ATGGCTCCAGCTGCAGCTTCAGATTCCATTAAGCCTCGAGATGTATGTGTTGTTGGTGTTGCTCGTACACCTATGGGAGG

GTTTCTTGGCACCCTTTCTTCTTTATCTGCCACCAAGCTAGGTTCTATAGCTATTGAATGTGCACTTAAAAGGGCTAATG

TTGATCCTAAACTCGTACAAGAGGTCTTCTTTGGAAATGTTTTGAGTGCAAATTTGGGGCAGGCTCCTGCTAGGCAGGCT

GCCTTGGGTGCTGGAATACCTAATACAGTTGTCTGCACGACTATTAATAAAGTCTGTGCATCCGGGATGAAGGCAACAAT

ACTTGCGGCACAGACTATCCAGTTGGGAATTAATGATATTGTTGTGGCTGGTGGCATGGAAAGCATGTCCAATGCACCCA

AGTATCTATCTGAGGCAAGAAAAGGTTCTCGATTTGGACACGATACTGTCGTTGATGGAATGCTCAAAGATGGTCTATGG

GATGTTTACAATGATTATGCGATGGGAATGTGCGCTGAGCTCTGTTCAGAACAACATACAATAACAAGAGAAGAACAGGA

TACTTATGCTATACAAAGCTTTGAACGTGGTATTGCTGCTAAAAATGCTGGTGCCTTTGCATGGGAAATAGTACAGGTTG

AAGTTTCTGGGGGGCGAGGAAAACCATCCACACTTGTTGATAAGGATGAAGTGTTTCACTGTAGGGATATCTGGATATTG

TGTTCTCTTTTCCCTTTGGCACATGTATTAGATAGGTGGATGGTAAGGTCTGGAAAAACATCCACACTTTATGACAAGGA

CGGACATATATGTATATTTTTCAATAATTATTCAACAGAA

>Cbur04G014840.1 gene=Cbur04G014840

ATGGCTCCAGCTGCTGCTACAGATAATATAAAACCTCGAGATGTATGCATTGTTGGTGTTGCACGCACACCGATGGGAGG

ATTCCTTGGCACCCTTTCCTCCTTACCTGCCACTAAATTAGGTTCTATTGTAATTGAGTGTGCTCTTAAAAGGGCACATG

TTCATGCCAACCTTGTACAAGAGGTCTTCTTTGGAAATGTTCTGAGTGCAAATTTGGGGCAGGCTCCTGCTAGGCAGGCT

GCCTTAGGTGCAGGAATCCCTAACTCAGTTGTTTGCACCACTATTAATAAAGTCTGTTCTTCAGGAATGAAAGCAACAAT

GCTTGCAGCCCAAAGTATCCAGTTGGGTATTAATGATGTTGTTGTGGCTGGTGGCATGGAAAGCATGTCTAATGCGCCTA

AATATCTGGCAGAGACAAGGAAAGGCTCTCGTTTGGGGCATGACATGGTTATTGATGGGATGCTTAAAGATGGTCTATGG

GATGTTTATAATGATTATGCTATGGGAATGTGTGCTGAGCTCTGTGCAGATCAACATACAATAACCAGAGAAGAGCAGGA

TTCTTTTGCCATTCAAAGTTTTGAGCGTGGTATTGCTGCTCAAAATAGCGGCGCGTTTGCTTGGGAAATTGCACCGGTCA

GAATTCTGTTGAAGTTCCTGGGGGTAGAGGGAAGCCATCCTATACTTGTTGATAAGGATGAAAGCCTTGAAAAATTTGAT

CCTGCCAAACTAAGGAAGCTTCGGCCAAATTTTAAGGAGGTTGGTGGTACTGTTACTGCTGGCAATGCCTCTATTATAAG

TGATGGTGCTGCTGCATTAGTGTTGGTCAGTGGACAGAAAGCTCTTGAACTAGGACTGCAGGTGATTGCGAAGATTGCTG

GATATGCCGATGCAGCTCAGGCACCAGAGTGGTTCACAACTGCCCCAGCACTTGCAATACCAAAAGCCATTTCAAATGCT

GGATTGGAGGCTTCTCAAATTGATTTCTATGAGATAAATGAGGCATTCTCTGTTGTGGCTCTTGTAAATCAGAAAATTCT

GGGACTTCATCCAGAAAAACTTAATGCGCATGGTGGAGCAGTGTCCTTGGGGCATCCTCTAGGCTGTAGTGGAGCTCGTA

TTTTGGTCACACTTCTGGGGGTGTTGAGACAGAGGAATGGGAAATATGGAGTTGCTGGTGTTTGCAATGGAGGCGGTGGA

GCATCTGCTCTTGCTCTAGAGCTCATAAAAAAACCGTCCTTGCTCTGGTATGATAGGAACATACAAATCTATTCAATTGC

TGTTTCCAGTTTCTCCATTACAGCCAAGTAA

>Cbur04G014790.1 gene=Cbur04G014790

ATGAAAGCAACAATGCTTGCCGCCCAAAGTATCCAGTTGGGTATTAAAGATGTTGTTGTGGCTTGTGGCATGGAAAGCAT

GTCTGATGCTCCTAAATATCTGGCAGAGACAAGGTTAGGTTTCTTTTTCTTAGGCAACATTAATTTGTTTAATATTTTTT

TTCTGGGTCCCGTAGAAAGCTTCAATGAAGGGAGAGAATATTTTACAACTAAAAGGGAACTGTGGTTGTTTAAAGAAACT

ATGGAAAAGAAAAACCTTACACAAGATTTTCTGATGAAAGTTATGCTAGTAAACCTTACATTCTAA
